# OATP1A2 mRNA downregulation in canine hepatocellular carcinoma

**DOI:** 10.1002/vro2.70038

**Published:** 2026-07-06

**Authors:** Shohei Kurokawa, Tomoki Motegi, Hideo Akiyoshi, Toshiyuki Tanaka

**Affiliations:** ^1^ Senri Momoyamadai Animal Hospital Osaka Japan; ^2^ Laboratory of Veterinary Molecular Pathophysiology Treatment Cooperative Department of Veterinary Medicine Faculty of Agriculture Tokyo University of Agriculture and Technology Tokyo Japan; ^3^ Neovets Veterinary Referral Center Osaka Japan; ^4^ Laboratory of Veterinary Advanced Diagnosis and Treatment School of Veterinary Science Osaka Metropolitan University Osaka Japan

**Keywords:** hepatocellular adenoma, magnetic resonance imaging, transporter

## Abstract

**Background:**

On gadolinium ethoxybenzyl diethylenetriamine pentaacetic acid (Gd‐EOB‐DTPA) magnetic resonance imaging, canine hepatocellular carcinoma (HCC) and hepatocellular adenoma (HCA) typically exhibit low and high signal intensities in the hepatobiliary phase, respectively. Reduced transporter protein expression is associated with decreased Gd‐EOB‐DTPA uptake. However, the specific transporters involved in Gd‐EOB‐DTPA uptake in canine HCC remain unknown. This exploratory study aimed to assess the mRNA expression of transporters that are downregulated in canine HCC.

**Methods:**

For the bioinformatics analysis, an RNA sequencing dataset comprising 14 canine HCC samples and four normal liver samples was obtained from the DDBJ BioProject repository. Differentially expressed genes (DEGs) were evaluated for members of the organic anion transporting polypeptides (OATP1‐6) family, multidrug resistance‐associated protein 2 (MRP2), bile salt export pump (BSEP), breast cancer resistance protein (BCRP), organic cation transporter 1 (OCT1) and multidrug resistance‐associated protein 3 (MRP3) in comparison between HCC and normal liver tissues. Genes with an absolute log‐fold change greater than 1 and a nominal *p*‐value less than 0.05 were considered candidate DEGs. For clinical samples, surgically resected samples from eight canine HCCs and four canine HCAs were evaluated. RNA was extracted from the samples, and the expression levels of OATP1A2 were compared between HCCs and HCAs using real‐time quantitative polymerase chain reaction.

**Results:**

In bioinformatics analysis, upregulated DEGs in HCC included OATP3A1/*SLCO3A1* and OATP5A1/*SLCO5A1*, whereas downregulated DEGs included OATP1A2/*SLCO1A2*. In clinical samples, OATP1A2 mRNA expression was significantly lower in HCC than in HCA (*p* = 0.01).

**Conclusion:**

Downregulation of OATP1A2 is associated with canine HCC and may contribute to reduced Gd‐EOB‐DTPA uptake.

## INTRODUCTION

In canines, gadolinium ethoxybenzyl diethylenetriamine pentaacetic acid (Gd‐EOB‐DTPA) magnetic resonance imaging (MRI) is used to differentiate hepatocellular carcinoma (HCC). During the hepatobiliary phase, hepatocyte‐specific uptake of Gd‐EOB‐DTPA occurs; canine HCC typically exhibit low signal intensity, whereas benign tumours, such as hyperplasia and hepatocellular adenoma (HCA), show high signal intensity.^1^, ^2^, ^3^ In humans, Gd‐EOB‐DTPA transport is mediated by organic anion transporting polypeptides (OATP1B1 and OATP1B3) and multidrug resistance‐associated proteins (MRP2 and MRP3).^4^ Transporter expression decreases with increasing HCC grade, corresponding to reduced Gd‐EOB‐DTPA enhancement of lesions.^5^ Moreover, malignant transformation of hepatocytes alters the expression patterns of certain OATPs.^6^

However, the mechanisms underlying Gd‐EOB‐DTPA uptake and hepatobiliary excretion in dogs remain unclear. In normal canine liver, OATPs (including OATP1A2, OATP2B1 and OATP1B4) are highly expressed, followed by MRP2, bile salt export pump (BSEP), breast cancer resistance protein (BCRP), organic cation transporter 1 (OCT1) and MRP3.^7^ The expression profiles of these transporters in canine HCC have not been fully characterised. We hypothesised that reduced transporter expression contributes to decreased Gd‐EOB‐DTPA uptake in HCC. Accordingly, this exploratory study aimed to identify transporters that are downregulated in canine HCC. We first analysed publicly available RNA sequencing (RNA‐seq) data to identify candidate transporters. We then compared the expression levels of the selected transporter mRNAs between clinically collected HCC and HCA tissues.

## MATERIALS AND METHODS

For the bioinformatics analysis, the RNA‐seq dataset was obtained from the DDBJ BioProject repository (accession numbers PRJDB 18013 and PRJDB 20428). The dataset comprised 14 canine HCCs and four normal liver samples. Gene expression profiles were reprocessed using a previously described pipeline.^8^ RNA sequencing reads were aligned to the canine reference genome following the same protocol. Gene expression levels were normalised, and differential expression analysis was performed using the parameters reported in the original study. Differentially expressed genes were evaluated for members of the OATP1‐6 family, MRP2, BSEP, BCRP, OCT1 and MRP3 in comparison between HCC and normal liver tissues. Genes with an absolute log‐fold change greater than 1 and a nominal *p*‐value less than 0.05 were considered candidate DEGs.

For the clinical samples, informed consent was obtained from all patients for the collection and use of HCC and HCA tissues for research and publication. All diagnostic procedures and treatments were conducted as part of routine clinical care, and the study did not meet the criteria for submission to a local ethics and welfare committee; therefore, formal ethical approval or exemption was not obtained.

Clinical specimens were collected from eight dogs with histopathologically confirmed HCC and four dogs diagnosed with HCA that underwent surgical resection at Osaka Metropolitan University between 2024 and 2025. Tissue samples were collected immediately after surgery.

Total RNA was extracted from liver tissues using a commercial RNA extraction kit (NucleoSpin RNA Plus, Takara Bio) according to the manufacturer's instructions. RNA quality and concentration were assessed by measuring absorbance at 260/280 nm. Complementary DNA was synthesised using a reverse transcription kit (ReverTra Ace, TOYOBO Co.) following the manufacturer's protocol. Real‐time quantitative polymerase chain reaction (RT‐qPCR) was performed using gene‐specific primers for OATP1A2 (forward: 5′‐GGT TAC ATA CAT TTT GCA C‐3′, reverse: 5′‐CAA GCC AAG GTA GAT GTA TC‐3′) and GAPDH (forward: 5′‐TGC TGG TGC TGA GTA TGT TG‐3′, reverse: 5′‐CGA AGT GGT CAT GGA TGA CT‐3′). The OATP1A2 primers were designed based on a previous report,^9^ and GAPDH served as the reference gene. Real‐time quantitative polymerase chain reaction was performed using SYBR Green chemistry on a real‐time PCR system, with each sample analysed in duplicate. ΔCt values are presented as mean ± 95% confidence interval (CI). Relative gene expression levels were calculated using the 2^−ΔΔCt^ method, with the HCA group as the reference. Statistical analyses were performed using R (version 2.12.1; R Foundation for Statistical Computing). Data normality was assessed using the Shapiro–Wilk test, which indicated that parametric testing was required. Homogeneity of variances was evaluated using Levene's test. Differences in OATP1A2 mRNA expression (ΔCt values) between groups were analysed using Student's *t*‐test. Statistical significance was defined as *p*‐value of less than 0.05.

## RESULTS

### Dogs

The HCC group comprised four neutered males, one intact male and three neutered females, with a mean age of 11.4 ± 2.0 years (mean ± standard deviation). Breeds included two Shiba Inu, two Toy Poodles, one Dachshund, one Shih Tzu, one Chihuahua and one Shetland Sheepdog. The HCA group consisted of two neutered males and two neutered females, with a mean age of 12 ± 1.6 years. Breeds represented were Shiba Inu, Chihuahua, Yorkshire Terrier and Greyhound.

### Bioinformatics analysis

A total of 2297 DEGs met the predefined cutoff criteria, including 1397 upregulated and 900 downregulated genes relative to normal liver tissue. Among these, three DEGs belonging to the OATP family were identified in HCC: OATP3A1/*SLCO3A1* and OATP5A1/*SLCO5A1* were upregulated, whereas OATP1A2/*SLCO1A2* was downregulated. Table 1 summarises the identified DEGs. False discovery rate‐adjusted *p*‐values (*q*‐values) are provided in the Supporting Information.

**TABLE 1:** Differentially expressed genes associated with transporters in hepatocellular carcinoma.

Gene	Log‐fold change	Nominal *p*‐value	Regulation
OATP3A1 (*SLCO3A1*)	2.07	0.02	Up
OATP1A2 (*SLCO1A2*)	−2.08	0.03	Down
OATP5A1 (*SLCO5A1*)	4.21	0.04	Up

### Gene‐expression levels

**FIGURE 1:**
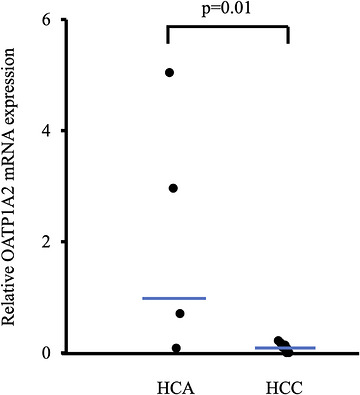
OATP1A2 mRNA expression in hepatocellular adenoma (HCA) and hepatocellular carcinoma (HCC). Relative OATP1A2 mRNA expression is significantly lower in HCC than in HCA (*p* = 0.01).

Based on the bioinformatic analysis, OATP1A2 was selected as the target gene for further investigation. The mean ΔCt values (±95% CI) for OATP1A2 mRNA were 3.19 ± 1.35 in HCC and −0.57 ± 4.09 for HCA. The relative expression of OATP1A2 mRNA in HCC (mean ± 95% CI: 0.11 ± 0.06) was significantly lower than in HCA (*p* = 0.01). Figure 1 shows the mRNA expression levels of OATP1A2 mRNA.

## DISCUSSION

In canines, the primary hepatic uptake transporter is OATP1B4, which is homologous to human OATP1B1/OATP1B3 and is highly expressed in normal hepatocytes.^9^ However, the uptake clearance of OATP1B4 substrates correlated more closely with OATP1B3 than with the more abundant hepatic analogue, OATP1B1.^9^ Efflux from hepatocytes into bile is primarily mediated by MRP2.^7^ Although we evaluated the expression of several hepatic transporters, including OATP1B4 and MRP2, using bioinformatics analysis, no significant differences in OATP1B4 mRNA or MRP2 mRNA expression were observed between HCCs and normal liver samples. In contrast, this study demonstrated downregulation of OATP1A2 mRNA in canine HCC, which may reduce Gd‐EOB‐DTPA uptake.^1^, ^2^, ^3^

OATP1A2 is the third most highly expressed transporter in canine liver. In humans, OATP1A2 is not liver specific, and the relative abundance of OATP transporters in liver tissue differs markedly between species, accounting for approximately 29% in humans and 69% in dogs.^7^ These differences suggest that OATP1A2 may play a more prominent role in hepatic drug uptake in canines. However, its specific function in the canine liver remains unclear. Our findings indicated that OATP1A2 may be associated with Gd‐EOB‐DTPA uptake and particularly decrease in HCC among canine hepatocellular neoplasia.

In this study, HCA was used as a comparator for HCC because surgically resected clinical samples were available. In humans, approximately 89% of HCAs exhibit low signal intensity during the hepatobiliary phase of MRI.^10^ Although malignant transformation of HCA is rare, lesions at higher risk of malignant transformation may exhibit high or isointense signals during the hepatobiliary phase.^11^, ^12^, ^13^ In canines, HCA is a benign tumour, and most cases show high signal intensity during the hepatobiliary phase; however, at least one case with low signal intensity has been reported.^14^ Because HCA represents neoplastic rather than normal liver tissue, this tumour‐to‐tumour comparison does not directly establish downregulation relative to a physiological baseline. This limitation should be considered when interpreting the results. Furthermore, OATP1A2 has not been definitively established as a major transporter responsible for Gd‐EOB‐DTPA uptake, and Gd‐EOB‐DTPA‐enhanced MRI was not performed in this study. Therefore, additional studies are needed to assess the relationship between OATP1A2 expression and hepatobiliary phase signal intensity in both HCC and HCA.

Bioinformatics analysis also indicated upregulation of OATP3A1 and OATP5A1 in canine HCC. Consistent with previous findings, mRNA levels of OATP2A1, OATP3A1, OATP4A1 and OATP5A1 are increased in HCC.^6^ Transporter expression profiles in canine HCC differ from those in humans, depending on the OATP family. These upregulated transporters may represent potential targets for anticancer drug development.^6^ Accordingly, further investigation of OATP3A1 and OATP5A1 expression in canine HCC may facilitate the identification of novel therapeutic strategies. Therefore, downregulation of OATP1A2 is associated with canine HCC and may contribute to reduced Gd‐EOB‐DTPA uptake.

## AUTHOR CONTRIBUTIONS

Shohei Kurokawa and Toshiyuki Tanaka served as principal investigators and conceived the study. Toshiyuki Tanaka supervised the surveillance components. Shohei Kurokawa and Toshiyuki Tanaka performed the specimen sampling and RNA isolation. Tomoki Motegi validated and analysed the RNA‐seq data. Shohei Kurokawa, Tomoki Motegi, Hideo Akiyoshi and Toshiyuki Tanaka interpreted the RNA‐seq data. Shohei Kurokawa prepared the initial draft, figures and tables. All the authors contributed to manuscript writing and editing and approved the final version. The manuscript was prepared following the STROBE statement.

## CONFLICTS OF INTEREST

The authors declare they have no conflicts of interest.

## ETHICS STATEMENT

Not applicable.

## Supporting information



Supporting Information

## Data Availability

The data supporting the findings of this study are available from the corresponding author upon reasonable request. The datasets are deposited in the DDBJ BioProject repository under accession numbers PRJDB 18013 and PRJDB 20428 (https://ddbj.nig.ac.jp/search/entry/bioproject/PRJDB18013 and https://ddbj.nig.ac.jp/search/entry/bioproject/PRJDB20428).
