# Supplementary material for: OATP1A2 mRNA downregulation in canine hepatocellular carcinoma
Source: Vet Rec Open. 2026 Jul 6;13(2):e70038. doi: 10.1002/vro2.70038 (PMC13334214; doi:10.1002/vro2.70038)
Supplement: Supplementary file 1 — Supporting Information [file VRO2-13-e70038-s001.pdf]

Candidate DEGs with nominal p-values < 0.05

Ensembl gene ID	Gene Symbol	NL		
		NL2	NL3	NL4
ENSCAFG00000045881		5.660962409	35.77670702	28.39604436
ENSCAFG00000016090	TOP2A	18.11507971	11.24410792	7.09901109
ENSCAFG00000012593	CENPF	4.528769927	4.088766517	4.73267406
ENSCAFG00000047783		49.47681145	126.1588909	71.98397245
ENSCAFG00000029840		0	23.12197465	14.88425992
ENSCAFG00000040843		20.37946467	7.155341405	9.46534812
ENSCAFG00000001738	GRM8	2.264384964	38.84328191	18.93069624
ENSCAFG00000012140	PRC1	3.396577445	5.110958146	2.36633703
ENSCAFG00000003243	ANLN	3.396577445	2.044383259	1.183168515
ENSCAFG00000013327	APOA5	33926.14772	9779.307317	20901.85499
ENSCAFG00000004133	CCNA2	3.396577445	0	1.183168515
ENSCAFG00000011403	ASPM	4.528769927	2.044383259	3.549505545
ENSCAFG00000018381	ESM1	2.264384964	7.155341405	2.36633703
ENSCAFG00000009538	NUSAP1	4.494804153	4.088766517	7.09901109
ENSCAFG00000007075	TPX2	9.057539854	10.22191629	3.549505545
ENSCAFG00000014650	CDCA3	3.396577445	1.022191629	3.549505545
ENSCAFG00000032722	PRIMA1	292.1056603	82.79752197	194.0396365
ENSCAFG00000012136	NEK2	3.396577445	1.022191629	1.183168515
ENSCAFG00000005259	CDC20	13.58630978	3.066574888	3.549505545
ENSCAFG00000013658	NR0B1	12.4541173	7.155341405	2.36633703
ENSCAFG00000009633	CBR3	309.1904448	12.31740913	386.7304608
ENSCAFG00000008369	PBK	0	2.044383259	0
ENSCAFG00000008378	ESCO2	1.132192482	4.088766517	1.183168515
ENSCAFG00000020429	DEPDC1	0	1.022191629	1.183168515
ENSCAFG00000008877	BUB1B	5.660962409	1.022191629	4.73267406
ENSCAFG00000008236	ARHGAP11A	5.660962409	8.177533034	4.73267406
ENSCAFG00000007765	TTC29	4.528769927	0	18.93069624
ENSCAFG00000023690	ERCC6L	0	0	0
ENSCAFG00000013322	APOC3	35667.45975	55860.72816	75884.87904
ENSCAFG00000017658	PLK1	7.925347372	3.066574888	4.73267406
ENSCAFG00000009745	UBE2C	3.396577445	2.044383259	1.183168515
ENSCAFG00000007705		1.132192482	3.066574888	3.549505545
ENSCAFG00000030804	RNLS	575.1537807	655.2248344	698.0694238
ENSCAFG00000009202	KNL1	4.528769927	1.022191629	1.183168515

ENSCAFG00000016831	KIF4A	4.528769927	7.155341405	3.549505545
ENSCAFG00000016677	NCAPG	4.528769927	2.044383259	7.09901109
ENSCAFG00000033956		902.357408	386.3884359	332.4703527
ENSCAFG00000014112	KIF15	7.925347372	6.133149776	3.549505545
ENSCAFG00000000255	B4GALNT1	52.08085416	58.26492287	31.9455499
ENSCAFG00000007497	KIF11	6.793154891	4.088766517	4.73267406
ENSCAFG00000015026	DLGAP5	2.264384964	3.066574888	2.36633703
ENSCAFG00000004052	MCM2	18.11507971	8.177533034	7.09901109
ENSCAFG00000013173	STRC	1232.957613	63.37588101	380.9802618
ENSCAFG00000015455	ECT2	7.925347372	15.33287444	1.183168515
ENSCAFG00000003539	CLSPN	12.4541173	3.066574888	2.36633703
ENSCAFG00000018292	ZFYVE21	2001.716308	1666.172356	1133.475437
ENSCAFG00000001765	SPAM1	2.264384964	21.46602421	2.36633703
ENSCAFG00000029919	CLIC3	0	0	2.36633703
ENSCAFG00000003409	RRM2	10.18973234	4.088766517	0
ENSCAFG00000000987	KIFC1	13.58630978	5.110958146	13.01485366
ENSCAFG00000029785	AS3MT	1905.479947	1620.173732	1051.83681
ENSCAFG00000007030	ESPL1	4.528769927	2.044383259	2.36633703
ENSCAFG00000042313	CENPT	0	0	0
ENSCAFG00000002081	CDC37L1	635.1599823	527.4508807	423.5743284
ENSCAFG00000011909	CAMK1G	11.32192482	16.35506607	41.41089802
ENSCAFG00000018724	SPAG5	3.396577445	1.022191629	2.36633703
ENSCAFG00000001545	TTC39B	73.59251132	88.93067175	31.9455499
ENSCAFG00000016010	FSCN1	53.11114932	30.21598456	36.67822396
ENSCAFG00000004421	CDADC1	1602.052362	1220.496805	682.6882331
ENSCAFG00000045478		4.528769927	1.022191629	2.36633703
ENSCAFG00000049840		0	0	3.549505545
ENSCAFG00000002196	MCM3	35.09796694	53.15396472	41.41089802
ENSCAFG00000018395	NDC80	1.132192482	1.022191629	1.183168515
ENSCAFG00000008340	RACGAP1	18.11507971	16.35506607	21.29703327
ENSCAFG00000001702	NMRK1	300.0310077	787.0875545	825.8516235
ENSCAFG00000007062	BUB1	7.925347372	1.022191629	4.73267406
ENSCAFG00000020345	PTGFR	20.37946467	8.177533034	0
ENSCAFG00000029325		33.96577445	16.35506607	11.83168515
ENSCAFG00000013664	TERB2	33.96577445	0	0
ENSCAFG00000004770	KIF2C	6.793154891	4.088766517	2.36633703
ENSCAFG00000029191	STMN1	50.94866168	19.42164096	27.21287584

ENSCAFG00000036553		2.264384964	2.044383259	1.183168515
ENSCAFG00000005820	ACSS3	1851.134708	3041.020097	2707.089562
ENSCAFG00000002174	GADD45G	2076.441012	1422.890748	1203.28238
ENSCAFG00000003278	CDCA8	0	0	2.36633703
ENSCAFG00000000040	CCDC102B	43.02331431	8.177533034	5.915842575
ENSCAFG00000019372	CCNF	2.264384964	0	5.915842575
ENSCAFG00000001720	C11H9orf72	423.4399882	580.6048454	389.2624414
ENSCAFG00000020084		99.6329384	1.022191629	3.549505545
ENSCAFG00000002842	TTK	1.132192482	1.022191629	0
ENSCAFG00000044628		1.132192482	1.022191629	0
ENSCAFG00000013600	PTH1R	628.3668274	308.701872	384.5297674
ENSCAFG00000013255	MKI67	22.64384964	1.022191629	10.64851663
ENSCAFG00000016958	KIF22	6.793154891	5.110958146	3.549505545
ENSCAFG00000013672	SORD	5346.212899	10113.56398	19173.24579
ENSCAFG00000014653	G6PC	25845.68997	9435.85093	4675.881971
ENSCAFG00000001204	KIF20A	4.528769927	5.110958146	0
ENSCAFG00000014295	SMC4	151.7137926	164.5728523	114.767346
ENSCAFG00000020091	SLC25A25	4486.878805	1525.109911	223.6188493
ENSCAFG00000009418	TP53INP1	293.2378528	107.3301211	67.44060535
ENSCAFG00000008962	CDCA2	4.528769927	0	2.36633703
ENSCAFG00000039915		2.264384964	1.022191629	0
ENSCAFG00000044594		287.5768904	4.088766517	46.14357208
ENSCAFG00000007429	NECAB3	5.660962409	1.022191629	3.549505545
ENSCAFG00000017535	KIF23	4.528769927	3.066574888	2.36633703
ENSCAFG00000007837	KNTC1	5.660962409	4.088766517	4.73267406
ENSCAFG00000033502		156.2425625	72.57560568	101.7524923
ENSCAFG00000008591	TDO2	5419.80541	16400.0425	9084.367858
ENSCAFG00000008822	CLDN8	16.98288723	0	0
ENSCAFG00000016106	CDC6	3.396577445	0	1.183168515
ENSCAFG00000003853	SLC26A3	0	3.066574888	2.36633703
ENSCAFG00000002661	SMC2	18.11507971	57.24273124	36.67822396
ENSCAFG00000005143	MCM6	61.13839402	57.24273124	46.14357208
ENSCAFG00000030087		14.71850226	7.155341405	3.549505545
ENSCAFG00000009220	RAD51	3.396577445	2.044383259	1.183168515
ENSCAFG00000005187	EDNRB	350.9796694	246.3481827	205.8713216
ENSCAFG00000040565		11.32192482	2.044383259	2.36633703
ENSCAFG00000046787		65.66716394	5.110958146	3.549505545

ENSCAFG00000048209		50.94866168	21.46602421	14.19802218
ENSCAFG00000015758	EXO1	1.132192482	0	0
ENSCAFG00000048907	DOLK	56.60962409	67.46464753	59.15842575
ENSCAFG00000013659	EIF4A2	7355.854554	10894.51838	8840.635144
ENSCAFG00000017744	AK7	46.41989175	69.50903079	68.62377387
ENSCAFG00000009098	KNSTRN	5.660962409	4.088766517	1.183168515
ENSCAFG00000004902	DIAPH3	7.925347372	3.066574888	0
ENSCAFG00000010654	AGPAT3	517.4119642	529.495264	1041.188293
ENSCAFG00000015888	INCENP	40.75892934	47.02081495	50.87624614
ENSCAFG00000044726		374.7557115	143.1068281	163.2772551
ENSCAFG00000047549		6.793154891	1.022191629	0
ENSCAFG00000030105	IL33	3409.031563	2654.631661	493.3812707
ENSCAFG00000009027	FAM83D	2.264384964	4.088766517	0
ENSCAFG00000003594	IRF3	2514.599502	2457.348677	2672.777675
ENSCAFG00000003725	NOCT	1149.175369	208.5270924	185.7574569
ENSCAFG00000002878		3.396577445	10.22191629	0
ENSCAFG00000013980	CDCA5	1.132192482	1.022191629	0
ENSCAFG00000030137	RGS13	4.528769927	1.022191629	0
ENSCAFG00000000771	AGPAT1	92.83978351	67.46464753	97.01981823
ENSCAFG00000011140	KIF14	2.264384964	1.022191629	0
ENSCAFG00000029331	AURKA	18.11507971	26.57698236	15.38119069
ENSCAFG00000010836	CENPE	16.98288723	17.3772577	10.64851663
ENSCAFG00000007735	CENPU	0	2.044383259	0
ENSCAFG00000029929		2.977666227	2.044383259	9.46534812
ENSCAFG00000017264		1.132192482	3.066574888	0
ENSCAFG00000012260	DTL	3.396577445	0	0
ENSCAFG00000019338	PKMYT1	1.132192482	0	2.36633703
ENSCAFG00000014767	SLC9A7	73.59251132	72.57560568	68.62377387
ENSCAFG00000005182	FANCD2	18.55663478	26.17832763	13.41713096
ENSCAFG00000018564	GRIA3	14.71850226	27.59917399	30.76238139
ENSCAFG00000013287	MAPK4	18.11507971	12.26629955	18.93069624
ENSCAFG00000013149	WHAMM	309.0885475	107.3301211	117.133683
ENSCAFG00000028905	CDKN2C	10.18973234	11.24410792	5.915842575
ENSCAFG00000018342	ADSSL1	89.44320606	37.82109028	97.01981823
ENSCAFG00000031706		60.00620153	37.22821914	75.72278496
ENSCAFG00000013645		31.70138949	3.066574888	2.36633703
ENSCAFG00000016822	NEURL1B	5.660962409	13.28849118	10.64851663

ENSCAFG00000010135	LUZP2	32.83358197	29.64355725	1.183168515
ENSCAFG00000012146	SCN4A	140.3918677	24.5325991	117.133683
ENSCAFG00000018934	UHRF1	4.528769927	4.088766517	8.282179605
ENSCAFG00000024944		11.32192482	6.133149776	7.09901109
ENSCAFG00000032243	DIO2	986.1396516	403.7656936	41.41089802
ENSCAFG00000016196	GDF10	19.24727219	6.133149776	1.183168515
ENSCAFG00000005245	ELOVL1	55.47743161	47.02081495	62.70793129
ENSCAFG00000003785	PCDH10	0	0	0
ENSCAFG00000013713		1118.606172	966.9932813	2000.737959
ENSCAFG00000014435	MAOA	58.87400905	50.08738983	84.00496456
ENSCAFG00000009799	NCAPD3	11.32192482	22.48821584	14.19802218
ENSCAFG00000013459	KLRD1	23.77604212	6.133149776	11.83168515
ENSCAFG00000016616	IQGAP3	1.132192482	1.022191629	1.183168515
ENSCAFG00000049439		78.81191866	56.22053961	26.65678664
ENSCAFG00000013306	APOA4	421.1756032	54.17615635	4743.322577
ENSCAFG00000019268	SLX4	28.30481204	16.35506607	31.9455499
ENSCAFG00000001067	RSPO3	117.7480181	53.15396472	117.133683
ENSCAFG00000015124	NCAPD2	83.78224365	57.24273124	93.47031268
ENSCAFG00000000244	MTFR2	5.660962409	3.066574888	5.915842575
ENSCAFG00000012443	CMA1	192.4727219	72.57560568	68.62377387
ENSCAFG00000044045		11.32192482	0	0
ENSCAFG00000025410	FABP4	0	0	0
ENSCAFG00000000972	TAPBP	1178.612374	445.6755504	1217.480402
ENSCAFG00000031967	GCSAML	4.528769927	14.31068281	1.183168515
ENSCAFG00000013297	IGF2BP2	13.58630978	10.22191629	13.01485366
ENSCAFG00000049897		2.264384964	13.28849118	65.07426832
ENSCAFG00000006062	COL4A1	504.9578469	207.5049007	451.9703727
ENSCAFG00000013954	KIF18B	0	0	1.183168515
ENSCAFG00000013006	ITGA6	11.32192482	7.155341405	13.01485366
ENSCAFG00000002334	MELK	3.396577445	1.022191629	2.36633703
ENSCAFG00000016083	AKAP8	241.3494713	239.7959343	336.5522841
ENSCAFG00000003683	ORC6	2.264384964	2.044383259	1.183168515
ENSCAFG00000000443	TUBB	517.4119642	453.8530834	567.9208872
ENSCAFG00000010352	MS4A2	167.5644873	52.13177309	50.87624614
ENSCAFG00000007520	CBR4	300.0310077	468.1637662	461.4357208
ENSCAFG00000014670	NID2	29.43700453	10.22191629	5.915842575
ENSCAFG00000016522	PLOD1	598.9298229	423.1873345	722.9159626

ENSCAFG00000003363	CHRM2	6.793154891	10.22191629	4.73267406
ENSCAFG00000025075	DEFB1	1326.929589	2135.358314	3760.109541
ENSCAFG00000045177		0	0	0
ENSCAFG00000006909	LMO1	21.51165715	10.22191629	16.56435921
ENSCAFG00000001709	MCM5	22.64384964	7.155341405	22.48020178
ENSCAFG00000009053	CKAP5	106.4260933	109.3745043	154.9950755
ENSCAFG00000012975		422.3077957	234.0818831	377.4307563
ENSCAFG00000015377	KIAA0586	57.74181657	82.79752197	85.18813308
ENSCAFG00000017275	FABP6	30.56919701	16.35506607	3.549505545
ENSCAFG00000004457	CENPA	0	0	1.183168515
ENSCAFG00000019132	RPH3AL	38.49454438	27.59917399	59.15842575
ENSCAFG00000031936	NRBF2	501.5612694	350.6117288	231.9010289
ENSCAFG00000014440	SLITRK3	2.264384964	2.044383259	1.183168515
ENSCAFG00000017254	ATP2A1	21.51165715	45.99862332	28.39604436
ENSCAFG00000004604	TMEM104	44.15550679	19.42164096	30.76238139
ENSCAFG00000019880	GPSM2	20.90027321	44.46533587	32.430649
ENSCAFG00000012459	HORMAD2	3.396577445	43.95424006	10.64851663
ENSCAFG00000018996	CDH10	10.18973234	2.044383259	5.915842575
ENSCAFG00000014758		0	3.802552861	2.248020178
ENSCAFG00000009616	SETD4	97.36855343	119.5964206	81.63862753
ENSCAFG00000017892	DNMT1	61.13839402	66.4424559	100.5693238
ENSCAFG00000041946	IGHM	285.3125054	180.9279184	293.4257917
ENSCAFG00000001480	TRIOBP	176.6220272	173.772577	215.3366697
ENSCAFG00000050044		5.638318559	2.044383259	0
ENSCAFG00000015862	FAM214A	1437.884452	1160.187499	1346.44577
ENSCAFG00000016686	ANXA2	499.2968845	353.6783037	141.9802218
ENSCAFG00000005294	TK1	88.31101358	68.48683916	86.37130159
ENSCAFG00000049434	SIAH2	790.2703523	475.3191076	709.901109
ENSCAFG00000036277		3.396577445	1.022191629	1.183168515
ENSCAFG00000003807	KLF15	1454.867339	1418.801981	1458.846779
ENSCAFG00000003875	PLK4	0	2.044383259	0
ENSCAFG00000003704	SHCBP1	3.396577445	25.55479073	5.915842575
ENSCAFG00000017900	SLC25A47	6729.752112	2315.26404	4164.753173
ENSCAFG00000009431	E2F8	4.528769927	2.044383259	0
ENSCAFG00000017482	ITGA11	11.32192482	1.022191629	3.549505545
ENSCAFG00000003685	CREM	348.7152844	234.0818831	147.8960644
ENSCAFG00000010412		1.132192482	3.066574888	0

ENSCAFG00000005354	KLF6	1709.610647	2535.035241	589.2179205
ENSCAFG00000013710	DNA2	0	0	1.183168515
ENSCAFG00000005806	SGO1	2.264384964	1.022191629	0
ENSCAFG00000031624	ZNF529	3.396577445	9.199724663	8.282179605
ENSCAFG00000014897	CDKN3	0	0	0
ENSCAFG00000045221		1.132192482	0	0
ENSCAFG00000020188	TAT	24951.25791	25714.25263	15800.03235
ENSCAFG00000002123	PON2	535.5270439	861.7075435	1069.584338
ENSCAFG00000011598		118.7103817	105.2039625	89.22273771
ENSCAFG00000009336	MYBL2	7.925347372	1.022191629	5.915842575
ENSCAFG00000011069	GJA5	5.660962409	15.33287444	7.09901109
ENSCAFG00000013622		4.528769927	6.133149776	2.36633703
ENSCAFG00000015521	ZWINT	5.660962409	6.133149776	1.183168515
ENSCAFG00000008940	HNRNPDL	2479.59211	1213.832116	1897.991605
ENSCAFG00000002628	EPS8L1	5.660962409	11.24410792	3.549505545
ENSCAFG00000007344	MYBL1	2.264384964	5.110958146	7.09901109
ENSCAFG00000008860	PAQR3	112.1096995	123.6851871	98.20298674
ENSCAFG00000018832	TUBB6	148.2266397	44.85376869	42.64139328
ENSCAFG00000001046	DESI1	86.04662862	85.86409686	63.89109981
ENSCAFG00000010596		1106.152055	245.325991	211.7871642
ENSCAFG00000009168	ARSB	23.77604212	15.33287444	26.02970733
ENSCAFG00000008889	BMP3	0	0	2.36633703
ENSCAFG00000004629		255.3660143	491.8581682	239.0946935
ENSCAFG00000000697		31.70138949	10.22191629	63.89109981
ENSCAFG00000030089		29.43700453	22.48821584	20.11386475
ENSCAFG00000006411	SMPD1	394.0029837	232.0374998	395.178284
ENSCAFG00000008737	RPN2	2086.630744	1221.518997	1581.896305
ENSCAFG00000011899	HJURP	5.660962409	4.088766517	4.73267406
ENSCAFG00000002348	COL21A1	69.06374139	39.86547354	73.35644793
ENSCAFG00000006767	PRODH2	5928.159835	4946.385294	5062.778076
ENSCAFG00000005217	NCAPG2	4.528769927	13.28849118	4.73267406
ENSCAFG00000011755	FCER1A	72.46031883	62.35368939	31.9455499
ENSCAFG00000005997	GPCPD1	512.8831942	600.0264864	293.4257917
ENSCAFG00000002618	TNNI3	21.51165715	34.7545154	39.04456099
ENSCAFG00000008613	TROAP	0	0	0
ENSCAFG00000010162	MUC15	7.925347372	3.066574888	0
ENSCAFG00000005747	TCIM	859.3340937	363.90022	250.8317252

ENSCAFG00000017920	ANGPTL6	1056.335586	439.5424006	447.2376987
ENSCAFG00000016946	SLC35B1	396.2673686	253.5035241	255.5643992
ENSCAFG00000012836	ADAMTS4	6.793154891	0	2.36633703
ENSCAFG00000007441	SLC35G2	5.660962409	12.26629955	9.46534812
ENSCAFG00000015323	PLIN4	2.264384964	1.022191629	3.549505545
ENSCAFG00000003744	ORC1	0	0	3.549505545
ENSCAFG00000007890	CDKN2AIP	391.7385987	312.7906386	139.6138848
ENSCAFG00000016193	GDF2	268.3296182	57.24273124	157.3614125
ENSCAFG00000016889	ARHGEF2	67.93154891	117.5520374	149.0792329
ENSCAFG00000013191	NRP2	23.77604212	25.55479073	54.42575169
ENSCAFG00000015221	EMC1	178.8864121	183.9944933	352.5842175
ENSCAFG00000018210	CDX1	26.04042708	21.46602421	21.29703327
ENSCAFG00000007242	CKAP2L	18.11507971	23.51040747	9.46534812
ENSCAFG00000016526	INTS1	227.5706888	127.7739537	312.356488
ENSCAFG00000005473	ARHGAP15	56.60962409	18.39944933	15.38119069
ENSCAFG00000002222	ASNS	80.38566621	19.42164096	13.01485366
ENSCAFG00000010551	CHEK1	3.396577445	5.110958146	1.183168515
ENSCAFG00000044771		12.4541173	1.022191629	2.36633703
ENSCAFG00000020023	PHYHD1	1449.206377	1271.606387	1723.876526
ENSCAFG00000008713	NUCB2	328.3358197	237.148458	341.9357008
ENSCAFG00000003895	HSD17B14	21162.94187	16608.56959	12454.03179
ENSCAFG00000004666	PVR	2.264384964	3.066574888	2.36633703
ENSCAFG00000010672	HYAL3	32.83358197	18.39944933	26.02970733
ENSCAFG00000010424	DHCR7	831.0292816	434.4314424	916.9555991
ENSCAFG00000024926	DGAT1	232.0994588	262.7032487	447.2376987
ENSCAFG00000004481	SLC6A6	862.7306711	606.1596362	1056.569484
ENSCAFG00000017924	REXO5	6.793154891	4.088766517	3.549505545
ENSCAFG00000010531	C32H4orf17	4.528769927	10.22191629	1.183168515
ENSCAFG00000001811	ZFAND5	3915.121602	481.4522574	380.9802618
ENSCAFG00000006180	SOCS2	257.0076934	315.8572134	364.4159026
ENSCAFG00000013867	LRRC75B	30.48994353	33.73232377	30.76238139
ENSCAFG00000017140	TOB1	4258.175924	1706.037829	805.7377587
ENSCAFG00000010042	PARP3	7.925347372	3.066574888	15.38119069
ENSCAFG00000025465	TRPV6	358.9050167	125.7295704	225.9851864
ENSCAFG00000041018		1.132192482	1.022191629	1.183168515
ENSCAFG00000016589	SRSF5	1073.318473	584.6936119	631.811987
ENSCAFG00000025146	SLC22A11	29.43700453	8.177533034	8.282179605

ENSCAFG00000000206	CDO1	23085.4047	23094.37548	16293.41362
ENSCAFG00000019345	CTNS	189.0761445	190.127643	307.6238139
ENSCAFG00000011620	FANCI	9.057539854	11.24410792	14.19802218
ENSCAFG00000014986	FMO2	26.04042708	17.3772577	10.64851663
ENSCAFG00000011022	ROBO4	29.43700453	31.68794051	53.24258317
ENSCAFG00000018266	SLC6A14	138.1274828	48.04300658	40.22772951
ENSCAFG00000010703	CCDC28B	1.132192482	1.022191629	1.183168515
ENSCAFG00000029218	NCR3LG1	21.51165715	29.64355725	26.02970733
ENSCAFG00000004719	ARMC7	67.93154891	66.4424559	53.24258317
ENSCAFG00000031869		48.68427672	71.55341405	114.767346
ENSCAFG00000017834	TYK2	106.4260933	80.75313871	173.9257717
ENSCAFG00000009303	FBXO21	635.1599823	1756.125219	2039.78252
ENSCAFG00000017486		417.7224162	445.8493229	478.4023573
ENSCAFG00000006538	HSPH1	116.6158256	272.925165	196.4059735
ENSCAFG00000025002	DPP4	756.3045778	370.0333698	1045.920967
ENSCAFG00000009531	OIP5	0	0	0
ENSCAFG00000014062	FANCM	4.528769927	2.044383259	1.183168515
ENSCAFG00000028810	GPIHBP1	39.62673686	6.133149776	8.282179605
ENSCAFG00000044552		18.11507971	49.0651982	15.38119069
ENSCAFG00000007392	CENPK	2.264384964	0	2.36633703
ENSCAFG00000031529	CLEC2D	88.31101358	27.59917399	22.48020178
ENSCAFG00000008598	LRRCC1	336.2611671	550.9612882	379.7970933
ENSCAFG00000023258	ERBB4	0	0	2.36633703
ENSCAFG00000000478	CCHCR1	72.46031883	47.02081495	29.57921287
ENSCAFG00000014975	EIF4G3	313.6173175	223.8599668	214.1535012
ENSCAFG00000006175	CDC25B	16.98288723	7.155341405	5.915842575
ENSCAFG00000030875		138.3199555	70.15301152	46.26188894
ENSCAFG00000004578		1.132192482	1.022191629	0
ENSCAFG00000001147	AKAP7	166.4322948	345.5007707	274.4950955
ENSCAFG00000004031	STIL	0	2.044383259	2.36633703
ENSCAFG00000015916	RAB27A	65.66716394	80.75313871	35.49505545
ENSCAFG00000017604		26.48198215	25.14591408	10.16341754
ENSCAFG00000001753	PPP2R5D	92.83978351	151.2843611	159.7277495
ENSCAFG00000005058	SCN1A	0	0	2.36633703
ENSCAFG00000014065	EFTUD2	283.0481204	340.3898125	312.356488
ENSCAFG00000009765	CASQ2	1.132192482	2.044383259	2.36633703
ENSCAFG00000007780	HTR1F	4.528769927	2.044383259	0

ENSCAFG00000047902		73.59251132	0	2.36633703
ENSCAFG00000031174	PPBP	177.7542196	82.79752197	41.41089802
ENSCAFG00000015737	ANXA11	552.5099311	476.3412992	662.5743684
ENSCAFG00000005177	HYI	1281.607924	987.4371139	1037.390322
ENSCAFG00000042676		3065.977241	251.4591408	113.5841774
ENSCAFG00000031208		40.75892934	28.62136562	67.44060535
ENSCAFG00000014043		15789.98658	13140.71294	14403.2191
ENSCAFG00000005917	FERMT1	47.55208423	84.84190523	140.7970533
ENSCAFG00000017121	SLC51B	5.660962409	1.022191629	15.38119069
ENSCAFG00000009579	TUBGCP5	174.3576422	262.7032487	196.4059735
ENSCAFG00000023863	BAAT	0	48.04300658	40.22772951
ENSCAFG00000030902	TRIM59	7.925347372	4.088766517	1.183168515
ENSCAFG00000018570	SGIP1	7.925347372	64.39807264	42.59406654
ENSCAFG00000017838	CYP46A1	44.15550679	13.28849118	10.64851663
ENSCAFG00000014067	PALD1	12.4541173	5.110958146	5.915842575
ENSCAFG00000000144	CCDC68	19.24727219	33.73232377	11.83168515
ENSCAFG00000035674		1.132192482	1.022191629	0
ENSCAFG00000023774	RAMP1	90.57539854	39.86547354	27.21287584
ENSCAFG00000018997	LEXM	0	0	0
ENSCAFG00000025442	KCNH2	115.4836331	30.15465306	166.8267606
ENSCAFG00000046742		216.248764	129.8183369	16.56435921
ENSCAFG00000016604	ZSWIM4	26.04042708	15.33287444	28.39604436
ENSCAFG00000007184	SKA3	0	7.155341405	2.36633703
ENSCAFG00000017584	SMARCA4	204.9268392	121.6408039	295.7921287
ENSCAFG00000023155	IER2	484.5783822	376.1665196	152.6287384
ENSCAFG00000004093	MGLL	5056.371624	3126.884194	6053.090123
ENSCAFG00000008969	KCTD9	24.9082346	27.59917399	31.9455499
ENSCAFG00000011954		10530.52227	4561.01905	2772.163831
ENSCAFG00000006621	WDR62	12.4541173	5.110958146	10.64851663
ENSCAFG00000019070	CHAF1A	28.30481204	39.86547354	15.38119069
ENSCAFG00000001570	PSAT1	126.805558	11.24410792	4.73267406
ENSCAFG00000019924	GINS2	21.51165715	11.24410792	8.282179605
ENSCAFG00000017505	SPESP1	156.2425625	69.50903079	172.7426032
ENSCAFG00000004762	SLC25A19	31.70138949	25.55479073	29.57921287
ENSCAFG00000010509	UBE2T	14.71850226	12.26629955	11.83168515
ENSCAFG00000012856	TDRKH	2.264384964	11.24410792	13.01485366
ENSCAFG00000005811	FMNL2	366.8303641	328.123513	385.7129359

ENSCAFG00000031783	CASP6	952.1738772	1580.308259	1434.00024
ENSCAFG00000004723	KCNN4	2.264384964	3.066574888	1.183168515
ENSCAFG00000002689	MBOAT7	152.40443	103.2413546	187.555873
ENSCAFG00000018327	C8H14orf180	80.38566621	23.51040747	27.21287584
ENSCAFG00000012038	HHAT	26.04042708	26.57698236	30.76238139
ENSCAFG00000011722	CD34	48.68427672	21.46602421	48.50990911
ENSCAFG00000005898	CKAP2	15.85069474	13.28849118	16.56435921
ENSCAFG00000046961		51.34492905	30.01154624	41.17426432
ENSCAFG00000003280	RSPO1	0	2.044383259	21.29703327
ENSCAFG00000018314	CD209	1904.347754	2597.38893	1420.985386
ENSCAFG00000008761	CX3CL1	2.264384964	0	0
ENSCAFG00000004095	EHD2	73.59251132	32.71013214	34.31188693
ENSCAFG00000015815	ADSS	947.6451073	669.5355172	707.534772
ENSCAFG00000001675	cdkn2A	12.4541173	13.28849118	9.46534812
ENSCAFG00000000115	CS	339.6577445	331.179866	408.1931377
ENSCAFG00000030843	INSIG2	615.9127101	1138.721475	659.0248628
ENSCAFG00000010707	CCDC150	1.132192482	0	0
ENSCAFG00000045451		2.830481204	2.044383259	0
ENSCAFG00000029836	CENPW	7.925347372	5.110958146	3.549505545
ENSCAFG00000041493		0	4.088766517	1.183168515
ENSCAFG00000028656		0	14.69911563	1.680099291
ENSCAFG00000017018	COL1A1	382.6810588	45.99862332	55.6089202
ENSCAFG00000020226	LHX2	151.7137926	36.79889865	48.50990911
ENSCAFG00000017001		344.1865145	100.1747797	227.1683549
ENSCAFG00000012681	GH1	4.528769927	0	0
ENSCAFG00000003818		4.528769927	1.022191629	0
ENSCAFG00000004318	CST7	3.396577445	1.022191629	7.09901109
ENSCAFG00000015949	ALOX15	31.70138949	7.155341405	157.3614125
ENSCAFG00000000395	MAPK9	985.0074591	1186.764482	3719.881811
ENSCAFG00000017593	CENPI	1.132192482	5.110958146	5.915842575
ENSCAFG00000012904	CDK1	7.925347372	17.3772577	4.73267406
ENSCAFG00000012126	HYOU1	549.1133537	404.7878852	535.9753373
ENSCAFG00000011936	TRPM8	1.132192482	4.088766517	0
ENSCAFG00000005479	RRBP1	2545.168699	1116.233259	2713.005405
ENSCAFG00000014773	HSPG2	206.0590317	60.30930613	127.7821996
ENSCAFG00000044694		5.660962409	5.110958146	1.183168515
ENSCAFG00000048561	SVIP	670.2579492	993.5702636	870.812027

ENSCAFG00000012245	EDN3	0	1.022191629	0
ENSCAFG00000002065	KIT	66.79935643	77.68656382	62.70793129
ENSCAFG00000002102	JAK2	1865.85321	1829.723016	1225.762582
ENSCAFG00000019194	SLC43A2	22.64384964	43.95424006	56.79208872
ENSCAFG00000013206	SFI1	24.9082346	27.59917399	44.96040357
ENSCAFG00000008386	STMN2	2.264384964	3.066574888	0
ENSCAFG00000004409	EML2	151.7137926	103.2413546	111.2178404
ENSCAFG00000031015	YWHAH	245.6857685	187.0610682	184.5742883
ENSCAFG00000006573	TAFA4	0	0	2.36633703
ENSCAFG00000015532	MYO9B	378.1522889	258.6144822	547.8070224
ENSCAFG00000001621	TONSL	7.925347372	4.088766517	9.46534812
ENSCAFG00000001501	CDC42EP1	149.4494076	79.73094708	147.8960644
ENSCAFG00000001891	UBAP2	164.1679099	91.99724663	205.8713216
ENSCAFG00000008708	TRIM35	12.4541173	11.24410792	9.46534812
ENSCAFG00000005995	C15H12orf29	174.3576422	351.6339205	229.5346919
ENSCAFG00000007717	CENPH	7.925347372	9.199724663	3.549505545
ENSCAFG00000015000	TACC3	37.3623519	6.133149776	8.282179605
ENSCAFG00000000992	ATAD2	49.8164692	30.66574888	28.39604436
ENSCAFG00000006091	KITLG	302.2953926	248.3925659	202.3218161
ENSCAFG00000007881	CEP55	2.264384964	1.022191629	2.36633703
ENSCAFG00000013894	SOAT1	39.62673686	54.17615635	59.15842575
ENSCAFG00000016657	CDHR2	252.4789234	33.73232377	247.2822196
ENSCAFG00000020035	NUDT7	93.97197599	192.1720263	106.4851663
ENSCAFG00000011370	CDK2AP2	63.40277898	121.6408039	82.82179605
ENSCAFG00000015078	FAM184B	0	0	0
ENSCAFG00000005178	HNMT	966.8923794	2876.447245	2420.762782
ENSCAFG00000004614	APOC4	5608.881555	5622.053961	6282.624815
ENSCAFG00000049055	DIO1	2853.125054	3790.286561	2940.17376
ENSCAFG00000011689	OPRD1	2.264384964	1.022191629	2.36633703
ENSCAFG00000029474	GADD45A	16779.09258	1940.119712	1347.628939
ENSCAFG00000007428	5-Mar	207.1912242	402.7435019	362.0495656
ENSCAFG00000007669	CLCN3	134.7309053	295.4133809	178.6584458
ENSCAFG00000043712	PSPC1	208.3234166	107.3301211	120.6831885
ENSCAFG00000024908	PLA2G4E	0	6.133149776	3.549505545
ENSCAFG00000023072	POC1A	50.22405849	47.02081495	53.24258317
ENSCAFG00000018181		868.3916335	1210.274889	1583.079473
ENSCAFG00000009286	SLC38A1	20.37946467	15.33287444	13.01485366

ENSCAFG00000020080	PTBP2	72.46031883	142.0846365	76.90595347
ENSCAFG00000010949	SLC37A2	21.51165715	17.3772577	13.01485366
ENSCAFG00000019574	ACOT7	6.793154891	9.199724663	20.11386475
ENSCAFG00000018783	RRN3	982.7430742	316.8794051	589.2179205
ENSCAFG00000018719	ALDOC	52.08085416	66.4424559	65.07426832
ENSCAFG00000030145	AUH	1585.069474	2054.605175	1697.846819
ENSCAFG00000024583		63.40277898	48.04300658	44.96040357
ENSCAFG00000041358	COR52A16	3.623015942	0.337323238	0
ENSCAFG00000017538	SPC24	12.4541173	5.110958146	9.46534812
ENSCAFG00000049080		0	3.393676209	2.626634103
ENSCAFG00000048477		15.85069474	0	1.183168515
ENSCAFG00000018746	CAPS	28.30481204	26.57698236	5.915842575
ENSCAFG00000020196	HSPA5	5123.17098	2483.925659	3079.787644
ENSCAFG00000000618	ZDHHC14	3.396577445	1.022191629	3.549505545
ENSCAFG00000000533	PRRC2A	701.9593387	198.3051761	797.4555791
ENSCAFG00000016542	CLCN6	22.64384964	18.39944933	21.29703327
ENSCAFG00000009187	LOXL2	43.02331431	22.48821584	28.39604436
ENSCAFG00000001741	GNMT	8329.291006	10543.88621	11208.11985
ENSCAFG00000014546	HTRA3	2.264384964	0	2.36633703
ENSCAFG00000020397	CDH16	4.528769927	3.066574888	2.36633703
ENSCAFG00000019835	SORT1	233.2316512	311.7684469	397.544621
ENSCAFG00000005078	UBE2O	238.8926137	238.1706496	344.3020379
ENSCAFG00000001909	KIF24	1.132192482	2.044383259	2.36633703
ENSCAFG00000009703	FAM160B2	64.53497146	50.08738983	59.15842575
ENSCAFG00000001319	TIGD5	9.057539854	2.044383259	8.282179605
ENSCAFG00000008921	NEK4	1014.648258	1346.880578	1355.213049
ENSCAFG00000004107	SEC61A1	2879.165481	2069.938049	2716.55491
ENSCAFG00000019614	RPUSD1	65.66716394	66.4424559	68.62377387
ENSCAFG00000032388	DCAKD	7.925347372	17.3772577	17.74752772
ENSCAFG00000005630	P2RY2	9.057539854	8.177533034	16.56435921
ENSCAFG00000016691	ANGPTL7	1.132192482	1.022191629	10.64851663
ENSCAFG00000024608	FAM174A	66.79935643	49.0651982	44.96040357
ENSCAFG00000014000	SAC3D1	26.04042708	27.59917399	37.86139248
ENSCAFG00000025384	PML	328.3358197	182.9723016	339.5693638
ENSCAFG00000018685	CPLANE1	89.44320606	104.2635462	73.35644793
ENSCAFG00000024758	ADAMTS5	2.264384964	9.199724663	5.915842575
ENSCAFG00000028482		16.98288723	15.33287444	23.6633703

ENSCAFG00000019187	MATK	10.18973234	5.110958146	7.09901109
ENSCAFG00000017093	CALR	7626.448557	4434.267288	4414.401729
ENSCAFG00000001474		34.95078191	68.11885017	42.49941306
ENSCAFG00000019703	RHBDF1	163.0357174	93.01943826	156.178244
ENSCAFG00000005493	ZNF219	2.49082346	1.022191629	1.183168515
ENSCAFG00000007571	PIGU	62.2705865	59.2871145	60.34159426
ENSCAFG00000006248	CHL1	83.78224365	74.61998894	52.05941466
ENSCAFG00000002073	ARHGEF39	8.253683192	9.475716403	12.3522793
ENSCAFG00000014620	VAT1	455.1413777	486.5632155	683.8714017
ENSCAFG00000042710		27.17261956	4.088766517	8.282179605
ENSCAFG00000029492	ZNF260	4.528769927	13.28849118	2.36633703
ENSCAFG00000013916	SYVN1	270.5940031	192.1720263	408.1931377
ENSCAFG00000012508	TP53BP1	54.34523913	100.1747797	59.15842575
ENSCAFG00000006757	PPFIBP2	159.6391399	274.9695483	249.6485567
ENSCAFG00000018417	PACS2	233.2316512	173.772577	254.3812307
ENSCAFG00000008618	DSN1	2.264384964	6.133149776	7.09901109
ENSCAFG00000018546	NNT	1330.326166	2266.198842	3173.257957
ENSCAFG00000000782	GTSE1	3.396577445	2.044383259	0
ENSCAFG00000012332	H2AFX	6.793154891	6.133149776	8.282179605
ENSCAFG00000025069	GABRA2	1.132192482	7.155341405	2.36633703
ENSCAFG00000048620		7.925347372	1.022191629	2.36633703
ENSCAFG00000016228	SERPINE2	38.49454438	26.57698236	7.09901109
ENSCAFG00000016863	LMNA	643.0853297	572.4273124	1075.50018
ENSCAFG00000014352	NMD3	627.2346349	1251.162554	841.2328141
ENSCAFG00000014672	STIP1	377.0200964	564.2497794	524.1436521
ENSCAFG00000001420	TMEM184B	54.34523913	31.68794051	101.7524923
ENSCAFG00000015342	RAD51AP1	4.528769927	4.088766517	3.549505545
ENSCAFG00000015885	FKBP10	32.83358197	12.26629955	18.93069624
ENSCAFG00000042998		46.41989175	21.46602421	33.12871842
ENSCAFG00000005825	APBB3	75.85689628	31.68794051	37.86139248
ENSCAFG00000006219	SIGLEC1	14.71850226	12.26629955	48.50990911
ENSCAFG00000012429	VWA5B2	117.7480181	44.97643169	106.4851663
ENSCAFG00000011661	C20H3orf62	2.264384964	2.044383259	2.36633703
ENSCAFG00000044204		22441.18718	14882.08793	18686.96353
ENSCAFG00000011405	TXNIP	3876.627058	6311.011119	3024.178724
ENSCAFG00000012060	ORC2	221.9097264	570.3829291	427.1238339
ENSCAFG00000000562		731.3963432	204.4383259	267.3960844

ENSCAFG00000013022	CEP164	86.04662862	51.10958146	57.97525723
ENSCAFG00000014931	PSMC3IP	1.132192482	1.022191629	0
ENSCAFG00000009858	VTCN1	2.264384964	2.044383259	2.36633703
ENSCAFG00000043534		1.132192482	4.088766517	3.549505545
ENSCAFG00000049822		224.1288237	191.0271717	256.6410826
ENSCAFG00000047066		559.9371138	115.9574184	66.57689234
ENSCAFG00000011969	DALRD3	213.9843791	183.9944933	208.2376586
ENSCAFG00000019676	ERRFI1	8549.18543	6714.776813	6185.604996
ENSCAFG00000011861	FANCB	1.132192482	3.066574888	1.183168515
ENSCAFG00000004851	INTS4	142.6562527	145.1512114	198.7723105
ENSCAFG00000017192	HMMR	30.56919701	34.7545154	14.19802218
ENSCAFG00000007391	CENPJ	9.057539854	16.35506607	11.83168515
ENSCAFG00000010236	UMODL1	2.264384964	3.066574888	0
ENSCAFG00000009654	RBL2	439.2906829	1448.445539	1031.722945
ENSCAFG00000006215	POLE	31.70138949	25.55479073	37.86139248
ENSCAFG00000017453	INTS3	150.5816001	121.6408039	102.9356608
ENSCAFG00000008214	FHDC1	15.85069474	2.044383259	0
ENSCAFG00000004406	FBLN2	57.74181657	34.7545154	28.39604436
ENSCAFG00000004272	RAD54L	1.132192482	1.022191629	1.183168515
ENSCAFG00000020063	SLC27A4	242.2891911	227.9487333	339.5693638
ENSCAFG00000006229	PCDH1	168.6966798	305.6352972	337.2030268
ENSCAFG00000008438	EPHX2	2163.619833	4777.723675	4298.451215
ENSCAFG00000009183	KCNJ3	0	0	1.183168515
ENSCAFG00000003297	LRRN3	22.64384964	0	7.09901109
ENSCAFG00000009140	GALNT13	519.6763491	704.2900326	435.4060135
ENSCAFG00000000217	SGK1	908.0183704	411.9432266	140.7970533
ENSCAFG00000045572		5.660962409	0	1.183168515
ENSCAFG00000009881	ALAS1	42486.65507	3833.21861	5386.966249
ENSCAFG00000007777	CGGBP1	194.7371069	146.173403	105.3019978
ENSCAFG00000018600	LEPR	295.5022377	703.2678409	1044.737799
ENSCAFG00000010737	KPNA6	82.65005117	132.8849118	113.5841774
ENSCAFG00000023585		0	0	3.549505545
ENSCAFG00000005255	VIPR2	35.09796694	12.26629955	17.74752772
ENSCAFG00000023205		752.9080004	560.1610128	537.1585058
ENSCAFG00000005179	CAPN5	271.7261956	305.6352972	435.4060135
ENSCAFG00000020417	CTH	2486.29469	3717.710956	3894.990751
ENSCAFG00000000257	IL20RA	2.264384964	3.066574888	0

ENSCAFG00000009326	BPHL	9237.558459	6371.320425	6015.22873
ENSCAFG00000011195		1.132192482	1.022191629	5.915842575
ENSCAFG00000001013	SREBF2	780.0806199	371.0555614	785.6238939
ENSCAFG00000007232	NOL4L	15.85069474	12.26629955	10.64851663
ENSCAFG00000009777	NAV2	95.10416847	27.59917399	171.5594347
ENSCAFG00000012092	AGAP1	98.50074591	60.30930613	102.9356608
ENSCAFG00000005847	ADAM9	272.8583881	595.9377199	363.2327341
ENSCAFG00000014083	ADAMTS14	4.528769927	2.044383259	2.36633703
ENSCAFG00000031675	COL8A1	4.528769927	8.177533034	3.549505545
ENSCAFG00000003789	KTI12	238.8926137	163.5506607	143.1633903
ENSCAFG00000019611	CHTF18	7.925347372	7.155341405	1.183168515
ENSCAFG00000030630	FCN1	1391.46456	1916.609305	830.5842975
ENSCAFG00000006320	DDX51	167.8248916	139.1713903	223.6188493
ENSCAFG00000012908	THEM4	104.1617083	161.5062774	275.678264
ENSCAFG00000015646	TAF6L	67.93154891	68.48683916	59.15842575
ENSCAFG00000017438	RAB3D	5.660962409	17.3772577	15.38119069
ENSCAFG00000019753	RBP7	0	0	1.183168515
ENSCAFG00000012685	PLXNB1	177.7542196	74.61998894	101.7524923
ENSCAFG00000047451	ZNF382	6.793154891	17.3772577	13.01485366
ENSCAFG00000031355		5.660962409	4.088766517	13.01485366
ENSCAFG00000046990		7.925347372	1.022191629	3.549505545
ENSCAFG00000004844	RALB	88.31101358	89.95286338	125.4158626
ENSCAFG00000020125	STXBP1	53.21304664	44.97643169	65.07426832
ENSCAFG00000001234	UHRF1BP1	65.66716394	112.4410792	124.2326941
ENSCAFG00000003024	SYNCRIP	455.1413777	463.0528081	534.7921688
ENSCAFG00000028766	CLDN18	13.58630978	4.088766517	4.73267406
ENSCAFG00000002659	MSH2	53.21304664	110.396696	43.77723505
ENSCAFG00000010715	TXLNA	54.34523913	95.06382152	107.6683349
ENSCAFG00000008478	MOGS	172.0932572	155.3731276	168.0099291
ENSCAFG00000003623	CASP2	32.53921193	36.33891242	35.74352084
ENSCAFG00000020003	MIGA2	474.3886499	888.2129724	1497.89134
ENSCAFG00000008862	NAA11	0	2.044383259	9.46534812
ENSCAFG00000005386	SERPINH1	459.6701476	270.8807818	191.6732994
ENSCAFG00000019012	FAM151A	0	0	0
ENSCAFG00000019735	PIK3CD	37.3623519	38.84328191	65.07426832
ENSCAFG00000019188	PITPNA	175.4898347	187.0610682	149.0792329
ENSCAFG00000020315	UOX	2273.442503	7491.642451	6747.610041

ENSCAFG00000006069	COL4A2	33.96577445	15.33287444	49.69307763
ENSCAFG00000020039	ZER1	37.3623519	55.19834798	76.90595347
ENSCAFG00000005530	ZFP36	1794.525084	654.2026427	184.5742883
ENSCAFG00000046723		10.00858154	0.63375881	0.922871442
ENSCAFG00000020131	NIBAN2	96.23636095	36.79889865	42.59406654
ENSCAFG00000044360		3.396577445	1.022191629	1.183168515
ENSCAFG00000013843	UPB1	2100.217054	3807.663819	2637.28262
ENSCAFG00000008749	ATP6V0D2	0	0	0
ENSCAFG00000012076	SLC25A20	523.0729266	743.1333145	991.4952155
ENSCAFG00000014367	SLC22A12	0	5.110958146	0
ENSCAFG00000032536	RCHY1	425.7043731	689.9793498	412.9258117
ENSCAFG00000023392	C5H11orf87	3.396577445	3.066574888	8.282179605
ENSCAFG00000019129	TLCD3A	14.71850226	12.26629955	9.46534812
ENSCAFG00000009277	DCLRE1B	73.47929207	59.96176097	119.50002
ENSCAFG00000029826	DUSP1	2656.123562	2939.823126	1438.732914
ENSCAFG00000011859	SPC25	6.793154891	7.155341405	1.183168515
ENSCAFG00000013670	MAPT	12.4541173	5.110958146	2.36633703
ENSCAFG00000003662	TBC1D9	191.3405294	139.0180616	190.4901309
ENSCAFG00000015363	ARHGDIG	11.32192482	6.133149776	0
ENSCAFG00000015226		1897.554599	1713.193171	1798.416143
ENSCAFG00000010532	SPHKAP	9.057539854	0	0
ENSCAFG00000017854	SLC39A6	58.87400905	96.33133914	114.1402666
ENSCAFG00000009592	NELL2	130.2021354	272.925165	171.5594347
ENSCAFG00000024289	ZFP28	11.32192482	38.84328191	31.9455499
ENSCAFG00000009949	CCSER1	61.13839402	96.08601315	59.15842575
ENSCAFG00000015444	DMXL2	9.057539854	4.088766517	7.09901109
ENSCAFG00000003477	PDIA6	2234.947959	1699.904679	1717.960684
ENSCAFG00000016410	PLEKHH1	6.793154891	0	5.915842575
ENSCAFG00000019042	METTL22	519.6763491	449.7643169	532.4258317
ENSCAFG00000018180	ANKRD29	45.28769927	54.17615635	29.57921287
ENSCAFG00000009107	NKX3-1	1.132192482	0	1.183168515
ENSCAFG00000014153	ATP13A1	313.6173175	187.0610682	320.6386676
ENSCAFG00000013828	ADORA2A	13.58630978	6.133149776	7.09901109
ENSCAFG00000016171	TMEM138	32.83358197	13.28849118	16.56435921
ENSCAFG00000002499	DYNC2LI1	12.4541173	8.177533034	9.46534812
ENSCAFG00000029822	VPS26B	100.8783501	92.00746855	195.2583
ENSCAFG00000000143	HSD17B6	126.2168179	244.8251171	200.4287464

ENSCAFG00000002340	IARS	511.7510018	504.9626649	478.00008
ENSCAFG00000045099		129.0699429	5.110958146	7.09901109
ENSCAFG00000004328	ADGRF3	1.132192482	0	0
ENSCAFG00000041974		13.58630978	20.44383259	2.36633703
ENSCAFG00000014529	CENPL	2.264384964	1.022191629	1.183168515
ENSCAFG00000001356	PDGFB	1.132192482	1.022191629	1.183168515
ENSCAFG00000041373		82.66137309	253.8408473	182.3854266
ENSCAFG00000031198	SH2B2	23.77604212	10.22191629	55.6089202
ENSCAFG00000011148	RGS9	2.264384964	0	0
ENSCAFG00000020591	MIR7-1	1.132192482	2.044383259	0
ENSCAFG00000007557	HHIP	409.8536784	518.251156	798.6387476
ENSCAFG00000016534	ACTN1	234.3638437	205.4605175	350.2178804
ENSCAFG00000004396	HDAC11	288.7090829	309.7240637	250.8317252
ENSCAFG00000012105	GALNT2	166.4322948	182.9723016	383.3465989
ENSCAFG00000006795	FKBP1A	519.7329588	381.0117079	701.0746719
ENSCAFG00000010588	MARCKSL1	100.7651309	59.2871145	47.3267406
ENSCAFG00000018129		0	0	3.549505545
ENSCAFG00000030542	FEN1	19.92658768	31.47328027	36.67822396
ENSCAFG00000000442	MDC1	168.6966798	161.5062774	201.1386475
ENSCAFG00000015818	ACLY	2516.863887	1090.678468	1791.317132
ENSCAFG00000011803	CCDC172	9.057539854	0	7.09901109
ENSCAFG00000001091	TGFBI	391.7385987	366.9667949	412.9258117
ENSCAFG00000016265	RELL1	433.6297205	255.5479073	163.2772551
ENSCAFG00000018844	ADAMTS12	2.264384964	2.044383259	9.46534812
ENSCAFG00000043864		0	0	0
ENSCAFG00000012079	MCAM	62.2705865	31.68794051	10.64851663
ENSCAFG00000020364	TPPP3	97.36855343	73.59779731	70.9901109
ENSCAFG00000014746	NAA40	7.925347372	22.48821584	13.01485366
ENSCAFG00000012300	PIGV	596.6654379	463.0528081	313.5396565
ENSCAFG00000018561	TCTEX1D1	0	59.2871145	52.05941466
ENSCAFG00000013245	GPR19	4.528769927	1.022191629	1.183168515
ENSCAFG00000020066	TERF2IP	460.8023401	485.5410239	397.544621
ENSCAFG00000018339	ADRB2	658.9360244	287.2358478	140.7970533
ENSCAFG00000047320		1.132192482	0	1.183168515
ENSCAFG00000001618	CCND3	91.70759102	51.10958146	43.77723505
ENSCAFG00000027581		1.132192482	4.088766517	0
ENSCAFG00000020350	ENKD1	10.18973234	12.2969653	7.09901109

ENSCAFG00000011532	EGF	5.660962409	7.155341405	11.83168515
ENSCAFG00000006595		0	0	0
ENSCAFG00000016648	MTOR	362.3015942	251.4591408	625.8961444
ENSCAFG00000008679	LMBR1L	396.2673686	128.7961453	166.8267606
ENSCAFG00000042778		4.279687581	0	0
ENSCAFG00000018312	CLEC4G	463.066725	750.2886559	347.8515434
ENSCAFG00000005945	RRM1	218.513149	263.7254404	288.6931177
ENSCAFG00000019598	G6PD	185.0342173	82.61352748	201.9313705
ENSCAFG00000005817	LIN7A	112.0870557	74.61998894	128.9653681
ENSCAFG00000003978	PGR	4.528769927	3.066574888	0
ENSCAFG00000028578		6.793154891	0	2.36633703
ENSCAFG00000001300	SLC35B4	20.37946467	28.62136562	33.12871842
ENSCAFG00000013035		279.651543	456.9196583	228.3515234
ENSCAFG00000017111	KLF1	0	0	0
ENSCAFG00000020134	LRSAM1	52.08085416	49.0651982	47.3267406
ENSCAFG00000010936	NPNT	597.7976304	640.9141516	771.4258718
ENSCAFG00000013215	GPRC5D	2011.90604	1568.041959	1987.723105
ENSCAFG00000002702	COL12A1	212.8521866	78.70875545	136.0643792
ENSCAFG00000015017	ATP6V0A1	106.4260933	147.1955946	182.2079513
ENSCAFG00000011326	PRKCA	1226.164458	1086.589702	1521.55471
ENSCAFG00000025301	NXPH1	3.396577445	0	0
ENSCAFG00000013190	BTBD1	275.1227731	571.4051208	433.0396765
ENSCAFG00000014600	BRCA1	13.58630978	23.51040747	33.12871842
ENSCAFG00000023591	VNN1	1834.15182	3029.775989	2040.965688
ENSCAFG00000011057	TRAIP	3.396577445	1.022191629	3.549505545
ENSCAFG00000004718	SLC16A5	330.6002047	95.06382152	150.2624014
ENSCAFG00000017647	CLTC	1239.750768	1550.664702	1934.480522
ENSCAFG00000009003	DDX60	156.2425625	461.0084248	256.7475677
ENSCAFG00000014254	NYX	0	0	0
ENSCAFG00000004331		4.528769927	2.044383259	1.183168515
ENSCAFG00000002641	TTC7A	292.1056603	147.1955946	275.678264
ENSCAFG00000008815		22.64384964	32.71013214	31.9455499
ENSCAFG00000030713	BTBD19	33.96577445	22.48821584	22.48020178
ENSCAFG00000000935	CEP85L	20.37946467	88.93067175	46.14357208
ENSCAFG00000029920	TSC22D3	458.6964621	509.7567436	370.8168443
ENSCAFG00000018487	EMB	202.6624542	90.975055	79.2722905
ENSCAFG00000047592		14.71850226	10.22191629	20.11386475

ENSCAFG00000019141		43.78188327	25.07436067	24.18396445
ENSCAFG00000002592	SCMH1	23.77604212	63.37588101	53.24258317
ENSCAFG00000031104	CX3CR1	1.132192482	3.066574888	4.73267406
ENSCAFG00000004956	PAK1	12.4541173	6.133149776	5.915842575
ENSCAFG00000014703	PIGC	2563.283779	603.0930613	513.4951355
ENSCAFG00000002138	IL18R1	29.43700453	4.088766517	9.46534812
ENSCAFG00000019091	MPND	690.6374139	371.0555614	619.9803018
ENSCAFG00000048640		163.0357174	20.44383259	130.1485366
ENSCAFG00000047522		97.36855343	105.2857378	164.4604236
ENSCAFG00000010019	PCBP4	37.3623519	28.62136562	34.31188693
ENSCAFG00000007026		765.3621177	572.4273124	315.9059935
ENSCAFG00000001667	RBM28	91.70759102	45.99862332	48.50990911
ENSCAFG00000028514	TTPA	3002.574462	6710.688046	3579.084758
ENSCAFG00000002848	BCKDHB	894.4320606	1643.68414	1481.326981
ENSCAFG00000020365	LRRC36	28.30481204	14.31068281	10.64851663
ENSCAFG00000012169	SYTL1	29.50493608	6.133149776	15.02624014
ENSCAFG00000009088	NUP93	64.53497146	89.95286338	70.9901109
ENSCAFG00000002525	COL15A1	97.36855343	7.155341405	3.549505545
ENSCAFG00000015798		82.32171535	37.50421088	38.78426392
ENSCAFG00000006421	GAS6	132.4665204	40.88766517	23.6633703
ENSCAFG00000017916	SEMA7A	152.9818481	64.54117947	107.7866517
ENSCAFG00000017425	CUEDC1	10.18973234	2.044383259	14.19802218
ENSCAFG00000044541		39.08328447	1.788835351	6.507426832
ENSCAFG00000000189	SLC18B1	115.4836331	157.4175109	144.3465588
ENSCAFG00000007055	CHCHD7	78.12128124	51.10958146	67.44060535
ENSCAFG00000016189	ZNF488	164.1679099	90.975055	105.3019978
ENSCAFG00000014990		754.0401929	2027.006001	1195.532626
ENSCAFG00000000253	PEX7	456.2735702	614.3371692	611.6981222
ENSCAFG00000006560	NCAPH	15.85069474	14.31068281	10.64851663
ENSCAFG00000008509	CCDC113	14.71850226	12.26629955	28.39604436
ENSCAFG00000001875	CKAP4	207.1912242	68.48683916	164.4604236
ENSCAFG00000030926	GIPC2	61.13839402	229.9931166	196.4059735
ENSCAFG00000003653	PREP	310.22074	304.6131055	505.2129559
ENSCAFG00000009759	SNX21	14.71850226	13.28849118	13.01485366
ENSCAFG00000006971	EPHB1	0	0	1.183168515
ENSCAFG00000012071		323.773084	471.9969848	481.7507242
ENSCAFG00000007486	CEP89	16.98288723	17.3772577	10.64851663

ENSCAFG00000016877	UBQLN4	64.53497146	45.99862332	80.45545902
ENSCAFG00000001983	SLC35B2	279.651543	166.6172356	333.6535212
ENSCAFG00000014319	UNC5B	47.55208423	12.26629955	70.9901109
ENSCAFG00000017807	CRYM	869.523826	932.2387659	641.2773351
ENSCAFG00000003538	SIM1	3.396577445	0	0
ENSCAFG00000010964	HTR2B	5.660962409	6.133149776	0
ENSCAFG00000012065	PCK1	67268.08411	19091.47306	9174.288665
ENSCAFG00000028626	SLPI	20.37946467	2.044383259	3.549505545
ENSCAFG00000020392	RABGGTB	1063.12874	1266.301212	1248.100803
ENSCAFG00000009047	TENT2	946.5129148	846.374669	744.2129959
ENSCAFG00000014444		13937.18755	17769.72817	18141.68848
ENSCAFG00000018970	GRIN2A	3.396577445	3.066574888	8.282179605
ENSCAFG00000007760	CCDC125	116.6158256	282.1248897	192.8564679
ENSCAFG00000029814	ZNF175	6.793154891	17.3772577	8.282179605
ENSCAFG00000013291	TCEA3	3863.040748	3026.709414	2968.569804
ENSCAFG00000002288	GCLC	922.7368727	1087.611894	1151.222965
ENSCAFG00000005220	SZT2	200.432035	68.48683916	216.7683036
ENSCAFG00000008246	CRISPLD1	6.793154891	10.22191629	15.38119069
ENSCAFG00000013929	TOR3A	74.7247038	55.19834798	76.90595347
ENSCAFG00000019130	RFLNB	3.396577445	1.022191629	0
ENSCAFG00000011551	CYP2W1	1.132192482	0	0
ENSCAFG00000002271	PHF14	110.9548632	125.7295704	93.47031268
ENSCAFG00000007638	CTR9	370.2269415	647.0473013	524.1436521
ENSCAFG00000008235	CPA3	224.1741114	111.4188876	101.7524923
ENSCAFG00000005919	FAM98C	207.8705397	220.3538495	210.6039957
ENSCAFG00000048926		1.132192482	1.022191629	0
ENSCAFG00000010693	FGF19	0	0	0
ENSCAFG00000041427		5.660962409	6.133149776	1.183168515
ENSCAFG00000028621	OLFML2A	64.53497146	20.44383259	9.46534812
ENSCAFG00000006598	MCM4	64.53497146	123.6851871	79.2722905
ENSCAFG00000005405	SEC23B	332.8645896	361.8558368	358.50006
ENSCAFG00000014027	LANCL1	161.9035249	247.3703743	288.6931177
ENSCAFG00000001910	POLH	26.04042708	50.08738983	35.49505545
ENSCAFG00000008901	BPI	233.2316512	240.2150329	429.4901709
ENSCAFG00000009262	GOLGA7B	0	0	0
ENSCAFG00000016385	ADGRE5	263.7102729	162.5284691	224.8020178
ENSCAFG00000009209	NR4A2	16.98288723	45.99862332	4.73267406

ENSCAFG00000004673	KHK	10715.06965	6843.572958	5940.689114
ENSCAFG00000015668	TMEM128	382.6810588	520.2955393	501.6634503
ENSCAFG00000015754		21.51165715	16.35506607	9.46534812
ENSCAFG00000043556		0	4.088766517	2.36633703
ENSCAFG00000004787	CASKIN2	83.06896239	80.21137715	226.4821171
ENSCAFG00000013238	PTPN23	105.6335586	43.65780449	103.2787797
ENSCAFG00000028613		1977.940266	1893.098897	1872.955759
ENSCAFG00000019515	DPP7	107.5582858	157.4175109	169.1930976
ENSCAFG00000000423	RMND1	324.9392423	461.0084248	346.6683749
ENSCAFG00000011840	SYT12	3.396577445	4.088766517	20.11386475
ENSCAFG00000004609	PCF11	619.3092875	334.2566628	283.9604436
ENSCAFG00000007291	PARPBP	0	1.022191629	0
ENSCAFG00000002917	AEBP1	224.1741114	25.55479073	88.73763862
ENSCAFG00000031118		0	0	1.183168515
ENSCAFG00000023111		119.2538341	92.4776767	61.00416863
ENSCAFG00000043200		8.559375162	0	2.36633703
ENSCAFG00000014571	CDC45	4.528769927	0	1.183168515
ENSCAFG00000008577	MCPH1	96.23636095	56.22053961	56.79208872
ENSCAFG00000001833	ABCB4	2014.170425	2219.178027	4478.292829
ENSCAFG00000015590	CPAMD8	21.51165715	13.28849118	11.83168515
ENSCAFG00000000459	TBC1D15	595.5332454	1052.857378	718.1832886
ENSCAFG00000029601	C11H9orf152	0	2.044383259	0
ENSCAFG00000011162	VSIG2	1.132192482	0	3.549505545
ENSCAFG00000049269		1.132192482	3.066574888	0
ENSCAFG00000006015		0	0	1.313317052
ENSCAFG00000016888	KCNAB3	0	1.022191629	3.549505545
ENSCAFG00000035722		2.705940031	0	1.739257717
ENSCAFG00000028906		0	0	4.850990911
ENSCAFG00000007436	FBXL17	125.3337077	153.2776348	205.6228562
ENSCAFG00000000970	SERHL2	221.9097264	142.0846365	178.6584458
ENSCAFG00000003110	TMEM17	66.79935643	142.0846365	78.08912199
ENSCAFG00000000437	PPP1R18	45.28769927	28.62136562	35.49505545
ENSCAFG00000000824	P4HA2	193.6049144	137.99587	132.5148737
ENSCAFG00000010250	KIF21A	202.6624542	631.7144269	508.7624614
ENSCAFG00000032133	ZSCAN31	21.51165715	17.3772577	11.83168515
ENSCAFG00000005667	NEK10	0	2.044383259	0
ENSCAFG00000010174	BBOX1	3876.627058	1817.456717	1299.119029

ENSCAFG00000015268	TBX19	918.2081027	589.8045701	480.3664171
ENSCAFG00000008572		1560.16124	3309.856496	4453.44629
ENSCAFG00000019400	DPH7	80.38566621	53.15396472	89.92080714
ENSCAFG00000020088	LCN2	7.925347372	4.088766517	8.282179605
ENSCAFG00000013018	RTKN2	0	0	0
ENSCAFG00000020120	TOR2A	167.5644873	157.407289	188.1237939
ENSCAFG00000007614	CCNE1	6.793154891	14.31068281	17.74752772
ENSCAFG00000019848	COL5A1	120.0124031	25.55479073	79.2722905
ENSCAFG00000001159	ZFAT	28.30481204	25.55479073	34.31188693
ENSCAFG00000018398	CDCA4	20.37946467	31.68794051	34.31188693
ENSCAFG00000016465	MAGED2	339.6577445	186.0388765	212.9703327
ENSCAFG00000020208	GOLGA1	64.53497146	101.1969713	84.00496456
ENSCAFG00000015337	PLVAP	168.6966798	209.549284	300.5248028
ENSCAFG00000031897	RITA1	29.43700453	24.5325991	26.02970733
ENSCAFG00000014700	MYNN	123.4089805	280.0805064	126.5990311
ENSCAFG00000005002	TIMP4	1.132192482	0	0
ENSCAFG00000030441	MGAT3	0	0	0
ENSCAFG00000006596	ANKS1B	39.62673686	73.59779731	67.44060535
ENSCAFG00000008640	TUBA1A	4077.828984	1371.147408	1125.796674
ENSCAFG00000006073	POSTN	2187.395875	1158.143116	1236.411098
ENSCAFG00000031495	ZNF461	4.528769927	7.155341405	5.915842575
ENSCAFG00000000974	DERL1	418.9112183	502.9182816	557.2723706
ENSCAFG00000006370	HGF	287.5768904	243.2816078	152.6287384
ENSCAFG00000013756	CLEC14A	234.3638437	302.5687223	210.6039957
ENSCAFG00000005253	WDR43	1088.036975	531.5396472	455.5198783
ENSCAFG00000012494	TACC2	58.87400905	21.46602421	16.56435921
ENSCAFG00000004539	ZNF862	44.15550679	51.10958146	34.31188693
ENSCAFG00000016107	NOTCH3	39.62673686	5.110958146	7.09901109
ENSCAFG00000016905	TLN2	36.23015942	22.48821584	67.44060535
ENSCAFG00000010218	KIF18A	7.925347372	16.35506607	9.46534812
ENSCAFG00000019506	CIRBP	863.8628636	353.6783037	570.2872242
ENSCAFG00000011365	CRB1	0	0	1.183168515
ENSCAFG00000012298	FES	88.31101358	94.04162989	80.45545902
ENSCAFG00000017612	ASB2	2.264384964	2.044383259	3.549505545
ENSCAFG00000018316	EVI5L	21.51165715	11.24410792	20.11386475
ENSCAFG00000045946		0	0	0
ENSCAFG00000013612	KLRB1	14.71850226	7.155341405	9.46534812

ENSCAFG00000028878		12.4541173	3.066574888	1.183168515
ENSCAFG00000009337	TUBB2B	45.36695274	29.19379293	41.17426432
ENSCAFG00000008990	OTULIN	427.9687581	409.8988433	448.4208672
ENSCAFG00000016356		0	0	0
ENSCAFG00000005423	STT3B	446.0838378	879.0848012	779.7080514
ENSCAFG00000001867	KLF9	141.5240602	284.1692729	239.00004
ENSCAFG00000029192		7.155456485	0.337323238	0
ENSCAFG00000030055	FCER2	36.23015942	23.51040747	37.86139248
ENSCAFG00000010838	TRIP13	19.24727219	22.48821584	26.02970733
ENSCAFG00000004756	MIF4GD	607.9873627	842.2859025	741.8466589
ENSCAFG00000002134	IL18RAP	53.21304664	7.155341405	7.09901109
ENSCAFG00000020022	SASS6	4.528769927	27.45606716	23.16643952
ENSCAFG00000028827	UBE2V2	738.1894981	890.3289091	637.7278296
ENSCAFG00000030141	LONRF1	219.6453415	185.0166849	104.1188293
ENSCAFG00000018430		313.6173175	133.9071034	132.5148737
ENSCAFG00000008698	ACOT12	2263.252771	3182.082542	3909.188773
ENSCAFG00000046721		6.793154891	1.022191629	0
ENSCAFG00000001853	ABCC10	38.49454438	39.86547354	44.96040357
ENSCAFG00000046619	VPS37B	11.32192482	9.199724663	13.01485366
ENSCAFG00000007162	SLC35A4	112.0870557	52.13177309	125.4158626
ENSCAFG00000009135	SAA1	247.1123311	203.5899068	135.2598246
ENSCAFG00000006030	PCNA	192.4727219	122.6629955	169.1930976
ENSCAFG00000015022	FAM53A	31.70138949	15.33287444	33.12871842
ENSCAFG00000011210	UNC93B1	378.1522889	210.5714756	378.6139248
ENSCAFG00000042194		464.1989175	161.5062774	111.2178404
ENSCAFG00000008972	PAK6	0	0	0
ENSCAFG00000002753	GPNMB	512.8831942	233.0596915	81.63862753
ENSCAFG00000003896	HSPA4L	12.4541173	14.31068281	5.915842575
ENSCAFG00000011725	ATRNL1	131.3343279	119.5964206	115.9505145
ENSCAFG00000020033	CLEC3A	0	4.088766517	0
ENSCAFG00000014215	C5H11orf53	2.264384964	2.044383259	0
ENSCAFG00000019894	BANP	49.8164692	47.02081495	30.76238139
ENSCAFG00000014481		2.264384964	3.066574888	0
ENSCAFG00000046219		4.528769927	6.133149776	9.46534812
ENSCAFG00000000064	CD63	2422.891911	1586.441409	1903.718141
ENSCAFG00000011197	MMP14	356.6406318	298.4799557	583.3020779
ENSCAFG00000044008		199.2658768	124.7073788	73.35644793

ENSCAFG00000001117	TMEM200A	9.057539854	1.022191629	3.549505545
ENSCAFG00000003749	SLC7A11	0	0	0
ENSCAFG00000018979	ZIC3	4.528769927	1.022191629	4.73267406
ENSCAFG00000024647	SERPINB5	28.30481204	41.9098568	34.31188693
ENSCAFG00000043675		0	0	0
ENSCAFG00000019984	ASB6	100.7651309	100.1747797	127.7821996
ENSCAFG00000004419	OPA3	0	0	0
ENSCAFG00000012349	PRUNE1	43.02331431	107.3301211	85.18813308
ENSCAFG00000000714	ZBED4	45.28769927	22.48821584	55.6089202
ENSCAFG00000028782	PHF13	5.660962409	7.155341405	21.29703327
ENSCAFG00000005097	RHBDF2	39.62673686	15.33287444	35.49505545
ENSCAFG00000019905	POMT1	234.3638437	134.9292951	197.589142
ENSCAFG00000019167	DAPK3	149.4494076	74.61998894	80.45545902
ENSCAFG00000006371	NRG1	532.1304664	145.1512114	53.24258317
ENSCAFG00000013079	OLFML2B	64.53497146	30.66574888	23.6633703
ENSCAFG00000001442	MICALL1	28.30481204	35.77670702	49.69307763
ENSCAFG00000002454	XPA	233.2316512	325.0569381	300.5248028
ENSCAFG00000018136	SYT17	1.132192482	1.022191629	2.36633703
ENSCAFG00000004479	ANO10	160.7713324	123.6851871	239.00004
ENSCAFG00000014683	CFAP70	1.132192482	1.022191629	2.36633703
ENSCAFG00000016249	PGM2	978.2143043	1201.075164	548.9901909
ENSCAFG00000011781	GPER1	0	0	0
ENSCAFG00000009797	STAT4	43.02331431	36.79889865	41.41089802
ENSCAFG00000013260	KLHL34	5.660962409	1.022191629	1.183168515
ENSCAFG00000004291		5.660962409	4.088766517	2.36633703
ENSCAFG00000003904	FGF21	5487.736959	220.7933919	104.1188293
ENSCAFG00000043976		0	0	0
ENSCAFG00000023831	GPRIN1	0	0	0
ENSCAFG00000019406	SPPL2B	67.93154891	49.0651982	111.2178404
ENSCAFG00000001862	ZNF804B	18.11507971	16.35506607	7.09901109
ENSCAFG00000020167	HYDIN	20.37946467	9.199724663	13.01485366
ENSCAFG00000049393		1.132192482	1.451512114	0
ENSCAFG00000004140	SLC8A2	1.132192482	0	0
ENSCAFG00000015996	CCNB3	3.396577445	1.022191629	0
ENSCAFG00000004722	JPT1	36.23015942	28.62136562	31.9455499
ENSCAFG00000014615	CXCR2	33.46760976	12.26629955	6.022327741
ENSCAFG00000008583	ASIC5	0	4.088766517	0

ENSCAFG00000017928	TVP23B	373.623519	606.1596362	469.7179004
ENSCAFG00000012339	ATF3	1119.738364	800.3760457	15.38119069
ENSCAFG00000016383	STARD3	190.2083369	145.1512114	240.1832085
ENSCAFG00000000811	SFT2D1	448.3482228	466.1193829	457.8862153
ENSCAFG00000041860		5886.325322	3201.320188	7131.749363
ENSCAFG00000023018	ASGR2	1431.091297	837.1749444	1198.549706
ENSCAFG00000031156	TMEM171	6.793154891	3.066574888	0
ENSCAFG00000004473	ESD	1677.909258	2043.361067	1772.386435
ENSCAFG00000004400	NINL	49.8164692	26.57698236	37.86139248
ENSCAFG00000017973	MPI	125.6733655	140.0402532	162.0940866
ENSCAFG00000013085	HMGCL	3128.247827	3301.678963	3280.926292
ENSCAFG00000025183	EPS8L2	366.8303641	260.6588655	495.7476078
ENSCAFG00000028848	JUNB	992.9328065	775.8434466	171.5594347
ENSCAFG00000018897	JUN	5191.102529	2127.180781	382.1634303
ENSCAFG00000014712	MACROD1	1773.013426	1040.591079	1381.940825
ENSCAFG00000024433		19.24727219	21.46602421	34.31188693
ENSCAFG00000047583		0	0.684868392	5.963169315
ENSCAFG00000003136	CCM2	276.2549656	135.9514867	194.0396365
ENSCAFG00000019474	ARHGEF16	109.8226707	67.46464753	89.92080714
ENSCAFG00000011468	HOMEZ	56.60962409	94.04162989	127.7821996
ENSCAFG00000029384		3274.176116	1159.022201	479.7038427
ENSCAFG00000010971	SATB2	0	1.022191629	0
ENSCAFG00000050036		280.7837355	56.22053961	100.5693238
ENSCAFG00000019081	MYO10	192.4727219	114.4854625	300.5248028
ENSCAFG00000014129	CXHXorf38	104.1617083	152.3065528	120.6831885
ENSCAFG00000014785	CEP152	15.85069474	19.42164096	8.282179605
ENSCAFG00000001541	HGH1	124.541173	53.15396472	152.6287384
ENSCAFG00000046664	TRBV18	4.528769927	2.044383259	3.549505545
ENSCAFG00000006001	PROSER1	88.31101358	78.70875545	92.28714417
ENSCAFG00000010132	APLP2	576.2859732	583.6714203	682.6882331
ENSCAFG00000048079		11.32192482	11.24410792	16.56435921
ENSCAFG00000032312	ZNF470	12.4541173	11.24410792	5.915842575
ENSCAFG00000009383	VPS18	141.5240602	105.2857378	127.7821996
ENSCAFG00000013814	FAM163A	1.132192482	0	0
ENSCAFG00000007408	THNSL2	440.4228754	453.8530834	428.3070024
ENSCAFG00000001209	CDC23	44.15550679	95.06382152	81.63862753
ENSCAFG00000046633	CRB2	1.132192482	1.022191629	11.83168515

ENSCAFG00000006006	FASN	5625.864442	626.6034687	2681.059855
ENSCAFG00000047081		132.4665204	89.95286338	189.3069624
ENSCAFG00000029326	FAM133B	3.136173175	5.438059468	7.09901109
ENSCAFG00000011935	EYA3	37.3623519	57.24273124	68.62377387
ENSCAFG00000008557	APP	1930.388181	1515.910186	1416.252712
ENSCAFG00000023928	CCL8	0	2.044383259	0
ENSCAFG00000009978	ETS2	3190.518414	610.2484027	298.1584658
ENSCAFG00000043032		2.264384964	3.066574888	1.183168515
ENSCAFG00000004253	UGGT1	354.3762468	422.1651429	509.94563
ENSCAFG00000009756	ELOVL2	0	0	11.83168515
ENSCAFG00000013274	DUSP16	600.0620153	284.1692729	278.044601
ENSCAFG00000007731	ATP1B3	76.98908876	40.88766517	50.87624614
ENSCAFG00000044020		4.528769927	0	0
ENSCAFG00000023615	MMP12	0	2.044383259	0
ENSCAFG00000010003	MOB2	767.6265026	249.4147575	198.7723105
ENSCAFG00000000399		3.396577445	3.066574888	7.09901109
ENSCAFG00000016457	FCRL2	9.057539854	9.199724663	8.282179605
ENSCAFG00000003560	CDK5RAP2	121.1445956	56.22053961	87.55447011
ENSCAFG00000025345	RELN	1374.481673	720.6450986	1620.940866
ENSCAFG00000015072	HDC	11.32192482	7.155341405	8.282179605
ENSCAFG00000004970	DPP10	87.1788211	80.75313871	41.41089802
ENSCAFG00000013860	PLOD3	451.7448002	227.9487333	414.1089802
ENSCAFG00000032377	EVA1A	948.7772997	427.276101	317.089162
ENSCAFG00000012638	DPP3	191.3405294	304.6131055	256.7475677
ENSCAFG00000024864		130.2021354	49.0651982	74.53961644
ENSCAFG00000011479	POLQ	6.793154891	7.155341405	3.549505545
ENSCAFG00000010789		0	1.063079294	1.242326941
ENSCAFG00000011826	CERS6	116.6158256	104.2635462	215.3366697
ENSCAFG00000011477	BSN	0	0	0
ENSCAFG00000019427	MOB3A	45.28769927	25.55479073	30.76238139
ENSCAFG00000004797	USP35	2.264384964	1.022191629	1.183168515
ENSCAFG00000009371	SORBS3	125.6733655	84.84190523	239.00004
ENSCAFG00000003804		2.264384964	4.088766517	2.36633703
ENSCAFG00000002731	RAPGEF5	43.02331431	37.82109028	37.86139248
ENSCAFG00000003239	ETAA1	277.387158	312.7906386	163.2772551
ENSCAFG00000044116		4.528769927	0	1.183168515
ENSCAFG00000012985	FOXK2	204.9268392	133.9071034	223.6188493

ENSCAFG00000013733	GATM	112.0870557	15.33287444	2.36633703
ENSCAFG00000030438		0	2.044383259	0
ENSCAFG00000015634	SLC2A9	587.607898	912.8171249	1002.143732
ENSCAFG00000009033	DYSF	253.6111159	179.9057268	309.9901509
ENSCAFG00000014236	FDX1	929.5300275	625.5812771	806.9209272
ENSCAFG00000001220	CDC25C	0	4.088766517	0
ENSCAFG00000049180		0	0	0
ENSCAFG00000001870	STEAP2	13.58630978	78.70875545	36.67822396
ENSCAFG00000042299		0	0	2.36633703
ENSCAFG00000009869	EHD4	61.13839402	68.48683916	65.07426832
ENSCAFG00000018246	AP2B1	502.6934619	785.0431713	765.5100292
ENSCAFG00000005618	ACP7	2.264384964	2.044383259	0
ENSCAFG00000024571	GABRA4	2.264384964	2.044383259	0
ENSCAFG00000001651	IMPDH1	116.6158256	54.17615635	121.866357
ENSCAFG00000018074	TTC19	266.0652332	389.4550108	488.6485967
ENSCAFG00000011124		17.57162732	14.58667455	3.289208472
ENSCAFG00000024882		6.793154891	0	0
ENSCAFG00000017248	UBAP2L	418.9112183	340.3898125	556.089202
ENSCAFG00000003893	AGK	95.10416847	104.2635462	137.2475477
ENSCAFG00000009104	STC1	0	5.110958146	1.183168515
ENSCAFG00000019714	SLC2A5	1.132192482	1.022191629	0
ENSCAFG00000016571	AGTRAP	41.89112183	21.46602421	59.15842575
ENSCAFG00000032120	C1QTNF6	5.660962409	6.133149776	5.915842575
ENSCAFG00000016984	PER1	1260.130232	403.7656936	648.3763462
ENSCAFG00000004380	PYGB	394.0029837	272.3322939	404.6436321
ENSCAFG00000010232	TRIM8	289.8412753	238.1706496	425.9406654
ENSCAFG00000004256	PRKD2	71.32812635	35.77670702	73.35644793
ENSCAFG00000012294	IGFBP3	560.4352785	158.4397025	334.8366897
ENSCAFG00000014737	CALCRL	193.6049144	345.5007707	216.5198382
ENSCAFG00000018924	TP53I13	775.55185	615.3593608	668.490211
ENSCAFG00000004652	GLB1	169.8288723	157.4175109	228.3515234
ENSCAFG00000016295	GIPC1	31.70138949	14.31068281	31.9455499
ENSCAFG00000006238		32.83358197	56.22053961	37.86139248
ENSCAFG00000018722	NDE1	30.08235424	46.13150823	23.6633703
ENSCAFG00000008937	KANSL2	144.9206377	158.4397025	127.7821996
ENSCAFG00000000709	CRELD2	146.0528301	71.55341405	81.63862753
ENSCAFG00000010775	MEF2A	244.5535761	463.0528081	468.5347319

ENSCAFG00000020400	SLC44A5	35.09796694	1.022191629	1.183168515
ENSCAFG00000008689	PRKCD	46.41989175	29.64355725	41.41089802
ENSCAFG00000017060	NACC1	44.15550679	41.9098568	72.17327941
ENSCAFG00000032716	EMCN	41.89112183	52.13177309	47.3267406
ENSCAFG00000018991	DPP9	277.387158	249.4147575	459.0693838
ENSCAFG00000031673		31.70138949	24.5325991	26.02970733
ENSCAFG00000010113	GAS2	652.1428695	625.5812771	362.0495656
ENSCAFG00000010757	CPT1A	7698.908876	2881.558203	4342.22845
ENSCAFG00000006055	ATP2C1	374.1896152	602.8272914	492.1981022
ENSCAFG00000003480	STK17A	47.55208423	45.99862332	37.86139248
ENSCAFG00000016759	TARDBP	125.6733655	161.5062774	143.1633903
ENSCAFG00000007264	CSTF3	141.5240602	132.8849118	120.6831885
ENSCAFG00000046399		733.6607282	395.5881605	343.1188693
ENSCAFG00000007141	CA8	289.8412753	307.6796804	287.5099491
ENSCAFG00000044096		2.264384964	0	2.36633703
ENSCAFG00000004912	PRKAG2	216.248764	239.1928412	435.4060135
ENSCAFG00000032668	SPOCK1	0	0	0
ENSCAFG00000014280	ASB16	0	2.044383259	0
ENSCAFG00000008668	SEPTIN11	62.2705865	99.30591678	111.2178404
ENSCAFG00000000223	GLI1	15.85069474	15.33287444	33.12871842
ENSCAFG00000014633	FRMD6	20.37946467	63.37588101	42.59406654
ENSCAFG00000003137	UGP2	3587.917975	6125.994434	3919.83729
ENSCAFG00000007048	HAMP	3793.977006	1846.078082	1516.822036
ENSCAFG00000023575	MINDY3	182.2829896	312.7906386	189.3069624
ENSCAFG00000024645	BNIP3	1274.848734	1662.083589	1913.183489
ENSCAFG00000019394	ARRDC1	49.8164692	34.7545154	37.86139248
ENSCAFG00000005537		2782.92912	820.8198783	3064.406454
ENSCAFG00000018622	DAB2	44.15550679	188.0832598	146.7128959
ENSCAFG00000016051	CACNA1C	5.660962409	9.199724663	4.73267406
ENSCAFG00000002784	FAM221A	18.11507971	45.99862332	24.84653881
ENSCAFG00000005076	ATP2B2	100.7651309	88.93067175	143.1633903
ENSCAFG00000030416	RGS16	1345.044668	2801.827256	2985.134163
ENSCAFG00000019370	HASPIN	3.396577445	2.044383259	2.36633703
ENSCAFG00000018678	SARM1	30.56919701	17.3772577	39.04456099
ENSCAFG00000001106	LAMA2	18.11507971	4.088766517	2.36633703
ENSCAFG00000005938	GPS1	358.9050167	269.8585901	382.1634303
ENSCAFG00000012385	BLM	3.396577445	8.177533034	1.183168515

ENSCAFG00000000221	ALDH8A1	2001.716308	2714.940967	2365.153861
ENSCAFG00000003376	CSF3R	264.9330407	47.02081495	65.07426832
ENSCAFG00000004830		136.9952903	226.9265417	239.00004
ENSCAFG00000007038	HM13	827.6327042	517.2289644	689.7872442
ENSCAFG00000016693	RNF40	233.2316512	209.549284	236.633703
ENSCAFG00000031939		122.276788	149.2399779	130.1485366
ENSCAFG00000047415		0	2.575922906	0
ENSCAFG00000005051	SCN5A	0	2.044383259	3.549505545
ENSCAFG00000010142	SLC39A10	62.2705865	43.95424006	48.50990911
ENSCAFG00000031331	OCIAD2	16.98288723	7.155341405	3.549505545
ENSCAFG00000023887		1.132192482	2.044383259	1.183168515
ENSCAFG00000001699	OSTF1	671.3901417	1118.277642	986.7625415
ENSCAFG00000002404	CNNM4	54.34523913	87.90848012	149.0792329
ENSCAFG00000008878	MIS18A	2.264384964	2.044383259	1.183168515
ENSCAFG00000015868	ASRGL1	52.08085416	31.68794051	39.04456099
ENSCAFG00000016452	PI4K2B	262.6686558	559.1388212	495.7476078
ENSCAFG00000011521	AEN	140.3918677	44.97643169	60.34159426
ENSCAFG00000014751		214.3806464	107.5652251	154.2851744
ENSCAFG00000001364	SRPK1	65.66716394	130.8405285	141.9802218
ENSCAFG00000015230	TRPM7	296.6344302	842.2859025	746.5793329
ENSCAFG00000001172	SGSM3	103.0295158	75.64218057	61.52476278
ENSCAFG00000041033		1.132192482	2.044383259	1.183168515
ENSCAFG00000016119	H3F3A	2677.635219	2504.369492	2197.143932
ENSCAFG00000009147	ARHGAP1	114.3514407	144.1290197	209.4208272
ENSCAFG00000031108	COLEC10	703.0915312	518.251156	459.0693838
ENSCAFG00000017106	GCDH	1420.901565	2698.585901	2709.455899
ENSCAFG00000005390	DYNC1LI1	109.8226707	150.2621695	94.6534812
ENSCAFG00000010465	AGFG1	382.6810588	382.2996693	328.9208472
ENSCAFG00000007394	ABHD6	48.68427672	36.79889865	86.37130159
ENSCAFG00000002142	IL1RL1	27.17261956	13.28849118	8.282179605
ENSCAFG00000018737	APCDD1	19.24727219	2.044383259	0
ENSCAFG00000002345	BMP5	43.02331431	18.39944933	10.64851663
ENSCAFG00000004398	QPCTL	100.7651309	70.53122242	126.5990311
ENSCAFG00000008799	TMA16	245.6857685	387.4106275	237.8168715
ENSCAFG00000003348	MBOAT2	0	0	0
ENSCAFG00000019014	SEMA6B	6.793154891	13.28849118	14.19802218
ENSCAFG00000013614	DTX2	114.3514407	88.93067175	106.4851663

ENSCAFG00000016086	MMRN2	113.2192482	140.0402532	99.38615526
ENSCAFG00000048581		6.793154891	0	0
ENSCAFG00000009421	MMP2	104.1617083	25.55479073	21.29703327
ENSCAFG00000003782	BTF3L4	653.275062	606.1596362	552.5396965
ENSCAFG00000041519		0	0	0
ENSCAFG00000003329	C10H2orf42	184.5473745	181.95011	215.3366697
ENSCAFG00000009464	7-Mar	391.7385987	491.6741737	326.5545101
ENSCAFG00000015233	PROM1	6.793154891	3.066574888	2.36633703
ENSCAFG00000009660	MORC3	262.6686558	455.8974667	295.7921287
ENSCAFG00000046486		1536.385198	1448.445539	944.1684749
ENSCAFG00000000460	LAPTM4B	489.1071521	179.9057268	302.8911398
ENSCAFG00000003383	ADAM17	78.12128124	90.975055	89.92080714
ENSCAFG00000043512	COQ10B	452.8769927	231.0153082	162.0940866
ENSCAFG00000010201	MFSD13A	648.7462921	496.7851318	724.0991312
ENSCAFG00000049457		1.947371069	6.296700436	2.129703327
ENSCAFG00000009920	GMPR	247.9501535	93.01943826	112.4010089
ENSCAFG00000001590	SAXO1	708.7524936	34.7545154	36.67822396
ENSCAFG00000017054	ERG28	212.8521866	181.95011	267.3960844
ENSCAFG00000006130	PXMP2	124.541173	144.1290197	106.4851663
ENSCAFG00000014088	TBATA	1.132192482	1.022191629	2.36633703
ENSCAFG00000017945	CSK	300.0310077	195.2386012	422.3911598
ENSCAFG00000007186	POFUT1	133.5987129	166.6172356	286.3267806
ENSCAFG00000015671	CHERP	184.5473745	53.15396472	140.7970533
ENSCAFG00000004608	CLPTM1	536.6592364	642.9585348	760.7773551
ENSCAFG00000017954	TNIP1	510.6188093	420.1207596	370.3317452
ENSCAFG00000018663	TNFAIP1	53.21304664	90.975055	104.1188293
ENSCAFG00000011514	GTF2I	211.7199941	352.6561121	562.0050446
ENSCAFG00000035099		1.132192482	5.110958146	0
ENSCAFG00000046362		97.36855343	89.95286338	69.80694238
ENSCAFG00000005899	HARS	319.2782799	281.0720323	285.1436121
ENSCAFG00000001131	SLA	74.7247038	19.42164096	22.48020178
ENSCAFG00000008384	SLC38A7	22.64384964	41.9098568	23.6633703
ENSCAFG00000006424	GXYLT2	2.264384964	0	0
ENSCAFG00000012675	SELENBP1	3088.62109	3484.651264	3585.0006
ENSCAFG00000030792	CA13	13.58630978	7.155341405	7.09901109
ENSCAFG00000010542	B3GALT2	5.660962409	17.3772577	14.19802218
ENSCAFG00000018189		138.1274828	140.0402532	120.6831885

ENSCAFG00000015209	TUBA4A	435.8261739	217.9108115	262.9355391
ENSCAFG00000011202	LRP10	491.3715371	284.1692729	616.4307963
ENSCAFG00000007028	NAT10	117.7480181	98.13039641	153.8119069
ENSCAFG00000011484	GIGYF2	131.3343279	176.8391519	324.1881731
ENSCAFG00000029284	SAT1	2534.978967	2620.899337	1400.871522
ENSCAFG00000016177	CYB561A3	270.5940031	85.86409686	118.3168515
ENSCAFG00000008919	RASGEF1B	812.9142019	662.3801758	365.5990711
ENSCAFG00000017877	STRA6	0	0	0
ENSCAFG00000016964	MAZ	160.7713324	84.84190523	157.3614125
ENSCAFG00000009282	CREB3L1	16.98288723	2.044383259	2.36633703
ENSCAFG00000030412	BCL2L11	82.65005117	43.95424006	52.05941466
ENSCAFG00000045686		14.71850226	31.68794051	17.74752772
ENSCAFG00000030478	ABCF1	550.2455461	469.1859578	782.0743884
ENSCAFG00000018726	BCORL1	29.43700453	31.68794051	68.62377387
ENSCAFG00000014582	SERPINI1	76.98908876	36.79889865	3.549505545
ENSCAFG00000013084		319.2782799	148.2177862	305.2574769
ENSCAFG00000024953		19.24727219	108.3523127	67.44060535
ENSCAFG00000011153		2835.462851	1727.892286	2049.247868
ENSCAFG00000017950	LMAN1L	0	0	0
ENSCAFG00000047328		333.6005148	202.0259536	190.7267646
ENSCAFG00000018705	ZDHHC9	110.9548632	61.33149776	93.47031268
ENSCAFG00000006446	ULK1	2348.167207	231.0153082	597.5001001
ENSCAFG00000003845		2.264384964	0	7.09901109
ENSCAFG00000008608	DCTN1	1082.376013	654.2026427	1390.223005
ENSCAFG00000044597		18.11507971	73.59779731	27.21287584
ENSCAFG00000017942	MAPRE2	266.0652332	242.2594161	218.8861753
ENSCAFG00000015642	FOXM1	115.4836331	18.39944933	40.22772951
ENSCAFG00000006243	GNPDA1	122.276788	197.2829844	205.8713216
ENSCAFG00000009472	PTDSS1	597.7976304	349.609981	638.5915426
ENSCAFG00000006673	PPP1R3B	1817.168933	3560.293445	2043.332025
ENSCAFG00000032731		4.528769927	5.110958146	0
ENSCAFG00000043458	HIST2H2AA3	6.793154891	9.199724663	5.915842575
ENSCAFG00000016607	MSN	225.3063039	227.9487333	199.955479
ENSCAFG00000009116	IVD	1320.136434	2374.551155	1835.094367
ENSCAFG00000005008	MGAT5	12.4541173	10.22191629	11.83168515
ENSCAFG00000011398	CDH24	2.264384964	1.022191629	4.73267406
ENSCAFG00000004393	TMEM135	447.2160303	975.1708143	687.4209072

ENSCAFG00000018884	FGGY	581.9469356	502.9182816	570.2872242
ENSCAFG00000008188	RHOF	6.793154891	5.110958146	7.09901109
ENSCAFG00000029302		59.2589545	25.11524833	9.477179805
ENSCAFG00000010052	CTSD	1804.714816	1831.7674	1985.356768
ENSCAFG00000046073		39.62673686	24.5325991	14.19802218
ENSCAFG00000017178	POMT2	388.3420213	263.7254404	369.1485767
ENSCAFG00000008462		0	0	0
ENSCAFG00000005654	SLC26A11	106.4260933	54.17615635	79.2722905
ENSCAFG00000020086	CIZ1	107.5582858	78.70875545	92.28714417
ENSCAFG00000016991	SEZ6L2	0	0	4.73267406
ENSCAFG00000029121	FAM78A	7.925347372	1.022191629	5.915842575
ENSCAFG00000010696	ADD3	175.4898347	170.7060021	99.38615526
ENSCAFG00000011180	MXRA5	37.3623519	4.088766517	1.183168515
ENSCAFG00000005933	RFNG	461.9345326	263.7254404	584.4852464
ENSCAFG00000000909	TTLL12	417.7790258	275.9917399	557.2723706
ENSCAFG00000017389	RAB13	191.3405294	158.4397025	216.5198382
ENSCAFG00000023577		0	9.199724663	1.183168515
ENSCAFG00000010891	PGPEP1L	31.70138949	28.62136562	14.19802218
ENSCAFG00000011930	CCNDBP1	578.5503582	558.1166296	455.5198783
ENSCAFG00000013228	CNKSR2	0	0	0
ENSCAFG00000018661	LIFR	339.6577445	297.4577641	252.0148937
ENSCAFG00000005906	PYCR1	92.83978351	16.35506607	50.87624614
ENSCAFG00000014893	GJC3	0	0	0
ENSCAFG00000005665	KRR1	249.082346	227.9487333	117.133683
ENSCAFG00000005165	MGAT5B	0	0	0
ENSCAFG00000009095		343.054322	155.7308947	132.9053193
ENSCAFG00000016980	FAM189B	133.5987129	82.79752197	168.0099291
ENSCAFG00000020011	DBT	198.1336843	451.8087001	407.0099692
ENSCAFG00000008465	FMNL3	109.8226707	81.77533034	274.4950955
ENSCAFG00000009275	BIN3	33.96577445	45.99862332	54.42575169
ENSCAFG00000015840	ATP13A2	172.0932572	72.57560568	203.5049846
ENSCAFG00000005748	IDO2	1116.341787	1076.367786	857.7971734
ENSCAFG00000000588	ALDH7A1	8454.081261	10427.37681	10698.20971
ENSCAFG00000007859	ZCCHC8	101.8973234	147.1955946	137.2475477
ENSCAFG00000019497	NME3	6.793154891	7.155341405	0
ENSCAFG00000006448	TEX15	6.793154891	27.59917399	8.282179605
ENSCAFG00000028728	SERPINE3	190.2083369	77.68656382	136.0643792

ENSCAFG00000031295		19.24727219	14.31068281	11.83168515
ENSCAFG00000038248		9.057539854	2.044383259	21.29703327
ENSCAFG00000019440	MECP2	281.915928	255.5479073	324.1881731
ENSCAFG00000008425	DQX1	10.18973234	14.31068281	20.11386475
ENSCAFG00000007419	DNASE1L3	306.8241626	857.618777	341.9357008
ENSCAFG00000002276	ELOVL5	601.1942078	1155.076541	1077.866517
ENSCAFG00000009453	ALX4	0	1.022191629	0
ENSCAFG00000017373	RPS6KA6	0	1.022191629	0
ENSCAFG00000008476	NPY2R	0	8.177533034	1.183168515
ENSCAFG00000010032	HPGDS	2.264384964	6.133149776	2.36633703
ENSCAFG00000000219	TRIM36	104.1617083	147.1955946	102.9356608
ENSCAFG00000015228	VWF	475.5208423	55.19834798	74.53961644
ENSCAFG00000007045	ID1	245.6857685	96.08601315	57.97525723
ENSCAFG00000029847	LASP1	81.51785869	96.08601315	195.222805
ENSCAFG00000004537	AGBL5	29.43700453	29.64355725	57.97525723
ENSCAFG00000001596	FOXP4	62.2705865	31.68794051	92.28714417
ENSCAFG00000004382	MASTL	3.396577445	5.110958146	2.36633703
ENSCAFG00000002039	LNX1	83.78224365	101.1969713	102.9356608
ENSCAFG00000019869		54.34523913	70.53122242	50.87624614
ENSCAFG00000017214	ABCB7	250.2145385	472.2525327	409.3763062
ENSCAFG00000032597	MRPL50	670.2579492	801.3982373	692.1417496
ENSCAFG00000029072		3.396577445	5.110958146	4.73267406
ENSCAFG00000013075	SH3KBP1	38.49454438	13.28849118	29.57921287
ENSCAFG00000032693	AIF1	500.4290769	667.4911339	651.9258518
ENSCAFG00000012175	SLC26A6	37.3623519	29.64355725	27.21287584
ENSCAFG00000012941	TFRC	601.1942078	828.9974113	296.9752973
ENSCAFG00000047367		174.3236764	315.3461176	288.941583
ENSCAFG00000014888	DDI1	2.264384964	1.022191629	0
ENSCAFG00000042491		2.264384964	1.022191629	0
ENSCAFG00000032483	LY96	52.08085416	23.51040747	34.31188693
ENSCAFG00000049498		1.132192482	0	0
ENSCAFG00000024748	CD55	16.98288723	17.3772577	23.6633703
ENSCAFG00000014966	SMPD4	129.0699429	142.0846365	244.9158826
ENSCAFG00000005468	PFKP	120.0124031	22.48821584	27.21287584
ENSCAFG00000001818	DNAJA1	1309.482503	1540.003243	1283.205413
ENSCAFG00000009696	WFDC2	20.37946467	15.33287444	4.73267406
ENSCAFG00000031923		0	0	0

ENSCAFG00000018481	ATAD5	11.32192482	23.51040747	5.915842575
ENSCAFG00000010410		6285.932659	19049.5632	10747.90279
ENSCAFG00000000033		0	0	0
ENSCAFG00000004043	LAMA4	45.28769927	13.28849118	13.01485366
ENSCAFG00000010726	CLPTM1L	910.2827554	777.8878299	841.2328141
ENSCAFG00000010193	GUSB	8953.378146	13831.27494	11162.01177
ENSCAFG00000049023		11.32192482	0	4.73267406
ENSCAFG00000024269	RNASE13	1.132192482	3.066574888	0
ENSCAFG00000023159	C1H18orf63	3.396577445	1.022191629	1.183168515
ENSCAFG00000009605	PPM1K	106.4260933	239.1928412	128.9653681
ENSCAFG00000008690	RAPSN	23.77604212	10.22191629	10.64851663
ENSCAFG00000005718	E2F7	1.132192482	2.044383259	0
ENSCAFG00000008622		2.264384964	0	1.183168515
ENSCAFG00000050075		2.264384964	0	1.183168515
ENSCAFG00000016972	MED12	146.0528301	75.64218057	242.5495456
ENSCAFG00000015602		1.132192482	1.022191629	2.36633703
ENSCAFG00000008374	CCNH	306.8241626	345.5007707	340.7525323
ENSCAFG00000023629	BSCL2	406.457101	306.6574888	244.9158826
ENSCAFG00000013453		0	0	2.36633703
ENSCAFG00000001586	TREM2	11.32192482	0	5.915842575
ENSCAFG00000009880	SNCA	4.528769927	1.022191629	0
ENSCAFG00000014278	ABCA12	0	0	0
ENSCAFG00000011065	SGO2	27.17261956	44.97643169	47.3267406
ENSCAFG00000005731	NRG2	0	1.022191629	0
ENSCAFG00000011330	NEK7	186.8117595	477.3634909	301.7079713
ENSCAFG00000017037	MUC1	2.264384964	5.110958146	8.282179605
ENSCAFG00000000671	SOD2	3736.23519	5137.535129	5133.768186
ENSCAFG00000024890	NMT2	218.513149	136.9736783	145.5297273
ENSCAFG00000017621	CARM1	202.6624542	134.9292951	347.8515434
ENSCAFG00000002594	KMT5C	9.057539854	6.133149776	15.38119069
ENSCAFG00000016011	EPHA2	216.248764	73.59779731	61.52476278
ENSCAFG00000009691	AP2A2	361.1694017	402.7435019	743.0298274
ENSCAFG00000009144	CDH17	0	0	1.183168515
ENSCAFG00000014890	TUBG1	261.5364633	180.9279184	252.0148937
ENSCAFG00000012433	PER2	355.5084393	199.3273677	356.133723
ENSCAFG00000010657	HYAL2	215.1165715	158.4397025	146.7128959
ENSCAFG00000037162		0	0	0

ENSCAFG00000004741	GGA3	67.60321309	77.58434466	94.99660007
ENSCAFG00000014193	CERKL	388.3420213	33.73232377	414.1089802
ENSCAFG00000013596	CD69	38.49454438	7.155341405	5.915842575
ENSCAFG00000014612		69.56190608	10.22191629	9.358862953
ENSCAFG00000004731	TENM4	7.925347372	3.066574888	1.183168515
ENSCAFG00000019913	STXBP3	514.0153867	832.0639862	597.5001001
ENSCAFG00000001883	GABRA1	0	0	1.183168515
ENSCAFG00000007076	RET	2.264384964	0	0
ENSCAFG00000000765	AGPAT4	21.51165715	6.133149776	9.46534812
ENSCAFG00000001837	DNPH1	20.37946467	11.24410792	7.09901109
ENSCAFG00000014891	PGPEP1	285.3125054	215.6824338	170.3762662
ENSCAFG00000005854	PPFIA2	4.528769927	9.199724663	1.183168515
ENSCAFG00000016233	AGMAT	4426.872604	1839.944933	3122.381711
ENSCAFG00000009385	CHST1	2.264384964	5.110958146	1.183168515
ENSCAFG00000001938	VEGFA	1862.456633	1136.677092	1015.158586
ENSCAFG00000013154		917.0759102	187.0610682	294.6089602
ENSCAFG00000033265		4.528769927	2.044383259	0
ENSCAFG00000046925		0	0	1.183168515
ENSCAFG00000006072	RAB20	457.4057626	153.3287444	76.90595347
ENSCAFG00000000636	VARS	861.5984786	280.0805064	832.9506345
ENSCAFG00000011306	GTF2E1	47.55208423	98.13039641	66.25743684
ENSCAFG00000001748	PEX6	268.5787005	164.5932961	199.9909741
ENSCAFG00000016192	SDK1	11.32192482	3.066574888	0
ENSCAFG00000009396	MAPK10	15.85069474	7.155341405	7.09901109
ENSCAFG00000010397	IRS1	906.8861779	451.6042618	519.4819682
ENSCAFG00000005183	SLAIN1	14.71850226	10.22191629	9.46534812
ENSCAFG00000008917	LBP	1936.049144	1697.860296	1552.317092
ENSCAFG00000037555		5.660962409	3.066574888	3.549505545
ENSCAFG00000002435	HAAO	11105.67605	8526.10038	8659.610361
ENSCAFG00000014812	COL3A1	1540.913968	209.549284	468.5347319
ENSCAFG00000036814		0	0	0
ENSCAFG00000013450	HOXD8	1.132192482	0	0
ENSCAFG00000010381	MS4A1	4.528769927	2.044383259	4.73267406
ENSCAFG00000015587	KLHDC7A	22.64384964	11.24410792	43.77723505
ENSCAFG00000009687	PIGT	665.7291793	655.2248344	1138.208111
ENSCAFG00000001056	SQLE	4855.973554	1390.180616	2548.544981
ENSCAFG00000019974	CDH13	19.24727219	7.155341405	1.183168515

ENSCAFG00000018839	SHISA9	3.396577445	0	8.282179605
ENSCAFG00000011094	NR1I2	93.97197599	221.8155836	267.3960844
ENSCAFG00000008350	PKIA	1.132192482	2.044383259	1.183168515
ENSCAFG00000001445	GRINA	909.7732687	497.4188906	858.9803419
ENSCAFG00000013431	WDR76	15.85069474	27.59917399	9.46534812
ENSCAFG00000019460	TUBB4B	1702.817493	509.0514314	650.7426832
ENSCAFG00000005884	PGAP2	66.79935643	82.79752197	120.6831885
ENSCAFG00000010457	RIPOR2	86.04662862	42.93204843	16.56435921
ENSCAFG00000006941	CAT	40192.8331	57156.86714	52538.59791
ENSCAFG00000034431		0	0	0
ENSCAFG00000013546	DNAJB11	764.2299252	481.4522574	448.4208672
ENSCAFG00000006921	RBCK1	317.0138949	310.7462553	325.3713416
ENSCAFG00000013415	MAP3K11	141.5240602	149.2399779	211.7871642
ENSCAFG00000016405	TMEM132A	167.5644873	164.5728523	126.5990311
ENSCAFG00000019503	MBD3	266.0652332	145.1512114	162.0940866
ENSCAFG00000015325	AKAP3	1.132192482	1.022191629	1.183168515
ENSCAFG00000007230		0	0	0
ENSCAFG00000029972	TRIM62	7.925347372	18.39944933	22.48020178
ENSCAFG00000002941	ME1	93.97197599	143.1068281	146.7128959
ENSCAFG00000019264	CCNL2	367.9625566	224.8821584	196.4059735
ENSCAFG00000011698	TLR7	2.275706888	4.088766517	1.183168515
ENSCAFG00000017606	UNC79	9.057539854	4.088766517	2.36633703
ENSCAFG00000016479	TMEM184A	135.8630978	40.88766517	65.07426832
ENSCAFG00000010038	NUAK2	31.70138949	24.5325991	54.42575169
ENSCAFG00000000941	VPS52	247.9048658	244.1913583	320.6386676
ENSCAFG00000012918	TNK2	36.23015942	22.48821584	29.57921287
ENSCAFG00000016940	TBC1D10B	85.51449815	36.94200548	86.7617472
ENSCAFG00000012555	SLC12A8	13.58630978	0	5.915842575
ENSCAFG00000031788	RMDN1	230.9672663	358.7892619	319.455499
ENSCAFG00000006591	TBCB	163.0357174	156.3953193	112.4010089
ENSCAFG00000018054	TECPR2	35.09796694	66.4424559	84.00496456
ENSCAFG00000012130	CFLAR	377.0200964	673.6242837	414.1089802
ENSCAFG00000020156	RALGPS1	1.132192482	4.088766517	1.183168515
ENSCAFG00000020182	CDC7	22.64384964	39.86547354	9.46534812
ENSCAFG00000002532	ALG2	91.70759102	156.3953193	133.6980422
ENSCAFG00000032319		550.721067	2480.133328	2206.810419
ENSCAFG00000005789	HGS	328.3358197	165.5950439	267.3960844

ENSCAFG00000003404	CAV1	30.56919701	28.62136562	28.39604436
ENSCAFG00000014661	WNT4	126.805558	39.86547354	18.93069624
ENSCAFG00000010253	CIP2A	12.4541173	57.24273124	18.93069624
ENSCAFG00000003850	RHOB	5099.394938	1900.254239	1199.732874
ENSCAFG00000001722	PRPH2	0	0	0
ENSCAFG00000020151	SF3B3	585.3435131	539.7171803	525.3268206
ENSCAFG00000018353		221.1058698	239.4177234	268.5674212
ENSCAFG00000004870	PCDH17	16.98288723	13.63603633	23.76985547
ENSCAFG00000036634		438.1584904	444.6533587	313.5396565
ENSCAFG00000033364		0	0	0
ENSCAFG00000044896	GPR143	4.528769927	2.044383259	1.183168515
ENSCAFG00000014686	VIL1	4.528769927	2.044383259	2.36633703
ENSCAFG00000009058	TOP1	425.7043731	475.3191076	725.2822997
ENSCAFG00000007668	OGFOD2	238.8926137	208.5270924	209.4208272
ENSCAFG00000036241		0	0	0
ENSCAFG00000019411	OAZ1	2281.367851	2785.47219	2295.346919
ENSCAFG00000049540		0	0	0
ENSCAFG00000014185	MEN1	209.4556091	170.7060021	246.0990511
ENSCAFG00000019871	CLCC1	91.18678248	111.9299834	85.8862025
ENSCAFG00000007113	PCM1	225.3063039	409.8988433	306.4406454
ENSCAFG00000012159	ASB18	7.925347372	0	1.183168515
ENSCAFG00000009839	CD101	41.89112183	15.33287444	9.46534812
ENSCAFG00000043550		0	0	3.549505545
ENSCAFG00000030753	C32H4orf54	0	0	0
ENSCAFG00000014472	GPR137	192.4727219	128.7961453	184.5742883
ENSCAFG00000004721	PDCD6IP	341.9221295	519.2733477	398.7277895
ENSCAFG00000016363	PDPN	6.793154891	4.088766517	0
ENSCAFG00000012503	METTL27	691.7696064	982.3261557	941.8021379
ENSCAFG00000042397		8.559375162	0	0
ENSCAFG00000019541	TRAF2	82.65005117	110.396696	66.25743684
ENSCAFG00000048570		0	0	0
ENSCAFG00000018298	CCT6B	19.24727219	17.3772577	15.38119069
ENSCAFG00000001411	STK38	84.91443613	99.15258804	121.866357
ENSCAFG00000045017		59.99487961	22.34510902	43.75357168
ENSCAFG00000019908	PRRC2B	216.248764	108.3523127	411.7426432
ENSCAFG00000001700	SND1	1371.085095	1245.029404	1933.297353
ENSCAFG00000013984	CCDC103	2.264384964	3.066574888	3.549505545

ENSCAFG00000010602	ELF3	70.19593387	52.13177309	29.57921287
ENSCAFG00000049892		5.660962409	0	0
ENSCAFG00000043524		0	2.044383259	1.183168515
ENSCAFG00000001969	ANKIB1	74.7247038	153.3287444	108.8515034
ENSCAFG00000005435		208.3234166	218.7490087	207.0544901
ENSCAFG00000047436		41.89112183	42.93204843	39.04456099
ENSCAFG00000015182	GLB1L	147.1850226	137.99587	130.1485366
ENSCAFG00000043711		1.143514407	5.254064974	1.206831885
ENSCAFG00000001588		103.006872	70.77654841	51.85827601
ENSCAFG00000024978	LRRN4	3.396577445	0	22.48020178
ENSCAFG00000018982	MYO5B	1825.094281	1379.9587	1270.722985
ENSCAFG00000010617	ALDH1A3	116.6158256	69.50903079	35.49505545
ENSCAFG00000029395		1964.987984	2351.90961	3239.255097
ENSCAFG00000028971	CCDC85C	33.96577445	51.10958146	76.90595347
ENSCAFG00000002029	SLC25A27	30.56919701	34.7545154	33.12871842
ENSCAFG00000001029	TPD52L1	78.12128124	70.53122242	89.92080714
ENSCAFG00000003880	PPP1R15A	350.9796694	418.0763764	137.2475477
ENSCAFG00000015735	EPS15L1	133.5987129	147.1955946	136.0643792
ENSCAFG00000009547	PNPLA2	2471.576188	415.0098015	831.767466
ENSCAFG00000003438	COBL	243.4213836	263.7254404	344.3020379
ENSCAFG00000013060	PDK1	755.1723853	1543.110705	1478.369059
ENSCAFG00000012029	TGM5	0	0	0
ENSCAFG00000015155	TIMP1	166.4322948	64.39807264	81.63862753
ENSCAFG00000011796	MCF2L2	2.264384964	4.088766517	0
ENSCAFG00000004082	ABTB1	430.1199238	422.1242552	542.3289522
ENSCAFG00000005728	GINS4	21.51165715	18.39944933	10.64851663
ENSCAFG00000007782	MYH7B	121.1445956	56.22053961	178.6584458
ENSCAFG00000006851	SLC38A9	24.9082346	52.13177309	34.31188693
ENSCAFG00000013383	PCNX3	211.7199941	104.2635462	363.2327341
ENSCAFG00000003180	SLC1A4	96.23636095	162.5284691	151.4455699
ENSCAFG00000018290	XRCC3	55.47743161	19.42164096	16.56435921
ENSCAFG00000007923	MSC	15.85069474	17.3772577	18.93069624
ENSCAFG00000008600	DNAJC22	1116.341787	1899.232047	1417.435881
ENSCAFG00000008483	NDRG4	61.13839402	74.61998894	60.34159426
ENSCAFG00000006140	RNF24	3.396577445	6.133149776	3.549505545
ENSCAFG00000032401		10.18973234	29.64355725	9.46534812
ENSCAFG00000010972	TCIRG1	238.8926137	141.0624448	256.7475677

ENSCAFG00000011618	RAD9A	32.83358197	30.66574888	84.00496456
ENSCAFG00000015710	UBXN1	961.231417	902.5952086	810.4704328
ENSCAFG00000011636	FAM241A	29.43700453	27.59917399	23.6633703
ENSCAFG00000017772	C9H17orf64	53.21304664	16.35506607	11.83168515
ENSCAFG00000013808	KIF1BP	76.98908876	122.6629955	113.5841774
ENSCAFG00000005944		0	1.022191629	0
ENSCAFG00000044169	MROH6	0	0	0
ENSCAFG00000041779		15.85069474	11.24410792	13.01485366
ENSCAFG00000004621	DDIAS	1.132192482	0	0
ENSCAFG00000002835	CTNNAL1	1536.385198	1600.752091	1714.411178
ENSCAFG00000015757	CNP	347.5830919	163.5506607	236.633703
ENSCAFG00000023924	CD151	1341.648091	1004.814372	1247.059615
ENSCAFG00000012302	DPAGT1	198.1336843	240.2150329	182.2079513
ENSCAFG00000001834	CUL9	269.4618107	113.4632708	250.8317252
ENSCAFG00000000609	ADM2	1.132192482	0	0
ENSCAFG00000007126	HPN	5651.904869	4000.858037	4490.124514
ENSCAFG00000000058	SERPINB8	6.793154891	5.110958146	0
ENSCAFG00000014029	IL1RAP	772.1552726	1418.801981	1200.916043
ENSCAFG00000030537	HSPB8	22.64384964	7.155341405	8.282179605
ENSCAFG00000011260	CSE1L	206.0590317	396.6103522	315.9059935
ENSCAFG00000029647		484.1028614	1720.041855	3460.554936
ENSCAFG00000030170	ZNF229	2.264384964	0	1.183168515
ENSCAFG00000030769	SLC35F6	66.79935643	43.95424006	65.07426832
ENSCAFG00000010598	TRIP12	868.3916335	847.3968607	1398.505185
ENSCAFG00000019565	CLCN7	278.5193505	227.9487333	330.1040157
ENSCAFG00000012990	FARP2	16.98288723	5.110958146	8.282179605
ENSCAFG00000007868	LACTB2	2093.423899	2298.908974	2251.569684
ENSCAFG00000001433	PLEC	1363.544694	586.4211158	3017.245357
ENSCAFG00000003403	PDIA4	4405.360947	3034.886947	3466.683749
ENSCAFG00000016546	PCGF2	54.34523913	36.79889865	56.79208872
ENSCAFG00000004655	BCL3	43.02331431	23.51040747	15.38119069
ENSCAFG00000009819	CTSA	1192.266615	947.2343171	1483.279209
ENSCAFG00000007112		643.8552205	1005.223248	806.4476598
ENSCAFG00000005973	SIPA1L3	37.3623519	20.44383259	61.52476278
ENSCAFG00000047691		181.8301126	323.5747602	344.7871369
ENSCAFG00000011617	SF3B4	212.8521866	145.1512114	176.2921087
ENSCAFG00000016714	TP53	356.6406318	243.2816078	365.5990711

ENSCAFG00000043745		65.66716394	5.110958146	3.549505545
ENSCAFG00000031572	INKA1	4.528769927	6.133149776	8.282179605
ENSCAFG00000014264	BARD1	180.1431458	730.0901493	397.9823934
ENSCAFG00000008682		60.87798975	160.4943077	138.4307163
ENSCAFG00000000145	RAB27B	67.93154891	60.30930613	67.44060535
ENSCAFG00000000355	MGAT4B	519.6763491	361.8558368	646.0100092
ENSCAFG00000018725	SKP2	3.396577445	26.57698236	39.04456099
ENSCAFG00000002858	DBNL	560.4352785	483.4966406	562.0050446
ENSCAFG00000013883		3378.462366	4660.171638	4661.683949
ENSCAFG00000013439	SLC5A1	27.17261956	36.79889865	3.549505545
ENSCAFG00000030397	NACC2	115.4836331	57.24273124	121.866357
ENSCAFG00000018035	SNUPN	69.06374139	98.13039641	62.70793129
ENSCAFG00000005216	ULK4	44.15550679	23.51040747	34.31188693
ENSCAFG00000000487	DLA88	5020.549054	5571.230593	4505.103428
ENSCAFG00000030721	COL10A1	62.2705865	12.26629955	2.36633703
ENSCAFG00000029087	GATA4	56.60962409	72.57560568	105.3019978
ENSCAFG00000011989	SLC49A4	109.8226707	228.970925	259.1139048
ENSCAFG00000008366	AQP6	0	3.066574888	3.549505545
ENSCAFG00000042483		21.51165715	28.62136562	21.29703327
ENSCAFG00000014951	NSD2	107.5582858	143.1068281	141.9802218
ENSCAFG00000023226	CCL16	10676.5751	9625.978573	9946.897705
ENSCAFG00000028867	ZNF22	80.38566621	147.1955946	147.8960644
ENSCAFG00000001306	LRGUK	4.528769927	3.066574888	13.01485366
ENSCAFG00000007897	PLS1	194.7371069	426.2539094	315.9059935
ENSCAFG00000009214	DCBLD2	151.7137926	82.79752197	131.3317052
ENSCAFG00000013343	ELL3	1.132192482	3.066574888	1.183168515
ENSCAFG00000002289	BICD2	64.53497146	94.04162989	74.53961644
ENSCAFG00000008276	HLTF	86.04662862	248.3925659	178.6584458
ENSCAFG00000015259	DPT	134.7309053	45.99862332	3.549505545
ENSCAFG00000018939	DROSHA	107.5582858	58.26492287	104.1188293
ENSCAFG00000008099	CYP2C18	32043.31162	42721.47695	35873.66937
ENSCAFG00000005330	SH2D4A	28.30481204	38.84328191	40.22772951
ENSCAFG00000007667	USP39	82.65005117	115.5076541	107.6683349
ENSCAFG00000000235	N4BP3	2.264384964	4.088766517	4.73267406
ENSCAFG00000011915		27.39905806	0	40.41703647
ENSCAFG00000004225	C21H11orf54	3763.407809	5550.500547	3335.352044
ENSCAFG00000005743	INPPL1	266.0652332	189.1054514	360.8663971

ENSCAFG00000017372	ANKFN1	4.528769927	7.155341405	0
ENSCAFG00000015107	OTUD3	26.04042708	30.66574888	16.56435921
ENSCAFG00000013641	DUSP10	180.0186046	166.6172356	46.14357208
ENSCAFG00000002577	MSANTD3-TME	146.0528301	69.50903079	87.55447011
ENSCAFG00000046815		194.7371069	331.1900879	281.5941066
ENSCAFG00000010370	MS4A7	3.396577445	17.3772577	4.73267406
ENSCAFG00000049307	CRB3	221.9097264	193.1942179	133.6980422
ENSCAFG00000001130	ITPR3	66.79935643	44.97643169	160.910918
ENSCAFG00000044898		19.24727219	31.68794051	15.38119069
ENSCAFG00000007586	MAST4	13.12211086	6.133149776	8.282179605
ENSCAFG00000015652	ERAS	2.264384964	2.044383259	1.183168515
ENSCAFG00000011937	PGBD1	1.132192482	7.155341405	2.36633703
ENSCAFG00000016692	SHBG	1693.759953	1070.234636	1942.762702
ENSCAFG00000042787	MCOLN3	11.32192482	13.28849118	2.36633703
ENSCAFG00000047614		2.830481204	1.022191629	1.183168515
ENSCAFG00000002440		13.85803598	7.451776977	0
ENSCAFG00000004408	CAB39L	16.98288723	3.066574888	16.56435921
ENSCAFG00000001757	GPR37	0	0	0
ENSCAFG00000011486	SPATA2	20.37946467	24.5325991	24.84653881
ENSCAFG00000009956	TMEM81	0	0	0
ENSCAFG00000003604	RRAS	293.2378528	150.2621695	112.4010089
ENSCAFG00000001299	ZC3H3	70.19593387	57.24273124	70.9901109
ENSCAFG00000025252	MOGAT2	2.264384964	2.044383259	3.549505545
ENSCAFG00000041926	CLEC10A	6064.022932	5214.199501	4910.149337
ENSCAFG00000003784	ARMC2	10.18973234	28.62136562	29.57921287
ENSCAFG00000009653	DOP1B	96.23636095	93.01943826	146.7128959
ENSCAFG00000000122	APOF	272.8583881	1072.279019	666.1238739
ENSCAFG00000001513	KCTD17	2.264384964	0	2.36633703
ENSCAFG00000013650	CTTNBP2NL	52.08085416	79.73094708	75.72278496
ENSCAFG00000011354		7.925347372	3.066574888	4.73267406
ENSCAFG00000031667	ADH4	12763.20585	23090.28671	21830.64227
ENSCAFG00000008947	RALGAPB	69.06374139	93.01943826	153.8119069
ENSCAFG00000016473	NSD1	198.1336843	223.8599668	347.8515434
ENSCAFG00000024246	LRRC8D	844.6155914	321.9903632	521.7773151
ENSCAFG00000020377	EXOC3L1	27.17261956	18.39944933	7.09901109
ENSCAFG00000001736	CNPY3	242.2891911	363.90022	283.9604436
ENSCAFG00000032730		2.264384964	2.044383259	3.549505545

ENSCAFG00000018741	NAPG	261.5364633	459.9862332	356.133723
ENSCAFG00000048834		1.132192482	1.022191629	1.183168515
ENSCAFG00000047686		2.264384964	0	1.183168515
ENSCAFG00000034030		1.132192482	0	1.183168515
ENSCAFG00000041918	ATG9B	0	0	3.549505545
ENSCAFG00000001809	TOPORS	369.0947491	386.3884359	304.0743083
ENSCAFG00000031845	CGRRF1	82.65005117	294.3911892	173.9257717
ENSCAFG00000038120		0	0	0
ENSCAFG00000046749		0	0	0
ENSCAFG00000043715	SNX27	54.34523913	87.90848012	123.0495256
ENSCAFG00000001191	LEMD2	84.91443613	67.46464753	114.767346
ENSCAFG00000015876	CNST	99.6329384	274.9695483	175.1089402
ENSCAFG00000012419	NSMCE4A	109.8226707	52.13177309	95.83664971
ENSCAFG00000012628	TMEM25	121.1445956	110.396696	50.87624614
ENSCAFG00000030711		204.9268392	170.7060021	147.8960644
ENSCAFG00000006126	STAR	0	0	0
ENSCAFG00000000936	PACSIN2	207.1912242	182.9723016	162.0940866
ENSCAFG00000018931	CIITA	20.37946467	8.177533034	26.02970733
ENSCAFG00000017428	SWSAP1	16.98288723	11.24410792	20.11386475
ENSCAFG00000001211	HMGA1	114.3514407	114.4854625	147.8960644
ENSCAFG00000043925		2.46817961	2.044383259	0
ENSCAFG00000013855		781.2014905	1950.290519	2406.564759
ENSCAFG00000050055		56.60962409	5.110958146	5.915842575
ENSCAFG00000017611	UACA	711.0168786	687.9349665	653.1090203
ENSCAFG00000008186		491.3715371	471.2303411	370.3317452
ENSCAFG00000006068	ADRA1D	9.057539854	4.088766517	4.73267406
ENSCAFG00000018305	LLGL1	56.60962409	47.02081495	76.90595347
ENSCAFG00000005595	FARP1	868.3916335	826.9530281	1361.826961
ENSCAFG00000015272	FGFBP1	66.79935643	0	22.48020178
ENSCAFG00000005015	CCDC97	44.15550679	41.9098568	61.52476278
ENSCAFG00000030910	NEU2	0	2.044383259	0
ENSCAFG00000016770	HSD3B7	4634.063828	4590.662607	6261.327781
ENSCAFG00000040590		0	0	0
ENSCAFG00000030371	COMMD7	962.3636095	617.4037441	928.7872843
ENSCAFG00000003557	KIAA0319L	218.513149	214.6602421	442.5050246
ENSCAFG00000011207	DAG1	510.6188093	541.7615635	1093.247708
ENSCAFG00000014372	G6PC3	13.58630978	17.3772577	15.38119069

ENSCAFG00000009150	OGFOD1	65.66716394	87.90848012	94.6534812
ENSCAFG00000019475	NDOR1	54.34523913	50.08738983	87.55447011
ENSCAFG00000043309		60.00620153	64.39807264	88.73763862
ENSCAFG00000013433	WNT9B	18.11507971	15.33287444	22.48020178
ENSCAFG00000030882	TRAT1	7.925347372	4.088766517	3.549505545
ENSCAFG00000013171	RPS6KA3	131.3343279	228.970925	208.2376586
ENSCAFG00000011243	PRMT5	262.6686558	355.722687	363.2327341
ENSCAFG00000000943	ARFGAP3	842.3512064	1352.359526	1363.010129
ENSCAFG00000014134	FZD2	1.132192482	0	0
ENSCAFG00000015653	INHA	4.528769927	0	0
ENSCAFG00000029939		4.528769927	0	0
ENSCAFG00000007683	AADAT	4156.278601	11418.90269	7193.664571
ENSCAFG00000002968	JOSD2	0	0	0
ENSCAFG00000044676		0	3.066574888	0
ENSCAFG00000009325	WDR75	528.733889	326.0791297	270.9455899
ENSCAFG00000005225	PLB1	1.132192482	0	0
ENSCAFG00000002324	NOL8	149.4494076	160.4840858	189.3069624
ENSCAFG00000011850	KLHL24	240.0248061	894.4176756	435.4060135
ENSCAFG00000017532	SEPTIN4	677.0511041	271.9029734	231.9010289
ENSCAFG00000014498	PVRIG	5.660962409	1.022191629	5.915842575
ENSCAFG00000002131	ERMP1	69.06374139	168.6616188	131.3317052
ENSCAFG00000044144		0	0	0
ENSCAFG00000016769	NES	92.83978351	27.59917399	40.22772951
ENSCAFG00000012968	HELZ2	106.4260933	18.39944933	54.42575169
ENSCAFG00000015467	CHRM1	0	1.022191629	0
ENSCAFG00000002024		0	0	4.73267406
ENSCAFG00000003265	COLEC11	1054.071201	523.3621142	514.678304
ENSCAFG00000005438	SLC39A2	1.132192482	10.22191629	0
ENSCAFG00000015430	ABHD8	58.18337164	43.33070316	56.54362333
ENSCAFG00000011767	CPNE6	25033.81739	15147.60221	24891.28625
ENSCAFG00000017077	TTLL5	91.70759102	87.90848012	92.28714417
ENSCAFG00000003417		0	1.042635462	0
ENSCAFG00000039137		0	1.022191629	0
ENSCAFG00000047531		0	1.022191629	0
ENSCAFG00000014323	EPHB2	1.132192482	0	0
ENSCAFG00000006502	TPP1	260.4042708	203.4161342	539.5248428
ENSCAFG00000015201	SNAP29	56.60962409	124.7073788	127.7821996

ENSCAFG00000016574	PIP4K2B	99.6329384	156.3953193	191.6732994
ENSCAFG00000043229	MRPS18C	407.5892934	302.5687223	299.3416343
ENSCAFG00000010790	SLC9B1	12.4541173	14.31068281	4.73267406
ENSCAFG00000014907		0	3.066574888	3.549505545
ENSCAFG00000016839	SMG5	339.6577445	228.970925	349.0347119
ENSCAFG00000008801	CCDC65	1.132192482	0	0
ENSCAFG00000029004		12894.54018	11768.49223	10274.63538
ENSCAFG00000028587		12.4541173	14.31068281	23.6633703
ENSCAFG00000006703	UHRF1BP1L	622.705865	1874.699448	1282.55467
ENSCAFG00000009348	SYT6	1.132192482	1.022191629	0
ENSCAFG00000020296		1.132192482	1.022191629	0
ENSCAFG00000047086		1.132192482	1.022191629	0
ENSCAFG00000047992		1.132192482	1.022191629	0
ENSCAFG00000029467		1.607713324	0	0
ENSCAFG00000023881	ZNF227	24.9082346	21.46602421	18.93069624
ENSCAFG00000046600		4.562735702	1.083523127	3.797970933
ENSCAFG00000018761	PIEZO2	2.264384964	0	0
ENSCAFG00000029657		222.8268023	373.7030377	414.4875942
ENSCAFG00000032114	RCE1	40.75892934	45.99862332	66.25743684
ENSCAFG00000015313		23.77604212	3.066574888	13.01485366
ENSCAFG00000029211	RBAK	89.44320606	82.79752197	76.90595347
ENSCAFG00000023236	ACSM4	5398.293753	5700.762716	6042.441606
ENSCAFG00000049972		23.77604212	10.22191629	10.64851663
ENSCAFG00000047810	PLAC8	32.83358197	5.110958146	14.19802218
ENSCAFG00000029568		1303.968725	90.19818937	157.6098779
ENSCAFG00000003488	PAPPA	2.264384964	1.022191629	0
ENSCAFG00000023594		1478.643381	1179.60914	1163.05465
ENSCAFG00000008000	EIF6	3427.146642	1971.807653	2827.772751
ENSCAFG00000005393	MAP3K10	15.85069474	5.110958146	7.09901109
ENSCAFG00000009509	F13A1	71.32812635	36.79889865	9.46534812
ENSCAFG00000005855	TMCO6	163.0357174	214.6602421	153.8119069
ENSCAFG00000045397		0	1.022191629	1.183168515
ENSCAFG00000050065		0	1.022191629	1.183168515
ENSCAFG00000018868	MYO18A	658.9360244	422.1651429	1088.515034
ENSCAFG00000012665	TOR1AIP1	920.4724877	1369.736783	1215.114065
ENSCAFG00000010785	RBM20	1.132192482	1.022191629	4.73267406
ENSCAFG00000009634	NFKBIZ	331.7323972	222.8377752	88.73763862

ENSCAFG00000008022	WDR66	12.4541173	9.199724663	10.64851663
ENSCAFG00000000932	SLC35F1	1.132192482	0	0
ENSCAFG00000003820		1.132192482	0	0
ENSCAFG00000015376	GHSR	1.132192482	0	0
ENSCAFG00000039409		1.132192482	0	0
ENSCAFG00000044277		1.132192482	0	0
ENSCAFG00000048956		1.132192482	0	0
ENSCAFG00000049311		1.132192482	0	0
ENSCAFG00000030628		0	0	0
ENSCAFG00000003825	MATN3	0	1.022191629	0
ENSCAFG00000007085	SOAT2	95.10416847	121.6408039	263.8465788
ENSCAFG00000000429	CCDC170	793.6669297	1079.434361	468.5347319
ENSCAFG00000036856		41.89112183	66.4424559	132.5148737
ENSCAFG00000003620	RCN3	56.60962409	20.44383259	27.21287584
ENSCAFG00000017474	MPO	5.660962409	0	1.183168515
ENSCAFG00000015612	GHDC	60.00620153	52.13177309	72.17327941
ENSCAFG00000009556	CAGE1	0	0	0
ENSCAFG00000026372	SNORA12	0	0	1.183168515
ENSCAFG00000010254	SFXN2	914.8115253	1094.767235	1170.153661
ENSCAFG00000001572	UNC5CL	5.660962409	2.044383259	1.183168515
ENSCAFG00000020235	STRBP	244.5535761	236.0547129	304.0743083
ENSCAFG00000049738		0	0	0
ENSCAFG00000003426	TNC	412.1180634	217.726817	288.6931177
ENSCAFG00000017668	TUBD1	14.71850226	37.82109028	14.19802218
ENSCAFG00000031747	FBXO42	45.28769927	62.35368939	44.96040357
ENSCAFG00000006632	TNKS	39.62673686	64.39807264	95.83664971
ENSCAFG00000023536		2.264384964	6.133149776	4.73267406
ENSCAFG00000010198	RAB7B	6.793154891	0	1.183168515
ENSCAFG00000041383		1740.179844	1445.378964	1501.440846
ENSCAFG00000007145	TM9SF4	301.1632002	244.3037994	564.3713816
ENSCAFG00000001997	CWH43	1.132192482	0	1.183168515
ENSCAFG00000017890	SLC36A2	1.132192482	0	1.183168515
ENSCAFG00000026176	SNORD8	1.132192482	0	1.183168515
ENSCAFG00000044019		1.132192482	0	1.183168515
ENSCAFG00000003611	PRR12	52.08085416	29.64355725	115.9505145
ENSCAFG00000018762	DUS3L	249.0257364	124.6971569	151.4337382
ENSCAFG00000001066		0	0	0

ENSCAFG00000001911	ALDH1L2	10.18973234	5.110958146	5.915842575
ENSCAFG00000014586	USP5	542.3201988	468.1637662	578.5694038
ENSCAFG00000012986	ADAM12	11.32192482	14.31068281	21.29703327
ENSCAFG00000012542	HEG1	43.02331431	24.5325991	50.87624614
ENSCAFG00000024903	MAFB	2345.902822	220.7933919	181.0247828
ENSCAFG00000023413	TTC32	155.11037	113.4632708	85.18813308
ENSCAFG00000010917	ARHGAP31	66.79935643	39.86547354	68.62377387
ENSCAFG00000012969	INPP5J	0	0	0
ENSCAFG00000006946		272.8583881	370.0333698	416.4753173
ENSCAFG00000005792	LRTOMT	38.49454438	56.22053961	22.48020178
ENSCAFG00000029357	NCEH1	433.6297205	440.5645922	765.5100292
ENSCAFG00000007052	MALL	2.264384964	2.044383259	2.36633703
ENSCAFG00000016797	PGD	1110.680825	1902.298622	1990.089442
ENSCAFG00000013677	POLA2	143.7884452	171.7281937	182.2079513
ENSCAFG00000013434	FAM43A	16.98288723	6.133149776	1.56178244
ENSCAFG00000042542	ICAM3	154.1480064	198.5198363	145.5652224
ENSCAFG00000019811	SPIRE2	32.83358197	72.57560568	39.04456099
ENSCAFG00000003435	NOL10	2230.419189	1716.259746	1706.128999
ENSCAFG00000004046	LIG1	83.78224365	72.57560568	61.52476278
ENSCAFG00000004290	SAP130	56.60962409	50.08738983	101.7524923
ENSCAFG00000012557	NCSTN	566.0962409	360.8336451	551.356528
ENSCAFG00000019674	PARK7	4533.298697	4348.403191	5623.599952
ENSCAFG00000005550	PAF1	317.0138949	166.6172356	254.3812307
ENSCAFG00000010482	SLC19A3	12.4541173	12.26629955	9.46534812
ENSCAFG00000018249	ESCO1	213.9843791	252.4813324	215.3366697
ENSCAFG00000009514	YWHAB	486.8427672	432.3870592	392.811947
ENSCAFG00000016125	WIPF2	31.70138949	53.15396472	62.70793129
ENSCAFG00000009591	TOMM34	33.96577445	39.86547354	66.25743684
ENSCAFG00000009390	PPP3CC	23.77604212	39.86547354	34.31188693
ENSCAFG00000041137		0	0	0
ENSCAFG00000013425	EIF2S3	2330.052128	2754.305567	2918.876726
ENSCAFG00000002989	KIAA1841	463.066725	752.3330391	605.7822797
ENSCAFG00000010856	PSMD1	846.8799764	810.597962	977.2971934
ENSCAFG00000009374	FITM2	110.9548632	141.0624448	183.3911198
ENSCAFG00000049411		2.320994588	4.150098015	2.449158826
ENSCAFG00000007324	ASCL1	149.4494076	29.64355725	14.19802218
ENSCAFG00000013234	LAMC1	302.2953926	148.2177862	227.1683549

ENSCAFG00000011438	SCN9A	13.58630978	15.70086343	11.83168515
ENSCAFG00000000078	DGKA	88.31101358	26.57698236	61.52476278
ENSCAFG00000009977	CAP2	109.8226707	175.8169602	241.3663771
ENSCAFG00000049322		40.75892934	24.5325991	30.76238139
ENSCAFG00000016138	FBLIM1	46.41989175	6.133149776	8.282179605
ENSCAFG00000002375		0	0	0
ENSCAFG00000031152		39.62673686	14.31068281	11.83168515
ENSCAFG00000016411	CDK12	183.415182	131.8627202	244.9158826
ENSCAFG00000018510	LAMP2	5975.711919	6959.080612	9969.377907
ENSCAFG00000014289	DNAJC10	612.5161326	757.4439973	739.4803219
ENSCAFG00000010239	IKBKE	140.3918677	109.3745043	140.7970533
ENSCAFG00000011203	CYP2U1	333.9967821	1096.811618	795.0892421
ENSCAFG00000029176		0	0	0
ENSCAFG00000028759	BAG2	79.25347372	157.4175109	108.8515034
ENSCAFG00000008573	SLA2	16.98288723	0	3.549505545
ENSCAFG00000004647	TMEM214	406.457101	298.4799557	493.3812707
ENSCAFG00000031142	FAM222B	41.89112183	21.46602421	53.24258317
ENSCAFG00000014577	VEGFB	194.7371069	71.55341405	115.9505145
ENSCAFG00000012666	F11R	463.066725	537.672797	837.6833086
ENSCAFG00000013651	UPK3B	0	1.022191629	34.31188693
ENSCAFG00000010095	ATP6V1B2	357.7728242	819.7976867	655.4753573
ENSCAFG00000020017	CENPN	12.4541173	12.26629955	5.915842575
ENSCAFG00000045258		0	0	0
ENSCAFG00000031661		10.18973234	12.26629955	13.01485366
ENSCAFG00000014573	P4HA1	381.5488664	742.1111228	257.9307363
ENSCAFG00000016225	ACAP1	70.19593387	26.57698236	28.39604436
ENSCAFG00000008575	CUX2	16.98288723	1.022191629	17.74752772
ENSCAFG00000012449	USP53	189.0761445	187.0610682	117.133683
ENSCAFG00000013329	LUZP1	44.15550679	107.3301211	101.7524923
ENSCAFG00000032530	RGS18	44.15550679	86.88628849	48.50990911
ENSCAFG00000007624	NMNAT3	31.70138949	19.42164096	43.77723505
ENSCAFG00000032259		11.32192482	1.022191629	1.183168515
ENSCAFG00000008248	ACTR8	609.1195552	536.6506054	506.3961244
ENSCAFG00000023663		1.132192482	9.199724663	4.73267406
ENSCAFG00000044849		6.793154891	6.133149776	21.29703327
ENSCAFG00000017831	CCNK	55.47743161	34.7545154	47.3267406
ENSCAFG00000016038	LDB3	0	0	0

ENSCAFG00000017105	PIF1	305.6919701	191.1498347	281.5941066
ENSCAFG00000005417	RNF170	37.29442035	73.77156988	61.03966369
ENSCAFG00000002854	IGLON5	2.264384964	1.022191629	4.73267406
ENSCAFG00000006996	CCDC92	44.15550679	34.7545154	35.49505545
ENSCAFG00000037206		6.793154891	2.044383259	0
ENSCAFG00000023610	SERPINA4	13726.70165	17497.87631	27303.97982
ENSCAFG00000003710	CRYBG1	296.6344302	199.3273677	527.6931577
ENSCAFG00000048362		0	0	0
ENSCAFG00000001417	KIF27	170.9610647	114.4854625	65.07426832
ENSCAFG00000009569	SPP1	206.0590317	339.3676209	255.5643992
ENSCAFG00000010641	SLC17A2	843.4833989	1077.389977	577.3862353
ENSCAFG00000019906	JPH3	6.793154891	0	1.183168515
ENSCAFG00000017293	CEP128	12.4541173	3.066574888	4.73267406
ENSCAFG00000006660	SLC7A1	58.87400905	18.39944933	29.57921287
ENSCAFG00000012890	HAT1	99.6329384	119.6066425	105.3493246
ENSCAFG00000016970	MVP	459.6701476	344.4785791	340.7525323
ENSCAFG00000012525	MUC13	0	0	0
ENSCAFG00000018208	ABCC1	6.793154891	7.155341405	4.73267406
ENSCAFG00000009588	CHUK	224.1741114	340.3898125	235.4505345
ENSCAFG00000005003	NCKAP5	7.925347372	14.31068281	10.64851663
ENSCAFG00000008696	RBL1	16.98288723	44.97643169	47.3267406
ENSCAFG00000005075	R3HDM1	45.28769927	68.48683916	91.10397565
ENSCAFG00000040884		0	0	0
ENSCAFG00000024468		0	0	3.135396565
ENSCAFG00000005999	NHLRC3	49.8164692	49.0651982	37.86139248
ENSCAFG00000018076	ZSWIM7	57.74181657	76.6643722	50.87624614
ENSCAFG00000017040	FOS	1561.293432	2007.58436	40.22772951
ENSCAFG00000018017	RNF138	116.6158256	112.4410792	117.133683
ENSCAFG00000006883	GAS2L3	0	3.066574888	7.09901109
ENSCAFG00000023636	PRSS22	4.528769927	1.022191629	0
ENSCAFG00000005553	OXTR	0	2.044383259	5.915842575
ENSCAFG00000049581		0	0	0
ENSCAFG00000010131	GBF1	234.3638437	206.4827091	533.6090003
ENSCAFG00000014337	BLOC1S4	166.4322948	127.7739537	169.1930976
ENSCAFG00000025364	INTS6	1405.05087	475.3191076	683.8714017
ENSCAFG00000015035	KIF17	0	0	0
ENSCAFG00000011655	SSH3	110.9548632	125.7295704	172.7426032

ENSCAFG00000030285	CDR2L	65.66716394	19.42164096	26.02970733
ENSCAFG00000007309	PAX8	121.1445956	245.325991	309.9901509
ENSCAFG00000008290		19.49635454	15.24087719	12.55341794
ENSCAFG00000000001	ENPP1	756.3045778	894.4176756	1152.406134
ENSCAFG00000014631	RUNDC1	50.94866168	62.35368939	36.67822396
ENSCAFG00000013663	GNAZ	2.264384964	0	0
ENSCAFG00000000674	ZBTB12	13.58630978	12.26629955	21.29703327
ENSCAFG00000004875	IPO13	201.5302618	146.173403	270.9455899
ENSCAFG00000013454	MOV10	465.33111	317.9015967	537.1585058
ENSCAFG00000015131	CEP126	5.660962409	5.110958146	1.183168515
ENSCAFG00000003198	FKBP15	2277.971273	582.6492287	556.089202
ENSCAFG00000009907	NTM	3.396577445	0	0
ENSCAFG00000038534		19.24727219	4.088766517	5.915842575
ENSCAFG00000000913	TSPO	795.9313147	566.2941626	453.1535412
ENSCAFG00000009314	NQO2	1393.728945	1039.568887	1684.831965
ENSCAFG00000012197	CCDC14	65.66716394	143.1068281	106.4851663
ENSCAFG00000018793	HSD11B1L	6.793154891	7.155341405	5.915842575
ENSCAFG00000000948	HSPA4	743.8504605	735.9779731	901.5744084
ENSCAFG00000028789	GADL1	0	1.022191629	3.549505545
ENSCAFG00000011911	COL6A1	1575.785496	282.1248897	448.2315602
ENSCAFG00000019776	ADAMTS13	547.9811612	153.3287444	250.8317252
ENSCAFG00000014373	TXNRD2	1270.319965	745.1776977	1128.742763
ENSCAFG00000014713	REX1BD	390.6064062	244.3037994	181.0247828
ENSCAFG00000013872	TRAPPC6B	109.8226707	150.2621695	91.10397565
ENSCAFG00000032287	CCL22	0	0	0
ENSCAFG00000031469		193.6049144	380.2552861	301.7079713
ENSCAFG00000004115	KIAA1549	3.396577445	1.022191629	1.183168515
ENSCAFG00000010700	CCND1	56.60962409	62.35368939	151.4455699
ENSCAFG00000007068	RASGEF1A	5.660962409	0	2.36633703
ENSCAFG00000015762	AP1M1	292.1056603	269.8585901	314.722825
ENSCAFG00000020037	TBC1D13	31.70138949	56.22053961	48.50990911
ENSCAFG00000031314	CAPNS1	714.413456	618.4259357	550.1733595
ENSCAFG00000008351	TLR2	20.37946467	20.44383259	33.12871842
ENSCAFG00000020018	LRRC39	3.396577445	2.044383259	2.36633703
ENSCAFG00000001277	ANKS1A	88.31101358	68.48683916	112.4010089
ENSCAFG00000019788	SLC16A4	717.8100334	717.5785237	1000.960564
ENSCAFG00000002214	TRAM2	83.78224365	58.26492287	74.53961644

ENSCAFG00000012171	TIAL1	581.9469356	752.3330391	532.4258317
ENSCAFG00000017492	ARHGAP17	153.9781775	110.396696	191.6732994
ENSCAFG00000019845	SLC22A31	0	0	0
ENSCAFG00000009334	OLFML3	449.4804153	127.7739537	210.6039957
ENSCAFG00000003165	BPGM	97.36855343	225.9043501	124.2326941
ENSCAFG00000017172	CHRNB2	0	0	0
ENSCAFG00000009416	TSPAN18	10.18973234	3.066574888	8.282179605
ENSCAFG00000003399	TES	298.8988152	120.6186123	110.0346719
ENSCAFG00000004369	NOX4	0	0	0
ENSCAFG00000003483	KCNF1	0	0	0
ENSCAFG00000006859	TMEM132B	116.7290449	58.38758586	30.76238139
ENSCAFG00000011925	HSD11B1	5616.806902	13896.6952	8548.392521
ENSCAFG00000012983	ADGRG2	2.264384964	1.022191629	0
ENSCAFG00000004587	CLASRP	192.4727219	88.93067175	95.83664971
ENSCAFG00000014992	FMO3	3774.729734	7571.373398	8353.169716
ENSCAFG00000030286		8227.744662	564.0044534	1001.895267
ENSCAFG00000016401	TMEM229B	10.18973234	8.177533034	18.93069624
ENSCAFG00000016239	MED24	175.4898347	168.6616188	216.5198382
ENSCAFG00000019060	ABR	122.276788	65.42026427	107.6683349
ENSCAFG00000028459	MT3	4.528769927	0	0
ENSCAFG00000001532	ZDHHC21	38.49454438	76.6643722	44.96040357
ENSCAFG00000001600	CPSF1	547.0074756	203.9987835	497.4277071
ENSCAFG00000015651	SLC35E1	27.17261956	72.57560568	92.28714417
ENSCAFG00000038716		2.264384964	9.199724663	9.46534812
ENSCAFG00000017232	HACD3	1312.211086	1969.76327	1598.460664
ENSCAFG00000010801	SLC9B2	0	6.133149776	2.36633703
ENSCAFG00000040988	TREX1	6.793154891	3.066574888	7.09901109
ENSCAFG00000012180	DVL3	225.3063039	162.5284691	375.0644192
ENSCAFG00000019241	DUSP9	0	0	1.183168515
ENSCAFG00000030293		4.528769927	1.533287444	1.183168515
ENSCAFG00000032444		4.528769927	1.533287444	1.183168515
ENSCAFG00000016211	MICAL3	101.8973234	93.01943826	143.1633903
ENSCAFG00000013792	STARD5	1591.862629	1175.520374	1844.559715
ENSCAFG00000049209		310.22074	185.0166849	76.90595347
ENSCAFG00000009074	HOMER1	331.7323972	535.6284137	564.3713816
ENSCAFG00000003775	OSR1	0	0	0
ENSCAFG00000017035	CIAO2A	1178.612374	1940.119712	1566.515114

ENSCAFG00000041552		0	2.698585901	0
ENSCAFG00000033431		1.132192482	1.022191629	0
ENSCAFG00000029454		0	0	0
ENSCAFG00000044926		1.132192482	1.022191629	1.183168515
ENSCAFG00000000827	SMC1B	2.264384964	0	0
ENSCAFG00000031200		2.264384964	0	0
ENSCAFG00000021151	SNORA63	0	1.022191629	2.36633703
ENSCAFG00000042025		0	2.044383259	1.183168515
ENSCAFG00000047193		2.264384964	0	1.183168515
ENSCAFG00000049943		0	2.044383259	0
ENSCAFG00000045335		1.132192482	1.022191629	0
ENSCAFG00000044734	KRT28	1.132192482	1.022191629	0
ENSCAFG00000018146	ALDH3A1	1.132192482	0	0
ENSCAFG00000000614	LY6G6C	2.264384964	0	1.183168515
ENSCAFG00000004639	SUSD5	2.264384964	0	0
ENSCAFG00000044728		2.264384964	0	0
ENSCAFG00000041021		2.264384964	0	1.183168515
ENSCAFG00000042461		175.4898347	99.15258804	171.5594347
ENSCAFG00000000072		15.85069474	2.044383259	5.915842575
ENSCAFG00000012475	PEA15	125.6733655	60.30930613	91.10397565
ENSCAFG00000009009	DECR1	1265.791195	1015.036288	1113.361573
ENSCAFG00000000870	RTL6	9.057539854	3.066574888	8.282179605
ENSCAFG00000001945	CYP51A1	2902.941523	1256.273512	1319.232894
ENSCAFG00000006691	SLC46A3	314.7495099	563.2275877	453.1535412
ENSCAFG00000014103	SESTD1	262.6686558	392.5215856	350.2178804
ENSCAFG00000018240	SLC26A2	5.944010529	8.525078188	6.164307963
ENSCAFG00000028865		3.396577445	2.044383259	9.46534812
ENSCAFG00000043796		0	2.667920152	0
ENSCAFG00000013414	ADAM23	396.2673686	693.0459246	410.5594747
ENSCAFG00000048604		0	0	0
ENSCAFG00000046211		1.132192482	3.066574888	4.73267406
ENSCAFG00000046836		0	0	0
ENSCAFG00000030114	RFXAP	18.11507971	27.59917399	31.9455499
ENSCAFG00000014054	ACHE	2.264384964	0	1.183168515
ENSCAFG00000000791	NOTCH4	11.32192482	9.199724663	14.19802218
ENSCAFG00000006553	TMF1	317.0138949	493.7185569	234.267366
ENSCAFG00000006379	N4BP2L1	118.8802106	219.7712003	124.2326941

ENSCAFG00000006103	NEK11	4.528769927	3.066574888	4.73267406
ENSCAFG00000018216	RBBP8	781.2128124	390.4772024	288.6931177
ENSCAFG00000018176	TAF15	390.6064062	353.6783037	598.6832686
ENSCAFG00000015689	OTUD5	263.8008483	201.371751	336.0198583
ENSCAFG00000033348		0	0	0
ENSCAFG00000015113	QDPR	4691.069719	5858.221115	4823.482243
ENSCAFG00000044714	RAD21L1	9.057539854	2.044383259	5.915842575
ENSCAFG00000025213	FAM111B	56.60962409	150.2621695	131.3317052
ENSCAFG00000011513	ABLIM1	62.2705865	91.99724663	166.8267606
ENSCAFG00000018700	XPNPEP2	13.58630978	10.22191629	37.86139248
ENSCAFG00000017329	FLRT2	4.528769927	16.35506607	3.549505545
ENSCAFG00000014100	SGPL1	123.4089805	152.3065528	404.6436321
ENSCAFG00000018091	SYNPO	56.60962409	17.3772577	123.0495256
ENSCAFG00000010258	SIRT4	38.49454438	54.17615635	59.15842575
ENSCAFG00000015177	SELL	181.1507971	263.7254404	76.90595347
ENSCAFG00000032883		3.396577445	0	0
ENSCAFG00000001376	SLC26A8	27.17261956	15.33287444	16.56435921
ENSCAFG00000029819	CELSR3	0	0	0
ENSCAFG00000009087	ZDHHC16	92.83978351	109.3745043	133.6980422
ENSCAFG00000018101	CD74	3097.67863	2559.56784	4209.713576
ENSCAFG00000018612	CARD6	170.9610647	245.325991	256.7475677
ENSCAFG00000002424	BZW2	214.1768518	172.2086238	195.5422605
ENSCAFG00000025195	ATN1	378.1522889	99.15258804	295.7921287
ENSCAFG00000000248	CLK4	215.1165715	294.3911892	93.47031268
ENSCAFG00000005205	BABAM2	305.6919701	388.4328191	372.6980822
ENSCAFG00000005052	CYP2B6	28694.28626	7485.509301	9511.491692
ENSCAFG00000018634	TUBB4A	37.53218077	2.167046254	11.85534852
ENSCAFG00000000621	CLIC1	685.4519723	376.1051881	329.3349561
ENSCAFG00000047702		0	0	0
ENSCAFG00000026738	SNORA50C	0	0	3.549505545
ENSCAFG00000009321	RIPK1	170.9610647	342.4341958	376.2475878
ENSCAFG00000028892	IMPACT	195.8692993	472.2525327	366.7822396
ENSCAFG00000002873		439.2906829	577.5382705	598.6832686
ENSCAFG00000002931	DCK	19.24727219	17.3772577	15.38119069
ENSCAFG00000012444	ALG3	288.7090829	129.8183369	317.089162
ENSCAFG00000009207	AP3B1	521.9407341	765.6215303	648.3763462
ENSCAFG00000018930	GIT1	74.7247038	47.02081495	68.62377387

ENSCAFG00000005549	C2CD3	46.41989175	49.0651982	69.80694238
ENSCAFG00000002053	RFX3	6.793154891	10.22191629	7.09901109
ENSCAFG00000012243	NEFH	3.396577445	0	0
ENSCAFG00000011587	NGEF	230.9672663	280.0805064	431.856508
ENSCAFG00000019078	NXN	13.58630978	12.26629955	3.549505545
ENSCAFG00000005312	PGS1	64.53497146	33.73232377	47.3267406
ENSCAFG00000017986	DYNC1H1	1857.927863	932.2387659	2559.193498
ENSCAFG00000018092	TMC5	0	1.022191629	0
ENSCAFG00000040193		0	1.022191629	0
ENSCAFG00000007976	TEAD1	89.44320606	65.42026427	121.866357
ENSCAFG00000015776	CCDC120	13.58630978	6.133149776	16.56435921
ENSCAFG00000007770	LNPEP	2.264384964	5.110958146	4.73267406
ENSCAFG00000019669	ELANE	3.396577445	0	0
ENSCAFG00000024019		0	0	0
ENSCAFG00000033145		0	0	0
ENSCAFG00000010470	GCA	27.17261956	96.08601315	23.6633703
ENSCAFG00000016241	SMC1A	209.4556091	213.6380505	239.00004
ENSCAFG00000000065	GDF11	9.057539854	4.088766517	7.09901109
ENSCAFG00000017139	CLN3	182.2829896	174.7947686	195.222805
ENSCAFG00000004904	CAND2	9.057539854	8.177533034	8.282179605
ENSCAFG00000012255	RAB17	1936.049144	1800.079459	1251.792289
ENSCAFG00000011241	SLCO3A1	9.057539854	5.110958146	9.46534812
ENSCAFG00000010758	MAP2K6	4.528769927	7.155341405	3.549505545
ENSCAFG00000003862		35.96975515	13.03294327	3.821634303
ENSCAFG00000014876	BCS1L	157.374755	175.8169602	138.4307163
ENSCAFG00000019340	PAQR4	7.925347372	5.110958146	5.915842575
ENSCAFG00000013233	PISD	76.98908876	112.4410792	131.3317052
ENSCAFG00000031944	GYPA	0	0	0
ENSCAFG00000007427	HSP90B1	8425.776449	6713.754621	7475.258678
ENSCAFG00000011795	SLAMF8	0	0	0
ENSCAFG00000015235	SLC19A2	249.082346	1511.82142	1151.222965
ENSCAFG00000030234	C35H6orf52	1.132192482	3.066574888	0
ENSCAFG00000000297	AVIL	4.528769927	1.022191629	0
ENSCAFG00000018629	WSB1	838.954629	574.4716956	282.7772751
ENSCAFG00000010888	UPK1B	2.264384964	11.24410792	56.79208872
ENSCAFG00000019005	BLMH	170.9610647	266.7920152	246.0990511
ENSCAFG00000000669	EHMT2	257.0076934	159.4618942	302.8911398

ENSCAFG00000015338	CYP19A1	0	0	0
ENSCAFG00000030509		9.702889569	11.80631332	5.596387076
ENSCAFG00000010085	ATP10A	84.91443613	65.42026427	29.57921287
ENSCAFG00000047349		0	0	0
ENSCAFG00000043339		15.85069474	9.199724663	10.64851663
ENSCAFG00000008385	CCDC25	141.1617586	169.1624927	162.7684926
ENSCAFG00000049853		0	2.044383259	2.36633703
ENSCAFG00000004972	OXSR1	121.1445956	197.2829844	176.2921087
ENSCAFG00000013412	MFAP1	170.9610647	182.9723016	162.0940866
ENSCAFG00000003729	SMC6	701.9593387	574.4716956	488.6485967
ENSCAFG00000011274	SV2B	0	1.022191629	0
ENSCAFG00000004109	FAM120B	151.7137926	156.3953193	261.4802418
ENSCAFG00000042623	GTF2A1L	0	0	0
ENSCAFG00000014053	RASGRF1	0	0	0
ENSCAFG00000044875		0	0	0
ENSCAFG00000042016		0	0	0
ENSCAFG00000017043	ZMYM3	53.21304664	31.68794051	81.63862753
ENSCAFG00000013063	TBCD	506.0900394	295.4133809	494.5644393
ENSCAFG00000019233	SRL	3.396577445	11.24410792	8.282179605
ENSCAFG00000019824	DPEP1	7.925347372	1.022191629	2.36633703
ENSCAFG00000017413	STX8	457.4057626	325.0569381	357.3168915
ENSCAFG00000030651	SPRY3	30.56919701	3.066574888	10.64851663
ENSCAFG00000005807	NUMA1	848.0121689	723.7116735	1357.094287
ENSCAFG00000001492	BTBD9	271.7261956	296.4355725	359.6832286
ENSCAFG00000004599	SLC9A3R1	341.9221295	332.2122795	438.9555191
ENSCAFG00000045032		0	0	0
ENSCAFG00000010446	ACADS	1255.601462	1202.097356	2011.386475
ENSCAFG00000019450	NELFB	151.7137926	203.4161342	199.955479
ENSCAFG00000017596	TMEM220	259.2720783	485.5410239	751.312007
ENSCAFG00000013917	HK1	98.50074591	54.17615635	86.37130159
ENSCAFG00000014601	TAF6	212.8521866	179.9057268	164.4604236
ENSCAFG00000015293	YDJC	3.396577445	0	0
ENSCAFG00000042902	SHISA5	221.9097264	195.2386012	178.6584458
ENSCAFG00000019687	FAM234A	133.5987129	58.26492287	171.5594347
ENSCAFG00000008401	P2RX7	3.396577445	4.088766517	4.73267406
ENSCAFG00000020159	GLMN	105.2939008	159.4618942	138.4307163
ENSCAFG00000011757	RCC1	72.46031883	86.88628849	66.25743684

ENSCAFG00000019901	SLC7A5	13.58630978	0	7.09901109
ENSCAFG00000003000	PTGR1	1479.775574	1463.778413	986.7625415
ENSCAFG00000015692	KAT2A	150.5816001	96.08601315	113.5841774
ENSCAFG00000011992	WDR6	131.3343279	86.88628849	162.0940866
ENSCAFG00000000688	HDAC10	125.6733655	108.3523127	114.767346
ENSCAFG00000014257	PPP2R2C	0	0	0
ENSCAFG00000011975	PIR	627.2346349	453.8530834	314.722825
ENSCAFG00000011868	PCK2	8131.406404	14424.14608	19237.13688
ENSCAFG00000001372	SCRIB	536.6592364	147.1955946	379.7970933
ENSCAFG00000008554	AADAC	407.5892934	1330.893501	585.6684149
ENSCAFG00000029621	ACER2	101.8973234	59.2871145	43.77723505
ENSCAFG00000014926	DTWD1	187.943952	161.5062774	191.6732994
ENSCAFG00000007541	MRAS	181.1507971	35.77670702	113.5841774
ENSCAFG00000001390	RMI1	151.7137926	104.2635462	54.42575169
ENSCAFG00000019846	CDH15	0	0	0
ENSCAFG00000005203	TSKU	8314.821586	582.6492287	1344.079433
ENSCAFG00000011756	PLXNA2	23.77604212	11.24410792	31.9455499
ENSCAFG00000011487	BCAT1	3.396577445	1.022191629	2.36633703
ENSCAFG00000003903	FAF1	544.5732618	436.4758257	470.8892373
ENSCAFG00000024796	RNH1	80.38566621	113.4632708	102.9356608
ENSCAFG00000005843	LTBP1	371.359134	152.3065528	165.6435921
ENSCAFG00000014660	AOC3	1145.744826	1056.445271	3652.902641
ENSCAFG00000017832	MYO19	84.91443613	107.3301211	156.178244
ENSCAFG00000000159	NEMP1	9.057539854	25.55479073	17.74752772
ENSCAFG00000000641		3530.968693	1576.004832	1889.271653
ENSCAFG00000009481	SEMA3G	13.58630978	8.177533034	10.64851663
ENSCAFG00000006994	ZNF664	420.0434107	369.0111782	246.0990511
ENSCAFG00000017340	TOM1L1	1033.691736	1154.054349	970.1981823
ENSCAFG00000017057	ARHGEF15	28.30481204	10.22191629	20.11386475
ENSCAFG00000044408	SGK2	315.8817024	223.8599668	143.1633903
ENSCAFG00000017318	EBF1	5.660962409	0	0
ENSCAFG00000025148	TMX2	365.6981716	347.545154	421.2079913
ENSCAFG00000045851		22.64384964	15.33287444	2.36633703
ENSCAFG00000013895		475.5208423	423.1873345	677.9555591
ENSCAFG00000009387	INTS8	170.9610647	145.1512114	144.3465588
ENSCAFG00000014076	CTSH	809.5176245	822.8642616	525.3268206
ENSCAFG00000002657	TTYH1	2.264384964	0	0

ENSCAFG00000024407	QRFP	0	0	1.183168515
ENSCAFG00000018704	SLC1A3	0	0	0
ENSCAFG00000046244		848.0121689	871.9294598	1095.614045
ENSCAFG00000032754	HKDC1	0	0	0
ENSCAFG00000011787	CRP	8568.432702	8530.189146	3334.168875
ENSCAFG00000029286	CD7	44.15550679	8.177533034	11.83168515
ENSCAFG00000012380	SLC37A4	2033.417697	1792.924118	2683.426192
ENSCAFG00000005767	PDE6G	0	0	0
ENSCAFG00000019382	SGTA	173.2254497	123.6851871	205.8713216
ENSCAFG00000018906	MYSM1	65.66716394	83.8197136	36.67822396
ENSCAFG00000014131	C18H7orf57	4.528769927	7.155341405	3.549505545
ENSCAFG00000013256	MRC2	41.89112183	28.62136562	39.04456099
ENSCAFG00000003752	KCNS3	27.17261956	17.3772577	16.56435921
ENSCAFG00000013909	SERPINE1	53.21304664	5.110958146	4.73267406
ENSCAFG00000014023	OTC	2948.229223	8351.305611	6619.827841
ENSCAFG00000030815	PCDHB6	11.74083604	0	4.436881931
ENSCAFG00000020060		72.46031883	28.62136562	30.76238139
ENSCAFG00000015902	P3H4	38.49454438	18.39944933	40.22772951
ENSCAFG00000001394	ETV7	32.83358197	62.35368939	35.49505545
ENSCAFG00000011047	IL3RA	2.264384964	1.022191629	1.183168515
ENSCAFG00000018202	MAPK7	23.77604212	37.82109028	49.69307763
ENSCAFG00000007432	ATP12A	0	0	0
ENSCAFG00000017651	DNM2	421.7869872	430.8333279	678.1685294
ENSCAFG00000012998	ACE	22.64384964	8.177533034	14.19802218
ENSCAFG00000005737	SFRP1	164.1679099	83.8197136	164.4604236
ENSCAFG00000046041		1.132192482	2.555479073	1.159505145
ENSCAFG00000047687		1.132192482	2.555479073	1.159505145
ENSCAFG00000016590	FBXO2	73.59251132	186.0388765	263.8465788
ENSCAFG00000004501	COG1	219.6453415	215.6824338	309.9901509
ENSCAFG00000023269	GAS1	75.85689628	134.9292951	154.9950755
ENSCAFG00000016245		0	22.48821584	16.56435921
ENSCAFG00000004146	ORC5	131.3343279	289.2802311	242.5495456
ENSCAFG00000019639	VBP1	168.6966798	318.9237883	159.7277495
ENSCAFG00000009360	ANO6	149.4494076	255.5479073	478.00008
ENSCAFG00000014220	C1R	35895.03044	27273.09486	52225.05825
ENSCAFG00000013418	NFKBIA	1667.719526	1643.68414	779.7080514
ENSCAFG00000007613	POLR1A	267.1974257	172.7503853	347.8515434

ENSCAFG00000001892	XPO5	233.2316512	259.6366738	356.133723
ENSCAFG00000041227		186.8117595	155.3731276	111.2178404
ENSCAFG00000017544	S100A14	4.528769927	0	9.46534812
ENSCAFG00000032290	RAPGEFL1	3.396577445	1.022191629	3.549505545
ENSCAFG00000007612	RBP2	62.2705865	42.93204843	98.20298674
ENSCAFG00000015365	FGF23	0	0	0
ENSCAFG00000018676		0	0	0
ENSCAFG00000016179	IQSEC2	3.396577445	2.044383259	4.73267406
ENSCAFG00000032142	PDE1B	2.264384964	1.022191629	9.46534812
ENSCAFG00000000400		22.41741114	24.5325991	29.57921287
ENSCAFG00000009061	SLC8B1	764.2299252	366.9667949	651.9258518
ENSCAFG00000003888	TTC39A	14.71850226	10.22191629	9.46534812
ENSCAFG00000013219	BNC1	0	1.022191629	23.6633703
ENSCAFG00000000277	CCDC28A	192.4727219	406.8322684	211.7871642
ENSCAFG00000013088	ING5	61.13839402	39.86547354	31.9455499
ENSCAFG00000049487	NRM	122.276788	87.90848012	144.3465588
ENSCAFG00000015041	DDOST	2868.975749	2955.156	2627.817272
ENSCAFG00000012374	FGFR2	396.2673686	659.3136009	495.7476078
ENSCAFG00000011788	ZFP64	69.06374139	98.13039641	93.47031268
ENSCAFG00000034058		29896.27841	52495.83686	56891.2619
ENSCAFG00000017670	UBFD1	181.1507971	225.9043501	209.4208272
ENSCAFG00000002697	LENG1	129.0699429	93.01943826	52.05941466
ENSCAFG00000011264	TBX10	0	4.088766517	0
ENSCAFG00000004665	AOC1	1.132192482	0	0
ENSCAFG00000002261	FAM120A	711.0168786	1086.589702	1490.792329
ENSCAFG00000007245	IL1A	7.925347372	25.55479073	1.183168515
ENSCAFG00000032573	CCL26	0	0	0
ENSCAFG00000009330	SV2C	4.528769927	25.55479073	0
ENSCAFG00000032723	H2AFV	301.1632002	343.4563874	295.7921287
ENSCAFG00000005838	PCCA	604.5907853	758.4661889	718.1832886
ENSCAFG00000001941	TMEM63B	785.7415824	459.9862332	666.1238739
ENSCAFG00000014255	SPAG16	6.793154891	1.022191629	8.282179605
ENSCAFG00000009661	PIK3C2B	39.62673686	99.15258804	106.4851663
ENSCAFG00000017845	DHRS11	10.18973234	4.088766517	3.549505545
ENSCAFG00000020049	SPTAN1	1089.169167	871.9294598	1837.460704
ENSCAFG00000006086	PCDHGC3	141.5240602	65.42026427	140.7970533
ENSCAFG00000011615	GOLGB1	658.9360244	741.0889312	1012.792249

ENSCAFG00000015761	SPAG7	759.7011553	691.0015414	725.2822997
ENSCAFG00000019380	CACNA1B	0	0	1.183168515
ENSCAFG00000017614	GEMIN5	174.3576422	187.0610682	227.1683549
ENSCAFG00000006864	MICU3	29.43700453	67.46464753	48.50990911
ENSCAFG00000001296	GRAP2	18.11507971	11.24410792	9.46534812
ENSCAFG00000002233	NPR2	36.23015942	18.39944933	18.93069624
ENSCAFG00000003890	KIAA1147	65.66716394	63.37588101	74.53961644
ENSCAFG00000007482	RILPL1	177.7542196	121.6408039	152.6287384
ENSCAFG00000016644	NPEPPS	101.8973234	356.7448786	379.7970933
ENSCAFG00000024725	TXK	23.77604212	2.044383259	8.282179605
ENSCAFG00000047307		4.528769927	0	0
ENSCAFG00000043695		3.396577445	0	0
ENSCAFG00000047414		0	0	0
ENSCAFG00000028954		0	0	0
ENSCAFG00000003304	ZC3H12A	75.85689628	23.51040747	17.74752772
ENSCAFG00000005521	PLEKHG2	29.43700453	5.110958146	20.11386475
ENSCAFG00000011703	ZGRF1	5.660962409	10.22191629	4.73267406
ENSCAFG00000033113		4.528769927	1.022191629	2.36633703
ENSCAFG00000014146	ZNF385B	9.057539854	14.31068281	15.38119069
ENSCAFG00000010473	SH3PXD2A	75.85689628	45.99862332	67.44060535
ENSCAFG00000043529		10.05386924	0	5.915842575
ENSCAFG00000011874	DOK5	0	0	0
ENSCAFG00000020139	ST3GAL2	50.94866168	62.35368939	82.82179605
ENSCAFG00000015765	TFE3	117.7480181	135.9514867	230.7178604
ENSCAFG00000043615		0	0	0
ENSCAFG00000001290	AKR1B1	131.0626017	71.25697848	79.2722905
ENSCAFG00000004023	ZEB1	318.1460874	278.0361232	235.4505345
ENSCAFG00000018254	GREB1L	30.56919701	42.93204843	76.90595347
ENSCAFG00000007950	MAP1B	12.4541173	10.22191629	1.183168515
ENSCAFG00000008918	EBF2	0	0	0
ENSCAFG00000008420	COPB1	1690.363375	3161.638709	2433.777635
ENSCAFG00000001316	DAPK1	158.5069474	141.0624448	208.2376586
ENSCAFG00000030514	TRAM1L1	12.4541173	9.199724663	17.74752772
ENSCAFG00000024350	CENPP	1454.867339	1433.112664	1680.099291
ENSCAFG00000010745	IGHMBP2	177.7655416	180.9279184	134.8812107
ENSCAFG00000024196	VSTM1	7.925347372	1.022191629	1.183168515
ENSCAFG00000011049	FAM207A	181.1507971	192.1720263	188.1237939

ENSCAFG00000003246	ALAD	2018.699195	2046.427642	1907.267646
ENSCAFG00000044598		0	0	0
ENSCAFG00000010874	SULF2	92.83978351	57.24273124	97.01981823
ENSCAFG00000006985	NCOR2	172.0932572	52.13177309	203.5049846
ENSCAFG00000046271		30.56919701	14.31068281	16.56435921
ENSCAFG00000025281	CD1A8	2.264384964	5.110958146	1.183168515
ENSCAFG00000007547	DYNLRB1	1076.71505	928.1499994	918.1387676
ENSCAFG00000019489	TP73	0	0	0
ENSCAFG00000019602	PLEKHG5	15.85069474	7.155341405	16.56435921
ENSCAFG00000017941	CYP1A2	1206.23787	3175.796063	494.3633006
ENSCAFG00000012976	NARF	75.85689628	113.4632708	121.866357
ENSCAFG00000018403		423.4399882	571.4051208	614.0644593
ENSCAFG00000003541	AP2A1	402.0528722	303.2433687	489.2283493
ENSCAFG00000044134		3.396577445	3.066574888	0
ENSCAFG00000010453	TARSL2	167.5644873	210.5714756	156.178244
ENSCAFG00000015752	RCC2	81.51785869	67.46464753	91.10397565
ENSCAFG00000015659	TTC9C	26.04042708	49.0651982	28.39604436
ENSCAFG00000046786		1.132192482	0	3.549505545
ENSCAFG00000031014	FAM180A	22.64384964	19.42164096	15.38119069
ENSCAFG00000001828	KCTD8	0	0	0
ENSCAFG00000018428	MTA1	340.789937	220.7933919	289.8762862
ENSCAFG00000028743	C7H18orf32	377.0200964	406.8322684	360.8663971
ENSCAFG00000001728		155.11037	115.5076541	159.7277495
ENSCAFG00000011851	LRFN4	1.132192482	1.022191629	0
ENSCAFG00000031148	GLYCTK	1803.582623	5625.048982	4339.862113
ENSCAFG00000010055	LZTS1	2.264384964	1.022191629	2.36633703
ENSCAFG00000002796	CCDC88A	105.2939008	134.9292951	117.133683
ENSCAFG00000000080	PMEL	6.793154891	5.110958146	18.93069624
ENSCAFG00000016285		386.0776363	606.1596362	405.8268006
ENSCAFG00000006815	TUSC3	115.4836331	33.73232377	85.18813308
ENSCAFG00000000959	POLDIP3	113.2192482	116.5298457	192.8564679
ENSCAFG00000011692		1.132192482	2.044383259	0
ENSCAFG00000016302	VWCE	197.0014918	129.8183369	351.4010489
ENSCAFG00000003990	KDM7A	163.0357174	212.6158589	151.4455699
ENSCAFG00000042338		0	0	0
ENSCAFG00000017659		1.132192482	0	0
ENSCAFG00000004991	PPARG	144.9206377	249.4147575	168.0099291

ENSCAFG00000017841	G3BP1	658.9360244	655.2248344	875.5447011
ENSCAFG00000006831	SLC15A4	350.9796694	426.2539094	399.9109581
ENSCAFG00000001380	NTRK2	0	0	0
ENSCAFG00000018709	FOXN1	0	0	0
ENSCAFG00000006777	ADAMTS9	10.18973234	19.42164096	10.64851663
ENSCAFG00000000380	MED23	140.3918677	354.7004954	237.8168715
ENSCAFG00000003465	EGFR	1288.435044	402.7435019	1154.772471
ENSCAFG00000017730	ZMAT1	37.3623519	52.13177309	41.41089802
ENSCAFG00000010161	LPL	14.71850226	10.22191629	1.183168515
ENSCAFG00000003235	NUP205	75.85689628	158.4397025	127.7821996
ENSCAFG00000044765		3.396577445	3.465229623	0
ENSCAFG00000019538	STK11	646.6177702	289.8219926	529.5152372
ENSCAFG00000000957	SNTB1	241.1569986	277.0139315	222.4356808
ENSCAFG00000023807		0	0	2.36633703
ENSCAFG00000009637	LGI3	69.06374139	30.66574888	43.77723505
ENSCAFG00000049417		31.70138949	42.93204843	5.915842575
ENSCAFG00000015298	CD38	47.55208423	81.77533034	37.86139248
ENSCAFG00000013220	ELOA	349.8474769	232.0374998	229.5346919
ENSCAFG00000016467		9.057539854	65.42026427	27.21287584
ENSCAFG00000029641	ERICH2	3.396577445	0	0
ENSCAFG00000010670	ZNF496	33.96577445	24.5325991	48.50990911
ENSCAFG00000019161	GABRA3	1.132192482	2.044383259	0
ENSCAFG00000045424		651.010677	242.2594161	440.1386876
ENSCAFG00000034297		0	2.044383259	0
ENSCAFG00000023478		2.16248764	0	2.460990511
ENSCAFG00000015008	GORAB	69.06374139	134.9292951	91.10397565
ENSCAFG00000002154	TLN1	919.3402952	514.1623895	1150.039797
ENSCAFG00000017015	CTC1	138.387887	72.57560568	145.316757
ENSCAFG00000018036	HSP90AA1	3501.610942	4039.905757	3515.832569
ENSCAFG00000009417	CDYL	63.40277898	68.48683916	76.90595347
ENSCAFG00000017501	SLC5A11	0	0	0
ENSCAFG00000040735		0	0	0
ENSCAFG00000002714	EPHA5	1.132192482	53.15396472	7.09901109
ENSCAFG00000032608	LURAP1L	711.0168786	413.9876099	306.4406454
ENSCAFG00000009443	IRX5	0	0	0
ENSCAFG00000005984	POGLUT2	134.8667684	170.1540186	86.98654922
ENSCAFG00000048160		5.26469504	1.032413546	3.549505545

ENSCAFG00000004840	INHBB	2.264384964	1.022191629	0
ENSCAFG00000004151	ANXA5	860.4662862	788.1097462	567.9208872
ENSCAFG00000019219	NADK	1904.347754	3151.416793	2962.653961
ENSCAFG00000018410		0	0	0
ENSCAFG00000000032	CBLN2	0	0	0
ENSCAFG00000019827	AMIGO1	26.04042708	51.10958146	127.7821996
ENSCAFG00000010343	KIRREL3	10.18973234	0	3.549505545
ENSCAFG00000003306	SLC13A4	0	0	0
ENSCAFG00000025256	FOCAD	146.0528301	125.7295704	269.7624214
ENSCAFG00000023992	ADGRE3	53.21304664	60.30930613	43.77723505
ENSCAFG00000043507		156.2425625	45.99862332	36.67822396
ENSCAFG00000004848	KCTD14	31.70138949	15.33287444	9.46534812
ENSCAFG00000011830	PITPNB	504.9578469	380.2552861	430.6733395
ENSCAFG00000014488	BAD	44.15550679	39.86547354	60.34159426
ENSCAFG00000019122	NAGPA	169.8288723	145.1512114	212.9703327
ENSCAFG00000005401	GPD1L	13.58630978	8.177533034	13.01485366
ENSCAFG00000008263	INTS9	50.94866168	103.2413546	84.00496456
ENSCAFG00000047196	NR2F1	386.0776363	178.8835351	333.6535212
ENSCAFG00000004890	VILL	18.40944975	4.252317178	9.46534812
ENSCAFG00000014861	ZNF142	72.46031883	21.46602421	81.63862753
ENSCAFG00000023355		336.6008248	38.65928742	22.59851864
ENSCAFG00000012317	TMEM237	159.6391399	267.8142069	197.589142
ENSCAFG00000016959	PKLR	7858.548016	7245.294268	4467.644313
ENSCAFG00000012674	ACADSB	4355.544477	6025.819655	7991.12015
ENSCAFG00000011705	CRTAM	19.24727219	0	3.549505545
ENSCAFG00000006060	TMTC3	32.83358197	119.5964206	48.50990911
ENSCAFG00000007457	GPATCH1	206.0590317	174.7947686	74.53961644
ENSCAFG00000011842	SESN2	127.9377504	390.4772024	622.3466389
ENSCAFG00000032391	FAM83H	321.5426648	171.7281937	468.5347319
ENSCAFG00000005895	STIM1	253.6111159	202.3939426	307.6238139
ENSCAFG00000018309	USP14	378.1522889	404.7878852	414.1089802
ENSCAFG00000001495	TMEM209	90.57539854	183.9944933	162.0940866
ENSCAFG00000018401	JAG2	12.4541173	2.044383259	4.73267406
ENSCAFG00000030305		1.132192482	8.177533034	8.282179605
ENSCAFG00000043476		3.396577445	2.044383259	2.36633703
ENSCAFG00000048864		0	0	0
ENSCAFG00000023358	SLC22A3	319.2782799	568.3385459	514.678304

ENSCAFG00000002852	ETFB	3259.582155	3101.329403	3306.955999
ENSCAFG00000017983	PPCDC	5.660962409	16.35506607	17.74752772
ENSCAFG00000001133	CCN4	0	0	0
ENSCAFG00000023027	ARRDC5	3.396577445	0	1.183168515
ENSCAFG00000016519	PPARGC1A	244.5535761	171.7281937	126.5990311
ENSCAFG00000012919	CDC25A	10.18973234	5.110958146	15.38119069
ENSCAFG00000016544	PACRGL	37.3623519	51.10958146	24.84653881
ENSCAFG00000012867	PPP1R7	351.3193271	237.148458	335.9370365
ENSCAFG00000017975	FAM219B	208.3234166	128.7961453	207.0544901
ENSCAFG00000037037		3.396577445	1.022191629	11.83168515
ENSCAFG00000002445	NCBP1	225.3063039	212.6158589	295.7921287
ENSCAFG00000030851	HLF	409.8536784	435.4536341	687.4209072
ENSCAFG00000006444	TRIM3	40.75892934	33.73232377	52.05941466
ENSCAFG00000006811	SDCBP2	1.132192482	0	1.183168515
ENSCAFG00000046645		0	0	0
ENSCAFG00000001120	L3MBTL2	164.1679099	118.574229	157.3614125
ENSCAFG00000001962	PHF24	0	0	0
ENSCAFG00000044353	C8orf88	5.660962409	14.31068281	18.93069624
ENSCAFG00000008709	CCDC102A	227.5706888	133.9071034	217.7030068
ENSCAFG00000007934	CTSB	2248.534269	2845.781496	2752.049966
ENSCAFG00000049203		0	0	0
ENSCAFG00000010136	MAGEL2	0	0	0
ENSCAFG00000003597	SCAF1	335.1289746	135.9514867	318.2723305
ENSCAFG00000017036	LRRC59	378.1522889	349.5895372	403.4604636
ENSCAFG00000014736	CLTCL1	21.51165715	29.64355725	27.21287584
ENSCAFG00000018119	TCOF1	112.0870557	50.08738983	68.62377387
ENSCAFG00000014965	GCH1	564.9640484	170.7060021	197.589142
ENSCAFG00000030867	HPCAL4	0	2.044383259	1.183168515
ENSCAFG00000016101	GPRIN2	0	0	0
ENSCAFG00000000549	MTRF1L	78.12128124	58.26492287	46.14357208
ENSCAFG00000006266	ARHGAP26	69.06374139	42.93204843	54.42575169
ENSCAFG00000030765	IKZF5	230.9672663	185.0166849	131.3317052
ENSCAFG00000019495	SPSB3	489.1071521	355.722687	359.6832286
ENSCAFG00000009258	CYTIP	86.04662862	7.155341405	10.64851663
ENSCAFG00000005940	THSD1	4.528769927	18.39944933	7.09901109
ENSCAFG00000030019	TRIAP1	112.0870557	156.3953193	152.6287384
ENSCAFG00000012523	CLDN4	26.04042708	29.64355725	53.24258317

ENSCAFG00000036085		0	0	0
ENSCAFG00000029379	UBE2S	168.6966798	68.48683916	46.14357208
ENSCAFG00000020053	PALMD	1406.183062	387.4106275	356.133723
ENSCAFG00000017078	NDEL1	132.4665204	97.10820478	72.17327941
ENSCAFG00000000710	SLC27A6	591.0044755	789.1319378	1150.039797
ENSCAFG00000008325	TM4SF18	6.793154891	18.39944933	16.56435921
ENSCAFG00000000679	SKIV2L	298.8988152	182.9723016	383.3465989
ENSCAFG00000016110	CTDNEP1	341.9221295	255.5479073	324.1881731
ENSCAFG00000011099	AMZ2	500.4290769	543.8059468	531.2426632
ENSCAFG00000015435	PORCN	1806.956557	1071.246606	907.490251
ENSCAFG00000001851	POLR3B	69.06374139	108.3523127	117.133683
ENSCAFG00000016934	NGFR	1467.321456	472.2525327	765.5100292
ENSCAFG00000002067	SLC1A1	2127.389673	1858.344382	1575.980462
ENSCAFG00000003798	CEP57L1	5.660962409	11.24410792	11.83168515
ENSCAFG00000007692	VAMP8	2040.210852	2143.535847	1308.584378
ENSCAFG00000012872	CD28	14.71850226	66.4424559	7.09901109
ENSCAFG00000005910	METTL25	48.68427672	37.82109028	30.76238139
ENSCAFG00000029050	UCHL3	151.7137926	206.4827091	126.5990311
ENSCAFG00000013602	PBLD	1840.944975	2979.688599	2611.252913
ENSCAFG00000006310	ARHGEF33	5.660962409	17.3772577	17.74752772
ENSCAFG00000015282	GGT6	28.30481204	48.04300658	29.57921287
ENSCAFG00000000149	STARD6	46.41989175	68.48683916	49.69307763
ENSCAFG00000009043	RRP12	268.3296182	154.350936	302.8911398
ENSCAFG00000005155	MFSD11	274.8963346	309.7547294	323.4901037
ENSCAFG00000016987	ITGA3	12.4541173	22.48821584	23.6633703
ENSCAFG00000000303	AIG1	1491.097499	1769.41371	1425.718061
ENSCAFG00000011994	KREMEN1	38.49454438	41.9098568	102.9356608
ENSCAFG00000016564		3.487152844	2.995021474	4.496040357
ENSCAFG00000029214	NPB	0	0	0
ENSCAFG00000015198	PELI2	12.4541173	22.48821584	10.64851663
ENSCAFG00000007504	AHCY	10444.50961	22847.50598	18521.85236
ENSCAFG00000012089		88.31101358	50.08738983	87.55447011
ENSCAFG00000005291	WNT11	5.660962409	1.022191629	0
ENSCAFG00000009647	REEP4	157.374755	262.7032487	181.0247828
ENSCAFG00000017343	KCNK10	0	0	0
ENSCAFG00000032084	SMIM19	380.4166739	678.7352418	517.044641
ENSCAFG00000000630	VWA7	4.528769927	0	2.36633703

ENSCAFG00000037735		6006.790602	6699.127059	3924.605459
ENSCAFG00000008956	SPR	1066.525318	1054.901761	900.3912399
ENSCAFG00000015767	GANAB	922.3292834	771.7137924	1379.113053
ENSCAFG00000012196		647.5008803	87.78581712	136.2418545
ENSCAFG00000019809	GSTM3	765.3621177	1219.474614	1588.995316
ENSCAFG00000009398	MSTN	0	2.044383259	0
ENSCAFG00000010263	PLA2G1B	0	2.044383259	0
ENSCAFG00000030460		0	2.044383259	0
ENSCAFG00000046883		0	2.044383259	0
ENSCAFG00000048107		0	2.044383259	0
ENSCAFG00000029278	SOX18	12.4541173	10.22191629	9.46534812
ENSCAFG00000016031	HSPA2	114.3514407	132.8849118	130.1485366
ENSCAFG00000015326	SLC38A5	0	0	0
ENSCAFG00000030756	PRSS54	0	2.044383259	1.183168515
ENSCAFG00000014943	CYP27A1	5156.004562	7823.85473	9595.496656
ENSCAFG00000006224	ZNF703	11.32192482	1.022191629	8.282179605
ENSCAFG00000012811	PPOX	142.6562527	128.7961453	114.767346
ENSCAFG00000010642	CSTB	261.5364633	97.10820478	208.2376586
ENSCAFG00000011806	PRR29	0	1.022191629	1.183168515
ENSCAFG00000013136	SMARCC1	276.2549656	292.346806	388.0792729
ENSCAFG00000034164		1.132192482	3.066574888	1.183168515
ENSCAFG00000023682	PCDHGA1	0	0	0
ENSCAFG00000009409	ECI2	5141.28606	5250.9984	3519.926332
ENSCAFG00000009684	DBNDD2	60.00620153	30.66574888	52.05941466
ENSCAFG00000028862	XIRP1	0	0	0
ENSCAFG00000019207	HMOX2	3118.058095	991.5258804	870.812027
ENSCAFG00000011922	KPNA1	201.5302618	300.524339	416.4753173
ENSCAFG00000004617	APOE	301647.7785	317590.8504	288232.8651
ENSCAFG00000008834	ZFYVE16	300.0310077	652.1582595	380.9802618
ENSCAFG00000008164	TMEM154	27.17261956	33.73232377	31.9455499
ENSCAFG00000031000	ZNF354A	148.8267017	212.840741	153.8000753
ENSCAFG00000020263	PTGS1	115.4836331	113.4632708	186.9406254
ENSCAFG00000019365		54.34523913	36.79889865	57.97525723
ENSCAFG00000017108		181.9999414	98.53927306	187.6741898
ENSCAFG00000015914	PELP1	70.19593387	36.79889865	106.4851663
ENSCAFG00000008221		1608.845517	1191.87544	1552.317092
ENSCAFG00000007178	KIF20B	97.36855343	143.1068281	136.0643792

ENSCAFG00000018779	PROCA1	4.528769927	8.177533034	4.73267406
ENSCAFG00000019575	EGFL7	252.4789234	394.5659689	194.0396365
ENSCAFG00000018621	CACHD1	28.30481204	9.199724663	16.56435921
ENSCAFG00000019556	RABL6	339.6577445	207.5049007	262.6634103
ENSCAFG00000010990		0	0	0
ENSCAFG00000011323		0	0	0
ENSCAFG00000032763	GDF3	0	0	0
ENSCAFG00000041507		0	0	0
ENSCAFG00000043553		0	0	0
ENSCAFG00000044284		0	0	0
ENSCAFG00000044490		0	0	0
ENSCAFG00000008211	CACNA2D3	2.264384964	0	0
ENSCAFG00000005671	LRRC3B	0	0	0
ENSCAFG00000001143	MCHR1	5.660962409	1.022191629	4.73267406
ENSCAFG00000045348		14.71850226	6.133149776	2.36633703
ENSCAFG00000013046	CEP19	5.660962409	28.72358478	13.81940825
ENSCAFG00000018588	L3MBTL4	61.13839402	110.396696	149.0792329
ENSCAFG00000018325	KIF26A	3.396577445	1.022191629	2.36633703
ENSCAFG00000004824	MEGF8	18.11507971	3.066574888	9.46534812
ENSCAFG00000015553	DKK1	3.396577445	4.088766517	4.73267406
ENSCAFG00000001254	EGR1	2787.45789	1506.710462	216.5198382
ENSCAFG00000015485	RBM3	180.0186046	71.55341405	179.8416143
ENSCAFG00000018154	PAK3	4.528769927	3.066574888	1.183168515
ENSCAFG00000012307	NF2	152.845985	215.6824338	231.9010289
ENSCAFG00000012113	VPS33B	212.8521866	180.9279184	177.4752772
ENSCAFG00000011576	ADNP	232.0994588	261.6810571	415.2921488
ENSCAFG00000001917	CNGA1	1.132192482	7.155341405	5.915842575
ENSCAFG00000009817	KCNJ15	6.793154891	3.066574888	0
ENSCAFG00000010461	TM4SF20	0	1.022191629	0
ENSCAFG00000016981	YLPM1	88.31101358	77.68656382	163.2772551
ENSCAFG00000007902	SLC11A2	64.53497146	88.93067175	80.45545902
ENSCAFG00000020422	ANKRD13C	206.0590317	239.1928412	255.5643992
ENSCAFG00000020287	SYDE2	106.4260933	381.2774777	305.2574769
ENSCAFG00000008398	DPH6	140.3918677	165.5950439	168.0099291
ENSCAFG00000000583	CLDN20	2.264384964	0	0
ENSCAFG00000002589	COL9A1	2.264384964	0	0
ENSCAFG00000005084	CNPY1	2.264384964	0	0

ENSCAFG00000025297		2.264384964	0	0
ENSCAFG00000033392		2.264384964	0	0
ENSCAFG00000037987		2.264384964	0	0
ENSCAFG00000038271		2.264384964	0	0
ENSCAFG00000039144		2.264384964	0	0
ENSCAFG00000039806		2.264384964	0	0
ENSCAFG00000041441		2.060590317	0	0
ENSCAFG00000043998		2.264384964	0	0
ENSCAFG00000045434		2.264384964	0	0
ENSCAFG00000045543		2.626686558	0	0
ENSCAFG00000045797		2.264384964	0	0
ENSCAFG00000047387		2.264384964	0	0
ENSCAFG00000047899		2.264384964	0	0
ENSCAFG00000048127		2.264384964	0	0
ENSCAFG00000048480		2.264384964	0	0
ENSCAFG00000017952	ACSM3	1056.335586	2367.395813	1228.128919
ENSCAFG00000020423	SRSF11	355.5084393	335.2788544	283.9604436
ENSCAFG00000007870	DCLK2	41.89112183	36.79889865	44.96040357
ENSCAFG00000018005	MAN2C1	187.943952	85.86409686	133.6980422
ENSCAFG00000007273	FAT1	623.8380575	271.9029734	380.9802618
ENSCAFG00000032080		131.3343279	125.7295704	201.1386475
ENSCAFG00000019798	CSF1	80.38566621	66.4424559	72.17327941
ENSCAFG00000023437	SEC14L4	7058.087931	16272.26855	10375.20471
ENSCAFG00000004029	TMEM143	38.49454438	48.04300658	65.07426832
ENSCAFG00000031330	HOPX	2553.094046	965.9710897	204.6881531
ENSCAFG00000040479		0	0	0
ENSCAFG00000006608	CPXM1	0	0	1.183168515
ENSCAFG00000029652		1.132192482	0	0
ENSCAFG00000004615	APOC1	107511.8659	83434.34736	77304.68126
ENSCAFG00000020052	FRRS1	39.62673686	291.3246143	150.2624014
ENSCAFG00000004833	B4GALT2	166.4322948	116.5298457	172.7426032
ENSCAFG00000013521	POLA1	36.23015942	65.42026427	61.52476278
ENSCAFG00000016780	STX1B	28.30481204	35.77670702	82.82179605
ENSCAFG00000005362	PLD3	515.1475792	587.7601868	902.7575769
ENSCAFG00000015628	SGMS1	945.3807223	337.3232377	399.9109581
ENSCAFG00000010756	CRCP	240.0248061	328.123513	330.1040157
ENSCAFG00000012633	PI4KB	169.8288723	247.3703743	222.4356808

ENSCAFG00000013204	PTPRE	33.96577445	26.57698236	21.29703327
ENSCAFG00000016869		644.2175221	1279.78392	896.8417343
ENSCAFG00000012093	CTSK	328.3358197	236.1262664	183.3911198
ENSCAFG00000019926	AIF1L	6.793154891	2.156824338	1.183168515
ENSCAFG00000002879	UBE3D	143.7884452	210.5714756	141.9802218
ENSCAFG00000011171	ALDH3B1	9.057539854	1.022191629	0
ENSCAFG00000017842	STOML1	93.97197599	117.5520374	105.3019978
ENSCAFG00000019006		0	0	2.36633703
ENSCAFG00000029850		0	0	2.36633703
ENSCAFG00000043031	TRBV24-1	0	0	2.36633703
ENSCAFG00000043978		0	0	1.987723105
ENSCAFG00000046473		0	0	2.36633703
ENSCAFG00000047338		0	0	2.36633703
ENSCAFG00000048413		0	0	2.36633703
ENSCAFG00000003075	AREG	0	0	0
ENSCAFG00000041233		0	0	3.549505545
ENSCAFG00000042097		0	2.064827091	0
ENSCAFG00000010625	LCK	39.62673686	18.39944933	29.57921287
ENSCAFG00000005096	ITIH5	141.5240602	52.13177309	74.53961644
ENSCAFG00000013182	EGLN3	304.5597776	318.9237883	375.0644192
ENSCAFG00000003082	AKIRIN2	212.3653438	110.2842549	129.6989326
ENSCAFG00000019077	YIPF1	347.5830919	620.470319	410.5594747
ENSCAFG00000045019		0	2.044383259	1.183168515
ENSCAFG00000041525		0	0	0
ENSCAFG00000011446	USP13	26.04042708	28.62136562	44.96040357
ENSCAFG00000045148		0	0.449764317	0
ENSCAFG00000000107	ALPK2	4.528769927	3.066574888	4.73267406
ENSCAFG00000023161	SDR9C7	12.4541173	10.22191629	10.64851663
ENSCAFG00000009928	XPO7	357.7728242	888.2845258	963.0991712
ENSCAFG00000029868		1.132192482	0	3.549505545
ENSCAFG00000008018		2.264384964	0	1.183168515
ENSCAFG00000019530	PAXX	712.149071	762.5549554	514.678304
ENSCAFG00000004532	DNAI2	0	0	2.36633703
ENSCAFG00000037737		3.396577445	1.022191629	1.183168515
ENSCAFG00000004901	STEAP3	117.7480181	351.6339205	609.3317852
ENSCAFG00000011146	ABHD4	407.5892934	485.5410239	405.8268006
ENSCAFG00000044566	FAHD1	1782.070966	1360.537059	1572.430956

ENSCAFG00000017958	COL5A3	6.793154891	2.044383259	0
ENSCAFG00000045929		0	0	0
ENSCAFG00000004555	RARRES2	9625.90048	6092.26211	10003.68979
ENSCAFG00000020252	NQO1	177.7542196	341.4120042	229.5346919
ENSCAFG00000013985	P3H2	132.4665204	146.173403	159.7277495
ENSCAFG00000005158	UBE3C	310.22074	434.4314424	494.5644393
ENSCAFG00000008992	NBN	928.3978351	1267.51762	1170.153661
ENSCAFG00000031614	IFIT3	14.71850226	0	2.36633703
ENSCAFG00000006842	GPAT2	5.660962409	7.155341405	2.36633703
ENSCAFG00000001019	UBE2B	423.4399882	447.7199336	246.0990511
ENSCAFG00000007304	IGF1	533.2626589	306.6574888	150.2624014
ENSCAFG00000009184	NT5DC2	166.4322948	86.88628849	100.5693238
ENSCAFG00000007247	ANKRD1	0	0	0
ENSCAFG00000014747	MFSD10	49.8164692	31.68794051	27.21287584
ENSCAFG00000017855	SPARC	1056.335586	261.6810571	306.4406454
ENSCAFG00000001264	TSNARE1	60.00620153	26.57698236	35.49505545
ENSCAFG00000032508	VGLL3	9.057539854	3.066574888	2.36633703
ENSCAFG00000007009	TMEM68	44.15550679	108.3523127	75.72278496
ENSCAFG00000019064	TMEM59	4994.101037	5081.314589	4471.193818
ENSCAFG00000009261	SLC38A2	619.3092875	794.2428959	487.4654282
ENSCAFG00000003028	HSDL2	5359.153859	6576.157406	6990.266072
ENSCAFG00000047530		215.1165715	70.53122242	126.5990311
ENSCAFG00000005305		10.18973234	6.133149776	3.549505545
ENSCAFG00000009284	F2R	55.47743161	50.08738983	125.4158626
ENSCAFG00000031403		35.09796694	12.26629955	8.282179605
ENSCAFG00000006051	EIF2AK2	57.74181657	111.4188876	70.9901109
ENSCAFG00000046415		0	0	0
ENSCAFG00000006541	ARL6IP5	240.0248061	296.4355725	224.8020178
ENSCAFG00000006049	PIK3R4	104.1617083	137.99587	136.0643792
ENSCAFG00000002191	DLX5	0	0	0
ENSCAFG00000028490		108.1130601	79.86383199	0
ENSCAFG00000008933	NLRC5	72.46031883	67.46464753	65.07426832
ENSCAFG00000029577	RGS12	62.2705865	52.13177309	74.53961644
ENSCAFG00000009165	RPUSD2	75.85689628	64.39807264	44.96040357
ENSCAFG00000015835	HNRNPU	1384.671405	934.2831492	1444.648757
ENSCAFG00000005005	TAMM41	153.9781775	170.7060021	127.7821996
ENSCAFG00000011713	DDX5	5088.073013	3045.108864	2388.817232

ENSCAFG00000005568	SAMD4B	97.36855343	51.10958146	108.8515034
ENSCAFG00000009612	IFIT2	16.98288723	0	8.282179605
ENSCAFG00000006680	LRIG1	47.55208423	129.8183369	100.5693238
ENSCAFG00000031160		37.3623519	220.7933919	136.0643792
ENSCAFG00000002448	AHR	2564.415971	2328.552531	2310.72811
ENSCAFG00000012970	MLXIPL	1676.777066	770.7324885	919.3219361
ENSCAFG00000006264	PCLO	18.11507971	3.066574888	15.38119069
ENSCAFG00000032342	PDGFD	170.9610647	49.0651982	28.39604436
ENSCAFG00000010031	VPS39	251.346731	307.6796804	347.8515434
ENSCAFG00000012994	GRHL3	4.528769927	2.044383259	1.183168515
ENSCAFG00000009233	GNAO1	1.132192482	0	1.183168515
ENSCAFG00000008875	TIPARP	1106.152055	352.6561121	160.910918
ENSCAFG00000020142	GARNL3	65.66716394	71.55341405	114.767346
ENSCAFG00000008205	AGTR1	1923.595027	2029.050384	1839.827041
ENSCAFG00000019532		0	0	0
ENSCAFG00000004251	GAREM2	1.132192482	0	3.549505545
ENSCAFG00000004836		1.132192482	0	0
ENSCAFG00000015499	DLG5	21.51165715	19.42164096	35.49505545
ENSCAFG00000013913	TRIM56	49.8164692	30.66574888	59.15842575
ENSCAFG00000011535	TMEM134	536.6592364	637.8475767	623.5298074
ENSCAFG00000017026	XYLT2	208.3234166	214.6602421	403.4604636
ENSCAFG00000002341	HMGCLL1	499.2968845	1264.451045	874.3615326
ENSCAFG00000024952	RNF130	1270.319965	1360.537059	2069.361733
ENSCAFG00000002568	GUCA2A	1.132192482	1.022191629	0
ENSCAFG00000009882	ZNF335	134.7309053	73.59779731	124.2326941
ENSCAFG00000031033	EMP1	110.9548632	34.7545154	16.56435921
ENSCAFG00000000194	INHBC	2730.848266	1129.52175	2355.688513
ENSCAFG00000042347		56.60962409	18.39944933	24.84653881
ENSCAFG00000030814	DOC2B	10.18973234	10.22191629	10.64851663
ENSCAFG00000009952	KCNIP2	3.396577445	1.022191629	1.183168515
ENSCAFG00000008745	MAPKAPK5	6344.806668	8001.716074	6925.085318
ENSCAFG00000005466	KYNU	238.8926137	1583.374834	1418.619049
ENSCAFG00000001286	FANCC	5.660962409	2.044383259	2.36633703
ENSCAFG00000001916	PIP5K1B	3.396577445	1.022191629	1.183168515
ENSCAFG00000030569	SDF2	116.6158256	177.8613435	121.866357
ENSCAFG00000019405	PLCH2	0	0	0
ENSCAFG00000048920		0	0	0

ENSCAFG00000002598	ALDOB	115593.4558	189410.0645	303366.7736
ENSCAFG00000032423	SCP2	17698.43288	26982.79244	24435.97934
ENSCAFG00000018824	DHRS13	217.3809565	393.5437773	338.3861953
ENSCAFG00000006745	PRAG1	6.793154891	16.35506607	17.74752772
ENSCAFG00000001154	MRTFA	91.70759102	54.17615635	127.7821996
ENSCAFG00000008481	VPS29	969.1567644	1113.166684	730.0149737
ENSCAFG00000029755	PYURF	1201.256223	1799.057268	1211.564559
ENSCAFG00000032267	WNT2	2.264384964	5.110958146	15.38119069
ENSCAFG00000001297	RHPN1	48.68427672	62.35368939	81.63862753
ENSCAFG00000012293	ADCY4	28.30481204	13.28849118	13.01485366
ENSCAFG00000031556	MBD6	79.25347372	36.79889865	69.80694238
ENSCAFG00000048887		0.362301594	0	0
ENSCAFG00000015513	USE1	409.8536784	352.6561121	351.4010489
ENSCAFG00000029288		0	6.133149776	3.549505545
ENSCAFG00000029344		19.42842299	9.537047901	11.18094247
ENSCAFG00000005812		3169.006757	2676.097685	3978.995716
ENSCAFG00000016917	SYT11	2.264384964	6.133149776	3.549505545
ENSCAFG00000004180	SAE1	176.6220272	229.9931166	195.222805
ENSCAFG00000009402		0	0	2.36633703
ENSCAFG00000031611	EFCAB8	533.2626589	324.0858561	279.4644032
ENSCAFG00000020121	ABCA4	23.77604212	20.44383259	11.83168515
ENSCAFG00000009245	RHOBTB2	16.98288723	36.79889865	47.3267406
ENSCAFG00000012431	SUMO1	719.2592398	795.8784025	631.6108483
ENSCAFG00000019298	PAFAH1B1	520.8085416	442.6089755	434.222845
ENSCAFG00000023267	PRIM2	78.12128124	82.79752197	60.34159426
ENSCAFG00000019228	SMIM24	2.264384964	0	0
ENSCAFG00000019486	MEIOB	88.31101358	53.15396472	98.20298674
ENSCAFG00000008812	TTI1	75.85689628	79.73094708	120.6831885
ENSCAFG00000010427	IRX2	3.396577445	3.066574888	0
ENSCAFG00000007552	CCDC110	0	0	0
ENSCAFG00000002489	PLEKHH2	36.23015942	52.13177309	47.3267406
ENSCAFG00000047159		9.057539854	1.022191629	0
ENSCAFG00000005554	ENPP7	1.132192482	0	0
ENSCAFG00000016089	PARP1	344.1865145	433.4092508	637.7278296
ENSCAFG00000001017	ZBTB9	21.51165715	26.57698236	57.97525723
ENSCAFG00000049308		60.00620153	109.3745043	92.28714417
ENSCAFG00000010710	KCTD3	318.1460874	463.0528081	524.1436521

ENSCAFG00000017228		303.4275851	484.7846021	341.5807503
ENSCAFG00000010519	DDX25	46.41989175	25.55479073	11.83168515
ENSCAFG00000000893	PNPLA3	202.6624542	43.95424006	87.55447011
ENSCAFG00000006402	CCKBR	1.132192482	0	1.183168515
ENSCAFG00000002380	ACTR1B	232.0994588	123.6851871	133.6980422
ENSCAFG00000012762	COCH	15.85069474	61.33149776	26.02970733
ENSCAFG00000018362	YES1	895.5642531	1460.711838	1474.22797
ENSCAFG00000003663	GSN	1089.169167	222.8377752	131.3317052
ENSCAFG00000015813	PADI2	22.64384964	2.044383259	2.36633703
ENSCAFG00000005723	PSD2	200.3980693	252.4813324	359.6832286
ENSCAFG00000014074	CRYAB	23.77604212	13.28849118	26.02970733
ENSCAFG00000016761	CPEB4	1197.859646	955.7491734	663.7575369
ENSCAFG00000007443	PXMP4	46.41989175	141.0624448	112.4010089
ENSCAFG00000000954	WDR46	420.0434107	264.747632	295.7921287
ENSCAFG00000001574	TSPO2	0	0	0
ENSCAFG00000004170	DHX34	89.44320606	33.73232377	53.24258317
ENSCAFG00000010365		292.1056603	606.1596362	401.0941266
ENSCAFG00000003871	EPS15	674.7867191	1132.588325	960.7328342
ENSCAFG00000006468	NLRP6	0	4.088766517	0
ENSCAFG00000018912	RMI2	0	0	2.36633703
ENSCAFG00000024938		1.947371069	0	2.129703327
ENSCAFG00000034977		1.132192482	0	0
ENSCAFG00000006992	ARL1	460.8023401	626.6034687	543.0743484
ENSCAFG00000002947	PRSS35	0	1.022191629	0
ENSCAFG00000047413	TMEM18	131.3343279	115.5076541	114.767346
ENSCAFG00000031371	PYGO2	36.23015942	35.77670702	53.24258317
ENSCAFG00000010811	LRRC28	340.789937	377.1887112	524.1436521
ENSCAFG00000019425	AP3D1	708.7524936	570.3829291	857.7971734
ENSCAFG00000010369	AZIN2	27.17261956	16.35506607	42.59406654
ENSCAFG00000012368	SLCO1A2	317.0138949	1644.706331	1094.430876
ENSCAFG00000023751		0	0	0
ENSCAFG00000018854	SEZ6	0	0	0
ENSCAFG00000016784	DFFA	189.0761445	197.2829844	240.1832085
ENSCAFG00000028849	TMEM141	35.09796694	57.24273124	52.05941466
ENSCAFG00000013958	FAH	6041.379083	4310.582101	5187.01077
ENSCAFG00000008112	CCDC66	193.6049144	164.5728523	115.9505145
ENSCAFG00000043083	CDK2AP1	31.49759484	6.634023674	45.80045321

ENSCAFG00000005988	CHGB	0	0	0
ENSCAFG00000029321	WNT5A	0	1.022191629	0
ENSCAFG00000049921		0	0	0
ENSCAFG00000019723	CAMSAP1	149.4494076	134.9292951	233.0841974
ENSCAFG00000014698	CRLF1	1.132192482	0	0
ENSCAFG00000038859		0	0	0
ENSCAFG00000006128	SEMA3D	149.4494076	88.93067175	124.2326941
ENSCAFG00000015264	ARMH4	3.396577445	1.022191629	0
ENSCAFG00000030895	CA10	2.264384964	1.022191629	14.19802218
ENSCAFG00000016472	MFN2	349.8474769	437.4980173	776.1585458
ENSCAFG00000029853	CDKN1A	95.10416847	31.68794051	13.01485366
ENSCAFG00000008515	PPP1CC	635.1599823	1281.828303	785.6238939
ENSCAFG00000003410	ST7	360.0372092	242.2594161	268.5792529
ENSCAFG00000003251	EIPR1	164.1679099	183.9944933	120.6831885
ENSCAFG00000043630		54.34523913	104.2635462	119.50002
ENSCAFG00000018423	ITGA2	0	6.133149776	4.73267406
ENSCAFG00000017222	SPTLC2	221.9097264	177.8613435	265.0297474
ENSCAFG00000032613		0	1.022191629	2.36633703
ENSCAFG00000014929	CHD4	543.4523913	481.4522574	1140.574448
ENSCAFG00000000108	ESYT1	473.2564574	462.0306164	832.9506345
ENSCAFG00000001315	CTNNA1	1126.531519	1330.893501	1372.475477
ENSCAFG00000000133	WDR7	98.50074591	111.4188876	112.4010089
ENSCAFG00000016060	RARA	470.9920724	210.5714756	435.4060135
ENSCAFG00000029425	SRPK3	0	0	0
ENSCAFG00000004968	FBF1	15.85069474	20.44383259	24.84653881
ENSCAFG00000010366	ALDH5A1	2185.13149	1856.299999	2460.990511
ENSCAFG00000012078	ZNF311	19.24727219	21.46602421	27.21287584
ENSCAFG00000048374		10.18973234	2.044383259	17.74752772
ENSCAFG00000005957	DPF1	0	0	0
ENSCAFG00000032347	RRAGA	3032.011466	311.7684469	369.1485767
ENSCAFG00000023338	JUND	3393.180868	937.349724	346.6683749
ENSCAFG00000015033	SH2D5	0	0	0
ENSCAFG00000043733		3.396577445	2.044383259	0
ENSCAFG00000017895	ZNF396	12.4541173	12.26629955	9.46534812
ENSCAFG00000046564		6321.030626	7674.614753	5061.594907
ENSCAFG00000011331	ANKRD35	2.264384964	1.022191629	7.09901109
ENSCAFG00000005915	LRRC45	116.6158256	84.84190523	80.45545902

ENSCAFG00000013952	TACR2	0	0	0
ENSCAFG00000032286		1823.713006	183.8104988	251.9084085
ENSCAFG00000019128	SEC14L5	0	0	0
ENSCAFG00000025389	SNAP47	61.13839402	104.2635462	91.10397565
ENSCAFG00000041148	HSP90AB1	5898.383172	6902.461418	7374.511879
ENSCAFG00000024169	PRR22	6.849764515	3.076796804	4.744505745
ENSCAFG00000017366	KANK2	151.7137926	122.6629955	170.3762662
ENSCAFG00000045223		1.132192482	0	1.183168515
ENSCAFG00000008434	ATP2A2	618.1770951	472.2525327	576.2030668
ENSCAFG00000002316	MLIP	108.6904783	27.59917399	39.04456099
ENSCAFG00000008467	EDIL3	0	1.022191629	0
ENSCAFG00000030384	RAB32	78.12128124	87.90848012	106.4851663
ENSCAFG00000013539	CCNYL1	29.43700453	23.51040747	23.6633703
ENSCAFG00000009782	PRICKLE1	3.396577445	21.46602421	30.76238139
ENSCAFG00000028804	RNF150	4.528769927	1.022191629	1.183168515
ENSCAFG00000003202	UTP11	498.164692	412.9654182	295.7921287
ENSCAFG00000019784		0	1.022191629	0
ENSCAFG00000018302	PCP2	0	0	0
ENSCAFG00000019866	CDT1	237.7604212	56.22053961	124.2326941
ENSCAFG00000015745	EVC	53.21304664	186.0388765	188.1237939
ENSCAFG00000002997	COL9A2	544.5845837	293.3689976	225.9851864
ENSCAFG00000018408	BRF1	141.5240602	99.15258804	179.8416143
ENSCAFG00000019567	RNF207	2.264384964	0	3.549505545
ENSCAFG00000023607	ZNF622	2134.182828	800.3760457	840.0496456
ENSCAFG00000044806		0	0	0
ENSCAFG00000042031		0	0	0
ENSCAFG00000004389	ABHD12	113.2192482	100.1747797	108.8515034
ENSCAFG00000001762	TMEM229A	0	0	0
ENSCAFG00000017392	PRKCSH	1313.343279	719.622907	1470.678464
ENSCAFG00000007875	TMSB10	388.3420213	135.9514867	92.28714417
ENSCAFG00000004776	TRANK1	181.1507971	347.545154	535.9753373
ENSCAFG00000015574	DUSP27	0	0	0
ENSCAFG00000013838	MASP1	1521.666696	994.5924553	1555.866597
ENSCAFG00000002240	HINT2	1376.746058	1142.810242	896.8417343
ENSCAFG00000016994	RPS6KL1	4.528769927	0	0
ENSCAFG00000010919	HEATR1	188.1137808	226.4869993	200.3104296
ENSCAFG00000018127	TMC7	1.132192482	1.022191629	1.183168515

ENSCAFG00000013062	DOCK1	309.0885475	353.6783037	397.544621
ENSCAFG00000016629	SOCS7	9.057539854	16.35506607	17.74752772
ENSCAFG00000015872	TSPAN14	178.8864121	256.5700989	250.8317252
ENSCAFG00000028589	SELENOP	7147.531137	16136.31706	9939.798694
ENSCAFG00000010408	MLEC	90.57539854	166.6172356	173.9257717
ENSCAFG00000005851		14.10711832	68.91615965	11.13361573
ENSCAFG00000004791	TMEM53	472.1242649	434.4314424	584.4852464
ENSCAFG00000031319	TMEM236	2.264384964	8.177533034	3.549505545
ENSCAFG00000006719	ACTR6	100.7651309	149.2399779	95.83664971
ENSCAFG00000012650	TTC36	3458.848032	3278.168555	2843.153941
ENSCAFG00000008284	EXTL3	122.276788	221.8155836	266.2129159
ENSCAFG00000019165	GABRQ	0	0	0
ENSCAFG00000018042	SNX33	74.7247038	58.26492287	104.1188293
ENSCAFG00000042315		2.264384964	1.022191629	3.549505545
ENSCAFG00000008582	VCAN	90.57539854	20.44383259	14.19802218
ENSCAFG00000013769		1527.327658	718.6007154	643.6436721
ENSCAFG00000009975	SLC2A13	36.23015942	102.2191629	132.5148737
ENSCAFG00000003366		29.43700453	12.26629955	29.57921287
ENSCAFG00000010883	FMO5	3042.201199	9237.545754	6025.877247
ENSCAFG00000007569	CEP70	40.75892934	27.59917399	10.64851663
ENSCAFG00000018415	MOCS2	1799.053854	2004.517785	1465.94579
ENSCAFG00000029612	MSS51	2.264384964	3.066574888	0
ENSCAFG00000017462	NSMCE1	886.5067132	888.2845258	752.4951755
ENSCAFG00000016815	PSEN1	224.1741114	336.301046	314.722825
ENSCAFG00000002349	UBE2M	287.5768904	217.726817	211.7871642
ENSCAFG00000018507	UTP6	126.805558	137.99587	106.4851663
ENSCAFG00000012482		4.528769927	0	0
ENSCAFG00000023628	NME1	843.4833989	595.9377199	428.3070024
ENSCAFG00000016575	DRAXIN	0	0	0
ENSCAFG00000013331	SRP54	388.3420213	603.0930613	475.633743
ENSCAFG00000023070	ACYP2	318.1460874	275.9917399	231.9010289
ENSCAFG00000002608	PPP6R1	286.4446979	220.7933919	451.9703727
ENSCAFG00000030310		4.528769927	2.044383259	2.36633703
ENSCAFG00000007912	TRPC4AP	623.8380575	584.6936119	937.0694639
ENSCAFG00000042913		0	0	0
ENSCAFG00000009217	CHMP7	74.7247038	145.1512114	108.8515034
ENSCAFG00000002703		774.4196575	556.0722463	475.633743

ENSCAFG00000013068	ATF6	257.0076934	286.2136562	491.0149337
ENSCAFG00000028527	TRMT10B	69.06374139	55.19834798	63.89109981
ENSCAFG00000048265		16.98288723	23.51040747	16.56435921
ENSCAFG00000004512	CCDC89	3.396577445	9.199724663	4.73267406
ENSCAFG00000048452		0	0	0
ENSCAFG00000010317	METAP1	134.7309053	387.4106275	345.4852064
ENSCAFG00000012707	PSMB4	2110.406786	1883.899173	1480.143812
ENSCAFG00000012011	BMP7	0	0	0
ENSCAFG00000002483	TBC1D2	5.660962409	7.155341405	8.282179605
ENSCAFG00000010432	TIRAP	113.2192482	132.8746899	73.27362613
ENSCAFG00000004324	TMEM60	53.21304664	54.17615635	35.49505545
ENSCAFG00000003189	RHBDL2	1.132192482	0	0
ENSCAFG00000048191		2.264384964	3.066574888	0
ENSCAFG00000005288	TIE1	58.87400905	44.97643169	31.9455499
ENSCAFG00000014312		11.77480181	1.022191629	5.217773151
ENSCAFG00000001624	CYHR1	244.5535761	131.8627202	209.4208272
ENSCAFG00000032662		54.16408833	51.8864471	70.93095247
ENSCAFG00000013100	NCBP2	433.6297205	426.2539094	405.8268006
ENSCAFG00000002937	POLD1	63.40277898	48.04300658	49.69307763
ENSCAFG00000001237	PTP4A3	33.96577445	8.177533034	8.282179605
ENSCAFG00000017655		576.2859732	310.7462553	323.0050046
ENSCAFG00000010629	SPICE1	65.66716394	101.1969713	70.9901109
ENSCAFG00000023724		432.4409184	282.3906595	463.1631469
ENSCAFG00000010357	MPHOSPH10	286.4446979	249.4147575	188.1237939
ENSCAFG00000006250	SPRY4	4.528769927	9.199724663	8.282179605
ENSCAFG00000008892	URB1	91.70759102	62.35368939	177.4752772
ENSCAFG00000012020	TRIM27	134.7309053	124.7073788	88.73763862
ENSCAFG00000004636	BCAM	310.22074	216.7046254	252.0148937
ENSCAFG00000007764	HIP1R	296.6344302	306.6574888	473.267406
ENSCAFG00000007954	TNKS1BP1	777.816235	373.0999447	804.5545902
ENSCAFG00000018651		438.6566551	279.2116435	493.6415678
ENSCAFG00000016646	SNCB	0	2.044383259	1.183168515
ENSCAFG00000018790	AGXT2	2382.132982	2179.312554	2555.643992
ENSCAFG00000012757	SEC14L3	690.6374139	2258.021309	1564.148777
ENSCAFG00000015068	ITIH3	10880.36975	4214.496087	19138.9339
ENSCAFG00000005998	LHFPL6	304.5597776	127.7739537	145.5297273
ENSCAFG00000014980	FMO4	90.57539854	144.1290197	50.87624614

ENSCAFG00000000039	DOK6	3.396577445	1.022191629	0
ENSCAFG00000028550		22.64384964	42.08362938	35.77901589
ENSCAFG00000003905	AMD1	102.4747415	58.8475721	52.1895632
ENSCAFG00000004788	TFCP2L1	0	0	2.36633703
ENSCAFG00000005511	RPGRIP1	2.264384964	0	0
ENSCAFG00000017906	WARS	295.5022377	228.970925	274.4950955
ENSCAFG00000024687		3.690947491	10.22191629	10.64851663
ENSCAFG00000013615	FRMD8	19.24727219	26.57698236	35.49505545
ENSCAFG00000000617	DDAH2	32.83358197	19.42164096	11.83168515
ENSCAFG00000043188		0	2.044383259	4.73267406
ENSCAFG00000018775	LONP1	840.0868215	639.8919599	1057.752652
ENSCAFG00000008457	PCGF1	58.87400905	44.97643169	24.84653881
ENSCAFG00000005875	FAM98A	366.8303641	292.346806	352.5842175
ENSCAFG00000046938		0	0	0
ENSCAFG00000008273		117.2045657	83.33928353	74.30298274
ENSCAFG00000017727	ADPGK	242.2891911	247.3703743	286.3267806
ENSCAFG00000046760		0	0	0
ENSCAFG00000003960	TRAF3IP2	526.469504	91.99724663	102.9356608
ENSCAFG00000016118	SRARP	0	0	0
ENSCAFG00000002590		199.2658768	979.2595808	940.6189694
ENSCAFG00000001786	ACO1	1485.436536	1846.078082	3815.718461
ENSCAFG00000005252	TMC6	195.8692993	109.3745043	153.8119069
ENSCAFG00000017283	TIPIN	67.93154891	94.04162989	63.89109981
ENSCAFG00000006417	LZTS3	40.75892934	39.86547354	66.25743684
ENSCAFG00000017863	MYOCD	1.132192482	0	0
ENSCAFG00000009617	IFIT1	23.77604212	27.59917399	10.64851663
ENSCAFG00000014903	POLN	0	0	0
ENSCAFG00000044861		0	1.022191629	2.36633703
ENSCAFG00000013033	SF3B2	773.2874651	730.8670149	933.5199583
ENSCAFG00000008143	MSRA	926.1334501	1226.629955	1283.737839
ENSCAFG00000004839	UPF2	138.1274828	273.9473566	436.589182
ENSCAFG00000016982	DOCK2	104.1617083	61.33149776	75.72278496
ENSCAFG00000048282		1.415240602	1.533287444	5.53722865
ENSCAFG00000017131	CLPX	721.2066109	2147.624613	1850.475557
ENSCAFG00000005830	ASPSCR1	1281.641889	986.4149222	1248.242783
ENSCAFG00000012960	SEPTIN2	450.6126077	469.1859578	598.6832686
ENSCAFG00000011536	ZKSCAN8	6.793154891	15.33287444	15.38119069

ENSCAFG00000017004	PPP1R9B	183.415182	58.26492287	131.3317052
ENSCAFG00000044149		2.638008483	1.10396696	3.821634303
ENSCAFG00000000918	MCAT	134.7309053	137.99587	179.8416143
ENSCAFG00000041399	FAM237A	2.264384964	2.044383259	4.73267406
ENSCAFG00000010798	TNNT2	2.264384964	5.110958146	1.183168515
ENSCAFG00000010979	ARMC9	19.24727219	7.155341405	14.19802218
ENSCAFG00000018271		0	0	0
ENSCAFG00000017477	ZKSCAN2	7.925347372	9.199724663	5.915842575
ENSCAFG00000007455	RCN1	241.1569986	222.8377752	299.3416343
ENSCAFG00000049607		0	0	0
ENSCAFG00000012789	PTPRO	1.132192482	2.044383259	0
ENSCAFG00000031441	WNT9A	21.51165715	32.71013214	21.29703327
ENSCAFG00000001405	NRBP2	581.9469356	649.0916846	698.0694238
ENSCAFG00000004554	GPR142	0	0	0
ENSCAFG00000011630		20.37946467	16.35506607	9.46534812
ENSCAFG00000007047	MAL	0	0	0
ENSCAFG00000009577	RPAP1	78.12128124	116.5298457	128.9653681
ENSCAFG00000002043	MEP1A	4.528769927	0	0
ENSCAFG00000032230		427.6177784	285.7025604	301.8854466
ENSCAFG00000002770	FKTN	15.85069474	56.69074776	68.62377387
ENSCAFG00000009603	SLC35B3	144.9206377	240.2150329	145.5297273
ENSCAFG00000018126	ULK2	235.4960362	211.5936673	308.8069824
ENSCAFG00000013466	LRRC15	0	0	0
ENSCAFG00000007862	SLC43A1	244.5535761	377.1887112	625.8961444
ENSCAFG00000004245	KIF3C	14.71850226	9.199724663	10.64851663
ENSCAFG00000018220	PEX11G	131.775883	62.35368939	100.7941258
ENSCAFG00000002162	MAP4K4	332.8645896	394.5659689	250.8317252
ENSCAFG00000017724	INTS2	18.11507971	62.35368939	56.79208872
ENSCAFG00000015921	LRIT1	0	0	1.183168515
ENSCAFG00000043091	CDV3	110.9548632	97.10820478	141.9802218
ENSCAFG00000011175	NKAIN1	3.396577445	3.066574888	3.549505545
ENSCAFG00000002297	LIPT1	237.7604212	168.6616188	136.0643792
ENSCAFG00000000848	DLA-DMA	343.054322	321.9903632	306.4406454
ENSCAFG00000011086	SLC16A6	13.58630978	5.110958146	2.36633703
ENSCAFG00000031806		488.529734	97.4353061	87.48348
ENSCAFG00000024327	NOL3	4.528769927	2.044383259	0
ENSCAFG00000010897	ARHGEF38	6.793154891	4.088766517	3.549505545

ENSCAFG00000013678	MBIP	139.2596753	152.3065528	102.9356608
ENSCAFG00000041833		0	13.28849118	26.02970733
ENSCAFG00000006383	BRCA2	41.89112183	42.93204843	24.84653881
ENSCAFG00000005628	P2RY6	2.264384964	1.022191629	0
ENSCAFG00000019237	ADCY9	283.0481204	266.7920152	739.4803219
ENSCAFG00000002143	DYNC1I1	26.04042708	5.110958146	9.46534812
ENSCAFG00000008442	ANAPC7	124.541173	163.5506607	138.4307163
ENSCAFG00000012084	ADCY5	109.8226707	25.55479073	52.05941466
ENSCAFG00000002793	IRAK1BP1	6.793154891	5.110958146	3.549505545
ENSCAFG00000016466	ALDH1A2	22.64384964	31.68794051	18.93069624
ENSCAFG00000044174		0	0	0
ENSCAFG00000018083	TMEM164	35.09796694	42.93204843	73.35644793
ENSCAFG00000028745	OBP	0	1.022191629	0
ENSCAFG00000028754	PLPP3	1153.704139	1087.611894	1464.762622
ENSCAFG00000007499	SLC7A9	0	0	0
ENSCAFG00000005186		3.928707912	6.000264864	7.205496256
ENSCAFG00000013517	NBEAL2	71.32812635	37.82109028	63.89109981
ENSCAFG00000042877		3689.713401	3101.789389	2391.065252
ENSCAFG00000023756	GNAS	4335.165013	2473.703743	3693.852104
ENSCAFG00000025153	TTC9	1.132192482	0	0
ENSCAFG00000010619	BICD1	12.4541173	14.31068281	5.915842575
ENSCAFG00000002023	RCAN2	80.38566621	27.59917399	27.21287584
ENSCAFG00000019767	REXO4	677.0511041	556.0722463	746.5793329
ENSCAFG00000030268	GPBAR1	22.64384964	4.088766517	10.64851663
ENSCAFG00000003581	PHF19	14.71850226	4.088766517	10.64851663
ENSCAFG00000011362		989.5362291	624.5590855	1184.351683
ENSCAFG00000044010		7.925347372	11.24410792	14.19802218
ENSCAFG00000049087		0	0	0
ENSCAFG00000004754	MCM10	36.69435833	19.24786838	46.10807703
ENSCAFG00000005634	NFKBIB	290.9734678	299.5021474	218.8861753
ENSCAFG00000007651	DTX4	13.58630978	16.35506607	14.19802218
ENSCAFG00000019221	PRPF8	1703.949685	1329.87131	2546.178644
ENSCAFG00000018162	HRH4	4.528769927	0	2.36633703
ENSCAFG00000010241	PXN	56.60962409	51.10958146	79.2722905
ENSCAFG00000032078		79.25347372	8.177533034	4.73267406
ENSCAFG00000011172	FSTL1	230.9672663	180.9279184	158.544581
ENSCAFG00000017079	CSNK1G1	4801.628315	2846.803688	2436.143972

ENSCAFG00000005911	MYADML2	0	0	0
ENSCAFG00000020181	GAPVD1	292.1056603	349.5895372	370.3317452
ENSCAFG00000019964	DNAAF1	2.264384964	0	0
ENSCAFG00000011188	GALNT3	32.83358197	19.42164096	23.6633703
ENSCAFG00000023729	TSPYL1	73.59251132	35.77670702	60.34159426
ENSCAFG00000030772	PALB2	32.8901916	79.84338816	67.44060535
ENSCAFG00000016670	SAT2	1436.752259	1418.801981	1051.83681
ENSCAFG00000001669	ARHGAP39	14.71850226	16.35506607	35.49505545
ENSCAFG00000005872	ARHGDIA	1041.617083	689.9793498	1125.193258
ENSCAFG00000010450	SLC37A1	35.09796694	4.088766517	5.915842575
ENSCAFG00000015929	N4BP2	130.2021354	481.4522574	377.4307563
ENSCAFG00000018151	SLC47A2	24.9082346	12.26629955	20.11386475
ENSCAFG00000041773		217.3356688	258.7984767	426.331111
ENSCAFG00000015011	TMEM129	48.68427672	66.4424559	108.8515034
ENSCAFG00000033391		14.71850226	27.59917399	11.83168515
ENSCAFG00000047621		0	0	0
ENSCAFG00000023746	TDRD3	151.8496557	228.8175962	217.7739969
ENSCAFG00000018798	ANGPTL3	10806.77724	19786.56337	16844.77015
ENSCAFG00000024672	TMEM211	0	0	0
ENSCAFG00000002758	SLC44A1	275.1227731	376.1665196	463.8020579
ENSCAFG00000001138		0	0	0
ENSCAFG00000010409	RHBDD1	19.24727219	49.0651982	37.86139248
ENSCAFG00000008414		1462.792686	850.4634356	1564.148777
ENSCAFG00000043554	RALYL	0	0	0
ENSCAFG00000014687	TMEM59L	2.264384964	1.022191629	1.183168515
ENSCAFG00000023821	CYP2F1	0	0	0
ENSCAFG00000006786	SLC17A8	1.132192482	0	13.01485366
ENSCAFG00000006138	LUM	829.8970891	199.3273677	75.72278496
ENSCAFG00000007686	SLCO5A1	1.132192482	0	0
ENSCAFG00000019142	LRP8	271.7261956	176.8391519	125.4158626
ENSCAFG00000007681	MFAP3L	108.6904783	310.7462553	388.0792729
ENSCAFG00000019103	TMEM185A	121.1445956	55.19834798	84.00496456
ENSCAFG00000019273		171.0629621	246.8592785	401.0941266
ENSCAFG00000002392	ZSCAN18	98.50074591	109.3745043	176.2921087
ENSCAFG00000018354	AKT1	543.4523913	353.6783037	598.6832686
ENSCAFG00000014358	CASK	41.89112183	69.50903079	55.6089202
ENSCAFG00000004880	CFAP221	16.98288723	9.199724663	1.183168515

ENSCAFG00000019781	MYMK	0	0	0
ENSCAFG00000017336	SBK1	13.58630978	8.177533034	46.14357208
ENSCAFG00000030099	C1D	129.0699429	79.73094708	53.24258317
ENSCAFG00000016696	TMED11	16.98288723	44.97643169	59.15842575
ENSCAFG00000007261	ACOX2	4930.698258	5340.951263	6328.768387
ENSCAFG00000024171	SPINK2	74.7247038	82.79752197	65.07426832
ENSCAFG00000031424	RAP2A	44.35930144	33.73232377	27.28386596
ENSCAFG00000041393		0	0	0
ENSCAFG00000010812	NCOA3	74.7247038	145.1512114	117.133683
ENSCAFG00000012457		335.1289746	379.2330945	359.6832286
ENSCAFG00000005849	TBC1D5	127.9377504	108.3523127	147.8960644
ENSCAFG00000025332		224.1741114	284.1692729	144.3465588
ENSCAFG00000015920	HAP1	0	0	0
ENSCAFG00000024782	LRRC73	2.264384964	0	0
ENSCAFG00000013089	PLBD1	884.2423283	615.3593608	883.8268807
ENSCAFG00000016108	LIN9	16.98288723	51.10958146	21.29703327
ENSCAFG00000001543	TNPO3	389.4742137	446.697742	509.94563
ENSCAFG00000050027		3.034275851	0	2.36633703
ENSCAFG00000019428	HCFC1	203.7946467	78.70875545	300.5248028
ENSCAFG00000016464	ZFYVE26	38.49454438	81.77533034	86.37130159
ENSCAFG00000005685	PAK5	0	0	0
ENSCAFG00000000874	THBS2	109.8226707	43.95424006	72.17327941
ENSCAFG00000030726	TEX38	1.132192482	0	4.73267406
ENSCAFG00000005539	PPME1	49.8164692	45.99862332	49.69307763
ENSCAFG00000042770	MYH1	0	0	0
ENSCAFG00000041568		1.132192482	0	4.73267406
ENSCAFG00000003303	AMBP	70038.55912	45694.01021	68588.27881
ENSCAFG00000000601	GPANK1	100.7651309	108.3523127	108.8515034
ENSCAFG00000002428	ANKMY2	153.7857048	217.246387	184.2548328
ENSCAFG00000024614	PCDHB2	2.570076934	0	0
ENSCAFG00000019101	VPS53	50.94866168	132.8849118	183.3911198
ENSCAFG00000006306	MORN2	12.4541173	20.44383259	11.83168515
ENSCAFG00000010481	YARS	605.7003339	485.0503719	423.4205165
ENSCAFG00000000685	MAPK12	0	0	2.36633703
ENSCAFG00000008536	FAM186B	0	0	0
ENSCAFG00000016600	CCNB2	39.62673686	97.10820478	48.50990911
ENSCAFG00000001355	KIAA2026	133.5987129	168.6616188	195.222805

ENSCAFG00000018363	LONRF3	91.70759102	90.975055	111.2178404
ENSCAFG00000030216	DTD2	9.057539854	26.57698236	23.6633703
ENSCAFG00000017996	ACACA	557.038701	731.8892066	1513.272531
ENSCAFG00000004584	TSC22D1	395.1351761	597.9821031	579.7525723
ENSCAFG00000010210	ACTR1A	612.5161326	562.2053961	650.7426832
ENSCAFG00000048560		3.396577445	22.48821584	3.549505545
ENSCAFG00000005720	CLPB	229.8350738	228.970925	280.410938
ENSCAFG00000017951	DTNA	19.24727219	7.155341405	7.09901109
ENSCAFG00000019882	TSC1	39.62673686	62.35368939	107.6683349
ENSCAFG00000013311	CYP2E1	151078.6326	159978.1009	293460.1036
ENSCAFG00000029254		10.18973234	32.71013214	8.282179605
ENSCAFG00000006159	AP5S1	148.3172151	333.2344711	266.2129159
ENSCAFG00000014551	GAL3ST4	4.528769927	5.110958146	1.183168515
ENSCAFG00000015007	MAPK1IP1L	172.0932572	146.173403	138.4307163
ENSCAFG00000015049	CDA	1124.267134	1666.172356	1069.584338
ENSCAFG00000008210	GREM1	3.396577445	0	0
ENSCAFG00000017615	ST8SIA5	1.132192482	0	1.183168515
ENSCAFG00000004855	EPB41L5	634.0277898	1260.362279	1011.60908
ENSCAFG00000020358	RIPOR1	156.2425625	108.3523127	269.7624214
ENSCAFG00000009641	PI3	10.18973234	1.022191629	2.36633703
ENSCAFG00000031406		55.81708935	36.4513535	60.12862393
ENSCAFG00000003949	WDCP	7.925347372	28.62136562	27.21287584
ENSCAFG00000004682	FAM171A1	135.8630978	109.3745043	240.1832085
ENSCAFG00000018388	SNX18	91.70759102	100.1747797	133.6980422
ENSCAFG00000025084	PAXBP1	166.4322948	242.2594161	181.0247828
ENSCAFG00000042696		772.7779784	759.7541504	506.3606294
ENSCAFG00000023570	HAPLN4	3.396577445	0	1.183168515
ENSCAFG00000019379	ABCA3	283.0481204	110.396696	164.4367602
ENSCAFG00000009094	HPSE	14.71850226	12.26629955	27.21287584
ENSCAFG00000005594	PLEKHB1	2.264384964	3.066574888	1.183168515
ENSCAFG00000009441	CD82	498.164692	602.0708696	819.9357809
ENSCAFG00000016100		1.132192482	1.022191629	0
ENSCAFG00000012603	IFT46	538.9236213	642.9585348	544.2575169
ENSCAFG00000000629	DENND6B	36.23015942	21.46602421	50.87624614
ENSCAFG00000013168	CTSW	48.68427672	8.177533034	29.57921287
ENSCAFG00000000413	MTHFD1L	113.2192482	76.6643722	60.34159426
ENSCAFG00000049446		0	0	0

ENSCAFG00000008233	ORAI1	28.30481204	36.79889865	36.67822396
ENSCAFG00000007133	DEPDC1B	10.18973234	17.3772577	9.46534812
ENSCAFG00000008785	ARL2BP	202.6624542	283.1470813	362.0495656
ENSCAFG00000007826	HCAR1	1.132192482	6.133149776	15.38119069
ENSCAFG00000020393	ACADM	3094.282053	5814.225987	5862.599992
ENSCAFG00000031860	HIST1H2AC	14.48074184	8.433080941	11.52406134
ENSCAFG00000016333		49.8164692	20.44383259	29.57921287
ENSCAFG00000015416	ANO8	82.65005117	40.88766517	57.97525723
ENSCAFG00000041093	SNRNP200	1071.054088	681.8018167	1671.817112
ENSCAFG00000018207	CABLES1	665.7291793	67.46464753	171.5594347
ENSCAFG00000013445	LSG1	182.2829896	150.2621695	160.910918
ENSCAFG00000008942	C18H11orf49	30.56919701	13.28849118	11.83168515
ENSCAFG00000008779	ABCC8	0	0	0
ENSCAFG00000006849	TMEM132C	0	0	0
ENSCAFG00000016794	NAXE	598.9298229	404.7878852	408.1931377
ENSCAFG00000004698	ABCB8	133.5987129	148.2177862	281.5941066
ENSCAFG00000019524	KCNAB2	6.793154891	3.066574888	2.36633703
ENSCAFG00000008595	NUP54	191.3405294	268.8363985	205.8713216
ENSCAFG00000003322	IFRD1	614.7805176	193.1942179	52.05941466
ENSCAFG00000046356		6.793154891	0	3.549505545
ENSCAFG00000020041	ZDHHC12	130.9154167	189.5858815	215.9519174
ENSCAFG00000009285	CCAR2	303.4275851	334.2566628	438.9555191
ENSCAFG00000001196	AGO2	64.53497146	62.35368939	101.7524923
ENSCAFG00000017313	SPNS1	332.9438431	239.9390411	298.1584658
ENSCAFG00000001885	APPL2	276.2549656	380.2552861	455.5198783
ENSCAFG00000005661	ECH1	4391.774637	2910.179569	4583.594827
ENSCAFG00000014019	MAJIN	1.132192482	1.022191629	1.183168515
ENSCAFG00000010813	BDH2	997.4615764	929.172191	745.3961644
ENSCAFG00000030354		7.925347372	11.24410792	13.01485366
ENSCAFG00000000855	IL5	0	0	0
ENSCAFG00000030308	CD70	0	0	0
ENSCAFG00000048895		0	0	0
ENSCAFG00000024335	ZBTB7A	47.55208423	59.2871145	137.2475477
ENSCAFG00000002957	HOXA7	0	0	0
ENSCAFG00000014041	PRPF39	140.3918677	155.3731276	102.9356608
ENSCAFG00000017430	SRSF1	120.0124031	224.8821584	151.4455699
ENSCAFG00000017422	IQCH	0	0	0

ENSCAFG00000017092	TBX6	0	1.022191629	1.183168515
ENSCAFG00000030400	TNFSF9	0	4.088766517	3.549505545
ENSCAFG00000001685	ZNF16	19.24727219	13.28849118	10.64851663
ENSCAFG00000006160	RMDN2	121.1445956	186.0388765	128.9653681
ENSCAFG00000008395	SCARA3	134.7309053	71.55341405	113.5841774
ENSCAFG00000013127	CLRN3	6.793154891	0	1.183168515
ENSCAFG00000001559	SCX	6.793154891	1.022191629	1.183168515
ENSCAFG00000016253	DNAJC16	61.13839402	66.4424559	124.2326941
ENSCAFG00000008948	LYZF2	2.264384964	0	8.282179605
ENSCAFG00000008692	CXCL13	0	0	0
ENSCAFG00000009511	HSD17B13	9527.399734	23180.23958	20112.68159
ENSCAFG00000031025	DAZAP2	806.121047	1189.831056	1107.44573
ENSCAFG00000009363	MAPK8IP1	358.9050167	74.61998894	160.910918
ENSCAFG00000013995	CABIN1	159.6391399	94.04162989	248.4653881
ENSCAFG00000044764		11.32192482	10.22191629	1.183168515
ENSCAFG00000009776	ZSWIM1	7.925347372	9.199724663	10.64851663
ENSCAFG00000005150	GCKR	2668.57768	2563.656606	2589.955879
ENSCAFG00000016132		266.0652332	222.8377752	621.1634704
ENSCAFG00000013253		138.1274828	44.97643169	162.0940866
ENSCAFG00000003455		63.61789555	61.6074895	36.41792689
ENSCAFG00000003938	NTN5	0	0	0
ENSCAFG00000015985	LRTM2	6.793154891	7.155341405	11.83168515
ENSCAFG00000049746		4.528769927	9.20994658	2.36633703
ENSCAFG00000018019	CENPV	177.7542196	154.350936	137.2475477
ENSCAFG00000035304		0	0	0
ENSCAFG00000009527	RREB1	324.9392423	175.8169602	580.9357409
ENSCAFG00000000493	OSR2	0	4.088766517	1.183168515
ENSCAFG00000003737	PARD3	674.7867191	523.3621142	634.178324
ENSCAFG00000030347	C33H3orf52	1.132192482	30.66574888	18.93069624
ENSCAFG00000013514		115.4836331	193.1942179	111.2178404
ENSCAFG00000004858	CIC	220.7775339	65.42026427	230.7178604
ENSCAFG00000001787	KLC4	389.4742137	424.2095261	441.3218561
ENSCAFG00000010223	PRDM15	5.660962409	10.22191629	1.183168515
ENSCAFG00000019582	ATP6AP1	249.082346	215.6824338	269.7624214
ENSCAFG00000000867	RAD50	285.3125054	428.2982927	359.6832286
ENSCAFG00000006973	SOX12	2.264384964	2.044383259	3.549505545
ENSCAFG00000009401	DLL4	12.4541173	61.33149776	26.02970733

ENSCAFG00000016676	BNIP2	341.3107456	573.3983944	326.0575794
ENSCAFG00000026374	SNORA53	1.132192482	2.044383259	1.183168515
ENSCAFG00000004822	ALG8	122.276788	104.2635462	125.4158626
ENSCAFG00000031175	DCTD	129.0699429	105.2857378	100.5693238
ENSCAFG00000012846	PASK	10.18973234	16.35506607	14.19802218
ENSCAFG00000045845		281.3158659	126.6904305	68.75392241
ENSCAFG00000011196	NID1	920.4724877	625.5812771	1933.297353
ENSCAFG00000002552	INVS	113.2192482	282.1248897	214.1535012
ENSCAFG00000049937		0	0	0
ENSCAFG00000044777	ZBTB32	2.264384964	3.066574888	11.83168515
ENSCAFG00000020008	SH3GLB2	245.6857685	104.2635462	227.1683549
ENSCAFG00000001003	CENPM	110.9548632	204.4383259	299.3416343
ENSCAFG00000001075	ACO2	2026.624542	1647.772906	2847.886616
ENSCAFG00000003548	NCDN	98.50074591	53.15396472	114.767346
ENSCAFG00000034757	CCDC194	0	0	0
ENSCAFG00000031691	CRADD	31.70138949	29.64355725	40.22772951
ENSCAFG00000015783	B3GAT3	332.8645896	201.371751	267.3960844
ENSCAFG00000008597	ANGPT2	7.925347372	50.08738983	49.69307763
ENSCAFG00000010406	CPNE8	86.04662862	54.17615635	86.37130159
ENSCAFG00000009393	SYT13	4.528769927	0	0
ENSCAFG00000035612		0	2.044383259	0
ENSCAFG00000019322	GNG10	773.2648212	930.1943826	1019.89126
ENSCAFG00000020110	F3	202.6624542	116.5298457	55.6089202
ENSCAFG00000015697	KCND1	2.264384964	3.066574888	0
ENSCAFG00000002526	P3H1	243.4213836	144.1290197	199.955479
ENSCAFG00000049587		2.264384964	1.022191629	2.36633703
ENSCAFG00000010944	CHD1L	628.3668274	413.9876099	496.9307763
ENSCAFG00000010478	CTTN	851.4087463	828.9974113	772.6090403
ENSCAFG00000011339	LHX9	0	0	4.73267406
ENSCAFG00000000898	KIF3A	12.4541173	22.48821584	4.73267406
ENSCAFG00000048653		5.660962409	2.044383259	1.183168515
ENSCAFG00000002774	ZNF462	50.94866168	19.42164096	35.49505545
ENSCAFG00000002328	HCRTR2	0	0	0
ENSCAFG00000010346	MED10	113.2192482	129.8183369	100.5693238
ENSCAFG00000011298	VWA2	29.2445318	10.22191629	43.77723505
ENSCAFG00000002078		139.2596753	51.04824997	40.1567394
ENSCAFG00000007882	PCSK1	0	0	0

ENSCAFG00000019342	AGRN	201.5302618	96.08601315	177.4752772
ENSCAFG00000013047	TRIM50	0	0	0
ENSCAFG00000004856	PROSER2	183.415182	320.9681716	285.1436121
ENSCAFG00000002612	SMAP1	67.93154891	93.01943826	85.18813308
ENSCAFG00000017715	SLC44A2	278.5193505	198.3051761	294.6089602
ENSCAFG00000019864	GBGT1	273.9905806	195.2386012	228.3515234
ENSCAFG00000018512		0	1.022191629	0
ENSCAFG00000047361		3.838132513	2.310153082	0
ENSCAFG00000005692	RPTOR	156.2425625	83.8197136	267.3960844
ENSCAFG00000018920	CLEC16A	83.78224365	69.50903079	95.83664971
ENSCAFG00000010264	WBP1L	1055.203393	776.8656382	879.0942066
ENSCAFG00000017100	ADAM15	210.5878016	128.8165891	162.0940866
ENSCAFG00000007338	NR4A1	425.7043731	531.5396472	8.282179605
ENSCAFG00000030403	LRAT	922.7368727	561.1832045	584.4852464
ENSCAFG00000043718		0	0	0
ENSCAFG00000028544		0	0	0
ENSCAFG00000015946	CCSER2	200.3980693	204.4383259	326.5545101
ENSCAFG00000002772	PPP2R1A	576.2859732	475.3191076	667.3070424
ENSCAFG00000048614		0	0	0
ENSCAFG00000019150	ATCAY	0	0	0
ENSCAFG00000010560	CFAP43	0	0	0
ENSCAFG00000017823		0	0	0
ENSCAFG00000042389		0	0	0
ENSCAFG00000009249		0	0	0
ENSCAFG00000007297	PMCH	0	0	0
ENSCAFG00000013302	BAZ1A	169.8288723	167.6394272	144.3465588
ENSCAFG00000005033	SLC6A1	790.2703523	910.7727417	3814.535292
ENSCAFG00000028760		186.8117595	130.8405285	145.3759154
ENSCAFG00000009056	ADAM28	13.58630978	12.26629955	2.36633703
ENSCAFG00000030330	CPTP	24.9082346	44.97643169	36.67822396
ENSCAFG00000050081		5.660962409	0	1.183168515
ENSCAFG00000015464	PIMREG	1.132192482	1.022191629	0
ENSCAFG00000015425	BHLHA15	1.132192482	0	0
ENSCAFG00000003479	CNTNAP2	55.47743161	38.84328191	9.46534812
ENSCAFG00000012028	DHRS9	0	0	0
ENSCAFG00000008237	HMBOX1	53.21304664	60.30930613	62.70793129
ENSCAFG00000005023	HRH1	1.132192482	1.022191629	1.183168515

ENSCAFG00000008523	P2RY14	3.396577445	0	0
ENSCAFG00000048198		0	0.020443833	0
ENSCAFG00000003153	VPS54	277.387158	326.0791297	483.9159226
ENSCAFG00000028803	ANKRD66	0	0	1.183168515
ENSCAFG00000006612	SUCLG2	2376.472019	5569.922188	5988.015854
ENSCAFG00000003684		0	0	2.36633703
ENSCAFG00000009193	AMBRA1	134.7309053	115.5076541	182.2079513
ENSCAFG00000013106	ATF7IP	125.6733655	217.726817	252.0148937
ENSCAFG00000019650	CAMTA1	193.6049144	160.4840858	126.5990311
ENSCAFG00000005406		138.1274828	74.61998894	14.19802218
ENSCAFG00000003716	NAA15	279.651543	388.4328191	378.6139248
ENSCAFG00000002940	FANCL	129.5341418	119.0751029	109.5377411
ENSCAFG00000004362	GPR17	4.528769927	1.022191629	0
ENSCAFG00000010939	MON1A	149.4494076	113.4632708	181.0247828
ENSCAFG00000010207	BDNF	1.132192482	1.022191629	3.549505545
ENSCAFG00000030984	DUSP22	11.32192482	9.199724663	8.282179605
ENSCAFG00000044907		0	0.337323238	0
ENSCAFG00000041410		0	0	0
ENSCAFG00000017235	FBXW9	33.96577445	21.46602421	20.11386475
ENSCAFG00000016736	FBXL19	319.2782799	394.5659689	341.9357008
ENSCAFG00000004837		12.4541173	5.110958146	8.282179605
ENSCAFG00000000135	BAZ2A	286.4446979	155.3731276	301.7079713
ENSCAFG00000009490	KLHL8	38.49454438	116.5298457	93.47031268
ENSCAFG00000011643	AP1AR	133.5987129	391.499394	195.222805
ENSCAFG00000045483		5.660962409	2.044383259	7.09901109
ENSCAFG00000048831		29.43700453	16.52883865	34.31188693
ENSCAFG00000009071	IL10RB	0	0	0
ENSCAFG00000017455	GAS7	4.528769927	10.22191629	14.19802218
ENSCAFG00000014085	MIS18BP1	26.04042708	45.99862332	28.39604436
ENSCAFG00000004316	GSAP	10.18973234	12.26629955	13.01485366
ENSCAFG00000009459	FMOD	190.2083369	116.5298457	207.0544901
ENSCAFG00000007581	PATL1	93.97197599	89.95286338	207.0544901
ENSCAFG00000000988	NAGA	148.3172151	145.1512114	230.7178604
ENSCAFG00000013126	FOXI2	0	0	0
ENSCAFG00000006211	ERLIN2	143.7884452	271.9029734	351.4010489
ENSCAFG00000004097	CYP4B1	0	0	0
ENSCAFG00000000981	DAXX	113.2192482	90.975055	72.17327941

ENSCAFG00000007908	PDE6C	26.04042708	49.0651982	85.18813308
ENSCAFG00000010500	WDR4	219.6453415	71.55341405	84.00496456
ENSCAFG00000043350		0	0	0
ENSCAFG00000050042		0	0	0
ENSCAFG00000023925	HSPB2	39.62673686	12.26629955	15.38119069
ENSCAFG00000001148	ST3GAL1	382.6810588	217.726817	636.5446611
ENSCAFG00000002402	DGKB	3.396577445	3.066574888	2.36633703
ENSCAFG00000007191	KIF2A	105.2939008	122.6629955	227.1683549
ENSCAFG00000008883	TLL1	0	3.066574888	0
ENSCAFG00000032565	TAFA2	0	0	0
ENSCAFG00000005066	WDR48	75.85689628	225.9043501	196.4059735
ENSCAFG00000008635	SOGA1	22.64384964	10.22191629	9.46534812
ENSCAFG00000008659	SAMHD1	15.85069474	65.42026427	44.96040357
ENSCAFG00000038628		24.20627526	10.22191629	8.282179605
ENSCAFG00000003840		4.528769927	3.066574888	1.183168515
ENSCAFG00000013475	HERC4	149.4494076	274.9695483	231.9010289
ENSCAFG00000031937	SLC35C1	69.06374139	58.26492287	93.47031268
ENSCAFG00000002825	GSDME	9.057539854	5.110958146	1.183168515
ENSCAFG00000014128	EHD1	372.4913265	520.2955393	845.9654882
ENSCAFG00000024277	C31H21orf62	3.396577445	6.133149776	3.549505545
ENSCAFG00000013052	TANC2	18.11507971	15.33287444	21.29703327
ENSCAFG00000044731		1.585069474	2.259043501	0
ENSCAFG00000011769	ANK2	18.11507971	27.59917399	20.11386475
ENSCAFG00000007987	SUCLG1	3429.411027	5806.048454	4170.669015
ENSCAFG00000011880	GRIK4	3.396577445	1.022191629	1.183168515
ENSCAFG00000016126	SLC2A4	35.09796694	10.22191629	13.01485366
ENSCAFG00000011782	GFRA1	0	1.022191629	0
ENSCAFG00000015071	ATG9A	256.1472271	129.2152439	326.9567874
ENSCAFG00000007277	IL1RN	192.4727219	98.13039641	60.34159426
ENSCAFG00000001944	TXNRD1	746.1148455	400.6991187	733.5644793
ENSCAFG00000009444	PIWIL2	0	0	0
ENSCAFG00000002026	CYP39A1	119.1745806	149.9248463	344.8699587
ENSCAFG00000004761	LYPD3	6.793154891	37.82109028	28.39604436
ENSCAFG00000045211		0	0	0
ENSCAFG00000015255	SLC35F4	1.132192482	3.066574888	0
ENSCAFG00000014165	GRN	1290.699429	881.1291844	1730.975537
ENSCAFG00000005329	AKR1E2	2808.969547	2829.42643	2459.807343

ENSCAFG00000003776	NRP1	554.7743161	696.1124995	630.6288185
ENSCAFG00000001886		38.14356471	77.91144598	87.68461864
ENSCAFG00000007215	YTHDF3	1220.503495	1116.233259	780.8912199
ENSCAFG00000006378	FAM160A2	151.7137926	123.6851871	156.178244
ENSCAFG00000019662	PIGQ	152.845985	51.10958146	157.3614125
ENSCAFG00000001188	IP6K3	15.85069474	14.31068281	3.549505545
ENSCAFG00000008929	OAS3	20.37946467	57.24273124	29.57921287
ENSCAFG00000030440	CCN3	166.4322948	74.61998894	18.93069624
ENSCAFG00000019272	NCLN	1043.881468	718.6007154	1417.435881
ENSCAFG00000007918		679.4287083	572.2126521	640.8750578
ENSCAFG00000010197	TRPM5	4.528769927	0	0
ENSCAFG00000014774	GPR160	270.5940031	441.5867838	298.1584658
ENSCAFG00000013771	STOX1	46.41989175	39.86547354	29.57921287
ENSCAFG00000019686	DNLZ	181.1507971	154.350936	118.3168515
ENSCAFG00000010242	SLC4A10	0	0	0
ENSCAFG00000013507	CASC4	130.2021354	196.2607928	123.0495256
ENSCAFG00000018597	LAMA1	73.59251132	53.15396472	41.41089802
ENSCAFG00000001502	CARD10	148.3172151	64.39807264	134.8812107
ENSCAFG00000006554	IKBIP	125.6733655	178.8835351	231.9010289
ENSCAFG00000030764	SLC25A29	43.02331431	23.51040747	31.9455499
ENSCAFG00000019519		133.5987129	101.1969713	99.45714537
ENSCAFG00000005174	DNAJB6	195.8692993	194.2164096	163.7978492
ENSCAFG00000001585	FLNC	33.96577445	5.110958146	11.83168515
ENSCAFG00000005979	COG6	84.91443613	175.8169602	121.866357
ENSCAFG00000002509	ERMAP	0	0	0
ENSCAFG00000024883	SOX4	0	2.044383259	2.36633703
ENSCAFG00000005068	KCTD12	36.23015942	20.44383259	33.12871842
ENSCAFG00000043472		0	0	0
ENSCAFG00000003916	KLHL29	16.98288723	4.088766517	5.915842575
ENSCAFG00000002139		0	0	0
ENSCAFG00000015901	FTH1	15215.1385	8399.164623	8647.24625
ENSCAFG00000012330	TMEM206	36.23015942	43.95424006	46.14357208
ENSCAFG00000044440		0	0	0
ENSCAFG00000032043		537.7914288	411.9432266	375.0644192
ENSCAFG00000006076		409.8536784	467.1415746	616.4307963
ENSCAFG00000012361	ASCC2	95.10416847	158.4397025	150.2624014
ENSCAFG00000007219	ASXL1	383.8132513	489.6297904	558.4555391

ENSCAFG00000002018	VPS50	116.6158256	179.9057268	124.2326941
ENSCAFG00000005674	OXSM	71.32812635	110.396696	139.6138848
ENSCAFG00000007948	FDFT1	2699.146877	1491.377587	2145.084518
ENSCAFG00000014079	CCDC141	12.4541173	7.155341405	9.46534812
ENSCAFG00000000615	MPIG6B	5.660962409	2.044383259	9.46534812
ENSCAFG00000006092	PLPP5	235.4960362	256.5700989	262.6634103
ENSCAFG00000009469	NKX2-3	0	0	0
ENSCAFG00000005226	TASOR2	157.374755	215.6824338	307.6238139
ENSCAFG00000024988	FRZB	147.1850226	52.13177309	93.47031268
ENSCAFG00000002463	THADA	87.1788211	89.95286338	121.866357
ENSCAFG00000018253	SHMT1	3402.238408	5998.220481	5346.738519
ENSCAFG00000014322	PCOLCE	226.4384964	52.13177309	82.82179605
ENSCAFG00000004623	SLC4A2	783.2620808	563.2275877	782.0743884
ENSCAFG00000004769	OPTN	388.3420213	376.1665196	262.6634103
ENSCAFG00000019270	PLXNB3	12.4541173	6.133149776	9.46534812
ENSCAFG00000032305	ZNF473	16.98288723	15.33287444	18.93069624
ENSCAFG00000007720	GGCX	696.3663078	1290.005836	2003.34093
ENSCAFG00000009519	CCN5	14.71850226	6.133149776	0
ENSCAFG00000004085	CENPO	14.71850226	9.199724663	16.56435921
ENSCAFG00000005669	ZMYND11	306.8241626	391.499394	446.0545301
ENSCAFG00000005260	SPRY2	30.56919701	16.35506607	9.46534812
ENSCAFG00000020348	GFOD2	20.37946467	25.55479073	22.48020178
ENSCAFG00000001304	ZNF76	150.5816001	144.1290197	194.0396365
ENSCAFG00000006873	ZDHHC2	76.03804708	158.0103821	110.5079393
ENSCAFG00000000986	TBC1D31	15.85069474	100.1747797	92.28714417
ENSCAFG00000009904	DPCD	43.02331431	33.73232377	29.57921287
ENSCAFG00000014008	MYEF2	21.51165715	53.05174556	46.97179004
ENSCAFG00000049619		0	2.044383259	0.130148537
ENSCAFG00000006721	RIMBP2	14.71850226	6.133149776	8.282179605
ENSCAFG00000007900	ELL2	1200.124031	964.948898	673.222885
ENSCAFG00000004893	TMEM37	45.28769927	64.39807264	49.69307763
ENSCAFG00000011217	RBM23	103.0295158	118.574229	131.3317052
ENSCAFG00000032202	C15H4orf46	3.396577445	16.35506607	3.549505545
ENSCAFG00000048382		0	1.022191629	1.183168515
ENSCAFG00000020276	CCN1	552.5099311	290.3024227	3.549505545
ENSCAFG00000014478		86.34099866	14.10624448	32.09936181
ENSCAFG00000003089	RNGTT	67.93154891	128.7961453	100.5693238

ENSCAFG00000016955	KAT7	31.70138949	79.73094708	74.53961644
ENSCAFG00000031778	TRAPPC6A	4.528769927	19.42164096	5.915842575
ENSCAFG00000014864	CASP12	14.71850226	11.24410792	9.46534812
ENSCAFG00000003720	NRCAM	2.264384964	1.022191629	1.183168515
ENSCAFG00000006959	PROM2	0	0	0
ENSCAFG00000002809	HMGN3	280.7837355	223.8599668	265.0297474
ENSCAFG00000045786		15.92994822	2.422594161	6.744060535
ENSCAFG00000007099		0	5.110958146	1.183168515
ENSCAFG00000013990		0	0	0
ENSCAFG00000000071		341.8089102	353.7396352	231.6998903
ENSCAFG00000029988	C18H11orf91	23.77604212	71.55341405	140.7970533
ENSCAFG00000012906	THEM5	0	0	0
ENSCAFG00000007308	DEPDC7	869.523826	2197.712003	1346.44577
ENSCAFG00000013549	YWHAG	178.8864121	212.6158589	253.1980622
ENSCAFG00000016843	HOXB2	4.528769927	9.199724663	7.09901109
ENSCAFG00000014685	SUCO	172.0932572	217.726817	207.0544901
ENSCAFG00000008363	P2RX4	103.0295158	111.4188876	131.3317052
ENSCAFG00000003314	ANXA4	1547.707123	1807.234801	1288.470513
ENSCAFG00000004234	CEP295	190.2083369	81.77533034	50.87624614
ENSCAFG00000042407	S1PR2	6.793154891	15.33287444	44.96040357
ENSCAFG00000002538	SLC3A1	14.71850226	43.95424006	31.9455499
ENSCAFG00000019897	SETX	177.7542196	176.8391519	327.7376786
ENSCAFG00000019920	NUP214	200.3980693	197.1705434	436.589182
ENSCAFG00000010034	RYR2	21.51165715	31.68794051	21.29703327
ENSCAFG00000015054	MMP1	1.132192482	0	0
ENSCAFG00000005134	LMBR1	112.0870557	198.3051761	176.2921087
ENSCAFG00000005091		116.8196203	96.92421029	95.83664971
ENSCAFG00000011848	C18H11orf86	0	0	0
ENSCAFG00000016655	FAM81A	18.11507971	14.31068281	9.46534812
ENSCAFG00000014251	DDX3X	1414.10841	2536.057432	2174.663731
ENSCAFG00000019191	RAX2	0	0	0
ENSCAFG00000005976	NTS	1.132192482	0	0
ENSCAFG00000011104	PDE6D	116.6158256	170.7060021	94.6534812
ENSCAFG00000006917	MAP3K1	172.0932572	330.1678963	288.6931177
ENSCAFG00000010548	DNER	0	0	0
ENSCAFG00000033405		0	0	0
ENSCAFG00000018386	PLD4	0	0	0

ENSCAFG00000031480	RWDD1	658.9360244	676.6908586	660.2080314
ENSCAFG00000004387	RAB38	2.264384964	0	1.183168515
ENSCAFG00000019740	CLSTN1	166.4322948	156.3953193	221.2525123
ENSCAFG00000012104	WASF2	377.0200964	285.1914646	425.9406654
ENSCAFG00000029756	PPP1R1A	1364.291941	758.4661889	997.4110581
ENSCAFG00000047022	FKBPL	65.66716394	53.15396472	63.89109981
ENSCAFG00000000367	ENPP3	14.71850226	8.177533034	7.09901109
ENSCAFG00000018927	SLC9A6	20.37946467	34.7545154	18.93069624
ENSCAFG00000030332	XKR5	0	0	0
ENSCAFG00000019545	CHD5	0	0	0
ENSCAFG00000010061	ABCC11	136.9952903	410.921035	125.4158626
ENSCAFG00000004966	MYD88	394.0029837	528.4730723	605.7822797
ENSCAFG00000008118	ARRDC3	156.2425625	4113.299116	2178.213236
ENSCAFG00000043540		1.132192482	0	3.549505545
ENSCAFG00000002816	MPP6	132.4665204	141.0624448	214.1535012
ENSCAFG00000029233	SKA2	67.93154891	101.1969713	81.63862753
ENSCAFG00000011898	SLC19A1	72.46031883	35.77670702	121.866357
ENSCAFG00000025409	METTL14	54.34523913	130.8405285	102.9356608

NL5	HCC1	HCC2	HCC3	HCC4	HCC5
103.4555226	0	0	1.944902317	0	0
19.60209902	122.5940058	309.1336487	247.0025942	183.3919342	188.3970988
4.356022004	67.2289709	73.68883487	139.0605157	136.501951	108.9525391
76.89467843	2.807798197	1.401885151	1.517023807	0	0
11.58701853	0	0	0	0	0
8.712044008	0	0	0	0	0
46.82723654	0	0	1.944902317	1.041999626	0
7.623038507	30.64850144	64.7023916	96.27266469	83.35997009	119.1668396
1.089005501	29.6598401	147.3776697	128.3635529	62.51997757	85.11917114
22290.8536	674.2670317	252.5190561	40.84294866	847.145696	110.0874613
3.267016503	21.75054941	65.60103592	60.29197182	48.97398243	59.01595865
6.534033006	29.6598401	49.42543803	123.5012971	65.64597645	66.96041463
1.089005501	291.6550944	66.49968025	60.29197182	133.3759521	247.4130574
2.178011002	19.73368028	89.47801571	73.74097135	38.48104619	46.42967055
11.97906051	65.25164823	99.74952038	158.5095388	84.40196972	93.06362711
1.089005501	13.84125872	53.02001534	59.31952067	33.34398804	74.9048706
304.9215403	74.14960026	14.37830924	37.92559518	36.46998691	23.83336792
1.089005501	12.85259738	23.36475252	44.73275329	36.46998691	41.99212443
6.534033006	30.64850144	75.48612353	110.8594321	68.77197532	124.841451
4.356022004	0	0.898644328	0	0	0
399.1749664	7.958723761	5.409838853	35.24162998	10.46167625	50.12951719
2.178011002	10.8752747	50.32408235	38.89804634	29.17598953	30.64290161
3.267016503	16.80724273	50.32408235	55.42971603	29.17598953	46.53181355
0	7.909290694	17.97288655	30.14598591	19.7979929	22.69844564
5.445027505	19.77322674	89.86443277	52.51236256	47.9319828	56.74611409
8.712044008	19.77322674	43.13492773	72.93383688	48.97398243	57.88103637
14.15707151	0	0	0	0	0
0	4.943306684	8.986443277	29.17353475	10.41999626	24.9682902
58126.75762	6339.296492	1690.34998	2635.342639	9569.724566	7207.891412
4.356022004	31.63716278	51.22272668	71.96138573	36.46998691	61.28580322
4.356022004	20.76188807	72.79019055	40.84294866	21.88199215	48.80165812
4.356022004	14.63218778	84.95783474	63.72472441	25.67487079	58.25556073
585.8849596	277.8138356	230.9515922	119.6114925	229.2399177	230.3892232
7.623038507	24.71653342	48.5267937	86.5481531	63.56197719	35.18259074

2.178011002	20.76188807	22.46610819	44.73275329	27.09199028	53.34134725
7.623038507	18.7845654	54.81730399	60.29197182	50.01598205	77.17471516
496.5865085	81.07022962	25.16204118	57.37461835	58.35197906	60.15088094
4.356022004	31.63716278	51.22272668	39.8704975	35.42798729	35.18259074
92.56546759	9.886613368	3.594577311	0.972451158	9.377996635	2.269844564
6.534033006	36.58046946	45.83086071	79.74099499	60.43597831	40.85720215
4.356022004	11.86393604	70.09425756	49.59500908	35.42798729	51.07150268
16.33508252	75.1382616	134.7966492	85.57570194	55.22598018	65.82549235
133.9476766	7.909290694	2.695932983	0	54.18398056	7.944455973
6.534033006	34.60314679	69.19561324	139.0605157	72.93997383	76.03979288
5.445027505	33.61448545	35.94577311	51.5399114	46.88998318	35.18259074
1218.597156	539.8090899	516.7204884	401.6223284	657.5017641	565.1912963
1.089005501	0	0	0	0	0
3.267016503	59.31968021	703.6385086	44.73275329	13.54599514	199.7463216
7.623038507	70.19495491	56.61459265	115.7216879	76.06597271	88.52393798
10.89005501	41.52377615	51.22272668	51.5399114	63.56197719	83.98424886
1191.372018	712.8248238	278.5797416	362.7242821	809.6337095	443.7546122
1.089005501	22.73921075	34.14848445	35.98069286	29.17598953	37.4524353
0	5.931968021	6.290510294	13.61431622	15.62999439	9.079378255
503.1205415	182.9023473	264.2014324	220.746413	277.1719005	268.9765808
33.75917053	0.988661337	0	0.972451158	0	2.269844564
1.089005501	4.943306684	37.74306176	46.67765561	21.88199215	24.9682902
234.1361827	20.76188807	3.594577311	2.917353475	5.209998131	5.674611409
37.3746688	272.2278991	227.339042	171.5112108	145.2443279	168.2862759
1413.52914	292.6437557	83.57392248	346.1926124	287.5918968	255.3575134
2.178011002	0	0	0	0	0
0	94.91148833	39.54035042	21.39392549	30.21798916	43.12704671
30.49215403	194.7662834	216.573283	140.0329668	107.3259615	300.7544047
4.356022004	12.85259738	17.97288655	13.61431622	18.75599327	19.29367879
21.78011002	49.43306684	106.0400307	148.7850272	68.77197532	88.52393798
1011.68611	108.752747	98.85087605	195.4626828	325.1038833	271.2464254
2.178011002	22.73921075	35.94577311	48.62255792	31.25998878	47.66673584
4.356022004	1.977322674	0	0	0	0
28.31414303	4.943306684	0.898644328	0	1.041999626	1.134922282
0	0	0	0	0	0
3.267016503	22.73921075	35.94577311	45.70520445	15.62999439	21.56352335
47.91624205	141.3785712	203.9922624	170.1789527	140.6699495	477.8022806

3.267016503	0	0	0	1.041999626	0
3020.90126	1257.57722	781.8205651	997.7348886	1507.773459	979.4379292
2697.466626	61.29700288	23.36475252	262.5618128	312.5998878	86.25409342
0	7.909290694	17.07424223	17.50412085	14.58799477	10.21430054
22.86911552	4.943306684	0	0.972451158	0	1.134922282
0	13.84125872	90.7630771	44.73275329	39.59598579	30.64290161
646.8692676	111.7187311	70.99290189	128.3635529	181.3079349	99.8731608
1.089005501	0	0	0	1.041999626	2.269844564
4.356022004	10.8752747	21.56746387	21.39392549	8.335997009	15.88891195
0	2.96598401	35.04712878	32.09088823	11.46199589	26.10321248
363.7278373	165.1064432	41.33763908	105.0247251	126.0819548	123.7065287
15.24607701	110.7300697	210.2827727	686.5505179	236.5339151	128.2462178
1.089005501	14.82992005	40.43899475	57.37461835	39.59598579	29.50797933
7897.467893	4319.461381	3790.481774	1675.533346	3889.784604	2783.964357
15146.97751	2201.748797	1913.213774	3508.60378	2231.963199	955.6045613
1.089005501	11.86393604	41.33763908	71.96138573	20.83999252	38.58735758
128.5026491	213.5508488	297.4512725	458.9969468	343.8598766	322.317928
2903.288666	215.5281714	529.301509	211.0219014	220.9039207	299.6194824
282.0524248	40.53511481	16.1755979	36.95314402	81.27597084	37.4524353
2.178011002	14.82992005	18.87153088	38.89804634	26.04999065	26.10321248
1.089005501	12.85259738	13.47966492	18.47657201	7.293997383	19.29367879
128.5026491	0.988661337	0	2.917353475	11.46199589	1.134922282
9.801049509	27.68251743	31.45255147	45.70520445	38.55398617	37.4524353
10.89005501	24.71653342	53.91865966	47.65010676	37.51198654	32.91274617
7.623038507	37.5691308	23.36475252	52.51236256	23.9659914	20.42860107
689.3404821	25.70519476	29.65526282	47.65010676	79.19197159	47.66673584
9772.735366	3173.602891	615.5713645	1617.186277	2523.723094	1394.819484
1.089005501	0	0.898644328	0	0	0
3.267016503	13.84125872	30.55390714	38.89804634	23.9659914	34.04766845
13.06806601	0	0	0.972451158	0	1.134922282
40.29320354	91.94550432	130.3034275	156.5646365	91.6959671	90.79378255
64.25132456	189.8229767	239.9380355	257.699557	187.5599327	204.2860107
2.178011002	20.76188807	53.91865966	35.98069286	41.67998504	94.19854939
3.267016503	19.77322674	25.16204118	22.36637664	10.41999626	22.69844564
214.5340837	109.7414084	75.48612353	88.49305542	84.40196972	96.46839396
8.712044008	0	0	0	0	0
13.06806601	0.988661337	0	0	0	1.134922282

21.78011002	5.931968021	1.797288655	0	0	0
0	7.909290694	8.986443277	11.6694139	5.209998131	6.809533691
56.62828605	121.6053444	262.4041437	172.123855	86.48596897	107.8176168
10104.88204	3369.357836	3687.137677	2493.36477	5073.49618	6274.985296
100.1885061	3.954645347	1.797288655	7.779609268	23.9659914	4.539689127
4.356022004	7.909290694	33.24984013	18.47657201	20.83999252	36.31751302
5.445027505	16.80724273	26.95932983	59.31952067	37.51198654	26.10321248
555.3928055	345.0428065	178.8302212	163.3717946	230.2819174	284.8654927
43.56022004	87.99085898	112.330541	187.6830736	115.6619585	69.23025919
156.8167921	33.61448545	60.20916996	36.95314402	59.39397869	57.88103637
2.178011002	0.988661337	0	0	0	0
2292.35658	452.8068923	255.2149891	132.2533576	319.8938852	175.9129537
2.178011002	9.886613368	20.66881954	22.36637664	9.377996635	23.83336792
2379.47702	1029.196452	955.2589204	291.7353475	1158.703584	1810.20104
1433.131239	44.48976016	62.00645861	64.18177646	26.04999065	13.61906738
5.445027505	0	0	0	0	1.134922282
2.178011002	3.954645347	7.189154622	14.58676738	9.377996635	14.75398966
3.267016503	0	0.898644328	0	0	0
127.4136436	291.6550944	358.5590868	253.8097524	175.0559372	312.1036275
2.178011002	8.897952031	11.68237626	24.31127896	25.00799103	15.88891195
14.15707151	21.75054941	63.80374727	164.3442458	33.30230805	55.61119181
20.69110452	45.47842149	33.24984013	104.052274	85.44396934	62.4207255
0	1.977322674	9.885087605	7.779609268	12.50399551	9.079378255
28.31414303	1.482992005	0.898644328	0	0	0
3.267016503	10.8752747	25.16204118	19.44902317	9.377996635	53.34134725
0	17.79590406	8.986443277	22.36637664	10.41999626	14.75398966
1.089005501	7.909290694	23.36475252	41.81539981	9.377996635	11.34922282
218.8901057	37.5691308	8.08779895	7.779609268	42.72198467	21.56352335
23.45717849	46.98118673	44.32113824	90.64217248	57.60173933	82.74718357
42.47121454	3.954645347	0	1.944902317	8.335997009	0
71.87436307	2.96598401	0	0	4.167998504	0
190.5759627	38.55779214	26.0606855	28.2010836	98.98996448	56.74611409
8.712044008	33.61448545	70.09425756	47.65010676	59.39397869	255.3575134
65.34033006	16.80724273	0	0	2.083999252	10.21430054
27.22513753	165.1064432	145.9488253	163.8385712	186.5179331	181.4513744
40.29320354	2.96598401	0.898644328	0	2.083999252	0
17.42408802	89.96818165	26.95932983	58.34706951	66.68797607	64.69057006

25.04712652	1.977322674	1.797288655	0	0	0
141.5707151	2.96598401	0	0	13.54599514	11.34922282
13.06806601	27.68251743	47.62814937	37.92559518	33.34398804	21.56352335
2.178011002	135.3378504	59.31052563	83.63079963	26.04999065	53.34134725
1105.340584	29.6598401	115.0264739	0.972451158	32.30198841	54.47626953
14.15707151	1.977322674	0	0	2.083999252	0
50.09425305	80.08156828	268.694654	167.2615993	102.1159634	212.2304667
0	17.79590406	4.493221639	3.889804634	8.335997009	22.69844564
1694.49256	644.6071916	833.0432918	483.3082258	789.8357166	573.1357523
58.80629706	365.8046946	414.2750351	619.4513879	267.7939039	324.5877726
30.49215403	44.48976016	53.02001534	52.51236256	65.64597645	52.20642496
3.267016503	1.977322674	1.797288655	0	1.041999626	1.134922282
2.178011002	3.954645347	32.3511958	47.65010676	6.251997757	21.56352335
42.01383223	4.943306684	0	2.917353475	20.83999252	1.134922282
2720.335742	20.76188807	169.8437779	116.694139	38.55398617	7.944455973
35.93718153	97.87747234	77.28341218	135.170711	77.10797233	80.57948201
131.7696656	12.85259738	3.594577311	12.64186506	5.209998131	4.539689127
94.74347859	175.981718	171.6410666	173.0963062	224.0299196	189.5320211
2.178011002	11.86393604	16.1755979	22.36637664	21.88199215	41.99212443
116.5235886	37.5691308	24.26339685	0	4.167998504	7.944455973
8.766494283	0	0	0	1.698459391	0
0	54.37637352	224.6610819	238.2213603	56.26797981	35.18259074
990.9950059	3065.838805	4502.208082	2857.061504	1738.055376	3543.227364
11.97906051	0.988661337	1.797288655	0	0	0
26.13613202	2.96598401	3.594577311	1.944902317	0	3.404766845
11.97906051	4.943306684	7.189154622	0	0	0
313.6335843	1592.733414	1981.510743	1606.489314	1298.331534	1038.453888
1.089005501	1.977322674	12.58102059	16.53166969	20.83999252	4.539689127
26.13613202	272.870529	207.5868397	131.2809064	69.81397495	108.9525391
7.623038507	15.81858139	26.0606855	35.0082417	12.50399551	14.75398966
326.3858387	157.8397824	78.01131409	92.83018759	222.6440601	110.3030966
0	4.943306684	11.68237626	4.862255792	11.46199589	17.02383423
504.209547	1136.960537	1169.13627	1356.569366	845.0616968	1821.538913
52.27226405	25.70519476	15.27695357	0.972451158	4.167998504	6.809533691
427.9791619	109.7414084	250.7217674	100.1624693	174.0139376	213.365389
17.42408802	174.0043953	87.16849979	155.5921854	62.51997757	77.17471516
726.3666692	1159.699748	3029.330029	1445.062421	1094.099607	1209.827152

5.445027505	0.988661337	0	0	1.041999626	0
2755.183918	66.24030957	29.65526282	14.58676738	121.9139563	301.889327
0	2.96598401	1.797288655	4.862255792	3.125998878	3.404766845
13.06806601	1.977322674	0	5.834706951	0	1.134922282
25.04712652	97.87747234	117.7224069	94.32776237	59.39397869	93.06362711
119.7906051	325.2695798	430.450633	553.3247092	314.6838871	212.2304667
356.1047988	934.2849633	1036.13691	2086.880186	987.8156456	619.6675659
83.85342358	154.2311685	149.1749584	143.9227715	157.3419435	137.3255961
6.534033006	0	0	0	0	0
2.178011002	1.977322674	9.885087605	18.47657201	9.377996635	6.809533691
39.20419804	120.6166831	88.96578845	72.93383688	129.2079536	155.4843526
545.591756	208.6075421	214.7759943	169.2065016	210.4839245	239.4686015
1.089005501	0	0	0	0	1.134922282
15.24607701	60.30834155	212.0800613	167.2615993	119.829957	73.76994832
57.71729155	101.8321177	172.5397109	133.2258087	73.98197345	60.15088094
39.81404112	68.24729208	84.00527176	154.2988253	95.17624585	86.87830067
18.51309352	0	0	0	10.41999626	0
1.089005501	0	0	0.972451158	0	0
2.853194413	0	0	0	0	0
133.9476766	19.77322674	56.61459265	24.31127896	51.05798168	54.47626953
98.01049509	262.9839156	264.2014324	198.3800363	218.8199215	167.9684977
563.015844	107.7640857	106.938675	126.4186506	33.34398804	71.50010376
202.5550232	430.0676815	328.0051796	507.6195047	370.9518669	514.1197937
0	0	0	0	0	0
1940.607803	570.4575913	638.0374727	425.9336074	1330.633523	547.0325398
227.6021497	1337.658789	7988.049429	1543.279988	1142.03159	1815.875651
83.85342358	120.6166831	624.5578078	166.2891481	170.8879387	590.1595865
808.0420818	198.7209287	505.0381122	299.5149568	283.4238983	180.4526428
7.623038507	0	0	0	0	0
1812.105154	677.2330157	375.633329	921.8836982	584.5617903	518.6594828
4.356022004	4.943306684	19.77017521	22.36637664	10.41999626	7.944455973
15.24607701	19.77322674	33.24984013	134.1982599	55.22598018	125.9763733
5185.844196	2189.884861	1789.200857	1865.161322	2046.487266	3400.227156
1.089005501	7.909290694	18.87153088	20.42147433	15.62999439	5.674611409
5.445027505	9.886613368	53.91865966	69.04403225	31.25998878	41.99212443
176.4188912	105.786763	179.7288655	97.24511585	104.1999626	94.19854939
2.178011002	3.954645347	14.37830924	20.42147433	3.125998878	12.4841451

1424.419195	432.0450042	405.2885918	1162.079134	290.7178957	246.2781352
1.089005501	2.96598401	4.493221639	7.779609268	12.50399551	7.944455973
3.267016503	2.96598401	8.986443277	11.6694139	9.377996635	17.02383423
10.89005501	43.50109882	33.24984013	29.17353475	26.04999065	43.12704671
3.267016503	3.954645347	11.68237626	15.55921854	18.75599327	18.15875651
6.534033006	0	0	0	0	0
50515.69818	6069.391947	6640.981582	4872.952755	4895.314244	3973.362909
936.5447309	2220.533362	4831.111906	6945.246174	2503.925102	8421.123331
61.13676883	95.03012769	402.6555639	285.6283543	170.3148389	150.309107
2.178011002	22.73921075	43.13492773	48.62255792	20.83999252	13.61906738
6.534033006	87.99085898	35.94577311	30.14598591	51.05798168	133.9208293
6.534033006	0	0	0	0	0
10.89005501	25.70519476	16.1755979	29.17353475	32.30198841	40.85720215
1314.45142	695.0585796	641.083877	326.7046912	707.830346	1429.49136
5.445027505	1.977322674	0	0	0	0
3.267016503	2.96598401	53.02001534	61.26442298	12.50399551	27.23813476
106.1998165	61.67269419	60.20916996	31.37127437	85.44396934	52.20642496
45.89069181	142.6539443	158.3591034	307.7321691	104.3458426	207.100618
76.23038507	129.5146351	136.5939378	309.2394684	126.0819548	175.9129537
535.7907065	224.4261235	131.2020718	282.010836	197.979929	141.8652852
22.86911552	74.14960026	80.8779895	71.96138573	42.72198467	37.4524353
5.445027505	0.988661337	0	0	0	0
64.63247649	156.3864503	82.50453573	56.01318673	157.8629434	58.44849751
29.40314853	4.943306684	5.391865966	7.779609268	2.083999252	7.944455973
23.95812102	8.897952031	5.391865966	0	0	1.134922282
352.8377823	570.4575913	1010.022306	1003.501524	473.0678303	551.572229
1357.98986	2841.412682	5286.72458	3575.70291	3097.864888	2920.155031
4.356022004	6.920629358	23.36475252	17.50412085	12.50399551	23.83336792
47.91624205	32.62582411	6.290510294	16.53166969	16.67199402	21.56352335
3711.330747	1596.688059	2013.861938	922.8561494	1981.883289	1361.906738
13.06806601	24.71653342	25.16204118	45.70520445	34.38598766	34.04766845
87.12044008	30.64850144	9.885087605	2.917353475	1.041999626	9.079378255
771.0158947	159.1744752	151.8708914	128.3635529	230.2819174	137.3255961
58.80629706	7.909290694	1.797288655	3.889804634	12.50399551	10.21430054
3.267016503	8.897952031	1.797288655	14.58676738	10.41999626	19.29367879
1.089005501	0.988661337	0	0	0	0
457.3823104	123.5826671	35.94577311	324.7986869	78.14997196	30.64290161

401.8430299	180.9250246	75.48612353	154.6197342	177.1399364	233.7939901
247.2042487	315.3829664	646.1252716	605.8370717	440.7658418	453.9689127
4.356022004	34.60314679	125.8102059	108.9145297	19.7979929	18.15875651
7.623038507	26.69385609	210.2827727	164.3442458	47.9319828	57.88103637
2.178011002	32.62582411	251.6204118	3.889804634	31.25998878	105.5477722
2.178011002	11.86393604	10.78373193	16.53166969	14.58799477	10.21430054
283.1414303	76.12692293	97.95223172	102.1073716	116.7039581	108.9525391
127.4136436	45.47842149	8.986443277	16.53166969	37.51198654	39.72227986
132.8586711	423.1470522	381.9238393	265.4791663	236.5339151	188.3970988
78.40839607	405.3511481	119.5196956	266.4516174	202.1479275	104.4128499
295.1204908	569.46893	920.2117916	712.8066991	419.9258493	515.2547159
37.02618703	10.8752747	1.797288655	0.972451158	7.293997383	1.134922282
10.89005501	25.70519476	45.83086071	55.42971603	62.51997757	57.88103637
218.8901057	627.7999489	656.0103592	617.5064856	520.9998131	528.8737833
26.13613202	10.8752747	11.68237626	2.917353475	7.293997383	6.809533691
5.445027505	66.24030957	131.2020718	890.7652611	46.88998318	99.8731608
1.089005501	9.886613368	10.78373193	5.834706951	10.41999626	12.4841451
3.267016503	0.988661337	0	0	1.041999626	0
1446.199305	742.4846639	317.2214477	212.9668037	524.1258119	912.4775146
246.1152432	471.5914577	860.901266	654.4596296	937.7996635	804.6598978
9648.588739	7973.553681	4830.213262	3988.994652	4854.676258	3752.053064
2.178011002	12.85259738	11.68237626	21.39392549	9.377996635	4.539689127
32.67016503	84.03621363	102.4454534	46.67765561	50.01598205	69.23025919
877.7384338	3013.439755	3316.896214	4636.647124	3300.012816	1687.629433
301.6545238	430.0676815	2516.204118	2185.097753	358.4478714	852.3266337
1183.74898	1403.642046	2007.571428	3026.268005	1802.659353	1765.939071
3.267016503	15.81858139	122.2156286	26.25618128	18.75599327	17.02383423
0	0.988661337	0	0.972451158	0	0
693.6965042	375.691308	405.2885918	465.8041049	376.161865	495.9610372
555.3928055	57.34235754	3.594577311	31.11843707	155.2579443	48.80165812
18.51309352	6.920629358	6.290510294	2.917353475	6.251997757	2.269844564
2689.843588	498.2853138	1221.257641	993.8450839	553.3018015	601.5088094
3.267016503	22.73921075	485.267937	44.73275329	14.58799477	32.91274617
166.6178417	1.977322674	0	0	3.125998878	0
2.178011002	0	0	0	0	0
792.7960047	219.4828168	442.1330092	270.3414221	305.3058905	473.2625915
4.356022004	53.38771219	239.0393912	973.4236096	2.083999252	358.6354411

17737.7216	7293.354682	3448.99693	8751.087975	11225.46197	5784.69887
200.3770122	533.8771219	835.7392248	405.5121331	658.5437637	473.2625915
11.97906051	15.81858139	63.80374727	23.3388278	54.18398056	52.20642496
31.58115953	5.931968021	0	1.944902317	1.041999626	1.134922282
56.62828605	180.9250246	134.7966492	84.60325079	103.157963	125.9763733
37.02618703	44.48976016	0	8.752060426	21.88199215	2.269844564
3.267016503	12.85259738	9.885087605	3.889804634	4.167998504	4.539689127
29.40314853	193.777622	47.62814937	47.65010676	89.61196785	53.34134725
45.73823104	109.7414084	129.4047832	156.5646365	146.9219473	112.3573059
125.2356326	340.0994999	81.77663382	571.8012812	451.1858381	318.9131612
119.7906051	290.666433	363.9509527	339.3854543	209.4419249	207.6907776
2034.262276	1246.701946	445.7275866	727.3934665	1352.515515	380.1989644
619.4807793	347.6330992	196.9199315	198.4578324	359.2710511	44.64784257
202.5550232	282.7571423	659.6049366	660.2943366	377.2038647	209.9606221
548.8587725	1781.567729	6089.213965	2042.147433	1126.401596	2449.162284
0	0.988661337	2.695932983	1.944902317	2.083999252	4.539689127
3.267016503	6.920629358	10.78373193	11.6694139	18.75599327	5.674611409
9.801049509	112.7073924	73.68883487	140.0329668	42.72198467	255.3575134
47.91624205	0	0	0	6.251997757	0
1.089005501	11.86393604	7.189154622	7.779609268	1.041999626	6.809533691
25.04712652	7.909290694	18.87153088	0.972451158	14.58799477	6.809533691
448.6702664	252.1086409	258.8095664	180.8759155	348.0278751	219.0400004
4.356022004	0.988661337	0	0	0	0
53.36126955	82.05889096	125.8102059	105.9971763	105.2419622	149.8097412
306.0105458	687.1196291	882.4687298	735.1730758	403.2538553	256.4924357
15.24607701	40.53511481	37.74306176	50.56746024	28.13398991	37.4524353
111.5903937	167.4990037	236.1906887	378.0306633	153.9762848	268.3410243
3.267016503	3.954645347	8.986443277	4.862255792	8.335997009	7.944455973
370.2618703	119.6280218	111.4318966	90.43795774	177.1399364	202.0161662
3.267016503	6.920629358	8.986443277	13.61431622	14.58799477	9.079378255
45.73823104	33.61448545	30.55390714	16.53166969	16.67199402	18.15875651
5.869739651	22.06692104	81.09366413	62.41191535	35.16748738	36.18132234
141.5707151	228.3807688	421.4641897	304.3772126	197.979929	340.4766845
4.356022004	0	0	0	1.041999626	0
273.3403808	430.0676815	932.7928122	693.357676	466.8158325	545.8976176
6.534033006	12.85259738	14.37830924	21.39392549	15.62999439	7.944455973
0	0	0	0	0	0

23.95812102	0.988661337	0	1.944902317	0	0
201.4660177	44.48976016	31.45255147	9.724511585	31.25998878	20.42860107
384.4189419	1039.083065	1127.798631	1137.767855	779.4157203	2120.034822
934.3558298	370.7084549	201.2603836	446.3161837	801.2560325	535.6719678
876.6494283	26.69385609	12.58102059	236.3056315	52.09998131	28.37305705
42.47121454	26.69385609	0	0.972451158	11.46199589	0
12184.38161	4250.215541	3635.807113	184.7657201	4005.967563	7878.335401
64.25132456	137.4239258	390.9102826	119.6114925	221.9459204	241.738446
2.178011002	9.886613368	1135.88643	50.56746024	8.335997009	136.1906738
224.3351332	94.91148833	121.3169842	77.79609268	166.7199402	128.2462178
111.0785611	2.96598401	20.66881954	0	4.167998504	1.134922282
1.089005501	10.8752747	27.85797416	20.42147433	5.209998131	19.29367879
62.07331356	6.920629358	3.594577311	5.834706951	7.293997383	2.269844564
16.33508252	63.27432556	154.5668244	92.38286005	31.25998878	106.6826945
7.623038507	50.42172818	315.424159	24.31127896	18.75599327	44.26196899
19.60209902	8.897952031	1.797288655	4.862255792	6.251997757	4.539689127
0	1.977322674	3.594577311	2.917353475	14.58799477	20.42860107
70.78535757	16.80724273	5.391865966	9.724511585	14.58799477	20.42860107
0	2.96598401	1.797288655	29.17353475	2.083999252	11.34922282
38.11519254	17.79590406	0.898644328	8.752060426	15.62999439	4.539689127
123.0576216	19.77322674	0	27.22863244	18.75599327	1.134922282
34.84817603	88.97952031	96.15494307	85.57570194	45.84798355	63.55564778
3.267016503	5.931968021	13.47966492	17.50412085	10.41999626	20.42860107
236.3141937	423.1470522	717.1181735	681.6882621	428.2618463	383.6037313
433.4241894	177.9590406	125.8102059	62.23687414	153.173945	154.3494303
4120.796816	1244.724623	2298.73219	1524.803416	4219.056486	1398.224251
29.40314853	34.60314679	115.9251183	81.68589731	69.81397495	61.28580322
4542.241945	2680.260884	2441.616638	1875.858285	3089.528891	2408.305082
4.356022004	20.76188807	63.80374727	33.06333939	31.25998878	28.37305705
23.95812102	69.20629358	109.634608	61.26442298	88.56996822	38.58735758
8.712044008	177.9590406	2765.128596	1112.484125	100.0319641	138.4605184
15.24607701	33.61448545	30.55390714	46.67765561	39.59598579	19.29367879
148.1047481	13.84125872	8.986443277	21.39392549	13.54599514	22.69844564
41.38220904	49.43306684	131.2020718	81.68589731	46.88998318	73.76994832
14.15707151	18.7845654	33.24984013	28.2010836	22.92399177	30.64290161
5.445027505	0.988661337	0	1.944902317	1.041999626	0
546.6807615	278.802497	166.2492006	269.3689709	225.0719192	160.0240417

1413.52914	488.3987004	396.3021485	670.0188482	791.9197159	742.2391723
2.178011002	22.73921075	11.68237626	9.724511585	7.293997383	7.944455973
160.3342799	292.6437557	761.1517456	438.7018911	161.509942	273.0282533
28.31414303	13.84125872	1.797288655	2.917353475	2.083999252	9.079378255
26.13613202	84.03621363	60.20916996	63.2093253	91.6959671	112.3573059
57.71729155	507.1832658	230.9515922	163.3717946	145.8799477	404.0323323
8.712044008	14.82992005	54.81730399	26.25618128	22.92399177	34.04766845
36.9608467	18.35944102	1.653505563	2.752036778	10.20117634	0
25.04712652	7.909290694	0	0	0	0
2859.728446	325.2695798	74.5874792	306.3221149	910.7076732	324.5877726
0	30.64850144	15.27695357	11.6694139	2.083999252	7.944455973
56.62828605	269.9045449	248.9244788	196.435134	109.4099607	128.2462178
680.6284381	352.9520972	348.6739992	430.7958632	426.1778471	465.3181355
8.712044008	47.24812529	199.4361357	35.0082417	53.14198093	60.15088094
386.5969529	594.1854634	1428.844481	1000.652242	544.9658045	752.4534729
1307.895607	321.3149345	117.7224069	358.8344775	655.4177648	308.6988607
3.267016503	10.8752747	7.189154622	3.889804634	7.293997383	3.404766845
1.089005501	0	0	0	0	1.134922282
1.089005501	11.86393604	17.97288655	15.55921854	10.41999626	19.29367879
2.178011002	0.988661337	0	0	0	0
2.570052982	0	0	0	0	0
118.7015996	7234.035001	2298.73219	3184.777544	557.4698	516.3896382
75.14137957	26.69385609	11.68237626	37.92559518	10.41999626	7.944455973
161.1728142	141.3785712	38.64170609	63.2093253	119.829957	140.7303629
1.089005501	0	0	0	0	0
0	0	0	0	0	0
5.445027505	3.954645347	15.27695357	53.48481372	27.09199028	152.0795858
467.1833599	112.7073924	38.64170609	35.0082417	13.54599514	26.10321248
2159.497909	874.9652831	458.3086071	1140.685209	1611.973422	752.4534729
1.089005501	8.897952031	10.78373193	20.42147433	21.88199215	15.88891195
35.93718153	39.54645347	27.85797416	63.2093253	52.09998131	82.84932657
418.1781124	1320.851546	1187.109157	2090.769991	1556.747441	1115.628603
0	0	0	0	0	0
2352.251882	6578.552535	4502.208082	3955.931313	4354.516438	5859.603741
186.2199407	1200.234863	793.5029414	975.3685119	997.1936422	292.8099487
1.089005501	11.86393604	21.56746387	44.73275329	16.67199402	17.02383423
613.1100971	341.0881612	136.5939378	414.2641935	271.9619024	259.8972025

1.089005501	5.931968021	3.594577311	5.834706951	1.041999626	2.269844564
123.0576216	63.27432556	14.37830924	3.889804634	7.293997383	9.079378255
1568.167921	759.2919067	1014.569446	897.5724193	1083.679611	1307.430469
84.94242908	158.1858139	243.5326128	234.3607292	351.153874	166.8335754
40.29320354	87.99085898	122.2156286	52.51236256	62.51997757	77.17471516
2.178011002	0	0	0	0	0
88.20944558	174.9930566	559.8554162	344.2477101	126.0819548	330.262384
130.6806601	422.1583908	513.1259111	778.9333779	307.3898897	517.5245605
6.534033006	0	0.898644328	0	0	0
406.1990519	845.305443	806.083962	1187.362864	640.8297701	725.2153381
3.267016503	17.79590406	23.36475252	42.78785097	43.7639843	6.832232137
95.83248409	200.6982514	317.2214477	317.9915288	130.2499533	244.0082906
129.5916546	313.4056438	573.3350811	663.2116901	327.1878826	286.000415
7.623038507	25.70519476	24.26339685	36.95314402	25.00799103	19.29367879
192.7539737	172.0270726	106.0400307	121.5563948	90.65396747	113.4922282
3.267016503	9.886613368	28.75661849	14.58676738	10.41999626	29.50797933
17.42408802	38.55779214	70.09425756	40.84294866	52.09998131	72.63502604
38.11519254	85.02487497	85.37121113	121.5563948	119.829957	78.30963745
254.8272872	213.5508488	62.00645861	75.85119036	131.2919529	118.0319173
0	4.943306684	10.78373193	8.752060426	5.209998131	4.539689127
43.56022004	128.5259738	434.9438546	183.7932689	73.98197345	29.50797933
74.05237407	363.8273719	975.9277399	127.3911018	475.1518295	2232.392128
136.1256876	48.4444055	18.87153088	38.89804634	57.30997944	52.20642496
33.75917053	131.4919578	187.8166645	398.704975	138.5859503	140.7303629
0	1.977322674	4.493221639	7.779609268	5.209998131	9.079378255
2721.424747	563.536962	904.0361937	1203.894534	1631.771415	860.2710896
4609.760286	2253.159187	1842.220872	1887.527699	3098.906888	4120.902805
2238.99531	1334.692805	588.6120347	1479.098212	2585.201072	990.787152
1.089005501	0	0	0	0	0
3239.791366	660.425773	540.9838853	1072.613628	993.0256437	718.4058044
336.5026998	189.8229767	128.5061389	169.2065016	285.5078976	200.8812439
215.6230892	277.8138356	330.7011126	452.1897887	326.145883	329.1274617
89.29845108	65.25164823	48.5267937	51.5399114	44.80598392	76.03979288
14.15707151	3.954645347	0	0	0	0
58.51226557	95.90014967	80.80609795	107.9420786	75.02397308	124.7960541
1327.497706	2600.179316	3080.552755	3373.433069	2238.215197	2273.24933
13.06806601	33.61448545	234.5461695	498.8674443	18.75599327	14.75398966

116.5235886	38.55779214	16.1755979	35.98069286	65.64597645	26.10321248
35.93718153	168.0724273	2756.142153	182.8208178	80.23397121	156.6192749
11.97906051	20.76188807	139.2898708	26.25618128	14.58799477	18.15875651
594.5970036	341.0881612	294.7553395	275.2036778	350.1118744	384.7386535
59.89530256	246.1766729	318.120092	109.8869809	126.0819548	157.7541972
1605.194109	794.8837148	787.2124311	645.7075692	1061.797619	1425.462386
62.07331356	4.943306684	13.47966492	12.64186506	29.17598953	21.56352335
1.785969022	0.988661337	0	0	0	0
25.04712652	15.81858139	60.20916996	35.0082417	28.13398991	78.30963745
0	0	0	0	0	0
2.178011002	0	0	0	0	1.134922282
6.534033006	278.802497	1948.260903	6.807158109	40.63798542	704.786737
2151.87487	6818.79724	7584.558126	8580.909022	6423.927695	5889.11172
2.178011002	21.75054941	8.08779895	15.55921854	22.92399177	10.21430054
584.7959541	1727.191355	2240.320309	1533.555477	1293.121536	1408.438552
32.67016503	73.16093892	142.8844481	59.31952067	66.68797607	37.4524353
22.86911552	207.6188807	130.3034275	97.24511585	78.14997196	85.11917114
8762.062031	3826.959736	2431.138446	1991.521625	2878.544807	2999.191019
3.267016503	30.64850144	6.290510294	112.8043344	4.167998504	2.269844564
1.089005501	0	0	0	0	1.134922282
376.7959034	934.2849633	1535.783156	926.745954	687.7197532	643.5009338
288.5864578	459.7275216	916.6172143	776.9884756	611.6537805	362.0402079
2.178011002	6.920629358	14.37830924	17.50412085	19.7979929	13.61906738
64.25132456	82.05889096	185.1207315	148.7850272	75.02397308	103.2779276
15.24607701	32.62582411	39.54035042	65.15422762	22.92399177	30.65425083
1633.464691	558.6430884	514.0515148	635.4287605	770.8504834	1109.783753
2009.215149	4152.377615	4786.179689	6990.951378	5513.220022	4620.268609
54.45027505	122.5940058	114.1278296	102.1073716	68.77197532	204.2860107
14.15707151	25.70519476	99.74952038	67.09912993	45.84798355	27.23813476
15.24607701	36.58046946	42.2362834	41.81539981	31.25998878	20.42860107
51.18325855	10.8752747	0	0	0	0
67.51834106	15.81858139	17.07424223	20.42147433	36.46998691	18.15875651
38.11519254	80.08156828	54.81730399	108.9145297	53.14198093	54.47626953
303.8325348	584.2988501	821.3609155	611.6717787	576.2257932	762.6677734
115.4345831	164.1177819	160.8573347	332.5782962	303.2218912	153.214508
8.712044008	95.90014967	13.47966492	42.78785097	61.47797794	30.64290161
17.42408802	10.8752747	2.695932983	0.972451158	1.041999626	0

7.623038507	28.67117877	35.04712878	37.92559518	19.7979929	19.29367879
3914.974776	10764.54464	12357.25815	8808.462593	9069.564746	14024.23464
46.13027302	74.29789946	163.6521185	259.2068563	241.4417334	117.4304085
94.74347859	239.2560435	232.7488809	270.3414221	327.1878826	509.5801045
2.178011002	10.66765582	13.41675981	1.944902317	6.251997757	4.539689127
51.18325855	96.88881101	338.7889116	65.15422762	71.8979742	197.476477
102.3665171	32.62582411	0.898644328	2.917353475	1.041999626	4.539689127
15.58366872	26.43680415	43.75499232	60.11693062	42.80534464	24.4689244
390.9529749	1690.610886	4013.345568	867.4264333	617.9057783	2294.812854
21.78011002	2.96598401	2.695932983	0.972451158	5.209998131	6.809533691
9.801049509	23.72787208	53.91865966	36.95314402	17.71399364	12.4841451
316.9006008	636.6979009	750.3680137	1266.131408	753.3657297	780.8265299
92.56546759	155.2198299	352.2685765	152.6748319	113.5779592	130.5160624
271.1623698	422.1583908	434.9438546	357.8620263	514.7478153	545.8976176
182.9529242	423.1470522	829.4487145	685.5780667	271.9619024	292.8099487
9.801049509	14.82992005	16.1755979	16.53166969	15.62999439	11.34922282
2238.99531	4635.833008	5825.911177	4265.170781	5616.377985	5176.380527
2.178011002	3.954645347	8.08779895	9.724511585	9.377996635	10.21430054
6.534033006	13.84125872	24.26339685	28.2010836	14.58799477	38.58735758
6.534033006	0	0	0	2.083999252	0
1.089005501	1.977322674	0	0	1.041999626	0
20.69110452	266.9385609	151.8708914	163.3717946	15.62999439	506.1753377
411.6440794	1246.701946	2516.204118	1215.563948	1253.52555	1757.994615
861.4033513	354.9294199	348.6739992	370.5038914	635.6197719	358.6354411
454.1152939	491.3646844	1355.155646	1773.750913	611.6537805	1503.772023
87.12044008	175.981718	242.6339685	256.7271058	151.0899458	153.214508
3.267016503	4.943306684	10.78373193	14.58676738	7.293997383	14.75398966
22.86911552	140.3899098	116.8237626	111.8318832	36.46998691	74.9048706
33.75917053	153.2425072	82.67527815	77.79609268	91.6959671	98.73823852
52.27226405	20.76188807	14.37830924	10.69696274	34.38598766	29.50797933
29.40314853	102.820779	153.66818	114.7492367	79.19197159	24.9682902
77.31939057	420.1810681	478.9774267	186.7106224	289.6758961	255.3121165
0	7.909290694	13.47966492	8.752060426	5.209998131	6.809533691
16221.82594	9977.570211	11077.58863	3136.154986	8783.014849	7139.796075
4011.896266	2773.19505	328.9038239	3000.011824	2072.537256	868.2155456
712.2095977	186.8569927	204.8909067	151.7023807	276.1299009	114.6271505
277.6964028	265.9498996	53.02001534	69.04403225	212.5679237	49.9365804

65.34033006	161.1517979	189.6139532	144.8952226	114.6199589	197.476477
0	1.977322674	8.986443277	1.944902317	2.083999252	5.674611409
0	0	0	0	0	1.134922282
3.267016503	63.27432556	3249.497889	1623.993435	0	1.134922282
246.0499029	360.8613879	368.417215	402.5753306	316.6636864	472.014177
220.1533521	82.23685	4.933557359	36.61278612	12.21223562	66.32485815
202.5550232	116.6620377	96.15494307	93.35531121	107.3259615	116.896995
8944.00218	3802.391501	1003.785714	4261.280976	3203.106851	4313.839593
2.178011002	13.84125872	8.08779895	14.58676738	15.62999439	3.404766845
164.4398307	209.5962034	288.4648292	306.3221149	190.6859316	223.5796895
38.11519254	58.33101887	57.51323697	65.97108659	73.98197345	125.9763733
14.15707151	31.63716278	26.95932983	24.31127896	23.9659914	29.50797933
5.445027505	0	0.898644328	0.972451158	0	0
1261.06837	578.366882	372.0387517	505.6746024	612.6957802	517.5245605
28.31414303	82.05889096	149.1749584	69.04403225	149.0059465	157.7541972
213.4450782	235.3013982	583.2201687	484.2806769	317.809886	288.2702596
4.356022004	15.81858139	297.4512725	80.71344615	3.125998878	24.9682902
55.53928055	446.8749242	129.4047832	227.5535711	100.0319641	184.9923319
4.356022004	4.943306684	8.08779895	9.724511585	7.293997383	3.404766845
203.6440287	302.5303691	975.0290956	565.9665742	283.4238983	416.5164774
370.2618703	620.8793195	720.7127508	597.0850113	728.3577387	372.2545084
3622.032296	1983.254642	461.9031845	1098.869809	2566.445079	1692.169122
8.712044008	1.977322674	0	0	0	0
10.89005501	2.96598401	2.695932983	0	4.167998504	1.134922282
628.3561741	442.9202789	257.9109221	228.5260222	318.8518856	326.8576172
419.2671179	193.777622	70.99290189	180.8759155	122.9559559	99.8731608
1.089005501	0	0	0	0	0
19039.08317	3643.217026	9191.334184	2522.538305	8206.789055	6507.644364
103.4555226	70.19495491	67.39832458	70.01648341	71.8979742	82.84932657
1206.618095	352.9520972	274.9851643	31.11843707	610.6117809	82.84932657
140.4817096	188.8343153	273.1878756	254.7822035	232.3659166	165.6986531
13.06806601	0	0	0	0	0
26.13613202	0.988661337	0	1.944902317	5.209998131	2.269844564
411.6440794	286.7117877	469.9909834	155.5921854	249.0379106	532.2785502
284.2304358	605.0607381	724.3073282	809.0793638	389.7078602	685.4930582
3137.424848	888.8065418	291.1607622	469.6939095	1543.201446	831.8980326
1.089005501	0	0	0	0	0

5539.770984	3509.747746	1295.845121	2727.725499	2417.439133	3508.044773
0	1849.785361	2.695932983	205.1871944	9.377996635	6.809533691
808.0420818	2879.970474	2203.475892	1869.051127	2717.535025	2518.392543
13.06806601	43.50109882	64.7023916	32.09088823	19.7979929	11.34922282
118.7015996	323.2922571	387.3157053	720.5863084	377.2038647	190.6669433
101.2775116	123.5826671	175.2356439	176.9861108	158.3839432	313.2385498
410.5550739	714.8021465	831.2460032	591.2503043	561.6377985	596.9691202
3.267016503	15.81858139	9.885087605	12.64186506	18.75599327	17.02383423
6.534033006	145.3332165	53.02001534	30.14598591	21.88199215	10.21430054
198.1990012	87.00219764	120.4183399	91.41040889	110.4519604	89.65886026
7.623038507	39.54645347	17.07424223	25.28373012	15.62999439	28.37305705
2390.367075	339.1108385	70.99290189	225.6086688	609.5697813	520.9293274
190.9571146	115.7524693	47.62814937	108.710315	138.7422502	92.01949861
108.9005501	346.0314679	589.510679	517.3440163	381.3718632	225.8495341
45.73823104	64.26298689	143.7830924	110.8594321	67.7299757	112.3573059
9.801049509	21.75054941	22.46610819	52.51236256	23.9659914	11.34922282
1.089005501	0.988661337	4.493221639	5.834706951	11.46199589	4.539689127
76.23038507	272.870529	315.424159	238.2505338	289.6758961	157.7541972
8.712044008	32.62582411	29.65526282	23.3388278	37.51198654	34.04766845
1.089005501	0	3.594577311	0	1.041999626	0
0	0	0	0	2.083999252	0
104.5445281	161.1517979	254.3163447	137.1156133	129.2079536	238.3336792
108.9005501	213.5508488	271.390587	182.8208178	154.2159447	200.8812439
141.5707151	279.7911583	265.998721	375.3661472	249.0379106	156.6192749
494.4084975	654.493805	929.1982349	883.958103	656.4597645	593.5643534
22.86911552	2204.714781	0	2.917353475	436.5978433	2419.654305
91.47646209	194.7662834	186.0193758	55.42971603	149.0059465	124.841451
103.4555226	203.6642354	197.7017521	155.5921854	274.0459017	287.1353373
149.1937536	311.4283211	293.8566952	896.5999681	388.6658605	186.1272542
24.17592212	66.50724813	99.62371017	92.64542187	49.94304208	47.30356071
1503.916597	459.7275216	479.876071	436.6305701	1099.309606	671.8739908
1.089005501	0	0	0	2.083999252	0
298.3875073	744.4619866	486.1665813	622.3687414	462.647834	475.5324361
1.089005501	19.77322674	50.32408235	90.43795774	31.25998878	56.74611409
55.53928055	121.6053444	165.3505563	105.9971763	129.2079536	51.07150268
147.0157426	195.7549447	405.2885918	312.1568219	184.4339338	224.7146118
7028.441504	2906.66433	226.4583706	27.22863244	2423.69113	18.15875651

47.91624205	221.4601394	247.1271901	252.8373012	161.509942	106.6826945
46.82723654	130.5032965	421.4641897	169.2065016	109.4099607	112.3573059
737.2567242	327.2469025	428.6533443	348.1375147	274.0459017	186.1272542
0	0	0	0	0	0
59.89530256	207.6188807	290.2621179	123.5012971	58.35197906	127.1112956
0	0	0	0	0	0
2154.052881	1040.071726	1223.953574	916.0489913	1719.299383	1562.787982
0	6.920629358	3.594577311	4.862255792	0	4.539689127
741.6127462	317.3602891	210.2827727	382.1733053	461.6058344	473.2625915
4.356022004	82.05889096	16.1755979	688.4954202	0	6.809533691
551.0367835	184.87967	124.0129172	134.1982599	301.1378919	470.992747
2.178011002	2.96598401	0	0	0	2.269844564
21.78011002	22.73921075	91.66172143	35.98069286	23.9659914	32.91274617
80.98933911	123.5826671	210.4085829	161.7866992	93.13392658	95.33347167
2994.765128	688.1082904	384.6197723	1590.930095	629.3677742	1012.350675
4.356022004	7.909290694	11.68237626	26.25618128	10.41999626	14.75398966
3.267016503	1.977322674	36.84441744	93.35531121	1.041999626	2.269844564
212.3560727	452.8068923	628.1523851	347.1650636	325.1038833	170.2383423
6.207331356	1.552198299	0	0.972451158	1.041999626	1.134922282
1792.503055	932.3076406	81.77663382	440.5203748	1298.331534	702.5168925
6.534033006	0	0	0	3.125998878	0
111.0785611	152.481238	194.4306867	163.5857339	140.6282695	165.9369868
426.8901564	33.61448545	1.797288655	9.724511585	133.3759521	53.34134725
27.22513753	50.42172818	51.22272668	56.40216719	47.9319828	52.20642496
87.12044008	39.54645347	42.2362834	3.889804634	47.9319828	21.56352335
5.445027505	22.73921075	12.58102059	22.36637664	4.167998504	3.404766845
1413.52914	3346.618625	2900.82389	3416.22092	3469.858755	2744.242077
3.267016503	17.79590406	73.68883487	49.59500908	9.377996635	13.61906738
430.1571729	275.836513	237.2421025	153.647283	228.1979181	99.8731608
47.91624205	36.58046946	15.27695357	28.2010836	22.92399177	19.29367879
0	7.909290694	2.695932983	2.917353475	5.209998131	2.269844564
249.3822597	442.9202789	992.1033378	575.6910858	423.0518482	431.2704671
10.89005501	24.71653342	23.36475252	36.95314402	21.88199215	43.12704671
6.534033006	10.8752747	186.9180202	38.89804634	63.56197719	66.96041463
2.178011002	8.897952031	79.97934517	26.25618128	7.293997383	18.15875651
100.2211763	250.1313182	319.234411	260.6363595	223.2484199	230.4005724
191.1313555	441.8129782	251.1351438	579.0752158	294.0627145	548.3263512

423.6231399	739.5186799	747.6720807	1200.977181	828.3897028	727.4851827
19.60209902	10.8752747	0	0	4.167998504	0
0	2.96598401	9.885087605	0.972451158	3.125998878	4.539689127
19.60209902	0	0	0.972451158	2.083999252	0
1.089005501	5.931968021	1.797288655	3.889804634	7.293997383	6.809533691
8.712044008	32.62582411	24.26339685	7.779609268	18.75599327	18.15875651
182.7786833	256.0632862	334.1608933	777.8831307	326.3334429	325.4389643
28.31414303	31.63716278	154.5668244	16.53166969	36.46998691	211.0955444
2.178011002	3.954645347	24.26339685	5.834706951	8.335997009	5.674611409
1.089005501	0	0	0	0	0
803.6860598	321.3149345	71.89154622	270.3414221	320.9358848	104.4128499
288.5864578	697.0062425	1863.788336	923.8286005	524.1258119	346.151296
250.4712652	106.7754244	45.83086071	117.6665902	246.9539114	26.10321248
264.6283367	560.570978	718.9154622	744.8975874	410.5478527	426.730778
434.0667027	731.9653073	1253.258366	957.2420223	791.3257761	650.389912
80.58640708	174.0043953	140.1885151	131.2809064	123.9979555	170.2383423
19.60209902	4.943306684	0	0	0	0
17.51120846	37.5691308	40.78946604	103.3521091	51.42268155	54.48761875
199.2880067	381.623276	846.5229567	505.6746024	326.145883	383.6037313
2278.199508	11369.60537	19064.73941	7847.680849	6955.347504	1651.31192
0	0	0	0	0	2.269844564
327.7906558	1178.484313	3243.207379	958.8368422	598.1077854	3790.640421
177.5078967	111.7187311	99.74952038	63.2093253	193.8119305	195.2066325
2.178011002	9.886613368	126.7088502	23.3388278	6.251997757	35.18259074
0	14.24660986	0	3.539722217	5.209998131	6.469057006
26.13613202	233.3240755	77.28341218	70.01648341	98.98996448	157.7541972
54.45027505	33.61448545	7.189154622	10.69696274	41.67998504	29.50797933
9.801049509	22.54147848	44.03357206	29.0471161	22.92399177	17.02383423
548.8587725	226.4034461	237.2421025	291.7353475	382.4138628	296.2147156
2.178011002	0	0	0	0	0
1.089005501	2.96598401	13.47966492	5.834706951	6.251997757	11.34922282
487.8744645	298.5757237	311.8295817	355.917124	323.0198841	374.524353
298.3875073	160.1631366	246.2285458	97.24511585	154.2159447	213.365389
2.178011002	0	0	0	0	0
60.98430806	87.99085898	833.9419361	180.8759155	91.6959671	450.5641459
1.089005501	0	0	0	0	0
9.801049509	14.82992005	54.81730399	35.0082417	14.58799477	31.77782389

13.06806601	1.977322674	1.797288655	0	3.125998878	1.134922282
0	0	4.007953702	36.43774491	24.34111127	4.165164774
420.3561234	972.8427554	1269.784435	1258.351799	1044.083625	596.9691202
206.9110452	86.0135363	51.22272668	84.60325079	130.2499533	113.4922282
3.963980024	0	0	0	0	0
804.7750653	104.7981017	7.189154622	61.26442298	252.1639095	81.71440429
197.1099957	442.9202789	495.1530246	390.9253657	368.8678676	376.7941976
142.1043278	249.1327703	2147.283662	739.0531559	181.9852347	533.6064092
30.49215403	40.53511481	37.74306176	0	35.42798729	6.809533691
1.089005501	0	0	0	0	0
2.178011002	0	0.898644328	0	0	0
30.49215403	29.6598401	89.86443277	82.65834847	63.56197719	63.55564778
476.9844094	110.7300697	20.66881954	79.74099499	175.0559372	78.30963745
0	0.988661337	0.898644328	0.972451158	2.083999252	2.269844564
44.64922554	87.00219764	216.573283	48.62255792	83.35997009	125.9763733
568.4608715	970.8654327	1555.553331	1244.737483	1094.099607	3250.417415
1580.146982	1277.350447	903.1375494	607.781974	1171.20758	1729.621558
143.7487261	996.5706275	470.8896277	1073.586079	455.3538366	234.9289123
162.2618197	387.555244	813.2731166	401.6223284	266.7519043	346.151296
1505.005602	688.1082904	345.0794218	736.145527	1145.157589	427.8657002
10.89005501	0	0	0	0	0
490.0524755	181.913686	121.3169842	247.0025942	310.5158886	365.4449747
32.67016503	57.34235754	53.91865966	46.67765561	81.27597084	64.69057006
3931.309859	1413.785712	1527.695357	671.9637505	1214.971564	1332.398759
2.178011002	8.897952031	99.74952038	7.779609268	5.209998131	12.4841451
149.1937536	264.9612383	797.996163	1072.613628	98.98996448	873.890157
1485.403503	3039.144949	4712.490855	3591.262128	2745.669015	1845.38363
295.1204908	746.4393093	371.1401074	2436.962603	1009.697638	944.2553385
0	3.954645347	2.695932983	5.834706951	3.125998878	1.134922282
3.267016503	25.70519476	2.695932983	15.55921854	13.54599514	18.15875651
194.9319847	442.9202789	695.5507097	907.2969308	499.1178209	236.0638346
32.67016503	28.67117877	88.06714412	70.01648341	65.64597645	90.79378255
16.33508252	76.12692293	39.54035042	37.92559518	56.26797981	28.37305705
80.58640708	19.77322674	1.797288655	22.36637664	36.46998691	14.75398966
829.0054377	295.7876987	248.7806957	301.2459199	187.4869927	563.7272466
57.71729155	57.34235754	60.20916996	24.31127896	73.98197345	28.37305705
9.801049509	24.71653342	53.91865966	11.6694139	14.58799477	45.39689127

23.97990113	57.33247092	39.63021485	45.1606318	40.83596535	226.2921538
46.82723654	157.1971526	78.18205651	176.9861108	205.2739263	105.5477722
4.356022004	32.62582411	25.16204118	44.73275329	14.58799477	30.64290161
13.06806601	27.68251743	34.14848445	9.724511585	10.41999626	11.34922282
854.8693183	301.5417077	490.6598029	369.5314402	687.7197532	658.2549235
8.712044008	3.954645347	0	0	1.041999626	3.404766845
449.7592719	274.8478516	192.3098861	97.24511585	215.6939226	535.683317
89.29845108	179.9363633	210.2827727	351.0548682	220.9039207	223.5796895
121.9686161	199.70959	234.5461695	236.3056315	231.323917	147.5398966
29.40314853	63.27432556	76.38476786	57.37461835	40.63798542	68.09533691
465.0053489	488.3987004	68.29696891	112.8043344	296.9698934	257.627358
26.13613202	72.17227759	184.2220872	117.6665902	61.47797794	47.66673584
3772.315056	2230.419976	1188.906446	815.8865219	2730.03902	2636.424461
1236.021244	719.7454532	546.3757513	336.4681008	677.299757	1164.430261
19.60209902	8.897952031	5.391865966	5.834706951	7.293997383	6.809533691
16.5093234	50.8073061	69.01588437	28.59006406	51.43310155	78.88844781
64.25132456	117.6506991	249.8231231	116.694139	72.93997383	93.06362711
5.445027505	357.8954039	407.0858805	411.34684	64.60397682	108.9525391
47.709331	116.2468	349.725413	143.2420556	77.97283202	144.5890987
50.09425305	448.8522469	1077.474549	112.8043344	300.0958923	727.4851827
77.3847309	230.3580915	799.3980482	254.7822035	181.4433949	191.8018656
4.356022004	18.7845654	31.45255147	34.03579055	6.251997757	10.21430054
9.441677694	0	0	0.486225579	6.251997757	0
210.1780617	119.6280218	17.97288655	39.8704975	98.98996448	69.23025919
35.93718153	10.8752747	22.46610819	25.28373012	22.92399177	10.21430054
66.42933556	2.96598401	0.898644328	1.944902317	47.9319828	2.269844564
1757.731109	678.2216771	457.4099628	544.5726487	1315.003528	322.317928
479.1624205	224.4261235	194.1071748	202.269841	406.3798542	501.6356486
9.801049509	26.69385609	23.36475252	20.42147433	35.42798729	14.75398966
17.42408802	22.73921075	67.39832458	61.26442298	31.25998878	46.53181355
145.9267371	291.6550944	221.9651489	347.1650636	298.0118931	315.5083943
108.9005501	19.77322674	70.09425756	1.944902317	32.30198841	0
370.2618703	580.3442047	705.4357973	834.363094	434.5138441	886.3743021
9.801049509	18.7845654	42.2362834	38.89804634	11.46199589	23.83336792
2.178011002	25.70519476	4.493221639	8.752060426	8.335997009	12.4841451
317.728245	534.3319061	958.2873518	717.707853	571.5993149	647.0872882
11.97906051	24.71653342	42.2362834	23.3388278	13.54599514	24.9682902

67.51834106	121.6053444	165.3505563	109.8869809	83.35997009	104.4128499
202.5550232	361.8500493	698.2466426	249.9199477	251.1219099	459.6435241
87.12044008	151.2651845	74.5874792	170.1789527	40.63798542	195.2066325
573.905899	132.4806191	272.2892313	17.50412085	356.3638721	32.91274617
0	0	8.08779895	194.4902317	0	0
5.445027505	50.42172818	18.87153088	20.42147433	14.58799477	22.69844564
46225.0165	3414.836257	364.8495971	1159.161781	1664.073403	297.3496378
2.178011002	2509.222473	5.391865966	32.09088823	68.77197532	36.31751302
1013.864121	432.0450042	269.5932983	350.9867966	499.1178209	776.3208884
1159.790859	298.5757237	321.7146693	260.6169105	851.3136945	659.3898457
14957.98061	8886.038662	4938.931252	4800.757981	13254.19356	15043.20191
3.267016503	0	0.898644328	0	1.041999626	0
300.5655183	132.4806191	71.89154622	43.76030213	121.9139563	154.3494303
13.06806601	19.77322674	47.62814937	32.09088823	25.00799103	35.18259074
2082.178518	2123.644551	1949.159547	1079.420786	1818.289348	1689.899278
1009.508099	1206.166831	2947.553395	2222.050897	969.0596523	1361.906738
205.8329297	404.4020332	394.5408056	391.9367149	383.4975424	275.7974637
11.97906051	2.96598401	0.898644328	1.944902317	0	0
113.2565721	170.0497499	301.0458498	366.6140867	93.77996635	119.1668396
1.089005501	29.6598401	19.77017521	7.779609268	9.377996635	19.29367879
0	0	7.189154622	5.834706951	5.209998131	1.134922282
129.5916546	126.5486511	301.0458498	162.3993435	164.6359409	165.6986531
523.811646	717.7681305	842.9283794	1146.519916	724.1897402	613.9929545
156.8167921	110.7300697	45.83086071	0.972451158	1.041999626	17.02383423
178.1177397	103.8094404	105.5367898	90.92418332	131.3232129	164.3934925
1.089005501	0	0	0	0	0
0	1.977322674	6.290510294	71.96138573	0	4.539689127
3.267016503	14.82992005	20.66881954	25.28373012	23.9659914	12.4841451
28.31414303	301.5417077	1662.492006	122.528846	103.157963	64.69057006
113.2565721	197.7322674	294.7553395	115.7216879	166.7199402	240.6035237
332.1466778	434.0223269	553.5649059	601.9472671	604.3597831	490.2864257
231.9581717	442.9202789	768.3409002	627.2309972	349.0698748	289.4051819
40.29320354	62.28566422	108.7359637	66.12667878	67.7299757	62.4207255
373.5288869	1000.525273	691.9561324	1002.597144	891.95168	625.3421773
0	0	0	4.862255792	0	15.88891195
200.3770122	568.4802687	1107.066907	469.1396124	420.967849	204.2860107
10.89005501	1.977322674	2.695932983	10.69696274	2.083999252	1.134922282

5645.404517	2951.15409	898.6443277	1782.502973	3093.69689	4379.665086
492.2304865	137.4239258	216.573283	134.1982599	349.0698748	363.1751302
14.15707151	7.909290694	0	0.972451158	0	0
1.089005501	0.988661337	0	0	0	0
133.9585667	294.3640264	497.8130118	209.8257865	204.4403266	249.1948854
95.9522747	229.3496569	335.7425073	258.4386199	165.4903806	173.0869972
1599.749081	619.8906582	1280.568167	705.999541	872.1536871	1164.430261
215.6230892	199.70959	1496.242806	240.1954361	531.4198093	486.8816589
320.1676173	166.0951046	137.4925821	99.19001816	274.0459017	369.9846639
10.89005501	40.53511481	4.493221639	43.76030213	36.46998691	162.2938863
369.1728648	252.1086409	190.5125975	136.1431622	320.9358848	192.9367879
5.445027505	2.96598401	8.08779895	20.42147433	5.209998131	13.61906738
70.78535757	653.5051436	437.6397876	516.3715651	217.7779219	282.5956482
0	3.954645347	4.493221639	3.889804634	0	2.269844564
109.9677755	189.8328633	78.09219208	174.61333	156.101964	724.7727184
2.820524248	3.954645347	0	0.486225579	0	0
5.445027505	4.943306684	6.290510294	15.55921854	7.293997383	15.88891195
88.20944558	99.85479502	133.8980048	103.0798228	117.7459578	113.4922282
2507.979669	5217.165874	13306.22656	10962.44191	3082.234894	6150.143845
21.78011002	53.38771219	5.391865966	79.74099499	89.61196785	89.65886026
888.6284888	369.75934	336.0929786	507.6195047	520.9998131	456.2387573
2.178011002	0	0	0	0	0
0	0.988661337	0	0	0	0
0	0	0	0	0	0
3.571938043	0	0	0	0	0
0	0	0	0	0	0
1.546387811	0	0	0	0	0
0	0	0	0	0	0
171.4965863	82.978346	84.92188897	70.24014718	155.1433243	117.1920748
154.6387811	80.08156828	46.72950504	33.06333939	79.19197159	43.12704671
154.6387811	46.46708283	35.94577311	50.56746024	44.80598392	88.52393798
70.78535757	96.88881101	132.9993605	165.3166969	75.02397308	68.09533691
119.7906051	402.3851641	518.5177771	579.5808904	195.8959297	146.4049744
707.8535757	263.9725769	98.85087605	216.8566083	301.1378919	189.5320211
20.69110452	35.59180813	38.64170609	48.62255792	37.51198654	47.66673584
2.178011002	6.920629358	5.391865966	4.862255792	11.46199589	1.134922282
1656.377367	848.271427	1070.285394	1470.346152	1274.365543	547.0325398

610.9320861	402.3851641	296.5526282	458.9969468	548.0918033	365.4449747
4161.090019	2505.267827	2167.530118	921.8836982	3254.164832	1910.0742
76.23038507	108.752747	244.4312571	211.0219014	93.77996635	116.896995
19.60209902	51.41038951	244.4312571	11.6694139	54.18398056	95.33347167
1.089005501	0	0.898644328	11.6694139	3.125998878	20.42860107
124.1466271	165.1064432	629.0510294	256.7271058	167.7619398	255.3575134
20.69110452	16.80724273	97.05358739	34.03579055	26.04999065	46.53181355
46.82723654	571.4462527	386.4170609	473.5837142	127.1239544	220.1749227
41.38220904	157.1971526	70.99290189	98.217567	56.26797981	157.7541972
32.67016503	60.30834155	41.33763908	54.45726487	101.0739637	48.80165812
184.0419297	390.521228	355.8631538	347.1650636	289.6758961	511.8499491
118.7015996	127.5373124	181.5261542	140.0329668	133.3759521	129.3811401
283.1414303	1060.833614	395.4035042	509.564407	626.2417753	557.2468404
16.33508252	41.52377615	47.62814937	125.4461994	35.42798729	60.15088094
227.6021497	117.6506991	86.26985546	71.96138573	104.1999626	140.7303629
0	6.920629358	6.290510294	9.724511585	16.67199402	1.134922282
2.178011002	12.85259738	6.290510294	7.779609268	2.083999252	6.809533691
68.60734656	22.73921075	5.391865966	13.61431622	20.83999252	6.809533691
1379.258137	4031.35558	3202.256157	6153.136083	2955.41312	4768.750491
1715.183664	566.502946	330.7011126	433.7132167	583.5197906	362.0402079
5.445027505	25.70519476	15.27695357	13.61431622	8.335997009	41.99212443
462.8273379	577.3782207	1740.674063	2475.860649	667.9217603	724.0804158
276.6073973	115.6733764	17.97288655	118.6390413	63.56197719	55.61119181
290.7644688	142.3672325	35.04712878	68.07158109	135.4599514	150.9446635
638.1572236	265.9498996	238.1407468	344.2477101	301.1378919	256.4924357
29.40314853	69.20629358	97.95223172	243.1127896	65.64597645	56.74611409
41.38220904	75.1382616	121.3169842	112.8043344	75.02397308	47.66673584
5.445027505	156.2084912	90.7630771	96.27266469	50.01598205	43.12704671
37.02618703	158.1858139	1321.007162	176.9861108	102.1159634	70.36518147
9.801049509	16.80724273	31.45255147	44.73275329	38.55398617	14.75398966
508.565569	216.5168328	337.8902672	210.0494502	241.7439133	452.8339904
6.534033006	0	0	0	0	0
80.58640708	157.1971526	129.4047832	103.0798228	121.9139563	94.19854939
4.356022004	41.52377615	13.47966492	109.8869809	5.209998131	2.269844564
27.22513753	57.34235754	70.09425756	41.81539981	29.17598953	19.29367879
7.623038507	0.988661337	0.898644328	0	0	0
6.534033006	0	0.898644328	0	1.041999626	1.134922282

9.801049509	41.52377615	52.12137101	23.3388278	98.98996448	12.4841451
24.33927295	190.9302774	1059.133218	257.3980971	17.41181375	83.57567683
383.3626065	290.666433	164.451912	240.3802019	315.7258867	244.0082906
0	1.977322674	1.797288655	0.972451158	0	2.088256999
635.9792126	1069.731566	2231.333866	1671.643541	1045.125625	970.358551
346.3037493	202.675574	91.66172143	176.9861108	183.3919342	108.9525391
1.481047481	0	0	0	0	0
87.12044008	34.60314679	22.46610819	1.944902317	3.125998878	6.809533691
17.42408802	43.50109882	33.24984013	36.95314402	43.7639843	41.99212443
679.5394326	404.3624868	474.484205	352.0273194	493.9078228	713.8661153
8.712044008	0.988661337	11.68237626	0.972451158	5.209998131	3.404766845
5.532147945	44.02508933	50.32408235	34.03579055	38.55398617	23.83336792
645.7802621	358.8840653	489.7611586	423.0162539	492.8658232	516.3896382
222.1571222	49.43306684	43.13492773	127.3911018	137.5439506	26.10321248
131.7696656	98.86613368	74.5874792	63.2093253	87.52796859	143.0002075
2929.424798	1034.139758	187.8166645	377.3110495	2631.049056	2654.583217
2.178011002	0	0	0	0	0
42.47121454	61.29700288	100.6481647	86.5481531	72.93997383	32.91274617
5.445027505	17.79590406	40.43899475	33.06333939	26.04999065	35.18259074
89.29845108	123.5826671	450.2208082	355.917124	101.0739637	215.6352335
1306.54524	307.7702741	66.40082938	103.9744779	193.6139505	387.8369914
119.7906051	153.2425072	251.6204118	355.917124	225.0719192	350.6909851
31.58115953	9.886613368	6.290510294	7.779609268	28.13398991	12.4841451
371.3508758	644.6071916	381.9238393	433.7132167	1185.795575	826.2234212
143.7487261	69.20629358	112.330541	73.90628804	46.88998318	73.76994832
1.089005501	23.72787208	27.85797416	0	6.251997757	46.53181355
427.9791619	3440.541452	1690.34998	2144.254804	2156.939226	1545.764148
3.267016503	17.79590406	29.65526282	54.45726487	3.125998878	13.61906738
147.0157426	93.922827	19.77017521	51.5399114	59.39397869	35.18259074
4.356022004	0	0	0	1.041999626	0
5.445027505	0.988661337	0	0	1.041999626	1.134922282
30.49215403	88.97952031	102.4454534	79.74099499	58.35197906	54.47626953
0	0	0	0	0	0
14.15707151	3.954645347	0	1.944902317	4.167998504	4.539689127
1364.523893	2316.433512	6486.414758	2331.937878	1868.30533	2900.861352
495.497503	1239.781316	1001.089781	703.0821876	811.7177087	530.0087056
192.7539737	25.70519476	16.1755979	17.50412085	28.13398991	54.47626953

3.267016503	16.80724273	54.81730399	30.14598591	7.293997383	11.34922282
0	1.977322674	1.797288655	3.889804634	0	1.134922282
1.089005501	0	0	0	0	0
44.64922554	72.17227759	963.3467193	134.1982599	9.377996635	32.91274617
0	0	2.695932983	0	3.125998878	3.404766845
105.6335336	128.5259738	233.6475252	180.8759155	125.0399551	125.9763733
0	0.988661337	5.391865966	1.944902317	0	1.134922282
67.51834106	125.5599898	110.5332523	172.123855	89.61196785	179.3177205
51.18325855	107.7640857	42.2362834	92.38286005	70.85597458	60.15088094
13.06806601	36.58046946	48.5267937	41.81539981	28.13398991	20.42860107
50.09425305	68.21763224	94.35765441	131.2809064	78.14997196	28.37305705
165.5288362	263.9725769	1063.994884	203.2422921	269.8779032	206.5558553
68.60734656	156.2084912	216.573283	208.1045479	114.6199589	152.0795858
96.92148959	58.33101887	10.78373193	1.944902317	5.209998131	0
42.47121454	134.4579418	64.7023916	111.8318832	45.84798355	94.19854939
49.00524755	74.14960026	194.1071748	104.052274	65.64597645	89.65886026
263.5393312	119.6280218	149.1749584	173.0963062	229.2399177	153.214508
1.089005501	13.84125872	1.797288655	5.834706951	6.251997757	6.809533691
180.7749132	249.1426569	1072.981327	564.9941231	181.3079349	232.6590678
3.267016503	2.96598401	32.3511958	9.724511585	7.293997383	12.4841451
517.277613	489.3873617	523.0109987	436.6305701	638.7457708	488.0165812
0	6.920629358	3.594577311	3.889804634	0	1.134922282
34.84817603	24.71653342	19.77017521	14.58676738	27.09199028	11.34922282
0	20.76188807	32.3511958	17.50412085	15.62999439	6.809533691
3.267016503	0	0	0	0	0
66.42933556	250.1313182	35.94577311	467.7490072	161.509942	24.9682902
0	2.006982514	0	0	1.052419622	2.303892232
1.089005501	3.954645347	2.695932983	0	3.125998878	27.23813476
79.49740157	110.7300697	319.9173807	195.4626828	105.2419622	85.11917114
1.089005501	5.931968021	0	0	0	1.134922282
13.06806601	40.53511481	19.77017521	169.2065016	26.04999065	70.36518147
0	5.931968021	21.35178923	19.35177805	1.041999626	5.322785502
0	7.909290694	6.290510294	1.944902317	2.083999252	5.674611409
2.178011002	2.96598401	8.986443277	15.55921854	6.251997757	5.674611409
17.42408802	33.61448545	51.22272668	67.09912993	19.7979929	62.4207255
9.801049509	0.988661337	1.797288655	3.889804634	2.083999252	3.404766845
0	0	0	0	0	0

566.2828605	271.8818676	212.0800613	351.0548682	431.3878452	453.9689127
87.12044008	93.922827	43.13492773	135.170711	40.63798542	31.77782389
175.3298857	243.2106889	396.3021485	270.3414221	235.4919155	272.3813476
449.7592719	158.1858139	156.364113	112.8043344	257.3739077	280.3258036
4257.151195	2166.058123	1319.31771	725.1860024	4019.732378	2054.379568
933.2777144	816.6342642	435.8424989	388.0080122	1105.561603	823.9535766
2.178011002	0.988661337	0	0	0	0
1831.707253	623.8453035	515.8218441	935.4980144	704.3917473	999.8665303
45.73823104	58.33101887	67.39832458	131.2809064	54.18398056	113.4922282
94.74347859	161.1517979	357.6604424	276.176129	221.9459204	335.9369954
2391.45608	1469.150747	1738.876774	1085.255493	1755.76937	2499.098865
303.8325348	499.2739751	812.3744723	904.3795774	699.1817491	702.5168925
678.4504271	353.9407586	315.424159	188.6555247	129.2079536	167.9684977
2010.304155	799.8270215	1716.410666	1346.844854	574.141794	797.8049672
889.7174943	831.4641843	345.0794218	519.2889186	761.7017267	1462.914821
40.29320354	3.954645347	0	0	1.041999626	0
3.495707658	1.651064432	0.296552628	0	0	0.226984456
147.0157426	262.9839156	314.5255147	491.087835	234.4499159	265.5718139
53.36126955	118.6393604	227.3570149	70.98893457	160.4679424	155.4843526
99.09950059	121.6053444	281.2756746	114.7492367	147.9639469	207.6907776
500.3980277	283.2218132	393.7230393	510.5174092	315.8926067	482.5689542
1.089005501	6.920629358	2.695932983	3.889804634	13.54599514	4.539689127
180.7749132	4.943306684	66.49968025	88.49305542	46.88998318	27.23813476
173.1518747	693.0515971	717.1181735	495.9500908	444.9338404	1298.35109
101.2775116	93.922827	70.99290189	54.45726487	70.85597458	29.50797933
16.33508252	32.62582411	61.10781429	19.44902317	30.21798916	29.50797933
105.6335336	136.4352645	221.9651489	498.8674443	117.7459578	181.5875651
1.089005501	0	0.898644328	1.944902317	1.041999626	0
139.3927041	204.6528967	276.7824529	194.4902317	160.4679424	82.84932657
690.4294876	1387.091856	1194.298312	1796.11729	788.793717	1677.415133
14.15707151	25.70519476	84.47256681	73.90628804	17.71399364	26.10321248
9.801049509	22.73921075	36.84441744	30.14598591	21.88199215	11.34922282
154.6387811	216.5168328	478.9774267	327.7160404	205.2739263	364.3100525
1.089005501	1.977322674	0	2.917353475	0	99.8731608
482.429437	293.632417	50.32408235	213.9392549	527.2518108	313.2385498
68.60734656	102.820779	239.9380355	137.1156133	115.6619585	113.4922282
17.42408802	5.931968021	0	0	0	0

1585.592009	7477.24569	26387.79204	10008.46732	6421.843696	3123.30612
133.9476766	446.8749242	888.7592401	447.3275329	247.995911	414.2466329
5.924189926	0	1.132291853	0	0	0
62.07331356	120.6166831	177.0329326	168.2340504	133.3759521	88.52393798
1891.602555	5305.156733	6995.047447	4506.338668	2777.971003	2937.178865
0	15.81858139	3.594577311	2.917353475	5.209998131	4.539689127
854.8693183	759.2919067	183.3234429	613.616681	385.5398617	474.3975138
1.753298857	0	0	1.944902317	0	1.134922282
402.9320354	832.4528456	1072.082683	1142.630111	1072.217615	444.8895345
4.356022004	397.4418574	485.267937	0.972451158	300.0958923	677.5486023
836.3562248	351.9634359	291.1607622	405.5121331	301.1378919	192.9367879
49.00524755	76.12692293	130.3034275	172.123855	85.44396934	191.8018656
0	0	0	0	0	0
1.089005501	15.81858139	16.1755979	17.50412085	0	0
251.5602707	168.0724273	117.7224069	224.6362176	143.7959484	172.5081868
0	22.73921075	4.493221639	3.889804634	17.71399364	19.29367879
15.24607701	6.920629358	5.391865966	0.972451158	1.041999626	1.134922282
91.47646209	219.4828168	373.8360403	261.5893616	161.509942	135.0557515
1631.330241	583.3101887	226.4583706	567.9114765	428.2618463	188.3970988
18.51309352	6.920629358	0	0.972451158	2.083999252	0
55.53928055	9.886613368	0.898644328	4.862255792	22.92399177	13.61906738
321.2566228	550.6843646	749.4693693	842.1427032	541.8398056	359.7703633
361.5498263	211.5735261	204.8909067	149.7574784	387.6238609	146.4049744
210.1780617	309.4509984	400.7953702	565.9665742	327.1878826	417.6513997
54.45027505	82.05889096	273.1878756	465.8041049	71.8979742	40.85720215
11.97906051	10.8752747	14.37830924	21.39392549	26.04999065	9.079378255
2.254241387	4.112831161	0.943576544	4.045396819	12.07677567	7.150010376
117.6125941	264.9612383	205.7895511	325.7711381	213.6099234	256.4924357
2.178011002	13.84125872	3.594577311	1.944902317	6.251997757	4.539689127
28.31414303	65.25164823	108.7359637	72.93383688	40.63798542	72.63502604
0	8.897952031	18.87153088	13.61431622	1.041999626	0
254.8272872	334.1675318	524.8082874	389.9529145	236.5339151	1080.446012
4.356022004	0	0	0.972451158	0	0
46.82723654	102.820779	775.5300548	97.24511585	94.82196598	105.5477722
253.7382817	102.820779	131.2020718	96.27266469	145.8799477	101.0080831
0	0	0	0	0	0
186.2199407	274.8478516	357.6604424	285.9006406	231.323917	217.9050781

17.42408802	969.8767714	97.95223172	15.55921854	950.303659	868.2155456
2.178011002	0	0	0	0	0
820.0211423	230.3580915	177.0329326	162.3993435	565.805797	1241.604976
291.8534743	866.067331	465.4977618	683.6331644	589.7717884	305.2940938
512.921591	349.9861132	303.7417828	284.9281894	516.8318146	638.9612447
2.178011002	1.977322674	7.189154622	3.889804634	8.335997009	11.34922282
0	0.988661337	7.189154622	0	0	3.404766845
34.84817603	1.977322674	0	22.36637664	5.209998131	0
1.089005501	19.77322674	2.695932983	22.36637664	4.167998504	18.15875651
67.51834106	136.4352645	127.6074945	145.8676738	131.2919529	79.44455973
742.7017517	1045.015033	2007.571428	1202.922083	1078.469613	1071.366634
0	0	0	0	0	0
0	0	0	0	0	0
78.40839607	127.5373124	246.2285458	264.5067151	119.829957	81.71440429
417.0891069	173.0157339	138.3912265	185.7381713	203.1899271	259.8972025
9.779269399	0	0	0	0	3.370719177
0	0	0	0	0	0
401.8430299	666.357741	1048.71793	941.3327214	690.8457521	506.1753377
137.2146931	180.9250246	321.7146693	174.0687574	190.6859316	226.9844564
2.178011002	17.79590406	30.55390714	16.53166969	6.251997757	7.944455973
2.178011002	9.886613368	26.0606855	33.06333939	1.041999626	2.269844564
40.29320354	33.61448545	37.74306176	163.3717946	76.06597271	254.2225911
8.712044008	156.2084912	17.07424223	13.61431622	25.00799103	30.64290161
763.3928562	526.9564925	292.9580508	605.8370717	456.3958362	291.6750264
304.3008072	822.5662322	2897.094516	3986.077299	309.0362491	897.7235249
302.7435293	399.4191801	499.6462462	431.7683144	543.9238048	1012.350675
65.34033006	117.6506991	228.2556592	164.3442458	64.60397682	124.841451
467.1833599	148.2992005	54.81730399	57.37461835	160.4679424	57.88103637
296.2094963	146.3218778	27.85797416	98.217567	145.8799477	110.0874613
1017.131138	264.9612383	244.4312571	93.35531121	312.5998878	234.9289123
157.9057976	356.9067426	419.666901	592.2227555	284.4658979	231.5241455
25.04712652	45.47842149	109.634608	55.42971603	23.9659914	41.99212443
45.73823104	89.96818165	63.80374727	52.51236256	63.56197719	79.44455973
20.2119421	57.99487402	665.5449756	92.77184052	62.81173746	40.85720215
160.0838087	96.88881101	74.5874792	93.35531121	103.157963	140.7303629
60.98430806	139.4012485	138.6608198	459.3859273	189.643932	300.7544047
686.0734656	270.8932063	363.0523084	279.0934825	458.4798355	187.2621765

3.267016503	0	0.898644328	0	2.083999252	1.134922282
39.20419804	102.820779	112.330541	54.45726487	63.56197719	47.66673584
49.00524755	93.922827	135.6952935	113.7767855	80.23397121	55.61119181
46.82723654	278.802497	58.4118813	93.35531121	129.2079536	251.9527466
309.2775623	486.4213777	929.1982349	591.2503043	430.3458456	405.1672546
37.02618703	10.8752747	4.493221639	3.889804634	22.92399177	5.674611409
468.2723654	190.811638	225.5597263	214.911706	368.8678676	443.7546122
6470.870687	1754.873873	2101.030438	2113.136367	2913.430955	2160.892025
548.3360499	613.5632256	1445.918723	1083.71902	600.1917846	753.531649
35.93718153	79.09290694	239.0393912	127.3911018	47.9319828	44.26196899
123.0576216	196.743606	270.4919426	252.8373012	226.1139189	221.309845
153.5497756	155.2198299	179.7288655	213.9392549	230.2819174	276.9210368
1461.445382	239.2560435	48.5267937	135.170711	155.2579443	39.72227986
308.1885568	227.3921075	195.0058191	31.11843707	304.2638908	141.8652852
0	0	0	0	0	0
443.2252389	195.7549447	184.2220872	144.8952226	284.4658979	211.0955444
1.089005501	0.988661337	754.8612353	0.972451158	5.209998131	1545.764148
4.356022004	0	0	0	2.083999252	0
100.1885061	249.1426569	505.0381122	569.8563789	172.9719379	766.3335724
8.712044008	52.39905085	12.58102059	109.8869809	154.2159447	52.20642496
56.62828605	170.0497499	101.546809	85.57570194	107.3259615	74.9048706
3697.173676	4439.089402	666.7940912	3750.744118	2284.06318	1784.097827
3185.34109	342.0768225	24.26339685	102.1073716	38.55398617	27.23813476
241.7592212	111.7187311	87.16849979	143.9227715	181.3079349	162.2938863
1460.356377	815.6456029	1026.251822	1364.348975	1259.777548	854.5964782
40.29320354	60.30834155	106.0400307	83.63079963	32.30198841	63.55564778
2114.848683	3848.858584	5829.505754	3398.716799	4249.274475	5968.55628
237.4031992	545.7410579	1145.771518	515.399114	324.0618837	219.0400004
16.33508252	135.4466031	66.49968025	76.82364152	32.30198841	12.4841451
33.75917053	5.931968021	3.594577311	5.834706951	11.46199589	2.269844564
143.7487261	157.1971526	508.6326895	227.5535711	183.3919342	196.3415548
3512.042741	2041.585661	347.7753548	2141.337451	1360.851512	795.5805196
2.178011002	2.96598401	5.391865966	5.834706951	6.251997757	5.674611409
28.31414303	87.00219764	107.8373193	26.25618128	51.05798168	119.1668396
6.534033006	90.95684299	38.64170609	109.8869809	7.293997383	20.42860107
248.2932542	346.0314679	833.0432918	601.9472671	257.3739077	735.4296386
6.534033006	66.24030957	12.58102059	10.69696274	110.4519604	45.39689127

2163.853931	2009.948498	409.7818134	755.5945501	2706.073029	1268.843111
56.62828605	27.68251743	37.74306176	17.50412085	54.18398056	10.21430054
174.2408802	251.1199796	568.8418595	340.3579055	246.9539114	365.4449747
495.497503	958.0128354	1979.713454	981.2032189	1056.587621	974.8982401
248.2932542	359.8727266	434.9438546	336.4681008	369.9098673	350.6909851
174.2408802	139.4012485	42.2362834	0.972451158	4.167998504	30.64290161
0.773193906	0	0	0	0	0
0	0	3275.558575	2.917353475	235.4919155	14.75398966
57.71729155	157.1971526	267.7960097	151.7023807	164.6359409	157.7541972
5.445027505	4.943306684	14.37830924	954.9470376	303.2218912	1205.287463
2.178011002	6.920629358	15.27695357	8.752060426	2.083999252	9.079378255
1226.220194	366.793356	542.7811739	644.7351181	612.6957802	940.8505716
137.2146931	194.7662834	263.302788	265.4791663	192.7699308	131.6509847
1.089005501	1.977322674	4.493221639	5.834706951	4.167998504	12.4841451
26.13613202	37.5691308	56.61459265	587.3604997	23.9659914	24.9682902
272.2513753	102.820779	124.0129172	109.8869809	479.319828	110.0874613
67.51834106	61.29700288	168.9451336	106.9696274	102.1159634	81.71440429
121.1083018	6361.037154	81.80359315	5196.924858	67.53199577	172.8600127
120.8796106	159.1744752	228.2556592	214.911706	162.5519417	163.4288086
1216.419145	426.1130362	135.6952935	286.8730917	557.4698	258.7622803
68.60734656	136.4352645	176.1342882	274.2312267	114.6199589	137.3255961
1.089005501	2.96598401	6.290510294	13.61431622	0	7.944455973
2082.178518	1692.588209	1344.362928	1428.530752	1722.414962	1958.875858
160.0838087	306.4850144	419.666901	479.4184211	277.1719005	306.4290161
456.2933049	323.2922571	88.06714412	218.8015107	246.9539114	203.1510884
2302.157629	1103.346052	530.2001534	734.2006246	1630.729415	1493.557723
78.40839607	116.6620377	277.6810973	348.1375147	164.6359409	128.2462178
460.6493269	365.8046946	978.6236729	730.31082	378.2458643	270.1115031
65.34033006	101.8321177	334.2956899	49.59500908	196.9379293	187.2621765
11.97906051	11.86393604	4.493221639	0	0	0
5.445027505	45.47842149	1321.007162	176.9861108	1.041999626	12.4841451
22.86911552	23.72787208	1.797288655	6.807158109	1.041999626	3.404766845
68.60734656	123.5826671	248.0258345	165.3166969	133.3759521	102.1430054
333.2356833	133.4692805	115.0264739	129.3360041	149.0059465	207.6907776
0	47.45574417	211.181417	0.972451158	1.041999626	3.404766845
14.15707151	16.80724273	16.1755979	46.67765561	13.54599514	9.079378255
96.92148959	162.1404592	164.451912	145.8676738	113.5779592	106.6826945

161.1728142	414.2491001	204.8909067	251.86485	277.1719005	259.8972025
1.089005501	0.988661337	0	0	0	0
41.38220904	759.2919067	192.3098861	184.7657201	139.6279499	128.2462178
605.4870586	292.6437557	319.9173807	281.7968967	357.4058718	667.3343017
0	1.977322674	36.84441744	14.58676738	5.209998131	0
233.0471772	351.9634359	363.0523084	423.0162539	233.4079163	468.7229024
351.7487768	184.87967	198.6003964	207.1320968	207.3579256	169.10342
2.178011002	30.64850144	12.58102059	5.834706951	12.50399551	26.10321248
388.7749639	246.1766729	118.6210513	173.0963062	249.0379106	208.8256999
726.3666692	523.9905085	225.5597263	949.1123307	568.9317959	678.6835245
26.13613202	16.80724273	1.797288655	1.944902317	1.041999626	0
111.0785611	221.4601394	204.8909067	164.3442458	112.5359596	105.5477722
223.2461277	76.12692293	236.3434582	174.0687574	133.3759521	110.0874613
688.2514766	428.0903588	359.4577311	331.605845	364.6998691	329.1274617
7.557698177	0.494330668	0	6.272309972	1.041999626	0
136.1256876	76.12692293	8.986443277	79.74099499	82.31797046	65.82549235
4.356022004	8.897952031	54.81730399	0.972451158	2.083999252	47.66673584
182.9529242	702.9382105	953.4616317	197.4075852	828.3897028	1694.438967
154.6387811	58.33101887	26.0606855	39.8704975	84.40196972	103.2779276
5.445027505	0	37.74306176	136.1431622	43.7639843	1.134922282
278.7854083	471.5914577	811.4758279	397.7325238	398.0438572	469.8578247
224.3351332	327.2469025	448.4235195	279.0934825	289.6758961	435.8101562
127.4136436	221.4601394	292.9580508	317.0190777	188.6019323	131.6509847
547.769767	1000.525273	2713.007225	1236.957874	746.0717323	997.5966857
493.319492	794.8837148	505.0381122	856.7294706	860.6916912	1962.280625
72.96336857	184.87967	346.8767105	271.3138732	177.1399364	206.5558553
370.2618703	634.7205782	1090.05557	684.6056156	607.485782	421.0561666
0	0	0	0	0	0
83.85342358	12.85259738	17.07424223	8.752060426	44.80598392	41.99212443
235.2251882	360.8613879	765.6269943	489.0067895	291.7598953	530.9847388
16.33508252	10.8752747	18.87153088	13.61431622	16.67199402	4.539689127
29.40314853	45.47842149	88.96578845	55.42971603	51.05798168	35.18259074
3.267016503	27.68251743	84.47256681	4.862255792	11.46199589	0
3138.513854	2163.191005	2860.384895	1747.494732	2932.186948	2369.717724
9.801049509	39.54645347	192.3098861	7.779609268	17.71399364	162.2938863
14.15707151	1.977322674	0.898644328	3.889804634	4.167998504	4.539689127
149.1937536	61.29700288	67.39832458	20.567342	62.25947766	86.25409342

93.13175045	419.9141296	1088.491928	684.7903813	372.1501665	157.0732438
468.2723654	784.9971014	1698.437779	1210.701692	702.307748	988.5173075
118.7015996	206.6302194	196.8031078	363.6967333	159.4259428	124.841451
198.1990012	546.7297193	613.7740758	493.0327373	443.8918407	203.1510884
2010.304155	934.2849633	855.5094	583.4706951	866.9436889	1683.089744
87.12044008	196.743606	2412.86002	655.4320808	175.0559372	585.6198974
535.7907065	407.3284708	150.9722471	494.9776397	231.323917	236.0638346
0	0	95.25629874	0.972451158	0	23.83336792
138.3036986	234.3127368	243.5326128	189.6279759	253.2059091	162.2938863
1.089005501	35.59180813	17.07424223	26.25618128	9.377996635	10.21430054
59.89530256	62.28566422	15.27695357	15.55921854	44.80598392	24.9682902
26.13613202	15.81858139	0.898644328	2.917353475	18.75599327	7.944455973
582.617943	978.7747234	1366.838022	934.5255633	803.3817117	1111.088914
52.27226405	94.91148833	193.2085305	172.123855	102.1159634	64.69057006
26.13613202	23.72787208	0	3.889804634	7.293997383	18.15875651
312.5445788	356.9067426	602.0916996	407.4570354	402.2118557	436.9450785
113.5614936	52.39905085	1.797288655	3.889804634	59.39397869	35.18259074
2035.841334	1249.737136	986.2172175	20.42147433	1014.751336	291.6409788
1.089005501	0.988661337	6.290510294	0	0	23.83336792
239.6465506	124.9964528	2.839716076	31.28375377	144.0147683	2.269844564
90.38745659	126.5486511	281.2756746	259.6444593	125.0399551	119.1668396
735.0787132	534.8657832	566.1459265	521.2338209	650.2077667	86.25409342
2.178011002	0.988661337	0	32.09088823	1.041999626	0
804.7750653	1994.129916	2331.98203	2696.607062	1933.951306	1714.867568
35.93718153	0.988661337	0.898644328	8.752060426	1.041999626	6.809533691
241.7592212	429.0790202	532.8960863	512.4817605	319.8938852	279.1908813
28.31414303	45.47842149	123.1142729	175.0412085	75.02397308	86.25409342
173.1518747	265.9498996	519.4164214	268.3965197	268.8359035	135.0557515
486.8072391	682.2356421	1063.644413	1208.970729	584.3533903	1038.839761
1459.267371	1585.812784	381.025195	1086.227944	1404.615496	911.3425923
4.356022004	57.34235754	19.77017521	64.18177646	1.041999626	1.134922282
7.623038507	9.886613368	42.2362834	116.694139	16.67199402	7.944455973
231.9581717	785.9857628	2829.830988	802.2722057	519.9578134	640.096167
2090.890562	1202.212186	886.9619515	625.2860949	1704.711388	1921.423423
14.15707151	60.30834155	25.16204118	44.73275329	38.55398617	20.42860107
4.356022004	11.86393604	6.290510294	20.42147433	9.377996635	10.21430054
872.2934063	551.6730259	522.1123544	385.0906587	656.4597645	331.3973063

524.9006515	404.3624868	135.6952935	223.6637664	613.7377798	334.8020731
10.89005501	15.81858139	19.77017521	25.28373012	16.67199402	17.02383423
9.278326869	24.9241523	81.99230846	95.83506167	33.73994789	129.9599505
1784.880016	3224.024619	5829.505754	4144.586837	2252.803192	9805.728515
16.33508252	5.931968021	0.898644328	8.752060426	11.46199589	4.539689127
288.5864578	345.0428065	1014.569446	474.5561653	385.5398617	417.6513997
0	0.988661337	2.695932983	0	0	1.134922282
90.38745659	122.5940058	398.0994372	85.57570194	67.7299757	143.0002075
96.92148959	166.0951046	376.5319733	144.8952226	142.7539488	133.9208293
8.712044008	1.977322674	0	0	0	0
5.445027505	16.80724273	14.37830924	16.53166969	17.71399364	10.21430054
148.1047481	151.2651845	44.03357206	76.82364152	118.7879574	80.57948201
15.24607701	521.0245245	143.7830924	161.4268923	31.25998878	59.01595865
370.2618703	477.5234257	706.3344416	825.6110335	456.3958362	645.7707784
408.3770629	639.6638849	692.8547767	763.3741594	507.4538179	569.7309855
140.4817096	322.3035958	508.6326895	141.005418	156.2999439	1014.62052
1.089005501	0	0	0	2.083999252	0
22.86911552	3.954645347	1.797288655	1.944902317	6.251997757	0
564.1048495	264.9612383	281.2756746	341.3303566	453.2698374	448.2943013
0	0.988661337	2.695932983	0	2.083999252	1.134922282
362.6388318	252.1086409	149.1749584	168.2340504	206.315926	148.6748189
40.29320354	93.922827	159.9586903	386.0631099	312.5998878	512.9848714
0	0	0	1.944902317	1.041999626	5.674611409
179.6859077	91.94550432	81.77663382	70.01648341	86.48596897	90.79378255
0	7.909290694	7.189154622	7.779609268	0	3.404766845
84.31080589	431.2936216	343.2821332	1012.652289	211.1820642	372.4247468
140.4817096	262.9839156	180.6275099	347.1650636	171.9299383	264.4368917
377.8849089	200.6982514	202.1949737	279.0934825	315.7258867	160.0240417
103.4555226	311.4283211	207.5868397	356.8895752	165.6779406	397.2227986
43.56022004	48.4444055	156.364113	67.09912993	59.39397869	54.47626953
175.3298857	314.3943051	391.8089269	234.3607292	253.2059091	173.6431091
1157.612848	328.2355638	192.3098861	1085.255493	932.5896654	743.3740946
8524.735062	4502.363728	2887.344225	4642.48183	6282.215746	8908.00499
96.92148959	76.12692293	92.56036576	58.34706951	114.6199589	89.65886026
6.534033006	0.988661337	0.898644328	0.972451158	2.083999252	0
19.60209902	0.988661337	2.695932983	0	1.041999626	2.269844564
149.1937536	61.29700288	50.32408235	46.67765561	108.3679611	53.34134725

35.93718153	20.76188807	5.391865966	0.972451158	0	5.674611409
8.712044008	0	0	2.917353475	0	0
264.6283367	218.4941554	221.9651489	195.4626828	181.3079349	214.5003113
7.623038507	156.2084912	31.45255147	253.8097524	161.509942	71.50010376
678.4504271	288.6891103	40.43899475	131.2809064	426.1778471	232.6590678
1288.293508	2239.317928	2657.291277	2550.739389	2293.441177	2865.678762
2.178011002	0	0	0	0	0
2.178011002	0	0	0	0	0
5.445027505	0.988661337	0	0	1.041999626	0
14.15707151	2.96598401	0.898644328	0	0	0
123.0576216	149.2878619	148.2763141	499.8398954	152.1319454	198.6113993
169.8848582	540.7977512	1932.085305	716.6965038	701.2657484	447.159379
78.40839607	92.93416566	25.16204118	19.44902317	51.05798168	54.47626953
160.0838087	353.9407586	215.6746387	260.6169105	206.315926	229.2543009
33.75917053	57.34235754	146.4790254	47.65010676	40.63798542	170.2383423
52.27226405	147.3105392	142.8844481	117.6665902	91.6959671	77.17471516
4.356022004	5.931968021	13.47966492	10.69696274	10.41999626	5.674611409
129.5916546	46.46708283	15.27695357	21.39392549	91.6959671	29.50797933
78.40839607	129.5146351	99.74952038	101.1349205	82.31797046	124.841451
426.8901564	257.0519476	235.4448139	218.8015107	340.7338777	354.0957519
622.9111466	249.1426569	474.484205	412.3192912	369.9098673	557.2468404
3.267016503	13.84125872	26.0606855	1.944902317	14.58799477	30.64290161
42.47121454	95.90014967	407.9845248	49.59500908	106.2839619	52.20642496
595.6860091	237.2787208	91.66172143	219.7739618	452.2278377	292.8099487
19.60209902	84.03621363	172.5397109	19.44902317	93.77996635	66.96041463
301.6545238	613.9586902	1975.220232	4269.060586	931.5476658	309.8337829
371.5360068	280.3151488	885.2725001	567.5516696	486.0198856	1154.261358
0	0	0	0	0	0
0	0	0	0	0	0
38.11519254	51.41038951	78.18205651	101.1349205	54.18398056	36.31751302
2.178011002	0	0	0	0	0
42.47121454	14.82992005	6.290510294	1.944902317	4.167998504	1.134922282
219.9791112	272.870529	436.7411433	632.093253	266.7519043	256.4924357
19.60209902	96.88881101	94.35765441	132.2533576	158.3839432	360.9052856
1185.230027	1048.771946	2933.974879	3764.805762	1564.343619	2063.70863
6.534033006	28.67117877	0.898644328	155.5921854	12.50399551	6.809533691
0	0	1.797288655	0	2.083999252	1.134922282

10.89005501	14.82992005	19.77017521	20.42147433	34.38598766	19.29367879
13644.14992	7984.428956	1837.72765	6000.996099	11028.52404	5742.706746
0	0	0.898644328	0.972451158	3.125998878	1.134922282
58.80629706	552.6616873	248.0258345	119.6114925	59.39397869	98.73823852
714.3876087	936.262286	868.0904206	2525.455659	910.7076732	1425.462386
9624.630618	4658.572219	3148.849724	4933.244727	6439.557689	7838.908201
8.712044008	61.29700288	40.43899475	51.5399114	20.83999252	14.75398966
1.089005501	86.0135363	8.986443277	19.44902317	2.083999252	12.4841451
2.178011002	0	0	0.972451158	0	1.134922282
265.7173423	69.20629358	42.2362834	32.09088823	160.4679424	85.11917114
6.534033006	3.954645347	2.695932983	0	2.083999252	2.269844564
4.356022004	6.920629358	6.290510294	7.779609268	4.167998504	0
0	0	0	0	0	0
0	0	0	0	0	0
211.2670672	525.6514596	437.6397876	588.3329509	308.4318893	215.6352335
0	0	1.797288655	23.3388278	0	0
356.1047988	98.86613368	163.5532676	105.9971763	212.5679237	342.7465291
291.8534743	403.3738254	843.8270237	556.2420626	360.5318706	502.7705708
1.089005501	0	0	0	0	0
11.97906051	42.51243748	58.4118813	34.03579055	15.62999439	13.61906738
2.178011002	0	0	0	0	0
0	2.96598401	62.00645861	1.944902317	0	0
34.84817603	83.04755229	1223.05493	92.38286005	132.3339525	128.2462178
1.089005501	8.897952031	1.797288655	78.76854384	8.335997009	2.269844564
340.8587218	176.9703793	60.20916996	168.2340504	242.7859129	155.4843526
10.89005501	2.96598401	0	0	0	3.404766845
3551.246939	2536.90499	991.2046935	1583.150486	3466.732756	4739.435449
128.5026491	259.0292702	476.2814937	374.393696	363.6578695	165.6986531
276.6073973	334.1675318	475.3828494	458.9969468	333.4398804	541.3579284
7.623038507	23.72787208	27.85797416	17.50412085	16.67199402	26.10321248
225.4241387	328.2355638	141.0871595	841.1702521	237.5759148	196.3415548
516.1886075	779.0651334	1576.222151	1309.89171	795.0457147	680.9533691
0	1.977322674	2.695932983	0	0	1.134922282
147.0157426	242.2220275	352.2685765	393.8427192	287.5918968	279.1908813
551.0367835	301.5417077	189.6139532	97.24511585	279.2558998	90.79378255
138.3036986	240.2447048	442.1330092	184.7657201	204.2319267	347.2862182
0	0.988661337	2.695932983	0.972451158	0	1.134922282

99.3390818	130.1276052	370.5110563	186.8953881	144.0043483	125.760738
238.4922047	284.734465	72.79019055	51.5399114	191.7279312	61.28580322
21.78011002	3.954645347	8.986443277	0	5.209998131	3.404766845
10.89005501	8.897952031	8.08779895	9.724511585	12.50399551	10.21430054
8.712044008	81.07022962	18.87153088	39.8704975	33.34398804	12.4841451
732.9007022	435.9996495	394.5048599	454.134691	569.9737955	458.5086019
7.623038507	2.96598401	0	0	0	0
0	7.909290694	12.58102059	1.944902317	2.083999252	1.134922282
18.51309352	40.53511481	133.8980048	16.53166969	10.41999626	55.61119181
8.712044008	16.80724273	19.77017521	39.8704975	18.75599327	30.64290161
339.7697163	156.2084912	125.8102059	106.9696274	215.6939226	290.5401041
5.445027505	0.988661337	2.695932983	0.972451158	4.167998504	1.134922282
2802.011154	1677.758289	558.9567718	1729.990611	2285.10518	1969.090159
7.623038507	26.69385609	15.27695357	12.64186506	2.083999252	14.75398966
1600.838087	927.3643339	495.1530246	927.7184052	1152.451586	834.1678771
165.5288362	21.75054941	131.2020718	0	108.3679611	1.134922282
1.089005501	0	0	0	1.041999626	1.134922282
4.356022004	0	0	0	1.041999626	0
111.0785611	56.3536962	26.95932983	107.9420786	52.09998131	167.9684977
494.4084975	906.6024459	1277.872234	1053.164605	785.6677181	828.4932657
67.51834106	71.18361625	212.9787057	87.52060426	71.8979742	115.7620727
204.8092646	363.9756711	642.2251553	504.7604983	349.5700346	725.6239101
7.623038507	25.70519476	17.07424223	36.95314402	9.377996635	12.4841451
14.15707151	1.977322674	3.594577311	4.862255792	3.125998878	4.539689127
918.2276584	514.2818542	559.1275143	254.3251515	336.4199993	160.3872169
15.24607701	6.920629358	2.695932983	2.917353475	1.041999626	1.134922282
717.6546252	2573.48546	5635.398579	4678.462523	2868.624971	4876.761045
2.178011002	0	0	0	0	0
7484.734809	4181.048793	3103.018864	2677.158039	4299.290457	8813.806441
490.0524755	8434.269864	2175.617917	3656.416356	1093.057608	1150.811194
0	0	5.391865966	0	1.041999626	0
1.089005501	4.943306684	4.493221639	11.6694139	1.041999626	2.269844564
6.534033006	0	2.695932983	2.917353475	1.041999626	2.269844564
31.58115953	61.29700288	77.28341218	28.2010836	28.13398991	83.98424886
920.2096484	1342.602095	2103.726371	739.0628804	1258.735548	1730.75648
2144.251832	6953.255182	5059.367565	4866.145597	5272.518108	2913.345497
15.24607701	171.0384113	36.84441744	26.25618128	28.13398991	27.23813476

4.356022004	2.96598401	0	0	1.041999626	1.134922282
184.0419297	122.5940058	430.450633	714.7516015	429.303846	528.8737833
1.089005501	0.988661337	0	0	0	0
710.0315867	992.1018783	2359.840005	2156.770251	1022.868513	1698.377147
13.06806601	28.67117877	43.13492773	23.3388278	33.34398804	55.61119181
441.0472279	841.3507976	4725.97052	2117.026172	877.3636852	1386.875028
65.34033006	109.7414084	185.1207315	157.5370877	70.85597458	112.3573059
63.16231906	41.52377615	26.0606855	10.69696274	38.55398617	14.75398966
53781.62567	25696.29681	7899.083641	27721.66517	39544.92781	55827.96197
0	5.931968021	20.66881954	0	11.46199589	54.47626953
417.0891069	648.5618369	672.1859571	909.2418332	830.473702	977.1680847
296.2094963	374.7026467	617.3686532	441.4928259	334.48188	485.7467366
130.6806601	263.9725769	266.8973653	396.7600727	301.1378919	253.0876688
172.0628692	267.9272223	480.7747153	340.3579055	179.2239357	282.5956482
169.8848582	203.6642354	313.6268704	386.0631099	220.9039207	242.8733683
1.089005501	1.977322674	5.391865966	2.917353475	2.083999252	4.539689127
1.089005501	0.988661337	2.695932983	4.862255792	1.041999626	13.61906738
13.06806601	46.46708283	58.4118813	19.44902317	26.04999065	9.079378255
101.2775116	73.16093892	397.2007929	229.4984734	255.2899084	484.6118143
290.7644688	136.4352645	111.4318966	108.9145297	182.3499346	158.8891195
3.267016503	26.69385609	28.75661849	16.53166969	3.125998878	10.21430054
0	0	1.797288655	1.944902317	0	1.134922282
39.20419804	108.752747	329.8024683	192.5453294	16.67199402	242.8733683
9.801049509	57.34235754	216.573283	153.647283	135.4599514	130.5160624
279.8199635	424.9859622	517.340553	282.9832871	342.817877	772.6437402
43.56022004	47.45574417	75.48612353	232.4158269	23.9659914	35.18259074
98.09761553	149.0505831	195.5450057	152.6164848	110.0976805	125.1592292
3.267016503	13.84125872	115.9251183	24.31127896	9.377996635	5.674611409
227.6021497	117.6506991	144.6817368	70.98893457	150.0479462	233.7939901
102.3665171	207.6188807	478.0787824	176.0136597	145.8799477	308.6988607
88.20944558	150.2765232	249.8231231	117.6665902	93.77996635	131.6509847
739.4347352	245.1880115	241.7353242	244.0852408	433.4718445	258.7622803
5.445027505	5.931968021	25.16204118	9.724511585	11.46199589	5.674611409
15.24607701	52.39905085	35.04712878	42.78785097	33.34398804	32.91274617
112.1675666	147.3105392	228.2556592	265.4791663	211.5259241	194.0717102
2560.012352	459.6780886	2.300529479	0	209.9837647	426.5037935
248.2932542	340.0994999	584.118813	485.2531281	280.2978994	375.6592753

41.38220904	141.3785712	57.51323697	133.2258087	43.7639843	93.06362711
37.02618703	38.55779214	0.898644328	11.6694139	54.18398056	15.88891195
33.75917053	43.50109882	44.93221639	68.07158109	36.46998691	36.31751302
1514.806652	1212.098799	209.3841284	1300.167199	924.2536684	640.096167
0	4.943306684	0.898644328	0	2.083999252	4.539689127
556.481811	867.0559924	829.4487145	1284.60798	745.0297327	736.5645609
231.3483286	339.8325613	402.2242146	358.2801803	306.0144502	316.0191094
28.12901209	134.5172615	27.03122138	80.48005787	89.39314792	57.9945286
418.1781124	168.0724273	23.36475252	93.35531121	408.4638534	34.04766845
0	0.988661337	0	10.69696274	2.083999252	3.404766845
3.267016503	6.920629358	15.27695357	18.47657201	4.167998504	9.079378255
7.623038507	2.96598401	686.5642664	650.569825	0	0
496.5865085	701.9495491	1342.574626	851.8672148	819.0117061	629.8818664
197.1099957	122.5940058	138.3912265	82.65834847	125.0399551	161.158964
0	0.988661337	0	0.972451158	0	0
2180.189013	1260.543204	2030.037536	1490.767626	1320.213526	1582.081661
0	1.977322674	1.797288655	0.972451158	0	1.134922282
203.6440287	275.836513	394.5048599	298.5425056	252.1639095	379.0640421
72.35352549	140.36025	155.0341194	278.4419402	135.1056715	126.4870883
531.4346845	632.7432556	695.5507097	692.3852248	607.485782	426.730778
0	26.69385609	0.898644328	29.17353475	39.59598579	11.34922282
7.623038507	3.954645347	27.85797416	4.862255792	3.125998878	3.404766845
3.267016503	0.988661337	0	0.972451158	0	0
0	1.977322674	6.290510294	0.972451158	0	4.539689127
177.5078967	199.70959	438.5384319	504.7021512	187.5599327	424.4609334
485.6964535	673.2783704	1073.879972	1048.302349	565.805797	456.2387573
3.267016503	66.24030957	17.07424223	11.6694139	5.209998131	24.9682902
840.7122468	450.8295696	235.4448139	426.9060586	562.6797981	388.1434204
2.820524248	3.954645347	0	0.486225579	0	0
64.25132456	76.12692293	147.3776697	133.2258087	132.3339525	102.1430054
5.39057723	0	0	0	0.385539862	0
16.33508252	2.96598401	10.78373193	2.917353475	7.293997383	6.809533691
91.47646209	241.2333662	175.1996981	150.7299296	181.255835	88.52393798
25.01445636	15.81858139	12.58102059	8.752060426	10.41999626	35.18259074
278.7854083	1108.289359	1314.716651	1024.963521	852.3556942	351.8259074
1317.696656	2306.546899	4803.253932	2946.52701	2226.753201	1566.192749
2.178011002	0.988661337	28.75661849	8.752060426	1.041999626	11.34922282

55.53928055	103.8094404	186.0193758	168.2340504	93.77996635	90.79378255
0	0	0	0	0	0
0	4.063398094	8.08779895	26.25618128	4.167998504	4.539689127
178.5969022	193.777622	478.0787824	289.7904452	195.8959297	140.7303629
221.0681167	91.94550432	106.0400307	78.76854384	96.90596523	161.158964
38.11519254	12.85259738	5.391865966	11.6694139	34.38598766	13.61906738
129.5916546	330.2128865	538.2879523	212.9668037	250.0799103	354.0957519
1.121675666	0	0	0	0	0
72.94158846	82.71140744	237.2600754	187.177399	68.74071534	126.7594697
52.27226405	24.71653342	0.898644328	2.917353475	1.041999626	0
1283.937486	1714.338758	5220.2249	1233.068069	1993.345285	6977.502189
69.69635207	110.7300697	6.290510294	14.58676738	16.67199402	23.83336792
2660.168188	1460.38132	1035.966167	1336.809159	1476.00289	2222.370765
65.34033006	97.87747234	110.5332523	60.29197182	116.7039581	139.5954407
54.45027505	18.7845654	2.695932983	0.972451158	43.7639843	20.74637931
84.94242908	30.64850144	5.391865966	61.26442298	31.25998878	14.75398966
257.0052982	167.0837659	213.87735	70.98893457	128.165954	187.2621765
144.8377316	204.6528967	222.8637933	188.6555247	215.6939226	233.7939901
1188.105002	424.1357135	1044.224709	992.8726328	334.48188	342.7465291
286.4084468	582.3215274	667.6927355	975.3685119	418.8838497	406.3021769
1184.620184	732.3113388	132.9993605	369.5314402	457.052296	384.7386535
0	0	9.885087605	6.807158109	0	1.134922282
68.60734656	226.4034461	284.8702519	311.1843707	86.48596897	161.158964
4.356022004	2.96598401	0	0	1.041999626	0
516.3955185	290.4588141	151.6642032	144.2631294	220.9039207	504.6885895
5.445027505	22.73921075	35.04712878	16.53166969	15.62999439	30.64290161
130.6806601	100.8434564	48.5267937	34.03579055	80.23397121	107.8176168
35.93718153	44.48976016	79.08070084	70.98893457	81.27597084	65.82549235
231.9581717	482.4667324	554.4635502	759.4843548	478.2778284	431.2704671
139.3927041	761.2692293	2160.340964	56.40216719	235.4919155	758.1280843
25.04712652	21.75054941	8.08779895	8.752060426	18.75599327	11.34922282
13.06806601	32.62582411	10.78373193	538.7379418	9.377996635	14.75398966
1351.455827	796.8610375	436.7411433	695.3025783	954.4716575	1492.422801
56.62828605	149.2878619	150.0736027	77.79609268	96.90596523	121.4366842
2.178011002	11.86393604	18.87153088	5.834706951	11.46199589	6.809533691
13.06806601	27.68251743	41.33763908	27.22863244	15.62999439	31.77782389
206.9110452	456.7615376	472.6869164	300.487408	390.7498598	836.4377217

51.18325855	48.4444055	88.06714412	102.1073716	59.39397869	47.66673584
742.7017517	457.7501989	569.7405038	449.2724352	417.8418501	844.3821777
21.78011002	10.8752747	8.08779895	22.36637664	17.71399364	5.674611409
5.445027505	0	8.986443277	8.752060426	4.167998504	9.079378255
120.8796106	129.5146351	200.3976851	529.0134302	140.6699495	296.2147156
5.445027505	0.988661337	0	0	0	0
0	0.988661337	1.797288655	1.944902317	0	3.404766845
33.75917053	7.909290694	0.898644328	0.972451158	4.167998504	2.269844564
1.089005501	3.954645347	1.797288655	3.889804634	2.083999252	2.269844564
1796.859077	651.527821	899.5429721	1163.051586	1345.221517	743.3740946
135.0366821	288.6891103	352.2685765	422.0438028	244.8699121	342.7465291
1170.680914	1558.130267	2514.406829	2884.290136	1520.277455	2221.042906
199.2880067	281.768481	311.8295817	369.5314402	356.3638721	392.6831095
211.2670672	443.9089402	685.6656221	310.2119195	384.497862	287.1353373
0	0	1.797288655	12.64186506	0	3.404766845
4805.781276	3374.301143	2634.825169	2612.003812	3713.686667	2800.988192
2.178011002	42.51243748	22.46610819	20.42147433	6.251997757	23.83336792
1153.256826	1001.513934	242.6339685	1049.2748	612.6957802	372.2545084
15.24607701	54.37637352	2051.605	52.51236256	9.377996635	12.4841451
303.8325348	371.7366626	779.1246321	635.9830576	363.6578695	309.8337829
3247.599535	973.8808498	0	0	77.60813215	121.6636686
2.178011002	8.897952031	9.885087605	15.55921854	0	1.134922282
53.36126955	61.29700288	110.5332523	129.3360041	57.30997944	70.36518147
1153.256826	1380.171226	2576.413288	2028.533117	1543.201446	1126.977826
301.6545238	273.8591903	707.2330859	497.8949931	428.2618463	274.6511922
10.89005501	34.60314679	37.74306176	45.70520445	15.62999439	11.34922282
1939.518797	900.6704778	1392.898708	742.9526851	1661.989404	1492.422801
1645.421972	6211.185756	5740.360237	11021.42107	5815.639573	3638.379248
3226.7233	5068.866674	12154.16453	6729.362017	5372.550072	5126.443947
44.64922554	97.87747234	244.4312571	71.96138573	73.98197345	202.0161662
15.24607701	47.45574417	36.84441744	60.29197182	58.35197906	23.83336792
1308.440109	2084.394696	2687.279038	1172.202351	1811.91231	2135.560559
829.3212493	1166.343552	1694.951039	1600.761576	1371.844608	1186.765532
38.11519254	115.6733764	148.2763141	126.4186506	110.4519604	38.58735758
408.2790524	295.856905	1023.052648	639.0365543	458.4381555	1314.85286
142.6597206	229.3694301	407.9845248	223.6637664	176.0979368	217.9050781
248.2932542	517.0698792	542.7811739	482.3357746	533.5038086	351.8259074

5.445027505	1.977322674	0.898644328	4.862255792	1.041999626	0
6.534033006	26.69385609	26.95932983	12.64186506	8.335997009	41.99212443
403.5309884	437.9769722	178.8302212	181.8483666	298.0118931	111.2223836
119.7906051	87.00219764	40.44798119	49.59500908	47.9319828	96.46839396
59.89530256	35.59180813	11.68237626	26.25618128	32.30198841	19.29367879
483.5184425	936.262286	2028.240248	1295.304943	743.987733	716.1359598
11.97906051	41.52377615	61.10781429	33.06333939	41.67998504	62.4207255
463.9163434	609.0153835	1181.717291	853.8121171	547.0498037	644.6358561
4291.77068	2210.646749	2336.475252	1750.412085	2820.692988	2835.03586
21.78011002	4.943306684	0	0	0	4.539689127
100.1885061	206.6302194	246.2285458	248.9474966	162.5519417	205.420933
56.62828605	102.820779	128.5061389	117.6665902	96.90596523	112.3573059
42.47121454	93.922827	206.6881954	169.2065016	55.22598018	45.39689127
5805.009164	18241.65191	36617.01549	13891.00775	6103.80457	24260.96124
1.089005501	131.4919578	691.057488	444.4101794	70.85597458	6.809533691
99.09950059	158.1858139	156.364113	138.0880645	185.4759334	65.82549235
170.9738637	220.4714781	727.0032611	286.8730917	237.5759148	382.468809
0	0	1521.404847	337.440552	2.083999252	2.269844564
35.93718153	18.7845654	0	4.862255792	18.75599327	17.02383423
114.3455776	133.4692805	230.9515922	195.4626828	290.7178957	217.9050781
8617.30053	5033.274866	843.8270237	2377.643082	7291.913384	8498.298046
157.9057976	117.6506991	44.03357206	94.32776237	145.8799477	47.66673584
4.356022004	66.24030957	18.87153088	34.03579055	12.50399551	3.404766845
228.6911552	201.6869127	83.57392248	122.528846	251.1219099	175.9129537
151.3717646	231.3467528	521.2137101	364.6691844	165.6779406	128.2462178
0	3.954645347	6.290510294	6.807158109	2.083999252	6.809533691
94.74347859	202.675574	184.2220872	157.5370877	131.2919529	173.6431091
193.8429792	244.1993502	542.7811739	568.8839277	262.5839058	183.8574097
45.73823104	82.05889096	2.695932983	0.972451158	3.125998878	1.134922282
111.0785611	155.2198299	173.4383553	164.3442458	125.0399551	219.0400004
29509.87107	21954.21365	6614.920896	192.5453294	19082.13915	12744.0423
52.27226405	97.87747234	138.3912265	185.7381713	123.9979555	264.4368917
105.6335336	140.3899098	163.5532676	189.6279759	144.837948	155.4843526
1.089005501	26.69385609	6.290510294	21.39392549	3.125998878	5.674611409
30.96042639	136.1485527	159.6261919	156.3993198	0	104.2199131
4640.25244	2093.984711	229.1543036	2952.361717	3516.748738	1563.922904
326.7016503	411.2831161	486.1665813	378.2835006	507.4538179	331.3973063

5.445027505	0	6.290510294	0	0	1.134922282
16.33508252	42.51243748	50.32408235	27.22863244	42.72198467	18.15875651
142.6597206	82.05889096	79.08070084	21.39392549	60.43597831	17.02383423
69.69635207	68.21763224	14.37830924	38.89804634	34.38598766	49.9365804
275.5183918	291.6550944	948.0697658	693.357676	324.0618837	264.4368917
9.801049509	19.77322674	42.2362834	65.15422762	7.293997383	21.56352335
118.7015996	66.24030957	93.45901008	76.82364152	111.49396	47.66673584
123.0576216	150.2765232	195.9044634	459.969398	283.4238983	433.5403117
21.78011002	37.5691308	57.51323697	65.15422762	39.59598579	51.07150268
37.02618703	65.25164823	4.25058767	97.24511585	91.6959671	10.21430054
1.089005501	0	0	0	1.041999626	0
2.450262377	8.897952031	5.391865966	11.6694139	11.46199589	17.02383423
1605.194109	1165.631716	467.2950504	249.9199477	1088.889609	628.7469441
6.534033006	2.96598401	0	0	0	2.269844564
16.47665323	5.931968021	0	0	0	0
5.847959541	37.99425517	28.99925246	30.66138503	6.700057596	14.16383008
7.623038507	20.76188807	23.36475252	26.25618128	27.09199028	21.56352335
0	58.33101887	13.47966492	6.807158109	0	152.0795858
26.13613202	40.53511481	54.81730399	55.42971603	32.30198841	27.23813476
0	0	1.797288655	0.972451158	0	1.134922282
115.4345831	298.5757237	571.5377924	486.2255792	365.7418688	1358.501971
66.42933556	130.5032965	165.3505563	186.7106224	98.98996448	137.3255961
3.267016503	10.8752747	206.6881954	12.64186506	3.125998878	1.134922282
4893.990722	3898.291651	3815.643816	2965.976033	4702.544313	3593.163944
22.86911552	318.3489505	130.3034275	104.052274	60.43597831	32.91274617
147.0157426	193.777622	653.3144263	542.6277464	114.6199589	182.7224874
1056.335336	252.1086409	1.797288655	201.2973898	855.481693	476.6673584
3.267016503	5.931968021	20.66881954	7.779609268	3.125998878	11.34922282
90.38745659	135.4466031	143.7830924	123.5012971	161.509942	170.2383423
10.89005501	22.73921075	17.07424223	11.6694139	14.58799477	10.21430054
15057.67906	9865.85148	8022.197914	1948.792122	13554.33114	12252.62095
129.5916546	209.5962034	364.8495971	259.6444593	189.643932	133.9208293
349.5707658	610.0040448	485.267937	627.2309972	606.4437824	355.2306742
561.9268385	296.598401	444.8289422	215.8841572	444.9338404	324.5877726
18.51309352	51.41038951	30.55390714	26.25618128	40.63798542	48.80165812
254.8272872	333.1788705	1063.09624	288.8179941	321.9778845	562.9214518
1.089005501	9.886613368	12.58102059	29.17353475	9.377996635	0

384.4189419	218.4941554	183.3234429	187.6830736	302.1798916	272.3813476
1.089005501	0	0	0.972451158	0	0
1.089005501	0	0	0	1.041999626	0
2.178011002	0	0	0	0	1.134922282
1.089005501	0	0	0	1.041999626	0
363.7278373	212.5621874	216.573283	419.1264493	275.0879013	247.4130574
150.2827591	89.96818165	97.05358739	67.09912993	157.3419435	115.7620727
0	0	0	0.972451158	2.083999252	1.134922282
1.089005501	4.943306684	6.290510294	0	3.125998878	2.269844564
102.3665171	123.5826671	172.5397109	160.4544411	156.2999439	119.1668396
106.7225391	96.88881101	173.4383553	125.4461994	135.4599514	144.1351298
327.7906558	113.6960537	62.90510294	159.48199	135.4599514	199.7463216
50.09425305	42.51243748	37.74306176	63.2093253	40.63798542	45.39689127
138.3036986	43.50109882	8.986443277	14.58676738	37.51198654	27.23813476
197.4584774	51.41038951	0	53.48481372	80.80707101	0
0	0	0	5.834706951	0	0
155.7277866	261.9952543	350.4712878	305.3496638	282.3818987	181.5875651
31.58115953	100.8434564	301.0458498	37.92559518	89.61196785	41.99212443
19.60209902	8.897952031	35.94577311	35.98069286	38.55398617	34.04766845
90.38745659	124.5713284	211.181417	146.8401249	106.2839619	163.4288086
2.178011002	0.988661337	0	0	0	0
1919.851358	1587.790107	356.7617981	1061.868042	2135.036394	987.3483375
31.58115953	1.977322674	2.695932983	11.6694139	10.41999626	3.404766845
927.8326869	750.3939546	339.6875559	277.1485802	519.9578134	431.2704671
430.1571729	197.7322674	176.1342882	198.3800363	301.1378919	351.8259074
10.89005501	4.943306684	0.898644328	4.862255792	0	0
66.42933556	112.7073924	127.6074945	78.76854384	106.2839619	123.7065287
961.5918574	2210.646749	2108.219593	2349.441999	1800.575354	898.8584472
117.6125941	3.954645347	2.695932983	0	22.92399177	48.80165812
46.82723654	97.87747234	145.5803811	86.5481531	54.18398056	80.57948201
1.089005501	1.977322674	8.08779895	0.972451158	2.083999252	10.21430054
3955.26798	2807.798197	957.9548534	3109.898805	3191.644855	2024.701351
4.356022004	0	0	0	0	0
633.8012016	418.2037455	478.0787824	260.6169105	581.4357914	364.3100525
321.2566228	498.2853138	515.8218441	547.4900022	487.655825	417.6513997
576.08391	1662.928369	1436.033636	1579.260681	1749.517372	969.2236287
21.78011002	24.71653342	78.18205651	39.8704975	21.88199215	24.9682902

88.20944558	86.0135363	111.4318966	94.32776237	95.8639656	173.6431091
91.47646209	72.17227759	180.6275099	117.6665902	88.56996822	107.8176168
41.38220904	27.68251743	18.87153088	50.56746024	55.22598018	15.88891195
35.93718153	6.920629358	0.898644328	14.58676738	9.377996635	1.134922282
4.356022004	1.977322674	0.898644328	0	4.167998504	1.134922282
152.4607701	313.4056438	232.7488809	414.2641935	311.5578882	174.7780314
314.7225898	317.3602891	836.6378691	410.3743889	445.97584	551.572229
1252.356326	599.1287701	315.424159	805.1895592	864.8596897	661.6596903
0	16.80724273	3.594577311	0.972451158	2.083999252	1.134922282
0	0	0	0	0	0
0	0	0	0	0	0
10003.60453	2722.773322	810.5771836	5353.343627	1710.963386	2873.623218
0	3.104396598	25.70122777	11.03732065	0	0
3.267016503	0	0	0	0	0
438.8692169	176.9703793	336.9916229	260.6169105	252.1639095	241.738446
0	0	4.493221639	8.752060426	0	1.134922282
178.5969022	256.0632862	291.1607622	201.2973898	187.5599327	196.3415548
748.1467792	432.0450042	298.3499168	359.8069286	527.2518108	198.6113993
242.8482267	297.5870624	154.5668244	210.0494502	144.837948	225.8495341
1.089005501	0.988661337	1.797288655	0	2.083999252	0
117.6125941	178.947702	296.5526282	301.4598591	164.6359409	136.1906738
0	0.988661337	0	1.944902317	1.041999626	1.134922282
65.34033006	309.4509984	226.4583706	176.0136597	117.7459578	152.0795858
43.56022004	159.1744752	299.2485611	263.5342639	158.3839432	81.71440429
0	0.988661337	153.66818	0.972451158	0	2.269844564
0	0	0	0	0	0
532.52369	295.6097397	93.45901008	260.6169105	162.5519417	397.2227986
0	0	0	0	0	0
41.04461733	50.55025415	116.6709931	77.07647882	37.41820657	93.29061157
18117.42515	11865.57722	2451.241119	422.0438028	9584.156261	27020.0935
120.8796106	155.2198299	244.4312571	118.6390413	214.651923	178.1827982
1.110785611	0	0	0	0	0
1.089005501	0	0	0	0	0
1.089005501	0	0	0	0	0
0	2.96598401	0.898644328	341.3303566	0	0
450.8482774	850.2487497	1509.722471	1102.759614	664.7957615	533.4134725
80.58640708	195.7549447	174.3369996	238.2505338	140.6699495	174.7780314

200.3770122	317.3602891	473.5855607	310.2119195	329.2718819	299.6194824
242.8482267	103.8094404	124.0129172	121.5563948	172.9719379	332.5322286
10.89005501	10.8752747	3.594577311	104.052274	68.77197532	24.9682902
0	0	0	0	0	0
235.2251882	215.5281714	746.7734363	692.3852248	435.5558437	345.0163737
2.178011002	3.954645347	2.695932983	8.752060426	3.125998878	2.269844564
9609.384541	5148.948242	6157.510934	4657.068598	6212.401771	7599.439599
14.15707151	2.96598401	5.391865966	0.972451158	1.041999626	4.539689127
1319.874667	627.7999489	736.8883487	879.0958472	1140.989591	760.3979288
0	0	0	0	0	0
0	0	0	0	0	0
0	0	0	0	0	0
0	0	0	0	0	0
3.267016503	0.988661337	0	0	0	0
4.356022004	30.64850144	31.45255147	27.22863244	28.13398991	32.91274617
2.178011002	8.779312671	8.671917763	26.20755872	5.262098112	4.074370992
2.178011002	52.39905085	6.290510294	9.724511585	11.46199589	1.134922282
465.1360296	385.6866741	1084.088571	631.713997	381.6427831	1540.305172
50.09425305	69.20629358	162.6546233	169.2065016	40.63798542	78.30963745
15.24607701	8.897952031	10.78373193	1.944902317	6.251997757	1.134922282
69.69635207	30.64850144	34.14848445	45.70520445	67.7299757	56.74611409
6104.964839	3459.326018	332.4984013	2061.596456	5349.62608	3576.14011
14.15707151	3.954645347	0	10.69696274	4.167998504	5.674611409
20.69110452	3.954645347	10.78373193	0.972451158	8.335997009	5.674611409
297.2876117	62.63169569	272.5767975	28.67758466	34.89656748	301.7531363
1.089005501	8.897952031	78.18205651	2.917353475	2.083999252	1.134922282
1202.262073	1039.083065	527.5042204	847.9774102	710.643745	572.00083
3001.299161	1376.216581	1970.727011	953.0021353	1625.519417	1939.58218
7.623038507	24.71653342	18.87153088	17.50412085	18.75599327	32.91274617
42.47121454	478.512087	197.7017521	270.3414221	44.80598392	93.06362711
237.4031992	104.7981017	143.7830924	116.694139	151.0899458	108.9525391
0	0	0	0	0	0
0	0	0	0	0	0
822.1991533	1489.912635	2062.388732	985.0930235	2095.461248	2734.027777
1141.277765	719.7454532	533.7947307	614.5891321	1035.747628	1036.184043
1.089005501	0	0.898644328	0	0	1.134922282
150.2827591	76.12692293	70.09425756	52.51236256	97.94796486	26.10321248

8.712044008	1.977322674	1.797288655	1.944902317	8.335997009	3.404766845
1.089005501	0	0	0	0	0
1.089005501	0	0	0	0	0
1.089005501	0	0	0	0	0
1.089005501	0	0	0	0	0
1.089005501	0	0	0	0	0
1.089005501	0	0	0	0	0
1.089005501	0	0	0	0	0
0	0.978774723	2.048909067	5.251236256	3.480278751	2.269844564
2.178011002	14.82992005	2.695932983	2.917353475	3.125998878	1.134922282
185.1309352	290.666433	473.5855607	173.0963062	264.667905	284.8654927
613.1100971	423.1470522	6.290510294	204.2147433	262.5839058	55.61119181
89.29845108	87.00219764	33.24984013	44.73275329	57.30997944	38.58735758
25.04712652	121.6053444	74.5874792	102.1073716	28.13398991	39.72227986
5.445027505	14.82992005	13.47966492	91.41040889	67.7299757	41.99212443
50.09425305	84.03621363	250.7217674	105.9971763	55.22598018	146.4049744
0	0	0	0.972451158	0	0
1.089005501	0	0	0	0	0
1105.340584	648.5618369	280.3770303	433.7132167	1122.233597	1348.287671
2.178011002	4.943306684	131.2020718	0	7.293997383	3.404766845
323.4346338	250.1313182	494.2543803	504.7021512	326.114623	178.1827982
0	1.977322674	0.898644328	3.889804634	1.041999626	0
310.3665678	217.5054941	185.1207315	154.6197342	149.0059465	44.26196899
11.97906051	40.53511481	39.54035042	31.11843707	12.50399551	30.64290161
46.82723654	66.24030957	76.38476786	107.9420786	64.60397682	87.3890157
86.03143458	192.7889607	169.8437779	187.6830736	154.2159447	80.57948201
11.97906051	0	0	4.862255792	0	0
5.445027505	16.80724273	17.97288655	10.69696274	4.167998504	783.0963745
1520.251679	897.7044938	699.145287	492.0602862	986.7736459	1585.486428
390.9529749	709.8588398	1838.626295	791.575243	609.5697813	554.9769958
0	0	0	0	0	0
0	0	0	0	0	0
0	0	0	0	0	0
0	0	0	0	0	0
62.07331356	197.7322674	237.2421025	217.8290595	186.5179331	211.0955444
137.2146931	72.12284452	97.05358739	52.51236256	90.65396747	90.70298876
0	1.364352645	0	4.444101794	0	0

9.801049509	39.54645347	9.885087605	21.39392549	14.58799477	15.88891195
299.4765128	628.7886102	984.0155389	866.4539822	615.821779	646.9057006
22.86911552	108.752747	22.46610819	108.9145297	39.59598579	19.29367879
70.78535757	251.1199796	123.1142729	165.3166969	113.5779592	77.17471516
733.9897077	394.4758734	275.8838086	276.176129	263.6259054	161.158964
103.4555226	23.72787208	26.0606855	52.51236256	48.97398243	70.36518147
74.05237407	283.7458037	73.68883487	132.2533576	489.7398243	112.3573059
0	0	5.391865966	0.972451158	1.041999626	0
393.1309859	371.7366626	735.9897044	534.8481372	561.6377985	507.31026
20.69110452	34.60314679	47.62814937	87.52060426	30.21798916	163.4288086
955.0578244	260.0179316	443.0316536	574.7186346	550.1758026	265.5718139
2.178011002	9.886613368	5.391865966	6.807158109	7.293997383	9.079378255
1598.660076	4394.599642	3751.840068	1895.307308	1653.653407	9348.354835
135.0366821	163.1291206	263.302788	316.0466265	134.4179518	248.5479797
9.801049509	38.55779214	91.66172143	20.985496	15.62999439	106.6826945
138.56506	98.55964867	91.23935859	79.4103616	59.72741857	85.95901363
28.31414303	43.50109882	104.242742	119.6114925	53.14198093	90.79378255
2248.79636	1088.516132	711.7263076	1594.8199	1509.857458	1710.327879
71.87436307	98.86613368	207.5868397	98.217567	135.4599514	112.3573059
68.60734656	144.3445552	292.0594065	181.8483666	114.6199589	97.60331624
574.9949045	775.1104881	1517.81027	1074.55853	721.0637413	1245.009743
3934.576875	2064.324871	1536.6818	2971.81074	2731.08102	3484.211405
175.3298857	290.666433	501.4435349	290.7628964	264.667905	415.3815551
22.86911552	45.47842149	606.5849212	24.31127896	26.04999065	93.06362711
243.9372322	134.4579418	143.7830924	112.8043344	170.8879387	152.0795858
339.7697163	560.570978	743.178859	665.1565924	482.4458269	515.2547159
53.36126955	115.6733764	185.1207315	169.2065016	112.5359596	78.30963745
23.95812102	45.47842149	90.7630771	41.81539981	28.13398991	98.73823852
59.89530256	47.45574417	151.8708914	98.217567	63.56197719	44.26196899
0	2.96598401	0.898644328	0	1.041999626	1.134922282
2744.402763	1245.713284	1447.716012	1921.563489	1867.97189	1917.133417
703.4975537	414.2491001	296.5526282	369.5314402	509.5378172	520.9293274
534.701701	880.8972511	1587.005883	1364.348975	901.3296766	1057.747567
166.6178417	183.8910086	372.937396	379.2559518	206.315926	118.0319173
2.580943037	0	0	0	1.187879574	1.441351298
67.51834106	0.988661337	0	0	0	1.134922282
212.3560727	1016.343854	1002.88707	889.79281	365.7418688	288.2702596

14.15707151	1.977322674	2.695932983	2.917353475	7.293997383	3.404766845
51.18325855	110.7300697	102.4454534	74.8787392	41.67998504	78.30963745
162.2618197	27.68251743	10.78373193	18.47657201	109.4099607	187.2621765
28.31414303	16.80724273	14.37830924	1.944902317	29.17598953	3.404766845
9.801049509	80.08156828	112.330541	33.06333939	29.17598953	236.0638346
0	0.988661337	4.511194525	0	0	0
18.51309352	9.886613368	7.189154622	11.6694139	8.335997009	7.944455973
248.2932542	339.1108385	634.4428954	476.5010676	400.1278564	190.6669433
6909.739904	5648.222217	5792.661337	2823.998164	6623.991623	5405.634828
977.9269399	998.5479502	1001.089781	1367.266329	1184.753575	695.7073588
128.5026491	213.5508488	259.7082107	413.2917423	134.4179518	200.8812439
895.1625218	675.255693	74.5874792	445.3826306	507.4538179	402.89741
0	1.898229767	1.851207315	0	1.073259615	0.987382385
130.6806601	138.4125872	122.2156286	219.7739618	132.3339525	478.9372029
1.089005501	1.977322674	1.797288655	0.972451158	1.041999626	0
356.1047988	654.493805	747.6720807	823.6661312	899.2456773	653.7152343
31.58115953	63.27432556	110.5332523	93.35531121	44.80598392	64.69057006
78.40839607	338.1221772	500.5448905	99.19001816	50.01598205	396.0878764
510.74358	1096.425423	1983.308031	1418.80624	1071.175616	1150.811194
89.29845108	39.54645347	0	0	0	0
633.8012016	918.4663819	1039.731487	723.5036619	1093.057608	1271.112956
10.89005501	11.86393604	12.58102059	24.31127896	17.71399364	19.29367879
1.339476766	3.934872121	4.897611586	0	10.14907636	1.407303629
17.42408802	18.7845654	23.36475252	35.0082417	12.50399551	7.944455973
993.1730169	136.4352645	159.060046	278.1210313	318.8518856	429.0006225
45.73823104	13.84125872	31.45255147	1.944902317	13.54599514	13.61906738
23.95812102	72.17227759	8.08779895	13.61431622	968.0176527	523.1991719
168.7958527	139.4012485	57.51323697	107.9420786	137.5439506	83.98424886
99.09950059	252.1086409	226.4583706	311.1843707	219.8619211	114.6271505
56.62828605	27.68251743	17.07424223	29.17353475	36.46998691	23.83336792
34.84817603	18.7845654	1.797288655	6.807158109	17.71399364	4.539689127
0	79.09290694	8.986443277	9.724511585	12.50399551	13.61906738
582.617943	371.7366626	368.4441744	450.2448864	365.7418688	410.841866
10.89005501	0	0	0	7.293997383	0
11.97906051	22.73921075	38.64170609	35.0082417	39.59598579	15.88891195
53.36126955	98.86613368	170.7424223	113.7767855	80.23397121	73.76994832
0	0.988661337	0	0.972451158	0	0

236.3141937	121.6053444	257.0122777	113.7767855	147.9639469	125.9763733
66.97383831	63.94661527	92.19192158	109.0701219	86.24630905	111.2791297
1.089005501	0.988661337	0.898644328	0	0	0
42.47121454	60.30834155	99.74952038	68.07158109	44.80598392	76.03979288
2.178011002	0	2.695932983	0.972451158	0	0
19797.031	12640.03519	8753.694396	9614.624604	16382.31812	8669.671311
302.7435293	724.6887599	1424.351259	183.7932689	877.3636852	836.4377217
0	0	1.797288655	0.972451158	0	2.269844564
124.1466271	79.09290694	16.1755979	59.31952067	143.7959484	44.26196899
324.5236393	551.6730259	21039.95965	636.9555088	517.8738142	83138.73176
1339.476766	595.1741248	95.25629874	139.0605157	591.8557876	204.2860107
0	0	0	0	0	0
6.534033006	24.71653342	18.87153088	16.53166969	7.293997383	26.10321248
30.49215403	52.39905085	41.33763908	177.958562	54.18398056	23.83336792
93.69803331	124.5713284	241.7622835	224.6362176	121.9139563	241.7724937
297.2985018	527.9451539	669.4900242	564.0216719	572.0577947	795.5805196
0	0	97.95223172	0.972451158	0	2.269844564
13.06806601	43.50109882	46.72950504	38.89804634	35.42798729	21.56352335
239.5812102	135.4466031	110.5332523	180.8759155	171.9299383	137.3255961
10.89005501	26.69385609	68.29696891	13.61431622	21.88199215	107.8176168
38.11519254	86.0135363	122.2156286	56.40216719	61.47797794	68.09533691
76.23038507	115.6733764	140.1885151	190.6004271	94.82196598	65.82549235
0	0	4.493221639	0	2.083999252	0
1.099895556	0	0	0	1.104519604	0
20.69110452	43.50109882	88.06714412	63.2093253	66.68797607	71.50010376
47.91624205	37.5691308	26.0606855	25.28373012	46.88998318	55.61119181
1491.937536	564.5256233	338.7889116	166.2891481	152.1319454	263.3019694
111.0785611	73.16093892	50.32408235	59.31952067	52.09998131	47.66673584
5.445027505	20.76188807	8.08779895	29.17353475	30.21798916	7.944455973
1.089005501	3.954645347	1.797288655	40.84294866	3.125998878	1.134922282
1.089005501	0	0	0	0	0
0	0.988661337	65.60103592	0	3.125998878	141.8652852
423.6231399	693.0515971	802.4893847	1000.652242	840.8936983	484.6118143
142.6597206	65.25164823	84.47256681	57.37461835	89.61196785	111.2223836
1095.539534	438.9656335	352.2685765	378.2835006	691.8877517	393.8180318
0	0	73.68883487	1.944902317	0	1.134922282
142.6597206	187.845654	253.4177004	394.8151703	136.501951	383.6037313

32.67016503	132.4806191	35.04712878	109.8869809	61.47797794	57.88103637
239.5812102	375.691308	442.1330092	205.1871944	387.6238609	562.9214518
9.692148959	40.72296046	12.80568167	33.37452376	17.03669389	24.95694098
965.9478794	930.3303179	574.2337254	690.4403225	809.6337095	495.9610372
39.20419804	50.42172818	127.6074945	93.35531121	62.51997757	76.03979288
3.267016503	8.897952031	1.797288655	15.55921854	10.41999626	20.42860107
17.42408802	36.58046946	34.14848445	22.36637664	22.92399177	45.39689127
177.5078967	228.3807688	380.1265506	495.9500908	200.0639282	221.309845
446.4922554	474.5574417	993.9006265	858.6743729	467.8578321	518.6594828
11.97906051	2.96598401	0	0.972451158	0	4.539689127
641.4242401	930.3303179	828.5500702	619.4513879	690.8457521	726.3502604
1.089005501	0	0	0.972451158	0	0
23.95812102	2.96598401	3.594577311	0	6.251997757	3.404766845
266.8063478	735.5640346	1336.284115	1134.850502	563.7217977	494.8261149
976.8379344	400.4078414	186.0193758	654.4596296	158.3839432	206.5558553
152.4607701	140.3899098	144.6817368	214.911706	150.0479462	108.9525391
8.712044008	7.909290694	16.1755979	15.55921854	2.083999252	10.21430054
759.0368342	964.9334647	1626.546233	2401.954361	1082.637612	988.5173075
1.089005501	0.988661337	0	0	0	0
565.8145882	3440.828164	2792.420425	3080.890587	936.7576639	1246.337602
265.7173423	164.1177819	61.10781429	181.8483666	146.9219473	79.44455973
703.4975537	625.8226262	696.449354	1095.952456	895.0776788	585.6198974
164.4398307	139.4012485	127.6074945	138.0880645	107.3259615	245.1432129
127.4136436	39.54645347	53.91865966	59.31952067	63.56197719	82.84932657
0	0	7.189154622	0	1.041999626	2.269844564
377.8849089	188.8343153	97.95223172	352.9997705	207.3579256	157.7541972
3.267016503	7.909290694	8.08779895	4.862255792	9.377996635	2.269844564
120.8796106	423.1470522	50.32408235	312.1568219	391.7918594	522.0642496
1.393927041	1.255599898	0	0	0	0
210.1780617	361.8500493	507.7340452	446.3550817	328.2298822	362.0402079
56.62828605	67.2289709	124.0129172	69.04403225	92.73796672	133.9208293
507.4765635	786.9744241	2854.094385	1317.67132	607.485782	837.572644
23.95812102	69.20629358	53.02001534	78.76854384	53.14198093	40.85720215
1.089005501	2.96598401	62.90510294	1.944902317	12.50399551	13.61906738
107.8115446	194.7662834	237.2421025	198.3800363	174.0139376	155.4843526
830.9111973	374.7026467	61.10781429	280.0659336	806.5077106	376.7941976
76.23038507	148.2992005	122.2156286	143.9227715	61.47797794	111.2223836

776.4609222	315.3829664	256.1136334	366.6140867	466.8158325	383.6037313
130.6806601	231.3467528	321.7146693	334.5231985	218.8199215	179.3177205
1.089005501	4.943306684	1.797288655	6.807158109	6.251997757	15.87756272
164.4398307	249.1426569	107.8373193	174.0687574	82.31797046	96.46839396
170.9738637	97.87747234	82.67527815	32.09088823	44.80598392	203.1510884
0	0.988661337	2.695932983	0	0	1.134922282
3.267016503	39.54645347	41.33763908	14.58676738	18.75599327	13.61906738
152.4607701	184.87967	411.5791021	305.3496638	78.14997196	317.7782389
0	3.954645347	127.6074945	0	0	140.7303629
0	0	2.695932983	0.972451158	0	3.404766845
57.74996172	159.2931146	234.5461695	76.82364152	224.0299196	339.4098576
7150.41012	3535.45294	19.77017521	3779.917653	3457.354759	124.841451
1.089005501	5.931968021	15.27695357	4.862255792	2.083999252	2.269844564
118.7015996	63.27432556	55.71594832	62.23687414	60.43597831	71.50010376
8289.509874	3471.189954	1268.885791	3963.710922	6548.96765	3559.116276
1565.87012	390.1751966	1503.144394	161.9228424	187.0493529	1834.170598
8.712044008	34.60314679	33.24984013	15.55921854	79.19197159	10.21430054
190.5759627	309.4509984	851.9148227	277.1485802	299.0538927	305.2940938
140.4817096	341.0881612	181.5261542	218.8015107	162.5519417	150.9446635
0	0	0	0	0	0
79.49740157	46.46708283	26.95932983	33.06333939	55.22598018	41.99212443
426.421884	650.5885927	1096.121419	1454.796658	677.7686568	394.7032712
93.65447309	119.6280218	190.5125975	195.4626828	136.501951	198.6113993
6.534033006	0.988661337	3.594577311	0.972451158	0	0
1254.534337	2665.430964	1728.093042	4684.29723	1699.50139	1679.684977
2.178011002	4.943306684	8.08779895	82.65834847	3.125998878	0
8.712044008	12.85259738	19.77017521	5.834706951	7.293997383	12.4841451
249.3822597	546.7297193	492.4570916	450.2448864	397.0018576	272.3813476
0	0	0	0	0	81.71440429
1.633508252	0.494330668	0	1.458676738	2.083999252	0.567461141
1.633508252	0.494330668	0	1.458676738	2.083999252	0.567461141
166.6178417	323.2922571	589.510679	163.3717946	269.8779032	399.4926432
1332.637812	563.536962	388.2143496	403.5672308	1942.287303	1215.501764
497.675514	92.93416566	13.47966492	49.59500908	91.6959671	10.21430054
956.1468299	226.4034461	270.4919426	274.2312267	696.0557502	398.3577209
1.089005501	9.886613368	0.898644328	1.944902317	1.041999626	2.269844564
1298.094557	557.604994	672.1859571	766.2915129	1002.40364	1352.82736

0	0	0	0	0	0
1.089005501	0	0	0	0	0
3.267016503	0	0	0	0	0
0	0	0	0	0	0
1.089005501	0	0	0	0	0
1.089005501	0	0	0	0	0
0	0	0	0	0	0
0	0.988661337	0	0	0	0
0	0	0	0	0	0
1.089005501	0	0	0	0	0
1.089005501	0	0	0	0	0
1.089005501	0	0	0	0	0
2.178011002	0	0	0	0	0
0	0	0	0	0	0
1.089005501	0	0	0	0	0
1.089005501	0	0	0	0	0
0	0	0	0	0	0
96.92148959	170.0497499	167.147845	342.3028078	178.1819361	163.4288086
9.801049509	18.7845654	33.24984013	14.58676738	12.50399551	15.88891195
87.12044008	155.2198299	234.5461695	170.1789527	123.9979555	137.3255961
874.4714173	541.7864126	389.1129939	394.8151703	550.1758026	738.8344055
7.623038507	19.77322674	60.20916996	13.61431622	5.209998131	12.4841451
1954.764874	4280.903588	5528.459904	2153.006865	3386.498785	2681.821352
484.607448	250.1313182	120.4183399	425.9336074	402.2118557	226.9844564
343.0367328	186.8569927	213.87735	310.2119195	239.659914	317.7782389
12.22953178	17.58828518	13.94695997	15.89957644	16.00511426	26.6593244
5.445027505	9.886613368	243.5326128	2.917353475	6.251997757	24.9682902
0.23958121	0	16.00485548	2.431127896	2.323659166	2.485479797
832.0002028	222.4488008	11.68237626	121.5563948	437.639843	81.71440429
0	0	0.898644328	0	0	0
2.178011002	2.96598401	0.898644328	0	2.083999252	0
0	2.96598401	3.594577311	0.972451158	3.125998878	0
28.31414303	38.55779214	59.31052563	30.14598591	37.51198654	40.85720215
0	2.96598401	4.493221639	13.61431622	2.083999252	1.134922282
22.86911552	110.7300697	44.03357206	42.78785097	39.59598579	20.42860107
461.7383324	223.4374621	134.7966492	230.4709246	284.4658979	180.4526428
177.5078967	112.7073924	19.77017521	46.67765561	138.5859503	79.44455973

7.623038507	5.931968021	23.36475252	24.31127896	7.293997383	17.02383423
360.4608208	230.3580915	119.5196956	194.4902317	263.6259054	280.3258036
384.4189419	675.255693	965.144008	641.8177646	636.6617716	669.6041463
241.7592212	496.3079911	488.8625143	355.917124	541.8398056	505.0404154
3.267016503	0	0	0	0	0
4115.59137	2319.785074	995.2665658	1621.834593	3436.275107	3242.654547
6.534033006	2.96598401	0.898644328	0	5.209998131	3.404766845
139.3927041	162.1404592	266.8973653	267.4240686	159.4259428	141.8652852
121.9686161	494.3306684	683.8683334	552.352258	278.2139002	244.0082906
23.95812102	57.34235754	98.85087605	0.972451158	18.75599327	2.269844564
15.24607701	152.2538459	21.56746387	25.28373012	118.7879574	59.01595865
251.5602707	365.8046946	3359.132497	443.4377283	438.6818426	1008.945909
112.1675666	398.4305187	194.1071748	213.9392549	160.4679424	150.9446635
33.75917053	20.76188807	25.16204118	18.47657201	29.17598953	26.10321248
199.2880067	33.61448545	108.7359637	17.50412085	42.72198467	49.9365804
0	0	0	0	0	0
25.04712652	101.8321177	115.0264739	59.31952067	25.00799103	2054.20933
0	1.977322674	1.797288655	0.972451158	1.041999626	0
109.9895556	108.752747	182.4247985	177.958562	142.7539488	181.5875651
3603.519203	16628.29502	42066.43963	3364.681008	9001.83477	12394.48624
218.8901057	435.0109882	323.511958	515.399114	359.489871	649.1755452
166.3564803	154.6464063	593.6534293	223.9457773	174.5140974	184.8674905
262.4503257	593.1968021	725.2059725	706.9719922	391.7918594	350.6909851
205.8220397	94.91148833	76.38476786	114.7492367	123.9979555	48.80165812
287.4974523	417.2150841	552.6662616	499.8398954	418.8838497	763.8026957
17514.47547	3609.602541	2912.506266	8255.137884	3778.290644	5255.825087
13.10073618	20.1192582	17.97288655	145.8773983	26.08125064	9.885173075
261.3613202	748.0409407	2027.790926	462.3324543	519.1554737	433.5403117
0	1.977322674	0	0	5.209998131	4.539689127
0	0	0	0	0	0
245.0262377	484.444055	467.2950504	668.0739459	433.4718445	306.4290161
331.0576723	179.9363633	154.5668244	141.9778691	298.0118931	241.738446
191.6649682	469.614135	2018.35516	564.9941231	800.2557129	1548.033992
26.13613202	37.5691308	159.060046	35.0082417	25.00799103	44.26196899
218.8901057	344.0541452	484.3692926	385.0906587	243.8279125	304.2045684
616.3771136	475.546103	1094.548791	893.6826146	1055.545621	758.1280843
63.16231906	97.87747234	124.0129172	110.8594321	80.23397121	60.15088094

92.56546759	163.1291206	131.2020718	92.38286005	116.7039581	113.4922282
19.60209902	13.84125872	39.54035042	8.752060426	36.46998691	11.34922282
0	4.943306684	14.37830924	8.752060426	0	1.134922282
377.8849089	495.3193297	3405.862002	402.5947796	499.1178209	923.8267374
23.95812102	68.21763224	34.14848445	36.95314402	11.46199589	40.85720215
53.36126955	58.33101887	149.1749584	74.8787392	59.39397869	48.80165812
1650.93234	4784.132209	5062.962142	6565.990222	4225.308484	1859.002698
0	0	8.08779895	4.862255792	0	0
0	0	0	28.2010836	1.041999626	0
103.4555226	212.5621874	237.2421025	217.8290595	252.1639095	56.74611409
21.78011002	27.68251743	56.61459265	21.39392549	9.377996635	111.2223836
5.445027505	14.82992005	9.885087605	11.6694139	15.62999439	5.674611409
3.267016503	9.886613368	4.493221639	55.42971603	35.42798729	41.99212443
0	1.977322674	5.391865966	0	0	0
1.089005501	3.954645347	2.695932983	2.917353475	0	1.134922282
40.29320354	12.85259738	8.08779895	36.95314402	29.17598953	24.9682902
271.1623698	474.5574417	620.9632305	703.0821876	621.0317772	199.7463216
5.445027505	11.86393604	10.78373193	6.807158109	10.41999626	21.56352335
160.0838087	263.9725769	493.3557359	211.0219014	174.0139376	376.7941976
5.445027505	16.80724273	2.695932983	45.70520445	3.125998878	4.539689127
1176.125941	772.1445041	436.7411433	674.881104	801.2977125	1097.469847
16.33508252	27.68251743	18.87153088	33.06333939	22.92399177	22.69844564
10.89005501	47.45574417	91.66172143	20.42147433	21.88199215	73.76994832
9.975290389	12.86248399	1.797288655	0.972451158	8.117177087	0
108.9005501	156.2084912	466.3964061	246.0301431	111.49396	394.9529541
8.712044008	28.67117877	11.68237626	146.8401249	66.68797607	32.91274617
125.2356326	183.8910086	841.1310908	154.6197342	340.7338777	154.3494303
0	0	0.898644328	2.917353475	0	4.539689127
6100.608817	9919.239192	11766.84883	10889.50807	13418.87119	15467.85578
1.089005501	4.943306684	5.391865966	3.889804634	1.041999626	1.134922282
1524.607701	508.1719271	275.8838086	591.2503043	987.8156456	288.2702596
3.267016503	0	0.898644328	0	1.041999626	1.134922282
22.86911552	0.988661337	0	0.972451158	0	1.134922282
1081.382463	138.4125872	63.80374727	99.19001816	244.8699121	76.03979288
141.5707151	15.81858139	0.898644328	0	1.041999626	0
208.0000507	241.2333662	471.7882721	298.5425056	203.1899271	245.1432129
182.9529242	506.1946044	635.3415397	297.5700545	278.2139002	516.3896382

0	0.988661337	0.898644328	0.972451158	1.041999626	0
12.75225442	25.08233811	46.44193886	52.14283112	6.272837749	5.527071512
57.71729155	81.07022962	288.4648292	114.7492367	245.9119118	249.682902
0	0	11.68237626	0	1.041999626	15.88891195
18.51309352	5.931968021	0.898644328	1.944902317	5.209998131	9.079378255
163.122134	187.6479217	242.0228903	223.8777057	197.2192692	210.664274
2.178011002	52.39905085	3.594577311	9.724511585	22.92399177	10.21430054
190.5759627	213.5508488	712.6249519	344.2477101	268.8359035	291.6750264
168.7958527	247.1653342	407.0858805	232.4158269	196.9379293	279.1908813
539.057723	386.5665827	186.0193758	206.1596456	388.6658605	263.3019694
0	0.988661337	46.72950504	0	0	0
238.4922047	309.4509984	481.6733597	279.0934825	213.6099234	424.4609334
1.089005501	48.4444055	31.45255147	0	47.9319828	3.404766845
0	0.988661337	0	0	0	2.269844564
0	0	0	2.051871944	1.052419622	2.292543009
0	0.988661337	0.898644328	1.944902317	0	1.134922282
50.09425305	134.4579418	195.0058191	111.8318832	100.0319641	68.09533691
353.9267878	590.2308181	904.934838	468.7214584	546.0078041	480.0721252
13.06806601	29.6598401	6.290510294	96.27266469	21.88199215	32.91274617
1.089005501	2.96598401	0	6.807158109	1.041999626	1847.653475
274.3313758	195.7549447	225.5597263	138.0880645	206.315926	329.1274617
1.089005501	0	0.898644328	2.917353475	1.041999626	0
1116.230639	2640.714431	2753.44622	2432.100347	2172.56922	2104.145911
325.6126448	260.0179316	186.9180202	201.2973898	271.9619024	190.6669433
384.4189419	376.6799693	1931.18666	374.393696	489.7398243	535.683317
0	0.988661337	0.898644328	0	1.041999626	4.539689127
1136.921743	742.4846639	1062.197595	654.4596296	856.5236927	1330.128914
197.1099957	277.8138356	437.6397876	281.0383848	204.2319267	220.1749227
676.2724161	272.870529	331.5997569	165.3166969	525.1678116	632.151711
89.29845108	187.845654	160.5158498	131.1933858	128.165954	121.4366842
216.7120947	224.4261235	235.4448139	253.8097524	257.3739077	270.1115031
1.089005501	0.988661337	8.08779895	2.917353475	1.041999626	21.56352335
160.0838087	218.4941554	186.9180202	888.8203588	177.1399364	240.6035237
125.2356326	183.8910086	247.1271901	331.605845	184.4339338	203.1510884
7.623038507	15.81858139	29.65526282	26.25618128	6.251997757	6.809533691
128.5026491	51.41038951	19.77017521	40.84294866	77.10797233	102.1430054
56.62828605	70.19495491	95.25629874	95.30021353	110.4519604	107.8176168

4.356022004	4.943306684	26.95932983	35.0082417	2.083999252	48.80165812
666.4713666	906.6024459	4029.521166	10797.12521	561.6377985	2013.352128
175.3298857	121.6053444	233.6475252	279.0934825	174.0139376	188.3970988
119.7906051	185.8683313	472.6869164	183.7932689	141.7119492	175.9129537
119.7906051	53.38771219	62.90510294	57.37461835	65.64597645	46.53181355
0	4.943306684	10.78373193	1.944902317	0	1.134922282
250.4712652	287.700449	3719.488872	1500.492137	128.165954	1670.605599
13308.73623	9789.724557	5118.678091	5899.861178	8994.540773	11663.59629
299.4765128	489.3873617	856.4080443	658.3494343	411.5898523	281.4607259
717.6546252	502.2399591	1.797288655	214.911706	317.809886	323.4528503
64.25132456	37.5691308	35.04712878	45.70520445	47.9319828	17.02383423
154.6387811	73.16093892	115.0264739	81.68589731	95.8639656	118.0319173
45.73823104	240.2447048	214.7759943	176.9861108	185.4759334	123.7065287
88.20944558	48.4444055	48.5267937	57.37461835	48.97398243	85.11917114
0	0	4.493221639	3.889804634	1.041999626	65.83684157
4758.954039	420.1810681	2691.439762	2650.901858	359.489871	211.0955444
26.13613202	130.5032965	886.9619515	53.48481372	86.48596897	85.11917114
6.534033006	29.6598401	13.47966492	25.28373012	9.377996635	4.539689127
456.2933049	563.536962	715.3118984	703.9962917	590.803368	626.4657503
83.85342358	100.8434564	136.5939378	219.7739618	107.3259615	136.1906738
340.8587218	747.4279706	674.8818901	585.4155974	563.7217977	379.0640421
1285.048271	2478.366353	6424.91154	5674.826256	1967.264034	1099.716993
109.9895556	106.7754244	187.8166645	289.7904452	111.49396	208.8256999
34.84817603	52.39905085	51.22272668	53.48481372	54.18398056	26.10321248
1071.439842	2778.860079	9612.160336	4787.163114	3023.716195	3477.367824
34.84817603	152.2538459	46.72950504	53.48481372	40.63798542	65.82549235
257.0052982	137.4239258	106.938675	181.8483666	252.1639095	160.0240417
889.7174943	569.46893	363.9509527	135.170711	748.1557316	1466.319588
28.31414303	122.5940058	44.03357206	48.62255792	42.72198467	91.92870483
63.16231906	161.1517979	1922.200217	269.3689709	87.52796859	339.3417623
1.089005501	39.54645347	8.08779895	16.53166969	7.293997383	14.75398966
251.5602707	316.3716278	447.5248752	983.1481212	420.967849	481.2070475
6.534033006	1.977322674	2.695932983	4.862255792	1.041999626	1.134922282
555.3928055	285.7231263	314.5255147	160.4544411	447.0178396	402.89741
175.3298857	184.87967	414.2750351	290.7628964	176.0979368	98.73823852
645.7802621	1035.12842	3086.843266	551.3798068	630.4097738	1532.14508
0	0.988661337	131.2020718	0.972451158	0	0

1.089005501	0	0.898644328	0	0	35.18259074
0	3.954645347	156.364113	0	0	3.404766845
814.5761148	607.0380608	336.0929786	368.5589891	722.1057409	654.8501566
0	0.988661337	0	0	0	0
14145.09245	1502.765232	3227.930425	548.4624534	2364.297152	5924.294311
14.15707151	1.977322674	13.47966492	0	14.58799477	2.269844564
1713.005653	1083.572825	351.3699321	846.0325079	1195.173571	1050.938033
0	0.988661337	5.391865966	1.944902317	0	0
175.3298857	171.0384113	255.2149891	269.3689709	190.6859316	372.2545084
92.56546759	44.48976016	17.07424223	42.78785097	79.19197159	15.88891195
5.445027505	7.909290694	10.78373193	26.25618128	21.88199215	21.56352335
57.71729155	213.5508488	55.71594832	70.01648341	60.43597831	57.88103637
18.51309352	14.82992005	4.493221639	5.834706951	9.377996635	9.079378255
4.356022004	26.69385609	168.9451336	176.0136597	11.46199589	6.809533691
5571.352143	2891.83441	465.4977618	2692.717258	2884.254965	2268.709641
0	28.72061183	7.988948074	26.440947	15.55705442	11.81454095
17.42408802	50.42172818	1141.278296	136.1431622	22.92399177	44.26196899
19.60209902	41.52377615	50.32408235	76.82364152	32.30198841	26.10321248
56.62828605	63.27432556	77.28341218	47.65010676	80.23397121	105.5477722
2.178011002	6.920629358	0	7.779609268	4.167998504	5.674611409
37.02618703	62.28566422	58.4118813	116.694139	58.35197906	103.2779276
0	0	0	2.917353475	0	13.61906738
470.4939367	741.9507868	1235.608991	878.7943874	689.9808924	454.8087552
8.712044008	101.8321177	19.77017521	35.0082417	17.71399364	122.5716064
176.4188912	166.0951046	14.37830924	46.67765561	42.72198467	14.75398966
2.722513753	0.612970029	0.449322164	0	0.520999813	0
2.722513753	0.612970029	0.449322164	0	0.520999813	0
177.5078967	71.18361625	17.97288655	1354.624464	222.98792	1050.938033
285.3194413	363.8273719	638.936117	456.0795933	392.833859	387.0084981
194.9319847	180.9250246	3.594577311	40.84294866	121.9139563	4.539689127
16.33508252	94.91148833	203.9922624	98.217567	47.9319828	54.47626953
209.0890562	162.1404592	178.8302212	112.8043344	109.4099607	146.4049744
162.2618197	90.95684299	78.18205651	92.38286005	88.56996822	186.1272542
321.2566228	648.5618369	693.753421	1217.50885	466.8158325	136.1906738
37902.83646	31658.91333	9807.804193	18194.56117	29418.77544	25313.30657
1319.874667	741.4960026	653.3144263	2042.147433	624.157776	528.8737833
173.1518747	374.7026467	487.0652256	522.2062721	385.5398617	270.1115031

385.5079474	588.2534954	640.7334057	423.9887051	422.0098486	562.9214518
79.49740157	67.2289709	40.43899475	81.68589731	22.92399177	51.07150268
10.89005501	5.931968021	0	0	0	0
0	20.76188807	8.08779895	1.944902317	11.46199589	23.83336792
43.56022004	174.9930566	151.8708914	36.95314402	164.6359409	556.1119181
0	0	1.797288655	21.39392549	0	1.134922282
0	0	1.932085305	1.059971763	0	2.57627358
3.267016503	13.84125872	8.986443277	20.42147433	13.54599514	1.134922282
2.178011002	1.977322674	7.189154622	505.6746024	8.335997009	3.404766845
25.71141988	46.01229862	44.77046041	28.63868662	38.1892863	50.57213688
590.2409816	207.6188807	138.3912265	798.3824011	407.4218538	136.1906738
8.712044008	11.86393604	45.83086071	26.25618128	5.209998131	3.404766845
81.67541258	38.55779214	0.898644328	0	0	0
275.5183918	78.10424561	171.6410666	75.85119036	180.2659353	205.420933
40.29320354	29.6598401	12.58102059	18.47657201	36.46998691	26.10321248
115.4345831	194.7662834	442.1330092	115.7216879	146.9219473	300.7544047
2732.314802	3256.650443	8101.278614	4052.203977	3562.596722	5201.348818
691.5184932	313.4056438	52.12137101	298.5425056	169.8459391	63.55564778
81.67541258	144.3445552	181.5261542	150.7299296	100.0319641	120.3017619
66253.01468	53568.36039	162629.2456	102895.6892	72511.27434	216385.2583
214.5340837	202.675574	263.302788	313.129273	254.2479088	332.5322286
89.29845108	57.34235754	58.4118813	32.09088823	61.47797794	65.82549235
2.178011002	0	0.898644328	0.972451158	0	1.134922282
0	1.977322674	0.898644328	0	12.50399551	4.539689127
1288.293508	2141.440456	2200.779959	2518.6485	2234.047198	1856.732853
17.42408802	2.96598401	3.594577311	0.972451158	3.125998878	9.079378255
0	0	9766.466554	0	1.041999626	0
9.801049509	0.988661337	3.594577311	0	6.251997757	2.269844564
271.1623698	299.5643851	491.5584473	381.2008541	324.0618837	333.6671509
674.0944051	389.5325667	312.728226	563.0492207	493.9078228	636.6914001
470.4503764	1328.760837	3470.564394	513.4542117	849.2296953	2494.559175
0	1.977322674	0	0.972451158	1.041999626	1.134922282
127.4136436	176.9703793	567.9432151	341.3303566	177.1399364	132.785907
3.267016503	12.85259738	79.08070084	13.61431622	5.209998131	23.83336792
1579.057976	3249.729814	5636.297224	2786.072569	2720.661024	2105.280833
132.4666291	375.3353899	1064.228532	285.832569	118.7879574	190.6669433
939.8117474	1955.572124	2972.715436	2059.651554	2063.15926	1786.367672

597.8640201	380.6346147	341.4848445	244.0852408	542.8818052	592.4294311
0	0	2.695932983	177.958562	0	0
222.1571222	247.1653342	238.1407468	347.1650636	205.2739263	426.730778
63.16231906	14.82992005	25.16204118	24.31127896	40.63798542	19.29367879
9.801049509	10.8752747	1.797288655	0.972451158	6.251997757	2.269844564
16.33508252	54.37637352	66.49968025	113.7767855	56.26797981	17.02383423
103.4555226	142.3672325	54.81730399	248.9474966	229.2399177	177.047876
165.5288362	151.2651845	234.5461695	269.3689709	235.4919155	272.3813476
336.5026998	368.7706786	981.3196059	586.3880485	401.1698561	306.4290161
6.534033006	0	8.08779895	1.944902317	2.083999252	2.269844564
0	0	0	0	0	0
0	11.86393604	9.885087605	16.53166969	10.41999626	7.433740946
0	0	0.898644328	0.972451158	1.041999626	0
0	0	5.391865966	0.972451158	0	0
49.00524755	19.77322674	7.189154622	29.17353475	36.46998691	12.4841451
15.24607701	50.42172818	41.33763908	35.98069286	35.42798729	54.47626953
8.712044008	13.84125872	5.391865966	18.47657201	12.50399551	13.61906738
1.089005501	3.954645347	3.594577311	6.807158109	8.335997009	2.269844564
19.60209902	83.04755229	57.51323697	4.862255792	84.40196972	152.0795858
82.76441808	228.3807688	85.37121113	179.9034643	161.509942	43.12704671
7.002305372	0.988661337	0	0.320908882	5.001598205	0
0	0.988661337	208.485484	0	0	0
69.69635207	180.9250246	81.77663382	193.5177805	146.9219473	125.9763733
165.5288362	236.2900595	461.9031845	330.6333939	238.6179144	270.1115031
0	0	0	0.972451158	1.041999626	1.134922282
35.5342495	153.8060442	419.4242671	328.1730924	250.6738501	126.5665329
282.0524248	315.3829664	165.3505563	259.6444593	236.5339151	155.4843526
76.23038507	84.03621363	52.12137101	144.8952226	138.5859503	62.4207255
11.97906051	102.820779	35.04712878	42.78785097	36.46998691	6.809533691
0	0.988661337	3.594577311	0	0	0
2627.770274	1534.402395	909.4280597	1488.822724	1942.287303	1425.462386
205.8220397	412.2717775	336.0929786	418.1539981	427.2198467	146.4049744
17.42408802	2.96598401	16.1755979	4.862255792	3.125998878	1.134922282
1264.335387	459.7275216	1295.845121	303.4436595	694.1593109	397.2227986
162.2618197	266.9385609	171.6410666	521.2435454	349.0698748	554.9769958
1.089005501	0	0.898644328	0	0	0
137.2146931	142.3672325	115.0264739	105.0247251	97.94796486	130.5160624

2021.19421	1251.645252	1516.012981	1595.792351	1530.697451	2056.479175
0	0	0.898644328	2.917353475	0	0
132.8586711	593.1968021	176.1342882	207.1320968	252.1639095	174.7780314
163.3508252	491.3646844	406.1872361	418.1539981	316.7678863	121.4366842
41.38220904	5.931968021	0	0	27.09199028	13.61906738
3.267016503	23.72787208	13.47966492	18.90445052	11.46199589	5.674611409
752.4374609	1004.420599	2048.80123	1147.492367	1071.175616	2246.011196
1.089005501	1.977322674	3.594577311	6.807158109	5.209998131	2.269844564
19.60209902	58.33101887	26.95932983	23.3388278	21.88199215	26.10321248
2204.550066	2026.745854	1197.362689	10.69696274	187.0701929	7.592630065
103.4555226	98.86613368	204.8909067	252.8373012	177.1399364	105.5477722
517.277613	622.8566422	1010.076224	600.9748159	561.6377985	811.4694315
449.7592719	644.4391192	1096.462904	879.0083266	600.9316044	594.8354664
0	0	0	0.972451158	0	0
173.1518747	72.17227759	81.77663382	87.52060426	149.0059465	123.7065287
78.40839607	157.1971526	177.0329326	116.694139	130.2499533	80.57948201
39.20419804	37.5691308	53.91865966	108.9145297	53.14198093	89.65886026
2.178011002	3.954645347	0.898644328	0.972451158	2.083999252	0
17.42408802	46.46708283	578.7269471	22.36637664	16.67199402	23.83336792
0	0.988661337	0	1.944902317	2.083999252	3.404766845
241.7592212	426.1130362	583.2201687	374.393696	316.7678863	341.6116068
357.1938043	152.2538459	198.6003964	204.2147433	217.7779219	349.5560628
132.8586711	154.2311685	216.573283	225.6086688	172.9719379	198.6113993
1.089005501	3.954645347	2.695932983	0.972451158	0	4.539689127
3161.350299	2210.547883	1104.433879	1158.121258	2452.86712	3270.811969
0	6.920629358	6.290510294	11.6694139	8.335997009	5.674611409
167.7068472	351.9634359	310.9309374	208.1045479	311.5578882	160.0240417
5.445027505	2.96598401	3.594577311	0	2.083999252	10.21430054
510.74358	271.8818676	217.4719273	227.5535711	334.48188	406.3021769
60.98430806	59.31968021	379.2279063	293.6802499	112.5359596	182.7224874
114.3455776	174.0043953	220.1678603	227.5535711	207.3579256	206.5558553
2.178011002	1.977322674	0	0	0	0
177.5078967	302.5303691	675.7805345	880.0682984	434.5138441	173.6431091
277.6964028	187.845654	88.96578845	126.4186506	225.0719192	70.36518147
0	0	3.738360403	2.197739618	2.125679237	4.006275655
0	1.977322674	594.902545	0	1.041999626	0
209.0890562	212.5621874	443.9302979	221.7188641	211.5259241	254.2225911

665.3823611	975.8087394	760.2531013	1278.773273	1090.973609	880.6996907
448.6702664	487.410039	1185.311868	463.8592026	533.5038086	480.0721252
1.089005501	14.82992005	3.594577311	1.944902317	1.041999626	0
0	1.977322674	0	18.47657201	0	0
14.15707151	38.55779214	21.56746387	18.47657201	39.59598579	13.61906738
267.8953533	484.444055	897.7456834	430.7958632	344.9018762	299.6194824
1304.62859	653.5051436	414.2660486	893.6826146	805.465711	69.23025919
77.31939057	23.72787208	5.391865966	3.889804634	39.59598579	1.134922282
3.267016503	26.69385609	10.78373193	53.48481372	10.41999626	9.079378255
118.7015996	238.2673822	288.4648292	186.7106224	166.7199402	104.4128499
1.938429792	1.977322674	3.15424159	11.86390413	2.448699121	8.863743021
388.197791	657.8552535	456.6551016	679.4905225	625.0955757	694.3568012
395.0585256	604.09185	910.8209584	301.4598591	542.1107255	358.3630597
0	0.988661337	0	36.95314402	0	6.809533691
44.64922554	98.86613368	50.32408235	85.57570194	43.7639843	7.944455973
17.42408802	283.7458037	9.885087605	62.23687414	8.335997009	676.41368
64.25132456	16.80724273	14.37830924	17.50412085	41.67998504	13.61906738
358.2828098	205.6415581	177.9315769	163.3717946	238.6179144	205.420933
31.58115953	27.68251743	33.24984013	83.63079963	48.97398243	95.33347167
0	0.988661337	44.03357206	0	1.041999626	10.21430054
53.36126955	108.752747	97.05358739	65.15422762	62.51997757	48.80165812
1.089005501	16.80724273	3274.65993	7.779609268	0	44.26196899
274.4293863	241.2333662	299.2485611	204.2147433	275.0879013	304.1591715
3.267016503	0	0	1.944902317	0	0
2.624503257	2.155281714	17.42471351	8.781233961	4.79319828	2.485479797
126.3246381	50.42172818	24.26339685	30.14598591	58.35197906	68.09533691
1114.052628	2438.038857	4195.770366	3207.143921	1948.539301	1184.858862
111.0785611	251.6538567	764.7912551	141.1999082	213.8912633	179.8284356
3263.553466	3160.898593	10420.13145	7094.945305	3507.558301	3212.034344
55.53928055	108.752747	98.85087605	114.7492367	115.6619585	86.25409342
0	0	0.898644328	0.972451158	0	0
0	13.84125872	0.898644328	0	3.125998878	2.269844564
6.534033006	9.886613368	77.28341218	15.55921854	90.65396747	77.17471516
426.8901564	220.4714781	264.2014324	568.8839277	133.3759521	238.3336792
0	0.988661337	51.74394039	0	0	11.34922282
114.9989809	172.145712	205.56489	118.6390413	138.1378904	215.6352335
4.443142444	0	0.898644328	0	0	6.196675659

0	1.977322674	6.290510294	6.807158109	2.083999252	7.944455973
483.5184425	789.9404081	5448.480559	1632.745495	517.8738142	1250.684355
2647.372373	2073.222823	1103.535234	1668.726188	2376.801147	2122.304667
0	0	3.199173807	3.452201613	0	0
0	0	0	286.8730917	0	0
58.80629706	114.6847151	107.8373193	220.746413	141.7119492	102.1430054
5.445027505	0.988661337	0.898644328	1.944902317	1.041999626	0
0	0	18.87153088	0	0	0
157.9057976	271.8818676	366.6468857	729.3383688	320.9358848	296.2147156
34.84817603	13.84125872	41.33763908	7.779609268	12.50399551	11.34922282
79.49740157	35.59180813	27.85797416	35.0082417	44.80598392	15.88891195
8.712044008	32.62582411	55.71594832	64.18177646	40.63798542	21.56352335
370.2618703	215.5281714	137.4925821	433.7132167	370.9518669	162.2938863
39.20419804	32.62582411	159.9586903	66.12667878	29.17598953	89.65886026
162.2618197	354.9294199	403.4913032	214.911706	251.1219099	312.1036275
6.534033006	10.8752747	176.1342882	12.64186506	5.209998131	1180.319173
90.38745659	120.6166831	176.1342882	124.4737483	96.90596523	120.3017619
384.4189419	315.3829664	48.5267937	172.123855	224.0299196	145.2700521
4.356022004	79.47848487	8.986443277	15.55921854	75.34699296	0
52.27226405	139.4012485	194.1071748	152.6748319	81.27597084	104.4128499
448.7138266	192.5417953	22.36725732	88.16242203	201.4393677	42.78657002
219.9791112	95.90014967	184.2220872	127.3911018	107.3259615	186.1272542
3324.733795	2788.02497	376.5319733	3605.848896	2878.002967	1223.44622
5880.629706	2303.580915	807.8812506	4985.757089	7086.639457	6213.699493
3.267016503	2.96598401	0	0	2.083999252	0
82.76441808	140.3899098	136.5939378	149.7574784	110.4519604	103.2779276
162.2618197	93.922827	115.9251183	69.04403225	102.1159634	52.20642496
1004.063072	462.6935056	239.9380355	295.6251522	334.48188	280.3258036
203.6440287	418.2037455	913.022637	1852.519457	492.8658232	372.2545084
219.9791112	417.2150841	511.3286225	595.140109	468.8998318	306.4290161
316.9006008	395.4645347	511.3286225	597.0850113	365.7418688	492.5562703
132.8586711	169.0610886	355.8631538	138.0880645	134.4179518	278.055959
6.534033006	48.4444055	28.75661849	20.42147433	18.75599327	14.75398966
8.712044008	6.920629358	0	0	0	0
0	0	0	0	0	0
0	4.943306684	0.898644328	0	2.083999252	0
480.251426	372.725324	40.43899475	71.96138573	357.4058718	35.18259074

2702.911654	1461.241456	1658.897429	1156.244427	1752.643371	3427.465291
13.06806601	25.70519476	40.43899475	10.69696274	17.71399364	35.18259074
1.089005501	2.96598401	5.391865966	13.61431622	0	0
2.178011002	0	0	0	0	0
279.8744138	112.7073924	139.2898708	102.1073716	169.8459391	99.8731608
11.97906051	6.920629358	31.45255147	19.44902317	15.62999439	20.42860107
53.36126955	14.82992005	12.58102059	1.944902317	11.46199589	13.61906738
279.3625812	349.2347306	563.3241833	395.7876215	271.0657827	517.0251947
210.1780617	142.3672325	185.1207315	130.3084552	129.2079536	129.3811401
10.89005501	2.96598401	2.695932983	0	3.125998878	0
215.6230892	246.1766729	508.6326895	462.8867514	270.9199028	317.7782389
739.4347352	392.4985507	699.145287	191.5728782	582.477791	116.896995
57.71729155	56.3536962	57.51323697	83.63079963	63.56197719	91.92870483
0	2.96598401	179.7288655	0.972451158	3.125998878	0
0	0	0.898644328	1.944902317	0	0
128.5026491	174.0043953	270.4919426	257.699557	162.5519417	324.5877726
5.445027505	0	0.898644328	0	0	0
9.801049509	3.954645347	0	3.889804634	3.125998878	1.134922282
148.1047481	223.4374621	234.5461695	197.4075852	374.0778658	270.1115031
2607.079169	3557.20349	6252.767232	5194.834088	2892.590962	3041.591715
0	0.988661337	1.797288655	0.972451158	1.041999626	0
0	1.977322674	0.898644328	0.972451158	0	0
272.2513753	431.0563429	963.3467193	542.6277464	361.5738703	407.4370992
344.1257383	440.9429562	500.5448905	515.399114	454.311837	401.7624878
30.49215403	11.86393604	720.7127508	0	108.3679611	232.6590678
76.23038507	126.5486511	202.1949737	106.9696274	105.2419622	90.79378255
186.2199407	82.05889096	130.3034275	236.3056315	211.5259241	231.5241455
3.267016503	0.988661337	0	0	0	0
0	0.988661337	0	0	1.041999626	0
43.56022004	27.68251743	25.16204118	18.47657201	30.21798916	32.91274617
65.34033006	109.7414084	72.79019055	156.5646365	152.1319454	68.09533691
245.0262377	109.7414084	44.03357206	150.7299296	188.6019323	140.7303629
392.0419804	243.2106889	137.4925821	271.3138732	244.8699121	240.6035237
10.89005501	13.84125872	21.56746387	2.917353475	10.41999626	3.404766845
22.86911552	30.64850144	486.1665813	24.31127896	30.21798916	41.99212443
124.1466271	81.07022962	124.0129172	99.19001816	97.94796486	130.5160624
17.42408802	201.6869127	118.6210513	21.39392549	25.00799103	3.404766845

1.089005501	1.977322674	1.797288655	5.834706951	1.041999626	1.134922282
60.98430806	62.28566422	209.3841284	169.2065016	132.3339525	171.3732646
325.6126448	728.6434052	653.3144263	1609.406667	404.2958549	1834.034407
116.5235886	75.1382616	61.10781429	82.65834847	97.94796486	74.9048706
753.5918067	359.8727266	34.14848445	427.8785097	1217.055563	396.0878764
20.69110452	69.20629358	23.36475252	19.44902317	51.05798168	54.47626953
268.9843588	429.0790202	592.206612	394.8151703	331.3558811	561.7865295
264.6283367	357.8954039	577.8283027	578.6084393	371.9938665	547.0325398
479.1624205	442.9202789	1063.09624	530.9583325	530.3778097	465.3181355
1041.089259	3069.773678	2110.016882	1860.279617	1855.801334	1639.962697
116.5235886	164.1177819	148.2763141	236.3056315	189.643932	138.4605184
776.4609222	749.4052933	228.2556592	854.7845683	202.1479275	325.7226949
2739.937841	12.85259738	0	0	4.167998504	5.674611409
15.24607701	22.73921075	36.84441744	25.28373012	3.125998878	15.88891195
1085.738485	723.7000985	823.1582042	778.9333779	816.9277069	1260.898655
60.98430806	2.96598401	3.594577311	13.61431622	33.34398804	1.134922282
38.11519254	23.72787208	8.986443277	12.64186506	13.54599514	31.77782389
119.7906051	138.4125872	94.35765441	224.6362176	165.6779406	219.0400004
2510.15768	1758.828518	880.6714412	1089.145297	2668.561042	3067.694928
14.15707151	8.897952031	2.695932983	5.834706951	4.167998504	5.674611409
13.06806601	2.96598401	860.0026216	1564.673914	1.041999626	0
63.16231906	26.69385609	10.78373193	13.61431622	30.21798916	30.64290161
254.8272872	334.1675318	435.8424989	496.922542	320.9358848	318.9131612
312.1307567	294.8781303	421.2754744	337.47945	360.1671708	487.8917397
65.34033006	121.6053444	47.62814937	62.23687414	52.09998131	66.96041463
1428.775217	1026.230468	519.4164214	509.564407	1236.853556	1138.327049
58.80629706	101.8321177	161.755979	416.2090958	113.5779592	85.11917114
7.448797627	1.295146351	0	1.303084552	2.594579069	0
2.178011002	8.897952031	20.66881954	3.889804634	3.125998878	60.15088094
20.69110452	13.84125872	0.898644328	6.807158109	8.335997009	10.21430054
16777.7088	9173.264554	5179.866783	5594.793525	7263.560574	15853.15054
50.88922706	110.7300697	289.3634735	127.3911018	66.68797607	78.30963745
0	3.954645347	5.391865966	42.78785097	0	9.079378255
172.0628692	162.1404592	285.7688962	215.8841572	213.6099234	196.3415548
1.089005501	0	50.32408235	0	0	0
353.9267878	363.8273719	298.3499168	260.6169105	340.7338777	247.4130574
1.089005501	7.909290694	29.65526282	0	2.083999252	2.269844564

4094.954715	2615.533226	969.88885	1825.037987	3231.376301	3621.99097
719.8326362	531.8997992	207.5868397	546.5175511	748.1557316	274.6511922
1119.029383	1878.634499	2300.835018	3556.681765	1995.491804	1356.084587
35.78472076	77.21445041	68.21609092	94.70701832	14.56715477	135.6686096
986.6389839	504.2172818	823.1582042	55.42971603	411.5898523	442.6196899
0	0	0	0	0	0
0	0	0	0	0	0
0	0	0	0	0	0
0	0	0	0	0	0
0	0	0	0	0	0
6.534033006	62.28566422	18.87153088	6.807158109	22.92399177	34.04766845
94.74347859	205.6415581	397.2007929	165.3166969	180.2659353	510.7150268
2.178011002	5.931968021	3.594577311	1.944902317	1.041999626	0
1.089005501	3.954645347	6.290510294	11.6694139	1.041999626	5.674611409
8390.787385	4007.044398	7934.13077	3467.760831	5746.627938	8702.584057
14.15707151	26.69385609	177.9315769	23.3388278	21.88199215	30.64290161
119.7906051	93.922827	354.0658651	182.8208178	177.1399364	293.944871
160.0838087	193.777622	605.6862769	426.9060586	176.0979368	281.4607259
2.178011002	9.886613368	0.898644328	4.862255792	3.125998878	2.269844564
345.2147438	580.3442047	796.1988744	578.6084393	447.0178396	379.0640421
4.356022004	0	0.898644328	0	1.041999626	1.134922282
0	1.08752747	0	5.902778532	0	0
3460.859482	1786.511036	1788.302212	2005.194289	2957.194939	707.0565816
50.09425305	52.39905085	110.5332523	48.62255792	48.97398243	57.88103637
0	0	3.594577311	1.944902317	1.041999626	0
616.3771136	714.8021465	726.1046168	581.5257928	863.8176901	981.7077738
341.9477273	422.1583908	849.2188897	739.0628804	399.0858568	262.1670471
224495.217	144100.3558	208040.6551	66654.71975	107948.0353	140076.6477
570.6388825	245.1880115	321.7146693	250.8923989	459.5218351	274.6511922
44.64922554	63.27432556	39.54035042	126.4186506	52.09998131	49.9365804
201.2482166	110.2653989	99.19236089	88.35691226	139.7008899	127.644709
208.0000507	155.2198299	20.66881954	75.85119036	142.7539488	79.44455973
37.02618703	63.27432556	94.35765441	87.52060426	64.60397682	63.55564778
141.7667361	253.9574376	323.1165545	159.3069488	149.5790463	384.4095261
93.65447309	172.0270726	239.0393912	98.217567	113.5779592	139.5954407
1252.356326	1146.847151	3656.58377	3165.328521	1288.953538	1878.296376
133.9476766	153.2425072	150.9722471	164.3442458	235.4919155	182.7224874

3.267016503	12.85259738	29.65526282	7.779609268	5.209998131	28.37305705
332.1466778	252.1086409	42.2362834	99.19001816	169.8459391	239.4686015
35.93718153	27.68251743	7.189154622	10.69696274	9.377996635	7.944455973
263.5393312	297.5870624	612.8754315	357.8620263	309.473889	333.6671509
2.178011002	0	0	0	0	0
1.807749132	0	0	0	0	0
2.178011002	0	0	0	0	0
2.178011002	0	0	0	0	0
2.178011002	0	0	0	0	0
2.178011002	0	0	0	0	0
2.178011002	0	0	0	0	0
0	0.988661337	70.09425756	104.052274	0	0
0	0.988661337	0.898644328	0.972451158	0	2.269844564
5.445027505	24.71653342	14.37830924	9.724511585	1.041999626	2.269844564
13.06806601	10.8752747	6.290510294	57.37461835	22.92399177	18.15875651
8.712044008	11.21141956	31.89288719	48.5155883	19.44371302	39.00727883
127.4136436	72.17227759	7.189154622	53.48481372	84.40196972	2.269844564
3.267016503	32.62582411	1.797288655	5.834706951	8.335997009	15.88891195
15.24607701	31.63716278	24.26339685	27.22863244	15.62999439	21.56352335
1.089005501	0.988661337	0	0	0	1.134922282
2351.162877	791.9177308	576.0310141	476.5010676	193.8119305	197.476477
87.12044008	55.36503486	12.58102059	6.807158109	23.9659914	178.1827982
2.178011002	9.886613368	9.885087605	8.752060426	3.125998878	48.80165812
203.6440287	281.768481	771.9354775	405.5121331	339.6918781	517.5245605
154.6387811	91.94550432	128.5061389	48.62255792	126.0819548	167.9684977
306.0105458	548.7070419	678.4764674	455.1071422	516.8318146	385.8735758
3.267016503	3.954645347	0	1.944902317	1.041999626	0
2.178011002	0.988661337	1.797288655	0	1.041999626	1.134922282
0	0	8.08779895	0.972451158	4.167998504	0
138.3036986	243.2106889	420.5655454	204.2147433	243.8279125	182.7224874
56.62828605	104.7981017	88.96578845	190.6004271	140.6699495	121.4366842
224.3351332	177.9590406	117.7224069	177.958562	197.979929	169.10342
248.2932542	136.4352645	5.391865966	47.65010676	249.0379106	69.23025919
137.2146931	92.93416566	108.7359637	98.217567	142.7539488	118.0319173
0	0	0	0	0	0
0	0	0	0	0	0
0	0	0	0	0	0

0	0	0	0	0	0
0	0	0	0	0	0
0	0	0	0	0	0
0	0	0	0	0	0
0	0	0	0	0	0
0	0	0	0	0	0
0	0	0	0	0	0
0	0	0	0	0	0
0	0	0	0	0	0
0	0	0	0	0	0
0	0	0	0	0	0
0	0	0	0	0	0
0	0	0	0	0	0
0	0	0	0	0	0
0	0	0	0	0	0
1745.675818	1429.604293	596.6998336	516.3715651	2064.201259	1256.358966
427.9791619	217.5054941	155.4654687	257.699557	289.6758961	178.1827982
123.0576216	61.29700288	14.37830924	27.22863244	44.80598392	36.31751302
105.6335336	290.666433	254.3163447	174.0687574	109.4099607	143.0002075
918.0316374	1125.096601	2003.976851	1094.980004	926.3376676	532.2785502
144.8377316	103.8094404	46.72950504	48.62255792	201.1059278	133.9208293
89.29845108	154.2311685	80.8779895	207.1320968	219.8619211	91.92870483
8396.232413	6427.287351	3815.643816	9029.209006	4655.65433	3240.203115
64.25132456	46.46708283	159.9586903	115.7216879	306.3478901	80.57948201
203.6440287	919.4550432	1956.348701	3094.339586	1477.55547	3079.044151
0	0	0	3.889804634	0	0
1.089005501	21.75054941	1.797288655	3.889804634	0	0
0	7.909290694	1.797288655	0.972451158	7.293997383	1.134922282
71961.48351	40060.55737	18732.24101	17713.19785	43148.16252	112358.4408
104.5445281	287.700449	337.8902672	390.9253657	156.2999439	190.6669433
124.1466271	240.2447048	159.060046	173.0963062	175.0559372	199.7463216
65.34033006	122.5940058	130.3034275	83.63079963	93.77996635	66.96041463
58.80629706	42.51243748	26.95932983	11.6694139	36.46998691	20.42860107
755.7698177	1313.930917	1446.817368	694.3301271	1020.117634	718.4058044
441.0472279	237.2787208	468.1936947	302.4323103	608.5277817	537.9531616
273.3403808	162.1404592	118.6210513	198.3800363	234.4499159	181.5875651
172.0628692	230.3580915	292.9580508	323.8262358	277.1719005	241.738446

55.53928055	56.3536962	141.9858038	258.6720081	44.80598392	83.98424886
849.4242908	614.9473515	347.7753548	409.4019377	851.3136945	780.8265299
345.2147438	1333.704143	497.8489576	641.8177646	691.8877517	569.7309855
2.178011002	6.920629358	178.4258313	0.972451158	0	6.809533691
164.4398307	87.00219764	57.51323697	68.07158109	75.02397308	144.1351298
6.534033006	41.52377615	68.29696891	0	15.62999439	22.69844564
72.96336857	73.16093892	234.5461695	140.0329668	78.14997196	150.9446635
0	0	0	0	0	0
0	0	0	0	0	0
0	0	0	0	0	0
0	0	0	0.408429487	0	0
0	0	0	0	0	0
0	0	0	0	0	0
0	0	0	0	0	0
0	0.988661337	66.49968025	1.944902317	0	0
5.445027505	0	0	0	0	0
0	1.977322674	3.594577311	1.944902317	8.335997009	4.539689127
23.95812102	12.85259738	26.0606855	4.862255792	20.83999252	13.61906738
53.36126955	461.7048443	217.4719273	282.9832871	128.165954	128.2462178
286.4084468	174.0043953	292.0594065	274.2312267	240.7019136	200.8812439
116.4364682	185.7892384	463.3050696	162.2632003	144.7129081	328.8891281
378.9739144	203.6642354	234.5461695	172.123855	315.7258867	314.3734721
0	1.977322674	1.797288655	7.779609268	0	6.809533691
0	0	9.300968792	0	0	0
28.31414303	35.59180813	59.31052563	46.67765561	63.56197719	73.76994832
0	0.988661337	0	1.944902317	4.605638347	0
23.95812102	11.86393604	4.493221639	0	5.209998131	0
15.24607701	450.8295696	3.594577311	0.972451158	5.209998131	0
776.4609222	1159.699748	1317.412584	1017.183912	1110.771601	1071.366634
0	0	0	0	0	0
1.089005501	0	18.87153088	0.972451158	1.041999626	0
615.2881081	632.7432556	1072.082683	1043.440093	474.1098299	1471.9942
3.267016503	0.988661337	0	0	2.083999252	0
0	0	0	0.972451158	0	0
559.7488275	1102.357391	902.238905	3196.446958	1884.977324	3097.202907
417.0891069	522.0131858	950.7656987	864.5090799	467.8578321	890.9139912
997.5290389	689.0969518	325.3092466	1230.150715	972.1856512	745.6439392

15.24607701	17.79590406	87.16849979	11.6694139	4.167998504	4.539689127
0	0	0.898644328	38.89804634	0	1.134922282
7993.300378	3680.786157	2809.162168	1183.47306	4770.274288	4797.316485
162.2618197	237.2787208	4338.654814	2305.681697	91.6959671	1074.771401
190.5759627	116.6620377	51.22272668	79.74099499	88.56996822	81.71440429
485.6964535	475.546103	716.2195292	678.7709086	683.5517547	514.1197937
1077.026441	241.2333662	487.96387	591.2503043	627.2837749	624.207255
5.445027505	20.76188807	8.986443277	32.02281665	22.92399177	25.75138657
8.712044008	2.96598401	0	1.944902317	1.041999626	0
364.8168428	192.7889607	270.4919426	335.4956497	184.4339338	286.000415
420.3561234	307.4736757	17.07424223	21.39392549	112.5359596	11.34922282
82.76441808	175.981718	1934.781238	171.1514039	57.30997944	225.8495341
0	0.988661337	1.797288655	0	2.083999252	1.134922282
32.67016503	61.29700288	119.5196956	43.76030213	43.7639843	37.4524353
421.4451289	2888.868426	978.6236729	1107.621869	722.1057409	953.3347167
50.09425305	27.68251743	27.85797416	22.36637664	26.04999065	24.9682902
4.356022004	30.64850144	11.68237626	24.31127896	14.58799477	7.944455973
98.01049509	40.53511481	28.75661849	44.73275329	48.97398243	29.50797933
4162.179025	2910.618976	2933.175086	2539.069975	3524.042736	5438.547575
789.5289882	393.4872121	350.4712878	517.3440163	257.3739077	150.9446635
5363.166962	2922.83883	3570.286955	4308.075326	3292.406219	5381.710667
104.5445281	79.09290694	68.29696891	85.57570194	147.9639469	99.8731608
13.06806601	27.68251743	8.986443277	7.779609268	15.62999439	15.88891195
72.96336857	107.3389613	425.058767	283.9557383	79.19197159	59.01595865
18.51309352	2.96598401	0	0	0	3.404766845
56.62828605	121.6053444	56.61459265	259.6444593	143.7959484	153.214508
0	0.988661337	0	0.972451158	1.041999626	3.404766845
132.8586711	265.9498996	366.6468857	634.0381553	215.6939226	170.2383423
105.6335336	195.7549447	332.4984013	331.605845	166.7199402	137.3255961
0	1.977322674	0	0	0	39.72227986
0	61.82099339	71.14567143	207.2098928	72.60653395	19.92923527
74.05237407	131.4919578	213.87735	182.8208178	202.1479275	133.9208293
69.69635207	37.5691308	37.74306176	45.70520445	63.56197719	26.10321248
43.56022004	45.47842149	35.04712878	23.3388278	29.17598953	43.12704671
1109.696606	1655.019078	2513.508185	1880.72054	1640.107412	1386.875028
138.3036986	84.03621363	94.35765441	85.57570194	109.4099607	162.2938863
3983.582123	1180.461636	2383.204757	1776.668267	2050.655264	2008.812439

96.92148959	172.0270726	319.0187363	190.6004271	132.3339525	144.1351298
3.267016503	42.51243748	13.47966492	47.65010676	58.35197906	23.83336792
161.1728142	238.2673822	168.0464893	254.7822035	185.4759334	86.25409342
160.0838087	267.9272223	316.3228034	233.388278	333.4398804	287.1353373
5520.168885	1554.175621	4156.230016	830.4732893	1430.665487	625.3421773
1110.785611	987.6726755	932.7928122	877.1509449	971.1436515	547.0325398
7.623038507	5.931968021	0	12.64186506	0	0
59.89530256	388.5439054	186.0193758	211.0219014	107.3259615	147.5398966
393.1309859	393.4872121	976.8263842	524.1511744	392.833859	354.0957519
0	0	0	0	1.041999626	0
0	1.977322674	0	0	1.041999626	0
238.4922047	171.0384113	204.8909067	379.2559518	129.2079536	121.4366842
57.71729155	80.08156828	489.7611586	300.487408	77.10797233	77.17471516
2000.503105	907.5911072	341.4848445	2329.020525	1400.447498	388.1434204
0	0	17.07424223	0	0	112.3573059
2.178011002	3.954645347	8.986443277	0	58.35197906	2.269844564
0	4.913646844	3.118295817	1.662891481	0	2.269844564
54.45027505	100.8434564	48.5267937	55.42971603	62.51997757	44.26196899
64.25132456	111.7187311	137.4925821	127.3911018	96.90596523	90.79378255
507.4765635	285.7231263	221.9651489	259.6444593	378.2458643	532.2785502
295.1204908	472.580119	416.9709681	434.6856678	395.9598579	278.055959
886.4504778	552.6616873	86.26985546	257.699557	549.133803	794.4455973
1379.76997	1021.287161	1460.297033	891.7377123	1292.079536	1129.24767
0	0.988661337	82.67527815	0.972451158	1.041999626	0
125.2356326	129.5146351	157.2627574	151.7023807	122.9559559	115.7620727
35.93718153	263.9725769	116.8237626	155.5921854	62.51997757	51.07150268
1571.434938	4467.760581	4076.250671	3391.909641	4588.966353	1250.684355
17.42408802	64.26298689	28.75661849	24.31127896	40.63798542	36.31751302
14.15707151	24.71653342	97.95223172	21.39392549	11.46199589	4.539689127
5.445027505	0	0	0	2.083999252	2.269844564
7746.096129	5914.172117	4708.896277	3919.95062	4092.974531	6630.21597
1432.042234	340.0994999	404.3899475	1.944902317	1303.541532	10.21430054
9.801049509	5.931968021	19.77017521	6.807158109	8.335997009	11.34922282
3.267016503	10.8752747	239.0393912	4.862255792	5.209998131	9.079378255
168.7958527	153.2425072	203.9922624	172.123855	111.49396	274.6511922
0	0	0	0	0	17.02383423
0	2.649612383	0	0	0	1.747780314

212520.5125	119791.1509	1972.524299	129219.3099	126494.5866	72417.12096
21659.23041	15065.22145	5611.135182	16629.88726	27942.26197	17147.54076
319.0786118	410.2944548	954.360276	437.6030213	340.7338777	455.103835
15.24607701	49.43306684	26.0606855	21.39392549	21.88199215	26.10321248
77.31939057	197.7322674	239.0393912	217.8290595	127.1239544	79.44455973
603.3090476	386.5665827	350.4712878	465.8041049	420.967849	549.3023844
1145.633787	739.5186799	785.4151424	811.9967173	885.6996822	1022.564976
22.86911552	5.931968021	0	0.972451158	17.71399364	1.134922282
49.00524755	64.26298689	149.1749584	109.8869809	38.55398617	63.55564778
29.40314853	41.52377615	47.62814937	59.31952067	27.09199028	19.29367879
90.38745659	89.96818165	145.5803811	95.30021353	105.2419622	69.23025919
0	0	0.898644328	509.1948756	0	3.404766845
321.2566228	183.8910086	232.7488809	98.217567	189.643932	385.8735758
1.089005501	6.920629358	9.885087605	9.724511585	2.083999252	2.269844564
23.88189064	7.909290694	0	0	1.041999626	6.843581359
2617.969224	2231.408637	2673.466875	2157.869121	2633.133055	2841.845394
3.267016503	16.80724273	9.885087605	10.69696274	7.293997383	3.404766845
186.2199407	231.3467528	310.9309374	267.4240686	208.3999252	341.6116068
3.267016503	1.977322674	0	0	0	0
326.7343205	470.2963113	1098.314111	482.4816423	446.5072598	377.6794369
22.86911552	96.88881101	122.2156286	591.2503043	85.44396934	29.50797933
38.11519254	47.45574417	78.18205651	30.14598591	61.47797794	48.80165812
633.7794215	403.077227	788.8479638	411.34684	377.2872246	575.4055969
408.3770629	548.7070419	863.5971989	585.4155974	579.3517921	539.0880839
57.71729155	97.87747234	141.0871595	70.01648341	60.43597831	114.6271505
0	0.988661337	0	0.972451158	3.125998878	0
88.20944558	19.77322674	6.290510294	40.84294866	27.09199028	44.26196899
151.3717646	162.1404592	208.485484	141.9778691	156.2999439	174.7780314
4.356022004	0	0	0	0	0
0	0	0	5.834706951	2.083999252	2.269844564
52.27226405	42.51243748	16.1755979	17.50412085	51.05798168	19.29367879
2.178011002	0.988661337	0	0	0	0
1.089005501	0	0	0.972451158	1.041999626	3.404766845
422.5341344	703.9268718	716.2195292	1056.081958	413.6738516	488.0165812
29.40314853	75.1382616	69.19561324	41.81539981	53.14198093	99.8731608
62.07331356	105.786763	267.7960097	106.9696274	67.7299757	131.6509847
537.9687175	685.1423064	817.7663382	1229.178264	508.4958175	439.2149231

324.1207073	201.4595206	208.3417009	120.5839436	226.0201389	438.8744464
44.64922554	2.96598401	1.797288655	0.972451158	12.50399551	30.64290161
109.9895556	150.2765232	149.1749584	596.1125601	217.7779219	26.10321248
2.178011002	0.988661337	0	0	0	0
192.7539737	212.5621874	429.5519887	256.7271058	200.0639282	224.7146118
32.67016503	22.73921075	2.695932983	8.752060426	9.377996635	5.674611409
1560.544883	611.9813675	591.3079676	877.1509449	873.1956867	691.1676696
315.8115953	1561.096251	1060.400307	1168.886292	261.5419062	803.5249755
5.445027505	35.59180813	331.5997569	11.6694139	12.50399551	10.21430054
214.5340837	318.3489505	503.2408235	446.3550817	283.4238983	234.9289123
13.06806601	33.61448545	55.71594832	279.0934825	14.58799477	14.75398966
1278.492458	773.1331654	283.0729632	1234.04052	1130.569594	404.0323323
82.76441808	147.3105392	259.7082107	210.0494502	105.2419622	121.4366842
245.0262377	347.0201292	478.0787824	362.7242821	289.6758961	625.3421773
0	0	1.797288655	0.972451158	0	0
29.40314853	87.99085898	185.1207315	93.35531121	60.43597831	73.76994832
760.1258397	157.1971526	12.58102059	70.01648341	395.9598579	127.1112956
1154.345831	704.9155331	518.5177771	925.7735029	751.2817304	665.0644572
0	0	0	0	0	3.404766845
2.178011002	4.943306684	5.391865966	7.779609268	1.041999626	3.404766845
0	0.494330668	0	0	1.041999626	0
0	0	0	1.944902317	0	1.134922282
512.921591	281.768481	211.181417	462.8867514	412.6318519	526.6039388
1.089005501	6.920629358	4.493221639	1.944902317	1.041999626	1.134922282
66.42933556	59.31968021	25.16204118	36.95314402	67.7299757	121.4366842
32.67016503	70.19495491	62.90510294	124.4737483	53.14198093	56.74611409
381.1519254	152.2538459	256.1136334	140.0329668	423.0518482	444.8895345
796.0630212	1015.355193	1726.295754	1285.580431	994.0676433	626.4770996
13.06806601	26.69385609	89.86443277	20.42147433	29.17598953	41.99212443
999.7070499	593.1968021	112.330541	198.3800363	779.4157203	329.1274617
0	0	3.594577311	4.862255792	0	1.134922282
0	1.977322674	0	0.972451158	0	0
176.4188912	156.2084912	328.0051796	215.8841572	209.4419249	309.8337829
38.11519254	22.73921075	15.27695357	9.724511585	21.88199215	45.39689127
3162.471975	1388.080517	541.8825296	1320.588673	3925.212592	1710.327879
157.9057976	69.20629358	39.54035042	81.68589731	103.157963	60.15088094
46.60943544	11.86393604	6.290510294	19.48792122	15.47369445	18.79431299

0	1.977322674	0	0	0	1.134922282
3.267016503	13.84125872	0	4.862255792	3.125998878	2.269844564
0	1.977322674	1.797288655	0.972451158	2.083999252	0
200.3770122	258.0406089	277.6810973	369.5314402	256.331908	121.4366842
0	1.977322674	6.290510294	2.917353475	0	1.134922282
0	0.988661337	2.695932983	0	1.041999626	0
222.1571222	124.5713284	43.13492773	98.217567	87.52796859	35.18259074
1.089005501	0.988661337	3.594577311	5.834706951	1.041999626	5.674611409
7.623038507	77.11558427	153.66818	16.53166969	1.041999626	14.75398966
462.8273379	660.425773	1126.001343	1011.349205	850.2716949	1049.803111
19.60209902	58.33101887	73.68883487	81.68589731	47.9319828	46.53181355
937.6337364	604.0720768	523.0109987	586.3880485	565.805797	769.4773071
241.7592212	290.666433	556.2608389	556.2420626	329.2718819	271.2464254
131.7696656	171.0384113	183.3234429	167.2615993	129.2079536	308.6988607
107.8115446	133.4692805	168.9451336	156.5646365	203.1899271	170.2383423
3.267016503	38.55779214	6.290510294	6.807158109	15.62999439	12.4841451
160.0838087	281.768481	567.0445708	335.4956497	222.98792	401.7624878
0	1.977322674	2.695932983	0.972451158	3.125998878	2.269844564
633.8012016	1152.779119	2066.881954	1383.797998	841.9356979	657.1200012
729.6336857	959.0014967	889.6578845	917.0214424	993.0256437	1709.192956
1157.612848	2236.351944	3532.570852	2387.367594	1813.079349	1712.597723
166.6178417	233.3240755	293.8566952	176.0136597	215.6939226	154.3494303
216.7120947	394.4758734	457.4099628	498.8674443	411.5898523	492.5562703
0	0	0.898644328	0	1.041999626	1.134922282
46.82723654	19.77322674	97.95223172	35.98069286	61.47797794	45.39689127
1617.173169	1577.903494	976.8263842	1679.423151	1789.113358	567.4611409
19.60209902	30.64850144	31.45255147	26.25618128	23.9659914	38.58735758
33.75917053	16.80724273	10.78373193	2.917353475	5.209998131	3.404766845
0	0	2.695932983	1.944902317	0	0
278.7854083	323.2922571	766.5436116	843.1151544	290.7178957	409.7069437
1156.523842	697.0062425	1203.284755	1309.89171	505.3698187	446.0244568
0	0.988661337	0	3.889804634	0	1.134922282
0	0	0	0	0	0
4.356022004	0.988661337	6.290510294	2.917353475	1.041999626	4.539689127
3973.781073	1665.894353	2087.550773	3080.72527	1828.709344	3858.735758
11.97906051	5.931968021	1.797288655	0.972451158	1.041999626	0
125.2356326	106.7754244	256.1136334	110.8594321	121.9139563	156.6192749

0	2.96598401	1.240129172	3.00487408	0	0
79.08357948	93.36917665	0	0	221.8521404	0.011349223
0	0.988661337	3.594577311	0	2.083999252	2.269844564
114.3455776	132.4806191	150.0736027	114.7492367	137.5439506	161.158964
6254.681315	8353.239181	19463.09047	6671.306682	6277.870607	12180.32641
5.445027505	1.038094404	0.898644328	0.972451158	0	3.495560628
119.7906051	280.7798197	266.8973653	446.3550817	257.3739077	148.6748189
2.178011002	0.988661337	0	0.972451158	0	0
606.5760641	884.8518964	1181.717291	1159.161781	956.5556568	600.3738871
59.89530256	36.58046946	0.898644328	5.834706951	96.90596523	1.134922282
0	4.943306684	1.797288655	0.972451158	0	0
83.85342358	122.5940058	201.2963294	85.57570194	129.2079536	124.841451
26.13613202	60.30834155	55.71594832	57.37461835	38.55398617	29.50797933
29.40314853	125.5599898	11.68237626	48.62255792	52.09998131	68.09533691
2.178011002	14.82992005	6.290510294	4.862255792	7.293997383	3.404766845
397.4870079	170.0497499	202.1949737	138.0880645	281.3398991	314.3734721
1.089005501	0	539.1865966	0	3.125998878	0
0	1.977322674	1.797288655	0	0	0
131.7696656	95.90014967	66.49968025	70.01648341	68.77197532	48.80165812
176.4188912	50.42172818	3.594577311	18.47657201	150.0479462	6.809533691
553.2147945	163.1291206	16.1755979	91.41040889	362.6158699	138.4605184
137.2146931	114.6847151	363.9509527	233.388278	157.3419435	149.8097412
3.267016503	0.988661337	0	0	1.041999626	1.134922282
962.6808629	588.2534954	548.1730399	671.9637505	525.1678116	774.0169962
0	0	0	0.972451158	0	2.281193786
0	0.988661337	0	0.972451158	2.083999252	1.134922282
128.5026491	141.3785712	270.4919426	146.8401249	127.1239544	192.9367879
0	0	200.3976851	0	0	0
1124.942683	1570.982864	2806.466236	1475.208407	1480.681469	1780.69306
107.8115446	433.0336655	253.4177004	266.4516174	202.1479275	240.6035237
447.5812609	677.2330157	1358.750224	571.8012812	952.3876583	332.5322286
0	0.988661337	0.898644328	0.972451158	0	0
1576.879965	1040.071726	491.5584473	1396.439864	1163.913582	888.6441467
792.7960047	343.0654839	651.5171376	620.4238391	517.8738142	1031.644354
1.089005501	1.977322674	38.64170609	10.69696274	4.167998504	2.269844564
242.107703	283.2910195	275.2817169	345.3952025	270.6594029	194.5483776
2.178011002	3.954645347	1.797288655	3.889804634	5.209998131	1.134922282

402.9320354	891.7725258	1025.353178	1251.544641	962.8076545	652.5803121
21.78011002	32.62582411	32.3511958	39.8704975	42.72198467	24.9682902
185.1309352	472.580119	419.666901	231.4433757	464.7318332	501.6356486
8796.986437	4089.103289	2984.397812	6223.687414	4121.108521	5605.38115
188.3979517	300.5530464	370.241463	277.1485802	486.6138254	317.7782389
7.808169442	94.37761121	9.633467193	21.76345693	21.06923244	153.5322863
478.0734149	337.1335159	431.3492773	386.0631099	336.5658792	303.0242492
9.801049509	1.977322674	0	1.944902317	1.041999626	1.134922282
102.3665171	50.42172818	63.80374727	88.49305542	63.56197719	99.8731608
2050.597358	1471.128069	1720.903888	653.4871785	1272.281543	3536.41783
332.1466778	441.9316176	549.9703286	363.6967333	408.4638534	255.3575134
1.089005501	0.988661337	0.898644328	0	1.041999626	289.4051819
103.4555226	172.0270726	265.1000767	126.4186506	81.27597084	72.63502604
2.178011002	1.977322674	0	0	2.083999252	0
53.36126955	393.4872121	189.6139532	216.8566083	48.97398243	32.91274617
498.7645195	732.5980506	158.1614017	415.2366447	836.7256998	701.3819702
142.6597206	74.14960026	6.290510294	34.03579055	25.00799103	27.23813476
47.91624205	6.920629358	1.797288655	11.6694139	8.335997009	12.4841451
7688.378837	2376.741854	140.1885151	1549.114695	4155.494509	7103.478562
29.40314853	44.48976016	69.19561324	45.70520445	22.92399177	52.20642496
1353.633838	595.1741248	266.8973653	586.3880485	952.3876583	1348.287671
1.089005501	4.943306684	0.898644328	16.53166969	6.251997757	172.5081868
567.371866	300.5530464	354.0658651	411.34684	440.7658418	685.4930582
250.4712652	406.3398094	750.3680137	505.6746024	416.7998504	400.6275655
196.0209902	227.3921075	372.937396	361.7518309	244.8699121	355.2306742
124.1466271	126.5486511	194.1071748	161.4268923	112.5359596	191.8018656
0	3.954645347	2.7408652	2.236637664	5.647637974	2.269844564
383.3299364	506.1946044	1057.668428	1171.793921	439.7238422	712.7084945
0	0	1.797288655	0	9.377996635	0
375.7068979	509.1605885	759.3544569	724.4761131	593.9397869	690.0327474
175.3298857	119.6280218	49.42543803	105.9971763	69.81397495	238.3336792
316.9006008	488.3987004	880.6714412	565.9665742	554.3438011	436.9450785
3.267016503	7.909290694	7.189154622	10.69696274	2.083999252	10.21430054
732.9007022	812.6796189	3577.503069	909.2418332	809.6337095	2374.257414
0	0	0	478.44597	1.041999626	0
142.6597206	150.2765232	176.1342882	154.6197342	150.0479462	141.8652852
427.9791619	363.8273719	256.1136334	424.9611562	353.2378733	627.6120219

290.7644688	544.7523966	708.1317303	1061.916665	524.1258119	489.1515035
60.98430806	97.87747234	107.8373193	114.7492367	77.10797233	78.30963745
21.78011002	10.8752747	7.189154622	2.917353475	31.25998878	10.21430054
4.356022004	10.8752747	10.78373193	6.807158109	9.377996635	44.26196899
0	0	1.204183399	1.944902317	1.041999626	0
286.4084468	187.845654	133.8980048	178.9310132	163.5939413	259.8972025
1277.403453	993.6046435	1134.987786	862.5641776	991.9836441	1677.415133
0	0.988661337	5.391865966	1.944902317	0	0
7.623038507	20.76188807	8.08779895	8.752060426	6.251997757	69.23025919
107.8115446	84.02632702	34.14848445	81.68589731	114.5470189	47.66673584
41.38220904	21.75054941	17.07424223	18.47657201	17.71399364	32.91274617
2.178011002	0.988661337	7.189154622	3.889804634	1.041999626	2.269844564
2.178011002	0.988661337	0	0	0	0
65.34033006	180.9250246	82.67527815	106.9696274	110.4519604	121.4366842
1.089005501	14.76071376	9.1571857	70.66802569	13.78565505	21.74511092
154.6387811	208.6075421	550.8689729	601.9472671	200.0639282	327.9925394
57.04210814	139.0255572	155.4474958	145.1383354	75.72211283	149.0152956
434.5131949	391.5098894	789.0097197	840.1978009	401.1698561	477.8022806
68.60734656	107.7640857	173.4383553	70.98893457	67.7299757	86.25409342
3.267016503	70.19495491	31.45255147	31.11843707	29.17598953	127.1112956
377.8849089	712.8248238	1221.257641	734.2006246	450.1438385	501.6356486
124.1466271	87.00219764	239.9380355	87.52060426	88.56996822	80.57948201
298.8339995	425.7571181	764.1981499	535.4705059	395.5117981	430.0561003
236.3141937	69.20629358	80.8779895	67.09912993	116.7039581	166.8335754
18.51309352	23.72787208	8.986443277	23.3388278	16.67199402	9.079378255
119.7906051	215.5281714	190.5125975	215.8841572	210.4839245	98.73823852
151.3717646	88.97952031	109.634608	307.2945661	194.8539301	323.4528503
332.1466778	799.8270215	932.7928122	361.7518309	299.0538927	514.1197937
286.4084468	592.2081408	465.4977618	546.5175511	944.0516613	733.1597941
461.7383324	924.3983499	1393.797352	1282.663078	950.303659	1099.739691
345.4869952	202.5470481	144.8524792	201.6085742	163.0625215	300.5614679
1.089005501	0.988661337	0	0	0	0
1911.204654	941.2055926	787.2124311	613.616681	1101.393605	2814.607259
1807.749132	1028.20779	542.7811739	1935.177805	880.4896841	66.96041463
13288.04512	20043.13128	20320.14554	16957.6033	16403.15811	91579.14877
157.9057976	238.2673822	48.5267937	103.0798228	98.98996448	70.36518147
98.01049509	368.7706786	186.0193758	188.6555247	183.3919342	244.0082906

0	0	0	0	0	0
37.45089918	74.46597189	68.20710447	86.60650017	45.7021036	35.37552752
51.57530053	71.69772015	153.5873021	112.3083843	53.82970069	83.31464471
2.178011002	16.80724273	10.78373193	12.64186506	11.46199589	5.674611409
0	0	0.898644328	97.24511585	2.083999252	0
241.7592212	390.521228	473.5855607	377.3110495	262.5839058	520.9293274
13.06806601	0.988661337	1.797288655	14.58676738	1.354599514	0
29.40314853	56.3536962	33.24984013	60.29197182	33.34398804	41.99212443
13.06806601	37.5691308	35.94577311	11.6694139	20.83999252	44.26196899
2.178011002	0	0	0	1.041999626	0
757.9478287	897.7044938	961.5494307	933.5531121	826.3057035	1014.62052
53.36126955	23.72787208	15.27695357	18.47657201	27.09199028	26.10321248
290.7644688	365.8046946	373.8360403	546.5175511	416.7998504	377.9291198
0	0	0	0.972451158	1.041999626	0
63.151429	287.8685214	44.66262309	236.1694883	38.94994602	131.004079
252.6492762	264.9612383	469.9909834	369.5314402	289.6758961	317.7782389
0	0.988661337	0	0	0	0
264.6283367	149.2878619	177.0329326	74.8787392	119.829957	76.03979288
0	0	1.797288655	0	1.041999626	1.134922282
1203.351079	251.1199796	869.8877092	149.7574784	111.49396	2.269844564
2180.189013	1544.289008	2884.648292	1177.638353	1330.633523	1914.613889
91.47646209	138.4125872	366.6468857	433.7132167	127.1239544	80.57948201
69.69635207	69.20629358	86.26985546	102.1073716	107.3259615	80.57948201
49.00524755	68.21763224	58.4118813	68.07158109	67.7299757	30.64290161
1.089005501	3.954645347	4.493221639	3.889804634	7.293997383	2.269844564
16.33508252	33.61448545	36.84441744	80.78151773	53.14198093	303.3760752
0	0	1.797288655	0.972451158	3.125998878	1.134922282
1.089005501	0	0	0	0	0
717.6546252	1060.833614	1331.790894	1258.351799	884.6576826	1280.192334
1098.806551	974.8200781	397.2007929	694.3301271	930.5056661	1544.629226
347.3927548	337.1335159	694.6520653	697.2474806	535.5878078	303.0242492
91.47646209	172.0270726	165.3505563	168.2340504	167.7619398	101.0080831
3.256126448	16.89622225	11.93399667	6.32093253	8.731956867	8.999933695
1575.79096	1032.162436	1180.818647	901.4622239	1454.631478	998.731608
720.9216417	1071.708889	5005.448905	2140.365	1243.105554	2614.860937
573.905899	971.8540941	1263.493925	817.8314243	614.7797794	1130.382593
21.78011002	49.43306684	41.33763908	71.96138573	41.67998504	12.4841451

123.0576216	283.7458037	190.5125975	139.0605157	174.0139376	144.1351298
1.165235886	5.63536962	0	4.220438028	2.292399177	5.981040425
140.4817096	131.4919578	212.9787057	294.652701	131.2919529	246.2781352
3.267016503	0	0	0	0	0
5.445027505	11.86393604	3.594577311	3.889804634	9.377996635	11.34922282
22.86911552	32.62582411	63.80374727	27.22863244	15.62999439	43.12704671
0	0	0.898644328	0	0	0
7.623038507	47.45574417	2.695932983	15.55921854	33.34398804	1.134922282
196.0209902	501.2512978	914.8199256	781.8507314	361.5738703	202.0161662
0	2.90666433	0.898644328	0	1.041999626	0
2.178011002	8.897952031	134.7966492	2.917353475	3.125998878	4.539689127
29.40314853	7.909290694	2.695932983	27.22863244	5.209998131	4.539689127
487.8744645	856.1807177	428.6533443	2405.844166	770.0377237	825.0884989
0	6.920629358	0	0	1.041999626	0
6.534033006	1.977322674	12.58102059	14.58676738	3.125998878	4.539689127
0	0	0	0	0	4.539689127
125.2356326	192.7889607	370.241463	170.1789527	172.9719379	123.7065287
3.267016503	20.76188807	29.65526282	0	7.293997383	47.66673584
199.1682161	177.4844832	171.9106599	177.7251737	197.3859892	334.8928669
61.49614064	115.0406332	152.7695357	247.3818502	71.57495432	78.30963745
168.7958527	97.87747234	56.61459265	107.9420786	107.3259615	141.8652852
249.3822597	428.0903588	236.3434582	713.7791503	540.797806	374.524353
1.089005501	0.988661337	0	4.862255792	0	0
634.8902071	241.2333662	17.07424223	62.23687414	416.7998504	11.34922282
6.534033006	39.54645347	26.0606855	13.61431622	21.88199215	14.75398966
74.237505	48.60259132	123.5815679	37.92559518	48.99482242	59.37913379
457.3823104	654.493805	415.1736794	530.9583325	601.2337843	343.8814514
65.34033006	58.33101887	87.16849979	77.79609268	71.8979742	59.01595865
0	0.988661337	2.695932983	0.972451158	3.125998878	0
125.2356326	155.2198299	288.4648292	230.4709246	159.4259428	72.63502604
1.089005501	4.943306684	1.797288655	22.36637664	3.125998878	3.404766845
216.7120947	82.05889096	55.71594832	80.71344615	118.7879574	140.7303629
388.7749639	786.9744241	2606.967195	261.5893616	628.3257746	492.5562703
4.356022004	22.73921075	0	30.14598591	15.62999439	1.134922282
239.6247704	89.38487146	10.78373193	10.70668725	12.50399551	19.7930446
0	4.943306684	3.594577311	4.862255792	1.041999626	2.269844564
5.445027505	1.977322674	1.797288655	0	1.041999626	6.809533691

161.1728142	63.27432556	106.0400307	67.09912993	105.2419622	122.5716064
1.089005501	0	0.898644328	0	0	0
43.56022004	50.42172818	29.65526282	42.78785097	66.68797607	49.9365804
6.534033006	10.8752747	6.290510294	6.807158109	8.335997009	7.944455973
481.3404315	493.3420071	150.9722471	287.8455429	547.0498037	180.4526428
14.15707151	0.988661337	6.290510294	2.917353475	10.41999626	4.539689127
108.9005501	149.2878619	209.3841284	225.6086688	137.5439506	196.3415548
51.18325855	115.6733764	35.94577311	841.1702521	48.97398243	53.34134725
11.97906051	6.920629358	0	0.972451158	0	0
59.89530256	35.59180813	2.695932983	3.889804634	0	4.539689127
0	0	2.16573283	1.089145297	0	0
69.69635207	64.26298689	235.4448139	105.0247251	101.0739637	28.37305705
0	0	919.3131473	0	0	0
1862.199407	1532.425072	396.3021485	1273.911018	1602.595425	780.8265299
0	0.988661337	0	0.972451158	0	0
0	7.583032453	8.08779895	4.1134684	5.209998131	7.081915039
75.14137957	166.0951046	102.4454534	141.9778691	134.4179518	116.896995
1978.232943	1043.690227	953.2549435	1129.511745	1572.419116	3116.099363
2545.005856	2810.764181	36430.1424	4618.170552	2244.467195	4506.776381
1.089005501	10.8752747	0.898644328	1.944902317	2.083999252	1.134922282
19.60209902	7.909290694	0	2.917353475	14.58799477	2.269844564
22.86911552	52.39905085	290.2621179	6.807158109	48.97398243	393.8180318
595.6860091	1053.912985	1229.34544	861.5917264	714.8117435	579.945286
16.33508252	8.897952031	4.493221639	13.61431622	7.293997383	3.404766845
8.712044008	23.72787208	29.65526282	26.25618128	20.83999252	22.69844564
870.1153953	1104.334713	1350.662425	3591.262128	1064.923618	1098.604769
11.97906051	8.897952031	0	2.917353475	8.335997009	14.75398966
0	0.988661337	0	6.807158109	0	0
58.16378381	31.30101792	32.3511958	128.441349	100.198684	22.69844564
264.6283367	138.4125872	211.181417	127.3911018	206.315926	258.7622803
18.51309352	75.1382616	17.97288655	65.15422762	30.21798916	28.37305705
1979.812001	3143.943051	5780.080316	2859.006406	2745.669015	1799.986739
1.089005501	1.977322674	1.797288655	0	0	0
87.12044008	162.1404592	150.0736027	145.8676738	94.82196598	104.4128499
26.13613202	11.86393604	0.898644328	0	3.125998878	3.404766845
178.5969022	642.6298689	190.5125975	259.6444593	229.2399177	299.6194824
2796.566127	3826.119373	6534.941551	3975.380336	3975.228574	6365.779079

0	0	13.47966492	1.944902317	0	2.269844564
409.4660684	431.0563429	685.6656221	541.6552953	518.9158138	348.4211405
2.178011002	0.988661337	0	0	0	0
27.22513753	16.80724273	6.290510294	11.6694139	7.293997383	11.34922282
55.53928055	106.7754244	157.2627574	84.60325079	86.48596897	89.65886026
67.51834106	66.24030957	48.5267937	71.96138573	81.27597084	125.0116893
916.9426319	469.614135	437.6397876	415.2366447	602.2757839	1393.684562
32.67016503	37.5691308	69.19561324	115.7216879	39.59598579	37.4524353
701.3195427	1047.981017	1569.931641	1511.1891	1197.25757	1517.391091
6.534033006	2.96598401	1.797288655	2.917353475	4.167998504	2.269844564
476.9844094	153.2425072	166.2492006	128.3635529	593.9397869	93.06362711
14.15707151	37.5691308	58.4118813	23.3388278	45.84798355	71.50010376
156.1524988	152.352712	44.87829773	29.2999534	0	52.54690165
78.40839607	66.24030957	170.7424223	93.35531121	97.94796486	136.1906738
43.56022004	113.6960537	68.29696891	63.2093253	36.46998691	13.61906738
0	0	85.37121113	0	0	0
231.6641402	181.8444797	72.00836998	117.4526509	126.0090148	231.1269227
15008.67382	7292.36602	8655.742165	4124.165363	7448.213328	11008.74613
0	0	0	1.944902317	0	0
467.1833599	804.7703282	2152.253165	1030.798228	473.0678303	515.2547159
0	0.988661337	0	1.944902317	0	0
39.20419804	47.45574417	79.97934517	64.18177646	46.88998318	38.58735758
1065.04738	1154.756441	36360.04814	108.9145297	1431.707486	6223.913794
0	0	0	0	14.58799477	0
1.089005501	3.954645347	22.46610819	3.889804634	1.041999626	3.404766845
0	0.988661337	130.3034275	2.917353475	0	0
0	2.96598401	1.797288655	0	1.041999626	0
265.7173423	1726.202694	537.389308	817.8314243	384.497862	576.5405192
0	0.988661337	1.797288655	0.972451158	3.125998878	34.04766845
157.9057976	76.12692293	102.4454534	87.52060426	91.6959671	101.0080831
283.1414303	166.0951046	7.189154622	29.17353475	183.3919342	89.65886026
62.07331356	66.24030957	26.95932983	57.37461835	89.61196785	65.82549235
320.9517013	318.5961158	546.6453446	451.9175024	315.8300867	294.6144751
120.8796106	209.5962034	526.605576	255.7546547	315.7258867	743.3740946
482.429437	762.2578907	999.2924924	1042.467642	624.157776	696.842281
47.91624205	66.24030957	95.25629874	52.51236256	37.51198654	48.80165812
10.89005501	0.988661337	0	0	1.041999626	0

2.178011002	0	2.695932983	0.972451158	2.083999252	0
27.22513753	55.36503486	33.24984013	55.42971603	51.05798168	88.52393798
125.2356326	30.64850144	39.54035042	36.95314402	37.51198654	88.52393798
57.71729155	5.931968021	10.78373193	0.972451158	5.209998131	15.88891195
6644.022562	2544.814281	2340.968474	1061.916665	4680.662321	10010.01453
82.76441808	40.53511481	183.3234429	339.3854543	45.84798355	153.214508
29.40314853	79.577351	71.89154622	41.81539981	51.20386163	49.9365804
0	0	0	72.93383688	1.041999626	0
111.0785611	242.2220275	364.8495971	315.0741753	171.9299383	76.03979288
327.7906558	197.7322674	249.8231231	306.3221149	226.1139189	409.7069437
196.0209902	280.7798197	1051.413863	314.1017242	236.5339151	208.8256999
107.8115446	186.8569927	374.7346847	376.3385983	134.4179518	321.1830058
4.356022004	0.988661337	0.898644328	2.917353475	1.041999626	0
4.356022004	1.977322674	466.3964061	1.944902317	1.041999626	1.134922282
534.701701	236.2900595	73.68883487	171.1514039	249.0379106	116.896995
15.24607701	40.53511481	44.03357206	35.98069286	41.67998504	23.83336792
395.3089969	519.0472018	1090.954214	594.1676578	388.6658605	520.9293274
2.178011002	0	2.695932983	626.4335873	0	2.269844564
199.2880067	535.8544446	760.2531013	612.6442298	483.4878265	271.2464254
75.14137957	174.0043953	272.2892313	166.2891481	144.837948	70.36518147
0	0	60.20916996	0	0	287.1353373
51.18325855	413.2604388	131.2020718	157.5370877	133.3759521	99.8731608
0	14.82992005	0	9.724511585	0	6.809533691
62.07331356	81.07022962	97.95223172	76.82364152	103.157963	251.9527466
0	1.977322674	0.898644328	0	3.125998878	1.134922282
1.089005501	0.988661337	0	0.972451158	0	0
50855.46789	47962.92743	31099.38425	6889.816458	44749.71594	50088.65999
89.29845108	130.5032965	220.1678603	99.19001816	123.9979555	38.58735758
177.769258	192.3737229	409.2336404	275.8941182	193.3117706	258.8871217
2.080000507	9.085797685	18.95240887	4.239887051	2.834238983	4.074370992
152.4607701	243.2106889	358.5590868	296.5976033	255.2899084	295.0797933
21.78011002	9.886613368	37.74306176	25.28373012	5.209998131	28.37305705
282.7820585	409.9879698	441.6297684	869.7992142	583.2384507	487.9257874
0	0	0	7.779609268	0	0
0	0.988661337	0	0	0	2.269844564
62.07331356	62.28566422	79.08070084	88.49305542	132.3339525	98.73823852
297.2985018	162.1404592	106.0400307	191.5728782	174.0139376	70.36518147

192.7539737	85.02487497	56.61459265	109.8869809	128.165954	60.15088094
20.69110452	23.72787208	38.64170609	39.8704975	27.09199028	19.29367879
951.7908079	1977.322674	2994.2829	3765.330886	4010.656561	613.9929545
358.2828098	710.8475012	1170.034915	1303.084552	394.9178583	1386.875028
510.74358	729.6320666	896.8470391	1099.84226	884.6576826	838.7075663
10.89005501	2.96598401	0	0	14.58799477	0
166.6178417	328.2355638	398.0994372	257.699557	304.2638908	273.5162699
5.445027505	7.909290694	0.898644328	2.917353475	4.167998504	6.809533691
126.3246381	213.5508488	186.0193758	167.2615993	210.4839245	102.1430054
195673.5974	221862.5246	209798.4034	23274.64603	83744.46795	44726.1522
15.24607701	34.60314679	36.84441744	29.17353475	20.83999252	19.29367879
211.2670672	290.666433	333.3970456	229.4984734	375.1198654	295.0797933
3.267016503	18.7845654	20.66881954	25.28373012	2.083999252	5.674611409
151.3717646	165.1064432	265.1000767	250.8923989	181.3079349	283.7305705
1171.769919	425.1243748	224.6610819	525.1236256	318.8518856	46.53181355
1.089005501	0	0	0	0	0
0	0	0	0.972451158	1.041999626	0
1010.597105	703.9268718	768.3409002	825.6110335	964.8916538	842.1123331
189.4869572	382.6119373	479.876071	287.8455429	391.7918594	178.1827982
3.267016503	11.86393604	195.9044634	20.42147433	1.041999626	4.539689127
55.44127006	77.77798737	91.88638251	132.8465528	75.41993294	68.34501981
26.13613202	41.52377615	31.45255147	59.31952067	31.25998878	19.29367879
173.1518747	258.0406089	762.9490342	246.0301431	182.3499346	155.4843526
129.5916546	195.7549447	172.5397109	131.2809064	172.9719379	166.8335754
205.8220397	125.5599898	43.13492773	104.052274	140.6699495	98.73823852
521.3396035	307.9383466	119.2680752	190.8532644	409.3703931	428.5466536
0	6.920629358	3.594577311	3.889804634	6.251997757	1.134922282
126.3246381	281.768481	2127.980782	299.5149568	278.2139002	157.742848
17.42408802	64.26298689	89.86443277	50.56746024	36.46998691	31.77782389
3.267016503	0.988661337	1.797288655	0.972451158	0	0
634.8902071	476.5347643	227.3570149	226.5811199	401.1698561	148.6748189
2.178011002	1.977322674	0	0.972451158	1.041999626	0
483.5184425	355.9180813	425.9574113	302.4323103	385.5398617	653.7152343
38.11519254	51.41038951	73.68883487	52.51236256	30.21798916	55.61119181
27.22513753	8.897952031	45.83086071	0	10.41999626	6.809533691
52.27226405	120.6166831	27.85797416	90.43795774	130.2499533	46.53181355
0	0	2.012963294	1.021073716	0	0

34.84817603	49.43306684	88.06714412	72.93383688	72.93997383	39.72227986
25.04712652	25.70519476	12.58102059	29.17353475	28.13398991	30.64290161
249.3822597	222.4488008	499.6462462	399.6774261	204.2319267	321.1830058
3.267016503	13.84125872	73.68883487	7.779609268	77.10797233	0
7540.274089	4059.443449	2188.198938	4031.782503	5239.17412	6517.858665
1.829529242	20.42574322	46.85531525	77.12510138	8.711116874	5.504373067
15.24607701	53.38771219	54.81730399	41.81539981	25.00799103	63.55564778
42.47121454	76.12692293	70.09425756	109.8869809	80.23397121	95.33347167
1180.481963	2050.483613	2015.659227	1800.979545	1847.465337	1372.121039
274.4293863	160.1631366	122.2156286	165.3166969	271.9619024	124.841451
145.9267371	131.4919578	292.9580508	251.86485	111.49396	175.9129537
8.712044008	37.5691308	26.95932983	16.53166969	15.62999439	54.47626953
0	0	0	0	0	1.134922282
0	1.977322674	0	0	0	2.269844564
300.5655183	292.6437557	356.7617981	221.7188641	207.3579256	321.1830058
203.6440287	265.9498996	516.7204884	252.8373012	242.7859129	315.5083943
9.801049509	20.76188807	40.43899475	9.724511585	5.209998131	3.404766845
234.1361827	98.86613368	139.2898708	114.7492367	131.2919529	162.2938863
209.0890562	100.8434564	130.3034275	76.82364152	108.3679611	125.9763733
0	0	0.898644328	0	2.083999252	1.134922282
191.425387	180.6976325	425.4361976	158.3247731	161.551622	523.5169502
358.2828098	449.8409082	890.5565288	411.34684	394.9178583	514.1197937
79.49740157	103.8094404	114.1278296	265.4791663	122.9559559	71.50010376
270.4545162	422.3363499	524.8622061	317.0190777	367.0443683	292.8099487
366.9948538	283.7458037	261.5054994	274.2312267	382.4138628	270.1115031
3226.7233	2128.587858	2193.590804	1415.888887	2552.899084	4421.65721
0	0.988661337	2.695932983	11.6694139	1.041999626	5.674611409
474.8063984	430.0676815	246.2285458	477.4735188	536.6298075	796.7154418
10.89005501	26.69385609	17.97288655	14.58676738	13.54599514	13.61906738
0	0	0	0	1.041999626	2.269844564
0	0.988661337	5.391865966	0	0	0
0	0	0	0.972451158	0	0
88.20944558	133.4692805	156.364113	218.8015107	141.7119492	132.785907
0	0	0	1.944902317	2.083999252	0
161.1728142	58.33101887	49.42543803	64.18177646	82.31797046	68.09533691
160.0838087	226.4034461	180.6275099	269.3689709	282.3818987	203.1510884
0	0	0	0	0	0

0	0.988661337	0	1.944902317	2.083999252	1.134922282
4.356022004	2.96598401	7.189154622	48.62255792	5.209998131	1.134922282
11.97906051	16.80724273	19.77017521	41.81539981	17.71399364	21.56352335
135.0366821	65.25164823	45.83086071	68.07158109	79.19197159	77.17471516
95.83248409	313.4056438	89.86443277	115.7216879	101.0739637	205.420933
1.089005501	0	0	8.752060426	0	1.134922282
2.178011002	0.988661337	0	0	0	0
94.74347859	171.0384113	121.3169842	92.38286005	171.9299383	146.4049744
0	1.977322674	0	0	4.167998504	0
0	0	888.7592401	0	0	1.134922282
15412.69486	11447.70962	3124.586328	13271.04096	14699.48873	5008.41203
932.1887089	843.3281203	759.3544569	573.7461835	844.0196972	1178.049329
70.78535757	150.2765232	540.085241	782.8231826	141.7119492	246.2781352
127.4136436	311.4283211	274.9851643	378.2835006	216.7359222	245.1432129
11.97906051	6.920629358	0	0.972451158	6.251997757	0
15.24607701	13.84125872	53.91865966	3.889804634	13.54599514	23.83336792
2243.351332	1636.234512	728.8005498	1885.582796	2496.631104	1955.471092
419.2671179	1045.015033	629.0510294	1171.803646	882.5736833	315.5083943
155.7277866	227.3921075	286.6675405	247.9750454	197.979929	183.8574097
59.48148047	80.08156828	74.34484523	108.0490482	73.51307362	44.18252443
0	0.988661337	0	0	0	72.63502604
18.51309352	2.96598401	0	0	8.335997009	1.134922282
3.267016503	14.82992005	12.58102059	12.65158957	11.46199589	6.809533691
250.4712652	94.91148833	23.36475252	74.8787392	123.9979555	94.19854939
0	0	0	0.972451158	0	0
380.0629199	668.3350637	651.5171376	1112.484125	751.2817304	453.9689127
1.089005501	1.977322674	4.493221639	0.972451158	0	3.404766845
664.2933556	657.459789	1612.167924	1225.28846	699.1817491	569.7309855
17.42408802	37.5691308	83.57392248	23.3388278	52.09998131	3.404766845
136.1256876	71.18361625	82.67527815	86.5481531	98.98996448	103.2779276
204.7330342	477.5234257	645.2266273	393.8427192	362.6158699	196.3415548
334.3246888	417.2150841	582.3215244	352.9997705	405.3378546	635.5564778
5.445027505	24.71653342	16.1755979	17.50412085	14.58799477	13.61906738
275.5183918	418.2037455	531.0987977	524.1511744	422.0098486	387.0084981
399.6650189	435.9996495	604.7876326	765.3190617	453.2698374	369.9846639
4.356022004	8.897952031	5.391865966	6.807158109	8.335997009	4.539689127
55.53928055	113.6960537	62.90510294	61.26442298	97.94796486	85.11917114

494.114466	335.3638121	303.0138809	256.8924225	348.5801349	238.8443942
2.178011002	0	0	0	0	0
94.74347859	116.6620377	212.9787057	149.7574784	112.5359596	119.1668396
118.7015996	147.3105392	253.4177004	176.9861108	120.8719566	129.3811401
20.69110452	17.79590406	64.7023916	18.47657201	32.30198841	14.75398966
211.0165959	33.01140204	49.57820756	46.91104388	48.44256262	68.51525815
1220.775167	2827.571423	1436.93228	2433.072798	2305.945173	1894.185288
268.9843588	201.6869127	379.2279063	729.3383688	332.3978807	263.3019694
0	0.988661337	0	0	0	0
1.089005501	19.77322674	20.66881954	11.6694139	4.167998504	17.02383423
137.2146931	163.1291206	371.1401074	267.4240686	147.9639469	164.5637309
201.4660177	80.08156828	106.938675	161.4268923	180.2659353	155.4843526
1821.906203	2273.921075	3567.617981	4518.008082	2760.25701	3575.005188
88.20944558	139.4012485	188.7153088	104.052274	104.1999626	121.4366842
0	2.96598401	0.898644328	3.889804634	0	0
41.38220904	13.84125872	25.16204118	10.69696274	34.38598766	17.02383423
230.8691662	329.2242252	479.876071	630.1483507	268.8359035	640.096167
18.51309352	91.94550432	50.32408235	49.59500908	90.65396747	77.17471516
50.09425305	49.43306684	95.25629874	17.50412085	13.54599514	76.03979288
3.267016503	0.988661337	0	0	0	1.134922282
2.178011002	0	0	0	0	0
466.0943544	751.382616	1328.196316	1364.348975	509.5378172	1483.343422
101.2775116	81.07022962	11.68237626	51.5399114	76.06597271	27.23813476
2.178011002	8.897952031	8.986443277	12.64186506	10.41999626	1.134922282
150.2827591	275.836513	256.1136334	243.1127896	286.5498972	223.5796895
0	0.988661337	0	0	0	2.269844564
453.0262884	301.5417077	177.0329326	181.8483666	352.1958736	531.1436279
657.7593226	1084.561486	861.7999103	1353.652013	836.7256998	899.9933695
20.69110452	12.85259738	0	0	0	0
13.06806601	26.69385609	35.94577311	51.5399114	20.83999252	22.69844564
0	0	0	0	0	0
63.16231906	83.04755229	99.74952038	494.9776397	37.51198654	18.15875651
0	0	0	0.972451158	37.51198654	0
91.47646209	29.6598401	31.45255147	26.25618128	36.46998691	63.55564778
127.2720729	83.04755229	0	0	2.083999252	5.674611409
62.05153345	154.9726645	49.42543803	103.0798228	86.48596897	90.48735353
0	0	7.189154622	0.972451158	0	0

180.7749132	649.5504983	232.7488809	337.440552	360.5318706	366.579897
0	0	5.391865966	1.944902317	0	0
224.3351332	420.1810681	341.4848445	1245.709934	427.2198467	281.4607259
84.94242908	83.04755229	118.6210513	95.30021353	97.94796486	215.6352335
317.9896063	495.3193297	297.4512725	582.4982439	294.8858942	1021.430054
121.9686161	159.1744752	328.9038239	421.0713516	579.3517921	70.36518147
0	1.977322674	0	3.520273194	3.125998878	3.416116068
3.680838593	8.789199284	6.155713645	4.531622398	4.657738329	8.943187581
175.3298857	331.2015478	503.2408235	349.1099659	303.2218912	182.7224874
98.01049509	123.5826671	161.755979	158.5095388	122.9559559	223.5796895
842.8902578	634.7205782	375.633329	1032.74313	584.5617903	322.317928
126.3246381	246.0283737	353.9939736	436.2707632	87.52796859	111.1429391
80.58640708	68.21763224	246.2285458	141.9778691	27.09199028	37.4524353
630.5341851	228.3807688	1073.879972	100.1624693	163.5939413	158.8891195
0	3.954645347	2.695932983	0	0	0
1.089005501	1.977322674	4.493221639	0.972451158	1.041999626	0
320.1676173	361.8500493	334.2956899	540.6828441	386.5818613	441.4847676
555.3928055	641.6412076	1217.663064	811.9967173	665.8377611	671.8739908
0	0	0	0.972451158	1.041999626	0
0	1.977322674	0.898644328	0.972451158	0	1.134922282
0	0	0.898644328	0.972451158	2.083999252	0
0	0	0.386417061	0	1.062839619	1.157620727
0	0	0.898644328	0	1.041999626	0
0	0	0	0	1.041999626	0
0	0	0	0.972451158	0	0
164.4398307	145.3332165	256.1136334	241.1678873	168.8039394	139.5954407
1722.806703	5599.777812	2278.063371	480.3908723	7033.497476	11853.12831
125.2356326	154.2311685	237.0533872	342.1763891	98.98996448	239.4686015
8.712044008	22.73921075	16.1755979	36.95314402	8.335997009	20.42860107
27.22513753	50.42172818	56.61459265	73.90628804	36.46998691	131.6509847
0	0	1.114318966	0	1.041999626	0
2.178011002	0.988661337	2.695932983	1.944902317	3.125998878	1.134922282
0	1.977322674	0.898644328	0.972451158	2.083999252	0
37.02618703	28.67117877	0	10.69696274	1.041999626	1.134922282
0	2.96598401	0	1.944902317	0	1.134922282
67.51834106	141.3785712	98.85087605	73.90628804	140.6699495	60.15088094
1.089005501	3.954645347	0	5.834706951	1.041999626	1.134922282

2.178011002	0.988661337	0.898644328	0.972451158	1.041999626	2.269844564
0	0	0	0	5.439238048	0
350.6597713	374.7026467	377.4306176	410.3743889	581.4357914	470.992747
0	0	0.898644328	0.972451158	1.041999626	9.079378255
4028.231348	3200.296747	1445.918723	3524.162998	3024.924915	2776.019901
0	1.977322674	63.80374727	0	0	0
150.2827591	211.5735261	334.2956899	350.082417	224.0299196	194.0717102
185.1309352	368.7706786	478.9774267	388.9804634	364.6998691	212.2304667
111.0785611	87.00219764	83.57392248	65.15422762	81.27597084	107.8176168
28.31414303	125.5599898	453.8153855	185.7381713	46.88998318	59.01595865
315.8115953	264.9612383	560.7540605	596.1125601	319.8938852	286.000415
91.41112176	68.07921965	75.57598796	50.16875526	87.07990875	88.84171622
0	0	0	0.972451158	0	0
150.2827591	114.6847151	397.2007929	146.8401249	134.4179518	180.4526428
1.089005501	0	0	0	1.041999626	0
11.97906051	12.85259738	59.31052563	38.89804634	4.167998504	20.42860107
0	1.482992005	0	0.486225579	1.041999626	1.134922282
0	0	0	0	4.272198467	24.18519383
26.13613202	36.58046946	65.60103592	40.84294866	25.00799103	35.18259074
382.2409309	369.75934	647.9225603	556.2420626	454.311837	363.1751302
2.178011002	4.943306684	74.49761477	1.944902317	1.041999626	0
285.3194413	463.682167	407.0858805	506.6470536	419.9258493	187.2621765
79.49740157	50.42172818	35.04712878	55.42971603	92.73796672	53.34134725
246.1152432	117.6506991	72.79019055	168.2340504	50.01598205	160.0240417
6.534033006	5.931968021	1.797288655	0.972451158	1.041999626	4.539689127
19.60209902	10.8752747	3.594577311	11.6694139	16.67199402	25.74003735
0	0	0	59.16392848	153.6428449	1.883970988
19.60209902	73.16093892	17.97288655	30.14598591	22.92399177	20.42860107
26.13613202	47.45574417	47.62814937	47.65010676	48.97398243	26.10321248
16.33508252	17.79590406	117.7224069	5.834706951	23.9659914	12.4841451
129.5916546	150.2765232	59.31052563	60.29197182	63.56197719	53.34134725
175.3298857	178.947702	342.3834889	253.8097524	220.9039207	114.6271505
165.5288362	269.9045449	262.4041437	239.222985	193.8119305	274.6511922
0	3.954645347	0	0	3.125998878	0
302.7435293	395.4645347	625.4564521	245.0576919	453.2698374	337.0719177
0	0.988661337	0	0	1.041999626	2.269844564
70.78535757	130.5032965	137.4925821	62.23687414	123.9979555	186.1272542

60.98430806	12.85259738	8.986443277	31.11843707	21.88199215	18.15875651
81.67541258	68.21763224	52.12137101	40.84294866	44.80598392	49.9365804
0	0.988661337	0	0.972451158	0	2.269844564
0	1.977322674	0	0	0	0
22.86911552	36.58046946	54.81730399	48.62255792	17.71399364	26.10321248
462.8273379	736.5526959	1041.528776	2495.309673	959.6816557	498.2308817
0	0	0	0.972451158	0	0
188.3979517	230.3580915	297.4512725	213.9392549	150.0479462	177.047876
2.178011002	10.8752747	2.695932983	9.724511585	4.167998504	1.134922282
0	0.988661337	0	0	0	0
209.0890562	274.8478516	492.4570916	359.8069286	233.4079163	254.2225911
18.51309352	72.17227759	22.46610819	74.8787392	31.25998878	9.079378255
53.36126955	92.93416566	60.20916996	83.63079963	130.2499533	44.26196899
23.95812102	12.85259738	2.695932983	2.917353475	8.335997009	5.674611409
1.089005501	0	1.797288655	0.972451158	1.041999626	1.134922282
258.0943037	191.8002993	177.9315769	571.8012812	276.1299009	259.8972025
101.2775116	125.5599898	124.0129172	255.7546547	135.4599514	59.01595865
2.178011002	13.84125872	17.07424223	1.944902317	3.125998878	9.079378255
516.1886075	572.434914	711.7263076	1798.062192	760.6597271	676.41368
5.445027505	1.977322674	0	0.972451158	0	1.134922282
16.33508252	33.61448545	10.78373193	68.07158109	15.62999439	6.809533691
2.090890562	0	8.635971989	4.589969468	9.013296766	6.798184468
45.73823104	46.46708283	1162.84576	17.50412085	62.51997757	688.8978251
3579.561082	1695.554193	1288.655966	1916.701233	2367.423151	3447.893892
0	0	0.898644328	0	0	0
8.712044008	7.909290694	10.78373193	9.724511585	8.335997009	12.4841451
1.089005501	2.96598401	5.391865966	1.944902317	8.335997009	2.269844564
245.8865521	465.4024377	904.8359871	398.8119446	412.0587521	340.7490659
95.83248409	83.04755229	620.0645861	112.8043344	188.6019323	140.7303629
484.607448	598.1401088	673.0846015	1713.458941	666.8797607	728.6201049
0	0	0	22.36637664	0	0
319.906256	99.52853678	69.61797607	15.25775868	326.3855429	112.3232582
30.49215403	20.76188807	7.189154622	7.779609268	25.00799103	31.77782389
0	0	0	0	0	12.52954199
3.267016503	2.96598401	0	0	0	1.134922282
1188.105002	2177.032264	3692.529543	1581.205584	2020.437275	1321.049536
2505.801658	1040.071726	1190.703734	1807.786704	1428.581487	2403.765393

759.0368342	1355.454693	1018.164023	2370.835924	959.6816557	730.8899495
47.9271321	75.74134501	87.9593068	71.99055926	137.9503305	209.8357807
985.5499784	766.212536	829.4487145	1092.062651	849.2296953	602.6437317
205.8220397	110.7300697	141.9858038	121.5563948	143.7959484	103.2779276
115.4345831	168.0724273	349.5726435	155.5921854	165.6779406	154.3494303
14.15707151	0.988661337	3.594577311	0	1.041999626	1.134922282
38.11519254	94.91148833	65.60103592	98.217567	77.10797233	166.8335754
47.91624205	221.4601394	110.5332523	261.5893616	177.1399364	110.0874613
899.5185438	1432.570277	1720.903888	2142.309902	1809.953351	895.4536804
703.5846741	442.1392364	583.1482772	257.8940472	443.266641	874.5370627
0	10.8752747	0	270.3414221	3.125998878	4.539689127
274.4293863	150.2765232	78.18205651	240.1954361	37.51198654	10.21430054
53.36126955	38.55779214	11.68237626	22.36637664	26.04999065	11.34922282
114.3455776	68.21763224	84.47256681	72.93383688	76.06597271	128.2462178
0	0	0	0	0	0
218.8901057	214.5395101	230.9515922	219.7739618	161.509942	246.2781352
71.87436307	32.62582411	8.08779895	8.752060426	18.75599327	31.77782389
101.2775116	141.3785712	130.3034275	181.8483666	87.52796859	48.80165812
177.5078967	234.3127368	303.7417828	294.652701	210.4839245	368.8497416
57.71729155	11.86393604	10.78373193	16.53166969	25.00799103	21.56352335
96.92148959	154.2311685	453.2222802	142.9503203	128.165954	80.57948201
193.8429792	207.6188807	225.7664145	273.2587755	246.8601314	280.7230264
28.31414303	107.7640857	41.33763908	43.76030213	9.377996635	11.34922282
107.8115446	116.6620377	253.4177004	189.6279759	119.829957	166.8335754
0	0	0.898644328	0	0	1.134922282
1.089005501	11.86393604	8.08779895	0.972451158	7.293997383	15.88891195
21.78011002	151.2651845	34.14848445	99.19001816	47.9319828	18.15875651
0	1.047981017	0	3.053496638	2.177779219	1.180319173
8.712044008	12.85259738	14.37830924	141.005418	5.209998131	3.404766845
0	0	0.898644328	0.972451158	0	0
8907.749187	12268.77309	13830.02837	21835.22382	11552.92077	21960.79155
52.27226405	36.58046946	39.54035042	74.8787392	35.42798729	41.99212443
0	0.988661337	4.493221639	0	2.083999252	0
376.7959034	106.7754244	53.02001534	276.176129	430.3458456	118.0319173
516.1886075	343.0654839	146.4790254	246.0301431	427.2198467	631.0167887
158.9948031	225.4147848	159.060046	239.222985	222.98792	157.7541972
752.5028012	829.4868616	1044.224709	816.8589731	964.8916538	552.7071512

132.8586711	158.1858139	305.5390714	173.0963062	169.8459391	132.785907
109.9895556	80.08156828	248.0258345	206.1596456	105.2419622	158.8891195
2412.147185	5132.140999	4007.055057	3949.124154	4802.576277	3525.068607
11.97906051	13.84125872	39.54035042	21.39392549	11.46199589	7.944455973
7.623038507	6.920629358	0.898644328	1.944902317	2.083999252	3.404766845
252.6492762	165.1064432	170.7424223	223.6637664	242.7859129	191.8018656
0	5.931968021	0	0.972451158	1.041999626	0
310.3665678	440.9429562	404.3899475	497.8949931	367.825868	241.738446
75.14137957	56.3536962	23.36475252	35.0082417	97.94796486	56.74611409
163.3508252	168.0724273	203.9922624	203.2422921	202.1479275	108.9525391
4288.503663	2919.516928	1741.572707	3839.237174	5044.32019	2995.059902
93.65447309	564.5256233	142.8844481	171.1514039	1648.443409	226.9844564
853.7258625	1081.51641	1681.363537	830.4732893	1096.131507	945.3902608
253.7382817	328.2355638	527.5042204	514.4266628	335.5238796	468.7229024
13.06806601	87.00219764	8.08779895	49.59500908	10.41999626	1.134922282
27.22513753	33.61448545	45.83086071	33.06333939	42.72198467	36.31751302
1353.644728	2509.222473	2712.28831	1829.307048	2369.51757	1460.769818
3.267016503	88.97952031	203.0936181	20.42147433	10.41999626	10.21430054
16.33508252	31.63716278	19.77017521	22.36637664	22.92399177	19.29367879
386.5969529	332.1902092	678.4764674	909.2418332	540.797806	516.3896382
20.69110452	9.886613368	3.594577311	5.834706951	7.293997383	17.02383423
27.22513753	38.55779214	34.14848445	44.73275329	19.7979929	43.12704671
148.1047481	180.9250246	209.3841284	123.5012971	141.7119492	258.7622803
62.57425609	116.6422645	664.0801853	151.6440336	157.9150433	161.9080127
54.45027505	87.00219764	97.95223172	136.1431622	116.7039581	68.09533691
38.11519254	21.75054941	84.47256681	60.29197182	41.67998504	91.92870483
34.84817603	12.85259738	239.380876	73.49785856	141.4097693	19.29367879
2.363141937	0	0	0.379255952	1.041999626	0
27.22513753	11.86393604	5.391865966	3.889804634	2.083999252	5.674611409
764.4818617	221.4601394	352.2685765	535.8205883	390.7498598	280.3258036
34.84817603	29.6598401	691.057488	50.56746024	18.75599327	45.39689127
127.4136436	173.0157339	401.6940145	182.8208178	184.4339338	181.5875651
11.97906051	15.81858139	12.61696636	6.807158109	10.41999626	18.15875651
0	8.897952031	1.797288655	1.944902317	1.041999626	1.134922282
141.5707151	139.4012485	115.0264739	63.2093253	65.64597645	59.01595865
29.54471924	25.83372073	43.43148036	2.917353475	27.16493025	17.25081868
142.6597206	170.0497499	139.2898708	141.005418	131.2919529	130.5160624

95.74536365	127.3395802	228.7229543	102.019851	181.7455748	83.68916906
4.356022004	11.86393604	34.14848445	4.862255792	9.377996635	24.9682902
8.712044008	30.64850144	29.65526282	21.39392549	25.00799103	24.9682902
2.178011002	0.988661337	0	2.917353475	3.125998878	3.404766845
0	0	0	0	0	0
192.7539737	197.7322674	97.05358739	39.8704975	242.7859129	300.7544047
6.446912566	19.27889607	9.552589204	7.750435733	20.98587247	20.7917762
1.089005501	10.8752747	10.78373193	3.889804634	4.167998504	32.91274617
0	1.651064432	1.500736027	0	0	0
243.7303212	281.3038102	575.9950683	458.9969468	286.1435173	382.2531737
116.5235886	13.84125872	3.594577311	130.3084552	0	87.3890157
1.089005501	0	52.12137101	0	0	2.269844564
1943.874819	691.0742744	316.3228034	789.6303407	1311.877529	1240.470054
175.3298857	267.9272223	282.1743189	448.299984	219.8619211	204.2860107
11.97906051	4.943306684	1.797288655	1.944902317	4.167998504	1.134922282
231.9581717	257.0519476	526.605576	252.8373012	314.6838871	329.1274617
93.65447309	179.9363633	236.3434582	163.3717946	161.509942	255.3575134
1074.84843	1612.50664	1510.621115	1386.715352	1509.857458	2024.701351
75.14137957	63.27432556	31.45255147	43.76030213	91.6959671	22.69844564
18.51309352	25.70519476	88.96578845	17.50412085	22.92399177	83.98424886
43.56022004	33.61448545	292.0594065	25.28373012	50.01598205	387.0084981
278.7854083	421.1697295	468.1936947	493.0327373	408.4638534	283.7305705
323.4346338	490.3760231	682.4754347	484.2806769	525.1678116	255.3575134
51.18325855	17.79590406	0	11.6694139	23.9659914	3.404766845
1.089005501	0.988661337	0	0	1.041999626	0
211.2670672	109.7414084	148.2763141	71.96138573	156.2999439	127.1112956
103.4555226	120.6166831	167.147845	141.9778691	145.8799477	105.5477722
0	0	0	0	0	0
14.15707151	23.72787208	15.27695357	47.65010676	13.54599514	23.83336792
2927.246787	1894.275121	1753.255083	2124.805781	2365.339151	814.8741984
0	0.988661337	0	0	0	0
0	8.897952031	0.898644328	0	0	0
111.0785611	119.6280218	225.5597263	130.3084552	151.0899458	474.3975138
292.9424798	386.5665827	372.937396	388.0080122	509.5378172	324.5877726
0	0	0.898644328	0	0	0
0	0	1.797288655	3.889804634	1.041999626	0
0	0	0	0.972451158	0	0

716.5656197	368.7706786	413.3763908	310.2119195	392.833859	643.5009338
2.178011002	6.920629358	188.7153088	1.944902317	1.041999626	1.134922282
267.8953533	471.5914577	643.4293387	502.7572489	390.7498598	539.0880839
404.0210409	850.2487497	1134.089142	783.7956337	743.987733	660.524768
682.8064491	591.2194794	91.66172143	680.7158109	418.8838497	226.9844564
54.45027505	79.09290694	96.15494307	71.96138573	76.06597271	175.9129537
11.97906051	2.96598401	2338.272541	2.917353475	2.083999252	13.61906738
38.11519254	54.37637352	52.08542524	53.88351869	66.0627763	30.79044151
2.178011002	0	0.898644328	4.862255792	1.041999626	5.674611409
0	0	0	0	0	0
119.7906051	367.7820173	49.42543803	1981.855461	195.8959297	12.4841451
463.9163434	462.6935056	2004.875495	1220.426204	514.7478153	2721.543632
478.0734149	961.9674807	404.3899475	241.1678873	1136.821592	889.779069
0	0	0	3.889804634	0	6.809533691
190.5759627	146.3218778	289.3634735	321.8813334	138.5859503	156.6192749
65.34033006	67.2289709	162.6546233	55.42971603	91.6959671	186.1272542
77.31939057	118.6393604	77.28341218	129.3360041	94.82196598	51.07150268
92.56546759	110.7300697	128.5061389	206.1596456	130.2499533	132.785907

		HCC			
HCC6	HCC7	HCC8	HCC9	HCC10	HCC11
0	0	0	0	0	0
185.1079493	254.7190579	517.6783699	419.6852947	389.2802243	400.1451714
38.29819642	91.11104764	163.8417426	79.08642487	82.77054603	187.4184221
2.234061458	0	1.107662485	3.912141817	8.703840231	3.816769327
0	0	0	0	1.293289782	0
0	1.959377369	0	0	0	0
0	0.979688684	0	0	0	1.36801768
70.2133601	48.00474553	160.7649024	128.6472511	121.5692395	69.08489284
28.72364731	122.4610855	213.8403965	161.3363067	292.2834907	154.5859978
435.6419842	313.500379	2604.545261	94.90370985	1276.477015	1724.386286
54.25577826	75.43602869	130.7657101	105.4485665	108.6363417	56.08872488
39.8939546	89.15167027	127.6888698	62.21465423	63.37119931	119.0175382
81.38366738	277.2518977	89.22836687	29.52559862	142.261876	113.5454674
36.60669274	59.76100974	101.2126596	74.86848221	78.63201873	56.58121124
146.8097529	112.6641987	276.9156213	132.8651938	209.5129446	173.7382454
97.34124922	61.72038711	122.3043994	43.23391226	58.19804018	58.82476024
15.95758184	27.43128316	9.99973077	70.65053955	27.15908542	41.0405304
31.91516368	47.02505685	107.6894083	35.85251261	55.61146062	55.40471604
205.8528057	114.6235761	272.300361	90.68576719	173.3008308	123.1215912
0	0	0	0	1.293289782	0
25.62787643	6.887211451	40.16814929	36.66446657	5.199024923	22.00456438
73.40487646	78.37509475	102.3049379	132.8651938	62.07790952	49.93264532
82.97942557	75.43602869	143.073071	143.4100504	129.3289782	51.300663
36.70243823	36.24848132	61.53680474	63.2691399	37.50540367	36.93647736
57.44729462	48.00474553	119.9967692	101.2306238	54.31817083	81.39705196
67.02184373	62.7000758	119.9967692	81.1953962	78.89067669	77.29299892
0	0	0	0	0	1.36801768
38.29819642	26.45159448	64.61364497	24.25317029	51.73159127	27.3603536
17473.55211	6546.279789	10533.56255	3072.771228	17127.03658	4521.298432
103.1019363	63.67976448	111.5277665	80.14091054	91.8235745	55.34999533
98.84126192	48.97463733	190.7640947	74.81575793	86.63748248	71.79356784
108.2721928	56.00880208	150.0421142	69.59605389	102.389752	46.21163723
296.8110222	158.7095669	96.92046746	235.1503033	265.1244053	245.5591735
15.95758184	62.7000758	113.0738787	85.41333886	67.25106865	62.24480444

51.06426189	41.14692474	99.22809764	36.90699827	50.43830149	45.14458344
59.04305281	53.88287764	181.533574	97.01268118	117.6893701	87.55313152
103.724282	30.37034921	76.15179586	145.5190218	125.4491088	49.93264532
68.61760191	45.06567948	155.380432	75.92296788	73.71751756	70.45291052
0	5.878132106	4.615260355	2.10897133	2.586579563	3.4200442
57.44729462	95.02980238	237.6859083	186.6439627	72.42422778	137.4857768
70.2133601	83.27353817	163.8417426	99.12165251	80.18396647	86.18511384
106.9157983	97.96886843	256.1469497	122.3203371	82.77054603	127.9096531
49.4685037	2.939066053	9.230520711	8.43588532	20.69263651	17.78422984
82.97942557	92.09073633	252.3008994	99.12165251	80.18396647	114.9134851
84.57518375	34.28910395	139.9962308	65.37811123	81.47725625	56.77273372
829.7942557	633.8585788	451.5263048	510.3710618	585.8602711	341.3204111
0	0	0	0	0	1.36801768
375.0031732	326.2363319	199.2254053	628.4734563	587.1535609	124.4896089
76.59639283	100.9079345	493.832858	83.30436753	161.6612227	134.7497415
111.7030729	100.9079345	196.9177752	119.1568801	142.261876	95.76123759
525.0044425	253.7393692	593.8301657	424.957723	344.0150819	326.2722167
25.53213094	29.39066053	73.84416569	23.19868463	43.97185258	81.39705196
41.48971278	12.7359529	30.76840237	27.41662729	32.33224454	4.78806188
213.8315967	204.754935	210.7635562	224.6054466	259.9512461	231.8789967
0	0.979688684	2.307630178	2.10897133	5.173159127	3.4200442
30.3194055	31.3500379	61.53680474	68.54156822	29.74566498	58.1407514
6.383032736	12.7359529	11.53815089	7.381399655	3.879869345	38.98850388
74.23467072	204.0691529	118.0506678	108.8229206	162.2302702	219.1906328
351.0668005	342.8910395	323.0682249	847.8064746	350.4815309	203.1506255
0	0.979688684	0	0	0	0
43.08547097	51.92350027	26.92235207	15.81728497	32.33224454	53.35268952
169.1503675	243.9424824	462.2952456	501.9351765	382.8137754	207.2546785
20.74485639	25.47190579	45.38339349	21.0897133	27.15908542	20.5202652
110.1073147	123.4407742	314.6069142	120.2113658	133.2088475	145.6938829
124.4691384	168.5064537	149.9959615	160.2818211	312.9761272	117.6495205
79.7879092	36.24848132	90.76678699	67.48708256	82.77054603	63.61282212
0	0	0	0	0	0.68400884
0	0	3.846050296	1.054485665	2.586579563	3.4200442
0	0	0	0	0	0.68400884
22.34061458	36.24848132	56.92154438	30.58008428	41.38527302	34.88445084
186.7037075	163.6080103	595.3685858	183.4805057	297.4566498	261.2913769

0	0	0	0	0	0
900.0076158	794.527523	1056.894621	756.0662218	869.0907333	814.6545284
252.1297931	61.72038711	90.76678699	785.5918204	75.01080734	95.07722875
9.574549104	15.67501895	44.61418344	22.14419896	23.27921607	25.99233592
0	0	3.076840237	2.10897133	2.586579563	2.05202652
19.14909821	97.96886843	94.61283728	110.7209948	69.83764821	75.2409724
296.8110222	66.61883053	82.30547634	260.4579592	77.5973869	256.503315
3.191516368	0	0	0	0	0
19.14909821	28.41097185	46.15260355	53.77876891	40.09198323	22.57229172
30.3194055	77.39540606	44.61418344	27.41662729	23.27921607	15.73220332
98.93700741	77.39540606	93.84362723	221.4419896	140.9685862	145.6938829
87.76670012	131.2782837	329.2219053	205.6247047	215.9793936	340.6364023
57.44729462	41.14692474	111.5354586	79.08642487	43.97185258	62.24480444
1650.013962	2415.912296	3796.820852	515.6434902	919.5290348	2726.459236
6057.498066	1484.228357	3327.602716	6596.86232	2012.3589	1770.898887
22.34061458	30.37034921	39.99892308	33.74354128	63.37119931	38.98850388
301.5982968	358.5660585	446.1418344	431.284637	446.1849747	276.3395713
100.5327656	417.3473795	506.140219	633.7458846	413.8527302	551.9951338
15.95758184	19.59377369	96.92046746	44.28839793	25.86579563	52.66868068
19.14909821	47.02505685	37.6912929	31.63456995	28.4523752	28.72837128
9.574549104	14.69533026	13.84578107	9.490370985	14.2261876	12.31215912
3.191516368	0.979688684	0	26.36214162	0	0
46.27698734	29.39066053	91.53599705	39.0159696	64.66448909	36.93647736
57.44729462	43.10630211	186.9180444	61.16016857	53.02488105	62.24480444
30.3194055	42.12661343	63.84443492	61.16016857	29.74566498	80.71304312
54.25577826	41.14692474	106.1509882	101.2306238	36.21211389	77.29299892
2277.146929	617.2038711	1766.875506	1195.786744	4055.756756	819.4425903
0	0	0	0	0	0
62.23456918	31.3500379	187.6872545	130.7562225	47.85172192	82.76506964
0	0	0	0	0	0
90.95821649	130.298595	134.6117604	219.3330183	134.5021373	178.5263072
274.4704076	227.2877748	1049.202521	787.7007917	464.2910316	342.00442
189.8952239	64.65945317	189.2256746	94.90370985	82.77054603	71.13691936
43.08547097	31.3500379	99.22809764	36.90699827	27.15908542	21.20427404
55.85153644	63.67976448	122.3043994	90.68576719	80.18396647	91.65718455
0	0	0	1.054485665	0	2.05202652
0	0	2.307630178	0	0	5.47207072

0	0.979688684	2.307630178	0	0	4.78806188
17.55334002	6.85782079	38.46050296	10.54485665	15.51947738	21.20427404
119.6818638	143.0345479	176.9183136	225.6599323	151.3149045	142.2738387
3834.606916	3213.378885	2987.61187	3103.351312	2540.021131	3590.362401
27.12788913	21.55315106	8.461310651	12.65382798	7.75973869	6.15607956
27.12788913	29.39066053	63.07522486	36.90699827	40.09198323	20.5202652
33.51092186	75.43602869	153.8420118	90.68576719	36.21211389	69.08489284
193.0867403	95.02980238	181.533574	121.2658515	148.7283249	365.2607205
167.5546093	148.91268	166.9185829	130.7562225	103.4631825	120.3855558
170.7461257	48.98443422	44.61418344	49.56082625	69.83764821	51.98467184
0	0	0	0	0	0
43.08547097	103.8470005	918.4368107	320.5636421	87.94370516	296.1758277
27.12788913	29.39066053	32.30682249	35.85251261	37.50540367	24.62431824
998.9446232	714.1930509	681.5201125	551.4960028	799.2530851	902.2076599
94.14973285	20.57346237	317.6837545	48.50634059	137.0887169	45.82859228
0	0.979688684	0	0	0	0.68400884
11.17030729	14.69533026	49.99865385	36.90699827	11.63960804	15.73220332
0	0	0	0	0	0
183.5121912	190.0596048	276.9156213	218.2785326	344.0150819	233.2470144
3.191516368	35.26879264	30.76840237	13.70831364	14.2261876	29.41238012
142.0224784	100.9079345	104.5894918	115.9934231	197.8733366	80.02903428
177.1291584	68.5782079	81.53626628	68.54156822	100.876603	92.34119339
39.8939546	9.796886843	24.6147219	24.25317029	23.27921607	15.73220332
0	0.979688684	1.923025148	0	0	0.608767868
47.87274552	19.59377369	66.92127515	21.0897133	14.2261876	28.04436244
11.17030729	28.41097185	57.69075444	26.36214162	19.39934673	25.99233592
33.51092186	4.898443422	56.15233432	31.63456995	24.57250585	29.41238012
20.74485639	4.898443422	36.15287278	6.32691399	10.34631825	23.9403094
79.58046064	74.48573067	154.5496851	95.3782284	86.10723367	100.6245404
1.595758184	0	1.538420118	1.054485665	0	7.52409724
0	2.939066053	0	0	0	6.8400884
38.29819642	34.28910395	38.46050296	132.8651938	49.14501171	56.77273372
46.27698734	61.72038711	65.38285503	32.68905561	58.19804018	41.72453924
3.191516368	11.75626421	16.9226213	13.70831364	6.466448909	6.15607956
225.0019039	310.5417192	241.5319586	279.9026749	230.2831785	121.712533
0	0	5.384470415	0	0	0
11.17030729	45.06567948	58.4599645	53.77876891	51.73159127	75.92498124

0	0.979688684	5.384470415	0	0	0
14.36182366	23.51252842	1.538420118	6.32691399	6.466448909	1.36801768
20.74485639	31.3500379	86.92073669	36.90699827	51.73159127	64.9808398
19.14909821	159.6892555	173.6414788	22.14419896	73.71751756	124.5238093
3.191516368	40.16723606	114.6122988	4.21794266	40.09198323	159.3740597
0	0	0	2.10897133	0	1.36801768
122.8733802	158.7095669	278.4540414	67.48708256	144.8484556	268.8154741
3.191516368	4.898443422	2.307630178	3.163456995	7.75973869	0.68400884
1064.370709	737.7055793	795.3632012	596.8388864	778.5604486	718.8932908
114.8945892	254.7190579	146.9191213	169.7721921	338.8419228	262.6593945
33.51092186	55.84225501	73.84416569	64.32362556	69.83764821	85.501105
0	1.959377369	1.538420118	2.10897133	1.293289782	2.73603536
14.36182366	9.796886843	33.84524261	15.81728497	18.10605694	29.41238012
3.191516368	0	3.076840237	1.054485665	1.293289782	6.128719206
663.8354045	104.8266892	201.5330355	119.1568801	78.89067669	109.4414144
49.4685037	77.39540606	80.76705622	103.3395952	75.01080734	112.8614586
1.595758184	0.979688684	12.30736095	41.12494093	2.586579563	11.62815028
156.384302	146.9533026	278.4540414	175.0446204	179.7672797	274.2875448
20.74485639	47.02505685	99.22809764	27.41662729	24.57250585	46.51260112
4.787274552	5.878132106	19.99946154	3.163456995	1.293289782	18.46823868
0	0	0	0	0	0
76.59639283	605.4280131	84.61310651	10.54485665	193.9805344	23.9403094
1361.181731	2749.006448	2355.321201	2485.422712	3449.203848	2404.291072
0	0	0	0	0	2.73603536
0	0	4.615260355	1.054485665	0	3.4200442
0	0.979688684	0	1.054485665	0	0
411.7056115	1134.479496	1646.109527	932.1653278	865.210864	2284.589525
36.70243823	11.75626421	19.99946154	25.30765596	7.75973869	22.57229172
25.53213094	151.8517461	315.3761243	31.63456995	53.02488105	172.3702277
31.91516368	24.49221711	49.99865385	36.90699827	20.69263651	31.46440664
120.0488882	121.2854591	107.1125007	172.9461939	73.66578597	128.8399051
51.06426189	11.75626421	45.38339349	24.25317029	20.69263651	22.57229172
877.6670012	1876.10383	1313.810781	2417.93563	2272.310147	1162.815028
19.14909821	1.959377369	24.6147219	1.054485665	3.879869345	23.25630056
274.4704076	188.1002274	89.99757693	185.589477	187.5270184	116.2815028
57.44729462	145.973614	213.8403965	44.28839793	71.130938	246.2431824
1364.373247	1215.793657	1662.262938	923.7294425	1934.761513	2450.119665

0	0	1.538420118	0	0	0
453.1953243	26.45159448	146.1499113	343.7623268	517.3159127	47.19660996
0	2.939066053	4.615260355	2.10897133	1.293289782	5.47207072
3.191516368	0	3.076840237	1.054485665	3.879869345	2.73603536
199.469773	83.27353817	618.4448876	160.2818211	164.2478023	180.5783338
153.1927857	289.9878506	346.9137367	292.0925292	210.8062344	320.8001459
641.49479	848.4104006	963.8202042	666.4349403	1103.176184	1075.261896
130.8521711	166.5470763	136.9193905	130.7562225	126.7423986	175.106263
1.595758184	0	0	3.163456995	5.173159127	0
12.76606547	25.47190579	35.38366272	26.36214162	16.81276716	15.04819448
180.3206748	70.53758527	113.0738787	99.12165251	56.9047504	97.81326411
196.2782566	207.6940011	276.1464113	229.877875	307.8029681	185.3663956
0	0	0	0	0	0
330.3219441	202.7955577	82.30547634	486.1178915	237.9653198	51.98467184
73.40487646	93.07042501	119.9967692	113.8844518	77.5973869	131.3296973
102.3519299	170.8087221	259.5083977	148.7773825	151.9744823	135.3995499
0	0	0	0	0	0
0	0	0	1.054485665	1.293289782	0
0	0.979688684	0	0	0	0.595087691
73.40487646	27.43128316	38.46050296	42.1794266	29.74566498	47.19660996
122.8733802	156.7501895	303.0687633	173.9901347	143.5551658	274.9715537
28.72364731	132.2579724	69.22890533	12.65382798	9.053028472	120.3855558
469.1529061	402.6520493	503.8325888	600.0023434	857.4511253	359.7886498
0	0	0	0	0	0.68400884
623.9414499	586.8335219	379.9897693	1077.68435	632.4187033	740.0975648
1507.991484	916.9886085	4872.176515	551.4960028	756.5745223	2679.946635
272.8746495	450.6567948	794.5939912	737.0854798	738.4684654	857.0630765
453.1953243	313.500379	306.9148136	534.6242321	241.8451892	365.9447294
0	0	0	0	0	0
1147.350134	402.6520493	636.136719	586.2940297	969.9673363	658.016504
28.72364731	26.45159448	40.76813314	55.88774024	23.27921607	26.67634476
183.5121912	49.9641229	156.149642	50.61531192	54.31817083	76.60899008
2806.938646	2566.784353	2532.239515	2199.657097	2117.115373	1619.732933
25.53213094	23.51252842	66.15206509	22.14419896	42.6785628	22.57229172
11.17030729	76.41571738	50.76786391	23.19868463	25.86579563	120.3855558
102.1285238	58.78132106	96.1512574	68.54156822	124.155819	119.0175382
44.68122915	35.26879264	26.92235207	24.25317029	37.50540367	9.576123759

457.9825988	305.6628695	726.1342959	673.8163399	632.4187033	565.6753106
6.383032736	5.878132106	35.38366272	18.98074197	20.69263651	13.6801768
11.17030729	18.614085	43.84497338	11.59934231	18.10605694	16.41621216
11.17030729	53.88287764	40.76813314	52.72428325	19.39934673	32.83242432
9.574549104	51.92350027	33.84524261	21.0897133	19.39934673	12.31215912
0	0	0	0	0	0.68400884
2551.617336	1986.808652	9321.287498	8604.603026	15641.04662	5825.019281
3930.352407	3628.766887	5303.703358	3030.591801	1171.720542	1753.114657
773.3682463	267.5921672	443.7880516	437.8224481	301.9702311	284.6844792
116.4903474	35.26879264	153.0728018	31.63456995	31.03895476	86.86912268
25.53213094	58.78132106	58.4599645	25.30765596	21.98592629	52.66868068
3.191516368	0	1.538420118	0	0	0.68400884
46.27698734	21.55315106	52.30628403	20.03522763	36.21211389	19.83625636
955.9389401	550.1245869	889.6683545	967.9334816	632.573898	789.9344489
0	0	1.538420118	0	1.293289782	2.05202652
6.383032736	18.614085	46.92181361	59.05119724	36.21211389	39.67251272
15.95758184	29.81192666	39.22971302	42.27433031	40.09198323	51.300663
173.2035933	244.5498894	494.594376	275.4422005	239.1680793	260.7852103
362.2371078	177.3236519	253.8393195	265.7303876	140.9685862	183.3143691
55.85153644	41.14692474	235.3782781	120.2113658	161.6612227	258.5553415
38.29819642	69.55789659	57.69075444	82.24988187	43.97185258	81.39705196
0	0	0	0	0	0
47.10678159	59.76100974	103.6510555	82.11279873	42.41990484	47.30605137
15.95758184	6.85782079	9.230520711	8.43588532	5.173159127	17.78422984
0	0.979688684	4.615260355	0	0	4.78806188
518.1745975	709.2946075	618.4448876	980.6716684	923.4089042	558.0280918
6507.501874	3144.800677	3682.208554	2870.30998	4002.731875	3894.062326
38.29819642	33.30941527	106.1509882	36.90699827	49.14501171	41.72453924
6.383032736	25.47190579	19.99946154	29.52559862	15.51947738	31.46440664
3633.541385	2172.949502	1885.333855	2179.621869	1751.114364	1584.164473
22.34061458	31.3500379	79.2286361	76.97745354	32.33224454	64.29683096
0	1.959377369	12.30736095	1.054485665	0	21.20427404
234.576453	177.3236519	204.6098758	410.1949237	328.4956046	233.9310233
1.595758184	0	4.615260355	12.65382798	6.466448909	5.47207072
28.72364731	15.67501895	27.69156213	21.0897133	11.63960804	23.9403094
0	0	0.769210059	0	0	0.68400884
31.91516368	43.10630211	305.3763935	82.24988187	60.78461974	95.07722875

344.6837677	64.65945317	94.61283728	327.9450418	269.0042746	134.7497415
607.9838681	669.1273714	623.829358	670.6528829	506.9695944	485.6462764
9.574549104	17.63439632	54.6139142	21.0897133	73.71751756	47.19660996
38.29819642	30.37034921	16.9226213	160.2818211	78.89067669	33.51643316
15.95758184	33.30941527	27.69156213	15.81728497	137.0887169	102.601326
28.72364731	13.71564158	51.53707397	33.74354128	16.81276716	33.51643316
114.8945892	126.3798403	166.1493728	149.7369644	131.9155577	101.2333083
12.76606547	1.959377369	9.230520711	73.81399655	27.15908542	29.41238012
105.3200401	272.3534542	313.8377042	237.2592746	366.0010082	364.5767117
95.74549104	147.9329913	168.457003	76.97745354	86.65041538	283.8636686
544.1535407	461.4333703	584.599645	401.7590384	761.7476814	577.9874698
17.55334002	7.837509475	8.461310651	4.21794266	2.586579563	1.36801768
47.87274552	41.14692474	103.0741479	69.59605389	137.0887169	49.93264532
328.7261859	443.798974	362.2979379	527.2428325	338.8419228	700.4250521
12.76606547	3.918754737	9.230520711	12.65382798	5.173159127	12.99616796
1645.226688	455.5552382	485.3715474	939.5467275	708.7228004	254.4512885
19.14909821	13.71564158	21.53788166	7.381399655	18.10605694	19.15224752
0	0	1.538420118	0	0	1.36801768
695.7505682	135.1970384	248.4548491	264.6759019	159.0746432	433.6616045
437.2377424	610.3460503	906.8986598	562.0408594	508.2628842	621.7640355
4922.913998	7407.426142	3082.993917	5034.114565	12381.95637	3080.775815
20.74485639	4.898443422	89.99757693	10.54485665	24.57250585	36.25246852
110.1073147	48.98443422	61.53680474	78.03193921	65.95777887	64.29683096
896.8160994	4224.417607	1708.415542	1418.283219	5420.177475	1090.310091
1354.798698	1169.748289	899.2065592	1499.478616	2666.76353	1097.150179
860.1136612	3756.126416	2816.078027	2952.559862	3508.695178	2006.881936
23.93637276	51.92350027	37.6912929	42.1794266	27.15908542	21.88828288
0	0	0	0	0	0
574.4729462	355.6269924	597.676216	802.463591	303.9230987	393.9890918
20.74485639	12.7359529	22.30709172	61.16016857	18.10605694	21.20427404
6.383032736	13.71564158	17.69183136	4.21794266	5.173159127	15.73220332
371.8116569	584.8741445	589.2149054	907.9121575	1141.974877	919.3078809
51.06426189	66.61883053	147.6883314	152.9004214	103.4631825	148.4299183
87.76670012	0	0.769210059	0	0	7.52409724
0	0	0	0	0	0
472.3444225	466.3318137	356.9134675	693.8515675	284.523752	273.603536
223.4061458	1258.899959	85.38231657	3036.918715	516.0226229	114.9134851

8551.668108	4115.672163	6565.207855	11304.08633	15731.57691	4487.09799
300.0025386	546.6662859	358.4518876	435.5025796	512.1427536	528.7388333
25.53213094	23.51252842	76.15179586	94.90370985	65.95777887	64.9808398
0	8.817198159	2.307630178	1.054485665	11.63960804	5.47207072
76.59639283	96.00949106	83.84389646	89.63128152	82.77054603	95.76123759
6.383032736	0.979688684	0.769210059	5.272428325	5.173159127	5.47207072
12.76606547	10.77657553	13.84578107	6.32691399	6.466448909	12.31215912
118.0861056	86.21260422	87.68994675	22.14419896	85.3571256	98.49727295
205.8528057	98.94855712	76.92100592	186.6439627	182.3538592	123.8056
215.4273548	220.429954	306.9148136	352.1982121	130.622268	305.0679426
132.4479293	323.2972658	223.8401272	314.2367282	166.8343818	325.5882078
315.9601204	382.0785869	196.1485651	497.7172339	644.0583113	432.9775957
80.96877026	274.881051	150.6651743	193.9199138	188.8203081	110.6931506
1613.311524	451.6364835	719.2114054	741.3034225	545.7682879	864.5871737
1468.097529	4670.175958	1289.965269	3777.167652	1989.079684	1280.464548
9.574549104	3.918754737	9.99973077	2.10897133	6.466448909	4.78806188
9.574549104	28.41097185	35.38366272	22.14419896	10.34631825	27.3603536
157.9800602	229.2471521	65.38285503	74.86848221	187.5270184	15.73220332
1.595758184	1.959377369	0	3.163456995	1.293289782	1.36801768
35.10668005	17.63439632	44.61418344	24.25317029	21.98592629	11.62815028
7.97879092	1.959377369	7.692100592	8.43588532	3.879869345	19.15224752
264.8958585	311.5410016	306.1456036	297.3649575	218.5659731	218.1988199
0	0	0	0	0	0
129.2564129	97.96886843	119.2275592	246.7496456	309.0962578	140.905821
395.7480296	481.027144	676.9048521	949.0370985	1228.625293	725.7333792
31.91516368	42.12661343	161.5341124	22.14419896	42.6785628	84.13308732
977.4976332	826.1322799	1241.305041	1989.107944	1522.90045	238.1992384
15.95758184	8.817198159	26.15314201	7.381399655	14.2261876	7.52409724
181.916433	120.5017082	123.8428195	145.5190218	133.2088475	158.006042
9.574549104	13.71564158	29.99919231	9.490370985	3.879869345	14.36418564
12.76606547	12.7359529	34.61445267	17.9262563	10.34631825	38.30449504
70.58038448	46.17272769	80.4901406	69.38515675	59.21973911	62.0874824
173.9376421	257.658124	298.453503	256.2400166	421.6124688	240.7711117
0	0	0	0	0	0
609.5796263	474.1693232	436.9113136	576.8036587	452.6514236	580.7235051
7.97879092	8.817198159	12.30736095	12.65382798	18.10605694	17.78422984
0	0	0	0	0	0

7.97879092	0	0	6.32691399	1.293289782	0.68400884
54.25577826	0	5.384470415	9.490370985	5.173159127	19.83625636
1030.859787	821.9588062	1025.357009	1018.633152	994.5398422	751.7257151
762.7245392	244.6870458	359.6826237	319.4880668	110.6280079	230.4767786
164.3630929	32.32972658	163.0725326	763.4476214	23.27921607	132.697715
1.595758184	12.7359529	6.153680474	8.43588532	9.053028472	1.36801768
6575.975858	3512.183933	1282.380858	2908.271464	3551.580667	1909.752681
150.0012693	237.0846616	161.5341124	126.5382798	302.6298089	179.2103161
116.4903474	265.4956335	180.7643639	421.794266	584.5669813	180.5783338
116.4903474	83.27353817	106.9201982	187.6984484	103.4631825	96.44524643
11.17030729	0.979688684	6.922890533	0	10.34631825	1.36801768
6.383032736	30.37034921	30.76840237	20.03522763	23.27921607	31.46440664
0	1.959377369	1.538420118	1.054485665	36.21211389	6.15607956
54.25577826	82.29384948	142.303861	82.24988187	107.3430519	51.98467184
41.48971278	65.63914185	44.61418344	158.1728497	197.8733366	55.40471604
3.191516368	11.75626421	8.461310651	5.272428325	3.879869345	10.2601326
3.191516368	18.614085	16.15341124	11.59934231	7.75973869	6.8400884
11.17030729	8.817198159	12.30736095	26.36214162	18.10605694	38.98850388
15.95758184	3.918754737	2.307630178	2.10897133	3.879869345	1.36801768
35.10668005	3.918754737	5.384470415	14.76279931	23.27921607	10.2601326
65.42608554	0.979688684	0.769210059	10.54485665	10.34631825	1.36801768
52.66002007	48.98443422	116.919929	31.63456995	46.55843214	99.18128179
47.87274552	23.51252842	33.07603255	29.52559862	29.74566498	19.15224752
319.1516368	417.3473795	416.9118521	417.5763233	386.6936447	575.9354432
76.59639283	103.8470005	337.683216	51.66979758	142.261876	203.1506255
1745.759453	784.7306361	974.589145	531.4607751	2858.170418	1158.710975
127.6606547	86.21260422	49.99865385	75.92296788	53.02488105	41.0405304
3438.858886	2671.611042	2653.774704	2077.33676	2899.555691	1656.66941
14.36182366	22.53283974	36.92208284	45.34288359	45.26514236	57.45674256
392.5565133	115.6032648	177.6875237	127.5927655	86.65041538	71.8209282
884.0500339	709.2946075	702.2887841	617.9285997	437.1319462	573.1994079
62.23456918	53.88287764	56.15233432	54.83325458	25.86579563	38.30449504
49.4685037	15.67501895	84.61310651	23.19868463	117.6893701	23.25630056
111.7030729	69.55789659	173.8414734	92.79473852	43.97185258	133.3817238
38.29819642	31.3500379	69.99811539	44.28839793	36.21211389	32.14841548
0	2.939066053	2.307630178	0	0	0
138.830962	280.1909637	146.9191213	368.0154971	153.901484	221.6188641

818.6239484	693.6195885	583.830435	739.1944511	1310.102549	445.9737637
6.383032736	20.57346237	15.38420118	5.272428325	9.053028472	29.41238012
243.1935472	389.6221898	494.3328446	214.1765834	307.1304574	467.1780377
1.595758184	0	0.769210059	5.272428325	3.879869345	0.68400884
39.8939546	75.43602869	61.53680474	78.03193921	25.86579563	67.71687516
118.0861056	367.3832566	289.2229823	42.1794266	186.2337286	281.8116421
59.04305281	49.9641229	92.30520711	84.3588532	50.43830149	45.14458344
0.829794256	4.731896345	0.699981154	0	12.4414477	8.775833417
0	0	0	0	0	0
92.55397467	35.26879264	405.3737012	860.4603026	160.3679329	339.9523935
1.595758184	21.55315106	9.99973077	2.10897133	11.63960804	15.04819448
49.4685037	151.8517461	162.3033225	97.01268118	99.58331319	220.2508465
496.2807952	404.6114266	548.4467722	305.8008428	292.2834907	419.9814277
550.5365735	47.02505685	11.53815089	39.0159696	73.71751756	145.6391622
582.4517372	990.4456661	611.5219971	533.5697465	950.5291909	792.0822367
113.2988311	308.6019356	181.533574	565.2043164	358.2412695	243.507147
7.97879092	5.878132106	9.99973077	17.9262563	10.34631825	12.31215912
0	0	0	0	0	0
76.59639283	24.49221711	72.30574557	28.47111295	25.86579563	34.200442
0	0	0.769210059	0	0	0
0	0	0	0	0	0
239.3637276	2954.741072	966.8970444	817.2263903	985.4868137	2388.558869
1.595758184	8.817198159	23.84551184	37.96148394	24.57250585	25.30832708
57.44729462	53.88287764	43.07576332	138.1376221	51.73159127	45.14458344
0	0	0	0	0	0.68400884
0	0	0	0	0	0.68400884
51.06426189	60.74069843	3.846050296	70.65053955	19.39934673	15.04819448
28.72364731	15.67501895	6.153680474	7.381399655	10.34631825	37.6204862
665.4311627	640.7163996	362.2979379	1126.19069	844.5182275	662.8045659
20.74485639	17.63439632	39.22971302	21.0897133	12.93289782	21.88828288
148.4055111	50.94381159	139.2270207	81.1953962	63.37119931	51.300663
1388.30962	1262.818714	1247.658716	648.508684	825.1188808	1055.42564
0	0	0	0	0	0.68400884
5044.19162	3402.458801	5519.851385	3635.866573	5127.893985	8376.372254
122.8733802	257.658124	264.6082604	461.8647213	412.5594404	1028.749295
25.53213094	12.7359529	19.23025148	8.43588532	6.466448909	21.88828288
517.0256516	100.9079345	255.3777397	22.14419896	58.19804018	305.7519515

14.36182366	5.878132106	3.076840237	2.10897133	11.63960804	4.78806188
1.595758184	9.796886843	28.46077219	4.21794266	2.586579563	34.200442
1153.733167	928.7448727	937.6670622	737.0854798	1108.349343	751.7257151
33.51092186	182.2220953	62.3060148	265.7303876	483.6903784	133.3817238
95.74549104	71.51727396	44.61418344	99.12165251	56.9047504	106.705379
0	0	0	0	0	0
165.9588511	159.6892555	208.4559261	230.9323606	314.269417	437.7656576
497.8765534	363.4645019	409.2197515	833.0436753	616.8992259	309.1719957
0	0	0	0	0	0.68400884
394.1522714	821.9588062	599.9838462	1101.93752	920.8223246	1013.701101
17.55334002	21.55315106	46.92181361	44.28839793	29.74566498	36.93647736
130.8521711	381.0988982	492.2944379	414.4128663	303.9230987	186.0504045
303.194055	297.82536	163.8417426	390.159696	225.032422	402.1971979
28.72364731	66.61883053	33.84524261	119.1568801	27.15908542	32.83242432
183.5121912	90.13135896	79.2286361	92.79473852	125.4491088	110.8094321
51.06426189	24.49221711	81.53626628	56.94222591	38.79869345	25.30832708
84.57518375	36.24848132	78.45942604	43.23391226	64.66448909	56.77273372
722.8784573	114.6235761	314.6069142	324.7815848	162.9545125	183.3143691
36.70243823	113.6438874	127.6888698	52.72428325	51.73159127	80.71304312
17.55334002	7.837509475	19.23025148	6.32691399	15.51947738	7.52409724
105.3200401	358.5660585	789.2095208	172.9356491	324.6157352	270.1834918
3110.132701	384.0379643	1105.354855	210.897133	1298.462941	2846.160783
103.724282	23.51252842	75.3825858	55.88774024	28.4523752	40.35652156
446.8122915	287.0487845	283.8385119	131.8107081	230.2055812	106.705379
0	1.959377369	1.538420118	2.10897133	0	5.47207072
861.7094193	460.4536816	841.5158048	1149.389375	1791.206348	398.7771537
2704.810122	2390.44039	2851.46169	2085.772645	3034.057828	1727.80633
1275.010789	1113.906034	730.7495563	853.078903	2179.193282	758.5658035
0	0	0	0	1.293289782	0
1487.246627	941.4808256	1927.640408	5200.7233	2418.451892	958.2963848
164.3630929	88.17198159	205.3790858	129.7017368	124.155819	135.4337503
320.747395	434.0020872	442.2957841	477.6820062	409.9728608	367.9967559
116.4903474	42.12661343	46.92181361	94.90370985	65.95777887	63.61282212
0	0	0.769210059	0	0	0
150.0012693	124.4204629	201.4714987	93.75432047	95.70344385	102.6834071
2237.252974	2191.563587	2282.246246	2845.002324	2928.008066	2282.537499
191.4909821	25.47190579	406.9121213	307.9098142	159.0746432	114.2294763

27.12788913	52.90318895	45.38339349	80.14091054	24.57250585	61.5607956
935.1142958	311.5410016	450.7570947	52.72428325	99.58331319	212.7267492
148.4055111	65.63914185	70.76732545	67.48708256	190.1135979	110.1254232
333.5134605	283.1300298	373.0668787	558.8774024	378.9339061	337.9003669
210.6400803	115.6032648	45.38339349	295.2559862	196.5800468	326.2722167
1251.074416	1033.571562	661.5206509	682.2522252	1198.879628	941.8801726
28.72364731	6.85782079	11.53815089	33.74354128	12.93289782	10.2601326
0	0	0	0	0	0
175.5334002	49.9641229	285.376932	69.59605389	68.54435843	45.14458344
0	1.067860666	0	0	0	0
0	0	0	3.163456995	0	0
381.386206	250.8003032	74.61337574	68.54156822	212.0995242	770.1939538
11744.78023	10284.77181	12389.66642	8722.705421	5210.664531	9357.240931
15.95758184	56.82194369	349.9905769	6.32691399	20.69263651	58.82476024
734.0487646	1106.068525	938.4362723	1298.071854	1563.587346	1625.205004
38.29819642	48.00474553	40.76813314	57.99671157	75.01080734	72.50493704
19.14909821	72.49696264	277.6848314	89.63128152	50.43830149	112.8614586
5190.522645	1752.731634	3279.796311	2949.238232	8424.075786	859.0809026
15.95758184	39.18754737	22.30709172	57.99671157	50.43830149	95.76123759
0	0	0.769210059	0	0	0.68400884
185.1079493	1301.026573	730.7495563	702.2874529	825.1188808	922.0439163
470.7486643	382.0785869	336.9140059	686.4701679	474.6373499	629.9721416
0	12.7359529	11.53815089	25.30765596	12.93289782	20.5202652
127.6606547	126.3798403	104.6125681	127.5927655	147.4350351	143.6418564
9.574549104	36.24848132	33.10680095	18.98074197	32.33224454	36.25246852
753.7882934	993.913764	799.7553907	807.6727502	870.7978758	595.1902921
6786.759556	5486.256632	5398.316196	3558.889119	3310.821841	5025.412947
154.7885438	108.745444	171.5338432	139.1921078	157.7813534	175.7902719
35.10668005	35.26879264	24.6147219	56.94222591	28.4523752	56.08872488
43.08547097	24.49221711	71.53653551	21.0897133	42.6785628	56.77273372
0	0	0	0	1.293289782	0
43.08547097	14.69533026	15.38420118	15.81728497	19.39934673	25.30832708
54.25577826	65.63914185	85.38231657	85.41333886	131.9155577	39.67251272
304.7898131	357.5863698	456.9107752	647.4541983	439.7185258	728.4694146
509.0468607	187.1205387	144.6114911	315.2912138	143.5551658	223.6708907
7.97879092	46.04536816	25.38393195	9.490370985	36.21211389	113.5454674
0	0	3.846050296	1.054485665	1.293289782	8.89211492

148.4055111	32.32972658	30.76840237	43.23391226	42.6785628	34.200442
10629.34526	16288.30407	15008.05747	6364.875474	9702.259943	11388.06318
40.27693656	120.3645518	72.7595795	186.5806936	106.6834741	128.0669751
146.8097529	180.2627179	160.7649024	412.303895	351.7748206	365.9447294
9.574549104	18.36916283	9.99973077	7.666110784	3.879869345	8.20810608
317.5558786	101.8876232	184.6104142	103.3395952	129.3289782	225.0389083
1.595758184	0	4.615260355	20.03522763	3.879869345	18.46823868
287.3322186	58.17391408	68.19816385	68.83682421	53.89138521	38.59861884
451.5995661	1495.984621	927.6673314	1663.978379	2492.169409	1428.210458
1.595758184	2.939066053	2.307630178	8.43588532	3.879869345	4.10405304
14.36182366	53.88287764	19.23025148	39.0159696	50.43830149	30.7803978
263.3001004	1619.425395	662.289861	618.9830853	386.6936447	595.7716996
86.17094193	140.0954819	139.2270207	194.0253624	148.7283249	263.3434034
509.0468607	326.2363319	436.1421036	466.0826639	459.1178725	321.4841548
234.576453	1108.027902	408.4505414	974.3447544	538.0085492	411.0893128
36.70243823	15.67501895	79.2286361	61.16016857	36.21211389	39.67251272
3853.756014	6390.509288	2930.690326	5479.107515	5436.990242	3339.331157
30.3194055	6.85782079	37.6912929	15.81728497	20.69263651	15.04819448
43.08547097	22.53283974	17.69183136	26.36214162	28.4523752	16.41621216
0	0	0	0	0	0
0	0.979688684	0	0	0	0
22.34061458	407.5504927	293.0690326	55.88774024	64.66448909	666.2246101
2720.767704	958.1355333	1881.487805	880.4955302	1470.470482	1518.499625
694.15481	462.413059	476.9102367	312.1277568	377.6406163	372.1008089
1150.541651	859.1869762	1350.732864	1174.697031	879.4370516	737.3615295
71.80911828	183.201784	143.073071	69.59605389	140.9685862	208.6226962
31.91516368	20.57346237	51.53707397	25.30765596	18.10605694	21.20427404
426.0674351	120.5017082	154.6112219	56.94222591	103.4631825	131.3296973
55.85153644	100.9079345	53.84470415	63.2691399	78.89067669	98.49727295
28.72364731	11.75626421	16.9226213	31.63456995	11.63960804	23.9403094
78.19215101	166.5470763	23.07630178	43.23391226	170.7142512	98.49727295
181.916433	332.114464	197.6869852	56.94222591	287.1103315	480.7898136
14.36182366	7.837509475	9.230520711	4.21794266	7.75973869	12.31215912
3020.770242	7243.818132	6493.67132	3219.344735	8542.179008	7097.959732
571.2814299	1054.145024	507.6786391	1555.366356	481.1037988	2789.388049
134.0436875	209.6533784	135.3809704	373.2879254	157.7813534	151.8499625
148.4055111	94.0501137	259.993	129.7017368	137.0887169	195.6265282

135.6394456	188.1002274	88.45915681	117.0479088	151.3149045	212.7267492
19.14909821	1.959377369	23.84551184	9.490370985	9.053028472	6.15607956
0	0	0.769210059	0	0	0
483.5147297	13012.22511	0	5734.293046	5240.410196	173.7382454
392.5565133	330.1550866	436.0267221	305.6743046	258.580359	355.6845968
42.5269556	5.133568706	101.3741937	0	0	0
146.8097529	115.6032648	149.2267515	155.0093927	155.1947738	104.6533525
2020.229861	922.8667406	4119.889077	2281.906979	1270.010566	3304.446706
9.574549104	9.796886843	27.69156213	14.76279931	9.053028472	8.89211492
240.9594858	268.4346995	242.3011687	396.48661	340.1352126	232.5630056
35.10668005	58.78132106	72.30574557	105.3642076	108.5716772	59.50876908
30.3194055	48.98443422	68.45969527	57.99671157	41.38527302	49.24863648
0	0	0	1.054485665	0	0
266.4916167	299.7847374	611.5219971	565.2043164	605.2596179	590.2996289
172.3418839	45.06567948	82.30547634	87.52231019	77.5973869	104.6533525
157.9800602	317.4191337	316.9145444	183.4805057	280.6438826	418.6134101
46.27698734	91.11104764	64.61364497	33.74354128	102.1698928	97.81326411
78.19215101	234.1455956	244.6087988	44.28839793	133.2088475	444.605746
15.95758184	5.878132106	39.99892308	11.59934231	19.39934673	16.41621216
406.9183369	402.6520493	571.523074	447.1019219	597.4998792	564.9913018
681.3887446	564.3006822	1128.431157	293.1470149	647.9381807	583.4595405
579.2602208	1002.221524	2048.406388	1387.703135	2185.659731	1181.283267
0	0	0	0	0	0
0	0	0.769210059	2.10897133	0	1.36801768
376.5989314	171.4455198	283.0693018	160.2818211	329.7888943	340.6364023
9.574549104	70.53758527	353.8366272	117.0479088	94.41015407	145.0098741
0	0	0	0	0	0
1069.157983	6496.315666	7802.866841	6264.699336	3101.308897	4032.232112
82.97942557	47.02505685	109.2278284	83.30436753	45.26514236	72.50493704
143.6182366	217.4908879	212.3019763	172.9356491	320.7358659	181.9463514
199.469773	168.5064537	173.8414734	187.6984484	289.6969111	176.4742807
0	0	0	0	0	0
0	4.898443422	9.230520711	0	6.466448909	13.6801768
349.4710423	216.5111992	189.9948846	277.3297299	204.3397855	212.0427404
277.661924	486.9052761	574.5999142	693.8515675	1094.123155	579.3554874
2029.80441	255.6987466	985.3580859	990.1620394	1174.307122	453.4978609
0	0	0	0	0	1.36801768

2680.873749	3203.581998	858.4384261	2713.191616	3978.159369	1059.529693
647.8778227	1630.201971	85.38231657	169.7721921	3423.338052	51.98467184
687.7717773	1805.566245	1195.352432	550.4415171	1356.660981	1937.113035
70.2133601	21.55315106	37.6912929	30.58008428	34.91882411	44.4605746
75.00063465	371.3020114	287.6845622	540.9511461	226.3257118	504.7985239
180.3206748	125.4001516	235.3782781	133.9196795	212.0995242	243.507147
534.5789916	724.9696264	1025.357009	584.1850584	619.4858055	1120.40648
3.191516368	1.959377369	19.23025148	18.98074197	9.053028472	16.41621216
4.787274552	59.76100974	47.69102367	13.70831364	16.81276716	127.2256442
103.724282	92.09073633	176.1491036	63.2691399	138.3820066	88.23714036
78.19215101	5.878132106	95.38204734	37.96148394	20.69263651	35.56845968
87.76670012	22.53283974	490.7560178	156.0638784	258.6579563	822.1786256
146.9054984	49.2979346	54.46007219	54.86488915	51.73159127	86.25351472
194.6824984	958.1355333	516.1399497	733.9220228	223.7391322	333.1123051
119.6818638	158.7095669	124.6120296	121.2658515	115.1027906	81.39705196
111.7030729	90.13135896	61.53680474	32.68905561	188.8203081	89.60515804
6.383032736	17.63439632	6.922890533	10.54485665	11.63960804	4.78806188
426.0674351	392.8551624	207.686716	194.0253624	252.1915074	221.6188641
41.48971278	64.65945317	19.99946154	59.05119724	28.4523752	34.200442
0	0	0	0	0	2.73603536
0	0	0	0	0	0.68400884
100.5327656	191.0392934	293.8382426	218.2785326	329.7888943	259.2393503
51.06426189	122.4610855	130.7657101	104.3940808	117.6893701	266.0794387
146.8097529	468.2911911	130.7657101	136.0286508	267.7109848	235.9830498
853.7306284	1005.16059	889.9760385	1223.203371	968.6740465	537.6309482
965.4337013	401.6723606	163.8417426	500.8806909	297.4566498	487.0142941
151.5970275	172.4252084	190.7640947	188.752934	162.9545125	175.106263
126.0648965	171.4455198	155.380432	127.5927655	265.1244053	188.7864398
430.8547097	287.0487845	422.2963225	380.6693251	603.9663281	256.503315
75.75064099	105.1597834	97.93582474	104.1199146	36.93635617	87.22480727
245.7467603	342.8910395	260.1776104	657.9990549	607.8461974	458.2859228
0	0	0.769210059	0	0	0
577.6644626	731.8274472	1017.664908	654.8355979	698.3764821	848.1709616
7.97879092	0	0.769210059	2.10897133	6.466448909	15.73220332
31.91516368	130.298595	172.3030533	132.8651938	113.8095008	140.905821
418.0886442	290.9675392	476.9102367	280.4931869	248.3116381	280.4436244
718.0911828	519.2350027	339.9908462	877.3320732	751.4013632	207.2546785

11.17030729	67.59851922	184.6104142	90.68576719	71.130938	252.3992619
65.42608554	117.5626421	69.99811539	159.2273354	126.7423986	157.3220332
84.57518375	145.973614	566.1386036	163.4452781	208.2196549	510.2705946
0	0	0.469218136	0	0	2.072546785
105.3200401	140.0954819	201.5330355	162.3907924	111.2229212	147.7459094
0	0	0	0	0	0
1675.546093	466.3318137	372.2976687	1072.411921	1548.067869	620.3960178
0	4.898443422	6.153680474	3.163456995	0	4.10405304
472.3444225	535.8897103	296.9150829	216.1695613	403.5064119	363.8927029
97.34124922	338.9722848	4.615260355	606.3292574	43.97185258	2.05202652
312.7686041	224.3487087	141.5346509	266.7848732	298.7499396	149.797936
0	0	0	0	0	0
30.3194055	30.37034921	39.22971302	26.36214162	24.57250585	42.40854808
316.3271448	188.7860095	258.616114	203.2099325	239.1422135	132.8276766
1630.864864	228.2674634	1280.734749	1457.299189	729.4154369	895.3675715
51.06426189	30.37034921	56.15233432	30.58008428	53.02488105	16.41621216
146.8097529	145.973614	31.53761243	799.300134	267.7109848	64.9808398
138.830962	365.4238793	451.5263048	433.3936083	525.0756514	618.3439913
0	2.939066053	1.538420118	1.054485665	0	0
943.0930867	414.4083135	883.053148	370.1244684	863.9175742	332.4282962
0	0	0.769210059	0	0	0
126.0648965	146.5026459	208.1559341	123.069022	133.402841	195.7701701
0	0	31.53761243	36.90699827	7.75973869	22.57229172
38.29819642	54.86256632	54.6139142	59.05119724	64.66448909	76.60899008
15.95758184	8.817198159	16.9226213	16.87177064	10.34631825	19.15224752
63.83032736	19.59377369	35.38366272	107.5575378	37.50540367	101.9173172
3205.878192	3388.743159	4079.120944	2686.829474	2975.859788	2699.098882
12.76606547	15.67501895	36.15287278	46.39736926	32.33224454	62.24480444
228.1934203	402.6520493	152.3035917	351.1437264	327.2023148	125.8576266
11.17030729	10.77657553	29.22998225	5.272428325	10.34631825	15.73220332
0	9.796886843	10.76894083	4.21794266	3.879869345	12.31215912
448.4080497	399.7129832	456.9107752	266.7848732	472.0507703	667.5926278
9.574549104	17.63439632	34.61445267	29.52559862	16.81276716	35.56845968
44.68122915	313.500379	47.69102367	108.6120235	71.130938	68.400884
194.6824984	41.14692474	52.30628403	50.61531192	55.61146062	60.19277792
206.3634484	205.0096541	193.925548	129.8388199	224.0624547	232.2210012
946.1250273	260.1955177	1812.335821	337.2666951	564.6373858	676.7651863

1075.541016	1018.876232	881.5147279	1021.796609	1063.084201	1092.362117
7.97879092	18.614085	5.384470415	4.21794266	3.879869345	5.47207072
0	2.939066053	6.153680474	4.21794266	5.173159127	9.576123759
0	0	0	1.054485665	0	0
7.97879092	5.878132106	13.07657101	7.381399655	5.173159127	12.31215912
3.191516368	22.53283974	27.69156213	7.381399655	11.63960804	21.20427404
486.1158156	544.8636587	301.9841772	394.1878313	359.6380225	163.9227185
102.1285238	85.23291554	217.6864468	88.57679586	113.8095008	110.1254232
3.191516368	4.898443422	16.15341124	7.381399655	3.879869345	11.62815028
0	0	0	0	0	0
41.48971278	67.59851922	119.9967692	233.041332	181.0605694	383.0449504
142.0224784	645.614843	875.3610474	939.5467275	681.563715	846.118935
126.0648965	59.76100974	84.61310651	47.45185492	121.5692395	123.8056
282.4491986	403.6317379	486.1407574	419.6852947	500.5031455	510.2705946
1664.886429	877.3308106	1044.794947	961.5116639	832.4776996	732.8881117
1128.201036	122.4610855	416.142642	180.3170487	439.7185258	269.4994829
0	0	0	0	0	0
83.56985609	79.58990871	181.9412553	142.0603088	65.88018148	40.9789696
114.8945892	410.4895587	258.4545799	622.1465423	530.2488105	411.0893128
2767.044691	10751.10362	2636.082873	3835.164363	10038.51529	4356.452302
0	0	0	0	0	0
307.9813295	835.6744477	620.7525178	1563.802241	818.6524318	1083.470003
98.93700741	152.8314348	137.6886006	60.1056829	73.71751756	152.5339713
14.36182366	29.39066053	4.615260355	13.70831364	15.51947738	51.98467184
0	0	8.038245119	8.46751989	1.293289782	0.68400884
36.70243823	94.0501137	178.4567337	88.57679586	82.77054603	152.5339713
12.76606547	15.67501895	17.69183136	41.12494093	27.15908542	18.46823868
23.93637276	34.28910395	33.07603255	42.15833689	48.62769579	30.04850834
129.2564129	205.7346237	253.0701095	246.7496456	306.5096783	213.4107581
0	0	0	0	1.293289782	2.73603536
7.97879092	4.898443422	29.99919231	7.381399655	14.2261876	23.9403094
180.3206748	257.658124	195.379355	274.1662729	249.6049279	209.306705
70.2133601	239.044039	147.6883314	80.14091054	243.138479	168.2661746
0	0	0	0	0	0
148.4055111	146.9533026	166.1493728	123.3748228	453.9447134	272.9195271
0	0	0	1.054485665	0	0
114.8945892	23.51252842	21.53788166	28.47111295	28.4523752	45.14458344

0	0	1.538420118	3.163456995	0	6.8400884
0	18.44753793	41.49119059	0	51.51173201	26.97730865
750.0063465	666.1883053	512.2938994	835.1526467	937.6350918	1001.388942
212.2358385	113.6438874	136.9193905	104.3940808	96.99673363	75.2409724
0	0	0.153842012	0	0	0
3.191516368	1.959377369	93.84362723	225.6599323	9.053028472	128.5936619
352.6625587	379.1395208	678.4432722	751.8482791	442.3051054	507.5345593
214.9007546	753.8704426	450.4340265	426.2758301	312.2518849	561.6738589
1.595758184	23.51252842	19.99946154	25.30765596	1.293289782	29.41238012
0	0	0	0	0	2.05202652
1.595758184	0	1.538420118	0	0	0
41.48971278	67.59851922	77.69021598	43.23391226	50.43830149	84.81709616
20.74485639	31.3500379	119.2275592	191.916391	75.01080734	180.5783338
11.17030729	0	1.538420118	3.163456995	3.879869345	8.89211492
70.2133601	49.9641229	108.4586184	99.12165251	103.4631825	117.6495205
2660.128893	815.1009854	1696.877391	642.18177	1774.393581	3048.6274
510.6426189	875.8416838	669.2127515	526.1883468	998.4197115	611.5039029
76.59639283	665.2086167	376.143719	345.8712981	324.6157352	1768.84686
75.00063465	231.2065295	332.2987456	301.5829002	212.0995242	457.6019139
209.0443221	310.5613129	618.4448876	1288.581483	578.1005324	418.6134101
0	0	0	0	2.586579563	1.36801768
258.5128258	118.5423308	170.7646331	228.8233893	290.9902009	161.4260862
23.93637276	46.04536816	130.7657101	191.916391	53.02488105	143.6418564
2283.529961	1214.813969	1011.511228	405.976981	734.588596	1281.148557
4.787274552	18.614085	29.22998225	25.30765596	14.2261876	16.41621216
253.7255513	775.913438	696.9043137	2292.451836	1077.310388	612.8719206
1362.777489	2905.756638	2359.936462	4433.057736	2841.35765	3640.295046
161.1715766	245.9018598	1382.270476	1419.337705	693.203323	2366.670586
3.191516368	0.979688684	1.538420118	12.65382798	3.879869345	0
15.95758184	5.878132106	7.692100592	4.21794266	12.93289782	17.78422984
338.300735	792.5681456	263.0698403	333.2174701	605.2596179	385.0969769
76.59639283	67.59851922	80.76705622	44.28839793	58.19804018	62.24480444
39.8939546	36.24848132	83.84389646	99.12165251	41.38527302	53.35268952
19.14909821	6.85782079	26.92235207	24.25317029	14.2261876	30.09638896
345.8646288	159.4639271	332.5372007	191.916391	218.5659731	341.2109697
43.08547097	24.49221711	63.07522486	36.90699827	19.39934673	70.45291052
118.0861056	40.16723606	37.6912929	28.47111295	73.71751756	38.98850388

89.77735543	88.54426329	241.1627378	146.8687634	23.34388056	96.08272175
43.08547097	77.39540606	112.3046686	100.1761382	50.43830149	120.3855558
0	59.76100974	39.99892308	6.32691399	19.39934673	19.83625636
103.724282	47.02505685	55.38312426	26.36214162	46.55843214	28.72837128
692.5590518	744.5634001	633.0598787	693.8515675	367.294298	337.9003669
6.383032736	0.979688684	3.846050296	2.10897133	2.586579563	4.10405304
148.4055111	240.0237277	217.6864468	171.8811634	155.1947738	230.5109791
277.661924	379.1395208	216.1480266	506.1531192	187.5270184	266.0794387
169.1503675	301.7441148	146.9191213	224.6054466	193.9934673	201.0985989
54.25577826	55.84225501	133.8425503	73.81399655	64.66448909	111.4934409
73.40487646	228.2674634	181.533574	166.6087351	104.7564723	300.9638896
197.8740148	117.5626421	149.2267515	181.3715344	153.901484	147.0619006
1498.416935	2657.895401	965.3586243	1714.593691	2263.257118	1141.610754
536.1747498	385.9973416	624.5985681	432.3391226	942.8082509	459.6539405
0	1.959377369	3.076840237	14.76279931	5.173159127	2.05202652
63.08032101	19.11372623	39.34509453	34.75584752	71.84224738	106.7258993
207.4485639	125.4001516	179.2259438	159.2273354	177.1807001	136.801768
98.93700741	296.8456714	294.6074527	76.97745354	222.4458425	441.8697106
97.19763099	92.79611218	231.9706776	66.2849689	76.93780912	221.4068214
156.384302	1629.222282	316.9145444	180.3170487	164.2478023	649.808398
271.2788913	275.4786611	186.1488343	235.1503033	166.7179858	230.1826548
47.87274552	13.71564158	34.61445267	30.58008428	32.33224454	45.82859228
0.797879092	0	0	0	0	8.495389792
22.34061458	54.86256632	88.45915681	49.56082625	43.97185258	97.81326411
49.4685037	16.65470763	38.46050296	24.25317029	28.4523752	17.100221
0	0	9.99973077	2.10897133	93.11686429	5.47207072
398.939546	565.2803709	346.9137367	606.3292574	1413.669195	319.4321283
414.8971278	359.5457471	234.6090681	308.9642998	217.2726833	235.2990409
25.53213094	33.30941527	76.92100592	68.54156822	32.33224454	52.66868068
57.44729462	38.20785869	32.30682249	62.21465423	76.30409712	23.25630056
1461.714497	172.4252084	562.2925533	393.323153	153.901484	420.6654366
0	30.37034921	1.538420118	3.163456995	1.293289782	0
673.4099536	587.8132106	474.6026065	651.6721409	1052.737882	451.4458344
44.68122915	25.47190579	55.38312426	39.0159696	62.07790952	54.03669836
1.595758184	32.32972658	9.230520711	0	11.63960804	19.83625636
563.5898754	1154.82763	992.480971	890.3549712	858.4857571	561.8585413
84.57518375	47.02505685	48.46023373	41.12494093	32.33224454	79.34502544

68.61760191	113.6438874	123.0736095	99.12165251	129.3289782	121.0695647
601.6008354	693.6195885	480.756287	686.4701679	876.850472	331.7442874
116.4903474	78.37509475	249.9932692	127.5927655	150.0216147	291.3877658
480.3232134	19.59377369	180.7643639	50.61531192	47.85172192	23.9403094
14.36182366	104.8266892	21.53788166	171.8811634	37.50540367	19.83625636
9.574549104	24.49221711	33.84524261	15.81728497	7.75973869	56.77273372
12191.59253	115.6032648	5093.709012	1552.202899	14901.28487	3509.649358
429.2589515	50.94381159	21.53788166	18.98074197	63.37119931	214.0947669
801.9801905	563.3209935	465.3720858	755.0117361	571.6340835	547.131831
430.8547097	252.7596806	325.3758551	607.383743	562.5810551	431.609578
9349.451454	4296.885179	4605.860608	6503.255628	4653.230769	4112.144864
0	0	0	3.163456995	0	1.36801768
177.1291584	98.94855712	115.3815089	125.4837941	151.3149045	79.34502544
30.3194055	27.44108005	32.30682249	39.0159696	42.6785628	54.03669836
1161.711958	1423.487658	1612.264284	1582.782983	2339.561215	825.5986698
1685.120642	3209.46013	2223.786281	5797.562186	3428.511211	1383.749883
301.6461695	235.3604095	215.6865006	273.1328769	362.7160522	470.6322823
0	0.979688684	3.076840237	6.32691399	0	2.05202652
154.7885438	937.5620709	264.6082604	395.4321244	377.6406163	321.4841548
23.93637276	18.614085	25.38393195	2.10897133	16.81276716	10.94414144
6.383032736	3.918754737	3.076840237	21.0897133	19.39934673	2.05202652
180.3206748	167.526765	211.5327663	276.2752442	243.138479	245.5591735
754.793621	1170.727978	873.0534172	2065.737418	1260.957537	840.6468643
0	5.878132106	16.15341124	2.10897133	2.586579563	58.1407514
181.916433	132.0914253	133.4733295	112.545255	88.78434352	146.2000495
0	0	0	0	0	0
0	9.796886843	239.2243284	460.8102356	387.9869345	102.601326
9.574549104	6.85782079	19.23025148	17.9262563	9.053028472	23.9403094
25.53213094	655.4117298	576.9075444	61.16016857	131.9155577	515.7426653
97.34124922	185.1611613	478.4486568	238.3137603	231.4988709	239.403094
617.5584172	567.2397482	594.5993758	641.1272843	580.687112	634.7602035
355.854075	368.3629453	288.4537722	419.6852947	384.1070652	365.9447294
55.85153644	113.6438874	124.335114	175.0446204	212.0995242	62.24480444
148.4055111	886.6182593	827.6700237	378.5603537	483.6903784	1045.849516
20.74485639	0	9.230520711	8.43588532	3.879869345	2.73603536
531.3874753	281.121668	971.4969206	282.6021582	620.7790952	440.583774
1.595758184	0.979688684	19.23025148	2.10897133	3.879869345	7.52409724

3464.391017	4448.766316	803.8245119	3283.668361	5240.410196	1320.137061
266.4916167	158.7095669	193.0717249	312.1277568	237.9653198	205.8866608
0	0	1.538420118	0	0	11.62815028
0	0	0	0	0	1.36801768
127.6446971	155.2022814	373.851473	266.4685275	262.7706179	437.5057342
47.58550905	183.3781279	130.7041733	187.3504681	120.5992721	220.5860108
1164.903474	1013.977788	796.9016214	1151.498346	1899.842689	596.4557084
148.4055111	995.3637033	361.5287278	544.1146031	629.8321237	582.0915228
148.4055111	189.0799161	149.2267515	209.8426473	159.0746432	153.2179802
60.63881099	106.7860666	13.07657101	7.381399655	155.1947738	32.83242432
173.9376421	259.6175013	250.7624793	339.5443841	157.7813534	291.3877658
20.74485639	8.817198159	33.07603255	8.43588532	7.75973869	15.04819448
47.87274552	517.2756253	335.3755858	255.1855309	259.9512461	807.1304312
1.595758184	11.75626421	2.307630178	2.10897133	6.466448909	6.15607956
215.0124577	267.0827291	673.4280227	278.0889596	109.864967	232.2415214
0	0	0	0	0	0.608767868
46.27698734	14.69533026	36.92208284	18.98074197	15.51947738	15.04819448
87.76670012	91.11104764	163.8417426	141.3010791	133.2088475	139.5378034
1448.948431	17926.34355	4614.491145	21854.21541	22867.94992	5824.335272
110.1073147	33.30941527	21.53788166	235.1503033	155.1947738	27.3603536
734.0487646	532.9506443	425.3731628	542.0056318	557.4078959	407.6692686
0	0.979688684	0	0	0	0
0	0	0	0	0	0
0	0	0	0	0	0
0	0	0.830746864	0	0	0
0	0	0.769210059	0	0	0
0	0	0	0	0	0.711369194
0	0	0	0	0	0.697689017
80.69749136	81.90197401	76.98254273	41.87362576	79.11053595	98.42887207
225.0019039	42.12661343	41.5373432	54.83325458	87.94370516	106.0213702
36.70243823	58.78132106	39.99892308	71.70502522	47.85172192	60.87678676
44.68122915	92.09073633	106.1509882	115.9934231	91.8235745	114.9134851
325.5346695	353.667615	260.7622101	194.0253624	309.0962578	344.7404553
169.1503675	148.91268	226.9169675	171.8811634	287.1103315	227.7749437
52.66002007	32.32972658	27.69156213	44.28839793	41.38527302	54.03669836
4.787274552	9.796886843	6.922890533	4.21794266	6.466448909	10.2601326
625.5372081	1098.231015	1376.886006	1289.635968	1590.746432	467.8620465

351.0668005	512.3771819	526.1396805	424.957723	342.7217922	307.803978
949.4761195	766.1165511	979.2044054	841.4795606	1546.774579	872.111271
191.4909821	98.94855712	93.07441717	159.2273354	124.155819	129.2776708
20.74485639	6.85782079	306.9148136	82.24988187	67.25106865	868.6912268
1.595758184	2.939066053	7.838250504	2.10897133	12.93289782	4.206654366
336.7049768	367.3832566	410.7581716	374.3424111	215.9793936	389.8850388
189.8952239	28.41097185	63.07522486	30.58008428	67.25106865	54.03669836
47.87274552	159.6892555	187.6872545	172.9356491	131.9155577	716.1572554
20.74485639	104.8266892	34.61445267	68.54156822	86.65041538	126.5416354
70.2133601	48.98443422	96.1512574	66.43259689	58.19804018	64.9808398
421.2801606	389.9160964	406.9121213	416.5218377	384.1070652	348.1604995
102.1285238	167.526765	153.0728018	308.9642998	186.2337286	217.5148111
137.2352038	615.2444938	504.6017989	335.3264415	531.5421003	406.3012509
188.2994657	63.67976448	47.69102367	33.74354128	46.55843214	54.03669836
55.85153644	130.298595	119.2275592	102.2851095	99.58331319	112.1774498
0	1.959377369	2.307630178	1.054485665	3.879869345	3.4200442
51.06426189	16.65470763	8.461310651	2.10897133	1.293289782	6.8400884
68.61760191	2.939066053	26.92235207	10.54485665	15.51947738	8.20810608
8418.390384	6770.87342	7111.377766	4646.78089	3612.132495	3840.668596
234.576453	99.9282458	396.9123906	2046.756676	350.4815309	653.912451
12.76606547	36.24848132	11.53815089	45.34288359	7.75973869	13.6801768
3596.838947	1342.173498	754.5950681	1308.61671	1048.858013	652.5444333
35.10668005	52.90318895	88.45915681	169.7721921	45.26514236	181.9463514
38.29819642	73.47665132	115.3815089	128.6472511	87.94370516	72.50493704
682.9845027	355.6269924	483.0639172	410.1949237	372.4674571	274.2875448
36.70243823	107.7657553	112.3046686	29.52559862	134.5021373	90.97317571
87.76670012	70.53758527	66.92127515	70.65053955	98.29002341	106.0213702
7.97879092	61.72038711	57.69075444	23.19868463	71.130938	125.1736177
151.5970275	728.8883811	66.92127515	142.3555648	503.0897251	132.697715
9.574549104	45.06567948	64.61364497	42.1794266	27.15908542	31.46440664
521.8129262	370.3223227	419.2194823	302.6373858	424.1990484	240.0871028
0	0	0	0	0	0
130.8521711	139.1157932	129.9965	167.6632207	134.5021373	127.9096531
17.55334002	39.18754737	1.538420118	255.1855309	24.57250585	10.94414144
33.51092186	36.24848132	46.15260355	45.34288359	49.14501171	75.2409724
0	0	0	0	0	0
1.595758184	2.939066053	0.769210059	3.163456995	1.293289782	1.36801768

140.4267202	316.439445	48.46023373	300.5284145	1317.862288	133.3817238
19.81931665	172.2488645	263.3775243	201.8918254	167.7914163	195.2161229
392.5565133	262.5467705	224.6401057	268.8938446	336.2553433	234.6150321
1.595758184	0.979688684	0	2.10897133	0	2.05202652
595.2178026	2230.751134	1284.580799	1322.325024	1140.681588	983.6047119
68.61760191	68.5782079	126.1504497	72.75951088	46.55843214	115.597494
0	0	0	0	0	0
0	3.918754737	5.384470415	3.163456995	0	21.88828288
98.93700741	45.06567948	176.1491036	51.66979758	64.66448909	69.08489284
438.8335006	446.7380401	553.8312426	430.2301513	384.1070652	412.4573305
3.191516368	3.918754737	8.461310651	2.10897133	0	7.52409724
31.91516368	41.48981578	50.6678666	63.65929959	31.03895476	31.46440664
702.133601	488.8646535	472.2949764	513.5345188	492.7434068	378.2568885
44.68122915	128.3392176	59.99838462	127.5927655	72.42422778	110.1254232
181.916433	73.47665132	107.6894083	49.56082625	82.77054603	56.08872488
780.325752	317.4191337	508.4478491	1423.555648	1364.42072	422.7174631
3.191516368	0	0	0	0	0
113.2988311	134.2173498	46.92181361	129.7017368	91.8235745	64.29683096
12.76606547	37.22817	23.07630178	29.52559862	11.63960804	22.57229172
116.4903474	196.9174256	193.8409349	224.6054466	322.0291557	134.0657326
98.76147401	213.6113207	134.5656078	118.9565279	10.03592871	114.9750459
799.4748502	226.3080861	774.5945296	345.8712981	252.1915074	308.4879868
12.76606547	9.796886843	6.153680474	8.43588532	16.81276716	6.8400884
612.7711426	499.641229	1026.126219	883.6589872	636.2985726	532.8428863
55.85153644	93.07042501	66.15206509	227.7689036	201.753206	67.71687516
15.95758184	120.5017082	180.7643639	0	0	147.7459094
31.91516368	2754.88458	3612.210438	543.0601175	532.8353901	2781.863952
46.27698734	15.67501895	82.30547634	165.5542494	72.42422778	62.92881328
7.97879092	18.614085	51.53707397	73.81399655	23.27921607	77.29299892
0	0	1.538420118	0	0	0
0	0	0.769210059	0	0	0.68400884
108.5115565	88.17198159	51.53707397	88.57679586	68.54435843	72.50493704
0	0	0	0	0	0
0	1.959377369	0.769210059	4.21794266	3.879869345	0.68400884
4594.187812	4630.008722	3720.669056	2633.050705	4490.302122	3643.031082
622.3456918	985.5668164	809.9781924	690.6881106	531.5421003	1011.649074
188.2994657	45.06567948	36.92208284	82.24988187	36.21211389	42.40854808

6.383032736	11.75626421	153.0728018	4.21794266	10.34631825	84.13308732
3.191516368	0	69.22890533	13.70831364	2.586579563	7.52409724
0	3.918754737	0	0	0	0
130.8521711	384.0379643	76.92100592	261.5124449	126.7423986	393.305083
4.787274552	1.959377369	5.384470415	0	6.466448909	0.68400884
193.0867403	169.4861424	163.8417426	268.8938446	208.2196549	162.7941039
3.191516368	1.959377369	0.769210059	0	2.586579563	0
135.6394456	106.7860666	131.5349201	131.8107081	166.8343818	110.8094321
110.1073147	93.07042501	92.30520711	62.21465423	64.66448909	82.0810608
9.574549104	50.94381159	19.23025148	25.30765596	49.14501171	34.200442
39.8939546	55.84225501	83.84389646	108.6120235	71.130938	114.2294763
309.5770877	247.8612371	386.9126598	417.5763233	389.2802243	437.0816487
92.55397467	247.8612371	344.6061065	142.3555648	164.2478023	202.4666166
7.97879092	0.979688684	80.76705622	0	0	64.29683096
62.23456918	157.7298782	161.5341124	54.83325458	113.8095008	124.4896089
114.8945892	164.587699	134.6117604	40.07045527	96.99673363	119.701547
199.469773	271.3737656	142.303861	228.8233893	131.9155577	132.697715
1.595758184	7.837509475	10.76894083	7.381399655	1.293289782	16.41621216
307.9813295	455.5552382	259.22379	328.9995275	301.3365191	363.208694
31.91516368	2.939066053	16.15341124	14.76279931	2.586579563	15.04819448
320.747395	595.6507201	376.143719	499.8262052	398.3332528	502.7464974
0	4.898443422	3.076840237	1.054485665	5.173159127	5.47207072
19.14909821	10.77657553	13.07657101	11.59934231	7.75973869	24.62431824
4.787274552	13.71564158	1.538420118	17.9262563	21.98592629	8.89211492
0	0	2.307630178	2.10897133	2.586579563	0
349.4710423	527.0725122	383.0666095	547.2780601	362.1211389	262.6593945
8.122409156	3.037034921	1.569188521	3.226726135	5.289555207	0.690848928
3.191516368	0	6.153680474	6.32691399	2.586579563	4.78806188
154.7885438	172.4252084	184.6104142	115.9934231	107.3430519	214.0947669
0	0	0.769210059	0	0	5.47207072
81.38366738	150.8720574	7.692100592	162.3907924	203.0464957	31.46440664
0	19.59377369	20.7379032	19.41308109	41.38527302	7.838741306
3.191516368	1.959377369	10.76894083	0	3.879869345	8.20810608
3.191516368	20.57346237	10.76894083	15.81728497	12.93289782	8.20810608
130.8521711	48.00474553	144.6114911	52.72428325	65.95777887	58.82476024
8.872415503	5.505850406	1.776875237	1.054485665	11.63960804	5.47207072
0	0	0	0	0	0

413.3013697	321.3378885	209.2251361	313.1822425	217.2726833	216.8308023
4.787274552	33.30941527	169.226213	46.39736926	63.37119931	114.9134851
279.2576822	370.3223227	198.4561953	481.8999489	300.0432294	293.4397923
459.578357	344.8504169	228.4553876	239.3682459	200.4599162	246.9271912
4612.794352	3888.854639	1811.935831	2178.430301	3048.788399	2838.472524
595.2178026	512.3771819	366.9131983	219.3330183	382.8137754	545.1550454
0	0	0	0	0	2.05202652
1172.882265	1795.769358	714.596145	2096.317502	1076.017098	687.4288842
75.00063465	68.5782079	63.07522486	100.1761382	55.61146062	89.60515804
231.3849367	232.1862182	218.4556568	176.099106	193.9934673	207.2546785
1042.030094	2014.239935	1145.353778	2015.122106	2411.985443	971.9765616
304.7898131	843.5119572	744.5953373	1226.366828	666.0442376	442.5537195
67.02184373	144.9939253	581.5228048	133.9196795	280.6438826	544.4710366
314.3643622	797.466589	906.3448286	850.9699316	903.3499797	1441.206626
697.3463264	373.2613887	337.683216	645.345227	544.4749981	304.3839338
33.51092186	0.979688684	2.307630178	1.054485665	0	7.52409724
0	0	1.538420118	0	0	0
682.9845027	286.0690958	374.6052988	453.4288359	362.1211389	327.6402343
234.576453	237.0846616	140.7654408	171.8811634	212.0995242	111.4934409
217.023113	317.4191337	122.3043994	149.7369644	210.8062344	128.5936619
1528.800171	367.5008193	150.4728718	734.9659636	505.3012506	142.4311607
1.595758184	1.959377369	3.076840237	1.054485665	9.053028472	21.20427404
0	18.614085	41.5373432	67.48708256	78.89067669	30.7803978
384.5777223	310.5613129	533.062571	282.6021582	86.65041538	515.7426653
70.2133601	45.06567948	54.6139142	62.21465423	69.83764821	49.93264532
27.12788913	17.63439632	58.4599645	44.28839793	31.03895476	45.82859228
315.9601204	225.3283974	227.6861775	241.4772173	426.785628	175.7902719
0	0	0	0	0	0.68400884
111.7030729	248.8409258	156.9188521	220.387504	139.6752964	202.4666166
702.133601	683.8227017	903.0526095	737.0854798	1407.099283	1053.373614
20.74485639	28.41097185	28.46077219	27.41662729	34.91882411	19.83625636
15.95758184	45.06567948	35.38366272	28.47111295	40.09198323	31.46440664
87.76670012	247.8612371	199.2254053	252.0220739	322.0291557	253.0832708
0	0.979688684	43.84497338	29.52559862	62.07790952	160.7420774
269.6831331	157.7298782	198.4561953	166.6087351	197.8733366	124.4896089
156.384302	124.4204629	103.0741479	269.9483302	116.3960804	136.1177592
0	0	0	0	0	4.10405304

3994.182735	11393.7794	3674.516453	4663.990096	10920.53892	7302.478375
82.97942557	393.8348511	182.302784	469.2461209	232.7921607	270.8675006
0	1.734048971	1.161507189	1.370831364	0	0
38.29819642	86.21260422	79.99784616	110.7209948	177.1807001	108.0733967
1961.186808	2594.215636	4590.645633	3378.572071	938.9283815	5231.983617
0	7.837509475	33.84524261	4.21794266	5.173159127	12.99616796
181.916433	350.728549	807.6705622	466.0826639	371.1741674	566.3593195
0	0	0	0	1.293289782	0
349.4710423	828.8166269	773.8253196	776.1014494	633.7119931	667.5926278
6.383032736	208.6736898	642.2903995	1.054485665	34.91882411	563.6232841
172.3418839	200.8361803	480.756287	190.8619054	206.9263651	288.6517305
212.2358385	68.5782079	204.6098758	57.99671157	43.97185258	125.1736177
0	0	0	0	0	0
11.17030729	7.837509475	176.9183136	8.43588532	12.93289782	32.14841548
288.8322313	349.7488603	185.3796243	201.406762	184.9404388	161.4260862
20.74485639	12.7359529	79.99784616	24.25317029	9.053028472	46.51260112
0	2.939066053	2.307630178	0	0	4.78806188
181.916433	133.2376611	120.7659793	165.5542494	153.901484	235.2990409
126.0648965	961.0745993	569.2154438	526.1883468	363.4144287	1125.878551
0	0	2.307630178	0	3.879869345	5.47207072
0	0	7.692100592	36.90699827	10.34631825	23.25630056
852.1348702	373.2613887	753.056648	586.2940297	613.0193565	881.6873947
225.0019039	310.5613129	153.8420118	345.8712981	437.1319462	116.2815028
395.7480296	479.0677666	353.8366272	359.5796118	397.039963	203.1506255
7429.850105	2409.054475	286.9153521	509.3165762	3083.20284	186.7344133
9.574549104	29.39066053	37.6912929	36.90699827	18.10605694	30.7803978
9.79795525	4.0950987	5.615233432	6.664349403	2.690042746	2.831796597
97.34124922	421.2661343	166.1493728	312.1277568	230.2055812	350.2125261
1.595758184	1.959377369	4.615260355	3.163456995	1.293289782	9.576123759
35.10668005	52.90318895	56.92154438	46.39736926	71.130938	67.71687516
20.74485639	2.939066053	19.23025148	2.10897133	2.586579563	19.83625636
175.5334002	591.7319653	170.7646331	526.1883468	871.6773129	344.7404553
0	0	0	1.054485665	0	2.05202652
15.95758184	321.3378885	132.3041302	194.0253624	113.8095008	93.02520223
288.8322313	166.5470763	143.8422811	196.1343337	102.1698928	137.4857768
0	0	0	0	0	0
185.1079493	287.0487845	306.1456036	285.7656152	283.2304622	356.3686056

1002.13614	92.09073633	519.986	146.5735074	29.74566498	534.8949129
0	0	0	0	0	0
331.9177023	90.13135896	154.6112219	99.12165251	99.58331319	144.3258652
475.5359388	527.0725122	272.300361	320.5636421	309.0962578	509.5865858
615.962659	336.0332187	240.7627485	318.4546708	579.3938222	199.0465724
9.574549104	12.7359529	15.38420118	20.03522763	3.879869345	17.78422984
0	1.959377369	1.538420118	1.054485665	1.293289782	4.78806188
20.74485639	2.939066053	1.538420118	1.054485665	1.293289782	8.89211492
7.97879092	6.85782079	3.076840237	8.43588532	27.15908542	4.10405304
71.80911828	126.3798403	142.303861	75.92296788	78.89067669	144.3258652
848.9433539	920.9073633	894.5912989	1379.26725	977.727075	1142.978772
0	0	0	0	0	0
0	0	0	0	0	0
134.0436875	203.7752463	198.4561953	279.4387012	253.4847972	272.9195271
560.1111226	140.0954819	112.3046686	143.4100504	177.1807001	126.5416354
0	5.349100216	3.930663403	2.151150757	0	1.819463514
0	0	0	0	0	0.68400884
556.9196062	679.9039469	634.5982989	584.1850584	663.457658	718.209282
336.7049768	185.1611613	180.7643639	257.2945023	125.4491088	223.6708907
0	6.85782079	15.38420118	10.54485665	19.39934673	12.31215912
7.97879092	12.7359529	111.5354586	4.21794266	3.879869345	34.88445084
188.2994657	77.39540606	117.6891391	108.6120235	168.1276716	44.4605746
12.76606547	26.45159448	29.99919231	24.25317029	11.63960804	36.93647736
336.7049768	277.2518977	255.3777397	666.4349403	347.8949513	443.9217371
2762.257416	885.1977107	777.3329175	624.4242314	512.1427536	800.0714599
528.1959589	548.6256632	605.3683166	422.8487516	362.1211389	653.912451
63.83032736	94.0501137	110.7662485	95.95819551	142.261876	175.7902719
3.191516368	56.82194369	81.53626628	191.916391	54.31817083	153.901989
6.383032736	81.3141608	96.1512574	192.9708767	65.95777887	123.8056
520.217168	211.6127558	291.5306124	885.7679586	369.8808776	359.7886498
279.2576822	575.0772577	548.4467722	171.8811634	300.0432294	272.9195271
36.70243823	160.6689442	153.0728018	72.75951088	93.11686429	63.61282212
62.23456918	100.9079345	63.07522486	91.74025285	69.83764821	99.18128179
95.74549104	117.9349238	143.6269023	89.63128152	90.53028472	72.10137182
95.74549104	114.6235761	103.843358	92.79473852	91.8235745	79.34502544
3157.478846	510.8586644	510.7554793	235.1503033	415.1460199	243.8012708
135.6394456	143.0345479	222.3017071	282.6021582	225.032422	307.803978

1.595758184	0	0	0	6.466448909	0.68400884
54.25577826	87.19229291	80.76705622	37.96148394	53.02488105	134.0657326
78.19215101	63.67976448	143.073071	66.43259689	85.3571256	125.8576266
51.06426189	160.6689442	126.9196598	75.92296788	126.7423986	114.2294763
445.2165333	771.9946833	689.9814231	585.2395441	888.4900801	354.3165791
11.17030729	10.77657553	18.46104142	22.14419896	5.173159127	20.5202652
330.3219441	337.9925961	268.4543107	279.4387012	490.1568273	104.6533525
6014.412595	1364.706337	1696.877391	773.9924781	2530.968103	3228.521725
543.7546012	963.0339767	686.4969016	964.3165958	563.8743448	849.9767449
41.48971278	177.3236519	146.1499113	76.97745354	50.43830149	129.9616796
137.2352038	243.9424824	229.9938077	228.8233893	204.3397855	237.3510675
247.3425185	294.886294	181.533574	227.7689036	208.2196549	199.7305813
183.5121912	139.1157932	382.2973994	612.6561713	53.02488105	482.910241
154.7885438	100.9079345	98.45888758	65.37811123	104.7564723	91.65718455
0	0	0	0	0	0
98.93700741	93.07042501	112.3046686	183.4805057	80.18396647	142.2738387
0	301.7441148	7.692100592	0	0	136.801768
0	0	0	0	0	0
36.70243823	391.8754737	366.2670618	106.5030522	406.0929915	431.8011005
63.83032736	199.8564916	21.53788166	264.6759019	464.2910316	31.46440664
35.10668005	117.5626421	156.9188521	63.2691399	109.9296314	101.9173172
1412.245993	2314.024672	1278.427118	1674.523236	3030.177959	1062.265728
483.5147297	28.41097185	663.0590711	544.1146031	121.5692395	53.35268952
188.2994657	157.7298782	144.6114911	172.9356491	148.7283249	160.7420774
864.9009357	854.2885327	773.0561095	944.8191558	1170.427252	827.6506964
76.59639283	64.65945317	73.07495563	67.48708256	59.49132996	106.0213702
3825.032367	2096.533784	4952.174361	3543.071834	2856.877128	7202.613085
87.76670012	435.9614645	406.1429113	255.1855309	187.5270184	670.3286632
35.10668005	54.86256632	12.30736095	29.52559862	36.21211389	42.40854808
3.191516368	2.939066053	23.07630178	7.381399655	7.75973869	5.47207072
132.4479293	434.9817758	137.6886006	280.4931869	227.6190016	227.7749437
684.5802609	270.3940769	715.3653551	622.1465423	854.8645457	1672.401614
6.383032736	10.77657553	33.84524261	8.43588532	14.2261876	16.41621216
47.87274552	64.65945317	17.69183136	147.6279931	93.11686429	73.18894588
52.66002007	202.7955577	51.53707397	10.54485665	131.9155577	176.4742807
526.6002007	746.5227775	485.3715474	1145.171432	1122.575531	556.7831957
9.574549104	3.918754737	15.38420118	21.0897133	11.63960804	15.04819448

890.4330667	654.4320411	1019.972539	849.915446	1357.954271	569.7793637
106.9157983	21.55315106	51.53707397	40.07045527	25.86579563	41.72453924
217.023113	219.4502653	505.3710089	394.3776387	226.3257118	386.4649946
1784.05765	845.4713346	1195.352432	801.4091054	1039.804985	1183.335293
248.9382767	275.2925203	320.7605947	418.630809	393.1600937	349.5285172
0	3.918754737	40.76813314	1.054485665	10.34631825	49.24863648
0	0	0	0	0	0
1.595758184	52.90318895	10.76894083	42.1794266	6.466448909	151.8499625
28.72364731	136.1767271	136.9193905	114.9389375	84.06383581	177.8422984
1.595758184	5.878132106	271.5311509	13.70831364	1255.784378	11.62815028
3.191516368	6.85782079	7.692100592	5.272428325	1.293289782	7.52409724
592.0262863	629.939824	560.7541332	639.018313	941.5149611	619.712009
108.5115565	186.14085	200.7638255	100.1761382	237.9653198	201.7826078
6.383032736	8.817198159	41.5373432	10.54485665	6.466448909	8.20810608
113.2988311	200.8361803	104.6125681	220.387504	98.29002341	67.03286632
276.0661658	134.2173498	129.2272899	163.4452781	239.2586096	200.4145901
288.8322313	222.3893313	181.533574	224.6054466	173.3008308	248.9792177
587.1432662	10902.18141	89.18990637	6595.807834	5195.390778	67.78527604
178.7249166	242.9627937	255.3777397	170.8266777	380.2271958	151.1659536
183.5121912	374.2410774	313.8377042	490.3358342	303.9230987	409.7212951
277.661924	80.33447211	160.7649024	84.3588532	109.9296314	136.801768
9.574549104	40.16723606	3.076840237	4.21794266	12.93289782	10.2601326
2036.187443	1709.556754	1713.02311	1409.836789	1655.397988	1485.6672
73.40487646	273.3331429	339.9908462	228.8233893	301.3365191	297.5438454
54.25577826	92.09073633	157.6880621	303.6918715	210.8062344	296.1758277
797.879092	359.5457471	1253.812397	579.9671157	1764.047262	610.8198941
217.023113	298.8050487	177.6875237	214.06059	196.5800468	127.2256442
840.964563	1697.80049	929.2057515	1976.106136	910.4760063	681.2728046
92.55397467	113.6438874	213.0711864	106.5030522	115.1027906	171.00221
0	1.959377369	3.076840237	0	0	5.47207072
4.787274552	637.7773335	77.69021598	179.262563	395.7466732	56.77273372
1.595758184	0.979688684	9.99973077	9.490370985	6.466448909	13.6801768
138.830962	129.3189063	227.6861775	99.12165251	150.0216147	220.2508465
300.0025386	244.9221711	223.0709172	206.6791903	111.2229212	158.006042
4.787274552	0	122.3043994	0	3.879869345	190.1544575
108.5115565	7.837509475	119.2275592	81.1953962	169.4209614	15.73220332
135.6394456	156.7501895	149.2267515	175.0446204	181.0605694	145.0098741

92.55397467	237.0846616	186.1488343	222.4964753	188.8203081	248.2952089
0	0	0	0	0	1.36801768
25.53213094	517.2756253	257.6853698	43.23391226	85.3571256	506.8505504
495.945686	406.482632	443.6726701	357.280833	426.785628	288.6517305
14.36182366	0	6.922890533	3.163456995	0	2.73603536
242.555244	479.0677666	326.1450651	295.2559862	275.4707235	208.6226962
98.93700741	252.7596806	256.9161598	418.630809	247.0183483	220.9348553
9.574549104	14.69533026	25.38393195	7.381399655	6.466448909	48.56462764
100.5327656	96.98917975	209.9943462	183.4805057	91.8235745	227.7749437
1418.629026	746.5227775	509.2170592	418.630809	603.9663281	463.7579935
110.1073147	2.939066053	113.8430888	0	1.293289782	17.100221
63.83032736	198.8768029	275.3772012	152.9004214	146.1417453	258.5553415
202.6612894	126.3798403	188.4564645	162.3907924	173.3008308	101.2333083
379.7904478	158.7095669	338.4524261	339.5443841	256.0713768	314.6440664
0	0	0.769210059	0	0	0.38304495
114.8945892	49.9641229	28.46077219	128.6472511	43.97185258	32.14841548
54.25577826	5.878132106	0	7.381399655	58.19804018	58.1407514
237.7679694	1010.059034	490.7560178	229.877875	416.4393097	739.413556
114.8945892	66.61883053	24.6147219	87.52231019	76.30409712	56.77273372
938.3058122	21.55315106	4.615260355	24.25317029	29.74566498	0
615.962659	355.6269924	495.3712781	392.2686674	395.7466732	520.5307272
167.5546093	294.886294	338.4524261	308.9642998	368.5875878	324.2201901
127.6606547	266.4753221	195.379355	321.6181278	430.6654973	228.4589525
596.8135608	1129.581053	1383.808897	700.1784815	1109.642633	1418.634334
1065.966467	583.8944559	923.8212811	431.284637	711.30938	577.3034609
36.70243823	127.359529	180.7643639	175.0446204	121.5692395	181.2623426
418.0886442	591.7319653	605.3683166	685.4156822	518.6092025	680.5887958
0	0	0	0	0	0
106.9157983	24.49221711	19.23025148	56.94222591	49.14501171	30.09638896
807.4376835	320.3581998	507.6094102	623.201028	603.9663281	359.7065688
6.383032736	3.918754737	11.53815089	8.43588532	15.51947738	28.04436244
31.91516368	56.82194369	46.15260355	40.07045527	69.83764821	71.8209282
0	20.57346237	24.6147219	20.03522763	37.50540367	49.93264532
2194.167503	894.4557688	1378.424426	1651.324551	2512.862046	1097.834188
79.7879092	25.47190579	247.6856391	0	14.2261876	467.8620465
1.595758184	1.959377369	2.307630178	5.272428325	1.293289782	9.576123759
137.2352038	118.5423308	85.38231657	80.14091054	41.55340069	73.18894588

980.3859555	1378.431776	253.4777908	1082.41899	715.5513704	395.5417919
526.6002007	914.0495425	870.745787	397.5410957	534.1286799	971.9765616
164.3630929	243.9424824	254.6085296	289.9835579	219.8592629	162.7941039
156.384302	389.9160964	258.4545799	528.2973181	434.5453667	402.8812067
1314.904744	667.167994	2270.708095	508.2620905	904.0095574	1123.142515
263.3001004	5208.025046	299.9919231	1027.069038	297.4566498	293.4397923
234.576453	254.7190579	242.3011687	396.48661	722.948988	180.5783338
0	15.67501895	24.6147219	11.59934231	0	51.300663
237.7679694	265.4956335	293.8382426	223.550961	297.4566498	261.2913769
4.787274552	27.43128316	22.30709172	35.85251261	45.26514236	21.88828288
15.95758184	20.57346237	25.38393195	16.87177064	14.2261876	38.30449504
7.97879092	2.939066053	6.153680474	3.163456995	7.75973869	1.36801768
893.624583	934.6230048	822.2855533	1404.574906	1559.707477	685.3768576
54.25577826	76.41571738	41.5373432	86.46782453	94.41015407	135.4337503
3.191516368	2.939066053	10.76894083	6.32691399	1.293289782	21.20427404
738.8360392	347.7894829	395.3739704	306.8553285	642.7650215	535.5789217
22.34061458	1.969174256	22.30709172	3.226726135	7.772671588	28.16064394
678.0695675	1153.691192	417.6810622	673.6581671	623.3656748	484.0388556
287.2205155	0.979688684	2.307630178	145.5084769	28.4523752	0.68400884
26.29809487	64.82600024	0.838438965	0	101.3680531	33.63271466
118.0861056	127.359529	239.2243284	90.68576719	107.3430519	235.2990409
172.3418839	433.0223985	166.1493728	320.5636421	366.0010082	425.4534985
360.6413496	61.72038711	84.61310651	5.272428325	210.8062344	7.52409724
1605.332733	1199.13895	1253.043186	1560.638784	2294.296073	1708.654082
11.17030729	0	6.153680474	4.21794266	9.053028472	3.4200442
304.7898131	428.1239551	497.6789083	392.2686674	256.0713768	337.2163581
188.2994657	115.6032648	147.6883314	130.7562225	203.0464957	87.55313152
205.8528057	302.7238035	259.993	360.6340974	294.8700702	263.3434034
1430.645085	1058.151951	905.6525395	504.845557	532.4215373	684.6928488
756.3893792	872.9026177	878.4378876	2273.471094	725.5355676	857.7470853
31.91516368	168.5064537	157.6880621	6.32691399	24.57250585	80.71304312
68.61760191	70.53758527	43.84497338	44.28839793	10.34631825	17.78422984
121.277622	668.1476827	866.8997367	219.3330183	373.7607469	915.2038279
1326.075051	504.5396724	506.909429	687.5246536	1109.642633	644.3363272
11.17030729	28.41097185	49.99865385	15.81728497	7.75973869	54.03669836
0	9.796886843	13.07657101	9.490370985	43.97185258	15.73220332
304.7898131	586.8335219	493.832858	652.7266266	475.9306397	333.1123051

347.8752841	238.0643503	106.9201982	160.2818211	184.9404388	186.0504045
7.97879092	17.63439632	17.69183136	9.490370985	14.2261876	21.20427404
307.0398322	61.82815287	70.03657589	220.2504208	22.71016857	80.17267613
2100.01777	4790.677666	3936.047873	1443.590875	2616.325228	4612.271608
11.17030729	0.979688684	20.7686716	9.490370985	6.466448909	8.89211492
611.1753845	653.4523524	539.9854616	448.1564076	527.662231	397.409136
0	1.959377369	0.769210059	2.10897133	2.586579563	0.68400884
161.1715766	153.8111234	116.1507189	182.42602	168.1276716	266.7634476
124.4691384	111.68451	152.3035917	156.0638784	129.3289782	225.0389083
0	0	1.538420118	1.054485665	0	0
4.787274552	9.796886843	10.76894083	22.14419896	15.51947738	34.88445084
38.29819642	62.7000758	85.38231657	70.65053955	80.18396647	139.5378034
4.787274552	184.1814727	66.15206509	57.99671157	38.79869345	212.7267492
804.2621247	482.0068327	744.5953373	833.0436753	640.178442	745.5696356
1613.311524	606.4272956	696.9043137	584.1850584	651.81805	593.0356642
229.7891785	360.5254358	344.6061065	327.9450418	494.0366966	339.2683846
0	0	0	0	0	0
7.97879092	6.85782079	2.307630178	28.47111295	9.053028472	3.4200442
304.7898131	182.2220953	206.1482959	510.3710618	360.8278491	162.7941039
0	0.979688684	0.769210059	2.10897133	3.879869345	2.73603536
43.08547097	118.5423308	179.2259438	168.7177064	109.9296314	201.7826078
499.4723116	59.76100974	273.069571	261.5124449	18.10605694	678.5367692
0	1.959377369	1.538420118	2.10897133	3.879869345	1.36801768
204.2570475	144.9939253	151.5343817	122.3203371	117.6893701	106.705379
0	4.898443422	43.84497338	0	0	8.20810608
202.6612894	419.0324441	775.1099004	167.0832536	205.6589411	758.6684048
150.0172269	192.9986708	216.9172367	214.06059	191.4068877	222.302873
175.5334002	143.0345479	202.3022456	217.224047	156.4880636	214.7787757
293.6195059	204.754935	389.22029	286.8201009	592.32672	283.1796597
135.6394456	123.4407742	78.45942604	99.12165251	76.30409712	64.29683096
301.5982968	206.7143124	207.686716	180.3170487	319.4425761	309.8560045
655.8566136	324.2769545	368.4516184	653.7811123	659.5777887	222.302873
4040.459722	2761.742401	1909.948577	4684.025324	6201.324503	2989.118631
47.87274552	56.82194369	69.22890533	50.61531192	67.25106865	107.3893879
0	0	1.538420118	2.10897133	0	3.4200442
7.97879092	0	3.076840237	1.054485665	2.586579563	6.8400884
63.83032736	139.1157932	68.45969527	100.1761382	56.9047504	41.0405304

0	1.959377369	10.76894083	1.054485665	0	6.15607956
0	0	4.615260355	8.43588532	0	0
164.3630929	162.6283216	170.7646331	182.42602	245.7250585	160.7420774
14.36182366	69.55789659	10.76894083	421.794266	178.4739899	34.200442
35.10668005	45.06567948	210.7635562	204.570219	91.8235745	230.5109791
370.2158987	2301.288719	2887.614562	1730.410976	2705.562223	1105.358285
0	0	0	0	0	0
0	0	0	0	0	0
0	0.979688684	2.307630178	0	0	1.36801768
0	0	3.846050296	0	0	3.4200442
116.4903474	451.6364835	196.9177752	670.6528829	518.6092025	255.8193061
323.9389113	1089.413817	683.0585326	102.2851095	792.7866362	1057.477667
38.29819642	50.94381159	108.4586184	40.07045527	42.6785628	57.45674256
90.95821649	134.2173498	406.1429113	264.6759019	257.3646666	428.1895338
62.23456918	82.29384948	59.22917456	81.1953962	77.5973869	93.02520223
76.59639283	82.29384948	109.2278284	75.92296788	170.7142512	121.7535735
23.93637276	4.898443422	19.99946154	17.9262563	14.2261876	17.100221
97.34124922	15.67501895	37.6912929	95.95819551	29.74566498	27.3603536
57.44729462	120.5017082	189.2256746	74.86848221	73.71751756	162.7941039
191.4909821	197.8971142	212.3019763	221.4419896	213.392814	249.6632266
847.3475957	292.9269166	233.0706479	558.8774024	390.5735141	336.5323493
11.17030729	26.45159448	9.99973077	13.70831364	12.93289782	7.52409724
122.8733802	59.76100974	135.3809704	28.47111295	43.97185258	167.5821658
143.6182366	121.4813969	143.8422811	333.2174701	226.3257118	200.4145901
510.6426189	43.10630211	400.7584409	18.98074197	10.34631825	244.8751647
1389.905378	2035.793086	1022.280169	1049.213237	3198.30563	577.9874698
1237.861538	770.1626654	413.2273359	1208.166406	684.473617	180.4278518
0	0	0	0	0	0
0	0	0	0	0	0
33.51092186	151.8517461	72.30574557	121.2658515	96.99673363	76.60899008
0	0	0	0	0	0
7.97879092	4.898443422	15.38420118	10.54485665	5.173159127	8.20810608
156.384302	459.473993	149.9959615	450.2653789	358.2412695	351.5805437
25.53213094	188.1002274	439.2189438	342.7078411	73.71751756	521.8987449
3260.532909	1755.48456	1930.217262	2759.030108	2093.202445	2321.34132
256.9170676	2241.52771	73.84416569	0	813.4792727	350.8965349
1.595758184	0	3.076840237	1.054485665	1.293289782	0.68400884

31.91516368	27.43128316	73.84416569	80.14091054	23.27921607	56.08872488
4019.714865	911.1104764	457.6799852	3737.097197	3358.673563	3916.634618
0	0.979688684	1.538420118	1.054485665	1.293289782	1.36801768
35.10668005	201.815869	253.0701095	11.59934231	95.70344385	318.0641106
1348.415665	1741.886481	1179.199021	1899.128683	1013.939189	975.3966058
5757.495528	2681.407929	4841.408113	4039.734582	11823.25518	2788.020032
1.595758184	43.10630211	28.46077219	13.70831364	12.93289782	54.7207072
3.191516368	33.30941527	0.769210059	42.1794266	23.27921607	2.05202652
0	0	0	2.10897133	0	0
9.574549104	23.51252842	104.6125681	27.41662729	38.79869345	88.23714036
3.191516368	4.898443422	2.307630178	8.43588532	0	5.47207072
6.383032736	9.796886843	30.76840237	16.87177064	6.466448909	23.9403094
0	0	0	0	0	0
0	0	0	0	0	0
135.6394456	231.2065295	196.1485651	292.0925292	482.3970886	343.3724377
547.3450571	0.979688684	80.76705622	214.06059	402.2131221	0.68400884
295.215264	155.7705008	223.8401272	216.1695613	259.9512461	177.8422984
611.1753845	560.3819274	449.9878846	486.1178915	341.4285024	337.9003669
0	0	0	0	0	0
22.34061458	113.6438874	164.6109527	6.32691399	33.62553433	65.66484864
0	0	1.538420118	0	0	1.36801768
12.76606547	78.37509475	0.769210059	66.43259689	0	15.04819448
30.3194055	68.5782079	113.8430888	153.9549071	113.8095008	227.7749437
43.08547097	1.959377369	1.538420118	0	5.173159127	0.68400884
169.1503675	150.8720574	304.6071835	128.6472511	234.0854505	162.7941039
0	0	2.307630178	0	1.293289782	4.78806188
2245.231765	1487.167423	2927.613485	1750.446204	2644.777604	1953.529247
421.2801606	510.4178045	216.1480266	376.4513824	239.2586096	309.1719957
406.9183369	361.5051245	381.5281894	345.8712981	278.0573031	339.2683846
31.91516368	19.59377369	19.23025148	20.03522763	18.10605694	50.61665416
113.2988311	296.8456714	788.4403107	299.4739288	446.1849747	270.8675006
512.2383771	1004.180901	871.5149971	636.9093416	633.7119931	730.5214411
38.29819642	0.979688684	206.9175059	163.4452781	0	138.8537945
726.0699737	313.500379	366.9131983	626.364485	488.8635375	272.2355183
143.6182366	363.4645019	128.4580799	275.2207586	208.2196549	193.5745017
220.2146294	376.2004548	409.9889616	130.7562225	237.9653198	370.7327913
0	0	0.769210059	1.054485665	0	4.10405304

59.47390752	162.7458842	135.8271123	156.5700315	147.6419615	162.2674171
68.61760191	15.67501895	27.69156213	21.0897133	71.130938	110.8094321
7.97879092	0.979688684	9.230520711	3.163456995	1.293289782	5.47207072
18.25547362	2.331659069	7.453645474	8.43588532	7.75973869	10.2601326
0	24.49221711	19.99946154	15.81728497	12.93289782	37.6204862
473.9401806	477.1083893	519.986	572.5857161	366.0010082	500.6944709
0	0	0	0	0	0
3.191516368	21.55315106	20.7686716	1.054485665	5.173159127	17.78422984
12.76606547	53.88287764	93.07441717	39.0159696	77.5973869	129.2776708
697.3463264	34.28910395	59.99838462	27.41662729	200.4599162	36.25246852
92.55397467	135.1970384	178.4567337	89.63128152	168.1276716	109.4414144
1.595758184	0	1.538420118	1.054485665	1.293289782	2.05202652
2107.996561	1741.886481	716.9037752	1476.279931	2051.157594	668.9606455
9.574549104	19.59377369	37.6912929	7.381399655	24.57250585	44.4605746
882.4542757	730.8477585	598.4454261	1328.651938	928.5820633	1000.020924
22.34061458	10.77657553	49.99865385	0	6.466448909	34.200442
0	0	0	0	0	0
0	0	0	0	1.293289782	0
157.9800602	79.35478343	93.84362723	76.97745354	62.07790952	85.501105
1602.141217	904.2526556	1097.662755	1272.764198	1436.844948	1023.277225
143.6182366	112.6641987	109.9970385	132.8651938	162.9545125	101.2333083
233.4594223	417.2788013	140.8808223	561.1445466	533.2492428	324.9383994
65.42608554	21.55315106	24.6147219	8.43588532	41.38527302	36.25246852
6.383032736	3.918754737	3.076840237	5.272428325	3.879869345	3.4200442
86.58583906	347.4074043	360.5056785	314.5952533	367.0227072	427.3687232
6.383032736	4.898443422	3.846050296	3.163456995	0	8.89211492
1549.481197	2671.611042	4151.42669	912.1301002	2217.991976	3586.258348
0	0	0	6.32691399	0	0
4651.635106	4895.504356	2653.774704	6202.484681	8418.023189	2017.142069
569.6856717	4689.769732	1822.25863	1372.940336	1730.421728	4977.532328
0	7.837509475	6.153680474	1.054485665	2.586579563	0.68400884
6.383032736	15.67501895	13.07657101	0	6.466448909	24.62431824
0	0	1.538420118	0	0	0
41.48971278	43.10630211	64.61364497	39.0159696	115.1027906	65.66484864
1405.86296	903.272967	2229.170752	894.2038439	1226.038713	2333.838162
13362.87903	11309.52617	3833.742935	2606.688564	8085.647715	4199.130269
4.787274552	111.68451	89.22836687	15.81728497	53.02488105	190.1544575

0	0	0.769210059	2.10897133	0	0
856.9221448	718.1118056	92.30520711	1056.594636	1467.883902	242.1391293
0	0	0	0	1.293289782	0
789.5173191	3034.713059	1467.89894	1869.118021	2944.484578	1395.583236
31.91516368	21.55315106	86.92073669	72.75951088	24.57250585	41.72453924
3126.090282	1935.86484	1518.420657	3122.332054	2459.837165	1463.778918
126.0648965	107.7657553	129.9965	144.4645361	205.6330753	112.1774498
12.76606547	23.51252842	16.9226213	15.81728497	20.69263651	48.56462764
9462.846031	26628.91813	16463.4029	25801.15525	20884.0434	9948.224568
0	0.979688684	3.846050296	0	9.053028472	3.4200442
2307.466334	1251.06245	1316.141488	739.1944511	732.0020165	930.9360312
461.1741152	361.5051245	387.6818698	421.794266	446.1849747	443.9217371
175.5334002	291.9472279	243.0703787	188.752934	222.4458425	162.7941039
156.384302	300.7644261	358.4518876	256.2400166	214.6861038	318.7481194
568.0899135	267.4550108	379.2205592	233.041332	331.0821841	274.2875448
12.76606547	2.939066053	1.538420118	7.381399655	9.053028472	2.73603536
6.383032736	2.939066053	2.307630178	0	12.93289782	3.4200442
31.91516368	29.39066053	35.38366272	27.41662729	60.78461974	35.56845968
60.63881099	681.8633243	257.6853698	330.0540131	457.8245827	196.9945459
309.5770877	223.36902	120.7659793	192.9708767	177.1807001	140.905821
0	28.41097185	19.23025148	6.32691399	2.586579563	36.25930861
0	0	0	1.054485665	1.293289782	0
400.5353042	114.6235761	137.6886006	402.813524	328.4956046	101.9173172
12.76606547	195.9377369	68.45969527	49.56082625	115.1027906	77.97700776
545.7492989	322.3175771	346.9137367	738.0661515	637.5918624	338.5843758
63.83032736	63.67976448	70.76732545	199.2977907	65.95777887	140.905821
114.7350134	125.3511672	125.3427792	82.26042672	104.3943512	157.3425535
311.1728459	30.37034921	8.461310651	49.56082625	78.89067669	3.4200442
255.3213094	153.8111234	191.5333047	228.8233893	223.7391322	104.6533525
432.4504679	261.5768787	236.1474882	147.6279931	263.8311155	270.1834918
67.02184373	129.3189063	93.07441717	94.90370985	112.516211	190.1544575
177.1291584	122.4610855	213.8403965	423.9032373	192.7001775	251.7152531
12.76606547	3.918754737	12.30736095	7.381399655	6.466448909	15.04819448
27.12788913	94.0501137	85.38231657	85.41333886	46.55843214	110.8094321
194.6824984	306.6425582	120.7659793	246.7496456	160.3679329	110.8094321
10.91498598	0	464.164426	2.10897133	37.66059844	253.6167977
300.0025386	295.8659827	412.2965917	515.6434902	353.0681104	406.3012509

30.3194055	103.8470005	398.4508107	50.61531192	94.41015407	88.23714036
0	16.65470763	3.846050296	3.163456995	5.173159127	13.6801768
119.6818638	100.9079345	109.9970385	117.0479088	98.29002341	51.98467184
2253.210556	777.8728154	869.2073669	899.4762722	2075.7301	554.0471604
6.383032736	0	3.846050296	0	1.293289782	4.10405304
1198.414396	824.8978722	650.7517101	744.4668795	689.3234537	733.2574764
279.2576822	368.8429928	446.2033712	278.647837	402.6787064	285.1154048
9.574549104	53.13831424	48.43715743	42.94920113	25.71060086	62.54576833
333.5134605	148.91268	83.0746864	322.6726135	217.2726833	112.8614586
3.191516368	5.878132106	1.538420118	0	3.879869345	0.68400884
4.787274552	11.75626421	6.922890533	5.272428325	5.173159127	6.15607956
411.7056115	162.6283216	16.15341124	0	0	6.15607956
796.2833338	651.4929751	726.903506	714.9412808	865.210864	800.9743516
212.2358385	55.84225501	50.76786391	118.1023945	106.0497621	110.8094321
3.191516368	4.898443422	1.538420118	18.56949256	14.29085209	0.68400884
1571.821811	2187.644832	1548.419849	1280.145597	1941.227962	1052.689605
1.595758184	0	0.769210059	1.054485665	5.173159127	3.4200442
362.2371078	318.3988224	317.6837545	380.6693251	248.3116381	162.1100951
113.0754249	252.4167895	205.0944781	210.8022293	102.8036048	149.8321364
323.9389113	473.1896345	738.4416569	741.3034225	472.0507703	945.9842257
14.36182366	10.77657553	1.538420118	30.58008428	15.51947738	5.47207072
6.383032736	1.959377369	1.538420118	3.163456995	2.586579563	5.47207072
0	0	0	1.054485665	0	0
0	9.796886843	1.538420118	0	6.466448909	1.36801768
209.0443221	304.6831808	306.9148136	185.589477	261.2445359	246.9271912
459.578357	766.1165511	624.5985681	725.4861375	456.531293	746.2536444
3.191516368	60.74069843	54.6139142	2.10897133	19.39934673	102.601326
1014.902205	291.9472279	303.8379734	532.5152608	698.3764821	156.6380244
0	0	0	0	0	0.608767868
165.9588511	124.4204629	89.99757693	156.0638784	143.5551658	121.0695647
0	0	0	0	0	0
7.97879092	0	14.61499113	5.272428325	3.879869345	8.20810608
110.1073147	181.2424066	142.303861	237.2592746	310.3895476	140.905821
23.93637276	8.817198159	21.52249746	18.93856254	16.81276716	16.41621216
114.8945892	409.50987	293.0690326	458.7012643	536.7152594	710.0011759
2358.530596	2405.13572	1669.185829	2761.697957	3812.618277	2142.315687
27.12788913	1.959377369	10.76894083	23.19868463	15.51947738	12.31215912

63.83032736	149.8923687	293.8382426	20.03522763	49.14501171	177.1582896
0	0	0	0	0	0
7.97879092	19.59377369	4.046044912	0	6.466448909	10.22593216
138.830962	300.7644261	155.380432	319.5091565	219.8592629	288.6517305
234.576453	77.39540606	177.6875237	125.4837941	100.876603	147.0619006
38.29819642	12.7359529	13.84578107	4.21794266	10.34631825	9.576123759
213.8315967	327.2160206	83.84389646	356.4161548	276.7640133	160.7420774
1.595758184	0	0.78459426	1.096665092	0	0.68400884
150.5119119	100.6728092	154.8112165	140.9003746	148.2239419	182.8834435
0	0	0	2.10897133	0	1.36801768
721.2826992	7966.828381	5044.479568	3379.626556	4556.259901	3170.380973
6.383032736	7.837509475	29.99919231	14.76279931	27.15908542	40.35652156
2266.870246	1794.554544	1118.692958	1854.355221	2258.342617	1634.028718
39.8939546	78.37509475	119.2275592	95.95819551	111.2229212	102.601326
11.17030729	5.878132106	7.692100592	17.9262563	11.63960804	17.78422984
31.91516368	13.71564158	16.15341124	26.36214162	11.63960804	6.8400884
63.83032736	144.9939253	245.3780089	79.08642487	161.6612227	292.7557835
362.2371078	169.4861424	203.0714556	130.7562225	225.032422	250.3472354
480.3232134	466.3318137	1123.046686	542.0056318	276.7640133	422.7174631
346.2795259	417.3473795	530.7549409	579.9671157	530.2488105	281.1276332
118.0861056	1787.118707	369.9900385	748.8957193	780.2158595	483.0128423
0	0	0.769210059	15.81728497	2.586579563	1.36801768
114.8945892	145.973614	751.5182279	394.3776387	166.8343818	572.515399
0	0	0	1.054485665	0	0.68400884
400.5353042	98.61546296	170.4107965	172.5560342	189.1436306	322.7632513
38.29819642	27.43128316	86.92073669	43.23391226	41.38527302	36.25246852
38.29819642	49.9641229	62.3060148	74.86848221	72.42422778	87.55313152
39.8939546	63.67976448	45.38339349	99.12165251	134.5021373	63.61282212
175.5334002	407.5504927	292.2998225	320.5636421	310.3895476	393.305083
54.25577826	301.7441148	1856.103873	175.0446204	236.6720301	1140.926745
11.17030729	13.71564158	4.615260355	18.98074197	19.39934673	10.94414144
9.574549104	675.9851922	82.30547634	689.6336249	278.0573031	33.51643316
1222.350769	835.6744477	760.7487486	776.1014494	812.1859829	618.3439913
70.2133601	75.43602869	155.380432	101.2306238	65.95777887	161.4260862
6.383032736	9.796886843	11.53815089	4.21794266	11.63960804	24.62431824
35.10668005	38.20785869	45.38339349	25.30765596	34.91882411	49.93264532
148.4055111	264.5159448	732.2879764	777.1559351	782.440318	442.5537195

157.9800602	112.6641987	129.9965	89.63128152	86.65041538	72.50493704
804.2621247	601.5288522	538.4470415	579.9671157	569.047504	359.104641
6.383032736	2.939066053	10.76894083	1.054485665	15.51947738	6.8400884
15.95758184	4.898443422	3.846050296	8.43588532	9.053028472	9.576123759
110.1073147	266.4753221	203.0714556	332.1629845	173.3008308	174.4222542
0	0	0.769210059	1.054485665	0	0
0	0	0.769210059	1.054485665	1.293289782	0.68400884
7.97879092	4.898443422	3.076840237	10.54485665	0	8.89211492
0	1.959377369	11.53815089	4.21794266	2.586579563	4.10405304
1246.287142	1628.242593	843.0542249	1648.161094	474.6373499	1008.913039
552.1323317	192.9986708	406.1429113	629.527942	375.0540367	351.5805437
1303.734436	2486.449881	2093.020571	1467.844046	2220.578555	1422.738387
429.2589515	454.5755495	258.4545799	314.2367282	266.417695	227.0909349
232.9806949	353.667615	186.1488343	383.832782	345.3083717	409.0372863
17.55334002	16.65470763	1.538420118	737.1487489	21.98592629	1.36801768
4549.506583	3369.149385	2693.773627	3100.187855	2404.225704	2736.719369
12.76606547	42.12661343	39.99892308	2.10897133	10.34631825	30.7803978
250.5340349	972.8308635	723.0574557	734.9765085	699.6697719	380.308915
39.8939546	33.30941527	566.9078136	9.490370985	19.39934673	289.3357393
517.0256516	363.4645019	522.2936302	570.4767447	609.1394872	428.1895338
67.27716504	0	771.9253707	3.163456995	351.6196259	242.2896113
1.595758184	7.837509475	7.692100592	25.30765596	18.10605694	32.14841548
105.3200401	125.4001516	89.22836687	83.30436753	208.2196549	93.70921107
1247.8829	1829.078774	1373.039956	2652.031447	2043.397855	1562.960199
502.663828	519.2350027	335.3755858	392.2686674	481.1037988	500.010462
11.17030729	31.3500379	22.30709172	15.81728497	23.27921607	26.67634476
1750.546728	1734.048971	1376.116796	1031.28698	1297.169651	1010.965065
981.5349014	3121.209773	2202.040713	2344.733235	5561.9867	5379.647445
15561.83381	5922.218097	9031.295305	3144.476253	4213.538109	5831.175361
28.72364731	102.8673119	146.9191213	172.9356491	99.58331319	183.3143691
82.97942557	61.72038711	96.1512574	14.76279931	37.50540367	75.92498124
1758.302113	1670.908036	1850.119419	1169.498416	1944.978503	2181.953999
563.1909359	1033.885062	1318.141434	2024.38049	1210.00192	1152.17185
81.38366738	90.13135896	59.99838462	33.74354128	45.26514236	132.0137061
1229.068911	799.2790131	481.0255105	1495.028686	690.5132803	188.1161112
300.0025386	265.4956335	342.2984764	327.9450418	303.9230987	233.9310233
724.4742155	495.7224743	476.9102367	420.7397803	275.4707235	424.7694896

22.34061458	0	3.846050296	4.21794266	7.75973869	0
11.17030729	31.3500379	23.84551184	7.381399655	16.81276716	20.5202652
95.74549104	147.9329913	251.0240107	368.4056568	210.7545028	209.9086328
71.84103344	35.27858952	23.07630178	13.26542967	64.66448909	25.30832708
1.595758184	3.918754737	14.61499113	20.03522763	5.173159127	30.09638896
523.4086843	1289.270309	638.4443492	786.6463061	491.4501171	1049.953569
35.10668005	64.65945317	69.99811539	40.07045527	25.86579563	65.66484864
826.6027393	919.9276746	616.1372574	1227.421314	888.4900801	621.0800267
1597.353942	2990.009865	2723.77282	3274.17799	2472.770063	1664.193508
0	0	13.07657101	0	0	17.100221
73.40487646	93.07042501	146.1499113	161.3363067	195.286757	209.306705
142.0224784	95.02980238	123.0736095	147.6279931	103.4631825	107.3893879
43.08547097	107.7657553	66.92127515	62.21465423	42.6785628	68.400884
6910.366985	5172.570112	18070.70578	11933.32956	9949.549882	4394.941479
2360.126354	13.71564158	203.8406657	594.729915	85.3571256	5.47207072
124.4691384	129.3189063	98.45888758	152.9004214	203.0464957	149.797936
269.6831331	410.4895587	309.2224438	144.4645361	345.3083717	309.8560045
38.29819642	307.6222469	0	136.0286508	684.1502945	0.68400884
17.55334002	4.898443422	6.922890533	7.381399655	2.586579563	6.15607956
172.3418839	170.4658311	237.6859083	195.079848	120.2759497	268.8154741
5538.876657	4479.136665	1746.876045	4854.852001	3612.15836	2971.334401
59.04305281	73.47665132	96.92046746	54.83325458	47.85172192	74.55696356
9.574549104	88.17198159	6.153680474	205.6247047	191.4068877	27.3603536
89.3624583	94.0501137	148.4575414	74.86848221	175.8874103	150.4819448
122.8733802	272.3534542	179.9951539	197.1888193	186.2337286	295.4918189
3.191516368	9.796886843	2.307630178	12.65382798	3.879869345	2.73603536
33.51092186	165.5673877	176.1491036	114.9389375	139.6752964	246.9271912
183.5121912	465.3521251	210.7635562	582.0760871	276.7640133	398.7771537
0	0	6.153680474	1.054485665	1.293289782	7.52409724
98.93700741	110.7048213	179.2259438	172.9356491	120.2759497	183.9983779
789.9003011	1484.228357	840.7465947	650.6176553	1317.862288	7024.770786
151.5970275	114.6235761	76.15179586	121.2658515	3.879869345	83.44907848
132.4479293	119.5220195	189.2256746	156.0638784	170.7142512	158.006042
1.595758184	16.65470763	24.6147219	2.10897133	10.34631825	30.09638896
66.23992222	217.1675907	134.5348394	101.5680592	175.2019667	129.6401954
1488.842386	2832.279986	653.0593403	3318.466388	1687.743165	706.5811317
312.7686041	863.1057309	383.8358196	557.8229168	296.16336	598.507735

0	0	0	0	0	0
78.19215101	46.04536816	36.15287278	59.05119724	51.73159127	68.400884
35.10668005	60.74069843	129.2272899	31.63456995	76.30409712	49.24863648
22.34061458	38.20785869	71.53653551	32.68905561	46.55843214	90.28916688
287.2364731	505.5193611	500.7557486	654.8355979	324.6157352	480.1742057
6.383032736	45.06567948	35.38366272	12.65382798	29.74566498	32.83242432
210.6400803	79.35478343	106.9201982	76.97745354	103.4631825	90.28916688
12.76606547	274.3128316	233.839858	297.3649575	547.0615777	730.5214411
19.14909821	122.4610855	41.5373432	70.65053955	71.130938	25.99233592
39.8939546	19.59377369	55.38312426	45.34288359	94.41015407	49.24863648
0	0	0	0	0	2.05202652
22.78742687	0.979688684	9.99973077	14.45699847	16.47651182	23.22210012
1034.051303	545.6865972	276.1464113	1043.940808	1043.684854	264.0274122
0	2.939066053	4.615260355	3.163456995	0	4.78806188
0	0	0.399989231	0	0	2.73603536
24.82999734	19.41742972	50.42941148	20.17231077	16.91623035	7.69509945
23.93637276	22.53283974	21.53788166	15.81728497	23.27921607	21.88828288
0	19.59377369	8.461310651	0	0	62.24480444
57.44729462	49.9641229	34.61445267	20.03522763	59.49132996	56.08872488
0	0.979688684	0	1.054485665	2.586579563	2.05202652
504.2595861	235.1252842	382.2973994	184.5349914	412.5594404	314.6440664
49.4685037	83.27353817	87.68994675	141.3010791	188.8203081	85.501105
70.2133601	8.817198159	9.230520711	4.21794266	10.34631825	4.78806188
3797.904478	4420.355344	2116.096873	2449.5702	3111.655215	2532.884734
306.3855713	96.00949106	47.69102367	133.9196795	629.8321237	45.82859228
137.2352038	522.1740687	323.0682249	409.140438	25.86579563	272.9195271
143.6182366	72.49696264	37.6912929	45.34288359	63.37119931	23.25630056
4.787274552	9.796886843	6.153680474	11.59934231	12.93289782	17.100221
84.57518375	93.07042501	163.0725326	69.59605389	82.77054603	168.2661746
54.25577826	6.85782079	43.07576332	15.81728497	7.75973869	18.46823868
84.57518375	4428.192853	4696.796622	3860.472019	8031.329545	6922.853469
89.3624583	185.1611613	154.6112219	153.9549071	131.9155577	281.1276332
229.7891785	407.5504927	390.7587101	434.448094	359.5345593	511.6386123
202.6612894	358.5660585	253.0701095	627.4189707	256.0713768	324.904199
31.91516368	37.22817	29.99919231	40.07045527	36.21211389	43.77656576
686.1760191	447.7177287	502.2941687	333.2174701	658.2844989	386.4649946
11.17030729	20.57346237	3.846050296	15.81728497	15.51947738	12.31215912

159.5758184	217.4908879	164.6109527	210.897133	290.9902009	233.2470144
0	0	0	1.054485665	0	0
0	0	0	0	0	0
0	0	0	0	0	0.68400884
0	0	0	0	0	0.68400884
170.7461257	251.7799919	189.9948846	256.2400166	240.5518994	248.9792177
54.25577826	115.6032648	93.84362723	88.57679586	102.1698928	90.97317571
0	0.979688684	0	3.163456995	0	1.36801768
1.595758184	1.959377369	0.769210059	3.163456995	3.879869345	1.36801768
103.724282	156.7501895	139.9962308	128.6472511	124.155819	164.1621216
86.17094193	170.4658311	190.7640947	158.1728497	151.3149045	145.0098741
202.6612894	102.8673119	77.69021598	95.95819551	122.8625293	88.9211492
38.29819642	40.16723606	56.92154438	67.48708256	37.50540367	42.40854808
59.04305281	28.41097185	17.69183136	167.6632207	14.2261876	35.56845968
84.57518375	42.12661343	10.76894083	153.5647474	20.69263651	20.44502423
525.0044425	6.85782079	33.84524261	15.81728497	84.06383581	0
694.15481	189.0799161	316.1453343	258.3489879	332.3754739	205.202652
12.76606547	33.30941527	67.69048521	20.03522763	34.91882411	199.0465724
90.95821649	35.26879264	36.92208284	33.74354128	45.26514236	24.62431824
563.3026389	404.6114266	228.4553876	303.6918715	196.5800468	110.1254232
0	0	1.569188521	0	0	0.861851138
180.3206748	855.2486276	428.450003	484.0089202	605.2596179	876.8993328
9.574549104	5.878132106	3.846050296	2.10897133	16.81276716	4.78806188
494.685037	143.0345479	516.1399497	286.8201009	473.3440601	645.7043449
550.5365735	192.0189821	198.4561953	195.079848	378.9339061	132.697715
0	0.979688684	3.076840237	3.163456995	1.293289782	2.73603536
87.76670012	77.39540606	86.15152663	105.4485665	53.02488105	158.006042
595.2178026	3542.554283	1821.48942	1917.054939	1714.902251	1393.326007
0	0	13.84578107	1.054485665	2.586579563	2.73603536
54.25577826	73.47665132	87.68994675	61.16016857	71.130938	102.601326
6.383032736	3.918754737	1.538420118	9.490370985	7.75973869	7.52409724
3651.094725	2506.043655	2153.788166	2398.954888	5724.100574	922.7279251
0	0	0	0	0	0
856.9221448	414.4083135	693.8274734	529.3518038	623.3656748	573.1994079
411.7056115	607.4069843	818.439503	231.9868463	385.400355	718.209282
1378.735071	777.8728154	1450.730172	430.2301513	796.6665056	1199.751505
20.74485639	29.39066053	29.99919231	23.19868463	27.15908542	39.67251272

225.0019039	98.94855712	174.6106834	141.3010791	192.7001775	128.5936619
201.0655312	57.80163238	94.61283728	179.262563	117.6893701	136.1177592
28.72364731	1.959377369	28.46077219	33.74354128	14.2261876	10.2601326
0	2.939066053	11.53815089	8.43588532	5.173159127	15.73220332
0	2.939066053	0.769210059	0	0	2.05202652
269.6831331	387.956719	494.6020681	245.6951599	283.2304622	252.3992619
737.240281	552.544418	533.8317811	484.0089202	404.7997017	424.0854808
1265.43624	587.8132106	712.2885148	683.3067109	713.8959595	610.1358852
0	11.75626421	6.153680474	0	3.879869345	20.5202652
0	0	0	0	0	0
0	0	0	0	0	0
2963.322948	1498.923687	1602.264553	4703.006066	9580.690703	935.0400842
60.57498066	0	40.70659633	0	0	55.86300196
0	0	0	0	0	0.68400884
322.3431532	275.2925203	200.7638255	377.5058681	181.0605694	240.7711117
46.27698734	0	5.384470415	1.054485665	7.75973869	6.15607956
240.9594858	250.8003032	207.686716	248.8586169	292.2834907	383.7289592
258.5128258	272.3534542	192.3025148	144.4645361	237.9653198	326.9562255
148.4055111	228.2674634	299.222713	199.2977907	162.9545125	298.2278542
1.595758184	0	0	0	0	2.05202652
268.0873749	535.8897103	230.7630178	105.4485665	58.19804018	588.9316112
0	0	6.922890533	1.054485665	2.586579563	2.73603536
22.34061458	120.5017082	157.6880621	136.0286508	86.65041538	210.6747227
17.55334002	37.22817	129.2272899	206.6791903	113.8095008	350.2125261
11.17030729	1.959377369	9.230520711	0	2.586579563	9.576123759
0	0	0	0	0	0
202.6612894	720.071183	203.8406657	683.3067109	499.2098558	185.3663956
0	0	0.769210059	0	2.586579563	0
194.0920679	61.24033966	160.2726079	124.3976739	187.2036959	74.61852435
5441.455619	3009.495873	9971.93921	2347.095283	3715.621543	6978.661751
111.7030729	245.9018598	136.9193905	128.6472511	96.99673363	178.5263072
0	0	0	0	0	0
0	0	0	0	0	0
0	0	0	0	0	0
0	0.979688684	33.84524261	4.21794266	12.93289782	2.05202652
159.5758184	1103.129459	366.9131983	613.710657	581.9804018	629.9721416
92.55397467	204.754935	115.3815089	115.9934231	157.7813534	153.2179802

87.76670012	366.4035679	232.3014379	233.041332	157.7813534	366.6287382
386.1734805	194.9580482	122.3043994	179.262563	175.8874103	142.2738387
173.9376421	14.69533026	55.38312426	73.81399655	2.586579563	103.2853348
0	0	0.769210059	2.10897133	0	0
1134.584069	443.798974	492.2944379	291.0380435	615.6059361	601.9277792
6.383032736	12.7359529	66.15206509	2.10897133	0	6.15607956
8243.686778	9077.795349	4296.038181	7364.527884	9382.817367	3051.363435
4.787274552	1.959377369	1.538420118	2.10897133	0	1.36801768
690.9632937	763.1774851	613.8296273	681.1977396	852.2779662	841.3308732
0	0	0	0	0	0
0	0	0	0	0	0
0	0	0	0	0	0
0	0	0	0	0	0
0	0	0.769210059	0	0	0
19.14909821	61.72038711	47.69102367	115.9934231	50.43830149	86.18511384
3.797904478	13.12782837	7.092116746	15.15295901	5.056763047	17.18914215
0	8.817198159	11.53815089	1.054485665	7.75973869	19.83625636
1185.823864	1062.325425	465.541312	1394.620561	771.3568245	242.4332531
102.1285238	222.3893313	100.7665178	152.9004214	94.41015407	43.77656576
1.595758184	4.898443422	6.922890533	3.163456995	2.586579563	7.52409724
41.48971278	49.9641229	66.15206509	54.83325458	43.97185258	60.87678676
3654.286241	1684.084848	279.9924616	2817.585697	3230.637875	911.7837837
7.97879092	8.817198159	3.076840237	5.272428325	3.879869345	9.576123759
4.787274552	4.898443422	7.692100592	1.054485665	2.586579563	22.57229172
457.0889742	38.18826492	172.8722687	42.90702171	152.8409864	174.2170515
1.595758184	9.796886843	26.15314201	1.054485665	1.293289782	102.601326
1792.036441	413.4286248	423.8347426	501.9351765	505.6763047	231.1949879
3461.199501	890.5370141	1323.041302	1473.116474	2197.299339	1327.661158
14.36182366	15.67501895	16.15341124	17.9262563	15.51947738	29.41238012
60.63881099	335.05353	487.6791775	15.81728497	191.4068877	548.5750896
114.8945892	70.53758527	130.7657101	163.4452781	153.901484	93.70921107
0	0	0	0	0	0
0	0	0	0	0	0
697.3463264	954.2167785	711.5193048	962.7454121	1079.896968	1433.682529
1160.1162	710.2742961	729.9803462	694.9060532	712.6026697	584.8275582
0	0	1.538420118	0	1.293289782	0
113.2988311	101.8876232	149.9959615	90.68576719	60.78461974	112.1774498

9.574549104	0.979688684	3.846050296	3.163456995	3.879869345	7.52409724
0	0	0	0	0	0
0	0	0	0	0	0
0	0	0	0	0	0
0	0	0	0	0	0
0	0	0	0	0	0
0	0	0	0	0	0
0	0	0	0	0	0
2.010655312	0.979688684	0	3.943776387	0	10.98518197
0	7.837509475	455.3723551	25.30765596	6.466448909	16.41621216
525.0044425	563.3209935	226.9169675	429.1756656	287.1103315	144.3258652
150.0012693	96.00949106	86.15152663	18.98074197	239.2586096	35.56845968
25.53213094	21.55315106	22.30709172	29.52559862	40.09198323	48.56462764
59.04305281	114.6235761	62.3060148	60.1056829	69.83764821	129.2776708
153.1927857	3.918754737	3.846050296	1.054485665	1.293289782	375.5208531
55.85153644	50.94381159	109.2278284	117.0479088	129.3289782	108.7574056
1.595758184	0.979688684	1.538420118	1.054485665	1.293289782	2.73603536
0	0	0	0	0	0
694.15481	285.0894071	425.3731628	635.854856	300.0432294	585.511567
9.574549104	41.14692474	27.69156213	1.054485665	46.55843214	51.98467184
552.1323317	801.3853438	403.066071	891.0403869	671.2173967	366.6287382
0	1.959377369	0	2.10897133	5.173159127	0.68400884
14.36182366	31.3500379	172.3030533	163.4452781	93.11686429	175.106263
46.27698734	38.20785869	38.46050296	95.95819551	51.73159127	57.45674256
73.40487646	56.82194369	88.45915681	70.65053955	72.42422778	73.18894588
31.91516368	94.0501137	137.6886006	140.2465934	104.7564723	274.9715537
1.595758184	0	0	0	1.293289782	0
4.787274552	15.67501895	47.69102367	2.10897133	9.053028472	113.5454674
1078.732532	203.7752463	655.3669705	914.2390715	543.1817083	537.6309482
256.9170676	510.4178045	478.4486568	589.4574867	923.4089042	733.2574764
0	0	0	0	0	0
0	0	0	0	0	0
0	0	0	0	0	0
0	0	0	0	0	0
57.44729462	80.33447211	66.92127515	67.48708256	73.71751756	270.8675006
204.2251324	89.15167027	127.627333	112.8299662	124.1299533	64.29683096
5.02663828	1.410751705	1.246120296	1.571183641	2.418451892	1.108094321

486.7062461	18.614085	25.38393195	18.98074197	16.81276716	45.82859228
1335.6496	573.1178803	581.5228048	610.5472	1090.243286	894.6835627
4.787274552	19.59377369	74.61337574	97.01268118	24.57250585	32.14841548
39.8939546	73.47665132	62.3060148	64.32362556	37.50540367	153.2179802
25.53213094	360.5254358	229.2245976	524.0793755	347.8949513	521.2147361
134.0436875	52.90318895	45.38339349	66.43259689	96.99673363	26.67634476
89.3624583	101.8876232	97.68967752	66.43259689	77.5973869	171.6862188
0	0.979688684	1.538420118	0	0	8.89211492
561.7068808	326.2363319	423.8347426	707.5598812	699.6697719	612.1879118
78.19215101	88.17198159	93.07441717	234.0958176	69.83764821	82.76506964
35.10668005	113.6438874	290.7614024	222.4964753	316.8559965	271.5515095
0	13.71564158	13.84578107	8.43588532	10.34631825	16.41621216
2265.976621	6702.050289	2096.866621	2117.407215	3548.787161	4904.343383
285.6407149	383.0582756	349.2213669	454.4833216	307.8029681	142.9578476
9.574549104	21.55315106	15.38420118	26.36214162	12.93289782	35.56845968
30.3194055	27.70559599	51.58322657	49.01249371	83.98623843	134.2230547
62.23456918	79.35478343	59.99838462	63.2691399	140.9685862	108.7574056
1964.378324	758.2790417	1349.963654	1018.633152	1454.951004	678.5367692
157.9800602	78.37509475	222.3017071	102.2851095	87.94370516	201.7826078
31.91516368	102.8673119	93.07441717	139.1921078	116.3960804	201.0985989
472.3444225	932.6636275	809.9781924	567.3132877	715.1892493	805.7624135
5305.895962	1692.902047	1182.275861	2240.782038	3976.866079	993.8648445
368.6201405	276.272209	379.2205592	295.2559862	375.0540367	422.7174631
1.595758184	60.74069843	163.8417426	10.54485665	50.43830149	56.77273372
108.5115565	177.3236519	189.9948846	182.42602	117.6893701	193.5745017
935.1142958	511.3974932	694.5966835	432.3391226	638.8851522	605.3478234
22.34061458	70.53758527	58.4599645	91.74025285	99.58331319	106.705379
255.3213094	90.13135896	79.99784616	63.2691399	159.0746432	76.60899008
172.3418839	111.68451	57.69075444	101.2306238	94.41015407	64.29683096
1.595758184	0	1.538420118	1.054485665	0	2.73603536
3448.433436	1804.586557	1887.641485	1668.196322	1667.580777	1290.040672
483.5147297	382.0785869	625.3677782	349.0347551	468.170901	315.3280752
1319.692018	1231.468676	1093.047494	1323.37951	1126.4554	1157.342957
422.8759188	162.6283216	173.8414734	207.733676	369.8808776	227.7749437
1.659588511	0	0.899975769	0	2.651244053	1.463778918
1.595758184	0	7.692100592	0	29.74566498	2.73603536
87.76670012	549.6053519	503.8325888	272.0573016	290.9902009	782.5061129

1.595758184	0	3.846050296	2.17224047	2.586579563	15.04819448
397.3437878	77.39540606	546.139142	166.6087351	85.3571256	193.5745017
20.74485639	17.63439632	36.92208284	59.05119724	15.51947738	28.04436244
4.787274552	1.959377369	2.307630178	1.054485665	2.586579563	15.73220332
14.36182366	38.20785869	158.4572722	35.85251261	18.10605694	93.02520223
0	5.907522767	0.769210059	18.13715344	20.83489838	1.374857768
6.383032736	6.85782079	10.76894083	10.54485665	11.63960804	17.100221
164.3630929	245.9018598	258.4545799	582.0760871	349.1882411	381.6769327
3509.072247	5954.547823	4502.186477	1972.942679	3543.614002	4123.889296
756.3893792	2080.858766	1095.355124	1729.356491	1700.676063	1305.088867
118.0861056	431.0630211	245.3780089	221.4419896	106.0497621	176.4742807
68.61760191	55.84225501	148.4575414	158.1728497	783.7336077	353.6325703
1.659588511	0	0.807670562	0	1.345021373	1.436418564
162.7673348	327.2160206	211.5327663	625.3099993	609.1394872	113.5454674
0	0.979688684	2.307630178	1.054485665	0	2.73603536
381.386206	635.8179561	654.5977604	790.8642487	649.2314704	569.7793637
41.48971278	46.04536816	55.38312426	64.32362556	59.49132996	64.9808398
231.3849367	699.4977206	213.8403965	399.650067	725.5355676	112.1774498
274.4704076	1302.98595	1519.959077	694.9060532	825.1188808	1195.647452
0	0	0	0	0	0.68400884
641.49479	1350.011007	756.9026983	1029.178009	1345.021373	972.6605704
43.08547097	29.39066053	25.38393195	31.63456995	29.74566498	11.62815028
4.34046226	3.526879264	0	4.007045527	0	5.950876908
55.85153644	44.08599079	22.30709172	24.25317029	27.15908542	26.67634476
552.1323317	126.3798403	299.9919231	207.733676	111.2229212	369.3647736
25.53213094	7.837509475	12.30736095	41.12494093	14.2261876	35.56845968
307.9813295	215.5315106	27.69156213	36.90699827	1.293289782	119.0175382
57.44729462	115.6032648	127.6888698	102.2851095	98.29002341	164.8461304
35.10668005	110.7048213	183.0719941	106.5030522	230.2055812	206.5706697
19.14909821	19.59377369	20.7686716	48.50634059	25.86579563	51.300663
22.34061458	5.878132106	13.07657101	5.272428325	1.293289782	14.36418564
31.91516368	60.74069843	14.61499113	4.21794266	18.10605694	67.71687516
523.4086843	349.7488603	448.4494645	347.9802694	413.8527302	317.3801017
0	0	1.538420118	0	1.293289782	4.10405304
11.17030729	19.59377369	20.7686716	13.70831364	20.69263651	21.88828288
19.14909821	106.7860666	89.22836687	89.63128152	63.37119931	99.86529063
4.787274552	0	0	5.272428325	1.293289782	1.36801768

170.7461257	138.1361045	139.9962308	263.6214162	245.7250585	164.8461304
56.07494259	109.6271638	97.61275652	134.1411214	84.81394389	101.212788
0	0	0.769210059	0	0	2.05202652
75.00063465	44.08599079	56.15233432	51.66979758	55.61146062	85.501105
0	0.979688684	0.769210059	0	0	0.68400884
18371.96397	12106.99276	15655.73234	7142.031409	8161.951813	5438.554287
264.8958585	818.0400514	858.4384261	269.9483302	1400.632834	477.4381703
0	0.979688684	0.769210059	2.10897133	1.293289782	0
28.72364731	43.10630211	40.76813314	51.66979758	54.31817083	42.40854808
95.74549104	1116.8451	7062.117554	302.6373858	143.5551658	25888.36657
167.5546093	541.7678424	173.0722633	807.7360194	448.7715543	311.2240222
0	0.979688684	0	0	1.293289782	1.36801768
3.191516368	18.614085	29.22998225	28.47111295	6.466448909	32.83242432
113.2988311	70.53758527	93.84362723	103.3395952	73.71751756	73.18894588
126.0648965	284.1587029	270.8234777	342.7289308	209.5258775	200.4214302
489.8977625	410.4895587	473.0641864	342.7078411	496.6232762	524.6347803
31.91516368	4.898443422	1.538420118	0	0	14.36418564
0	12.7359529	37.6912929	7.381399655	6.466448909	38.98850388
157.9800602	182.2220953	204.6098758	254.1310453	169.4209614	144.3258652
22.34061458	4.898443422	19.23025148	105.4485665	42.6785628	45.82859228
28.72364731	96.00949106	145.3807012	112.8299662	63.37119931	127.2256442
67.02184373	126.3798403	109.9970385	127.5927655	99.58331319	116.9655116
0	11.75626421	2.307630178	3.163456995	1.293289782	6.8400884
0	0.989485571	0	0	0	0
47.87274552	108.745444	39.22971302	99.12165251	55.61146062	63.61282212
9.574549104	26.45159448	18.46104142	53.77876891	29.74566498	12.99616796
14.36182366	162.6283216	1444.576491	231.9868463	285.8170418	583.4595405
55.85153644	59.76100974	60.76759468	85.41333886	89.23699494	58.82476024
0	14.69533026	33.84524261	5.272428325	16.81276716	23.9403094
3.191516368	1.959377369	88.45915681	76.97745354	7.75973869	11.62815028
0	0	1.538420118	0	0	1.36801768
0	106.7860666	2.307630178	0	1.293289782	38.30449504
315.9601204	381.0988982	474.6026065	734.9765085	437.1319462	783.1901218
113.2988311	57.80163238	74.61337574	98.06716684	139.6752964	75.2409724
406.9183369	895.4354575	496.9096983	827.771247	318.1492863	442.5537195
1.595758184	9.796886843	6.922890533	2.10897133	0	3.4200442
154.7885438	196.9174256	217.6864468	534.6242321	210.8062344	238.0350763

67.02184373	84.25322685	196.9177752	51.66979758	103.4631825	127.2256442
258.5128258	337.9925961	323.0682249	366.9610114	565.1676346	448.0257902
36.06413496	81.66684873	15.70726941	62.86843535	48.95101824	12.02487541
314.3643622	578.9960124	841.5158048	501.9351765	369.8808776	586.1955758
31.91516368	93.07042501	83.84389646	111.7754805	73.71751756	90.28916688
9.574549104	2.939066053	3.846050296	0	2.586579563	6.8400884
12.76606547	38.20785869	16.15341124	52.72428325	49.14501171	32.14841548
540.9620244	327.2160206	307.6840237	203.5157333	397.039963	324.904199
540.9620244	666.1883053	555.3696628	926.8928995	962.2075976	456.9179051
0	0	1.538420118	1.054485665	0	3.4200442
307.9813295	368.3629453	254.6085296	575.7491731	521.195782	478.1221791
0	0	0	0	0	0
11.17030729	2.939066053	1.538420118	1.054485665	5.173159127	0.68400884
4029.289415	925.8058067	990.7425563	1071.357436	1709.729091	648.4403803
2163.848097	241.983105	400.7584409	284.7111295	757.8678121	508.9025769
292.0237477	262.5565674	136.1501805	231.9868463	305.2163885	152.5339713
20.74485639	21.55315106	19.99946154	27.41662729	32.33224454	23.9403094
1238.308351	827.8369383	704.5964143	1472.061988	1223.452134	1102.62225
0	0.979688684	0	0	1.293289782	0
369.402062	2293.774507	2397.704676	1222.865936	1238.363765	3319.816384
67.02184373	145.973614	93.84362723	377.5058681	96.99673363	227.0909349
603.1965935	751.4212209	266.1466805	374.3424111	407.3862813	353.6325703
202.6612894	93.07042501	189.2256746	151.8459358	183.647149	140.2218122
90.95821649	121.4813969	74.61337574	90.68576719	64.66448909	54.7207072
0	0.979688684	1.538420118	0	1.293289782	0.68400884
35.10668005	171.4455198	116.919929	226.714418	169.4209614	229.8269702
41.48971278	3.918754737	9.99973077	1.054485665	19.39934673	4.10405304
81.38366738	166.5470763	269.9927308	167.6632207	156.4880636	97.81326411
1.595758184	0	2.307630178	0	0	0
662.2396464	331.1347753	505.3710089	316.3456995	424.1990484	320.8001459
54.25577826	68.5782079	66.92127515	78.03193921	64.66448909	115.597494
815.432432	803.3447211	1271.504228	753.9572504	1011.352609	1263.364327
6.383032736	79.35478343	95.38204734	29.52559862	23.27921607	79.34502544
14.36182366	114.6235761	7.692100592	7.381399655	188.8203081	14.36418564
52.66002007	163.6080103	109.2278284	133.9196795	175.8874103	172.3702277
584.0474953	176.3439632	36.15287278	278.3842156	324.6157352	208.6226962
89.3624583	138.1361045	148.4575414	67.48708256	112.516211	119.701547

694.15481	402.6520493	453.8339349	583.1305727	465.5843214	328.3242432
82.97942557	223.36902	273.8387811	244.6406743	269.0042746	245.5591735
4.787274552	0	0	0	0	5.47207072
39.8939546	214.5518219	119.2275592	84.3588532	137.0887169	157.3220332
23.93637276	14.69533026	64.61364497	81.1953962	40.09198323	121.7535735
0	0.979688684	0.769210059	2.10897133	1.293289782	0
1.595758184	26.45159448	9.99973077	5.272428325	20.69263651	23.25630056
611.1753845	420.2864456	794.5939912	563.0953451	259.9512461	352.2645526
0	1.959377369	4.615260355	2.10897133	0	34.88445084
1.595758184	2.939066053	3.076840237	0	3.879869345	4.78806188
102.6232088	93.09981567	123.8735879	12.65382798	161.9328136	175.1883441
1297.351404	555.483484	2498.394272	507.2076048	6492.314704	470.5980819
0	0.979688684	39.22971302	1.054485665	5.173159127	9.576123759
134.0436875	48.98443422	93.07441717	108.6120235	38.79869345	109.4414144
628.7287245	902.2932783	486.1407574	1691.395007	6419.890477	1060.213702
3420.459795	208.6932835	863.2536811	297.6918481	753.7551506	998.7760279
51.06426189	6.85782079	9.230520711	20.03522763	34.91882411	38.98850388
189.8952239	157.7298782	252.3008994	587.3485154	236.6720301	461.705967
177.1291584	222.3893313	276.9156213	88.57679586	54.31817083	293.4397923
0	0	0.769210059	0	0	0.68400884
27.12788913	33.30941527	26.92235207	21.0897133	12.93289782	36.93647736
652.904461	637.0523639	712.911575	796.4002985	718.7199304	804.2781143
36.70243823	95.02980238	148.4575414	91.74025285	128.0356884	125.8576266
1.595758184	2.939066053	4.615260355	4.21794266	2.586579563	0.68400884
3277.68731	2916.533213	2382.243553	4679.807381	3635.437576	1329.713185
31.91516368	1.959377369	16.15341124	30.58008428	42.6785628	6.8400884
14.36182366	11.75626421	15.38420118	17.9262563	16.81276716	19.83625636
225.0019039	515.316248	452.2955148	422.8487516	358.2412695	515.0586565
1115.434971	19.59377369	0.769210059	0	0	122.4375824
1.595758184	0	0.38460503	0	0.646644891	0.68400884
1.595758184	0	0.38460503	0	0.646644891	0.68400884
165.9588511	177.3236519	223.8401272	126.5382798	128.0356884	462.3899758
253.7255513	291.937431	305.3763935	190.7986362	1136.788785	501.2621982
44.68122915	62.7000758	159.2264823	267.8393589	9.053028472	88.23714036
272.8746495	179.2830292	286.146142	344.8168124	460.4111623	241.4551205
7.97879092	8.817198159	4.615260355	1.054485665	0	8.20810608
1113.839212	1047.287204	879.9763078	806.6815337	759.1611019	554.0471604

0	0	0	0.991216525	0	0
0	0	0	1.054485665	0	0
0	0	0	1.054485665	0	0
0	0	0	0	0	0
0	0	0	0	0	0
0	0	0	0	0	0
0	0	0	0	0	0
0	0	0	0	0	0
0	0.979688684	0	0	0	0
0	0	0	0	0	0
0	0	0	0	0	0
0	0	0	0	0	0
0	0	0	0	0	0
0	0	0	0	0	0
0	0	0	0	0	0.68400884
0	0	0	0	0	0.68400884
0	0	0	0	0	0.68400884
191.4909821	372.2817	189.2256746	353.2526978	228.9122914	168.9501835
11.17030729	28.41097185	26.15314201	12.65382798	18.10605694	19.83625636
86.17094193	182.2220953	189.9948846	88.57679586	138.3820066	143.6418564
740.4317974	711.2539848	543.0623018	429.1756656	253.4847972	412.4573305
9.574549104	18.614085	26.92235207	10.54485665	10.34631825	30.7803978
2650.554344	8546.804082	3520.674441	1927.599796	7374.338336	3122.500354
113.2988311	163.6080103	196.1485651	328.9995275	284.523752	165.5301393
142.0224784	176.3439632	230.7630178	254.1310453	204.3397855	261.9753857
6.383032736	14.02914196	48.43715743	32.1090885	14.66590613	17.46274568
12.76606547	43.10630211	9.99973077	33.74354128	19.39934673	19.15224752
17.4895097	2.292471521	45.33724089	31.37094853	7.242422778	5.260027979
1.595758184	5.878132106	61.53680474	281.5476725	34.91882411	112.1774498
20.74485639	24.49221711	0	8.43588532	14.2261876	0
0	0.979688684	0	0	0	0.68400884
0	0	0	0	2.586579563	0.68400884
86.17094193	37.22817	46.15260355	41.12494093	46.55843214	41.0405304
1.595758184	4.898443422	10.76894083	11.59934231	29.74566498	4.10405304
4.787274552	22.53283974	31.53761243	31.63456995	31.03895476	39.67251272
119.6818638	277.2518977	327.6834852	282.6021582	172.007541	294.1238012
49.4685037	75.43602869	32.30682249	147.6279931	85.3571256	49.93264532

7.97879092	17.63439632	5.384470415	29.52559862	12.93289782	15.73220332
282.4491986	149.8923687	605.3683166	315.2912138	261.2445359	243.507147
414.8971278	457.5146156	547.6775622	878.3865589	459.1178725	632.0241681
191.4909821	445.7583514	279.9924616	431.284637	415.1460199	383.7289592
0	0	0	0	0	0
4873.317833	1117.021444	1523.643593	1613.531785	4198.936867	1427.109204
1.595758184	1.959377369	1.538420118	2.10897133	3.879869345	1.36801768
73.40487646	241.983105	246.147219	220.387504	230.2055812	211.3587315
9.574549104	324.2769545	158.4572722	310.0187855	234.0854505	240.0871028
272.8746495	152.8314348	130.7657101	39.0159696	169.4209614	57.45674256
3.191516368	41.14692474	37.6912929	16.87177064	11.63960804	62.92881328
213.8315967	424.2052003	596.907006	287.8745865	300.0432294	976.0806146
20.74485639	164.587699	204.6098758	164.4997637	190.1135979	241.4551205
30.3194055	33.30941527	8.461310651	41.12494093	28.4523752	19.83625636
52.66002007	23.51252842	341.5292663	28.47111295	45.26514236	95.76123759
0	0	0	0	0	0
4.787274552	126.3798403	211.5327663	184.5349914	75.01080734	390.5690476
1.595758184	0	2.307630178	0	2.586579563	12.99616796
582.4517372	264.5159448	222.3017071	152.9004214	131.9155577	158.006042
1026.072512	3370.129074	8995.911643	4118.821007	5654.262926	29854.24983
111.7030729	361.5051245	373.8360888	344.8168124	563.8743448	274.2875448
502.7117007	421.3249156	338.4524261	299.2524869	390.7933734	249.232301
201.0655312	402.6520493	460.7568255	393.323153	332.3754739	559.5192311
177.1291584	85.23291554	76.92100592	254.1310453	98.29002341	120.3855558
542.5577826	323.2972658	394.6047604	533.5697465	769.5074201	400.8291802
100.5327656	2188.624521	5061.40219	2913.543892	4858.88971	2806.48827
20.34591685	131.9542689	18.90718326	82.56622757	18.80443343	247.3444366
1023.806536	1312.224414	760.7872091	979.1321193	2136.411256	302.3319073
0	0.979688684	1.538420118	0	0	0.68400884
0	0	0	0	0	0
264.8958585	361.5051245	385.3742397	400.7045527	447.4782645	421.3494454
354.2583168	182.2220953	236.9166982	191.916391	174.5941205	210.6747227
1418.629026	4124.489361	2855.30774	889.9859012	2472.770063	1304.404858
17.55334002	182.2220953	116.1507189	12.65382798	54.31817083	235.2990409
351.0668005	294.886294	422.2963225	277.3297299	312.9761272	363.2770949
810.6451575	1018.876232	945.3591628	1578.56504	2113.235503	855.01105
81.38366738	72.49696264	98.45888758	103.3395952	72.42422778	135.4337503

60.63881099	64.65945317	136.9193905	112.8299662	65.95777887	154.5859978
28.72364731	22.53283974	28.46077219	25.30765596	18.10605694	30.09638896
6.383032736	1.959377369	6.153680474	3.163456995	7.75973869	8.20810608
488.3020043	992.4246372	307.6840237	755.0117361	669.9241069	744.2016179
9.574549104	22.53283974	43.84497338	21.0897133	29.74566498	73.87295472
70.2133601	114.6235761	66.15206509	138.1376221	62.07790952	99.18128179
1693.099433	3032.136478	1505.344086	2949.396405	2486.99625	4292.155471
205.8528057	0	46.92181361	0	95.70344385	1.36801768
0	23.51252842	1.538420118	53.77876891	12.93289782	0.68400884
143.6182366	108.745444	212.3019763	86.46782453	100.876603	238.7190851
19.14909821	43.10630211	20.7686716	37.96148394	34.91882411	84.13308732
6.383032736	4.898443422	9.230520711	5.272428325	7.75973869	27.3603536
172.3418839	0.979688684	0	7.381399655	0	227.7749437
0	0.979688684	0	13.70831364	7.75973869	2.05202652
1.595758184	0	14.61499113	0	0	3.4200442
4.787274552	18.614085	43.07576332	20.03522763	14.2261876	18.46823868
386.1734805	354.6473037	558.446503	295.2559862	288.4036213	385.0969769
7.97879092	24.49221711	16.9226213	44.28839793	15.51947738	12.31215912
213.8315967	349.7488603	246.916429	291.0380435	367.294298	277.0235802
12.76606547	30.37034921	22.30709172	59.05119724	129.3289782	10.94414144
2264.380863	953.2370899	442.2957841	760.2841644	884.6102107	393.305083
1.595758184	24.49221711	28.46077219	10.54485665	25.86579563	43.77656576
23.93637276	1.959377369	9.99973077	16.87177064	3.879869345	15.73220332
9.829870413	4.153880022	3.484521568	5.514960028	0	5.923516554
636.7075154	263.5362561	128.4580799	401.7590384	371.1741674	158.006042
11.17030729	10.77657553	67.69048521	1.054485665	11.63960804	68.400884
368.6201405	385.0176529	70.76732545	102.2851095	232.7921607	177.8422984
27.12788913	0.979688684	0	2.10897133	1.293289782	21.20427404
16907.05796	12999.48915	16201.10227	6695.983973	7643.34261	13412.72934
0	4.898443422	1.538420118	1.054485665	1.293289782	2.73603536
593.6220444	472.2099458	258.4545799	426.0122086	552.2347368	230.5109791
0	0	1.538420118	0	0	0
0	0	1.538420118	0	0	6.8400884
178.7249166	172.4252084	635.3675089	573.6402017	213.392814	530.7908598
59.04305281	0.979688684	18.46104142	0	0	0.68400884
266.4916167	322.3175771	403.066071	311.0732712	278.0573031	376.8888708
135.6394456	365.4238793	409.2197515	446.0474363	650.5247602	374.1528355

0	0	0.769210059	0	1.293289782	0.68400884
9.574549104	37.8257801	39.92200207	24.99131026	9.428082509	78.59261571
19.14909821	79.35478343	105.3817781	81.1953962	96.99673363	220.2508465
0	5.878132106	4.615260355	0	0	3.4200442
9.574549104	7.837509475	7.692100592	3.163456995	5.173159127	7.52409724
216.0497005	279.4268065	245.2395511	407.3689021	239.8147242	174.5248555
0	12.7359529	10.76894083	2.10897133	1.293289782	19.15224752
173.9376421	351.7082377	217.6864468	485.0634059	172.007541	248.9792177
250.5340349	170.4658311	237.6859083	285.7656152	252.1915074	191.5224752
314.3643622	297.82536	550.7544024	596.8388864	390.5735141	454.8658786
0	0	0.769210059	0	319.4425761	34.200442
236.1722112	350.728549	209.2251361	419.6852947	534.1286799	231.1949879
1.595758184	0	0.769210059	1.054485665	14.2261876	1.36801768
9.574549104	3.918754737	1.538420118	28.47111295	0	2.05202652
1.81916433	0.989485571	0.930744172	1.434100504	1.500216147	1.703182012
3.191516368	0	1.538420118	0	1.293289782	0.68400884
33.51092186	78.37509475	77.69021598	74.86848221	86.65041538	125.8576266
976.6040086	470.2505685	567.6770237	584.1850584	556.1146062	827.6506964
23.93637276	37.22817	8.461310651	28.47111295	40.09198323	9.576123759
1163.307716	0.979688684	6.922890533	0	1.293289782	110.8094321
365.4286241	207.6940011	161.5341124	170.8266777	343.8986859	190.1202571
11.17030729	0	0	0	2.586579563	0
341.4922514	1410.751705	1033.81832	2240.782038	2011.065611	1652.565357
62.23456918	124.4204629	76.92100592	125.4837941	219.8592629	125.1736177
376.5989314	736.7258906	804.5937219	859.4058169	637.5918624	465.81002
0	0.979688684	0.769210059	1.054485665	0	0.68400884
1053.200401	941.4808256	329.2219053	469.2461209	897.5431085	627.2361062
177.1291584	191.0392934	291.5306124	409.140438	239.2586096	306.4359603
239.3637276	349.7488603	149.9959615	202.4612477	316.8559965	350.2125261
245.7467603	112.6641987	148.4575414	89.63128152	64.66448909	177.3771724
373.4074151	253.7393692	339.9908462	322.6726135	259.9512461	297.5438454
3.191516368	5.878132106	3.076840237	31.63456995	3.879869345	9.576123759
264.8958585	566.2600595	306.9148136	389.1052104	362.1211389	228.4589525
70.2133601	169.4861424	197.6869852	170.8266777	204.3397855	229.1429614
0	12.7359529	8.461310651	39.0159696	12.93289782	22.57229172
157.9800602	53.88287764	96.92046746	83.30436753	77.5973869	57.45674256
110.1073147	103.8470005	266.1466805	170.8266777	259.9512461	127.2256442

60.63881099	35.26879264	29.22998225	36.90699827	3.879869345	18.46823868
750.0063465	12653.65905	1349.194444	16540.66214	2529.674813	4188.186127
655.8566136	133.2376611	291.5306124	346.9257838	159.0746432	304.3839338
111.7030729	240.0237277	203.8406657	180.3170487	181.0605694	213.4107581
119.6818638	35.26879264	102.3049379	66.43259689	36.21211389	88.9211492
0	0	1.538420118	0	1.293289782	1.36801768
1576.609086	3888.384388	365.3747781	4884.3776	2453.370716	873.4792886
10662.85619	8457.652412	4908.329388	6103.363029	9773.390881	3807.877212
577.6644626	482.9865214	417.6810622	731.8130515	594.9132996	839.9628555
71.80911828	54.86256632	183.0719941	152.9004214	340.1352126	34.200442
6.383032736	10.77657553	19.23025148	42.1794266	27.15908542	29.41238012
204.2570475	89.15167027	104.6125681	148.6824788	91.8235745	133.3817238
232.9806949	465.3521251	122.3043994	141.3010791	131.9155577	104.6533525
62.23456918	75.43602869	94.61283728	51.66979758	63.37119931	56.08872488
0	0	0.769210059	5.272428325	0	51.300663
75.00063465	1353.929762	1018.434118	3428.132897	766.9208406	1402.902131
25.53213094	72.49696264	44.61418344	28.47111295	31.03895476	127.9096531
1.595758184	18.614085	19.99946154	6.32691399	1.293289782	30.09638896
697.3144112	683.8227017	683.7969742	536.7226586	761.6571512	479.4901968
225.0019039	178.3033405	214.6096065	130.7562225	150.0216147	106.0213702
153.1927857	462.413059	448.4494645	701.2329672	310.3895476	784.5581394
2117.491322	4189.756221	4556.223483	3318.972541	5819.183239	3805.510541
234.576453	69.55789659	241.5319586	292.0925292	218.5659731	196.9945459
19.14909821	41.14692474	43.84497338	20.03522763	31.03895476	85.501105
3343.08148	2091.556966	4805.07063	9384.511169	4432.569666	6097.569443
3.191516368	65.63914185	71.53653551	18.98074197	55.61146062	189.4704487
221.8103876	206.7143124	135.3809704	236.204789	223.7391322	286.5997039
272.8746495	145.973614	259.22379	364.8520401	429.3722075	575.2514344
22.34061458	67.59851922	49.22944379	28.47111295	28.4523752	43.77656576
363.8328659	499.641229	319.2221746	611.6016857	1249.317929	324.2201901
0	15.67501895	13.07657101	3.163456995	6.466448909	16.41621216
1463.310255	686.7617677	749.2105977	747.6303365	574.2206631	292.7557835
15.95758184	0.979688684	4.615260355	4.21794266	3.879869345	2.05202652
579.2602208	198.8768029	249.9932692	527.2428325	363.4144287	230.5109791
362.2371078	312.5206903	425.3731628	250.9675883	296.16336	271.5515095
3379.815834	1110.966968	2570.700018	647.4541983	1316.568998	1426.158431
17.55334002	0.979688684	0	314.2367282	38.79869345	1.36801768

0	1.959377369	223.8401272	1.054485665	0	2.73603536
0	6.85782079	33.84524261	0	0	24.62431824
1185.648331	302.7238035	656.9053906	452.3743503	574.2206631	374.8368443
49.4685037	0	4.615260355	0	1.293289782	2.05202652
1744.163695	2701.981391	3199.913846	500.8806909	4898.981693	8130.129072
4.787274552	7.837509475	8.461310651	3.163456995	2.586579563	9.576123759
3019.174484	535.8897103	1159.968769	500.8806909	1797.672797	536.2629305
60.63881099	0	8.461310651	0	40.09198323	0
470.7486643	225.3283974	316.9145444	218.2785326	429.3722075	314.6440664
19.14909821	38.20785869	28.46077219	43.23391226	25.86579563	43.77656576
4.787274552	43.10630211	33.84524261	8.43588532	1.293289782	21.20427404
39.8939546	114.6235761	53.07549409	65.37811123	62.07790952	125.1736177
1.595758184	5.878132106	6.922890533	9.490370985	5.173159127	8.20810608
9.574549104	19.59377369	133.8425503	482.9544346	29.74566498	91.65718455
3397.369174	2216.055804	1983.023533	2671.012189	5684.008591	1190.85939
22.70763896	13.49031318	5.099862693	2.10897133	43.54506695	43.94072788
15.95758184	1597.872244	48.46023373	2037.266305	346.6016615	55.40471604
421.2801606	35.26879264	128.4580799	30.58008428	99.58331319	105.3373614
84.57518375	48.00474553	126.9196598	91.74025285	42.6785628	93.02520223
6.383032736	11.75626421	1.538420118	12.65382798	5.173159127	8.20810608
90.95821649	34.28910395	34.61445267	43.23391226	75.01080734	69.08489284
0	0	1.538420118	2.10897133	1.293289782	1.36801768
526.7119038	639.6093513	1156.853469	593.9812302	685.0814632	816.9049175
9.574549104	21.55315106	28.46077219	43.23391226	9.053028472	58.82476024
4.787274552	95.02980238	52.30628403	45.34288359	29.74566498	145.0098741
0	0	1.153815089	0	0	1.7100221
0	0	1.153815089	0	0	1.7100221
239.3637276	1307.884394	759.9795385	2021.44902	3879.869345	250.3472354
240.9594858	363.4645019	298.453503	386.996239	261.2445359	387.1490034
4.787274552	21.55315106	18.46104142	6.32691399	34.91882411	30.7803978
0	79.35478343	72.30574557	12.65382798	20.69263651	145.6938829
71.80911828	174.3845858	128.4580799	236.204789	102.1698928	114.9134851
205.8528057	143.0345479	157.6880621	136.0286508	111.2229212	114.9134851
245.7467603	676.9648809	431.5268432	813.0084477	487.5702477	454.1818697
16760.24821	18396.59411	18521.03981	7836.937462	16548.93605	17649.4801
510.6426189	460.4536816	906.8986598	1275.927655	336.2553433	486.3302852
473.9401806	344.8504169	336.9140059	419.6852947	409.9728608	351.5805437

317.5558786	432.0427098	337.9601316	551.4960028	572.9273733	403.5652156
39.8939546	130.298595	35.38366272	71.70502522	155.1947738	26.67634476
0	2.939066053	2.307630178	1.054485665	2.586579563	1.36801768
3.191516368	3.918754737	9.230520711	1.054485665	5.173159127	13.6801768
579.2602208	604.4679182	43.07576332	379.6148394	133.2088475	119.701547
0	0	0	9.490370985	6.466448909	1.36801768
2.95215264	0	0.738441657	0	0	1.518499625
4.787274552	2.939066053	5.384470415	7.381399655	7.75973869	7.52409724
4.787274552	18.614085	5.384470415	15.81728497	16.81276716	29.41238012
41.48971278	41.42123757	47.29103444	51.80688072	54.31817083	32.14841548
389.3649969	144.9939253	193.0717249	235.1503033	332.3754739	231.1949879
14.36182366	71.51727396	59.22917456	45.34288359	33.62553433	19.15224752
0	0.979688684	0	0	0	0.68400884
191.4909821	170.4658311	107.6894083	211.9516187	252.1915074	156.6380244
27.12788913	29.39066053	18.46104142	27.41662729	37.50540367	27.3603536
71.80911828	260.59719	288.4537722	261.5124449	269.0042746	227.0909349
2293.10451	6985.180319	5680.616287	4355.025796	5810.750989	4130.045376
6.383032736	18.614085	220.763287	175.0446204	108.6363417	256.503315
97.34124922	110.7048213	247.6856391	117.0479088	84.06383581	142.2738387
185393.3986	155759.7438	70513.81689	221527.8775	107577.8745	36368.39434
454.7910824	311.5410016	275.3772012	429.1756656	382.8137754	221.6188641
98.93700741	41.14692474	64.61364497	40.07045527	67.25106865	51.98467184
0	0	0	0	0	0.68400884
3914.394825	1.959377369	0.769210059	0	1.293289782	1.36801768
944.6888449	1630.201971	1358.424965	1174.697031	1432.965078	2355.726445
3.191516368	1.959377369	6.153680474	7.381399655	5.173159127	3.4200442
0	940.501137	13.84578107	14.76279931	10.34631825	853.6430323
0	0	4.615260355	2.10897133	0	2.05202652
682.9845027	422.2458229	638.4443492	672.7618542	708.7228004	418.6134101
614.3669008	665.2086167	309.2224438	352.1982121	468.170901	536.2629305
271.2788913	3030.177101	963.8202042	1899.128683	1646.357892	1569.116279
0	0	1.538420118	3.163456995	0	1.36801768
22.34061458	324.2769545	131.5349201	88.57679586	178.4739899	205.202652
1.595758184	1.959377369	13.84578107	35.85251261	9.053028472	19.15224752
587.2390117	1529.294036	2421.473266	1315.99811	1968.387048	3232.625778
39.8939546	341.8427726	182.1027894	291.4809275	321.8739609	326.7852233
309.5770877	1196.199884	599.2146361	1239.020656	1568.760505	1788.683117

823.4112229	312.5206903	161.5341124	353.2526978	578.1005324	220.2508465
3.191516368	11.75626421	0.769210059	3.163456995	7.75973869	1.36801768
505.8553443	234.1455956	310.7608639	224.6054466	236.6720301	267.4474564
6.383032736	18.614085	12.30736095	64.32362556	29.74566498	23.25630056
0	1.959377369	9.99973077	4.21794266	3.879869345	7.52409724
111.7030729	27.43128316	61.53680474	15.81728497	54.31817083	45.82859228
134.0436875	104.8266892	76.15179586	88.57679586	91.8235745	205.8866608
368.6201405	179.2830292	206.9175059	176.099106	228.9122914	275.6555625
178.7249166	529.0318895	398.4508107	801.4091054	688.0301639	465.1260112
6.383032736	0	1.538420118	1.054485665	1.293289782	0
0	0	0	0	0	1.36801768
4.787274552	0	6.922890533	0	0	4.95906409
1.595758184	1.959377369	0	1.054485665	5.173159127	0.68400884
17.55334002	0	0	0	7.75973869	2.05202652
23.93637276	14.69533026	22.30709172	17.9262563	11.63960804	35.56845968
17.55334002	36.24848132	57.69075444	27.41662729	56.9047504	89.60515804
12.76606547	11.75626421	8.461310651	62.21465423	20.69263651	41.72453924
9.574549104	2.939066053	5.384470415	6.32691399	3.879869345	7.52409724
1.595758184	9.796886843	54.6139142	18.98074197	124.155819	61.5607956
82.97942557	93.07042501	163.8417426	114.9389375	81.47725625	170.3182012
0	0	0	0.527242832	5.173159127	2.05202652
0	0	82.30547634	0	0	35.56845968
46.27698734	108.745444	99.22809764	89.63128152	106.0497621	114.9134851
94.14973285	340.9316621	230.7630178	252.0220739	240.5518994	329.6922609
0	0	0.769210059	1.054485665	5.173159127	0.68400884
544.8556743	108.9217879	148.7959939	33.60645814	28.34891202	198.1915614
157.9800602	157.7298782	195.379355	172.9356491	191.4068877	229.8269702
276.0661658	213.5721332	98.45888758	114.9389375	49.14501171	56.08872488
0	21.55315106	48.46023373	4.21794266	11.63960804	62.92881328
0	2.939066053	6.922890533	0	2.586579563	3.4200442
2534.063996	1435.243923	1623.802435	1898.074197	1795.086217	1408.374201
181.916433	192.9986708	327.6834852	147.6279931	239.2586096	422.7174631
0	5.878132106	6.153680474	2.10897133	0	10.2601326
1255.861691	983.6074391	98.45888758	294.2015005	1078.603678	258.5553415
255.3213094	227.2877748	192.3025148	239.3682459	201.753206	127.2256442
1.595758184	0	0	0	0	3.4200442
210.6400803	60.74069843	113.0738787	115.9934231	104.7564723	104.6533525

507.4511025	718.1118056	743.8261273	1399.302477	2036.931406	590.9836377
1.595758184	7.837509475	1.538420118	2.10897133	0	0.68400884
31.91516368	264.5159448	219.9940769	194.0253624	109.9296314	366.6287382
94.14973285	279.211275	146.1499113	244.6406743	340.1352126	305.0679426
0	3.918754737	4.615260355	6.32691399	0	11.62815028
1.595758184	14.69533026	3.846050296	2.10897133	3.879869345	13.6801768
2821.300469	1177.51722	1300.557292	1395.084535	2044.691145	895.9831795
0	2.939066053	3.846050296	1.054485665	1.293289782	4.10405304
15.95758184	50.94381159	58.4599645	13.70831364	41.38527302	58.82476024
362.0137016	29.92948931	29.27613485	747.7252402	147.202243	136.6307658
95.74549104	101.8876232	123.8428195	192.9708767	170.7142512	209.306705
665.4311627	862.1260422	895.3605089	816.1719047	825.1188808	679.9047869
438.4664762	602.067681	611.8142969	404.9224953	616.4853732	947.1607209
0	0.979688684	0	1.054485665	0	0
162.7673348	45.06567948	74.61337574	138.1376221	156.4880636	98.49727295
226.5976621	91.11104764	233.839858	121.2658515	124.155819	162.7941039
38.29819642	74.45634001	69.99811539	78.03193921	58.19804018	34.200442
231.3849367	19.59377369	11.53815089	5.272428325	29.74566498	2.05202652
31.91516368	51.92350027	59.99838462	28.47111295	32.33224454	111.4934409
0	0.979688684	3.076840237	1.054485665	0	0
505.8553443	431.0630211	376.912929	414.4128663	248.3116381	349.5285172
357.4498332	292.9269166	228.4553876	285.7656152	337.548633	191.5224752
181.916433	311.5410016	193.0717249	323.7270991	384.1070652	116.2815028
1.595758184	2.939066053	5.384470415	7.381399655	2.586579563	5.47207072
1902.143755	1251.003669	1356.117334	1240.686744	3741.44854	664.0699823
0	3.918754737	9.99973077	0	2.586579563	7.52409724
94.14973285	231.2065295	313.8377042	93.84922418	182.3538592	459.6539405
1.595758184	0.979688684	0.769210059	4.21794266	7.75973869	1.36801768
282.4491986	373.2613887	324.606645	487.1723772	394.4533834	239.403094
46.27698734	104.8266892	219.2248669	185.589477	45.26514236	208.6226962
156.384302	174.3845858	191.5333047	119.1568801	191.4068877	183.3143691
0	0	0	0	1.293289782	0.34200442
260.108584	420.2864456	144.6114911	392.2686674	297.4566498	249.6632266
86.17094193	107.7657553	107.6894083	181.3715344	77.5973869	155.2700067
0	5.18255314	0	3.363809271	0	0
4.787274552	0	397.6816006	0	0	35.56845968
339.8964932	499.641229	256.9161598	268.8938446	435.8386564	233.2470144

1214.371978	947.3589577	888.4376184	1024.960066	923.4089042	829.018714
714.8996664	1139.37794	379.2205592	534.6242321	880.7303414	611.5039029
1.595758184	0	11.53815089	0	6.466448909	10.2601326
0	10.77657553	0	1.054485665	12.93289782	0
15.95758184	33.30941527	17.69183136	26.36214162	18.10605694	36.93647736
169.1503675	574.097569	299.222713	543.0601175	770.8007099	373.4688266
59.04305281	221.4096427	539.2085594	413.3583807	338.8419228	378.9408973
12.76606547	4.898443422	13.84578107	50.61531192	6.466448909	28.72837128
28.72364731	12.7359529	48.46023373	10.54485665	25.86579563	32.14841548
170.7461257	122.4610855	250.7624793	196.1343337	151.3149045	270.1834918
8.808585176	6.142648051	48.16793391	31.64511481	9.259954837	3.399523935
605.6700187	811.15284	815.839573	596.8072518	863.6718491	683.6052747
1256.308503	1115.277598	256.1469497	629.7388391	464.2910316	528.9440359
0	68.5782079	20.7686716	0	2.586579563	95.76123759
105.3200401	116.5829534	68.45969527	124.4293085	115.1027906	476.0701526
114.8945892	61.72038711	16.15341124	37.96148394	103.4631825	25.99233592
11.17030729	6.85782079	23.84551184	52.72428325	10.34631825	22.57229172
95.74549104	234.1455956	316.1453343	163.4452781	226.3257118	225.0389083
47.87274552	133.2376611	176.9183136	123.3748228	76.30409712	43.77656576
0	20.57346237	34.61445267	1.054485665	5.173159127	15.73220332
38.29819642	96.00949106	59.99838462	54.83325458	50.43830149	118.3335293
0	11.75626421	10.76894083	1.054485665	3.879869345	7.52409724
489.8977625	234.1455956	194.610145	255.1855309	249.6049279	240.7711117
0	0	0	0	0	0.68400884
9.766040086	2.2336902	6.399827693	7.223226805	2.638311155	6.149239471
70.2133601	63.67976448	71.53653551	91.74025285	49.14501171	52.66868068
708.5166337	1127.621676	983.0504557	1446.754332	1138.095008	2224.396748
107.2030348	422.0890728	88.12839649	168.7598858	363.5308248	210.0112341
6553.85865	6599.829572	6258.216121	8429.895841	4153.956249	5484.034034
70.2133601	90.13135896	139.2270207	64.32362556	99.58331319	134.0657326
3.191516368	0	5.384470415	0	0	19.83625636
0	4.898443422	0	0	1.293289782	0.68400884
81.38366738	98.94855712	46.15260355	70.65053955	71.130938	32.14841548
226.5976621	159.6892555	300.7611332	349.0347551	309.0962578	217.5148111
0	0.979688684	20.54560068	0	2.586579563	13.86485919
329.1729982	361.1916241	334.5294548	178.2080774	91.8235745	273.9729008
0	0	2.061482959	0	0	0.930252022

0	0.979688684	48.46023373	1.054485665	5.173159127	25.99233592
307.9813295	1292.209375	2107.635562	1644.997637	1047.564723	1346.129397
1412.245993	2901.837883	721.5190356	1127.245176	1623.078676	1196.331461
0	0	1.753798935	2.446406743	0	0
0	111.68451	0	145.5190218	1.293289782	0.68400884
137.2352038	96.98917975	49.99865385	112.8299662	138.3820066	122.4375824
0	0	0.769210059	0	2.586579563	3.4200442
20.74485639	0	2.307630178	0	0	283.8636686
234.576453	308.6019356	127.6888698	209.8426473	236.6720301	334.4803227
1.595758184	10.77657553	19.23025148	9.490370985	7.75973869	26.67634476
71.80911828	31.3500379	36.92208284	46.39736926	19.39934673	70.45291052
106.9157983	51.92350027	48.46023373	26.36214162	11.63960804	7.52409724
323.9389113	200.8361803	134.6117604	216.1695613	288.4036213	217.5148111
173.9376421	164.587699	108.4586184	72.75951088	312.9761272	52.66868068
90.95821649	246.8815485	289.9921923	222.4964753	235.3787403	294.80781
116.4903474	30.37034921	153.8420118	7.381399655	5.173159127	115.597494
118.0861056	163.1083691	83.0746864	142.3555648	125.4491088	121.0695647
126.0648965	87.19229291	174.6106834	258.3489879	160.3679329	298.2278542
494.2063096	76.20018587	10.76894083	58.29196756	8.277054603	22.09348553
73.40487646	88.17198159	52.30628403	74.86848221	86.65041538	134.7497415
29.48961124	11.87382685	2.5922379	117.5540619	9.531545691	21.90196306
75.00063465	102.8673119	113.8430888	75.92296788	29.74566498	134.0657326
5430.3651	2274.837125	1987.638793	3751.859996	2876.276475	755.1457593
1689.907917	3521.001131	1183.045071	3155.02111	2119.701952	2039.030352
6.383032736	0	2.307630178	1.054485665	0	0.68400884
49.4685037	109.7251326	129.9965	87.52231019	120.2759497	137.4857768
135.6394456	98.94855712	148.4575414	82.24988187	90.53028472	100.5492995
17.55334002	164.587699	275.3772012	287.8745865	435.8386564	171.00221
122.8733802	773.9540606	309.2224438	953.2550411	901.4229779	528.7388333
188.2994657	272.3534542	389.22029	327.9450418	347.8949513	307.803978
442.025017	438.9005306	592.2917456	509.3165762	596.2065894	511.6386123
84.57518375	356.6066811	267.6851006	319.5091565	366.0010082	274.2875448
7.97879092	29.39066053	19.99946154	13.70831364	10.34631825	25.30832708
0	0	2.307630178	0	0	4.10405304
0	0	0	0	0	0
0	0	3.076840237	0	0	2.05202652
183.5121912	48.00474553	19.99946154	22.14419896	51.73159127	51.300663

3483.540116	1868.266321	1239.197405	1441.481904	2594.339302	1788.683117
31.91516368	22.53283974	20.7686716	27.41662729	20.69263651	23.9403094
0	10.77657553	0.769210059	1.054485665	2.586579563	9.576123759
0	0	0.769210059	0	2.586579563	0
23.93637276	50.94381159	96.92046746	85.41333886	208.2196549	95.76123759
44.68122915	28.41097185	19.23025148	29.52559862	18.10605694	17.100221
51.06426189	23.51252842	14.61499113	14.76279931	14.2261876	19.15224752
716.862449	646.0752967	367.8285582	498.8033541	743.9390812	306.1213162
98.93700741	140.0954819	127.6888698	157.1183641	109.9296314	144.3258652
0	0.979688684	3.846050296	0	1.293289782	4.10405304
357.4498332	335.05353	281.5308817	461.8647213	269.0042746	323.5361813
244.1510021	190.0596048	197.6869852	440.775008	234.0854505	259.2393503
59.04305281	56.82194369	91.53599705	60.1056829	93.11686429	80.02903428
0	38.20785869	5.384470415	0	14.2261876	4.10405304
0	3.918754737	0.769210059	1.054485665	1.293289782	0
373.4074151	138.1361045	245.3780089	133.9196795	228.9122914	212.0427404
0	0	0	0	0	0.68400884
11.17030729	5.878132106	0.769210059	0	3.879869345	4.10405304
368.6201405	209.6533784	209.2251361	186.6439627	322.0291557	280.4436244
1571.821811	7569.074775	7350.571326	3818.292593	5206.784661	4407.752965
0	0.979688684	2.307630178	0	1.293289782	0
0	2.939066053	0.769210059	0	1.293289782	0.68400884
274.4704076	364.4441906	381.5281894	339.5443841	382.8137754	526.0027979
722.8784573	515.316248	745.3645474	697.0150245	684.1502945	453.4978609
394.1522714	189.0799161	36.15287278	2.10897133	46.55843214	384.4129681
331.9177023	93.07042501	132.3041302	94.90370985	86.65041538	134.0657326
129.2564129	136.1767271	256.1469497	194.0253624	182.3538592	114.2294763
0	0	1.538420118	0	0	0.68400884
1.595758184	0	0.769210059	29.52559862	7.75973869	7.52409724
51.06426189	41.14692474	31.53761243	39.0159696	31.03895476	41.72453924
65.42608554	93.07042501	68.45969527	141.3010791	107.3430519	79.34502544
54.25577826	126.3798403	83.84389646	231.9868463	59.49132996	101.9173172
375.0031732	143.0345479	157.6880621	437.611551	409.9728608	149.797936
4.787274552	6.85782079	14.61499113	1.054485665	15.51947738	12.99616796
9.574549104	45.06567948	140.7654408	9.490370985	18.10605694	85.501105
82.97942557	98.94855712	76.92100592	124.4293085	106.0497621	86.86912268
349.4710423	123.4407742	175.3798935	27.41662729	375.0540367	31.46440664

6.079838681	0.979688684	0.769210059	9.195114998	0	0
721.2826992	133.2376611	390.7587101	156.0638784	177.1807001	168.2661746
1233.521076	1731.109905	1246.889506	3846.763706	1720.07541	702.4770786
51.06426189	68.5782079	66.15206509	54.83325458	71.130938	63.61282212
542.5577826	183.201784	189.2256746	142.3555648	212.0995242	158.6900509
23.93637276	34.28910395	33.07603255	26.36214162	21.98592629	23.9403094
311.1728459	345.8301056	313.0684941	398.5955814	729.4154369	360.4726587
509.0468607	488.8646535	309.9916539	404.9224953	464.2910316	303.6999249
1059.583434	1530.273725	606.9067367	1031.28698	1229.918582	621.0800267
5214.395187	5423.184275	2255.246973	513.5345188	3548.696631	1610.813458
480.3232134	162.6283216	118.4583491	109.6665092	116.3960804	135.4337503
124.4691384	545.6865972	254.6085296	736.0309941	486.2769579	614.2399383
1088.307081	0	33.84524261	0	217.2726833	87.55313152
12.76606547	29.39066053	23.07630178	189.8074197	42.6785628	16.41621216
1878.207383	1022.794986	1215.351894	922.6749568	1736.888177	841.3308732
33.51092186	2.939066053	0.769210059	2.10897133	20.69263651	0.68400884
14.36182366	26.45159448	16.9226213	43.23391226	14.2261876	19.15224752
453.1953243	445.7583514	381.5281894	286.8201009	241.8451892	194.2585105
1099.477389	1343.153186	833.0544941	1930.763253	1394.166385	766.7739096
15.95758184	3.918754737	7.692100592	5.272428325	5.173159127	7.52409724
4.787274552	711.2539848	3.076840237	1148.334889	619.4858055	8.89211492
65.42608554	19.59377369	33.07603255	32.68905561	24.57250585	26.67634476
880.8585176	341.9113508	441.526574	429.1756656	368.5875878	490.4343383
502.2489308	478.8816258	381.3743474	419.9700058	387.8576055	349.6516388
67.02184373	35.26879264	125.3812397	18.98074197	32.33224454	121.7535735
1461.714497	923.8464293	643.8288196	760.2841644	1211.812525	744.8856267
59.04305281	173.4048971	72.30574557	82.24988187	100.876603	189.4704487
4.531953243	1.547908121	0	3.005284145	0	1.908384663
0	4.898443422	3.846050296	1.054485665	3.879869345	0.68400884
1.595758184	7.837509475	11.53815089	4.21794266	6.466448909	10.94414144
17352.05109	5653.74421	3725.484311	3701.940645	11786.99134	4084.059461
103.724282	87.19229291	75.3825858	161.3363067	109.9296314	165.5301393
0	12.7359529	58.4599645	70.65053955	15.51947738	4.10405304
457.9825988	292.9269166	323.8374349	249.9131026	601.3797485	257.1873238
6.383032736	39.18754737	0	2.10897133	11.63960804	1.36801768
279.2576822	419.3067569	336.9140059	469.2461209	419.0258893	155.9540155
1.595758184	9.796886843	21.53788166	8.43588532	14.2261876	2.73603536

5203.496159	2776.153622	4460.695286	2291.218087	2920.66218	2504.956653
734.0487646	278.2315863	646.9056598	652.7266266	1092.829866	269.4994829
880.9223479	1622.129336	1267.51972	1190.988834	1381.414547	1282.94066
300.8802056	111.1946657	61.41373113	119.9899238	58.6894903	56.11608523
124.4691384	291.9472279	181.533574	95.95819551	237.9653198	738.0455383
0	0	0	0	0	0
0	0	0	0	0	0
0	0	0	0	0	0
0	0	0	0	0	0
0	0	0	0	0	0
6.383032736	20.57346237	42.30655326	9.490370985	23.27921607	34.200442
55.85153644	201.815869	303.8379734	197.1888193	186.2337286	268.8154741
4.787274552	6.85782079	4.615260355	2.10897133	0	6.8400884
1.595758184	0	3.076840237	5.272428325	6.466448909	2.73603536
5821.325855	3501.407358	938.4362723	3519.87315	4293.722075	2282.537499
17.55334002	16.65470763	36.15287278	7.381399655	10.34631825	50.61665416
197.8740148	183.201784	228.4553876	179.262563	283.2304622	151.8499625
403.7268205	378.1598322	879.2070977	263.6214162	96.99673363	372.7848178
1.595758184	6.85782079	3.076840237	3.163456995	1.293289782	6.8400884
394.1522714	384.0379643	507.6786391	342.7078411	372.4674571	609.4518764
1.595758184	0	0.769210059	1.054485665	0	0
0	3.379925961	1.761491036	4.681916352	0	11.82651284
5518.1318	2750.965826	2651.467074	2100.535445	2787.03948	1747.642586
103.724282	100.9079345	235.3782781	43.23391226	128.0356884	192.8904929
0	0	3.846050296	0	0	0
1096.285872	751.4212209	617.6756776	1328.651938	1010.05932	470.5980819
411.7056115	477.1083893	377.6821391	511.4255475	598.7931689	297.5438454
135816.5748	116525.1518	106374.8283	76817.17172	131702.1649	81211.68556
108.5115565	211.6127558	253.8393195	362.7430687	362.1211389	311.908031
19.14909821	43.10630211	95.38204734	120.2113658	67.25106865	73.18894588
210.9432743	106.5411444	98.17427986	183.7757617	76.4592919	141.8360731
9.574549104	19.59377369	52.30628403	52.72428325	33.62553433	88.9211492
52.66002007	103.8470005	43.84497338	91.74025285	125.4491088	77.97700776
191.6824731	220.9295952	253.4008698	269.0414726	235.3787403	241.9339267
79.7879092	90.13135896	112.3046686	106.5030522	96.99673363	127.2256442
4233.546462	1454.837696	1726.107373	1924.436339	3136.227721	2053.387697
204.2570475	236.1049729	190.7640947	361.6885831	214.6861038	249.6632266

15.95758184	5.878132106	8.461310651	7.381399655	20.69263651	10.94414144
253.7255513	128.3392176	177.6875237	210.897133	124.155819	188.7864398
6.383032736	4.898443422	10.76894083	1.054485665	3.879869345	17.78422984
499.4723116	324.2769545	399.2200207	583.1305727	382.8137754	443.9217371
0	0	0	0	0	0
0	0	0	0	0	0
0	0	0	0	0	0
0	0	0	0	0	0
0	0	0	0	0	0
0	0	0	0	0	0
0	0	0	0	0	0
0	112.6641987	10.76894083	0	0	0
0	2.939066053	0	35.85251261	0	1.36801768
1.595758184	15.67501895	3.076840237	2.10897133	6.466448909	10.94414144
38.29819642	30.37034921	6.922890533	40.07045527	34.91882411	14.36418564
25.70766434	24.40404513	36.27594639	28.47111295	30.62510203	40.59592465
43.08547097	54.86256632	16.9226213	45.34288359	24.57250585	43.77656576
3.191516368	9.796886843	18.46104142	1.054485665	6.466448909	18.46823868
19.14909821	26.45159448	14.61499113	21.0897133	23.27921607	54.7207072
0	0	0	0	0	2.73603536
23.93637276	526.0928235	2307.630178	535.6787178	549.6481572	965.820482
60.63881099	49.9641229	64.61364497	99.12165251	73.71751756	54.03669836
1.595758184	12.7359529	8.461310651	3.163456995	2.586579563	218.1988199
322.3431532	413.4286248	169.9954231	147.6279931	280.6438826	437.7656576
170.7461257	73.47665132	69.99811539	165.5542494	122.8625293	101.9173172
196.2782566	407.5504927	414.6042219	420.7397803	373.7607469	545.8390543
1.595758184	0.979688684	0	0	2.586579563	1.36801768
0	0	0.769210059	2.10897133	0	0.68400884
0	1.959377369	4.615260355	3.163456995	6.466448909	1.36801768
46.27698734	161.6486329	180.7643639	199.2977907	147.4350351	294.80781
36.70243823	211.6127558	138.4578107	71.70502522	104.7564723	92.34119339
68.61760191	128.3392176	206.9175059	207.733676	146.1417453	182.6303603
41.48971278	12.7359529	50.76786391	16.87177064	60.78461974	65.66484864
165.9588511	60.74069843	86.15152663	137.0831364	89.23699494	74.55696356
0	0	0	0	0	0
0	0	0	0	0	0
0	0	0	0	0	0

0	0	0	0	0	0
0	0	0	0	0	0
0	0	0	0	0	0
0	0	0	0	0	0
0	0	0	0	0	0
0	0	0	0	0	0
0	0	0	0	0	0
0	0	0	0	0	0
0	0	0	0	0	0
0	0	0	0	0	0
0	0	0	0	0	0
0	0	0	0	0	0
0	0	0	0	0	0
0	0	0	0	0	0
0	0	0	0	0	0
1265.43624	713.2133622	332.2987456	686.4701679	681.563715	560.2032399
250.5340349	210.6330671	266.9158906	423.9032373	173.3008308	286.5997039
4.787274552	19.59377369	33.07603255	13.70831364	18.10605694	55.40471604
164.3630929	144.0142366	158.4572722	299.4739288	206.9263651	205.202652
421.2801606	629.939824	1673.801089	699.1239959	451.3581338	4225.122604
59.04305281	76.41571738	47.69102367	81.1953962	65.95777887	75.92498124
52.66002007	193.9783595	156.149642	94.90370985	106.0497621	138.8537945
8527.731735	4137.225314	5491.390613	1350.796137	9314.273008	3120.448328
86.17094193	123.4407742	37.6912929	114.9389375	247.0183483	51.98467184
9135.715603	3087.978733	2998.380811	4278.048343	5491.308413	1829.723647
22.34061458	6.85782079	0	8.43588532	21.98592629	0
0	32.32972658	2.307630178	0	0	0.68400884
0	0	0.769210059	6.32691399	2.586579563	4.10405304
42399.29495	19174.46693	39169.71464	20541.38075	19999.43318	25995.07195
98.93700741	284.1097185	681.5201125	332.1629845	172.007541	255.1352973
295.215264	116.5829534	280.7616716	158.1728497	256.0713768	360.4726587
78.19215101	39.18754737	145.3807012	90.68576719	73.71751756	125.8576266
46.27698734	35.26879264	47.69102367	15.81728497	28.4523752	19.15224752
2361.722112	1064.9216	1519.959077	401.7590384	2739.187758	1885.128363
137.2352038	370.3223227	200.7638255	299.4739288	187.5270184	254.4512885
298.4067804	162.6283216	219.9940769	233.041332	325.909025	178.5263072
370.2158987	284.1097185	229.9938077	293.1470149	353.0681104	246.2431824

7.97879092	66.61883053	83.0746864	34.79802694	71.130938	125.1736177
531.3874753	573.1178803	633.0598787	965.9088691	611.7260668	449.3938079
89.3624583	944.4198917	1727.645793	279.4387012	290.9902009	1072.525861
288.57691	16.65470763	13.84578107	3.163456995	40.09198323	7.52409724
100.5327656	127.359529	114.6122988	130.7562225	169.4209614	69.76890168
14.36182366	13.71564158	73.84416569	2.10897133	14.2261876	49.24863648
204.2570475	166.5470763	127.6888698	128.6472511	164.2478023	174.4222542
0	0	0	0	0	0
0	0	0	0	0	0
0	0	0	0	0	0
0	0	0	0	0	0
0	0	0	0	0	0
0	0	0	0	0	0
0	0	0	0	0	0
0	3.918754737	189.9948846	0	0	39.67251272
1.595758184	0	0	2.10897133	0	0
0	6.85782079	1.538420118	3.163456995	1.293289782	0.68400884
11.17030729	0.979688684	15.38420118	3.163456995	7.75973869	17.100221
12.76606547	482.9865214	199.2254053	87.52231019	192.7001775	474.7021349
130.8521711	187.1205387	245.3780089	313.1822425	232.7921607	223.6708907
172.3259263	309.4738585	162.0648674	363.4812087	323.1931165	170.1403589
188.2994657	294.886294	150.7651716	165.5542494	647.9381807	240.7711117
20.74485639	0.979688684	10.76894083	11.59934231	3.879869345	6.15607956
1.787249166	7.024367867	4.215271125	5.97893372	0	0.98497273
25.53213094	62.7000758	52.30628403	36.90699827	40.09198323	80.71304312
0	2.664753221	1.538420118	0	2.638311155	0
0	0	0.769210059	0	1.293289782	1.36801768
12424.57322	3.918754737	0.769210059	16.87177064	228.9122914	1.36801768
742.0275556	1371.564158	713.826935	1230.584771	1493.749698	789.3462013
0	0	0	0	2.586579563	0
19.14909821	3.918754737	0	5.272428325	2.586579563	0
1833.526153	917.9682972	923.0520711	1349.741651	885.9035005	915.8878367
0	0	0	0	0	0
0	0	0	1.054485665	0	0.68400884
51.06426189	1708.577065	588.4456953	7258.024832	2008.479031	570.4633725
587.2390117	800.4056551	706.1348344	447.1019219	387.9869345	757.1977858
1278.202305	794.527523	759.2103285	1550.093927	1141.974877	350.8965349

143.6182366	26.45159448	76.92100592	4.21794266	25.86579563	34.200442
49.4685037	0	0.769210059	1.054485665	2.586579563	0
6983.037813	2197.441719	626.9061983	1369.776879	2558.127188	2704.570953
161.1715766	3129.125658	200.7638255	3184.546708	563.8743448	910.415766
11.17030729	20.57346237	41.5373432	131.8107081	41.38527302	84.81709616
521.8129262	511.3974932	599.2146361	431.284637	503.0897251	698.3730256
1587.779393	815.1009854	609.983577	368.0154971	741.0550449	399.4611625
3.191516368	7.837509475	20.7686716	18.92801769	12.93289782	34.91865128
0	0.979688684	3.076840237	8.43588532	0	1.36801768
402.1310624	312.5206903	182.302784	262.5669306	294.8700702	225.7229172
22.34061458	289.0081619	113.0738787	167.6632207	31.03895476	77.97700776
419.6844024	897.3948348	297.6842929	109.6665092	230.2055812	244.8751647
0	0	3.076840237	0	0	4.78806188
63.83032736	42.12661343	43.07576332	67.48708256	55.61146062	69.76890168
497.8765534	1374.503224	1429.9615	667.4894259	786.3201873	1410.426228
14.36182366	19.59377369	26.92235207	28.47111295	41.38527302	38.98850388
0	27.43128316	20.7686716	10.54485665	9.053028472	54.03669836
52.66002007	71.51727396	70.76732545	59.05119724	32.33224454	64.29683096
3759.606281	3189.866356	1458.422272	1833.750571	3018.538351	1781.159019
161.1715766	342.8910395	984.5888758	209.8426473	297.4566498	367.9967559
5464.48241	6188.517075	1985.492697	4526.738242	4303.96493	3037.888461
98.93700741	25.47190579	36.15287278	61.16016857	72.42422778	72.50493704
20.74485639	9.796886843	36.15287278	8.43588532	18.10605694	51.300663
105.3200401	63.67976448	146.1499113	93.84922418	357.801551	107.3893879
0	0	11.53815089	0	0	25.30832708
43.08547097	74.45634001	149.2267515	253.0765596	102.1698928	158.6900509
1.595758184	0.979688684	0	0	0	1.36801768
290.4279895	427.1442664	380.7589793	379.6148394	531.5421003	248.9792177
75.00063465	185.1611613	177.6875237	280.4931869	162.9545125	176.4742807
4.787274552	0.979688684	41.5373432	0	1.293289782	4.78806188
1336.351734	514.9439663	523.4320611	777.2402939	321.2919805	74.61852435
75.00063465	44.08599079	69.22890533	114.9389375	58.19804018	217.5148111
27.12788913	15.67501895	27.69156213	20.03522763	29.74566498	54.7207072
28.72364731	36.24848132	36.15287278	34.79802694	28.4523752	41.72453924
1370.75628	1564.562829	1994.561684	1719.86612	1537.72155	1771.582896
156.384302	74.45634001	103.0741479	75.92296788	129.3289782	73.18894588
3300.027924	1883.94134	3005.303701	3839.382306	1951.574281	2647.114211

47.87274552	139.1157932	183.0719941	94.90370985	108.6363417	268.8154741
6.383032736	9.796886843	17.69183136	48.50634059	6.466448909	38.30449504
97.34124922	254.7190579	271.5311509	94.90370985	122.8625293	197.6785547
90.95821649	228.2674634	213.8403965	198.243305	385.400355	176.4742807
185.1079493	886.6182593	2499.163482	524.0793755	999.7130013	2610.861742
443.6207751	1117.824789	611.5219971	782.4283634	452.6514236	599.8757526
1.595758184	0.979688684	0.769210059	0	1.293289782	3.4200442
51.06426189	475.1490119	337.683216	82.24988187	177.1807001	424.7694896
537.770508	394.8145398	427.6807929	527.2428325	433.2520769	487.6983029
0	0	1.538420118	1.054485665	0	1.36801768
39.8939546	1.959377369	2.307630178	0	29.74566498	2.73603536
186.7037075	504.5396724	320.7605947	163.4452781	120.2759497	157.3220332
73.40487646	276.272209	67.69048521	191.916391	157.7813534	185.3663956
920.7524722	489.8443422	166.9185829	156.0638784	1471.763772	417.9294012
1.595758184	1.959377369	0	5.272428325	5.173159127	2.73603536
1.595758184	3.918754737	13.07657101	1.054485665	47.85172192	6.15607956
0	6.85782079	0	1.054485665	1.293289782	1.36801768
49.4685037	41.14692474	66.92127515	41.12494093	33.62553433	110.8094321
27.12788913	61.72038711	67.69048521	99.12165251	62.07790952	107.3893879
764.3681701	348.7691716	372.2976687	287.8745865	413.8527302	283.1796597
384.5777223	700.4774093	260.7622101	422.8487516	438.425236	344.7404553
352.6625587	528.0522009	156.149642	722.3226805	638.8851522	181.2623426
750.0063465	1296.128129	694.5966835	636.9093416	1105.762763	1182.651284
4.787274552	3.918754737	12.30736095	0	0	4.78806188
237.7679694	153.8111234	153.8420118	207.733676	213.392814	169.6341923
31.91516368	313.500379	441.526574	80.14091054	106.0497621	355.0005879
5910.688313	5064.010809	1872.257284	2432.698429	2249.03093	2192.248332
154.7885438	36.24848132	206.1482959	33.74354128	42.6785628	77.29299892
1.595758184	40.16723606	23.84551184	75.92296788	31.03895476	25.99233592
0	0.979688684	0	1.054485665	0	2.05202652
4198.439782	4313.569277	1994.561684	1738.846862	3848.83039	3453.560633
63.83032736	4.898443422	340.7600562	1.054485665	28.4523752	181.9463514
4.787274552	6.85782079	19.99946154	9.490370985	12.93289782	15.04819448
1.595758184	16.65470763	11.53815089	11.59934231	0	13.6801768
172.3418839	244.9221711	266.1466805	271.0028159	190.1135979	182.6303603
0	0	0	22.47108952	2.625378257	1.381697857
0	0	0	2.826021582	3.427217922	1.545859978

102867.3598	131037.2803	14898.82964	46616.70228	102670.3959	21429.99696
9684.656419	7757.175003	4529.878039	9197.22397	13010.4952	5755.934388
178.7249166	275.2925203	513.0631095	775.0469637	693.203323	500.6944709
6.383032736	32.32972658	82.30547634	14.76279931	20.69263651	46.51260112
47.87274552	108.745444	176.1491036	98.06716684	113.8095008	211.3587315
1268.627756	616.2241824	343.0676864	495.6082625	613.0193565	431.609578
1101.073147	1231.468676	736.1340267	931.1108422	1425.205339	832.4387582
0	0.979688684	4.615260355	1.054485665	0	4.10405304
129.2564129	94.0501137	113.0738787	119.1568801	270.2975644	131.3296973
28.72364731	42.12661343	43.07576332	122.3203371	95.70344385	45.14458344
81.38366738	108.745444	105.3817781	91.74025285	89.23699494	143.6418564
0	0	0	1102.612391	4.487715543	5.533631515
454.7910824	189.0799161	179.2259438	133.9196795	231.4988709	153.901989
0	17.63439632	19.99946154	26.36214162	25.86579563	8.20810608
1.595758184	0.979688684	8.461310651	0	0	19.37113035
2527.680963	2372.805993	1897.641216	2663.63079	2192.12618	2215.504633
6.383032736	17.63439632	9.99973077	3.163456995	3.879869345	25.30832708
411.7056115	325.2566432	506.140219	226.714418	213.392814	306.4359603
0	0	0	0	1.293289782	0
1328.293155	477.5198585	637.2520736	547.2675153	475.5426527	593.1793061
7.97879092	22.53283974	17.69183136	400.7045527	55.61146062	12.31215912
38.29819642	43.10630211	94.61283728	41.12494093	62.07790952	67.71687516
660.8194216	502.1982165	391.743299	570.308027	464.5108909	420.7885582
480.3232134	518.255314	683.8277427	772.9379924	501.7964353	607.3998499
130.8521711	145.973614	131.5349201	197.1888193	155.1947738	106.0213702
517.0256516	0	0	1.054485665	9.053028472	25.30832708
67.02184373	77.39540606	16.9226213	74.86848221	60.78461974	39.67251272
142.0224784	133.2376611	136.1501805	176.099106	177.1807001	134.0657326
0	0.979688684	0.769210059	0	0	1.36801768
0	1.959377369	0	0	3.879869345	0.68400884
9.574549104	26.45159448	26.92235207	22.14419896	9.053028472	41.0405304
0	0	2.307630178	0	0	3.4200442
4.787274552	0	1.538420118	1.054485665	0	0.68400884
931.9227794	522.1740687	577.6767545	526.1883468	1077.310388	616.2919648
12.76606547	75.43602869	85.38231657	53.77876891	90.53028472	69.76890168
30.3194055	259.6175013	296.1458728	476.6275206	329.7888943	80.02903428
473.9401806	1849.652236	615.3680474	978.5626971	631.1254135	692.216946

357.4498332	216.3838397	266.1774489	242.5317029	113.6931047	177.0214878
0	0	2.307630178	6.32691399	28.4523752	19.83625636
1720.227322	569.1991256	237.6859083	181.3715344	451.3581338	86.18511384
0	0	0.769210059	0	0	0.68400884
296.8110222	251.7799919	331.5295355	171.8811634	258.6579563	316.6960929
1.595758184	18.614085	26.92235207	6.32691399	0	38.30449504
931.9227794	997.3230806	1042.27963	896.3128152	893.6632392	816.7065549
343.0880096	2330.67938	1056.894621	1271.709712	1200.172917	1885.128363
3.191516368	13.71564158	86.15152663	5.272428325	117.6893701	38.30449504
544.1535407	528.0522009	416.9118521	305.8008428	517.3159127	239.403094
92.55397467	38.20785869	72.30574557	21.0897133	32.33224454	77.29299892
159.5758184	365.4238793	502.2941687	733.9220228	425.4923382	495.906409
181.916433	129.3189063	96.1512574	85.41333886	281.9371724	151.1659536
949.4761195	382.0785869	444.6034142	788.7552774	850.9846764	321.4841548
3.191516368	0	2.307630178	1.054485665	0	2.73603536
70.2133601	87.19229291	96.92046746	70.65053955	84.06383581	101.9173172
4.787274552	0.979688684	82.30547634	361.6885831	47.85172192	61.5607956
266.4916167	719.0914943	782.2866302	506.1531192	518.6092025	781.8221041
98.93700741	15.67501895	0	36.90699827	85.3571256	4.78806188
1.595758184	2.939066053	18.46104142	10.54485665	6.466448909	8.20810608
0	0	0.769210059	0	0	0.38304495
1.595758184	4.898443422	3.076840237	6.32691399	3.879869345	2.73603536
454.7910824	457.5146156	433.8344734	382.7782964	421.6124688	288.6517305
0	14.69533026	6.922890533	0	2.586579563	2.73603536
89.3624583	48.98443422	61.53680474	50.61531192	107.3430519	38.30449504
81.38366738	34.28910395	39.22971302	68.54156822	59.49132996	64.9808398
212.2358385	148.91268	162.3033225	188.752934	239.2586096	170.3182012
813.8366738	1318.660969	839.9773847	1015.469695	907.8894268	1115.618418
38.29819642	81.3141608	46.15260355	46.39736926	71.130938	51.98467184
1.595758184	205.7346237	19.23025148	169.7721921	1.293289782	62.92881328
0	0.979688684	0	1.054485665	1.293289782	5.47207072
1.595758184	0	0	1.054485665	0	2.73603536
531.3874753	278.2315863	270.7619408	320.5636421	332.3754739	239.403094
30.3194055	0.979688684	35.38366272	23.19868463	7.75973869	25.30832708
4233.546462	1260.859337	1539.958539	2151.150757	4244.577064	1004.808986
162.7673348	76.41571738	136.9193905	147.6279931	122.8625293	109.4414144
17.18631564	8.817198159	30.75301817	15.23731786	23.27921607	27.0662298

0	0.979688684	0	1.054485665	1.293289782	0
3.191516368	1.959377369	5.384470415	3.163456995	9.053028472	12.99616796
0	0.979688684	0	0	1.293289782	2.05202652
162.7673348	275.2925203	260.7622101	314.2367282	209.5129446	296.1758277
0	3.918754737	2.307630178	1.054485665	0	17.100221
1.595758184	1.959377369	1.538420118	0	0	1.36801768
6.383032736	98.94855712	58.4599645	47.45185492	51.73159127	167.5821658
3.191516368	1.959377369	12.30736095	2.10897133	12.93289782	16.41621216
3.191516368	77.39540606	8.461310651	1.054485665	1.293289782	62.24480444
647.8778227	605.4476069	483.8331273	690.6881106	1524.788653	686.0608665
63.83032736	50.94381159	54.6139142	113.8844518	103.4631825	72.50493704
703.7293591	615.2444938	436.9113136	592.6209437	640.178442	471.2820907
274.4704076	349.7488603	356.1442574	490.3358342	606.5529076	331.0602785
386.1734805	235.1252842	322.2990148	256.2400166	263.8311155	180.5783338
103.724282	173.4048971	118.4583491	121.2658515	85.3571256	153.2179802
0	2.939066053	46.92181361	11.59934231	11.63960804	23.9403094
248.9382767	452.6161722	313.8377042	237.2592746	191.4068877	335.1643316
0	6.85782079	5.384470415	4.21794266	7.75973869	2.73603536
928.7312631	871.9229291	908.4370799	1124.081719	1020.405638	1164.867054
582.4517372	901.3135896	749.2105977	519.8614328	1751.114364	1013.701101
1081.924049	1202.078016	1373.039956	1751.50069	1641.184733	1937.797044
84.57518375	223.36902	240.7627485	123.3748228	118.9826599	331.0602785
370.2158987	422.2458229	569.9846539	622.1465423	360.8278491	487.6983029
0	0.979688684	4.615260355	0	0	2.05202652
44.68122915	17.63439632	44.61418344	95.95819551	32.33224454	71.8209282
1710.652773	1301.026573	885.3607782	601.056829	1564.880636	777.718051
67.02184373	31.3500379	55.38312426	36.90699827	45.26514236	49.24863648
0	6.85782079	4.615260355	7.381399655	1.293289782	2.05202652
0	0	1.538420118	1.054485665	1.293289782	0
561.7068808	384.0379643	193.0717249	765.5565928	350.4815309	347.4764907
486.7062461	773.9540606	1024.587799	405.976981	1149.734616	954.8763406
0	0	1.538420118	1.054485665	0	2.05202652
0	0	0.769210059	1.054485665	0	0.68400884
0	0.979688684	5.384470415	5.272428325	9.053028472	2.05202652
4072.374886	3788.456142	1181.506651	4413.022508	4126.887694	2185.408244
0	3.918754737	0.769210059	1.054485665	1.293289782	4.10405304
95.74549104	145.973614	246.916429	267.8393589	130.622268	261.9753857

0	0	0.769210059	0	1.293289782	12.77728513
534.4194158	71.98752452	0	0	71.22146828	0
0	0	0.769210059	0	0	1.36801768
135.6394456	769.0556172	110.7662485	134.9741651	228.9122914	150.4819448
10083.21298	11884.23115	7554.227381	11208.20195	14146.44335	11351.3319
1.627673348	1.959377369	3.138377042	4.21794266	3.905735141	2.05202652
75.00063465	307.6222469	189.9948846	143.4100504	214.6861038	284.5476774
0	0	0	1.054485665	0	0
569.6856717	1010.059034	643.8288196	579.9671157	642.7650215	1034.905375
7.97879092	10.77657553	2.307630178	12.65382798	5.173159127	8.89211492
1.595758184	4.898443422	24.6147219	0	2.586579563	8.89211492
121.277622	166.5470763	226.9169675	71.70502522	125.4491088	186.7344133
28.72364731	52.90318895	33.84524261	40.07045527	33.62553433	46.51260112
23.93637276	65.63914185	73.07495563	25.30765596	24.57250585	68.400884
0	24.49221711	13.84578107	3.163456995	5.173159127	8.89211492
630.3244827	250.8003032	232.3014379	192.9708767	212.0995242	216.1467934
3.191516368	2.939066053	0	1.054485665	190.1135979	8.89211492
0	77.39540606	0.769210059	1.054485665	2.586579563	0.68400884
68.61760191	62.7000758	119.9967692	135.5435874	82.77054603	64.9808398
143.6182366	21.55315106	33.84524261	32.68905561	11.63960804	64.29683096
7.97879092	5.878132106	92.30520711	186.6439627	40.09198323	136.1177592
118.0861056	333.0941527	186.1488343	253.0765596	259.9512461	229.8269702
0	0	1.538420118	1.054485665	0	1.36801768
1503.204209	440.8599079	676.1356421	713.8867952	684.1502945	519.8467184
1.595758184	2.292471521	0.769210059	1.054485665	0	0.574567426
1.595758184	1.959377369	0	0	0	0.68400884
156.384302	139.1157932	248.4548491	90.68576719	91.8235745	181.9463514
0	0	24.6147219	0	0	19.83625636
1720.227322	1441.122055	1718.415272	1043.940808	1694.209614	1837.247744
2392.041518	537.8490877	1105.354855	242.5317029	341.4285024	471.2820907
612.7711426	1012.9981	206.9175059	1143.062461	483.6903784	463.7579935
0	0	0.769210059	0	0	4.10405304
647.8778227	352.6879264	643.8288196	1273.818683	866.5041538	1004.124977
1434.586607	372.2817	311.530074	331.1084988	481.1037988	166.898157
0	7.837509475	11.53815089	9.490370985	1.293289782	2.73603536
310.2792213	287.352488	309.622433	385.1403443	207.5600771	328.2490022
6.383032736	3.918754737	28.46077219	1.054485665	2.586579563	11.62815028

92.55397467	581.9350785	556.9080829	569.4222591	439.7185258	771.5619715
30.3194055	25.47190579	25.38393195	14.76279931	23.27921607	27.3603536
493.0892788	386.9770303	361.5287278	175.0446204	162.9545125	300.2798807
11077.75331	6519.828194	5036.018258	6508.285524	6441.876403	4284.631374
194.6824984	363.4645019	164.6109527	119.1568801	140.9685862	269.4994829
271.1193155	11.13906034	113.2277207	23.72592746	330.2415458	81.28761054
513.8341352	172.4252084	398.4508107	218.2785326	496.6232762	216.8308023
0	1.959377369	1.538420118	2.10897133	2.586579563	4.78806188
114.8945892	76.41571738	109.9970385	63.2691399	63.37119931	66.34885748
2004.272279	1237.346808	676.9048521	2315.65052	2887.916083	640.2322742
130.8521711	573.1178803	449.2186746	311.0732712	413.8527302	426.1375073
0	0	22.30709172	0	0	50.61665416
90.95821649	152.8314348	99.9973077	131.8107081	111.2229212	134.0657326
1.595758184	0.979688684	0	1.054485665	0	0
31.91516368	125.4001516	229.2245976	56.94222591	43.97185258	303.6999249
145.2139947	186.14085	302.2995533	368.0154971	226.3257118	232.5630056
19.14909821	9.796886843	136.9193905	12.65382798	38.79869345	82.0810608
0	4.898443422	22.30709172	21.0897133	6.466448909	12.31215912
1642.035171	474.1693232	5400.623826	2876.636894	4282.082467	1550.64804
36.70243823	64.65945317	63.07522486	81.1953962	19.39934673	56.08872488
2767.044691	1012.9981	406.9121213	865.7327309	1028.165376	337.9003669
52.66002007	0	3.076840237	11.59934231	0	7.52409724
1035.647061	499.641229	521.5244202	456.5922929	518.6092025	354.3165791
225.0019039	656.3914185	440.7573639	341.6533554	305.2163885	368.6807647
663.8354045	265.4956335	496.1404882	247.8041313	395.7466732	315.3280752
223.4061458	156.7501895	188.4564645	328.9995275	125.4491088	166.898157
7.228784573	1.361767271	9.299749616	23.3041332	5.483548675	15.2123566
4346.845293	652.4726638	1092.255208	1093.501635	1132.908916	788.6621925
0	3.918754737	12.30736095	0	0	2.05202652
576.0687044	713.2133622	743.8261273	875.2231019	538.0085492	551.9951338
398.939546	131.2782837	101.5357278	65.37811123	153.901484	45.82859228
378.1946896	393.8348511	527.6781006	263.6214162	381.5204856	710.6851847
3.191516368	15.67501895	3.846050296	13.70831364	7.75973869	12.31215912
1273.415031	1030.632496	739.980077	698.0695102	1214.399105	1842.035806
3.191516368	0	0	0	135.7954271	0
151.5970275	154.7908121	179.2259438	179.262563	214.6861038	124.4896089
670.2184373	274.3128316	316.1453343	208.7881617	402.2131221	320.8001459

306.3855713	506.4990498	353.8366272	320.5636421	472.0507703	483.5942499
92.55397467	89.15167027	63.07522486	132.8651938	73.71751756	78.6610166
14.36182366	2.939066053	7.692100592	3.163456995	5.173159127	8.89211492
19.14909821	11.75626421	13.84578107	7.381399655	11.63960804	4.78806188
0	0	0.769210059	2.10897133	0	0
226.5976621	151.8517461	104.6125681	162.3907924	183.647149	163.4781128
1539.906648	1319.640658	1553.80432	1100.883034	1224.745423	1013.017092
0	2.939066053	63.84443492	0	0	7.52409724
11.17030729	5.878132106	22.30709172	14.76279931	10.34631825	22.57229172
54.25577826	121.4813969	43.07576332	44.28839793	76.30409712	71.06851847
38.29819642	53.88287764	21.53788166	26.36214162	27.15908542	14.36418564
3.191516368	0	10.76894083	3.163456995	1.293289782	6.8400884
0	0	0.769210059	2.10897133	1.293289782	0
23.93637276	77.39540606	106.9201982	64.32362556	58.19804018	132.0137061
13.80330829	13.56868828	8.115166125	11.51498346	10.86363417	5.041145151
231.3849367	367.3832566	243.0703787	428.12118	615.6059361	255.8193061
38.56947531	110.4403054	141.7884902	53.29370551	102.4932152	108.3675205
1282.98958	703.4164753	483.0639172	637.9638273	993.2465524	417.2453924
137.2352038	58.78132106	133.0733402	127.5927655	76.30409712	200.4145901
12.76606547	24.49221711	68.45969527	13.70831364	45.26514236	44.4605746
462.7539158	323.2972658	666.9051213	441.8294936	506.9695944	835.1747936
188.2994657	161.6486329	123.8428195	120.2113658	159.0746432	168.2661746
1413.331108	483.4567719	630.5445618	526.3676094	577.2081625	471.6514555
268.0873749	90.13135896	126.1504497	166.6087351	159.0746432	123.1215912
14.36182366	17.63439632	69.22890533	14.76279931	32.33224454	31.46440664
145.2139947	99.9282458	199.9946154	153.9549071	130.622268	289.3357393
175.5334002	340.9316621	510.7554793	256.2400166	173.3008308	257.8713327
1742.567937	284.1097185	569.9846539	172.9356491	962.2075976	385.7809857
255.3213094	388.9364077	359.9903077	542.0056318	398.3332528	442.5537195
450.0038079	999.282458	596.1377959	1049.213237	1257.077668	798.2383162
632.6223745	268.6698248	196.6562437	288.3596499	242.879821	190.2228584
0	0	1.538420118	0	0	0
2974.493255	605.4476069	943.8207427	857.2968456	1559.707477	1004.124977
234.576453	172.4252084	56.92154438	1037.613894	1117.402371	294.1238012
26432.13856	23372.43294	22598.62233	19496.38546	27863.92835	22929.34433
25.53213094	144.0142366	126.9196598	119.1568801	78.89067669	158.006042
55.85153644	276.272209	57.69075444	244.6406743	314.269417	96.44524643

0	0	0.769210059	0	0	0.68400884
16.37247897	70.18489735	71.50576711	64.87195811	97.28125738	103.0732921
106.4051557	134.4720688	145.5422353	84.50648119	89.26286074	76.91679405
1.595758184	13.71564158	0	12.65382798	5.173159127	3.4200442
6.383032736	6.85782079	2.307630178	2.10897133	1.293289782	2.05202652
692.5590518	270.3940769	433.0652633	308.9642998	256.0713768	400.8291802
0	0	0	0	6.466448909	1.36801768
47.87274552	35.26879264	52.30628403	42.1794266	21.98592629	43.09255692
38.29819642	48.98443422	72.30574557	61.16016857	89.23699494	48.56462764
0	0	0	0	2.586579563	0.68400884
2326.615432	954.2167785	1164.58403	888.9314156	1550.654448	1534.915837
54.25577826	30.37034921	33.07603255	32.68905561	27.15908542	31.46440664
943.0930867	346.8097943	393.0663403	595.7844007	530.2488105	363.208694
0	0.979688684	0	2.10897133	1.293289782	1.060213702
251.443617	439.3903749	63.48290619	251.8744459	388.8534387	46.04063502
421.2801606	301.7441148	476.9102367	247.8041313	306.5096783	311.2240222
1.595758184	0	1.538420118	2.10897133	0	0.68400884
39.8939546	100.9079345	247.6856391	29.52559862	170.7142512	93.02520223
0	0	3.846050296	6.32691399	1.293289782	0.68400884
1.595758184	213.5721332	152.3035917	0	412.5594404	99.18128179
633.515999	1816.342821	779.9790001	1405.629391	1046.271433	913.1518013
303.194055	228.2674634	236.1474882	593.6754294	112.516211	173.0542365
229.7891785	105.8063779	173.0722633	184.5349914	121.5692395	88.9211492
165.9588511	36.24848132	50.76786391	111.7754805	208.2196549	96.44524643
0	2.939066053	3.076840237	0	1.293289782	2.73603536
6.383032736	13.71564158	64.61364497	67.53980684	21.98592629	141.5556294
0	0	0	1.054485665	0	0.68400884
0	0	0	0	0	2.031506255
840.964563	1165.829534	946.8975829	1168.370117	867.7974436	817.3905638
998.9446232	423.2255116	396.1431805	453.4288359	760.4543917	333.1123051
373.4074151	373.2613887	346.1445267	548.3325458	597.4998792	398.0931449
54.25577826	90.13135896	168.457003	94.90370985	106.0497621	162.1100951
1.978740148	13.9213762	8.376697545	5.652043164	12.00172917	1.121774498
775.5384774	847.4307119	937.6670622	1631.289324	1026.872087	1038.325419
882.4542757	2866.56909	1640.725056	1463.626103	1382.526777	1552.016058
532.9832334	715.1727396	727.672716	1072.411921	457.8245827	720.2613085
19.14909821	23.51252842	17.69183136	23.19868463	19.39934673	56.08872488

247.3425185	171.4455198	177.6875237	127.5927655	118.9826599	295.4918189
16.99482466	10.54145024	4.084505414	10.67139493	4.823970886	5.177946919
606.3881099	211.6127558	250.7624793	139.1921078	213.392814	223.6708907
0	0	0	1.054485665	0	0
3.191516368	5.878132106	6.922890533	9.490370985	14.2261876	38.30449504
22.34061458	16.65470763	36.15287278	17.9262563	27.15908542	55.40471604
0	0.979688684	3.084532337	3.163456995	0	0
14.36182366	6.85782079	39.99892308	48.50634059	16.81276716	19.15224752
252.1297931	340.9316621	377.6821391	433.3936083	131.9155577	285.2316863
1.595758184	0	1.538420118	0	0	0
0	8.817198159	21.53788166	2.10897133	1.293289782	17.100221
1.595758184	9.796886843	5.384470415	18.98074197	24.57250585	8.89211492
1046.817369	796.4869004	720.7498255	3233.053049	1012.645899	800.9743516
1.595758184	0.979688684	0	0	0	1.36801768
4.787274552	1.959377369	4.615260355	2.10897133	1.293289782	7.52409724
0	0	2.307630178	0	0	820.810608
100.5327656	133.2376611	169.226213	202.4612477	115.1027906	212.7267492
0	13.71564158	5.384470415	3.163456995	23.27921607	279.0756067
242.2999227	209.3594718	179.010565	242.9113178	273.2979967	160.6394761
27.12788913	116.5829534	48.47561793	235.1503033	59.49132996	91.10313739
116.4903474	188.1002274	150.7651716	109.6665092	91.8235745	105.3373614
395.7480296	360.5254358	247.6856391	250.9675883	247.0183483	339.9523935
0	0.979688684	1.538420118	0	1.293289782	78.6610166
3.191516368	17.63439632	450.7570947	39.0159696	108.6363417	32.83242432
11.17030729	14.69533026	59.99838462	6.32691399	16.81276716	60.87678676
79.67620613	67.90222271	60.35991335	47.29368207	28.67223446	57.58670424
569.6856717	444.7786627	641.5211894	327.9450418	442.3051054	483.5942499
39.8939546	70.53758527	89.99757693	178.2080774	77.5973869	123.8056
0	0.979688684	1.538420118	4.21794266	3.879869345	0
193.0867403	302.7238035	143.8422811	200.3522763	139.6752964	161.4260862
78.19215101	0.979688684	18.46104142	1.054485665	5.173159127	9.576123759
292.0237477	67.59851922	176.1491036	106.5030522	87.94370516	98.49727295
132.4479293	405.5911153	932.2825918	407.0314667	540.5951288	1312.612964
23.93637276	52.90318895	10.76894083	33.74354128	43.97185258	4.78806188
20.74485639	8.817198159	392.1509803	10.54485665	3.879869345	181.8574303
6.383032736	7.837509475	8.461310651	4.21794266	5.173159127	9.576123759
0	0	2.307630178	0	0	2.73603536

68.61760191	160.6689442	107.6894083	120.2113658	73.71751756	82.0810608
0	0	2.307630178	0	19.39934673	0
54.25577826	72.49696264	86.92073669	167.6632207	63.37119931	101.2333083
1.595758184	22.53283974	6.153680474	1.054485665	10.34631825	18.46823868
60.63881099	101.8876232	189.2256746	208.7881617	253.4847972	247.6112001
3.191516368	1.959377369	3.846050296	17.9262563	1.293289782	5.47207072
271.2788913	228.2674634	180.7643639	195.079848	177.1807001	198.3625636
148.4055111	253.7393692	66.15206509	180.3170487	240.5518994	149.1139271
0	1.959377369	4.615260355	2.10897133	0	3.4200442
0	3.918754737	26.92235207	0	5.173159127	26.67634476
1.851079493	0	0.846131065	0	0	0
46.27698734	135.1970384	156.9188521	56.94222591	58.19804018	89.60515804
0	308.6019356	0	0	0	0
531.3874753	894.4557688	515.3707397	767.6655641	772.0939997	600.5597615
14.36182366	1.959377369	0	0	0	0
38.39394191	11.51134204	12.71504228	19.07564568	16.03679329	8.987876157
55.85153644	60.74069843	100.7665178	66.43259689	59.49132996	202.4666166
4094.460179	1414.631273	1303.395677	1020.900297	2133.643616	936.3123407
5752.708253	10074.13874	3916.817622	7593.351273	6488.434835	4822.262322
15.95758184	0.979688684	7.692100592	25.30765596	0	7.52409724
1.595758184	2.939066053	3.846050296	3.163456995	6.466448909	7.52409724
210.6400803	20.57346237	55.38312426	92.79473852	206.9263651	49.24863648
1509.587242	662.2695506	673.8280119	1433.046019	1026.872087	742.8336002
1.595758184	1.959377369	4.615260355	5.272428325	6.466448909	10.2601326
7.97879092	16.65470763	17.69183136	31.63456995	12.93289782	25.99233592
1091.498598	1898.63667	1680.723979	1340.25128	920.8223246	1218.219744
3.191516368	0.979688684	1.538420118	1.054485665	2.586579563	2.73603536
15.95758184	21.55315106	0	0	6.466448909	0
100.5327656	35.26879264	285.3846241	99.31145993	178.1894661	54.90538958
234.576453	151.8517461	281.5308817	115.9934231	122.8625293	180.5783338
4.787274552	43.10630211	59.99838462	25.30765596	14.2261876	54.03669836
1897.356481	1906.47418	2029.176136	3390.171413	2594.339302	3279.138379
0	0	0.769210059	0	0	0
67.02184373	93.07042501	63.84443492	59.05119724	89.23699494	140.905821
1.595758184	0.979688684	21.53788166	2.10897133	0	32.83242432
129.2564129	410.4895587	407.6813314	205.6247047	332.3754739	444.605746
14230.97148	5860.49771	6971.350767	3588.414718	4222.591137	4244.274852

0	7.837509475	0.769210059	0	0	2.05202652
354.2583168	410.4895587	350.759787	630.5824276	398.3332528	565.6753106
0	0	0	1.054485665	0	0.68400884
9.574549104	5.878132106	22.30709172	7.381399655	18.10605694	31.46440664
27.12788913	100.9079345	63.07522486	120.2113658	177.1807001	101.2333083
212.2358385	88.17198159	143.3961392	147.6279931	95.70344385	101.9173172
1563.84302	627.9804467	270.7619408	708.6143669	929.8753531	296.1758277
25.53213094	63.67976448	46.15260355	55.88774024	47.85172192	67.71687516
1026.072512	1068.840355	1198.429272	1598.600268	1229.918582	1203.855558
4.787274552	1.959377369	6.922890533	4.21794266	6.466448909	4.10405304
145.2139947	151.8517461	89.22836687	218.2785326	85.3571256	156.6380244
27.12788913	111.68451	9.230520711	27.41662729	47.85172192	16.41621216
148.9480689	283.6394679	93.28210388	125.6208773	74.10550449	210.154876
116.4903474	73.47665132	98.45888758	98.06716684	122.8625293	106.705379
11.17030729	117.5626421	83.84389646	127.5927655	292.2834907	32.83242432
0	0	14.61499113	0	7.75973869	22.57229172
132.0489897	233.8125014	114.8199855	117.2166265	103.9675656	106.5412169
2171.826888	5504.870717	3371.44769	9037.996634	21768.65361	4287.367409
9.574549104	0	0	0	6.466448909	0
116.4903474	892.4963914	386.9126598	684.3611966	484.9836682	934.3560754
19.65974083	0	0.769210059	0	1.293289782	6.176599825
27.12788913	74.45634001	81.53626628	76.97745354	53.02488105	67.03286632
2218.103876	8228.40526	11665.83976	1501.587587	5893.521535	7584.290018
3.191516368	0	0	0	2.586579563	0
0	5.878132106	10.76894083	0	1.293289782	15.73220332
0	0	0	1.054485665	135.7954271	0.68400884
0	0	0	0	0	0
277.661924	2360.070041	1056.894621	476.6275206	905.3028472	2138.895643
0	1.959377369	1.538420118	0	0	22.57229172
298.4067804	121.4813969	97.68967752	156.0638784	81.47725625	79.34502544
186.7037075	7.837509475	11.53815089	2.10897133	235.3787403	30.7803978
43.08547097	39.18754737	56.92154438	37.96148394	47.85172192	51.300663
266.8267259	314.107786	417.4349149	696.8673965	350.7660546	415.8705346
157.9800602	187.1205387	107.6894083	272.0573016	175.8874103	228.4589525
247.3425185	816.080674	1039.20279	827.771247	673.8039763	850.2229881
157.9800602	48.98443422	329.2219053	65.37811123	106.0497621	204.5186431
0	0	7.692100592	1.054485665	0	12.99616796

90.95821649	2.939066053	0.769210059	3.163456995	1.293289782	4.78806188
9.574549104	87.19229291	28.46077219	63.2691399	54.31817083	65.66484864
135.6394456	51.92350027	72.30574557	50.61531192	55.61146062	58.82476024
52.66002007	13.71564158	30.76840237	11.59934231	43.97185258	10.2601326
1898.952239	5081.645206	1949.17829	2348.339576	3004.312163	2715.515095
1350.011424	223.36902	249.2240592	161.3363067	351.7748206	162.7941039
36.70243823	58.86949304	140.0193071	31.63456995	37.47953788	78.99618093
1.595758184	0	0	1.054485665	59.49132996	0.68400884
67.02184373	155.7705008	147.6883314	144.4645361	188.8203081	222.302873
239.3637276	262.5565674	192.3025148	204.570219	347.8949513	173.7382454
57.44729462	328.1957093	236.9166982	189.8074197	121.5692395	319.4321283
1093.094356	78.37509475	195.379355	120.2113658	344.0150819	153.901989
3.191516368	0	0	502.9896622	0	1.36801768
3.191516368	94.0501137	5.384470415	3.163456995	7.75973869	3.4200442
568.0899135	80.33447211	766.902429	25.30765596	75.01080734	134.0657326
51.06426189	48.00474553	83.0746864	32.68905561	59.49132996	42.40854808
478.7274552	769.0556172	439.9881539	750.7937935	1138.095008	549.2590985
0	2.939066053	0	1206.510863	5.858602711	6.094518764
89.3624583	242.9627937	268.4543107	197.1888193	239.2586096	487.6983029
25.53213094	192.9986708	63.84443492	138.1376221	96.99673363	145.0098741
0	0	0	0	0	62.24480444
15.95758184	240.0237277	214.6096065	114.9389375	58.19804018	364.5767117
54.25577826	11.75626421	2.307630178	5.272428325	47.85172192	2.05202652
47.87274552	51.92350027	116.919929	56.94222591	45.26514236	96.44524643
0	0	3.076840237	0	0	11.17670444
1.595758184	0	0	0	1.293289782	0
43173.23767	20990.80975	17039.54123	32539.31865	31210.9623	22539.45929
210.6400803	183.201784	153.8420118	396.48661	337.548633	144.3258652
167.5067366	308.5431542	278.4540414	222.7179173	297.2367905	194.0054273
0	0	9.422823226	0	13.23035447	34.93233146
70.2133601	124.4204629	115.3815089	176.099106	210.8062344	213.4107581
111.7030729	44.08599079	35.38366272	43.23391226	49.14501171	37.6204862
1815.86111	804.9905981	935.8748028	1360.771571	1259.082267	449.947855
4.787274552	0	0.769210059	10.54485665	3.879869345	0.68400884
3.191516368	0	1.538420118	2.10897133	1.293289782	0.68400884
300.0025386	152.8314348	119.2275592	223.550961	107.3430519	64.29683096
47.87274552	128.3392176	80.76705622	132.8651938	78.89067669	178.5263072

70.2133601	62.7000758	62.3060148	56.94222591	53.02488105	68.400884
65.42608554	50.94381159	35.38366272	71.70502522	28.4523752	24.62431824
671.8141955	1879.042897	717.6729853	1119.863776	2148.154327	1180.599258
335.1092186	1349.031318	1294.58053	383.832782	579.3938222	603.9798057
895.2203412	578.0163238	859.2076362	704.3964242	693.203323	755.8297682
1.595758184	0	1.538420118	0	3.879869345	2.73603536
737.240281	254.7190579	310.7608639	298.4194432	239.2586096	301.6478984
0	0.979688684	6.922890533	2.10897133	1.293289782	5.47207072
38.29819642	134.2173498	89.22836687	157.1183641	146.1417453	215.4627846
4536.740517	113771.2469	68860.45371	25874.96925	39134.9488	111772.5165
79.7879092	44.08599079	19.99946154	68.54156822	56.9047504	31.46440664
834.5815302	431.0630211	226.1477574	489.2813485	472.0507703	352.9485614
4.787274552	29.39066053	14.61499113	2.10897133	9.053028472	25.30832708
204.2570475	232.1862182	220.763287	237.2592746	146.1417453	190.8384663
1019.68948	410.4895587	339.9908462	424.957723	1857.164127	203.1506255
0	0	1.538420118	0	0	0.68400884
23.93637276	0	46.92181361	1.054485665	2.586579563	0.68400884
568.0899135	521.1943801	362.2979379	919.5114998	848.3980968	435.7136311
205.8528057	208.6736898	170.7646331	284.7111295	219.8592629	311.908031
12.76606547	7.837509475	332.2987456	3.163456995	3.879869345	15.04819448
53.15470511	120.4723175	70.25195471	98.96347966	87.7238459	59.3172466
17.55334002	54.86256632	26.92235207	63.2691399	58.19804018	43.09255692
148.4055111	407.5504927	298.453503	172.9356491	181.0605694	364.5767117
226.5976621	145.973614	171.5338432	90.68576719	111.2229212	190.8384663
129.2564129	85.23291554	198.4561953	159.2273354	67.25106865	209.306705
791.7673381	230.5109505	559.1541763	340.7781323	370.7603146	335.7320589
1.595758184	0.979688684	3.846050296	5.272428325	9.053028472	2.05202652
170.7461257	544.7069085	378.4513491	122.3203371	478.5172192	804.3943958
7.97879092	32.32972658	29.99919231	26.36214162	23.27921607	208.6226962
0	0.979688684	0	1.054485665	0	2.73603536
138.830962	492.7834082	200.7638255	259.4034736	431.9587871	242.1391293
84.57518375	0.979688684	0	220.387504	12.93289782	8.89211492
450.0038079	470.2505685	385.3742397	355.3616691	545.7682879	370.7327913
150.0012693	47.02505685	77.69021598	55.88774024	28.4523752	100.5492995
4.787274552	7.837509475	6.922890533	4.21794266	2.586579563	7.52409724
1077.136774	153.8111234	201.5330355	180.3170487	311.6828374	102.601326
0	0	2.307630178	0	0	1.491139271

35.10668005	49.9641229	32.30682249	41.12494093	54.31817083	53.35268952
17.55334002	21.55315106	50.76786391	29.52559862	20.69263651	36.93647736
590.4305281	412.4489361	398.4508107	353.2526978	422.9057586	361.8406763
23.93637276	53.88287764	3.076840237	8.43588532	15.51947738	2.73603536
2226.082667	3206.521064	2020.714826	4145.183149	4084.209131	2253.125119
65.93672816	24.39424824	23.86858814	31.48694196	28.12905275	16.60089455
81.38366738	34.28910395	74.61337574	54.83325458	43.97185258	56.08872488
79.7879092	63.67976448	106.1509882	56.94222591	94.41015407	95.07722875
1367.564764	1170.727978	1324.579722	1672.414265	1967.093758	1894.704487
7.97879092	326.2363319	46.15260355	169.7721921	84.06383581	123.8056
371.8116569	222.3893313	246.916429	250.9675883	345.3083717	198.3625636
19.14909821	37.22817	36.15287278	24.25317029	43.97185258	43.09255692
22.34061458	0	1.538420118	0	1.293289782	0
0	1.959377369	1.538420118	0	1.293289782	3.4200442
517.0256516	251.7799919	266.1466805	205.6247047	344.0150819	216.1467934
231.3849367	198.8768029	239.9935385	344.8168124	222.4458425	382.3609415
4.787274552	17.63439632	21.53788166	5.272428325	5.173159127	23.9403094
151.5970275	171.4455198	113.0738787	176.099106	270.2975644	143.6418564
150.0012693	164.587699	266.1466805	126.5382798	250.8982177	250.3472354
0	0	0	0	0	2.05202652
318.6250366	352.5311762	204.5175705	341.7060797	196.8904364	273.6514166
485.1104879	465.3521251	415.373432	506.1531192	488.8635375	441.8697106
111.7030729	104.8266892	139.2270207	217.224047	125.4491088	93.70921107
550.5365735	491.2844845	377.1975367	411.2494093	385.400355	420.2003106
376.5989314	143.0345479	287.6845622	201.406762	209.5129446	289.3357393
3188.324852	2192.543276	1226.890044	2201.766068	4704.988226	1980.205592
6.383032736	1.959377369	1.538420118	4.21794266	3.879869345	0.68400884
368.6201405	703.4164753	216.9172367	491.3903199	1003.592871	173.0542365
11.17030729	31.3500379	19.99946154	20.03522763	18.10605694	29.41238012
6.383032736	0	0	0	1.293289782	1.36801768
1.595758184	0	2.307630178	0	0	8.89211492
4.787274552	2.939066053	0.77690216	2.151150757	0	0
89.3624583	86.21260422	119.2275592	67.48708256	135.7954271	133.3817238
0	0.979688684	2.307630178	0	1.293289782	3.4200442
118.0861056	95.02980238	77.69021598	209.8426473	38.79869345	125.8576266
145.2139947	240.0237277	299.222713	238.3137603	191.4068877	215.4627846
0	0	0.769210059	0	15.51947738	0

6.383032736	1.959377369	1.538420118	6.32691399	3.879869345	0.68400884
1.595758184	25.47190579	8.461310651	13.70831364	0	9.576123759
35.10668005	19.59377369	33.84524261	22.14419896	37.50540367	33.51643316
108.5115565	120.5017082	108.4586184	76.97745354	147.4350351	34.88445084
232.9806949	168.5064537	261.5314201	94.90370985	197.8733366	147.0619006
60.63881099	0.979688684	2.307630178	152.9004214	0	6.8400884
1.595758184	0.979688684	0	2.10897133	1.293289782	2.73603536
122.8733802	167.526765	93.07441717	73.81399655	104.7564723	141.5898299
0	0	0	0	0	0
0	2.939066053	0.769210059	0	0	21.20427404
3459.603743	6943.053706	2733.00334	13223.25024	15655.27281	3753.156505
295.215264	583.8944559	816.1318728	399.650067	523.7823616	680.5887958
162.7673348	925.8058067	123.8428195	900.5307579	488.8635375	339.2683846
140.4267202	170.4658311	172.3030533	207.733676	302.6298089	333.7963139
0	0.979688684	1.538420118	3.163456995	2.586579563	0
33.51092186	14.69533026	25.38393195	8.43588532	34.91882411	16.41621216
2578.745225	986.5465051	1230.736095	2349.394062	1830.005041	833.8067759
276.0661658	435.9614645	357.6826775	585.2395441	667.3375274	865.2711826
78.19215101	137.1564158	221.5324971	186.6439627	148.7283249	215.4627846
147.7672078	79.96219041	121.3351947	52.92463552	65.181805	86.78704161
0	0.979688684	0	0	0	4.10405304
1.595758184	5.878132106	3.846050296	0	0	4.78806188
4.787274552	9.826277504	10.76894083	7.381399655	11.63960804	9.576123759
322.3431532	47.02505685	63.84443492	49.56082625	32.33224454	56.77273372
1.595758184	0	3.076840237	1.054485665	1.293289782	0
416.492886	499.641229	423.8347426	443.9384649	406.0929915	650.4924068
413.3013697	12.7359529	6.153680474	1.054485665	6.466448909	17.78422984
818.6239484	730.8477585	925.3597012	1261.164855	2077.023389	603.2957968
35.10668005	51.92350027	20.7686716	47.45185492	28.4523752	26.67634476
102.1285238	111.68451	73.84416569	103.3395952	111.2229212	65.66484864
87.76670012	288.0284732	263.8390503	169.7721921	276.7640133	542.4190101
724.4742155	573.1178803	462.2952456	923.7294425	1117.402371	372.1008089
1.595758184	7.837509475	11.53815089	12.65382798	7.75973869	12.99616796
201.0655312	339.9519735	499.9865385	185.589477	254.778087	426.1375073
481.9189716	502.5802951	350.759787	743.4123938	619.4858055	578.6714786
67.02184373	3.918754737	3.846050296	2.10897133	1.293289782	12.31215912
17.55334002	69.55789659	68.45969527	80.14091054	84.06383581	67.03286632

236.8264721	352.7663015	403.0199184	373.3511945	182.5995843	388.3254986
4.787274552	0	0	0	0	0
157.9800602	166.5470763	202.3022456	180.3170487	94.41015407	194.2585105
94.14973285	162.6283216	169.9954231	203.5157333	128.0356884	184.6823868
22.34061458	37.22817	24.6147219	62.21465423	25.86579563	25.99233592
223.2306124	55.39159821	46.32182977	85.3078903	210.0690592	77.14935706
892.0288248	1840.835038	1633.802166	1498.42413	1152.321196	2233.288862
343.0880096	394.8145398	149.2267515	515.6434902	310.3895476	218.8828288
0	0.979688684	1.538420118	1.602818211	0	1.36801768
6.383032736	13.71564158	7.692100592	22.14419896	9.053028472	23.25630056
427.6631933	248.8409258	239.9935385	323.7270991	397.039963	282.4956509
151.5970275	177.3236519	129.2272899	133.9196795	196.5800468	58.1407514
4388.335006	3902.10003	1916.871468	3547.289777	5464.149328	1876.920257
106.9157983	106.7860666	176.9183136	115.9934231	165.5410921	111.4934409
0	1.959377369	1.538420118	0	2.586579563	0
17.55334002	15.67501895	13.07657101	20.03522763	15.51947738	15.04819448
225.0019039	510.4178045	235.3782781	499.8262052	580.687112	242.1391293
30.3194055	44.08599079	29.99919231	102.2851095	43.97185258	41.72453924
9.574549104	22.53283974	24.6147219	27.41662729	11.63960804	41.0405304
0	0	2.307630178	0	0	0.68400884
0	0	0.769210059	1.054485665	0	0
2586.708059	1778.134962	846.9002752	1807.38843	1810.605694	777.718051
35.10668005	63.67976448	141.5346509	43.23391226	36.21211389	92.34119339
0	6.85782079	4.615260355	3.163456995	11.63960804	5.47207072
370.2158987	230.2268408	243.0703787	138.1376221	248.3116381	311.2240222
0	0	0	0	0	0
320.747395	285.0894071	465.3720858	368.0154971	269.0042746	548.5750896
920.7524722	1200.118638	1087.663024	1258.001398	973.8472057	748.9896798
0	0	0	0	0	0
20.74485639	28.41097185	19.23025148	10.54485665	24.57250585	49.93264532
0	0.979688684	1.538420118	0	1.293289782	0
36.70243823	62.7000758	59.22917456	143.4100504	168.1276716	99.86529063
0	0	0	0	1.293289782	0.68400884
197.8740148	73.47665132	52.30628403	40.07045527	71.130938	34.200442
1.595758184	0.979688684	20.7686716	7.381399655	1.293289782	37.6204862
116.2190685	100.8295594	455.441584	71.45194866	82.77054603	208.2464913
0	0	111.5354586	0	0	6.15607956

157.9800602	203.7752463	310.7608639	108.6120235	186.2337286	614.9239471
0	0	0	0	0	3.4200442
486.7062461	215.5315106	329.2219053	386.996239	518.6092025	220.9348553
73.40487646	126.3798403	93.07441717	129.7017368	162.9545125	110.8094321
336.7049768	209.6533784	557.6772929	216.1695613	642.7650215	520.5307272
951.0718777	432.0427098	466.1412959	237.2592746	206.9263651	562.9392753
1.595758184	0	1.538420118	0	1.293289782	0.68400884
9.574549104	3.340738414	6.345982989	3.796148394	2.909902009	3.987771537
126.0648965	177.3236519	186.9180444	332.1629845	256.0713768	336.5323493
39.8939546	99.9282458	148.4575414	125.4837941	109.9296314	166.2141481
375.0031732	303.7034921	443.8342042	883.6589872	818.6524318	320.1161371
644.5426881	229.2471521	407.1813449	208.0605666	162.9545125	323.4472602
4.787274552	17.63439632	300.7611332	28.47111295	117.6893701	73.18894588
55.85153644	144.0142366	609.983577	118.1023945	166.8343818	751.0417063
0	0	0	0	0	0.978132641
0	0	0.769210059	0	3.879869345	3.4200442
205.8528057	231.2065295	575.3691243	498.7717195	355.65469	329.008252
842.5603211	704.396164	737.6724468	598.9478577	746.2282041	830.3867317
0	0	0	0	1.293289782	0.68400884
0	0.979688684	0	0	0	0.68400884
0	0.979688684	0.769210059	0	1.293289782	0.68400884
0	0.999282458	0.78459426	0	1.345021373	1.40905821
0	1.626283216	0	1.054485665	1.293289782	0
1.595758184	0.979688684	0.769210059	1.054485665	0	0.68400884
3.191516368	0	0.769210059	0	0	0.68400884
186.7037075	250.8003032	210.7635562	304.7463572	169.4209614	254.4512885
3703.754745	617.2038711	5492.929033	1249.565513	4031.18425	2919.349729
424.4716769	280.1027917	147.6883314	262.5669306	359.5345593	96.44524643
4.787274552	33.30941527	65.38285503	12.65382798	15.51947738	70.45291052
47.87274552	51.92350027	40.76813314	37.96148394	65.95777887	49.93264532
0	0	0.769210059	1.054485665	1.293289782	0
7.97879092	0.979688684	7.692100592	5.272428325	10.34631825	4.78806188
4.787274552	0	9.99973077	6.32691399	1.293289782	2.05202652
0	4.898443422	17.69183136	6.32691399	1.293289782	43.09255692
1.595758184	0.979688684	0	1.054485665	1.293289782	0
127.6606547	76.91535861	78.45942604	122.3203371	59.49132996	175.106263
3.191516368	2.939066053	3.076840237	21.0897133	14.2261876	0.68400884

33.51092186	5.878132106	4.615260355	40.07045527	0	7.52409724
0	3.722817	1.284580799	0	1.293289782	0.861851138
507.4511025	700.4774093	388.4510799	752.9027648	465.5843214	414.509357
0	3.918754737	0	2.10897133	1.293289782	1.36801768
3314.389748	2425.709182	2192.248669	1975.05165	2887.916083	1837.247744
23.93637276	1.959377369	46.92181361	0	1.293289782	0
90.95821649	259.6175013	151.5343817	210.897133	173.3008308	167.5821658
118.0861056	268.4346995	337.683216	248.8586169	267.7109848	282.4956509
245.7467603	85.23291554	84.61310651	72.75951088	82.77054603	81.39705196
124.4691384	297.82536	88.45915681	347.9802694	494.0366966	350.2125261
529.7917171	485.9255874	602.2914764	1053.431179	579.3938222	376.8888708
102.3519299	76.74881153	109.3278257	92.90018708	89.59911608	84.6118935
0	0	0	1.054485665	0	1.36801768
488.3020043	190.0596048	265.3774704	199.2977907	266.417695	175.106263
0	0	1.538420118	0	0	0.68400884
0	84.25322685	36.15287278	39.0159696	20.69263651	8.89211492
0.526600201	0	5.799843847	2.130061043	0	0
1.595758184	0	0	0	16.91623035	3.4200442
106.9157983	56.82194369	47.69102367	43.23391226	28.4523752	56.08872488
587.2390117	691.6602111	581.5228048	366.9610114	757.8678121	397.409136
67.02184373	19.59377369	96.1051048	12.65382798	10.34631825	42.37434764
390.9607551	258.6378127	416.142642	198.243305	214.6861038	492.4863648
27.12788913	29.39066053	26.92235207	21.0897133	31.03895476	71.8209282
209.0443221	138.1361045	75.3825858	122.3203371	152.6081942	166.898157
0	0.979688684	0.769210059	0	0	2.73603536
3.191516368	3.918754737	9.230520711	9.490370985	18.10605694	7.52409724
0	1.734048971	0	0	0.41385273	0
3.191516368	35.26879264	43.07576332	5.272428325	24.57250585	48.56462764
25.53213094	59.76100974	69.22890533	93.84922418	55.61146062	58.1407514
55.85153644	39.18754737	201.5330355	7.381399655	10.34631825	79.34502544
7.97879092	134.2173498	88.45915681	72.75951088	50.43830149	216.1467934
119.6818638	385.9973416	202.3022456	296.3104719	165.5410921	188.7864398
333.5134605	166.5470763	446.1418344	111.7754805	252.1915074	339.2683846
0	3.918754737	3.076840237	0	0	2.05202652
181.916433	499.641229	312.299284	429.1756656	319.4425761	435.7136311
0	0	1.538420118	0	0	3.4200442
111.7030729	142.0548592	172.3030533	197.1888193	215.9793936	123.1215912

35.10668005	3.918754737	3.076840237	64.32362556	32.33224454	0
105.3200401	43.10630211	88.45915681	78.03193921	45.26514236	67.71687516
0	0.979688684	1.538420118	0	0	4.10405304
0	0.979688684	1.007665178	2.10897133	0	0
70.2133601	79.35478343	32.30682249	18.98074197	59.49132996	24.62431824
134.0436875	824.8978722	1033.81832	578.9126301	964.7941772	767.4579184
0	0	2.307630178	0	0	1.36801768
159.5758184	169.4861424	329.9911154	182.42602	322.0291557	280.4436244
0	10.77657553	2.307630178	5.272428325	2.586579563	17.100221
1.595758184	0	0	1.054485665	51.73159127	0
129.2564129	284.1097185	161.5341124	383.832782	236.6720301	258.5553415
28.72364731	26.45159448	36.92208284	13.70831364	7.75973869	48.56462764
60.63881099	40.16723606	78.45942604	61.16016857	56.9047504	112.1774498
1.595758184	2.939066053	7.692100592	10.54485665	6.466448909	15.73220332
0	0	0	1.054485665	0	2.73603536
464.3656315	265.4956335	299.9919231	525.1338612	315.5627067	348.1604995
44.68122915	199.8564916	118.4583491	120.2113658	113.8095008	103.9693437
1.595758184	16.65470763	32.30682249	11.59934231	15.51947738	21.20427404
1125.00952	889.5573254	699.2119438	678.0342826	1029.458666	406.3012509
0	0	0.769210059	0	0	8.89211492
14.36182366	60.74069843	29.99919231	80.14091054	65.95777887	25.99233592
2.60108584	0.7445634	15.74572991	5.567684311	10.42391564	6.901649195
1.595758184	594.6710314	20.7686716	14.76279931	87.94370516	469.9140731
4659.613897	2425.709182	2509.163213	2881.909322	4942.953546	1421.370369
0	0	0.769210059	0	0	1.36801768
0	4.898443422	5.384470415	4.21794266	5.173159127	6.8400884
0	9.796886843	5.384470415	3.163456995	2.586579563	6.8400884
277.5821361	256.5706695	180.2951458	250.7988706	268.0213744	364.371509
92.55397467	280.1909637	1564.57326	413.3583807	305.2163885	149.1139271
1857.462526	939.5214483	649.21329	782.4283634	691.9100332	857.0630765
6.383032736	0	0	0	2.586579563	0
96.11251542	24.34526381	59.12148515	142.3871993	154.8714514	149.4764518
1.595758184	3.918754737	9.99973077	11.59934231	6.466448909	12.31215912
0	0	7.476721776	0	21.3522143	0
0	0	0	1.054485665	0	0.68400884
877.6670012	1680.166094	1790.721018	1813.715344	1489.869829	2807.172279
986.1785577	1018.876232	1179.968231	1562.747755	4045.410437	709.317167

540.9620244	909.1510991	742.2877072	656.9445693	668.6308172	821.4946168
176.8897947	100.0164178	55.16005335	84.92827546	103.527847	67.22438879
309.5770877	675.0055035	876.8994675	433.3936083	785.0268975	705.8971228
87.76670012	119.5220195	119.2275592	132.8651938	73.71751756	137.4857768
116.4903474	165.5673877	174.6106834	184.5349914	172.007541	183.3143691
0	25.47190579	0.769210059	0	3.879869345	2.73603536
25.53213094	21.55315106	33.07603255	51.66979758	46.55843214	201.0985989
54.25577826	278.2315863	432.2960533	123.3748228	103.4631825	561.5712576
1018.093721	1334.335988	1018.434118	1301.235311	1519.615494	1221.639788
337.2794498	499.298338	554.854292	360.9398983	369.9067434	563.9379282
0	55.84225501	0.769210059	11.59934231	0	2.05202652
108.5115565	19.59377369	268.4543107	87.52231019	266.417695	35.56845968
0	4.898443422	39.22971302	7.381399655	27.15908542	19.15224752
204.2570475	59.76100974	91.53599705	85.41333886	102.1698928	75.2409724
0	0.979688684	10.76894083	13.70831364	1.293289782	0.68400884
210.6400803	194.9580482	206.9175059	195.079848	217.2726833	261.9753857
86.17094193	31.3500379	33.84524261	10.54485665	10.34631825	52.66868068
256.9170676	223.36902	203.8406657	147.6279931	234.0854505	178.5263072
159.5758184	215.5315106	446.1418344	164.4997637	201.753206	235.2990409
25.53213094	5.878132106	49.22944379	6.32691399	5.173159127	16.41621216
66.20800705	292.9269166	159.2264823	370.8520635	280.6438826	246.5578265
209.0443221	186.14085	238.6397288	257.2945023	200.4599162	284.5476774
3.191516368	27.43128316	38.46050296	9.490370985	28.4523752	125.1736177
145.2139947	243.9424824	171.5338432	228.8233893	195.286757	151.1659536
7.97879092	0	0	1.054485665	0	5.47207072
0	3.918754737	26.92235207	1.054485665	1.293289782	22.57229172
15.95758184	63.67976448	76.15179586	23.19868463	42.6785628	139.5378034
0	2.20429954	1.615341124	0	0	0
3.191516368	33.30941527	16.15341124	388.0507247	53.02488105	18.46823868
0	0	1.538420118	0	2.586579563	1.36801768
16588.73612	44922.29224	14563.86942	16438.29267	17092.66094	8400.79137
300.0025386	73.47665132	153.8420118	48.50634059	51.73159127	91.65718455
0	0	0	0	1.293289782	0
716.4954246	138.1361045	123.8428195	192.9708767	126.7423986	67.71687516
194.6824984	366.4035679	208.4559261	473.4640636	222.4458425	231.8789967
178.7249166	183.201784	131.5349201	145.5190218	188.8203081	208.6226962
619.1541754	651.4929751	620.7525178	607.383743	734.588596	883.7394212

186.7037075	237.0846616	126.1504497	349.0347551	292.2834907	203.1506255
454.7910824	234.1455956	115.3815089	354.3071834	168.1276716	60.87678676
2294.700269	7821.834456	2170.710787	2204.929525	6558.272483	1881.02431
4.787274552	17.63439632	57.69075444	18.98074197	12.93289782	38.30449504
0	1.959377369	3.076840237	1.054485665	1.293289782	4.78806188
247.3425185	192.9986708	143.073071	228.8233893	137.0887169	81.39705196
0	0.979688684	0	0	0	4.10405304
221.8103876	274.3128316	333.8371657	359.5796118	244.4317688	494.5383913
17.55334002	21.55315106	56.15233432	46.39736926	42.6785628	60.87678676
164.3630929	184.1814727	145.3807012	148.6824788	111.2229212	135.4337503
2529.276722	2375.745059	1278.427118	2645.704533	4304.068394	824.2306522
587.2390117	206.7143124	152.3035917	74.86848221	126.7423986	731.2054499
995.7211916	726.821238	716.0807204	833.0436753	1043.684854	1244.20524
244.1510021	322.3175771	431.5268432	618.9830853	570.3407938	443.9217371
212.2358385	0	58.4599645	9.490370985	11.63960804	21.88828288
7.97879092	37.22817	48.46023373	32.68905561	19.39934673	51.300663
1134.584069	1637.089182	1967.716253	1976.169405	2828.424753	2320.889875
0	34.28910395	18.46104142	4.21794266	10.34631825	69.76890168
12.76606547	26.45159448	38.67588178	36.90699827	33.62553433	27.3603536
386.1734805	472.2099458	533.8317811	544.1146031	547.0615777	398.7771537
14.36182366	13.71564158	18.46104142	13.70831364	3.879869345	12.31215912
30.3194055	29.39066053	43.07576332	16.87177064	56.9047504	53.35268952
320.747395	274.3128316	200.7638255	362.7430687	382.8137754	183.3143691
85.08582637	257.2074672	180.0720749	106.0601682	138.7441278	260.1217217
406.9183369	69.55789659	124.6120296	130.7562225	100.876603	102.601326
137.2352038	45.06567948	81.53626628	31.63456995	86.65041538	91.65718455
73.40487646	99.9282458	160.7649024	69.15316991	35.47493871	149.1481276
0	1.283392176	0.330760325	0.938492242	0	0
0	8.817198159	7.692100592	7.381399655	1.293289782	15.04819448
698.9420846	193.9783595	602.2914764	745.5213651	516.0226229	298.9118631
100.5327656	321.3378885	135.3809704	76.97745354	80.18396647	100.5492995
51.06426189	213.5721332	208.4559261	151.8459358	177.1807001	170.3182012
20.74485639	16.65470763	25.38393195	13.70831364	23.27921607	23.30418118
3.191516368	3.918754737	3.846050296	5.272428325	0	5.47207072
9.574549104	80.33447211	349.2213669	57.99671157	73.71751756	156.6380244
9.574549104	10.8745444	21.53788166	5.272428325	5.173159127	32.20313619
103.724282	168.5064537	159.9956923	197.1888193	175.8874103	123.8056

31.91516368	71.99732141	92.84365415	117.3431648	104.7564723	124.0655234
57.44729462	17.63439632	26.15314201	10.54485665	11.63960804	15.04819448
3.191516368	27.43128316	14.61499113	18.98074197	18.10605694	34.200442
0	0	6.922890533	2.10897133	0	1.36801768
0	0	2.307630178	1.054485665	0	2.05202652
263.3001004	76.41571738	70.76732545	49.56082625	69.83764821	149.797936
82.2453768	17.7225683	9.87665716	43.1284637	28.80156344	8.043943958
3.191516368	0.979688684	5.384470415	6.32691399	0	44.4605746
2.34576453	1.763439632	0	0	2.198592629	0
660.1970759	539.7398869	538.9931806	439.6150737	492.4847489	327.5102727
0	14.69533026	0.769210059	5.272428325	7.75973869	2.05202652
0	0	24.6147219	1.054485665	1.293289782	14.36418564
1787.249166	1132.520119	1243.043456	654.8355979	1506.682596	506.1665416
220.2146294	214.5518219	371.5284586	302.6373858	245.7250585	261.2913769
1.595758184	0.979688684	3.846050296	3.163456995	2.586579563	6.8400884
108.5115565	335.05353	472.2949764	210.897133	221.1525527	422.0334543
150.0012693	305.6628695	186.9180444	95.95819551	208.2196549	191.5224752
5326.640818	3446.544791	2435.319048	1364.50445	2909.902009	2805.120253
31.91516368	46.04536816	47.69102367	88.57679586	31.03895476	72.50493704
17.55334002	143.0345479	64.61364497	28.47111295	59.49132996	63.61282212
494.685037	147.9329913	18.46104142	73.81399655	23.27921607	35.56845968
127.6606547	241.0034163	227.6861775	447.1019219	270.2975644	512.3226211
206.108127	327.2160206	273.069571	661.1625119	455.2380032	473.3341173
3.191516368	1.959377369	8.461310651	31.63456995	5.173159127	20.5202652
0	0	20.7686716	3.163456995	0	4.78806188
57.44729462	84.25322685	128.4580799	145.5190218	112.516211	145.6938829
372.1148509	145.973614	173.0722633	119.1568801	154.664525	137.4857768
6.383032736	0	0.769210059	0	0	0
22.34061458	13.71564158	67.69048521	27.41662729	14.2261876	41.72453924
832.985772	1036.510628	1890.718326	1147.280403	1099.296314	1599.212668
6.383032736	1.959377369	0	3.163456995	10.34631825	0
0	16.65470763	19.99946154	0	3.879869345	15.73220332
229.7891785	237.0846616	170.7646331	191.916391	256.0713768	179.2103161
167.5546093	294.886294	348.4521568	498.7717195	409.9728608	360.4726587
14.36182366	0	0	5.272428325	0	0
0	0	0	7.381399655	1.293289782	0
0	0	0	9.490370985	1.293289782	0.68400884

936.710054	456.5349269	543.8315119	327.9450418	333.6687637	402.1971979
0	0	119.2275592	1.054485665	2.586579563	10.94414144
138.830962	290.9675392	266.9158906	139.1921078	161.6612227	389.8850388
124.4691384	516.2959366	490.7560178	426.0122086	500.5031455	831.0707406
1195.22288	516.2959366	568.4462338	600.0023434	543.1817083	219.5668376
234.576453	84.25322685	86.92073669	122.3203371	340.1352126	57.45674256
17.55334002	130.298595	19.99946154	5.272428325	51.73159127	123.1215912
17.85653408	68.91130206	42.30655326	34.79802694	32.33224454	51.57426653
0	0.979688684	6.922890533	2.10897133	6.466448909	1.36801768
3.191516368	2.939066053	0	1.054485665	0	0.68400884
2449.488812	158.7095669	1361.501805	137.0831364	6956.605736	72.50493704
248.9382767	808.2431646	976.1275652	1013.360724	609.1394872	794.818272
550.5365735	454.5755495	574.5999142	1130.408633	977.727075	553.3631515
27.12788913	0	53.84470415	2.10897133	1.293289782	4.10405304
199.469773	419.3067569	109.9970385	935.3287848	457.8245827	274.9715537
95.74549104	197.8971142	143.8422811	342.7078411	77.5973869	135.4337503
110.1073147	103.8470005	134.6117604	183.4805057	73.71751756	205.202652
89.3624583	133.2376611	110.7662485	126.5382798	179.7672797	123.1215912

HCC12	HCC13	HCC14	a.value	m.value	p.value
0	0	0	1.294690506	-8.28469546	1.23E-09
182.6119431	544.1413896	143.9798939	5.999342839	4.380870676	1.63E-08
93.91471359	245.7412727	51.17357676	4.472520079	4.652669425	2.00E-08
0	4.335578168	1.821432393	3.759645283	-5.16498639	4.52E-08
0	0	0	0.097881303	-7.06838131	9.17E-08
0	0	0	0.338773826	-6.35146693	1.30E-07
0	0	0	1.673840567	-6.13162629	1.61E-07
59.47931861	139.545937	27.75516027	4.326797296	4.235169662	2.04E-07
56.34882815	175.5294805	36.42864786	3.933336398	5.972038176	2.10E-07
1225.065264	342.282487	231.5821185	11.95608785	-4.90190044	2.48E-07
37.56588544	107.9506305	21.68371896	3.5308893	5.117583544	3.07E-07
36.52238862	124.6259312	26.88781152	4.125736827	4.135413687	3.84E-07
72.00128042	150.0777058	52.04092551	4.349088412	5.32516033	4.47E-07
28.1326742	75.43379425	33.80058112	4.009136958	3.700857803	4.87E-07
56.34882815	351.9366084	62.44911062	5.148570561	4.054445247	4.94E-07
32.34840135	87.76474025	16.47962641	3.519102351	4.680048947	6.06E-07
49.04435043	24.57412727	35.5612991	6.44758319	-2.64736472	7.86E-07
32.34840135	75.47767662	12.14288262	3.097157137	4.709895128	8.06E-07
67.82729315	114.9718097	43.36743793	4.796089649	4.110710158	1.04E-06
0	0	0	0.02179236	-5.3938857	1.16E-06
7.32534766	8.802803447	9.575530295	6.195099785	-3.83575646	1.19E-06
27.13091726	58.80237597	22.55106772	2.927234298	5.698346006	1.24E-06
32.34840135	47.39295974	26.02046276	3.663367043	4.779360968	1.33E-06
16.69594908	39.49413311	6.938790069	2.047359625	5.812690703	1.46E-06
30.26140771	121.9929889	24.28576524	4.045620308	3.940028932	1.57E-06
29.21791089	115.8494571	33.82660158	4.395114572	3.250345795	1.70E-06
0	0	0	-0.06098644	-6.58856321	1.76E-06
18.78294272	47.39295974	8.673487586	-8.70511228	12.39747767	1.82E-06
12515.70083	7651.330055	3716.58943	14.36533454	-2.83537626	1.84E-06
34.43539498	190.4143804	21.64035153	4.246456605	3.837449633	1.89E-06
28.17441408	108.8195014	24.28576524	3.761599984	4.609547931	2.03E-06
32.70319027	149.7441998	23.41841648	3.867498413	4.540102547	2.14E-06
315.1360389	266.8048104	226.378026	8.565012312	-1.46189541	2.34E-06
37.56588544	97.41886168	32.95925283	3.869363156	4.051327605	2.43E-06

35.4788918	82.49885584	22.55106772	3.810174381	3.376390388	2.67E-06
88.6972295	228.1883247	39.89804289	4.425673398	4.026433671	2.73E-06
252.5262299	28.96236428	32.09190407	7.689530629	-2.7176437	2.78E-06
32.34840135	82.49885584	23.41841648	4.165269406	3.416397745	2.81E-06
11.47846499	0	7.806138827	4.034085781	-3.68310348	2.84E-06
57.39232497	169.3859487	28.62250903	4.506123593	4.073956268	2.84E-06
27.13091726	168.5083013	19.08167269	3.886055101	4.589393116	2.93E-06
48.00085361	128.1365208	44.23478669	5.155684065	3.039470187	2.97E-06
35.4788918	49.14825454	1.734697517	6.539804153	-4.56594986	3.00E-06
59.47931861	172.8965383	52.90827427	4.797790887	3.689477586	3.09E-06
58.43582179	112.3388675	40.76539165	4.256825518	3.425167874	3.45E-06
774.2746387	227.3106772	448.4193082	9.808848267	-1.49338044	4.02E-06
0	0	0	-0.29524462	-6.12004686	4.03E-06
74.08827405	402.8401578	134.4390576	4.302924827	7.617861626	4.03E-06
117.9151404	119.3600467	48.57153048	4.70690325	4.507886025	5.14E-06
49.04435043	113.2165149	29.48985779	4.946774915	3.067839356	5.61E-06
504.0089629	412.4942792	161.3268691	9.636914753	-1.71431282	5.63E-06
24.00042681	94.78591947	8.673487586	3.329487206	4.00690779	5.67E-06
14.60895545	21.06353766	4.336743793	-8.70511228	11.71224549	6.23E-06
394.4417971	169.3859487	215.9698409	8.463452411	-1.13071099	6.36E-06
4.173987271	0.877647403	0	2.708765197	-3.95118308	6.40E-06
17.7394459	60.55767077	12.14288262	3.076789127	4.199343931	7.35E-06
17.7394459	0.877647403	9.540836344	5.069005775	-3.34899762	7.39E-06
108.2732298	221.2987925	85.65068991	6.324817312	2.053417329	7.70E-06
218.0908349	167.6306539	367.7558736	9.286096448	-1.95596884	7.88E-06
0	0	0	-1.2506732	-5.17257289	7.93E-06
18.78294272	36.86119091	5.204092551	2.51220327	5.369170405	9.42E-06
74.08827405	251.0071571	170.0003567	6.628284442	2.609919118	9.74E-06
10.43496818	43.88237013	9.540836344	2.698531419	3.509754704	1.12E-05
65.74029951	315.0754175	74.59199324	5.667007163	2.78000544	1.23E-05
401.7462748	89.52003506	108.4185948	8.534588798	-1.95892515	1.23E-05
36.52238862	120.2376941	8.673487586	3.886449703	3.798580786	1.24E-05
0	0	0	0.322689902	-5.43579318	1.26E-05
3.130490453	0	0.867348759	2.605369176	-3.78789053	1.31E-05
0	0	0	-0.6346291	-7.44127789	1.32E-05
15.65245226	48.27060714	12.14288262	3.509883489	2.928308814	1.39E-05
180.5249495	150.9553532	93.67366593	6.521222016	2.672704837	1.41E-05

0	0	0	-1.30862085	-4.87875863	1.42E-05
1542.288297	1222.562832	355.612991	10.66165273	-1.4257264	1.49E-05
566.618772	201.8589026	119.6941287	9.297046021	-3.11255856	1.57E-05
10.43496818	40.37178052	8.673487586	1.750828645	5.016346156	1.59E-05
1.043496818	0.877647403	0.867348759	2.40588603	-3.83157212	1.61E-05
26.08742044	81.62120843	17.34697517	3.445204884	4.826127821	1.63E-05
141.9155672	84.25415064	127.5002675	8.071466631	-1.8460233	1.65E-05
0	1.755294805	0	1.978413243	-5.47972252	1.83E-05
21.91343317	37.73883831	14.7449289	2.698842694	3.992190307	1.88E-05
13.56545863	42.12707532	3.469395034	1.945418795	5.6762866	1.90E-05
181.5684463	38.61648571	182.1432393	7.838937602	-1.75974926	2.10E-05
102.2626881	924.1627148	68.52055193	5.853548623	4.444851717	2.17E-05
28.17441408	87.76474025	7.806138827	3.84693934	3.597646289	2.26E-05
1056.018779	1332.268757	2024.392002	12.28724598	-2.1779278	2.26E-05
2953.095994	4841.103072	3175.363805	12.66779256	-2.16417471	2.36E-05
27.13091726	87.76474025	15.61227765	3.351640378	3.856464987	2.37E-05
323.4840135	495.8707824	215.1024921	7.794188348	1.332095971	2.46E-05
628.1850842	492.3601928	856.9405735	9.95985281	-2.39590281	2.70E-05
36.52238862	39.49413311	13.87758014	6.433273478	-2.2351813	2.74E-05
9.391471359	84.25415064	16.47962641	3.057111115	3.7510254	2.74E-05
6.260980906	9.654121428	5.204092551	1.851442415	3.443934921	2.80E-05
7.304477724	1.755294805	0	4.433811893	-4.86267895	2.89E-05
18.78294272	74.60002921	13.87758014	3.867998265	3.087281241	2.91E-05
28.17441408	103.5623935	15.61227765	4.116440943	3.468690579	2.95E-05
14.60895545	45.63766493	15.61227765	3.870041401	2.807423515	2.96E-05
33.39189817	52.65884415	39.89804289	6.889765359	-2.20892478	3.18E-05
6012.628663	1989.626661	1573.370648	12.17973674	-2.26437437	3.18E-05
0	0	0	-0.89292854	-6.1372085	3.41E-05
7.304477724	72.84473441	27.75516027	3.396918556	4.849642057	3.59E-05
0	0	0	-0.26118965	-4.94143856	3.76E-05
98.08870086	214.1459662	86.73487586	6.147917869	1.793737593	3.79E-05
203.4818794	314.1977701	160.4595203	7.14485519	2.614126298	3.81E-05
40.69637589	98.29650908	25.153114	4.543446135	3.51355735	3.82E-05
10.43496818	37.73883831	8.673487586	3.099960268	3.587645252	3.84E-05
161.7420067	26.32942208	125.76557	7.248784093	-1.48471936	3.88E-05
0	0	0	0.219694972	-4.78350836	3.89E-05
2.086993635	0	0.867348759	2.163343928	-4.57229272	4.23E-05

4.173987271	0	0	2.636577902	-4.24709491	4.29E-05
2.086993635	23.69647987	5.204092551	0.938639161	5.519039825	4.38E-05
168.0029876	143.934174	165.6636129	6.596692615	1.381275844	4.53E-05
6066.890498	2510.949219	4318.529469	12.53547384	-1.29476353	4.59E-05
16.69594908	14.92000584	0.867348759	4.82602745	-2.65496002	4.64E-05
20.86993635	36.86119091	11.27553386	3.390691886	2.91255469	4.77E-05
31.30490453	129.0141682	33.82660158	3.955162434	3.832583478	4.80E-05
452.8776189	195.7153708	237.6535598	8.600907419	-1.53463964	4.83E-05
103.3061849	277.3365792	161.3268691	6.316761645	1.614508288	4.84E-05
107.4801722	35.9835435	39.89804289	6.831750164	-1.75796306	5.11E-05
0	0	0	-1.25141857	-5.14477607	5.14E-05
715.8388169	21.94118506	136.1737551	9.63129695	-2.9601343	5.18E-05
14.60895545	44.76001753	6.07144131	2.843946502	3.502408322	5.19E-05
1375.328806	748.6332343	483.9806073	10.54678038	-1.48883904	5.23E-05
83.47974541	275.5812844	110.1532923	8.06187752	-2.95513568	5.44E-05
0	0	0	-0.03494835	-4.57538213	5.44E-05
20.86993635	25.45177467	12.14288262	2.132369602	4.03440956	5.49E-05
0	0	0	-1.41054245	-5.10198068	5.86E-05
144.0025608	216.7789084	439.7458206	7.304601725	1.433739216	6.19E-05
21.91343317	27.20706948	5.204092551	2.350885288	3.801545333	6.28E-05
183.6554399	173.7741857	118.8267799	5.505328506	2.582783479	6.35E-05
43.82686634	174.6518331	33.82660158	5.212580528	2.349528398	6.55E-05
9.391471359	18.43059545	8.673487586	1.487227818	4.911124271	6.63E-05
0	2.632942208	0	1.352057574	-4.1350665	6.82E-05
12.52196181	23.69647987	10.4081851	2.797547911	3.794508333	7.07E-05
4.173987271	31.59530649	12.14288262	2.071447143	4.614730788	7.11E-05
7.304477724	24.57412727	6.938790069	2.339765557	4.284092775	7.17E-05
4.173987271	16.67530065	37.29599662	5.536758461	-2.44747057	7.28E-05
51.34004343	117.4379989	31.01639161	5.331651328	1.961980327	7.29E-05
0	0.877647403	0	2.895295973	-3.91418672	7.47E-05
4.173987271	0	0	2.756009993	-4.33014892	7.95E-05
76.17526769	34.2282487	45.10213545	6.625480374	-1.74924236	8.17E-05
29.21791089	50.02590194	32.95925283	4.576721168	2.80864918	8.30E-05
20.86993635	8.776474025	1.734697517	4.630856812	-3.0943655	8.46E-05
219.5517304	62.31296558	78.65118543	6.594475068	1.8986083	8.63E-05
0	0	0.867348759	2.038140699	-4.4732671	8.68E-05
44.87036316	50.02590194	43.36743793	4.640667142	2.170802716	8.94E-05

4.173987271	2.632942208	0	2.373237755	-4.19549071	9.16E-05
0	22.81883247	0	4.81163184	-3.83005049	9.27E-05
26.08742044	56.16943376	17.34697517	4.114126999	2.417573183	9.52E-05
26.08742044	122.2475067	19.08167269	4.516873029	3.552743875	9.83E-05
31.30490453	0.877647403	5.204092551	7.401973996	-3.81347729	9.88E-05
1.043496818	0	0	1.320807885	-4.05376245	0.000103531
181.5684463	177.2847753	241.9903036	6.607279068	1.714143343	0.000104537
4.173987271	7.898826623	11.27553386	-8.70511228	10.48525159	0.000104719
594.7931861	533.6096207	372.9599662	9.953000479	-1.08812089	0.000105499
96.00170723	847.8073908	262.8066738	7.149972243	2.347988843	0.000106139
52.17484088	92.15297726	46.83683296	5.102387429	1.615521712	0.000106916
3.130490453	0	0.867348759	1.988231399	-3.00774948	0.000112779
7.304477724	43.88237013	4.336743793	2.41462726	3.902237879	0.00011451
2.086993635	14.92000584	0	3.903766481	-3.53311497	0.000114555
27.13091726	223.8000876	447.5519594	9.166740626	-3.57600557	0.000116359
40.69637589	190.4494863	105.8165485	5.677753164	1.726851307	0.000117145
39.65287907	0.877647403	26.88781152	5.184789456	-3.05757445	0.000117889
129.3936054	247.4965675	124.0308725	6.962641814	1.199302816	0.00011858
37.56588544	31.59530649	8.673487586	3.551429488	2.958408788	0.000119858
27.13091726	0	5.204092551	5.163903774	-3.30100932	0.000125809
0	0	0	-0.35741804	-5.37142033	0.000126066
6.260980906	43.88237013	7.806138827	-8.70511228	14.50333372	0.000126384
1915.860157	2257.309119	2765.107842	10.64472876	1.481119121	0.000126996
0	0	0	0.828953979	-4.34224368	0.000127502
2.086993635	0.877647403	1.734697517	2.426325568	-3.10004001	0.0001289
0	0	0.867348759	2.317918772	-4.43023637	0.000132419
757.5786896	1373.518185	754.59342	9.415882447	1.772773567	0.000138327
3.130490453	41.24942792	5.204092551	1.611579396	4.855012134	0.000138742
38.60938225	79.86591363	100.612456	5.411101327	3.124793948	0.000140895
10.43496818	49.14825454	8.673487586	3.28225945	2.866893577	0.000141123
167.4081945	158.7751916	140.4931519	7.603824406	-1.11230453	0.000146034
9.391471359	27.20706948	9.540836344	2.368274184	3.821918354	0.000146993
725.2302883	1178.680462	1048.624649	9.709200091	1.424894708	0.000154533
10.43496818	0.877647403	2.602046276	4.832221081	-3.00495154	0.000156347
275.4831599	209.7577292	183.8779368	8.104474425	-1.18082834	0.000158507
26.08742044	98.29650908	43.36743793	5.361828768	2.769150294	0.000160309
1484.895972	2238.878524	2323.627324	9.993183788	1.444142188	0.000160888

2.086993635	0	1.734697517	0.921760776	-3.68678117	0.000165461
1336.719423	118.4823993	114.4900361	9.653034412	-3.26288002	0.000166955
4.173987271	5.265884415	0.867348759	-8.70511228	9.227478083	0.000167269
3.130490453	0.877647403	1.734697517	2.485697296	-2.90734063	0.000168566
44.87036316	100.9294513	59.84706434	5.741982483	2.938182225	0.000169699
178.4379558	408.1060422	449.2866569	7.631044005	1.385361867	0.000170264
620.8806065	1418.278202	2384.341737	9.271425073	1.661267015	0.000172609
109.5671659	163.2424169	156.9901253	6.730132704	0.911920143	0.000172967
1.043496818	0	1.734697517	1.846844605	-3.97248572	0.000173519
11.47846499	34.2282487	3.469395034	1.885817852	4.27370051	0.000176586
141.9155672	202.73655	151.7860327	6.134834555	1.546547439	0.000178106
186.7859304	214.1459662	197.755517	8.217472873	-0.90577507	0.000178282
0	0	0	-1.45323363	-4.34305557	0.000188903
98.08870086	348.4260188	98.01040972	6.15809044	2.723390255	0.000189494
78.26226132	151.8330006	147.449289	6.009533996	1.522134549	0.000198398
64.80115238	199.5770193	44.88529826	6.041961405	1.87503353	0.000201102
0	0	0	1.915775063	-4.68369426	0.000203773
1.043496818	0	0.867348759	0.422855469	-3.68620743	0.000204134
0.34435395	0	0	-0.85624316	-4.02131815	0.000208161
57.39232497	40.37178052	54.64297179	6.10931829	-1.29481814	0.000217448
114.7846499	239.5977409	142.2451964	6.99256317	1.28624832	0.000219694
15.65245226	14.04235844	74.59199324	7.205693586	-2.32711403	0.000224625
177.394459	426.5366376	490.9193973	8.215326445	1.259652465	0.000226988
0	0	0	-1.70682714	-5.29688181	0.000229837
629.2285811	360.7130824	608.0114798	9.937609164	-1.17049662	0.000230949
3002.140344	2088.800818	1391.227409	9.697852797	2.884332243	0.000231196
137.7415799	384.4095623	177.8064955	7.574667509	2.442842151	0.000232367
300.5270835	216.7789084	178.6738443	8.865891591	-1.1536194	0.000233355
0	0	0	-8.70511228	-9.52322727	0.000236774
1013.23541	851.3179804	854.3385272	10.04682511	-1.07656653	0.000242804
12.52196181	22.81883247	6.938790069	2.572326605	3.788326695	0.000244947
40.69637589	107.9506305	78.06138827	5.013745102	2.733247631	0.000247217
1752.031157	1426.177029	1297.553743	11.60673836	-1.12067163	0.00024726
10.43496818	59.68002337	6.938790069	2.786985698	3.698469585	0.000251521
18.78294272	55.29178636	39.03069414	3.950194225	3.069594516	0.000256433
97.04520404	77.23297142	39.89804289	7.215764006	-1.21874614	0.000260265
11.47846499	49.14825454	12.14288262	2.559875748	3.774096015	0.000260826

652.185511	915.3862408	510.00107	9.882793286	-1.4575158	0.00026747
1.043496818	14.04235844	13.01023138	1.370565471	4.372984284	0.00027147
12.52196181	13.16471104	11.27553386	2.298291488	3.172010774	0.000274755
12.52196181	31.59530649	34.69395034	4.021148459	2.063243448	0.000280388
5.217484088	10.53176883	4.336743793	1.892673988	4.369400491	0.000283686
0	0	0	-1.70837581	-5.29378448	0.000300857
23243.89161	8217.41263	7110.525123	13.88096262	-1.90991011	0.000302532
1212.543302	3062.111787	8458.385094	10.8553351	2.245179	0.000307303
285.7720385	699.3182268	332.5675345	7.504103905	1.912306919	0.000313054
13.56545863	151.8330006	12.14288262	3.94627905	3.710615444	0.000316275
19.82643954	16.67530065	59.84706434	4.355958764	2.484276455	0.000318237
0	0	0	0.459664368	-3.66067794	0.000323452
13.56545863	43.00472272	13.87758014	3.726094281	2.298257316	0.000323817
893.6506746	640.0594742	977.8750109	10.19212733	-1.12295912	0.000324489
0	0	0.867348759	0.918837205	-3.55204179	0.000327018
31.30490453	43.00472272	45.10213545	3.629171825	2.960252507	0.00032925
54.26183452	30.71765909	45.9694842	6.148874871	-1.26626579	0.000332194
134.2980404	239.3695526	263.6740226	6.997169178	1.719203299	0.000332298
184.6989367	203.6141974	527.3480452	7.055690544	1.540278782	0.000334481
148.1765481	207.124787	104.081851	8.200821148	-1.66940747	0.000342099
52.17484088	174.6518331	71.1225982	5.293152353	1.667187716	0.000344207
0	0	0	-1.42911601	-4.78938119	0.000344931
135.111968	55.19524514	25.0143382	7.172515999	-1.72992835	0.000355489
9.391471359	16.67530065	6.938790069	4.116402498	-1.9254723	0.000362887
3.130490453	0	0	2.816235475	-3.53737008	0.000366503
573.8189	928.5509519	864.6513039	8.972468848	1.096486367	0.000368844
2499.174878	7335.37699	3016.638982	11.2702085	1.322042937	0.000371534
9.391471359	58.80237597	17.34697517	3.660325583	2.849438273	0.000377502
16.69594908	10.53176883	20.81637021	5.058352713	-1.5768065	0.000378077
4421.296016	2322.255027	1288.880255	11.64216499	-1.23995575	0.000378797
21.91343317	48.27060714	16.47962641	4.234624547	2.160179789	0.000379122
7.304477724	1.755294805	0.867348759	4.412431977	-3.1511419	0.000380976
378.7893448	276.4589318	299.2353217	8.495610471	-1.1854961	0.00038418
13.56545863	0	1.734697517	3.912709327	-2.71034632	0.000384807
8.347974541	61.43531818	3.469395034	1.953144784	4.490342084	0.000394206
0	0	0	-0.46235157	-4.11402182	0.000395597
138.7850767	67.57884999	15.61227765	7.796772559	-2.23738965	0.000400912

299.4835867	106.1953357	331.3272258	8.418215715	-1.55426219	0.000403171
452.8776189	580.1249331	529.9500915	8.626492852	0.911784642	0.000406081
14.60895545	32.47295389	13.01023138	3.578350239	3.643611546	0.000410052
16.69594908	93.90827207	255.000535	4.794105001	3.328354191	0.000413951
9.391471359	123.7482838	6.938790069	3.587288325	4.830211936	0.00041571
5.217484088	18.43059545	2.602046276	2.389077569	3.742335696	0.000418173
147.1330513	107.9506305	270.6128127	7.579648932	-1.11797417	0.000423408
60.52281542	7.02117922	62.44911062	6.101453075	-2.30406753	0.000426696
197.2208985	377.3883831	274.0822077	7.514139451	1.291118393	0.000428347
90.78422314	269.4377526	188.2146806	6.496443525	1.974683386	0.00043278
288.0051217	951.3697843	541.2256253	8.591895221	1.221838777	0.000433734
5.217484088	5.265884415	4.336743793	3.610270789	-2.23065915	0.000434671
62.60980906	100.9294513	16.47962641	4.960671649	2.013840152	0.000434833
310.9620517	866.2379863	399.8477777	8.402232358	1.220214134	0.000437305
7.304477724	1.755294805	6.07144131	3.928694079	-1.87163782	0.000440758
165.915994	1057.56512	289.6944854	6.949865659	4.127919194	0.000442635
7.304477724	23.69647987	14.7449289	2.612135802	2.363778786	0.000448361
0	0	0	0.376269487	-3.75992181	0.000452346
928.7121677	497.6260772	729.440306	9.724315111	-1.59990889	0.00045296
358.9629053	457.2542967	465.7662833	8.722150222	1.100607763	0.000454968
6895.426971	3890.610935	1257.6557	13.11243291	-1.5144246	0.00045567
22.95692999	23.69647987	21.68371896	2.898284498	3.188917541	0.000455848
35.4788918	114.9718097	87.60222462	5.483295848	1.405632799	0.00045622
2122.472527	2066.859633	3336.690674	10.49739686	1.836015927	0.000459028
607.3151479	618.7414188	1535.207303	9.295942904	2.031051948	0.000459751
3715.892168	2334.542091	3741.742544	10.58656261	1.459333716	0.000460541
16.69594908	29.84001169	4.336743793	3.585101166	2.879101175	0.000461522
1.043496818	0	0.867348759	0.069865915	-3.84831501	0.000468127
445.5731411	466.9084181	263.6740226	9.618371217	-1.59856742	0.000469868
291.1356121	8.776474025	17.34697517	7.162176949	-2.76301216	0.000469994
8.347974541	7.898826623	2.602046276	3.87634769	-1.9003442	0.000477684
1706.117297	1167.271045	1085.053297	10.495748	-1.42369043	0.000478798
20.86993635	47.39295974	91.07161965	4.781831063	3.784702438	0.000479781
1.043496818	49.14825454	0	5.605631117	-4.3423855	0.00048336
0	0	0	-8.70511228	-8.26154238	0.000483716
405.9202621	299.2777643	426.7355892	9.089331741	-1.00121927	0.000487192
906.7987345	102.6847461	699.9504482	6.454557678	5.606854693	0.000496369

9130.597155	10685.35713	5315.113192	13.63115495	-1.32071202	0.000503035
548.8793261	253.6400993	370.3579199	8.354428361	1.122610606	0.00050317
45.91385998	60.55767077	15.61227765	4.572887218	2.068776921	0.000504193
2.086993635	0.877647403	1.734697517	3.036929803	-2.76681417	0.000508978
86.61023587	59.68002337	91.93896841	6.026859024	1.218083648	0.000516717
5.217484088	4.388237013	0.867348759	4.520083431	-3.04232355	0.000516844
5.217484088	8.776474025	12.14288262	1.93495488	2.422980869	0.000521496
113.7411531	78.98826623	96.2757122	5.590015761	1.708227192	0.000521524
73.04477724	102.6847461	181.2758905	6.470269596	1.20777629	0.000527918
641.7505429	216.7789084	210.7657483	7.388404585	1.791174663	0.000534076
190.9599176	356.3248454	318.3169944	7.489942835	1.160725786	0.000538649
567.6622688	416.0048688	409.388614	9.909778115	-1.49746036	0.000546645
202.4175127	105.3879001	151.3610319	8.238999775	-1.39741731	0.000549738
404.8767653	748.6332343	1211.686216	8.513967514	1.781993865	0.000552518
1884.555253	2095.821997	1819.697695	10.32290318	1.825839078	0.000554354
0	3.51058961	0.867348759	-8.70511228	9.545969233	0.000563434
8.347974541	28.08471688	8.673487586	2.756963671	2.588948788	0.000564266
60.52281542	31.59530649	122.296175	5.402670656	2.812423	0.000566228
18.78294272	0	5.204092551	3.264211382	-3.52689636	0.000579473
10.43496818	19.30824286	9.540836344	2.274498368	3.517103907	0.000580847
19.82643954	5.265884415	6.07144131	4.280538688	-2.14411337	0.000582292
218.0908349	251.0071571	197.755517	8.363870406	-0.76140525	0.000584446
0	0	0	-1.5374195	-4.5727742	0.00058505
81.39275178	108.8282779	241.9903036	6.425639873	1.528840644	0.000589143
755.491696	1721.066556	1071.175717	8.830232984	1.567196475	0.000595157
33.39189817	71.0894396	27.75516027	4.584100532	2.165313332	0.000596601
429.3780705	2410.967626	419.9269015	8.084237757	3.134512845	0.000596967
9.391471359	14.04235844	5.204092551	1.894487097	2.911642957	0.000598875
223.308319	69.3341448	137.0411039	7.664699136	-1.02217491	0.000602758
17.7394459	26.32942208	6.938790069	2.333212021	2.785061837	0.000607165
21.91343317	13.16471104	13.01023138	5.102402746	-1.4565873	0.000614339
55.21141662	146.7601986	55.13736058	5.028738953	1.896970439	0.000615201
183.6554399	263.2942208	300.1026705	7.604419086	1.026376782	0.000616617
0	0	0	-1.49951628	-4.49696776	0.000617813
470.6170648	794.2708993	973.1653071	8.737529846	0.995540935	0.00062024
6.260980906	27.20706948	13.01023138	2.70196456	2.215561636	0.000623551
1.043496818	0	0	-1.51467156	-4.46251433	0.000625834

0	4.388237013	0.867348759	2.724186008	-3.83694361	0.000625951
20.86993635	0	7.806138827	5.595803932	-2.7596792	0.000630467
995.4959641	817.9673791	1588.115577	9.554265723	1.069566067	0.000643839
530.0755134	142.8897736	436.2590786	9.332955706	-1.43432405	0.000646175
107.4801722	74.60002921	62.44911062	8.570839824	-3.00370438	0.000652515
3.130490453	2.632942208	6.07144131	4.087777631	-2.7967812	0.000667472
6463.440159	1683.2224	889.9171733	12.76294862	-1.99545432	0.000674886
231.6562935	229.9436195	208.1637021	7.055976806	1.314958989	0.000676532
7.304477724	137.7906422	99.74510724	5.247872994	5.296830001	0.000678422
123.1326245	112.3388675	130.9696625	7.312576947	-0.86384784	0.000688244
0	0.877647403	1.734697517	3.896911679	-3.48448606	0.000689511
9.391471359	20.18589026	24.28576524	3.078785345	2.484452299	0.000690923
5.217484088	0	1.734697517	3.989123418	-2.95681429	0.000692592
35.4788918	339.6495448	124.8982212	5.556268815	2.313252132	0.000694762
9.391471359	133.4024052	21.68371896	4.678638602	3.4392248	0.000701398
10.43496818	2.632942208	4.336743793	3.513964714	-1.77087015	0.000704942
2.086993635	2.632942208	9.540836344	1.114884205	4.01521742	0.000706727
19.82643954	1.755294805	19.08167269	4.915753445	-1.83982477	0.000726418
21.91343317	43.88237013	33.82660158	-8.70511228	11.26863179	0.000729278
9.641910595	67.57884999	13.01023138	5.247620116	-2.41196151	0.000731252
3.130490453	6.143531818	0	5.244050835	-3.35967682	0.000736576
51.13134407	100.9294513	70.25524944	5.432527194	1.447093004	0.000739433
12.52196181	24.57412727	9.540836344	3.042575196	2.731556107	0.00074524
267.1351853	915.3862408	671.3279391	8.358065517	1.224197752	0.000753159
136.6980831	76.35532402	75.459342	7.782849332	-1.43159658	0.000758506
1035.148843	382.6542675	1026.073581	11.36365366	-1.60080049	0.000759169
57.39232497	64.94590779	90.20427089	5.501723924	1.341312115	0.000759663
1896.033718	2932.219972	656.5830102	11.83542913	-1.23232992	0.000765221
17.7394459	64.06826038	9.540836344	4.078092912	2.105276383	0.000765577
41.73987271	293.1342324	125.76557	5.877299308	2.257955328	0.000769216
92.87121677	897.8332928	117.9594312	7.311452222	4.13666344	0.00077683
25.04392362	53.53649155	31.22455531	4.582087762	1.534868499	0.000782634
55.30533134	39.49413311	27.75516027	6.150392918	-1.88789189	0.000788018
44.87036316	132.5247578	117.0920824	5.771202866	1.537504685	0.000789303
26.08742044	58.80237597	52.04092551	4.475295511	1.496189983	0.000791013
0	1.755294805	1.734697517	1.429683123	-3.13778068	0.000792954
194.0904081	131.6471104	211.6330971	8.192835905	-0.9509416	0.000795178

926.6251741	558.183748	537.7562303	9.915111165	-0.95656476	0.000800142
12.52196181	15.79765325	2.602046276	2.387469189	2.535515765	0.000811638
274.0013944	445.8448805	405.1646256	7.882621004	1.290650272	0.000812667
16.69594908	0	15.61227765	3.861634318	-2.91016498	0.000813107
38.60938225	86.88709285	88.46957337	5.447676926	1.345360186	0.000818015
49.04435043	142.1788792	72.85729572	6.603057463	2.281069457	0.000819235
22.95692999	71.96708701	46.83683296	4.679618964	1.827341702	0.000819638
0	1.474447636	0	3.731152453	-3.17236811	0.000820047
0	0	0	1.386393497	-4.4204002	0.000821035
1279.327098	29.84001169	666.9911953	9.898887543	-2.40304057	0.000823938
3.130490453	7.02117922	7.806138827	1.288297549	4.218356601	0.000825083
83.47974541	100.9294513	111.8879899	6.401758121	1.555858345	0.000834995
588.5322052	583.6355227	520.4092551	9.170130309	-0.76636806	0.000843406
135.6545863	28.96236428	212.5004458	5.164278799	3.414945259	0.000859819
456.0081093	918.0016301	592.3992021	9.050871771	1.06713762	0.000860978
730.4477724	142.1788792	184.7452856	9.109266922	-1.50484557	0.000864639
6.260980906	14.04235844	6.07144131	1.638948106	3.003407935	0.000867119
0	0	0	-1.52425631	-4.20101022	0.000878197
27.13091726	14.04235844	8.673487586	3.466020639	2.644794604	0.000879074
0	0	0	-1.04814771	-3.89075537	0.000895823
0	0	0	-8.70511228	-10.0421043	0.000896817
300.5270835	1744.763036	470.1030271	9.009233335	3.546485171	0.000899338
32.34840135	2.632942208	16.47962641	5.273060784	-2.02619897	0.000907081
62.60980906	25.45177467	72.85729572	6.968068718	-1.46717713	0.000911978
0	0	0	-1.93263455	-4.845267	0.000916144
0	0	0	-1.94126517	-4.82800575	0.000921234
137.7415799	68.4564974	170.8677054	4.003881386	3.839156615	0.000926911
7.304477724	25.45177467	4.336743793	6.046930463	-2.65361602	0.000932038
1263.674646	772.3297142	771.0730464	10.35601815	-1.2379226	0.000932633
9.391471359	18.43059545	3.469395034	2.921645511	2.387885959	0.000933929
85.56673905	93.03062467	21.68371896	5.101318221	2.115052421	0.000935609
456.0081093	2982.245874	847.3997371	9.612503197	1.42923278	0.000941481
0	0	0	-1.98547661	-4.73958288	0.000943073
16723.08	8320.975023	9125.376289	11.8749587	1.56748991	0.00094487
259.8307076	803.0473733	725.9709109	8.22148542	2.081310191	0.000945515
5.217484088	17.55294805	49.43887924	3.009384493	2.608119185	0.000947841
338.0929689	109.7059253	200.3575632	8.757324612	-1.72556158	0.000949902

12.52196181	7.898826623	6.07144131	0.856980492	3.557838394	0.000951854
29.21791089	3.51058961	10.4081851	5.176951435	-2.3809395	0.000952369
1211.499805	971.5556746	713.8280283	10.29269346	-0.74239929	0.000955301
150.2635417	473.9295974	527.3480452	6.82731644	2.249143175	0.000955502
86.61023587	126.381226	52.04092551	5.72804247	1.24403719	0.000965776
0	0	0	-8.70511228	-8.70664925	0.000966091
265.0481917	317.7083597	458.8274933	7.509868778	1.364211057	0.000966823
175.3074654	1344.555821	466.6336321	8.311408607	1.529021409	0.000968197
0	0	0	-0.99557224	-4.29887511	0.000968848
528.0093897	872.3815181	997.4510724	9.155935814	1.040847378	0.000969783
12.52196181	21.06353766	11.27553386	3.688968436	2.1196342	0.000970349
213.9168476	351.9366084	396.3783827	7.536391149	1.301945313	0.000970411
230.6127967	748.6332343	428.4702867	7.893676417	1.370259143	0.000972618
15.65245226	50.02590194	62.44911062	4.345810342	1.991573161	0.000973291
139.8285736	177.2847753	152.6533815	7.429724842	-0.91888961	0.000973529
29.21791089	29.84001169	8.673487586	3.776136313	2.38937801	0.000978495
41.73987271	118.4823993	30.35720655	5.000099559	1.774725081	0.000981499
76.17526769	623.1296558	71.98994696	6.491547901	2.583879583	0.000988444
107.4801722	14.04235844	46.83683296	7.218412358	-1.51726868	0.001000465
7.304477724	8.776474025	3.469395034	1.844526608	2.691063911	0.001002648
153.3940322	319.4636545	149.1839865	6.790848923	2.344610581	0.00100698
1096.715155	2237.123229	1612.401342	8.790640242	3.087581568	0.001010552
87.65373268	16.67530065	51.17357676	6.343658923	-1.40559255	0.001012167
153.3940322	182.5506597	370.3579199	7.034310077	1.595400063	0.001018154
1.043496818	2.632942208	2.602046276	-8.70511228	9.325158973	0.00101964
996.5394609	665.2567311	366.8885249	10.47901666	-1.30874154	0.001022995
5223.745069	1095.303958	1727.758727	11.88169594	-1.10316878	0.001024887
1434.808124	1209.398121	334.7966208	10.88194754	-1.29461966	0.001035402
0	0	0.867348759	-0.97136893	-3.4490565	0.001039982
1373.241812	680.1767369	440.6131694	11.47292886	-2.0710482	0.001049363
255.6567203	122.8706364	169.1330079	7.878131947	-0.95110384	0.001062272
310.9620517	313.3201227	624.4911062	8.141644355	0.908796949	0.001069372
41.73987271	68.4564974	37.29599662	6.492449383	-1.09093727	0.001074647
0	0.877647403	0.867348759	0.730725244	-3.68914111	0.00107583
73.04477724	209.4066702	91.14968104	6.283603002	1.152491576	0.001076065
2132.907495	7442.449973	2630.668785	10.89212364	1.215036434	0.00107952
21.91343317	514.3013779	372.0926174	5.825411018	3.751973541	0.001091673

42.78336952	48.27060714	41.63274041	6.055074729	-1.23452087	0.001093241
35.4788918	931.1838941	74.59199324	6.652005579	4.390367652	0.00109581
160.6985099	45.63766493	428.4702867	5.168536348	3.161994374	0.0011023
374.6153575	315.9530649	259.3372788	8.858936037	-0.83837326	0.00111022
103.3061849	120.2376941	444.0825644	6.789747743	1.724023922	0.001114806
1342.980404	715.282633	532.5521378	10.31422955	-0.8941096	0.001118177
35.4788918	5.265884415	6.07144131	4.913091249	-1.71652575	0.001119136
0	0	0	-1.65058562	-4.34644198	0.001120308
46.95735679	51.78119675	56.37766931	4.977124154	2.549068957	0.001123215
0	0	0	-1.56139683	-4.30246936	0.001126298
0	0	0	0.280182116	-3.9674885	0.001129215
99.13219768	179.0400701	128.3676163	6.297192361	4.447958037	0.001130773
3401.799626	17309.83972	6627.411864	12.35884705	1.421257799	0.001134036
21.91343317	43.00472272	39.89804289	3.485041588	4.284323629	0.00113585
666.7944665	1973.829008	2175.310686	9.828553721	1.344288866	0.00113706
36.52238862	154.4659428	116.2247336	5.378012907	1.615995787	0.001138838
101.2191913	60.55767077	103.2145023	5.798585432	1.861953836	0.001139911
7906.471038	2969.835939	2571.91458	12.52428037	-1.44219995	0.001152556
3.130490453	182.5506597	30.35720655	3.263470163	4.564061127	0.001152916
1.043496818	0	0.867348759	-0.0858453	-3.103819	0.001156065
666.7944665	638.927309	770.2056976	9.021930758	1.312658783	0.00115819
402.7897716	652.0920201	883.828385	8.641541231	1.050519943	0.001159565
6.260980906	21.94118506	5.204092551	2.350536629	2.80352632	0.001166125
77.21876451	239.5977409	256.7352325	6.506658254	1.223291704	0.0011717
38.63025219	15.80642972	34.70262383	4.054613401	1.881310861	0.001187208
1108.673629	617.8286655	654.1197398	9.987748894	-0.79525997	0.001187287
3039.70623	5465.110375	3399.139785	11.73799298	0.995931767	0.00118756
108.523669	101.8070987	88.46957337	6.508292816	1.025801247	0.001195035
28.17441408	80.74356103	31.22455531	4.67888735	1.681524826	0.001200515
16.69594908	87.76474025	116.2247336	4.579442559	1.926786847	0.001216774
0	0	0	1.898710063	-4.20195581	0.001233462
50.08784725	19.30824286	16.47962641	5.191547614	-1.28702606	0.001246415
69.91428678	98.29650908	60.7144131	5.618383169	1.200562181	0.001248583
434.0946761	741.6120551	528.215394	8.665428026	0.98422629	0.001254966
132.5240958	186.9388967	324.3884357	7.225966474	1.293317795	0.001268026
32.34840135	11.40941623	49.43887924	4.034229665	2.657388491	0.00127022
3.130490453	0	0.867348759	2.753004542	-2.88953381	0.001279075

4.173987271	32.47295389	26.88781152	4.083206761	2.350446026	0.001294168
8342.757057	13666.72535	7906.751283	12.87190127	1.112641166	0.001303235
99.33046207	107.6610069	155.4896119	6.323334143	1.481265494	0.001309683
323.4840135	365.1013194	173.4697517	7.582670295	1.193788308	0.001325817
4.173987271	17.55294805	25.89903393	2.062525135	2.562668882	0.00133103
112.6976563	220.289498	156.9901253	6.619230212	1.509065998	0.001352574
30.26140771	0.877647403	32.95925283	4.877675938	-2.82310132	0.001360823
27.97614968	25.11826866	19.60208194	4.714505378	2.402954935	0.001361074
916.1902059	1878.165441	1526.533815	9.806383808	1.657450782	0.001363431
4.173987271	0	1.734697517	2.855352896	-2.16603875	0.001366663
14.60895545	54.41413896	19.08167269	3.941081417	2.069858606	0.001368429
471.6605616	1056.687473	1091.992087	8.91299477	1.397691912	0.001369015
139.8285736	224.677735	226.378026	6.859783887	1.202493065	0.001369952
360.0064021	946.9815473	403.3171727	8.379876405	0.959771439	0.001372794
321.3970198	691.5861532	677.3993804	8.428035929	1.41271688	0.001374878
13.56545863	40.37178052	22.55106772	3.783317726	2.24477875	0.001381256
2442.82605	7079.103949	6571.034195	11.70248441	1.130737321	0.001393225
9.391471359	30.71765909	2.602046276	2.419948841	2.980705087	0.001418008
10.43496818	15.79765325	11.27553386	3.634949854	1.681846119	0.001419581
3.130490453	0	0	0.339246433	-3.52815056	0.001425674
0	0	0	-0.08755412	-3.44031511	0.001434802
893.2332759	39.49413311	554.2358567	6.365652911	3.657284038	0.001456573
1251.152684	2544.29982	1006.12456	10.01623569	1.232146063	0.001464033
810.7970273	673.1555577	452.756052	9.3544204	-0.90347968	0.001468269
548.8793261	1584.153562	1589.850274	9.51470708	1.216341146	0.001472864
138.7850767	248.3742149	280.153649	6.781181164	1.356771983	0.001479728
10.43496818	14.92000584	6.938790069	3.062694806	2.229298466	0.001482682
38.60938225	915.3862408	49.43887924	5.954084335	3.02561031	0.001491462
85.56673905	30.71765909	62.44911062	5.707214022	1.26563543	0.001495257
14.60895545	9.654121428	12.14288262	4.950248299	-1.35352762	0.001500042
79.30575814	196.5930182	110.1532923	5.69952664	1.973370423	0.001504892
203.4818794	178.1624227	118.8267799	7.235105721	1.596546822	0.001522659
4.173987271	9.654121428	4.336743793	1.894988696	2.312396222	0.00153084
16735.60196	7696.96772	5506.797268	13.51111002	-1.25848353	0.001536205
1180.194901	1426.177029	486.5826536	11.24282222	-1.65857272	0.001554103
514.4439311	154.4659428	190.8167269	8.318821098	-1.19355808	0.001561431
142.959064	146.5671162	138.7758014	7.860943953	-1.34268202	0.001567169

45.91385998	287.868348	136.1737551	6.650886812	1.251806899	0.001593761
2.086993635	7.02117922	4.336743793	0.998552935	3.782554881	0.001598078
0	0	0	-1.06971529	-3.61701176	0.001599003
4.173987271	5803.004625	8580.681268	6.603168351	10.02741346	0.00161414
314.0508022	503.6730678	473.5724222	8.201972415	0.719725142	0.001627078
25.63871681	7.10894396	13.72145736	6.369730888	-3.08219772	0.00165186
126.2631149	107.0729831	56.37766931	7.268756732	-0.78166343	0.001663988
6682.55362	2659.27163	2128.473854	12.22066688	-1.34162324	0.001667563
1.043496818	7.898826623	6.07144131	2.171530547	2.506308897	0.001668205
298.4400899	362.4683772	289.6944854	7.732402808	0.771691256	0.001675893
56.34882815	85.13179804	42.29192547	5.522043545	1.287692702	0.001678304
15.65245226	42.12707532	19.94902145	4.436079748	1.504690098	0.001681518
0	0	0.867348759	-0.22714703	-3.31379037	0.001709549
841.058435	530.0990311	654.8483127	9.552270601	-0.95439738	0.001715189
36.52238862	343.1601344	32.95925283	5.887134214	1.879129764	0.001721036
195.1339049	480.0731292	340.0007134	7.780472719	1.158642567	0.001722875
5.217484088	267.6824578	98.01040972	4.465692993	3.979776479	0.00175352
51.13134407	103.5623935	97.14306096	6.477336056	2.028740843	0.001757296
5.217484088	14.92000584	6.07144131	2.268217214	2.649126294	0.001762682
516.5309247	469.5413603	435.4090768	8.470836263	0.971550382	0.001767722
591.6626956	1640.322995	686.072868	8.833099962	1.252667179	0.001770167
3012.575313	1042.645114	1526.533815	11.23097592	-1.25669589	0.001771686
0	0	0	-0.75853796	-4.1305373	0.001784695
0	0	0	1.732134329	-3.22488532	0.00183565
208.6993635	266.8048104	56.37766931	8.616111539	-1.08717728	0.001836995
318.2665294	98.29650908	131.8370113	8.020108401	-1.71285664	0.001838514
0	0	0	-8.70511228	-8.78592542	0.001840753
8366.757484	2500.41745	5951.747181	13.25509546	-1.71052787	0.001843934
103.3061849	70.2117922	87.60222462	6.675025699	-0.85478213	0.001847834
56.34882815	121.1153415	6.938790069	8.651438997	-2.02968185	0.001862466
130.4371022	305.4212961	248.9290937	7.308221087	0.865859754	0.001866873
0	0	0	-8.70511228	-9.85267172	0.001872691
5.217484088	0.877647403	2.602046276	3.221379599	-2.58068054	0.001875255
401.7462748	200.1036078	135.3064063	8.628978641	-1.02864535	0.001883405
562.4447847	896.9556454	1012.196001	8.865391315	1.04886168	0.001895955
2843.528828	2006.301962	405.0518703	10.88682934	-1.61079231	0.001896281
0	0	0	-1.33635127	-4.03783356	0.00189836

6623.074302	1580.642972	1207.349472	12.05569724	-1.34740925	0.00190932
20.86993635	22.81883247	29.48985779	5.099414053	8.17360971	0.001910017
1212.543302	1507.798238	1976.687821	10.09540512	1.345837341	0.001919728
27.13091726	71.96708701	89.33692213	4.557789892	1.723949685	0.001924612
126.2631149	542.3860947	1057.298137	7.683554335	1.987361616	0.001949906
94.95821041	258.0283363	371.2252687	7.074470187	1.141104298	0.001967493
1104.019633	1044.400409	1051.226695	9.156628905	0.949755088	0.001968141
17.7394459	7.898826623	14.7449289	2.660299447	2.101441932	0.001978532
10.43496818	26.32942208	152.6533815	4.097407894	3.174177816	0.001981635
131.480599	105.3176883	80.66343455	7.130553661	-0.81645653	0.001989674
17.7394459	57.04708116	7.806138827	3.842102878	2.527910803	0.002001684
1147.846499	65.82355519	505.6643262	9.62172843	-2.10184695	0.002011461
75.13177087	123.4586601	44.28682761	6.951736735	-1.08652661	0.002020253
517.5744216	206.2471396	464.8989346	8.068765761	1.447844354	0.002021526
103.3061849	143.0565266	150.918684	6.383280882	0.937013658	0.002041195
221.2213253	93.90827207	510.00107	5.182549792	3.181952548	0.002056648
6.260980906	0	7.806138827	1.00520733	3.642268003	0.002057691
64.6968027	364.223672	248.0617449	7.386948962	1.275117098	0.002058635
9.391471359	30.71765909	31.22455531	4.304454554	1.568233699	0.002061479
0	0	0	0.82547768	-3.50131342	0.002062144
1.043496818	0	0	-0.11673957	-3.52050923	0.002070601
175.3074654	149.2000584	228.9800723	7.175426125	1.004415973	0.002076886
137.7415799	132.5247578	280.153649	6.744525431	1.312382838	0.002088181
166.9594908	371.2448513	383.3681513	7.407053322	1.225849106	0.002089175
634.4460651	1274.344028	1033.87972	9.34365172	0.832644004	0.002095256
0	827.6215006	277.5516027	6.413353675	5.825302968	0.002103079
99.13219768	173.7741857	182.1432393	6.761835307	1.076985391	0.002125018
77.21876451	343.1601344	204.694307	7.063129535	1.138284712	0.002154054
178.4379558	485.3390136	223.7759797	7.954595149	1.244355919	0.00216227
28.01788955	79.98000779	76.35271122	5.617171203	1.216419337	0.00216356
584.3582179	263.2942208	418.5998579	9.52729706	-1.12854743	0.002165694
0	0.877647403	0	-0.12632039	-3.56305516	0.002166012
488.3565107	386.1648571	333.0619233	8.751953575	0.997129848	0.002166409
2.086993635	5.265884415	3.469395034	1.253775441	6.261528399	0.0021741
142.959064	80.74356103	252.3984887	6.30203582	1.362617586	0.002178732
284.8746312	292.256585	255.000535	7.787397023	0.848075651	0.002190209
2961.443969	132.5247578	706.0218895	11.18057162	-2.68464947	0.002190922

70.9577836	141.3012318	121.4288262	6.17892375	1.960309815	0.00219216
69.91428678	174.6518331	238.5209086	6.500455877	1.487213552	0.002200877
555.140307	168.5083013	196.0208194	8.967326411	-1.50289128	0.002200892
0	0.438823701	0	-0.35001894	-3.76347743	0.002210832
60.52281542	151.8330006	287.9597878	6.579745088	1.400021937	0.002211735
0	0	0	-8.70511228	-8.28395997	0.002226414
2367.694279	1090.038074	809.2363917	10.79332863	-1.18377778	0.002243881
2.086993635	0.877647403	0.867348759	-8.70511228	9.1995135	0.002252448
784.7096069	258.0283363	300.1026705	9.081016428	-0.93880104	0.002261331
172.1769749	107.9506305	336.5313183	4.362634381	6.239460167	0.002264992
424.7032048	88.64238765	145.7145914	8.444538708	-1.15516754	0.002274528
1.043496818	0	0	0.462098663	-3.23768802	0.002276663
41.73987271	63.19061298	69.38790069	4.61168121	1.495968113	0.002281396
109.5671659	249.7784508	190.1575418	6.95254625	1.138285139	0.002283261
2117.255043	816.2120843	866.4814098	10.64553788	-1.29283248	0.002284416
15.65245226	22.81883247	5.204092551	3.468909267	2.368836639	0.002292587
237.9172744	367.7342617	232.4494673	4.987641596	4.903372787	0.002293757
328.7014976	405.4731	834.3895057	8.133686616	1.231228413	0.002295436
0	0.877647403	0.867348759	1.16977502	-2.78949969	0.002303226
1182.281894	752.1438239	780.6138827	10.14561272	-1.33700489	0.002305553
0	0	0	0.058520181	-3.80834877	0.002307555
153.9470855	164.3043702	186.3238603	6.951384189	0.759838134	0.002308199
134.6110895	2.632942208	39.03069414	6.573136996	-2.78984094	0.002319403
50.08784725	73.72238181	71.1225982	5.305658987	1.066071384	0.002323097
64.6968027	8.776474025	18.21432393	5.412950324	-1.66523588	0.002333897
36.52238862	114.0941623	81.53078331	4.124544084	2.882779453	0.002365731
2698.48277	4281.164029	1696.534172	11.19554263	0.817589353	0.002366489
8.347974541	3.51058961	25.153114	3.429074417	2.862477645	0.002388152
409.0507525	219.4118506	196.8881682	8.421520138	-0.98875383	0.002391651
14.60895545	12.28706364	13.01023138	4.797424808	-1.3396981	0.002396011
13.56545863	3.51058961	24.28576524	1.047568826	3.672663358	0.00239822
515.4874279	707.3838064	428.4702867	8.538165662	0.947655991	0.002399483
22.95692999	18.43059545	25.153114	3.961288072	1.448938466	0.002406961
34.43539498	61.43531818	115.3573849	5.286547471	2.346841471	0.00242227
28.17441408	54.41413896	17.34697517	4.323009863	2.6213285	0.002424127
183.7597896	341.5628161	166.6784109	7.376698183	0.889762624	0.002433011
469.9283569	467.961595	293.0424516	8.36142004	1.573266932	0.002439361

413.2247398	1943.988997	647.9095226	9.40424143	0.997202091	0.002446164
0	7.898826623	0	3.810155804	-3.03940682	0.002452491
1.043496818	1.755294805	9.540836344	0.161685511	3.965132525	0.002461044
13.56545863	0.877647403	0	2.106812496	-3.40101161	0.002462138
4.173987271	6.143531818	1.734697517	1.576215153	2.202908991	0.002470738
7.304477724	13.16471104	9.540836344	2.799421998	2.417018102	0.00247606
272.1126651	599.749129	451.6371721	8.048487193	1.187691736	0.002477963
192.0034145	248.3742149	136.1737551	5.921423724	2.079493212	0.002487105
3.130490453	12.28706364	6.07144131	1.605230071	2.907784161	0.002489844
0	0	0	-8.70511228	-7.8907764	0.002490826
354.788918	43.00472272	398.1130802	8.502564149	-1.60520499	0.002493556
421.5727143	621.374361	979.2367484	8.813366846	1.476768471	0.002501748
227.4823063	140.4235844	88.46957337	7.452831115	-1.30022117	0.00251082
332.8754848	580.1249331	1161.379988	8.517374232	1.110750891	0.002522636
625.5346023	1284.568621	861.3987461	9.43996025	0.897038313	0.002525311
169.8186721	1342.800526	347.8068522	7.347283219	2.355063173	0.002538095
0	0	0	0.515580886	-4.03491879	0.002538414
45.93472991	92.72344807	46.10826001	5.486754304	1.528868929	0.002562923
195.1339049	504.6472564	609.7461773	8.140167315	1.254341799	0.002587394
4716.605616	5929.385851	4208.376177	11.82582171	1.838967389	0.002597619
2.086993635	0	0	0.164986979	-3.69812073	0.002601867
1694.638832	1117.245143	595.0012484	9.455381505	1.810381758	0.002604244
159.6550131	163.2424169	81.53078331	7.484577535	-1.04759791	0.002608696
46.95735679	27.20706948	199.4902145	3.714084035	3.436814216	0.002609685
6.678379633	3.51058961	5.204092551	-8.70511228	9.789915001	0.002633715
48.00085361	74.60002921	55.51032055	5.861825267	1.662466866	0.002635699
70.9577836	8.776474025	32.09190407	5.462643483	-1.49758189	0.002640437
27.01613261	43.34700521	47.60009987	4.39498009	1.321653782	0.002656673
307.8315612	54.41413896	162.1942179	8.397563207	-1.02183398	0.002659376
8.347974541	0.877647403	15.61227765	2.935432985	-3.7828548	0.002671519
5.217484088	19.30824286	4.336743793	2.228279572	2.521065211	0.002671646
408.0072557	188.6941915	287.9597878	8.483222548	-0.71158832	0.002691295
135.6545863	150.9553532	74.59199324	7.859700995	-1.152681	0.00269238
0	0	0	-8.70511228	-7.96583256	0.00269375
73.04477724	152.710648	219.4392359	6.938280268	1.973073575	0.00271636
1.043496818	0	0	-1.0403602	-3.39598418	0.002720328
22.95692999	44.76001753	14.7449289	4.222507064	1.845736579	0.00272579

2.086993635	1.755294805	0.867348759	2.015936187	-2.44234138	0.00272984
12.16717289	38.94999172	0	-8.70511228	11.8188388	0.002751935
453.9211157	2296.803252	900.3080114	9.304919615	1.215881577	0.002775798
126.2631149	154.4659428	65.91850565	7.302050207	-1.01961645	0.002800139
0	0	0	-2.73227357	-7.55111982	0.002809195
285.918128	7.898826623	154.388079	7.935070029	-2.54630669	0.00283971
225.3953126	431.802522	338.2660158	8.352720957	0.867591135	0.002839756
437.1312519	192.4505224	936.4157402	8.219736503	1.926190175	0.002849141
22.95692999	14.04235844	13.01023138	5.356772434	-2.15708363	0.002855864
0	0	0	-0.82594381	-3.88872347	0.002869755
0	0	0	-0.14626698	-3.29859598	0.002874087
72.00128042	48.27060714	190.8167269	5.490954101	1.351226086	0.002882523
270.2656758	33.3506013	206.4290045	7.662140558	-1.66324396	0.002887898
3.130490453	5.265884415	0	-8.70511228	9.272576243	0.002889923
90.78422314	157.9765325	142.2451964	6.169058485	1.151379269	0.002905726
856.7108873	3474.606067	1320.972159	10.04996088	1.435178857	0.002910947
1297.066544	566.0825746	303.5720655	10.27619697	-1.05418046	0.002936334
274.439663	357.2024928	392.9089876	8.141313873	1.966044349	0.002946824
378.7893448	458.1319441	470.9703759	7.871231995	1.294019381	0.002950826
633.4025683	419.5154584	838.7262495	9.827396557	-1.1100845	0.002970919
0	0	0	0.006384919	-3.66041814	0.002981504
289.0486185	294.8895272	309.6435068	8.345656341	-0.88713515	0.002985618
45.91385998	78.11061882	62.44911062	5.477054949	1.584034917	0.003001011
2428.217095	639.8049564	573.3175294	10.81334388	-1.18045575	0.003009675
7.304477724	21.06353766	3.469395034	2.818198788	2.95063803	0.003028211
238.9607712	529.2213837	2089.443159	8.60294385	2.201735067	0.003030544
2821.615395	2399.487998	3493.6808	11.07888559	0.956876925	0.003035165
1784.379558	958.3909635	1052.094044	9.155254983	1.928097716	0.003047291
0	1.755294805	0.867348759	-8.70511228	9.183769291	0.003048132
9.391471359	6.143531818	10.4081851	2.515396098	2.105813643	0.003053143
664.7074729	1502.532353	592.3992021	8.516910961	1.374657066	0.003055007
29.21791089	64.94590779	66.78585441	5.451519994	1.089987529	0.003060145
90.78422314	45.63766493	106.6838973	5.236268635	1.324659072	0.003062999
14.60895545	35.1058961	9.540836344	5.091823897	-1.58212873	0.003064575
195.1339049	340.263898	355.3701334	8.634197747	-0.89626921	0.00306854
41.73987271	10.53176883	50.306228	6.090920765	-1.31874902	0.003084606
34.43539498	92.15297726	52.90827427	4.6757744	1.79642885	0.003092261

174.9526764	76.36410049	112.5298279	5.786950517	1.832675707	0.00312104
81.39275178	129.8918156	138.7758014	6.182123164	1.267037966	0.003122042
2.086993635	42.12707532	15.61227765	3.193877894	2.923768155	0.003137502
18.78294272	56.16943376	69.38790069	4.257746552	2.052384392	0.003137733
687.6644028	364.223672	285.3577416	9.587965211	-1.11767864	0.003139167
7.304477724	1.755294805	0.867348759	2.572743913	-2.23887346	0.00314027
410.0942493	40.37178052	93.67366593	8.406157513	-1.30290035	0.003143352
88.6972295	309.8095331	538.6235791	7.398348916	1.487951797	0.003151072
160.6985099	280.8471688	184.7452856	7.317492055	0.76711114	0.00315299
34.43539498	56.16943376	101.4798048	5.582759591	1.127477586	0.003153724
305.7445676	44.76001753	75.459342	8.321670015	-1.45458824	0.003163531
78.26226132	263.2942208	244.5923499	6.450246547	1.439861849	0.003171911
4411.904545	1061.07571	1386.890665	11.47803312	-1.16138225	0.003178725
1364.893838	536.2425629	603.674736	9.870008801	-0.97918749	0.003217072
4.173987271	1.755294805	4.336743793	3.270030691	-1.83429078	0.003223801
11.47846499	98.85820342	134.9854873	5.004424349	1.869201689	0.003243552
94.95821041	297.5224695	130.9696625	6.723645835	1.090168405	0.003244495
12.52196181	395.8189785	64.18380813	6.34373367	3.033901931	0.003246306
83.01017185	129.0492741	96.9782647	6.396913914	1.416354789	0.003246735
21.91343317	214.1459662	146.5819402	7.363577735	2.82911518	0.003263574
136.7919978	135.3332295	240.2556061	7.316865454	1.326647636	0.003272075
16.69594908	63.19061298	58.97971558	3.966574121	2.044540773	0.003272275
3.130490453	0	0	2.140589015	-3.375538	0.003292402
124.1761213	34.2282487	45.10213545	6.654820968	-1.27695999	0.003299205
39.65287907	13.16471104	7.806138827	5.203283473	-1.31695492	0.003303638
16.69594908	0.877647403	4.336743793	5.244823167	-2.98568865	0.003318659
915.1467091	294.8895272	288.8271366	9.862838235	-1.24513186	0.003340254
482.0955298	256.2730415	252.3984887	8.668631683	-0.81833254	0.003344267
29.21791089	40.37178052	19.08167269	4.407936464	1.493117987	0.00337079
29.21791089	121.64193	42.30059896	4.927263836	1.482599025	0.003388248
934.9731486	464.2754759	943.6754493	8.078447284	1.767059723	0.003389377
36.52238862	0	49.43887924	5.678986471	-3.08248396	0.003391715
442.4426507	909.242709	1037.349115	8.99052023	0.898249547	0.00343581
14.60895545	26.32942208	39.03069414	4.406906104	1.478998812	0.003447861
3.130490453	14.92000584	2.602046276	1.609124603	3.720314011	0.003456879
705.0594948	377.7921009	637.4839906	9.072188713	0.865245528	0.003475978
22.95692999	84.25415064	31.22455531	4.627146819	1.589135197	0.00347654

84.52324223	155.3435902	180.4085418	6.45172695	0.875330419	0.003482019
425.7467016	399.3295681	622.7564087	8.467282152	1.054003708	0.003488611
230.6127967	439.7013487	369.4905711	6.675678246	1.815928596	0.003489605
524.8788993	49.14825454	44.23478669	8.438110935	-2.24152428	0.003493585
8.347974541	58.80237597	164.7962641	2.804314283	6.080465068	0.003500811
6.260980906	18.43059545	9.540836344	3.287017444	2.35880314	0.003501182
5880.104568	21717.38498	6879.810353	13.78528156	-2.65559569	0.003526157
3136.751434	479.1954818	111.0206411	5.908466551	6.186667212	0.003528179
1301.74141	694.9036604	347.7548113	9.694782565	-0.93986847	0.003540395
589.575702	758.2873558	241.1229549	9.365529521	-0.97313489	0.003548208
9549.018509	2581.134681	2310.582399	13.35519276	-1.25732005	0.00356869
1.043496818	0	1.734697517	0.78653082	-2.76876713	0.003575302
108.523669	154.4659428	106.6838973	7.337379286	-0.92756748	0.003595465
16.69594908	7.898826623	33.82660158	4.247597233	1.47829544	0.003597763
2516.914324	631.0284824	1097.19618	11.07581476	-0.93552137	0.003620467
2342.650356	3066.500024	3998.477777	10.67411809	1.295825771	0.003626869
206.6332398	710.1835016	442.3652138	7.966812187	1.066369591	0.003637518
2.086993635	0.877647403	7.806138827	2.261548289	-2.42027481	0.003647467
98.08870086	107.0729831	145.7145914	7.213371793	1.782115861	0.003649511
0	17.55294805	0.867348759	2.156526713	3.3901144	0.003650858
2.086993635	97.41886168	26.02046276	1.010743109	5.663247721	0.003656848
130.4371022	197.4706656	305.306763	7.264069163	0.838749386	0.003670602
638.6200524	1397.214665	1615.003388	9.522270476	1.020368103	0.003684669
19.82643954	0	6.07144131	5.784534684	-2.86035707	0.003696826
144.3469148	157.4148381	88.95528868	7.332896537	-0.68240119	0.003697877
0	0	0	-8.70511228	-7.4955364	0.003706721
0	56.16943376	246.3270474	-8.70511228	14.43754467	0.003715996
6.260980906	7.02117922	7.806138827	2.946652324	1.849581802	0.003734913
24.00042681	78.98826623	531.684789	6.688900756	3.498448102	0.003758464
175.3074654	219.4118506	96.2757122	7.152418542	1.159444048	0.003758676
285.918128	821.4779687	453.6234007	8.786446141	0.700786944	0.003764651
322.4405167	759.1650032	825.7160182	8.367284011	1.012626327	0.003774205
40.69637589	99.17415648	71.98994696	5.904347894	1.314434318	0.00377985
760.7091801	1000.518039	544.6950204	8.919705884	1.203533561	0.003780578
8.347974541	21.06353766	16.47962641	-8.70511228	10.60792663	0.003793521
259.8202726	1207.642826	398.6334894	8.424477211	1.381507634	0.003796026
3.130490453	1.755294805	0.867348759	3.193947192	-2.20517606	0.003823628

2918.660599	2211.671454	312.2455531	12.10154568	-1.4588041	0.003824928
540.5313515	186.0612493	220.3065847	8.43203138	-0.91474824	0.00382606
0	0	0.867348759	2.326583433	-3.23135272	0.003826504
0	0	0	-0.82762456	-3.48595018	0.003831778
248.4565923	286.2622533	538.1725577	7.622440527	1.179571636	0.003836616
173.7317852	321.8684084	264.09035	7.047567382	1.204919475	0.003841278
1384.720277	451.1107649	749.3893274	10.39636769	-0.89186473	0.003872594
474.791052	131.6471104	455.3580982	8.182861319	1.678054369	0.003875791
355.8324148	117.6047519	172.602403	8.048449249	-0.91230983	0.003879853
437.2251666	134.2800526	476.1744685	5.099886613	3.667004889	0.003895022
211.829854	289.6236428	132.7043601	8.24315352	-0.81346135	0.003902638
3.130490453	20.18589026	5.204092551	2.158985414	2.931679838	0.003908287
238.9607712	252.7624519	201.224912	7.582002345	1.810314093	0.003914387
2.086993635	0.877647403	11.27553386	0.161886107	3.838461081	0.003943921
509.5812359	171.132467	348.8997116	7.392107922	1.624049614	0.003946757
0	0	0	0.154895023	-3.2521395	0.003947168
9.391471359	24.57412727	3.469395034	2.77406838	2.588391069	0.003961674
146.0895545	159.7318273	167.3983104	6.610706488	0.788324419	0.003962197
10042.61337	8429.803301	15963.5539	12.40929929	1.911125948	0.003971203
29.21791089	299.2777643	164.7962641	5.366785314	2.541218007	0.004009986
625.0545938	574.8590486	640.9707326	9.340583038	-0.65589699	0.004022788
0	0	0	-1.87944917	-3.91502094	0.004039672
0	0	0	-1.79838736	-4.05083849	0.004039672
0	0	0.867348759	-1.97135247	-4.08263666	0.004039672
0	0	0	-1.89322009	-4.36330797	0.004039672
0	0	0	-1.99658777	-4.37863523	0.004039672
0	0	0	-1.85787334	-4.88162235	0.004039672
0	0	0	-2.02420971	-4.60497837	0.004039672
149.3974394	68.60569745	90.36039367	6.934545006	-0.84482962	0.004040175
75.13177087	49.14825454	111.0206411	6.854164156	-1.18290823	0.004048587
98.08870086	22.81883247	62.44911062	6.279847737	-1.01357498	0.004053732
94.95821041	42.12707532	151.7860327	6.064919557	1.143097198	0.00406419
153.3940322	529.2213837	202.0922607	7.761425124	1.143664297	0.004064345
493.5739948	459.8872389	215.1024921	8.477873131	-1.04846733	0.004079896
19.82643954	26.32942208	27.75516027	4.706170586	1.096163873	0.004094038
2.086993635	19.30824286	5.204092551	1.448737554	2.741352518	0.004097758
1380.54629	709.1391012	1455.411217	10.58162545	-0.99357789	0.004113484

477.9215425	459.0095915	311.3782043	9.017311029	-0.65320522	0.004118835
1724.90024	819.7226739	379.0314075	11.08650318	-1.26503401	0.004118892
115.8281468	258.9059837	138.7758014	6.722754302	0.990846409	0.004124153
60.52281542	19.30824286	45.9694842	5.214711625	3.792905817	0.00413695
4.173987271	6.143531818	0.867348759	0.309071079	4.372119674	0.004158792
273.3961662	225.5553824	294.8985779	7.799708606	0.968021226	0.004159171
16.69594908	114.0941623	39.03069414	4.887101025	1.982507723	0.004175298
61.56631224	262.4165733	234.1841648	7.076116648	1.980845224	0.004183379
257.743714	42.12707532	234.1841648	5.888245138	1.741677487	0.004188938
49.04435043	40.37178052	39.03069414	5.40062664	1.010418817	0.004193046
227.4823063	355.447198	346.0721547	8.192083282	0.684703091	0.004224287
120.002134	268.5601052	232.4494673	6.993615379	0.936670676	0.00425353
521.7484088	447.6001753	431.072333	8.445966226	1.07240965	0.004261249
49.04435043	158.8541799	45.10213545	5.3721719	1.564385683	0.004262165
142.959064	50.02590194	121.4288262	7.132283113	-0.86636411	0.004287556
3.130490453	0.877647403	6.07144131	0.179583	4.000927502	0.004294928
0	7.02117922	45.10213545	1.380799887	4.51557729	0.004295384
32.34840135	31.59530649	13.87758014	5.141670666	-1.63981835	0.004297067
3705.175455	4569.462425	2024.105777	11.57806606	1.241191113	0.0042976
852.5369	146.5671162	717.2974233	9.868917237	-1.50312472	0.004302666
10.43496818	12.28706364	26.88781152	3.428955763	1.805150128	0.004315206
624.011097	3155.142412	1374.747782	9.703381354	1.56021292	0.004317781
92.87121677	15.79765325	169.1330079	7.177774025	-1.45851756	0.004320956
247.3087458	24.57412727	176.071798	7.379821302	-1.28037552	0.004326516
369.3978735	615.2308292	287.9597878	8.979263717	-0.85308907	0.004331202
50.08784725	81.62120843	98.87775848	5.744875362	1.528156793	0.004334729
44.87036316	164.9977117	73.72464448	5.928880468	1.022744698	0.004344745
13.56545863	60.55767077	226.378026	5.057253329	2.434500158	0.004360721
18.78294272	236.0871513	136.1737551	6.744456793	2.788181341	0.004384431
12.52196181	52.65884415	25.153114	4.23610777	1.583225201	0.004388523
313.0490453	215.901261	349.5415497	8.765082979	-0.80014294	0.004395274
0	0	0	-8.70511228	-8.74611184	0.004400268
139.8285736	158.8541799	136.1737551	6.74286161	0.638276895	0.004405402
20.86993635	246.6189201	18.21432393	3.72983346	4.238669358	0.004405804
41.73987271	104.4400409	36.42864786	4.969142597	1.291013016	0.004411691
0	0	0	-0.98033033	-3.82139299	0.004418716
8.347974541	0	4.336743793	2.092350054	-2.30151262	0.004420792

2.086993635	2883.949365	5.204092551	5.656682106	5.856988715	0.004433556
103.932283	158.9946034	52.21439527	6.423111845	2.586127097	0.004434202
302.6140771	307.1765909	287.9597878	8.408511047	-0.59363975	0.004435627
1.043496818	0.877647403	1.734697517	-8.70511228	7.91148591	0.004437044
1081.062703	1438.464093	1631.483015	9.901762977	0.962704419	0.004438094
196.1774017	77.23297142	31.22455531	7.393798357	-1.17552964	0.004440302
0	0	0	-8.70511228	-8.96375746	0.004449942
15.65245226	2.632942208	1.734697517	4.334089301	-2.39025141	0.004450033
34.43539498	87.76474025	20.81637021	5.166623337	1.508046577	0.004452851
595.8366829	301.9107065	520.4092551	9.175817162	-0.62370349	0.004457365
4.173987271	0	3.469395034	3.114059958	-2.27453976	0.004466391
41.73987271	66.70120259	27.75516027	4.644406656	1.442352114	0.004492859
606.2716511	471.2966551	321.7863894	9.208218106	-0.59917933	0.004495672
61.56631224	40.37178052	117.0920824	6.934193436	-1.1588235	0.004505848
73.04477724	115.8494571	74.59199324	6.99618719	-0.95831819	0.004536575
648.0115238	1091.793369	154.388079	10.75401216	-1.66094927	0.004549866
1.043496818	0	0	-0.2020095	-3.04595793	0.004555085
60.52281542	112.3388675	138.7758014	5.924621473	1.102788113	0.004570696
17.7394459	13.16471104	34.69395034	3.974391463	1.379345254	0.004579402
113.7411531	272.0706948	288.8271366	7.180343884	1.229088733	0.004580244
103.2122702	92.96918935	97.01295865	8.037846862	-1.69647258	0.004585414
184.6989367	426.5366376	302.7047167	7.853353167	1.229326425	0.004600557
17.7394459	7.898826623	12.14288262	4.17342821	-1.26125436	0.004608786
618.7936129	466.0307707	372.9599662	8.867143587	0.961077868	0.004618151
55.30533134	107.9506305	77.19403951	7.175542833	-1.21383553	0.004641221
0	14.04235844	1.734697517	1.754117931	7.262213378	0.004641536
679.3164283	939.0827207	961.8897732	9.513169238	2.43810641	0.00464763
10.43496818	114.0941623	176.9391467	4.558752583	2.781853318	0.004650915
79.30575814	8.776474025	58.97971558	6.281481722	-1.44770743	0.004651231
0	0	0	-0.68084393	-3.51781046	0.004666726
0	0	0	-0.15693621	-2.88579993	0.004669111
54.26183452	89.52003506	45.10213545	5.767216649	0.925195484	0.004675017
0	0	0	-8.70511228	-8.21242937	0.004677482
1.043496818	0.877647403	0.867348759	2.072713394	-2.05352885	0.004684685
3977.809869	2446.880958	5481.644154	11.33738763	1.016290942	0.004720489
554.0968102	1162.882808	491.7867461	9.198366621	0.877089719	0.004722583
74.08827405	37.73883831	81.53078331	6.489791602	-1.42996828	0.004727112

12.52196181	49.14825454	44.23478669	3.613068241	3.068873517	0.004740449
7.304477724	7.02117922	3.469395034	-8.70511228	10.7453994	0.004740762
0	0	0	-0.1647321	-3.34445507	0.004753132
475.8345489	75.47767662	236.7862111	6.566767713	2.691796381	0.004757712
1.043496818	0.877647403	1.734697517	-8.70511228	8.812215922	0.004768092
151.3070386	177.2847753	205.5616558	7.119968486	0.714471026	0.004771097
2.086993635	1.755294805	2.602046276	-8.70511228	8.414316942	0.004771941
106.4366754	162.3647695	124.8982212	6.646366606	0.805814799	0.004781891
107.4801722	174.6518331	122.296175	5.982382933	1.069473689	0.004782462
18.78294272	30.71765909	44.23478669	4.295402622	1.470512409	0.004811663
53.2183377	121.9929889	111.8879899	5.754382257	1.238907403	0.004814438
435.138173	499.381372	603.674736	8.097256546	1.161493335	0.004831196
117.9151404	235.2095039	199.4902145	7.038656656	0.990227186	0.00484273
106.4366754	14.04235844	1.734697517	6.173334487	-3.03837286	0.004870781
55.30533134	114.0941623	45.9694842	5.957849276	1.247861813	0.00488296
37.56588544	80.74356103	49.43887924	5.977701359	1.261846192	0.004890877
141.9155672	93.03062467	199.4902145	7.766404107	-0.73181265	0.004891488
5.217484088	6.143531818	3.469395034	1.62261638	2.269371818	0.004905416
289.0486185	503.769609	635.76664	8.070443496	1.221075204	0.004941815
1.043496818	6.143531818	25.153114	2.325112675	2.727816783	0.004948276
765.9266642	365.1013194	299.2353217	9.283366901	-0.7617672	0.004983798
0	2.632942208	0	-8.70511228	9.04697598	0.004998217
28.17441408	3.51058961	15.61227765	4.667760378	-1.23678678	0.005009601
4.173987271	3.51058961	9.540836344	2.326589853	2.701801017	0.005014622
0	0	0	0.471964091	-2.94261725	0.005016118
128.3501086	488.8496032	104.9491998	9.356678893	-2.32939087	0.005036486
2.097428604	0	2.662760689	-8.70511228	8.807504199	0.005046199
4.173987271	3.51058961	9.540836344	0.293903947	4.341785411	0.005053425
104.3496818	224.677735	130.9696625	6.789070376	1.047267061	0.005053622
4.173987271	0	0	1.867603199	-3.09429343	0.005079221
17.7394459	31.59530649	38.16334538	5.013909791	2.430572247	0.005079403
0	0	49.43887924	1.642986089	4.547089917	0.005099962
0	4.388237013	2.602046276	0.125997262	3.893756027	0.00511527
3.130490453	30.71765909	1.734697517	2.054647507	2.665770996	0.005134253
170.0899813	86.88709285	139.6431501	5.586335747	1.501299157	0.005158081
5.217484088	0	1.734697517	2.938148567	-2.01141833	0.005185882
0	0	0.867348759	-1.99050256	-4.04433648	0.005189569

395.4852939	269.4377526	244.5923499	8.62346646	-0.70731764	0.005201359
526.9658929	40.37178052	78.06138827	7.824203291	-2.31560694	0.005204088
281.7441408	303.6660013	337.3986671	7.901523082	0.698197326	0.005220731
434.0946761	165.8753591	244.5923499	8.404595091	-0.8536034	0.005223649
1289.177708	1546.046111	1125.636545	11.75400587	-1.1353578	0.005238259
906.7987345	433.5578168	317.4496456	9.617184996	-0.97226704	0.00524306
1.043496818	0	0.867348759	0.045006604	-3.08897229	0.005259049
820.1884987	860.9721019	875.1548974	10.39595993	-0.88544962	0.005259912
49.04435043	65.82355519	85.00017834	5.793389589	0.943047872	0.005271713
102.2626881	266.8048104	307.0414605	7.45261183	0.846366569	0.005288668
1936.730094	388.7977993	1028.675628	11.07394717	-0.97809511	0.005297109
318.2665294	805.6803155	941.9407518	8.973184528	0.98868955	0.005302809
208.6993635	126.381226	44.23478669	8.61496042	-1.4794442	0.005305908
1833.246514	886.4238765	366.0211761	10.581179	-1.32837628	0.005305994
788.8835942	533.6096207	176.071798	9.763690592	-1.09683207	0.005314554
6.260980906	0.877647403	0	3.443958682	-2.81104014	0.005315012
0	0	0	-0.28612133	-3.25727973	0.005315882
168.0029876	562.571985	381.6334538	8.042476374	0.970937512	0.005319856
81.39275178	215.901261	123.1635237	6.830035994	1.011086216	0.005330728
172.1769749	127.2588734	211.6330971	7.029080452	0.937243363	0.00535698
667.1909953	341.5891455	158.7334963	9.639723724	-1.52513791	0.005357786
1.043496818	2.632942208	2.602046276	0.757552235	3.358981881	0.005359527
65.74029951	5.265884415	28.62250903	6.31113728	-1.92227821	0.005370911
379.8328416	365.1013194	642.7054301	8.30246268	1.387917195	0.005375324
128.3501086	65.82355519	60.7144131	6.475228857	-0.85386411	0.00539121
25.04392362	42.12707532	26.02046276	4.517462038	1.226456999	0.005414021
116.8716436	229.9436195	252.3984887	7.339362962	1.142639145	0.005416298
1.043496818	0.877647403	0	0.188973772	-2.59590953	0.005428275
127.3066118	240.4753883	260.2046276	7.095562873	0.912808926	0.005430376
1111.324111	1752.661863	2490.158286	9.804755572	0.996162858	0.005442923
25.04392362	33.3506013	44.23478669	4.432972912	1.394497313	0.005457242
5.217484088	24.57412727	35.5612991	4.040546441	1.479738556	0.00546349
136.6980831	252.7624519	421.5314967	7.5595645	1.023622684	0.005499346
3.130490453	104.4400409	196.0208194	2.403272767	6.503869551	0.005523672
247.3087458	146.5671162	132.7043601	8.290101114	-1.05538383	0.005525997
79.30575814	125.5035786	138.7758014	6.652880057	0.951266883	0.005540575
0	0.877647403	0.867348759	1.362098274	-3.22212198	0.00554128

6835.947653	16930.69604	8365.578776	12.25675664	1.792060544	0.005552451
156.5245226	368.6119091	248.0617449	7.764218079	1.344645049	0.005569516
5.290528866	0	0	1.021758078	-2.82205982	0.005584694
63.65330588	147.4447636	158.7248228	6.351834623	1.07223444	0.005587012
3201.448237	4941.154876	9316.193016	11.37734389	1.311569217	0.005589871
2.086993635	49.14825454	0.867348759	1.21811324	4.372895115	0.005593446
403.8332684	445.8448805	971.4306096	9.618112389	-1.31241189	0.005596326
0	0	0.364286479	-0.25791036	-2.61069761	0.005602206
517.5744216	832.887385	1620.207481	9.206183507	0.967743987	0.005615452
26.08742044	0	4.336743793	4.959427465	5.88520143	0.005620839
160.6985099	280.8471688	175.2044492	8.505343856	-0.91890674	0.005623424
108.523669	300.1554117	139.6431501	6.431814355	1.330461606	0.005625937
1.043496818	0	0	-1.78340474	-3.92504797	0.005633046
4.173987271	21.94118506	2.602046276	2.098209766	4.900971981	0.005644307
209.7428604	108.8282779	181.2758905	8.054754774	-0.92853197	0.005654358
1.043496818	56.16943376	53.77562303	3.247465718	2.971900579	0.005655912
1.043496818	0	0	2.153839735	-2.46218298	0.00567287
98.08870086	607.3320025	156.1227765	7.111251985	1.267828756	0.005689202
526.9658929	107.9506305	1007.859257	9.70635752	-1.35652758	0.005690255
3.130490453	0	0	2.161782403	-2.67756384	0.005701035
18.78294272	2.632942208	45.10213545	4.931623338	-2.23516683	0.005710014
499.8349757	1019.826282	432.8070305	8.901961321	0.870629382	0.005713773
292.1791089	210.6353766	46.83683296	8.429251408	-1.1509106	0.00572594
327.6580007	529.2213837	549.8991129	8.279624239	0.73682628	0.005731082
257.743714	14206.4785	1098.063528	8.676237497	4.816493194	0.005734069
9.391471359	28.08471688	21.68371896	3.659590077	1.556138455	0.00574449
15.30809832	4.572542967	5.412256253	1.398155615	2.418466561	0.005756525
520.704912	290.5012902	638.3686863	7.686939999	1.147288318	0.005757665
3.130490453	2.632942208	3.469395034	0.630357139	3.014691795	0.005769452
44.87036316	74.60002921	67.65320317	5.495680191	0.948422817	0.005801172
5.217484088	19.30824286	21.68371896	1.819495422	3.318605719	0.005803933
277.5701535	384.4095623	451.8887032	8.143841691	1.367416326	0.005825972
2.086993635	0	0.867348759	0.35782169	-2.70194736	0.005829924
105.3931786	69.3341448	635.76664	6.521932497	2.301914318	0.005845478
151.3070386	116.7271045	176.9391467	7.587607102	-0.77703564	0.005878666
0	0	0	-8.70511228	-8.31201449	0.005901009
321.3970198	312.4424753	329.5925283	7.859547695	0.622712588	0.005917299

60.52281542	352.8142558	85.8675271	6.938914087	3.474357479	0.005919929
0	0	0	-8.70511228	-7.87609535	0.005925831
376.7023512	150.0777058	208.1637021	8.933979422	-1.5282262	0.005934997
523.8354025	1190.089878	418.0621016	8.536413681	1.040992514	0.005940189
517.5744216	418.637811	210.7657483	9.057656927	-0.86333228	0.005941972
7.304477724	7.02117922	3.469395034	1.930983052	2.566518552	0.005949305
1.043496818	0.877647403	0.867348759	-8.70511228	8.448963588	0.005966346
10.43496818	26.32942208	0.867348759	4.122084769	-2.46779165	0.005981064
0	3.51058961	3.469395034	1.511592497	3.445527528	0.005983326
72.00128042	139.545937	144.8472427	6.443039982	0.81683587	0.005989672
1215.673793	939.0827207	1111.07376	9.796325061	0.694406066	0.00600735
0	0	0	-8.70511228	-7.90530955	0.006026789
0	0	0	-8.70511228	-7.90530955	0.006026789
96.00170723	154.4659428	312.2455531	7.075292758	1.079515789	0.006030085
276.5266567	129.8918156	171.7350542	8.125666854	-0.96565269	0.006032768
8.577543841	1.316471104	0.876022246	2.233693776	-2.53082319	0.006033446
1.043496818	0	0	-1.12729137	-3.78274624	0.006039238
473.7475552	1288.386387	804.0322992	9.131409124	0.771077633	0.006040567
115.8281468	172.0188909	215.1024921	7.292937507	0.809423818	0.006048361
8.347974541	6.143531818	16.47962641	2.361423298	2.557394532	0.006048723
0	39.49413311	3.469395034	2.245148003	4.259966362	0.006072048
103.3061849	86.00944545	104.081851	6.074890743	1.455712182	0.006084371
570.7927593	14.92000584	81.53078331	4.477504519	3.507673633	0.00609165
306.7880644	713.5273382	247.1943962	9.136706484	-0.89995247	0.006096924
213.9168476	1503.41	1122.349294	9.38147008	1.911921436	0.006098875
475.8345489	676.6661473	377.29671	8.684013525	0.777186809	0.006112288
68.87078997	144.8118214	148.3166377	6.451235691	1.019770256	0.006112681
360.0064021	23.69647987	198.6228657	7.704887783	-1.73163417	0.006126648
179.4814526	37.73883831	113.6226874	7.354442055	-1.36850133	0.006132782
657.4029951	155.3435902	346.9395034	9.026293098	-1.1215929	0.006154342
246.265249	183.4283071	458.8274933	7.968069191	0.978584163	0.006189914
85.56673905	16.67530065	124.8982212	5.480397378	1.587660676	0.006214107
48.00085361	90.39768246	104.9491998	5.850342372	0.837209106	0.006223024
42.78336952	162.2770047	41.06029023	5.947378204	2.078831811	0.006224447
116.8716436	72.84473441	78.06138827	6.901970626	-0.61119226	0.006225097
77.21876451	1166.770786	140.6752952	7.796688798	2.607835118	0.006231698
274.439663	315.0754175	241.1229549	8.455515479	-0.81457626	0.006233111

1.043496818	5.265884415	1.734697517	1.960486115	-2.763735	0.006246436
73.04477724	93.90827207	105.8165485	5.794371351	1.00541505	0.006251063
67.82729315	165.8753591	145.7145914	6.180387558	0.970403593	0.006273283
84.52324223	55.29178636	52.90827427	6.222779668	1.333668308	0.006295679
320.353523	852.1956278	612.3482235	8.7806346	0.883464774	0.006316598
10.43496818	8.776474025	12.14288262	4.239308954	-1.31803786	0.00632436
393.3983003	221.1671454	200.3575632	8.612547179	-0.85828702	0.006327792
1181.238398	2228.346755	4267.355892	11.82235379	-1.12507116	0.006336557
1094.482072	978.3047831	1297.319559	9.386778793	0.816778647	0.006338755
221.2213253	76.35532402	384.2355	6.213159657	1.65288222	0.006356328
135.6545863	277.3365792	176.071798	7.436924132	0.649487182	0.006367929
132.5240958	265.0495156	204.694307	7.42583347	0.652224758	0.006375498
216.0038413	41.24942792	431.9396818	8.668604453	-1.69991364	0.00637725
98.08870086	63.19061298	19.08167269	7.570584906	-1.30011759	0.006384394
0	0	0	-8.70511228	-8.0092712	0.006384657
202.4383826	171.1412435	343.4701084	7.9157489	-0.93174569	0.006392298
111.6541595	0	281.8883465	2.967841134	9.689659785	0.006411657
0	0	0	-1.03491845	-3.42616342	0.006418614
57.39232497	169.3859487	274.3684328	7.436257831	1.786584693	0.006419314
6.260980906	185.1836019	82.39813206	5.55746909	2.734327493	0.00642902
48.00085361	104.4400409	165.6636129	6.101118244	1.171188467	0.006462019
2306.127967	2080.901991	1385.155967	11.5602994	-1.041523	0.006482663
2687.004305	20.18589026	18.21432393	9.930143152	-2.81222976	0.006500729
153.3940322	173.7741857	122.296175	7.545011023	-0.62015034	0.006504163
1584.028169	1131.287502	811.838438	10.30909019	-0.62888412	0.006504965
73.04477724	75.47767662	118.8267799	5.794746926	0.89689935	0.006524076
4289.815417	6235.684795	9314.458318	11.66905895	1.137120366	0.006540477
854.6238937	478.3178344	327.8578307	8.058089481	1.58094416	0.006557284
7.304477724	120.2376941	3.469395034	4.36844476	2.402780447	0.006560129
20.86993635	4.388237013	29.48985779	4.097150927	-1.68413749	0.006581411
135.6545863	284.3577584	499.5928849	7.458269368	1.123220511	0.006604817
1739.509195	1058.442767	191.6840756	10.69691169	-1.36169545	0.006622124
5.217484088	14.92000584	4.336743793	2.324345008	2.009075946	0.006630324
49.04435043	104.4400409	57.24501807	5.536455996	1.374285492	0.006630495
0	96.54121428	12.14288262	4.560045157	3.202013815	0.006633924
382.9633321	426.5366376	734.6443985	8.805355449	1.01408094	0.006635915
11.47846499	64.94590779	12.14288262	3.572038541	2.604318364	0.00664294

1606.985099	683.6873266	763.2669075	10.64576208	-1.0575986	0.006659506
19.82643954	28.08471688	79.79608579	6.044640539	-1.43153617	0.006673872
269.222179	427.414285	432.8070305	8.019418246	0.834690597	0.00668615
697.0558742	1053.176883	863.8793635	9.704800004	0.799589691	0.006706186
245.2217522	453.7437071	307.0414605	8.149079843	0.583106075	0.006708733
31.30490453	2.632942208	6.07144131	5.93261625	-2.4741475	0.006710833
0	0	0	-8.70511228	-7.54181475	0.006718161
293.2226058	0	1150.971803	4.515742134	8.063781117	0.006719737
74.08827405	97.41886168	42.50008917	6.365399126	1.268811652	0.006754652
618.7936129	2.632942208	740.7158398	5.821461445	5.54254978	0.006758651
2.086993635	7.898826623	26.88781152	1.846743823	2.275896905	0.006760762
604.1846574	459.0095915	281.8883465	9.600856001	-0.73176788	0.00676143
189.9164208	261.5389259	278.4189515	7.196972475	0.907321351	0.006770164
2.086993635	7.898826623	3.469395034	1.934714633	2.432840948	0.006773247
353.7454212	265.927163	333.929272	6.346203662	2.255048622	0.006798994
284.8746312	251.8848045	165.6636129	8.131782686	-1.00570549	0.006822515
185.7424335	543.2637422	282.7556953	6.985889451	1.389637994	0.006824625
131.824953	4421.552508	10129.02023	9.512347026	4.579880157	0.006825694
113.7411531	406.3507474	268.0107664	7.3161025	0.945194882	0.006841932
611.4891352	281.7248162	719.8994696	9.088941709	-1.020007	0.00684379
316.1795358	252.7624519	693.8790069	6.995192387	1.44929133	0.006863704
3.130490453	6.143531818	24.28576524	1.907270236	2.922690004	0.006869618
1764.553119	1544.650652	1035.614418	10.92909506	-0.55746359	0.006904294
232.6997903	275.5812844	437.1437743	7.770074484	0.950975948	0.006925716
468.5300711	33.3506013	365.1538274	8.417040063	-1.28826443	0.006933697
2365.607286	848.6850382	1197.808636	10.62382507	-1.06549609	0.006935884
125.2196181	251.0071571	185.6126343	7.208610723	0.899797498	0.006965659
794.1010782	989.98627	1896.891735	9.254581076	1.304597869	0.006982926
77.21876451	66.70120259	149.1839865	6.515070243	1.250283257	0.006988363
5.217484088	0	0.867348759	2.579509794	-2.68929189	0.006995544
35.4788918	0	222.0412822	5.281045999	5.080594827	0.00700799
7.304477724	4.388237013	4.336743793	3.665882721	-1.80613488	0.007055538
150.2635417	164.9977117	202.9596095	6.924904767	0.814449258	0.007055583
180.5249495	155.3435902	235.0515136	7.871179137	-0.72523802	0.007055678
19.82643954	0	53.77562303	-8.70511228	13.16849826	0.007066081
149.2200449	243.1083305	93.67366593	4.940534688	2.685009663	0.007069094
246.265249	190.4494863	163.0615666	6.993316495	0.651070314	0.007090332

261.9177012	130.769463	311.3782043	7.440276112	0.870322337	0.007114057
0	0	0	-0.79600427	-3.54919075	0.007121745
122.0891277	204.4918448	62.44911062	6.718771055	2.261675998	0.00718536
468.3839816	265.0495156	396.3783827	8.924324171	-0.629906	0.00719359
0	1.755294805	3.469395034	-8.70511228	10.31306459	0.007194698
264.0046949	287.868348	485.7153048	8.031514989	0.722137639	0.007199465
345.3974466	234.3318565	246.3270474	8.242349736	-0.73313345	0.007200458
6.260980906	7.02117922	4.336743793	2.902485376	2.108159967	0.00720489
328.7014976	156.2212376	298.3679729	8.020140342	-0.86859186	0.007218748
536.3573643	426.5366376	330.459877	9.706779317	-0.95579905	0.007219969
54.26183452	113.2165149	1.734697517	6.46203641	-3.00183527	0.007224189
154.437529	249.2518623	147.449289	6.993129625	0.922750089	0.007232413
178.4379558	131.6471104	172.602403	7.668281672	-0.78815646	0.007248202
844.1889255	272.0706948	447.5519594	8.923826903	-0.79381422	0.007249987
0	1.158494571	0.867348759	0.907379231	-2.51408574	0.00725095
44.87036316	79.86591363	48.57153048	6.592263599	-1.22215064	0.007256301
57.39232497	29.84001169	0	6.19951537	-2.83238027	0.007291291
408.0072557	186.9388967	374.6946637	8.481978887	1.517826102	0.007291678
95.59474347	50.90354935	28.62250903	6.538826145	-1.02092386	0.007292851
20.86993635	426.5366376	366.0211761	4.255757056	5.877557737	0.007293832
371.4848671	595.0449389	641.8380813	8.588180375	0.727281186	0.007346674
307.8315612	345.7930766	503.06228	8.03326809	0.739854549	0.007350828
158.6115163	328.2401285	293.1638804	7.474668106	0.983850706	0.007392029
1355.502366	1360.353474	846.5323884	9.745995188	0.930257611	0.007394706
759.6656833	776.7179512	1062.502229	9.268604854	0.918652871	0.007396292
125.2196181	119.3600467	168.2656592	6.8812295	1.107171392	0.007399334
490.4435043	965.4121428	731.1750035	8.947864966	0.800701061	0.007421569
2.086993635	0	0	-1.05182722	-3.388203	0.007449333
31.30490453	26.32942208	16.47962641	5.766116739	-1.29486823	0.007524409
266.0708186	783.7391304	440.5958224	8.563384108	0.86635017	0.007529601
20.86993635	6.143531818	10.4081851	4.352791241	-1.40414982	0.007612682
60.52281542	80.74356103	56.37766931	5.348517552	0.941050408	0.007617455
0	10.53176883	23.41841648	2.479957394	4.024624677	0.007621712
2334.302381	1703.513608	1317.502764	11.31024544	-0.77708921	0.007633724
86.61023587	93.90827207	16.47962641	4.965204421	3.461888178	0.007696883
10.43496818	3.51058961	7.806138827	2.882202136	-1.60261328	0.007697416
72.00128042	51.78119675	65.05115689	6.64444787	-0.90740366	0.007738687

407.8820361	1039.924407	700.9132053	8.706420662	1.453121374	0.007776717
684.5339124	955.7580213	1043.420557	9.319190323	0.91582839	0.007779423
117.9151404	454.6213545	368.6232224	7.412513613	0.961236175	0.007828396
235.8302808	477.440187	654.8483127	8.201068076	1.006252715	0.007831019
2018.122845	582.7578753	1018.267443	10.5806795	-0.96780793	0.007844524
365.2238862	133.4024052	1712.146449	8.53042632	2.792537124	0.007851668
282.7876376	302.7883539	136.1737551	8.734379841	-0.96070927	0.007899122
43.82686634	0	271.4801614	-8.70511228	12.87779589	0.007920425
160.6985099	501.1366668	120.5614774	7.511532074	0.862610407	0.007921683
18.78294272	39.49413311	8.673487586	3.509379209	2.037282786	0.007927095
46.95735679	12.28706364	20.81637021	5.319399864	-1.15760498	0.007971756
11.47846499	0.877647403	0.867348759	3.581798596	-1.8293947	0.007988592
497.747982	1498.144116	962.757122	9.615507435	0.792527954	0.008018959
45.91385998	173.7741857	224.6433285	6.153923021	1.291924121	0.008019609
8.347974541	0.877647403	8.673487586	4.111933859	-2.10541576	0.008025383
306.8506742	664.3790837	440.6131694	8.480706348	0.793661342	0.008038078
24.00042681	6.143531818	14.7449289	5.30428962	-1.93062501	0.008049026
1706.910354	424.7550134	77.17669254	10.26487366	-1.62670254	0.008049122
0	356.3248454	230.7060963	2.198434169	8.150845855	0.00807206
0	24.85497444	3.469395034	6.61979467	-2.59216877	0.008105341
145.0460577	167.6306539	210.7657483	6.934659561	0.916684318	0.008126633
504.0089629	624.0073032	205.5616558	9.285607619	-1.29599003	0.008147213
313.0490453	277.3365792	190.8167269	4.157812489	5.25813055	0.008203011
1071.671232	3068.255319	989.6449335	10.38366969	0.885554796	0.008218339
30.26140771	0	27.75516027	4.127844529	-2.29400709	0.008238569
233.7432872	437.0684064	613.2155723	8.278688813	0.716761111	0.008250469
75.13177087	283.480111	98.87775848	6.347863379	1.373240064	0.008265575
312.0055485	344.9154292	319.1843432	7.824886725	0.753198128	0.008269195
1206.063187	1039.16963	828.5175546	9.42514135	0.81554339	0.008271688
1157.237971	1922.925459	1301.023138	10.64254413	-0.94761973	0.008278226
3.130490453	371.2448513	1.734697517	3.974915612	4.335997419	0.008284046
26.08742044	5.265884415	10.4081851	4.007642689	2.246894447	0.008284753
413.2247398	325.6071863	392.0416389	8.625909944	1.672202673	0.008302836
1433.764627	573.1037538	932.3999155	10.43377143	-0.92386234	0.00831564
26.08742044	28.08471688	70.25524944	4.353382447	1.497152031	0.008332776
9.391471359	14.04235844	23.41841648	2.722366083	2.185955353	0.008362702
521.7484088	358.0801402	398.1130802	9.208426741	-0.66737348	0.008378808

452.8776189	206.2471396	85.00017834	8.564243036	-1.05180904	0.008413513
16.69594908	13.16471104	20.81637021	3.480745137	1.158001412	0.008421016
193.6312695	76.82925362	42.03172084	5.68689868	1.997164975	0.008434894
6326.721205	2837.434052	3377.456066	11.42672038	1.144202987	0.008477349
2.086993635	0.877647403	0.867348759	3.649916007	-1.8305227	0.00848794
545.7488356	731.9579337	541.2256253	8.723013631	0.735760122	0.00851093
0	0.877647403	1.734697517	-8.70511228	7.76275099	0.008514248
185.7424335	165.8753591	160.4595203	6.887679183	1.040424051	0.008527444
126.2631149	210.6353766	143.1125452	6.969710877	0.834270481	0.008534988
1.043496818	0	0	0.215285569	-3.06736406	0.008539655
6.260980906	17.55294805	17.34697517	3.14144009	1.594869537	0.00858874
61.56631224	61.43531818	69.38790069	6.780910597	-0.86529497	0.008591448
4.173987271	135.1577	35.5612991	5.370587277	3.031170378	0.008618892
630.2720779	541.5084473	791.8894166	9.047061097	0.66493207	0.00862409
513.4004343	1289.264034	1116.277852	9.146439929	0.899952784	0.008640836
339.1364657	283.480111	672.1952879	8.045916302	1.161590527	0.008649383
2.086993635	0	0.867348759	0.022813157	-2.99443825	0.008675924
11.47846499	9.654121428	4.336743793	3.702886539	-1.80562723	0.00867957
357.9194085	157.9765325	386.8375463	8.68824593	-0.77218144	0.008699027
0	0	0.867348759	-8.70511228	7.994236316	0.008717043
199.3078922	72.84473441	306.1741118	7.832433051	-0.91463972	0.008721645
54.26183452	518.6896149	15.61227765	6.873863124	2.454762921	0.008736629
10.43496818	1.755294805	0	-8.70511228	8.791539225	0.008757466
141.9155672	114.9718097	110.1532923	7.232062489	-0.72769779	0.008763657
0	3.51058961	2.602046276	-8.70511228	10.28618265	0.008772142
789.8123063	324.1590681	504.467385	8.173281916	1.378926869	0.008778013
174.2639685	459.8872389	594.1338996	7.529737027	0.987743139	0.008799156
327.6580007	316.8307123	189.0820294	8.138840019	-0.69566277	0.008808778
132.5240958	1133.042797	340.8680621	7.830616235	1.353882087	0.008811563
57.39232497	74.60002921	113.6226874	5.953581588	0.95650094	0.008831754
235.8302808	588.9014071	356.4803398	7.734607352	0.900705928	0.008845898
475.8345489	330.8730707	230.7147698	9.523037096	-1.03184579	0.008850654
10352.53193	4791.07717	3705.313897	12.73872719	-0.95788579	0.008883485
72.00128042	71.0894396	52.04092551	6.555796582	-0.72173006	0.008883753
1.043496818	0	0.867348759	1.174132746	-2.36437846	0.008932447
4.173987271	7.02117922	13.01023138	2.937025738	-2.04718543	0.00894203
74.08827405	86.00944545	111.8879899	6.678048561	-0.86697259	0.008944164

1.043496818	0.877647403	2.602046276	3.188916499	-2.31348566	0.008944887
3.130490453	0.877647403	0	1.937169739	-2.84856567	0.008974604
228.5258031	164.1200643	257.6025813	7.882475394	-0.50974974	0.008981211
1.043496818	56.16943376	14.7449289	5.213368069	3.012718271	0.009001785
452.8776189	20.18589026	162.1942179	8.30740892	-1.57179156	0.009012929
3849.45976	1428.809971	3166.690318	10.58727004	1.15598097	0.009050958
0	0	0	-8.70511228	-7.47619731	0.009054072
0	0	0	-8.70511228	-7.47619731	0.009054072
1.043496818	0	0.867348759	0.592267839	-2.59163158	0.009057687
0	1.755294805	0	1.25983321	-2.75890712	0.009058779
126.2631149	212.3906714	444.0825644	7.553658693	1.309506549	0.009064268
178.4379558	369.4895565	609.7461773	8.505719958	1.814800089	0.009066061
89.74072632	14.04235844	36.42864786	6.294115865	-1.21444	0.009074137
216.0038413	427.414285	703.4198432	7.642285337	1.169071856	0.009076788
67.82729315	57.04708116	61.58176186	5.769150717	1.065029403	0.009088614
62.60980906	187.8165441	139.6431501	6.368718633	0.941529381	0.009088715
4.173987271	19.30824286	4.336743793	2.774329434	1.690990759	0.009094732
31.30490453	36.86119091	61.58176186	6.107699758	-1.19591039	0.009099372
109.5671659	121.9929889	188.2146806	6.429051446	0.878903991	0.009105314
270.2656758	389.6754467	173.4697517	8.291254344	-0.62980852	0.009150595
443.4861475	374.7554409	264.5413714	9.07006268	-0.74856801	0.009161659
2.086993635	17.55294805	19.08167269	2.967619149	1.845186684	0.00916388
49.04435043	87.76474025	42.50008917	5.823031342	1.741547863	0.00917606
880.7113141	66.70120259	208.1637021	8.625919007	-1.22441257	0.009189611
139.8285736	92.15297726	165.6636129	6.016939317	2.372635676	0.009228314
612.532632	6881.633283	4236.131337	10.00398575	2.035119139	0.009229933
1299.195278	373.2722168	363.8701512	8.817027507	1.298848955	0.009240467
0	0	0	-8.70511228	-7.51461966	0.009243368
0	0	0	-8.70511228	-7.51461966	0.009243368
218.0908349	58.80237597	76.32669075	5.831591763	1.243919242	0.009244321
0	0	0	-8.70511228	-7.52495396	0.009293088
28.17441408	4.388237013	3.469395034	3.854447418	-1.59305723	0.009326514
318.2665294	408.9836896	333.0619233	7.980215498	0.913112745	0.009348265
14.60895545	443.2119383	215.1024921	6.679274482	2.229250085	0.00935707
1867.661039	2372.7022	2286.903778	10.76804911	0.782804851	0.009373534
150.2635417	51.78119675	2551.740048	6.227721395	5.347551755	0.009386095
0	0.877647403	1.734697517	-8.70511228	7.743397827	0.009390838

28.17441408	50.90354935	21.68371896	4.427511434	1.474287585	0.009393407
5235.223534	817.0897317	6178.992556	12.84813686	-1.50724166	0.009393563
1.043496818	0	0.867348759	-8.70511228	7.640432708	0.009393624
26.08742044	218.5342032	48.57153048	6.18932312	2.325104955	0.009401828
1443.156099	1543.781781	2260.310865	10.07447449	0.822018658	0.009408457
8969.898645	6896.553289	2449.392894	12.92064357	-0.98089364	0.009422413
20.86993635	13.16471104	19.94902145	3.72867041	2.196687981	0.009458293
0	11.40941623	8.673487586	2.291656561	3.777992704	0.009509535
0	0	0	-0.38580073	-2.6911227	0.009517279
129.3936054	13.16471104	121.4288262	6.804590818	-1.45475721	0.009542661
10.43496818	1.755294805	2.602046276	2.75848596	-1.83807321	0.00954858
7.304477724	21.94118506	9.540836344	2.205571939	2.584848901	0.009554772
0	0	0	-8.70511228	-7.58360699	0.009584766
0	0	0	-8.70511228	-7.58360699	0.009584766
212.8733508	638.0496616	486.5826536	7.953540141	1.107406714	0.00959016
0	232.5765617	499.5928849	3.668700223	6.98429459	0.009593745
243.1347585	100.0518039	123.1635237	7.999653069	-0.79643295	0.009596019
443.4861475	611.7202395	472.7050734	8.624787786	0.674418713	0.009598997
0	0	0	-8.70511228	-7.58686279	0.009601764
4.173987271	57.92472857	10.4081851	4.194943973	2.652432652	0.009631037
0	0	0	-0.65891312	-3.21837953	0.009633538
0	7.02117922	1.734697517	-8.70511228	11.7652105	0.009655646
64.6968027	78.11061882	91.07161965	6.403719336	2.267802007	0.009671236
1.043496818	34.2282487	17.34697517	1.475546252	4.794969915	0.009674931
290.0921153	174.6518331	176.9391467	7.943106696	-0.81730371	0.009686267
0	0.877647403	0.867348759	1.483980469	-2.49305572	0.009690226
3590.67255	1669.28536	1114.543155	11.6447921	-0.91022667	0.009704313
103.3061849	280.8471688	186.4799831	7.777481506	0.958749854	0.009725099
270.2656758	566.0825746	373.8273149	8.259530289	0.699122376	0.009734467
10.43496818	53.53649155	10.4081851	3.940752866	1.370888092	0.009743785
325.5710071	521.3225571	443.2152156	7.861280617	1.378729222	0.009746141
675.142441	1133.920444	1115.410504	9.389712649	0.814676767	0.009754361
0	19.30824286	862.144666	2.461447534	8.437583935	0.009776168
222.2648222	371.2448513	326.1231332	8.136343111	0.839064382	0.00981079
98.08870086	188.6941915	105.8165485	8.042771843	-0.94192397	0.009828815
271.3091726	288.7459954	261.9393251	7.758378265	0.790357994	0.00983331
1.043496818	0.877647403	0.867348759	-8.70511228	7.663633164	0.009850876

105.8105773	213.0576834	397.0982821	6.94322461	1.071716185	0.009861681
63.65330588	58.80237597	4.336743793	7.185352332	-1.76866619	0.009870552
14.60895545	0.877647403	0.867348759	3.217143044	-1.95900032	0.009886513
1.043496818	0.877647403	5.204092551	3.816021703	-1.65661351	0.009887013
13.56545863	8.776474025	31.22455531	3.514342105	2.259598816	0.009897724
505.0524597	552.0402162	377.29671	9.139800971	-0.49264056	0.009903946
0	0	0	-0.55016167	-3.37736486	0.009906959
0	7.02117922	1.734697517	1.035652604	3.713066712	0.009914478
41.73987271	13.16471104	25.153114	4.763417453	1.931602707	0.009920554
27.13091726	312.4424753	18.21432393	5.174331877	3.212926316	0.009923225
144.0025608	82.49885584	155.2554278	7.58804709	-0.78744255	0.009927646
0	0	0.867348759	1.369336073	-1.95619013	0.009960299
1417.068678	2720.706948	941.9407518	11.09779632	-0.95151485	0.009979409
10.43496818	14.04235844	2.602046276	3.063309041	2.094062254	0.009989646
1191.673366	695.0967428	810.9710893	10.12890233	-0.65240296	0.009995808
343.310453	66.70120259	137.0411039	7.335053244	-2.55244999	0.010016117
1.043496818	0	0.867348759	-0.41914544	-2.71379269	0.010019315
0	0	0	-1.05703921	-3.05342873	0.010036138
132.5240958	108.8282779	33.82660158	7.056457323	-1.17017388	0.010041363
617.7501161	2282.760894	1163.114685	9.725370437	0.911274086	0.010060771
102.2626881	213.2683188	193.4187732	6.579021357	0.905064715	0.010063297
274.5440127	214.2688368	471.612214	8.197319206	0.973121145	0.010067466
8.347974541	8.776474025	13.01023138	3.508258347	2.096140569	0.010093155
5.217484088	0	6.938790069	2.73666292	-1.46267537	0.010102327
352.7540992	38.78323872	342.7241885	8.882732573	-1.1330386	0.010105095
5.217484088	0.877647403	0	2.70552717	-1.85649643	0.010111473
12203.69528	8276.215006	2219.545473	11.27714732	1.499398711	0.010119062
0	0	0	0.35382457	-2.99935502	0.010145518
6678.379633	3238.518915	1956.738799	12.64015925	-0.97310026	0.010168778
699.1428678	2481.986854	954.9509832	10.36212284	1.917102581	0.010176422
1.043496818	0.877647403	2.602046276	-8.70511228	8.676350062	0.010200974
0	7.898826623	0.867348759	0.98964637	3.676616757	0.010203192
0	0.877647403	0.867348759	0.979748541	-2.35456774	0.010211422
44.87036316	172.8965383	119.6941287	5.45429237	1.365702147	0.010228836
1384.720277	1700.880666	2204.800544	10.1521952	0.859311679	0.010230389
8321.887121	6650.812016	4446.897085	12.01655468	1.198735807	0.010235607
5.217484088	88.64238765	24.28576524	4.691013305	2.540801821	0.010248553

1.043496818	0	0	0.68800924	-2.63024666	0.010248563
202.4383826	581.8802279	734.6443985	8.386921038	1.606828077	0.010261493
0	0	0.867348759	-0.85319928	-2.59824902	0.010263461
793.3497605	710.894396	996.5837236	10.08612974	1.093743995	0.010272316
19.82643954	41.24942792	27.75516027	4.673091258	1.258125209	0.010315163
889.0592886	2036.141974	1561.227765	10.32939516	1.279092323	0.010324642
192.0034145	243.9859779	230.7147698	6.819449029	0.857524884	0.010325132
20.86993635	8.776474025	17.34697517	5.102832012	-1.2049836	0.010326697
24908.26904	14452.21978	24303.11222	15.07931094	-1.11312497	0.010332725
28.17441408	0	0	-8.70511228	10.9134884	0.01034395
658.4464919	1514.819417	613.2155723	9.509994244	0.932304126	0.010358229
400.702778	550.2849214	562.0419956	8.549115482	0.524327812	0.010388415
182.6119431	256.2730415	368.6232224	7.651260883	0.689341687	0.010388855
131.480599	229.9436195	272.3475102	7.690818079	0.779561935	0.010398627
285.918128	473.9295974	238.5209086	7.919820738	0.764445994	0.010400421
3.130490453	21.06353766	1.734697517	1.323611782	2.354852831	0.010442683
1.043496818	0	10.4081851	0.142360454	4.038698425	0.010462492
30.26140771	45.63766493	61.58176186	4.579506807	1.256534443	0.010528248
546.7923325	128.1365208	345.2048059	7.616322689	1.388551776	0.01056277
198.2643954	129.8918156	100.612456	7.715797286	-0.72207712	0.010570282
6.271415874	55.29178636	4.336743793	2.779256953	2.688681517	0.01057062
0	1.755294805	0	0.657381323	-2.59598264	0.010589794
146.0895545	285.2354058	139.6431501	6.926294772	1.583436785	0.010591038
30.26140771	39.49413311	67.65320317	5.752305892	1.679772158	0.01059354
286.9616249	431.7498632	666.9911953	8.493254572	0.799529141	0.01059685
37.56588544	171.1412435	443.2152156	5.970932914	1.856187438	0.010605549
104.6731658	111.9790321	133.3982391	6.631767368	0.73637785	0.010607862
7.304477724	67.57884999	78.06138827	4.176309312	3.334599175	0.010617525
206.6123699	84.25415064	98.01040972	7.744017128	-0.81352722	0.010655454
159.6550131	200.1036078	162.1942179	7.503141634	0.8838187	0.010667744
92.87121677	117.6047519	137.0411039	6.542592478	0.891703706	0.010682656
569.7492624	172.0188909	552.5011592	8.649912476	-0.9122383	0.010698491
2.086993635	10.53176883	19.94902145	2.486406447	1.839388341	0.01070883
29.21791089	36.86119091	39.03069414	5.095901187	1.298603448	0.010709328
207.6558667	227.3106772	231.5821185	7.30964522	0.722739859	0.010725953
1067.38246	0	14.7449289	9.323756464	-3.21014449	0.010732378
285.918128	424.7813428	531.684789	8.310565156	0.661936376	0.010800868

26.08742044	221.1671454	31.22455531	5.883504911	1.745252898	0.010862485
1.043496818	2.632942208	2.602046276	4.700241198	-2.19649898	0.010909272
43.82686634	95.66356687	46.83683296	5.556560017	1.242513722	0.010953288
757.5786896	1808.831297	470.1030271	10.64357571	-1.20460418	0.010978572
0	4.388237013	0	-8.70511228	8.826328738	0.011006801
588.5322052	1021.581577	749.3893274	9.405060848	0.594550714	0.011023224
274.5857526	400.1896626	318.3430149	8.160926929	0.506752474	0.011025421
53.60443152	21.94118506	61.43431257	5.072826166	1.412385736	0.01103414
123.1326245	126.381226	71.98994696	7.998092689	-1.31761101	0.011044155
0	0	0.867348759	-8.70511228	8.858541508	0.011068213
10.43496818	3.51058961	8.673487586	2.260332823	1.595687091	0.011084073
64.6968027	331.7507181	563.7766931	4.87141201	5.643121917	0.011095867
666.7944665	1529.739423	913.3182428	9.398261194	0.692891472	0.011098638
198.2643954	129.0141682	57.24501807	7.317353751	-0.84130987	0.011128438
0	3.159530649	8.378589008	-8.70511228	9.629398921	0.011160791
2014.992355	705.6285116	1171.788173	10.89084684	-0.65857194	0.011179419
0	0.877647403	0	-8.70511228	8.035368288	0.011185271
290.0921153	360.7130824	379.8987563	7.999675761	0.605747817	0.011186726
113.6368034	140.9501728	94.75785187	6.902743534	0.810929046	0.01119088
442.4426507	1011.049808	751.1240249	8.922825011	0.796408954	0.011195197
2.086993635	38.61648571	23.41841648	2.673058068	2.971684126	0.011218404
4.173987271	1.755294805	8.673487586	3.355168344	-1.72072477	0.011227761
0	0	0	-0.72293745	-2.98394656	0.011228423
0	4.388237013	0	-8.70511228	9.027407707	0.011299286
226.4388094	232.5765617	368.6232224	7.804742495	0.776518785	0.01131774
634.4460651	688.0755636	910.7161965	9.112521374	0.685995569	0.01132747
1.043496818	68.4564974	4.336743793	3.4010211	3.156798489	0.011336596
709.577836	318.5860071	405.0518703	9.306499018	-0.89728633	0.011338746
0	0	0	0.018620586	-2.97959063	0.011344253
120.002134	151.8330006	125.76557	6.668078236	0.660417613	0.011350724
0	0	0	-2.37598161	-5.61284275	0.011353083
4.173987271	4.388237013	6.07144131	3.35139124	-1.48656811	0.011366678
112.6976563	164.1200643	163.0615666	7.027682263	0.786396933	0.011406691
31.23185975	9.654121428	19.89698052	4.698877537	-1.08111771	0.011427705
294.2661026	742.4897025	978.3693997	8.672984032	1.371072984	0.011428931
1381.589787	4405.789961	2583.831952	10.9503354	0.863802887	0.011432655
9.391471359	9.654121428	16.47962641	2.564888527	2.195615605	0.011448808

112.6976563	146.5671162	140.5104989	6.35003011	1.306873374	0.011471826
0	0	0	-8.70511228	-8.2990814	0.011479887
0	30.46314134	1.734697517	1.43941999	3.497959504	0.011481566
165.915994	192.2047811	546.4297179	7.513285158	1.006902706	0.011486962
172.1769749	119.3600467	153.5207303	7.390281281	-0.69965676	0.011498548
8.347974541	0	1.734697517	4.5000636	-1.67926864	0.011512779
213.9168476	115.8494571	326.1231332	7.579599236	0.979391468	0.011516445
1.116541595	0	0.919389684	-0.02522998	-2.30112966	0.011516498
105.0279547	68.63202688	175.7075115	6.664517473	0.885055759	0.011594858
1.043496818	0	0.867348759	2.804266138	-2.96778034	0.01160721
2977.096421	1817.607771	7721.138649	11.20910723	1.434651821	0.011619682
16.69594908	4.388237013	6.938790069	5.365308955	-1.64226697	0.011643327
1738.298739	1574.52577	899.6228059	10.98989114	-0.65739397	0.011643907
157.5680195	163.2424169	83.26548082	6.274531132	0.891879833	0.011681801
9.391471359	14.04235844	13.20972159	4.530798356	-1.45134354	0.011705093
109.5671659	8.776474025	35.5612991	5.594006026	-1.48737214	0.011720035
133.5675927	58.80237597	109.2859436	7.691403575	-0.98522987	0.011721382
157.5680195	300.1554117	197.755517	7.45329163	0.639289673	0.01175
450.7906252	569.5931642	908.1141502	9.74276355	-1.03540556	0.011791812
251.4827331	478.3178344	1281.074116	8.632101244	0.959994619	0.011801631
402.8628164	315.8126413	255.6597201	9.655183617	-1.24262164	0.011820974
1.043496818	0.877647403	3.469395034	-8.70511228	9.25633684	0.011822091
120.002134	163.2424169	130.1023138	7.29608087	1.444289272	0.011832638
0	0	0	0.068049176	-2.70545212	0.011834005
474.791052	254.9565704	236.7862111	8.459747619	-0.88068155	0.011842184
31.30490453	30.71765909	19.94902145	4.447596616	1.280243776	0.011843033
19.82643954	57.92472857	50.306228	6.454482419	-0.94483823	0.011910623
67.82729315	104.4400409	38.16334538	5.679230011	0.953446771	0.011912566
284.8746312	491.4825454	582.8583658	8.283245777	0.9033246	0.01191784
286.9616249	337.0166026	4725.316037	8.485296005	2.766099327	0.011931228
20.86993635	8.776474025	11.27553386	4.307473213	-1.11368735	0.011978939
147.1330513	5.265884415	213.3677946	5.820292638	3.585803155	0.011991383
1519.331367	647.7037831	684.3381705	10.13570537	-0.72452517	0.012001149
103.3061849	71.96708701	126.6329188	6.375310267	0.787734128	0.012011588
4.173987271	11.40941623	13.01023138	2.684264579	1.505684245	0.0120154
20.86993635	27.20706948	37.29599662	4.497097965	1.06878772	0.012017264
457.0516061	292.256585	143.9798939	8.289406456	1.137935583	0.012017431

99.13219768	161.4871221	117.9594312	6.125419546	0.982123706	0.012018928
653.2290079	678.4214421	596.7359459	9.459187649	-0.55865276	0.012022301
17.7394459	9.654121428	5.204092551	4.004777772	-1.34883199	0.012023514
9.391471359	2.632942208	4.336743793	3.632016104	-1.61670376	0.012028681
150.2635417	110.5835727	569.8481344	7.339983611	1.156094499	0.012059404
0	0	0	-0.81121483	-3.00872065	0.012094017
0	0.877647403	0.867348759	-8.70511228	7.579001656	0.012096609
13.56545863	0	4.336743793	3.259359075	-1.89506937	0.012099594
1.043496818	5.265884415	2.602046276	0.454075633	2.605475284	0.012107824
1008.017926	701.2402746	854.3385272	10.34331329	-0.71095963	0.012114204
291.1356121	727.5696967	489.1846998	8.223347691	0.87479622	0.01213697
1716.552265	1352.454647	1710.411752	10.55142006	0.666801787	0.012145273
169.0464845	444.0895857	257.6025813	8.009043136	0.659672678	0.012148392
258.7872108	434.4354642	387.7048951	8.116357282	0.787017981	0.012155741
0	21.94118506	21.68371896	2.058061944	7.757885392	0.012198021
4707.214144	2523.236282	2302.810954	11.90596601	-0.60768571	0.012204082
2.086993635	22.81883247	1.734697517	3.069512579	2.507439057	0.012216207
486.269517	451.9884123	686.072868	9.711791016	-0.87662973	0.012220517
210.7863572	5.265884415	274.0822077	5.877369502	4.281126255	0.012222892
327.6580007	757.4097084	522.1439527	8.618654582	0.726289021	0.012258631
1088.471531	42.12707532	12.14288262	9.593868132	-3.05542764	0.012334101
0	28.08471688	30.35720655	2.114249576	3.244503418	0.01235105
78.26226132	169.3859487	93.67366593	6.279938323	0.879086969	0.012358891
1170.803429	1748.273626	2675.77092	10.42954164	0.740698258	0.012372326
426.7901984	817.9673791	375.5620125	8.509360004	0.713575779	0.012379295
9.391471359	59.68002337	57.24501807	4.105196286	1.476623586	0.012380878
2348.911337	956.6356687	778.0118364	10.7166199	-0.7014344	0.012390033
1398.139646	4086.931883	4301.026371	11.39953617	1.417054044	0.012414144
5653.665758	14264.40323	5649.909813	12.32094046	1.068186979	0.012421798
85.56673905	20.18589026	212.5004458	6.274622444	1.370535058	0.012426921
88.6972295	43.88237013	56.37766931	5.205546494	1.206482434	0.012428254
1852.394681	2302.411419	2001.806241	10.58039935	0.625340744	0.012428258
1498.200556	1363.916722	2627.650411	10.07417879	0.785136336	0.012431179
38.60938225	262.4165733	101.4798048	5.963228039	1.329026925	0.012444614
1576.483687	387.218034	381.5293719	8.954997865	1.315083075	0.012448527
173.2204717	401.9625103	197.755517	7.743967827	0.682102435	0.01249586
320.353523	796.9038415	462.2968883	8.587312034	0.683959425	0.012510894

1.043496818	1.755294805	0	3.073449766	-2.48874525	0.012515522
13.56545863	10.53176883	4.336743793	3.432303497	1.522231757	0.012519829
244.1782553	92.15297726	63.31645938	8.215702783	-1.05110143	0.012529743
93.91471359	59.68002337	91.97366236	6.371488923	-1.06835946	0.012544837
52.17484088	1.755294805	42.50008917	5.202265548	-1.59069463	0.01256215
787.8400973	683.6873266	888.1651288	9.408427005	0.869372275	0.012599554
25.04392362	78.11061882	40.76539165	4.976287738	1.272975881	0.01260337
560.3577911	1079.506305	822.2466231	9.3422255	0.653825378	0.012611975
5350.008184	2089.678465	2301.076256	11.69875877	-0.70763361	0.012620727
0	0	0	2.991428359	-2.97835646	0.012636925
94.95821041	308.9318857	289.6944854	7.092462506	0.935105855	0.012643391
77.21876451	114.9718097	82.39813206	6.477296273	0.629504192	0.01264409
34.43539498	63.19061298	124.0308725	5.787747919	1.226662712	0.012646324
47827.52879	4577.940499	17749.19081	13.16402076	1.625349647	0.012654842
0	960.1462583	407.6539165	6.511393804	4.452270666	0.012661507
108.523669	111.4612201	151.7860327	6.745856509	0.727876854	0.012696045
443.4861475	417.7601636	477.0418172	8.03084304	0.888451675	0.012700295
0	20.18589026	0.867348759	4.246852641	7.041751735	0.01272319
8.347974541	4.388237013	10.4081851	3.969920679	-1.55297869	0.012732932
190.9599176	275.5812844	130.9696625	7.322697638	0.673763115	0.012737505
4209.466162	1903.617216	243.7250012	12.57448696	-1.34352392	0.012773656
68.87078997	67.57884999	29.48985779	6.624553729	-0.86894927	0.012793981
5.217484088	15.79765325	13.01023138	4.140169604	2.996528148	0.012840968
303.6575739	97.41886168	27.75516027	7.671467838	-1.03074916	0.012854593
169.0464845	239.5977409	417.1947529	7.488371395	0.947503382	0.01285841
6.260980906	1.755294805	17.34697517	1.52246714	2.188683872	0.01289668
110.6106627	133.4024052	149.1839865	6.799488122	0.885106739	0.01293198
204.5253763	342.282487	343.4701084	7.944930077	0.958969698	0.012942945
1.043496818	0	0	4.390241031	-2.91071463	0.012944876
90.78422314	190.4494863	205.5616558	6.931528052	0.715605732	0.012964164
3116.924994	90.39768246	7.806138827	13.75063182	-2.6919264	0.0129656
35.4788918	119.3600467	90.20427089	6.080529405	1.523555889	0.012993444
109.5671659	185.1836019	114.4900361	6.966644739	0.564075716	0.013009314
9.391471359	12.28706364	8.673487586	2.640934924	2.070210156	0.013019024
30.57445676	81.80551439	143.1906066	5.747488996	2.242785491	0.013035012
2794.484478	1221.685184	1885.616201	11.49399046	-1.16723827	0.013047957
351.6584276	591.5343493	716.4300746	8.539679059	0.762798362	0.013072153

0	0	0	0.591750434	-3.01326057	0.013148553
21.91343317	49.14825454	47.70418172	4.999239828	1.027445101	0.013160728
111.6541595	42.12707532	71.1225982	6.508688406	-1.11178073	0.013163567
57.39232497	9.654121428	37.29599662	5.995911452	-1.09279136	0.013196121
364.1803894	977.6992064	1053.828742	8.589303307	1.016865809	0.013218435
10.43496818	50.90354935	19.94902145	3.98807178	1.692333226	0.013221706
137.7415799	82.49885584	92.80631717	7.00096751	-0.76332853	0.013222958
114.7846499	147.4447636	814.4404843	7.509089739	1.761329755	0.013225892
18.78294272	37.73883831	52.04092551	5.063060881	1.204095727	0.013246552
43.34685781	69.30781538	207.9121709	5.003983928	1.982668399	0.013256116
0	0	0	-0.72978769	-2.89616369	0.013258438
6.260980906	6.38927309	6.565830102	2.620366052	1.81683204	0.013268977
744.013231	1681.572423	470.9703759	10.10122779	-1.04528518	0.013281708
2.086993635	0	1.734697517	1.938817504	-2.25547224	0.013303797
0.521748409	0	0	0.940619928	-2.97296599	0.01330547
22.74823063	19.74706656	14.69288797	3.627956517	1.729326538	0.013317065
16.69594908	29.84001169	47.70418172	4.039407344	1.14446054	0.013319157
0	0	0.867348759	-8.70511228	12.13514598	0.013320051
27.13091726	61.43531818	71.1225982	5.057550167	0.948340939	0.013325322
0	2.632942208	3.469395034	-8.70511228	7.864863921	0.013336807
169.0464845	250.1295097	179.541193	8.031748888	1.281717039	0.013342133
83.47974541	83.37650324	145.7145914	6.472598321	0.847044875	0.013423875
24.00042681	9.654121428	16.47962641	3.141956379	3.332370613	0.013445171
4655.039304	1915.90428	2045.208373	12.02318742	-0.68112301	0.013445379
2.086993635	309.8095331	2.602046276	5.910516331	2.797229302	0.013449433
238.9607712	763.5532402	965.3591683	7.746271808	1.660877828	0.013451875
196.1774017	14.92000584	26.88781152	8.505511287	-2.15476356	0.013461749
0	6.143531818	13.03625184	2.100473928	2.238068657	0.013468248
90.78422314	153.5882954	111.8879899	6.593148942	0.748715764	0.01348154
10.43496818	25.45177467	18.21432393	3.519279975	1.570262428	0.013501342
6466.549779	1488.489995	1950.667358	13.34695205	-1.60709046	0.013511366
155.4810258	384.4095623	431.9396818	7.300904811	1.003332254	0.013551282
275.4831599	609.9649447	538.6235791	8.487230768	0.717400521	0.013568962
469.5735679	275.5812844	298.3679729	8.777640423	-0.71653538	0.013577285
27.13091726	18.43059545	29.48985779	4.644194757	0.981420284	0.013603591
530.0963834	334.3836604	725.9709109	8.581215249	0.840224335	0.013611727
1.043496818	10.53176883	3.469395034	2.316515998	2.310133901	0.013631489

303.6575739	242.2306831	392.9089876	8.214557102	-0.59848972	0.013653156
0	0	0	-1.32093422	-2.93423917	0.013660385
0	0	0.867348759	-1.34633807	-3.05587297	0.013660385
0	0	0	-1.38823275	-3.11206251	0.013660385
0	0	0	-1.4031268	-3.23357716	0.013660385
238.9607712	256.2730415	258.4699301	8.216834552	-0.51634929	0.013665822
181.5684463	71.0894396	46.83683296	7.034321958	-0.83892525	0.01366901
1.043496818	2.632942208	1.734697517	-8.70511228	7.722617956	0.013681615
0	6.143531818	1.734697517	-0.23271193	3.288553664	0.01368245
116.8716436	249.2518623	136.1737551	6.858784925	0.67303641	0.013706334
139.8285736	127.2588734	174.3371005	6.859119365	0.625460886	0.013733267
111.6541595	170.2635961	128.3676163	7.380751305	-0.79301487	0.013748829
43.82686634	56.16943376	22.55106772	5.884566258	-0.76336628	0.013754255
28.17441408	3.51058961	26.02046276	5.954871186	-1.52369929	0.013761946
88.6972295	25.45177467	50.03734988	6.550116941	-1.88742653	0.013762279
0	39.49413311	291.4291829	-8.70511228	13.7740014	0.013763589
180.5249495	482.7060714	416.3274041	7.890982617	0.84681364	0.013771498
74.08827405	23.69647987	111.8879899	5.393854145	1.929552238	0.013784067
26.08742044	40.37178052	37.29599662	4.656109676	1.139715065	0.013828161
164.8724972	452.8660597	397.2457314	7.431821305	1.128341178	0.01385955
0	0	0	-0.64567739	-2.7756147	0.013860592
590.6191988	337.89425	363.4191298	10.19160017	-1.18684952	0.013871073
2.086993635	7.898826623	24.28576524	3.786328891	-1.69239007	0.013875335
420.5292175	545.8966844	245.4596987	9.152966865	-0.77615643	0.013885434
366.267383	210.6353766	202.9596095	8.405660799	-0.75646052	0.01389633
2.086993635	0	0.867348759	1.838809605	-2.01527068	0.013921661
77.21876451	150.9553532	100.612456	6.319159161	0.741984243	0.013937208
1357.58936	2009.812552	2760.771099	10.42891079	0.912749677	0.013952474
0	0	0.867348759	4.260759199	-2.86395183	0.01395875
49.04435043	114.9718097	95.40836344	5.996750073	0.78747363	0.013965198
0	7.02117922	5.204092551	1.006903661	2.71835977	0.013994925
2490.826904	2814.61522	1139.696269	11.78546635	-0.92274473	0.013998699
0	0	0	-8.70511228	-7.9210453	0.014031007
368.3543766	296.644822	422.3988454	9.279351611	-0.67655847	0.014047433
301.5705803	724.9367545	1110.206411	8.671364482	0.892470685	0.014068154
949.5821041	3009.452943	1523.06442	9.905019071	0.989440752	0.014074418
41.73987271	38.61648571	89.33692213	4.665590331	1.150961406	0.014074698

99.13219768	184.3059545	189.9493781	6.774806535	0.761214465	0.01407699
116.8716436	201.8589026	133.5717088	6.579005894	0.863972827	0.014081024
46.95735679	15.79765325	22.55106772	5.359626441	-1.26406428	0.014081332
12.52196181	0.877647403	6.938790069	3.657566962	-1.72776348	0.01410905
2.086993635	0	0.867348759	1.293582003	-2.04507554	0.014137965
162.7855036	248.3742149	384.2355	7.857158001	0.726590369	0.01417295
328.7014976	676.6661473	449.2866569	8.668615453	0.656759191	0.014181892
1205.238824	604.6990603	874.2875486	9.891526614	-0.68063543	0.014208082
1.043496818	6.143531818	0	0.291488554	4.224738611	0.014210451
0	0	0	-8.70511228	-7.97715331	0.014211591
0	0	0	-8.70511228	-7.97715331	0.014211591
7043.603519	2725.972832	3387.864251	12.37046975	-1.25945235	0.014219864
0	23.64382102	0	-8.70511228	11.59032954	0.014231127
2.086993635	0	0	-0.83696156	-2.99997072	0.014272139
214.9603444	305.4212961	292.2965316	8.32465313	-0.57390137	0.014279475
2.086993635	1.755294805	16.47962641	0.517368567	4.676498637	0.014284193
225.3953126	452.8660597	221.1739334	7.716780073	0.623969464	0.014308973
533.2268738	426.5366376	336.5313183	8.760672256	-0.83597931	0.014324983
224.3518158	71.0894396	184.7452856	8.07227365	-0.80631838	0.014355468
0	0	0	0.528977364	-2.49172524	0.014361716
305.7445676	587.1461123	437.1437743	7.5769736	1.30034915	0.014364127
0	3.51058961	0	-8.70511228	8.259081656	0.014392089
39.65287907	93.03062467	121.4288262	6.478331023	1.316214535	0.01440492
131.480599	137.7906422	148.3166377	6.558983412	1.518606866	0.014409002
0	14.92000584	7.806138827	0.986748828	5.910166292	0.014417484
0	0	0	-8.70511228	-8.04068962	0.014417638
327.6580007	215.901261	314.8475994	8.853049663	-1.00970319	0.014435597
2.086993635	0	0	0.071066114	-2.8681529	0.014438098
46.76952737	98.03321486	183.5830382	6.192883632	1.111034887	0.014439383
6217.060125	2379.302108	9471.405076	13.57949056	-1.52926842	0.014442667
135.6545863	179.9177175	113.6226874	6.969440623	0.703705165	0.014461132
0	0	0	-8.70511228	-6.9046645	0.014463838
0	0	0	-8.70511228	-6.87609535	0.014463838
0	0	0	-8.70511228	-6.87609535	0.014463838
0	224.677735	12.14288262	1.842372864	7.326507231	0.014472856
768.0136578	1279.609913	804.0322992	9.059822699	1.107638274	0.014481214
140.8720704	317.7083597	144.8472427	7.00379788	0.795262068	0.014483837

243.1347585	272.9483422	388.5722438	7.763269035	0.846488235	0.01449487
229.5692999	118.4823993	104.081851	7.887657577	-0.80552415	0.01452142
5.217484088	240.4753883	62.44911062	4.740509333	2.669882732	0.01462558
0	0	0	-0.77811032	-3.00817418	0.014629144
286.9616249	895.2003506	592.3992021	8.645458855	0.948842984	0.014633589
1.043496818	3.51058961	20.81637021	1.522902623	3.591965436	0.014635187
10340.00997	4812.140708	7078.433219	13.07358248	-0.73891975	0.014642938
24.00042681	0	6.07144131	3.016111225	-1.99404282	0.01464677
1256.370168	923.2850674	881.2263387	10.00838865	-0.61569221	0.014650891
0	0	0	-8.70511228	-6.90530955	0.014682819
0	0	0	-8.70511228	-6.90530955	0.014682819
0	0	0	-8.70511228	-6.90530955	0.014682819
0	0	0	-8.70511228	-6.90530955	0.014682819
0	0	0	-1.35410157	-3.27884766	0.014687872
19.82643954	24.57412727	69.38790069	4.82442226	1.404286915	0.014707212
11.7393392	7.679414772	1.734697517	2.407153435	1.736674187	0.01471224
1.043496818	14.04235844	2.602046276	1.775277605	3.247879227	0.014714112
1762.643519	456.8769083	553.8368763	9.130617518	1.205981038	0.01473533
44.87036316	97.41886168	141.3778476	6.214331834	1.096439505	0.014759062
6.260980906	4.388237013	6.07144131	3.077571026	-1.41300455	0.014761806
63.65330588	50.90354935	45.10213545	5.992464607	-0.64847589	0.014767684
3093.968064	1811.464239	1228.165842	11.86443062	-1.28058596	0.014768888
3.130490453	0.877647403	4.336743793	3.114571797	-1.52651894	0.014779859
3.130490453	3.51058961	1.734697517	3.365618898	-1.64182935	0.014816149
60.1367216	156.1422494	34.84139963	8.001805661	-1.7015482	0.014820993
1.043496818	2.632942208	488.3173511	2.914664935	5.570379961	0.014841198
585.4017147	177.2847753	501.3275825	9.797615157	-0.99376378	0.014852576
1520.374863	2074.75846	802.2976017	11.06637301	-0.7768874	0.014874925
9.391471359	22.81883247	14.7449289	3.710018701	1.105650583	0.014877371
6.260980906	437.9460539	26.02046276	6.578107567	2.511212451	0.014932344
187.8294272	97.41886168	135.3064063	7.285698828	-0.60194821	0.014935941
0	0	0	-8.70511228	-6.93904833	0.014937649
0	0	0	-8.70511228	-6.93904833	0.014937649
1340.893411	1981.727835	1386.890665	10.01053825	0.927465658	0.014953215
934.9731486	563.4496324	940.2060543	9.898032385	-0.5678554	0.014958852
0	0	0	-0.2645781	-2.52051005	0.014980802
125.2196181	36.86119091	230.7147698	7.108827103	-1.04684794	0.015009779

5.217484088	3.51058961	3.469395034	2.712079584	-1.29195825	0.015012735
0	0	0	-8.70511228	-6.94937205	0.015017935
0	0	0	-8.70511228	-6.94937205	0.015017935
0	0	0	-8.70511228	-6.94937205	0.015017935
0	0	0	-8.70511228	-6.94937205	0.015017935
0	0	0	-8.70511228	-6.94937205	0.015017935
0	0	0	-8.70511228	-6.94937205	0.015017935
0	0	0	-8.70511228	-6.94937205	0.015017935
0	0	0	-8.70511228	8.802504911	0.015043942
3.130490453	0.877647403	12.14288262	2.489999981	5.623673448	0.015085119
231.6562935	975.9439116	236.7862111	7.944858541	1.132162903	0.015102982
899.4942568	65.82355519	5.204092551	8.518402631	-2.02083166	0.015128998
77.21876451	35.1058961	43.36743793	5.899184758	-0.93555908	0.015147756
44.87036316	42.12707532	36.42864786	5.575512894	1.121568529	0.015169698
4.173987271	36.86119091	0.867348759	3.737020823	4.235413211	0.015208269
132.5240958	81.62120843	150.918684	6.344972294	0.94421928	0.015236505
0	1.755294805	0.867348759	-8.70511228	7.482092647	0.01524518
0	0	0	-8.70511228	-6.98210739	0.015268706
628.1850842	416.8825162	340.0007134	9.624659296	-0.88088955	0.015293517
1.043496818	44.76001753	20.81637021	3.136775279	3.337289776	0.015309444
388.1808162	816.1418725	614.0829211	8.565874675	0.903959747	0.015322656
0	1.755294805	0	-8.70511228	8.089392858	0.015333051
252.5262299	70.2117922	176.9391467	7.673745275	-1.17882379	0.015362457
63.65330588	41.24942792	28.62250903	4.878965223	1.160735682	0.015399293
62.60980906	165.8753591	122.296175	6.023524688	0.767586151	0.015408515
103.3061849	151.8330006	236.7862111	6.680435196	1.042208492	0.015413266
0	6.143531818	0	1.319635038	-2.66102621	0.015424203
6.260980906	11.40941623	23.41841648	3.999014875	4.505113777	0.015448157
1500.548424	652.0920201	1121.481945	10.16359988	-0.87227794	0.015468507
491.4870011	965.4121428	790.1547191	9.03329177	0.963568124	0.015479085
0	0	0	-8.70511228	-7.0092712	0.01547942
0	0	0	-8.70511228	-7.0092712	0.01547942
0	0	0	-8.70511228	-7.0092712	0.01547942
0	0	0	-8.70511228	-7.0092712	0.01547942
84.52324223	243.1083305	131.8370113	6.633994677	1.226049033	0.01548482
78.26226132	104.4400409	92.76294973	7.008073903	-0.72684501	0.015516465
0	2.764589318	0	-8.70511228	8.221289199	0.015526255

16.69594908	1557.824139	12.14288262	5.157630267	4.405231844	0.015533247
537.4008611	869.7485759	529.9500915	9.23486675	0.703637708	0.015550326
96.00170723	180.7953649	36.42864786	5.037146063	1.824033272	0.015566922
53.2183377	284.3577584	220.3065847	6.251912581	1.376021472	0.015641783
531.1398802	561.6943376	303.5720655	9.089936342	-1.35128855	0.015678662
68.87078997	67.57884999	68.52055193	6.379540267	-0.91439181	0.015679212
123.1326245	82.49885584	169.1330079	6.585438154	1.246923925	0.01570826
1.043496818	0.877647403	4.336743793	-8.70511228	8.452980499	0.015733631
377.745848	784.6167778	711.225982	8.823788695	0.638936346	0.015752432
46.95735679	35.9835435	94.54101468	5.756553532	1.298434552	0.015753152
220.1778285	93.90827207	474.4397709	8.773407997	-1.13595719	0.015763361
7.304477724	1.755294805	3.469395034	2.084264027	1.876157322	0.015767549
2257.083617	2883.071717	2248.167982	11.24643018	1.115603967	0.015770347
256.7002171	203.6141974	734.6443985	7.774612287	0.938189588	0.015783223
17.7394459	9.654121428	24.28576524	4.051518001	1.887754915	0.015798248
217.3812571	42.81164029	62.73533571	6.814858828	-0.9996669	0.015819544
65.74029951	136.9129948	45.9694842	5.911581694	0.957780765	0.01586473
1260.544156	1318.226399	704.287192	10.60211341	-0.69163295	0.015864912
79.30575814	223.8000876	70.25524944	6.62259322	0.887787278	0.01589136
98.08870086	138.6682896	213.3677946	6.623551343	1.019026204	0.015898035
568.7057656	710.0167486	821.3792744	9.35756258	0.707693985	0.01591114
3420.582568	2012.445494	1511.788886	11.73170916	-0.87766477	0.015914061
269.222179	452.8660597	261.9393251	8.137805563	0.605587613	0.015918317
60.52281542	10.53176883	112.7553386	5.198387032	2.728217273	0.015949462
216.0038413	107.9506305	201.224912	7.578048748	-0.5528278	0.015963937
424.7032048	963.656848	603.674736	8.990939606	0.60221254	0.016006907
68.87078997	104.4400409	246.3270474	6.211165385	1.121310617	0.016017724
56.34882815	221.1671454	128.3676163	6.01883047	1.321733553	0.016052372
33.39189817	92.15297726	43.36743793	5.847318282	1.089839333	0.016066505
1.043496818	0	0	-8.70511228	7.61319452	0.016087217
1554.810258	1681.791835	1777.197606	11.10219462	-0.57907456	0.016088582
512.3569375	381.7766201	514.3378138	9.039601252	-0.52456669	0.016093238
898.45076	1007.539218	1234.237283	9.902430216	0.544802418	0.01610921
252.5262299	482.7060714	251.53114	7.661316971	0.855268681	0.016121779
0	0	0	0.467089884	-2.11324382	0.016133406
76.17526769	0	0.867348759	4.568796911	-2.9161095	0.016166619
252.5262299	542.3860947	426.7355892	8.409196674	1.222948378	0.01617561

11.47846499	2.632942208	5.204092551	2.977575121	-1.62200873	0.016179073
100.1756945	109.7059253	179.541193	6.583780896	1.506898788	0.01618378
157.5680195	5.265884415	87.60222462	6.616265354	-1.62531103	0.0161967
19.82643954	2.632942208	0	4.008259105	-1.90042605	0.016201196
18.78294272	23.69647987	118.8267799	5.157583961	2.030493382	0.0162173
1.053931786	0	28.82199925	-8.70511228	10.16939803	0.016239586
16.69594908	4.388237013	13.01023138	3.871876839	-1.0505584	0.01624246
189.9164208	527.4660889	677.3993804	8.127541065	0.936920459	0.016267497
6284.981333	3295.565996	2960.261313	12.49328112	-0.74083373	0.01627351
1125.933066	1522.718243	986.1755385	9.941991541	0.69966017	0.016295275
257.743714	185.1836019	286.2250903	7.439209766	0.838870757	0.016299681
280.700644	218.5342032	148.3166377	8.938905167	-1.33783057	0.016303298
2.149603444	0	0	-8.70511228	7.52821738	0.016307301
234.786784	129.0141682	450.1540057	7.518795621	1.246724546	0.01635935
1.043496818	2.632942208	0.867348759	1.374467228	-2.11984139	0.016366048
365.2238862	615.2308292	298.3679729	8.944054713	0.683782317	0.016369421
42.78336952	113.2165149	137.0411039	5.685842048	0.949252933	0.016407769
245.2217522	198.348313	171.7350542	7.568665489	1.442280133	0.016421437
1766.640112	564.3272798	1021.736838	9.673037747	0.950209096	0.016423061
1.043496818	0	0	3.260670931	-3.40173185	0.01643787
1027.844365	567.8378694	950.6142394	9.600831174	0.664757219	0.016460261
29.21791089	28.96236428	10.4081851	3.956937401	1.161966944	0.016460614
2.80700644	2.255553824	1.092859436	0.042906283	3.242473451	0.01648807
31.30490453	55.29178636	42.50008917	4.327958865	1.205800271	0.016492698
602.0976638	334.3836604	234.1841648	8.71411019	-0.99891617	0.016503377
11.47846499	7.898826623	16.47962641	4.778623885	-1.27689082	0.016514045
151.3070386	68.4564974	2.602046276	5.69470508	3.589573673	0.016515263
97.04520404	84.25415064	99.74510724	7.044298661	-0.65306841	0.016519281
126.2631149	163.2424169	451.0213545	7.035901344	1.150173256	0.016524059
67.82729315	14.04235844	22.55106772	5.401879781	-0.9637263	0.016531028
18.78294272	5.265884415	30.35720655	4.294818414	-1.44950997	0.016537329
4.173987271	9.654121428	0.867348759	3.171239981	2.826879157	0.016539099
520.704912	500.2590194	356.4803398	8.903362425	-0.44511481	0.016555447
0	0	0	1.360702726	-2.67443638	0.016558726
36.52238862	28.08471688	33.82660158	4.103439737	1.147087791	0.016570829
62.60980906	107.9506305	96.2757122	6.041321639	0.928995062	0.016583896
2.086993635	7.898826623	1.734697517	-8.70511228	8.527445905	0.016588752

146.0895545	150.9553532	130.1023138	7.678658918	-0.61649971	0.016598406
72.12650004	128.7859798	105.52165	6.247782766	0.692872384	0.016607349
1.043496818	0	0	-0.04805329	-2.47045631	0.016607932
54.26183452	75.47767662	51.17357676	5.648277527	0.709587989	0.01661754
0	0.877647403	0	0.22856117	-2.46581669	0.01665079
9447.820187	16635.80651	7499.964715	13.85291968	-0.80855281	0.016659266
928.7121677	1153.228687	441.4805181	8.972894486	1.199191874	0.01667254
2.086993635	0.877647403	0	-8.70511228	7.52241718	0.01668431
93.91471359	92.15297726	42.50008917	6.392109605	-0.99732725	0.016691954
539.4878547	179.0400701	241.9903036	10.71949884	5.16625934	0.016712477
827.4929764	236.0871513	21.68371896	9.216063146	-1.37995351	0.016718721
0	0	0	-0.4736489	-2.93874561	0.016726612
9.391471359	46.51531233	13.01023138	3.535891632	1.584818036	0.016743459
22.95692999	223.8000876	106.6838973	5.780006088	1.356694686	0.016751598
77.21876451	142.1964322	93.67366593	7.15026117	0.88382406	0.016764719
398.6157843	638.927309	507.3990238	8.771120695	0.554130806	0.016773755
0	3.51058961	4.336743793	-8.70511228	11.1425295	0.01677994
104.3496818	14.92000584	104.081851	4.091682737	2.206065499	0.016796469
264.0046949	206.2471396	138.7758014	7.738810315	-0.565993	0.016814595
17.7394459	4.388237013	122.296175	4.464055662	2.024067022	0.016837367
35.4788918	57.04708116	39.03069414	5.74931103	1.091412123	0.01683749
80.34925496	175.5294805	181.2758905	6.525988864	0.782007095	0.0168822
0	0	1.734697517	-8.70511228	8.878340858	0.016899919
0	0	0	-1.32931422	-2.82355139	0.016905871
160.6985099	55.29178636	111.8879899	5.780710777	0.964211873	0.01691031
30.26140771	28.08471688	21.68371896	5.390352845	-0.9501259	0.016925098
339.1364657	294.8895272	13.01023138	9.37722433	-1.87870447	0.016928208
147.1330513	74.60002921	85.00017834	6.497058204	-0.67968314	0.016934753
13.56545863	21.94118506	8.673487586	3.016599536	2.104288412	0.016944177
0	41.24942792	19.94902145	2.581183759	3.70001535	0.016954104
1.043496818	0	0.867348759	-0.18066994	-2.71693038	0.016970901
0	0	0.867348759	-8.70511228	12.30124925	0.01700381
416.3552302	864.4826915	476.1744685	8.864807384	0.831155065	0.017007191
183.6554399	78.98826623	49.43887924	6.878218253	-0.73019269	0.017029126
511.3134407	471.2966551	923.7264279	9.456156692	-0.76276976	0.01703403
0	1.755294805	0.867348759	-8.70511228	10.49463589	0.017057524
200.351389	122.8706364	590.6645046	7.60092942	0.984356468	0.017068449

40.69637589	45.63766493	40.76539165	5.766704816	1.197797104	0.017071008
221.2213253	525.7107941	325.2557845	8.208008389	0.73747707	0.017072922
20.4942775	41.31963971	100.1267407	4.561723863	1.458538578	0.017128617
807.6665369	433.5578168	302.7047167	9.540308241	-0.67937406	0.017131532
65.74029951	82.49885584	127.5002675	5.974477116	0.821649903	0.017176606
3.130490453	57.04708116	17.34697517	1.992972906	3.0506557	0.01722929
25.04392362	25.45177467	63.31645938	4.550734825	1.075718704	0.017233174
237.9172744	533.6096207	295.7659267	8.013146842	0.752474848	0.017238946
638.6200524	1209.398121	869.0834561	9.143652617	0.713322881	0.017240609
1.043496818	2.632942208	1.734697517	1.544250257	-2.07349677	0.017251283
459.1385998	587.1461123	475.3071197	9.556388917	-0.8604237	0.017267593
1.043496818	0	0	-1.31530051	-2.961192	0.017270217
0	10.53176883	0.867348759	2.787596526	-1.89205807	0.017271694
775.3181355	1158.494571	942.8081006	9.614106097	1.180446604	0.017281726
601.054167	758.2873558	261.0719763	9.69815242	-1.23340975	0.01732269
153.3940322	285.2354058	246.3270474	7.262939487	0.786876794	0.017335257
14.60895545	18.43059545	20.81637021	3.502722446	1.33194207	0.01733897
1074.801722	2249.410293	1446.737729	9.988580167	0.74365865	0.017379227
0	0	0	-0.80038815	-2.6027386	0.017386965
611.1760861	1609.42103	1399.822835	10.1722214	1.368806396	0.017388404
173.2204717	37.73883831	185.6126343	7.718014892	-1.06423295	0.017424845
524.8788993	435.3131116	350.4088985	9.531008029	-0.75757363	0.01743306
185.7424335	152.710648	163.0615666	7.623009039	-0.62836116	0.017455872
108.523669	66.70120259	83.26548082	6.569048424	-0.66727115	0.017492013
0	0.877647403	2.602046276	-8.70511228	8.012423933	0.017493284
120.002134	188.6941915	153.5207303	7.861535236	-0.86031409	0.017505834
3.130490453	78.98826623	9.540836344	2.507386854	2.71724575	0.017519966
241.0477649	130.769463	458.8274933	7.282730446	1.341283137	0.017534355
0	0	0	-0.10217594	-2.67618165	0.017574654
273.3961662	485.3390136	369.4905711	8.376981088	0.582047736	0.017577894
112.6976563	48.27060714	69.38790069	5.986049581	0.786286357	0.017596061
749.2307151	982.0874434	943.6754493	9.644096781	0.842013078	0.017618996
55.30533134	90.39768246	45.10213545	5.224103739	1.221434827	0.017621062
0	1.755294805	1.734697517	3.071225109	3.83603829	0.017648155
86.61023587	222.9224402	245.4596987	6.96630048	0.815679889	0.017658026
841.058435	227.3106772	275.8169052	9.055367616	-1.23699615	0.017669449
85.56673905	150.0777058	149.1839865	6.536337832	0.684963	0.017673053

663.663976	351.058961	239.3882574	9.052144836	-0.63181376	0.017719307
181.5684463	238.7200935	255.000535	7.538810752	0.684521343	0.017752678
1.043496818	4.388237013	10.24338884	0.130395337	4.014768191	0.017811534
126.2631149	57.04708116	98.01040972	7.427883468	-0.93477224	0.0178221
41.73987271	27.20706948	78.06138827	6.681457066	-1.18224962	0.017832301
1.043496818	0	4.336743793	-8.70511228	7.745088525	0.017834203
9.391471359	19.30824286	25.153114	3.448259916	1.631343178	0.01785885
207.6558667	290.5012902	326.1231332	7.964590757	1.101871209	0.017880425
3.130490453	0.877647403	0	-8.70511228	12.12617161	0.01788425
0	0	0	-8.70511228	8.350355926	0.01788746
262.0324859	171.2377847	170.9023994	6.703608512	1.32248677	0.01790002
6709.684538	693.341448	537.7562303	12.1007115	-2.00616108	0.017920337
39.65287907	0.877647403	26.88781152	1.793780947	3.328611984	0.017961008
60.52281542	55.29178636	66.78585441	6.576651988	-0.75474914	0.01797628
5289.485369	1823.751302	1822.299742	12.10701291	-1.33109643	0.017989568
275.8692537	808.392246	203.679509	10.6018925	-1.73944211	0.018042945
24.00042681	57.04708116	37.29599662	4.268760139	1.489768823	0.018043531
201.3948858	489.7272506	395.5110339	8.018932831	0.931577657	0.01805448
184.6989367	302.7883539	503.06228	7.292612145	1.049867712	0.01806659
0	0	0	-1.54449182	-3.44722214	0.018073125
45.91385998	28.08471688	37.29599662	5.491952757	-0.82526405	0.018074796
423.8579724	517.2590496	607.5778054	9.105469516	0.791296597	0.018091961
110.6106627	153.5882954	159.5921716	6.617687136	0.918762405	0.01813684
1.043496818	0	3.469395034	1.855622432	-1.84764135	0.018142777
1843.858877	2815.492867	2719.138358	10.99567834	0.825675839	0.018166115
4.173987271	20.18589026	30.35720655	2.878580873	2.924158353	0.018186969
31.30490453	7.02117922	19.94902145	3.303309471	1.242507939	0.018189224
250.4392362	380.8989727	326.990482	8.314385827	0.661982147	0.018204708
2.086993635	121.9929889	0	2.475514985	8.465718837	0.018208997
0	0.877647403	0	0.239688115	-1.82133178	0.018220006
0	0.877647403	0	0.239688115	-1.82133178	0.018220006
99.13219768	356.3248454	189.9493781	7.507732589	1.056912096	0.018225457
782.6226132	78.98826623	173.4697517	9.868171998	-1.3383746	0.018247299
97.04520404	14.04235844	209.0310508	7.248159514	-1.62998271	0.018257251
449.7471284	504.6472564	375.5620125	8.848114585	-0.74679889	0.018285809
0	4.388237013	0	-0.00448852	3.745000475	0.018293167
1793.77103	543.2637422	1123.216642	10.20127807	-0.69091538	0.018303581

0	0	0	-2.19393958	-3.25228639	0.018310464
0	0	0	-2.01665655	-3.42831778	0.018310464
0	0	0	-2.01142085	-3.43878918	0.018310464
1.043496818	0	0	-2.00356907	-3.48471931	0.018310464
1.043496818	0	0	-2.00015412	-3.4915492	0.018310464
1.043496818	0	0	-2.00015412	-3.4915492	0.018310464
1.043496818	0	0	-1.99263487	-3.5065877	0.018310464
0	0	0	-2.06668318	-3.51424684	0.018310464
0	0	0	-2.02569336	-3.62253257	0.018310464
0	0	0.867348759	-2.18247351	-3.66039458	0.018310464
0	0.877647403	0	-2.14906962	-3.69314391	0.018310464
0	0	0.867348759	-2.15758423	-3.71017314	0.018310464
0	0	0.867348759	-2.14287545	-3.73959071	0.018310464
0	0	0.867348759	-2.11354894	-3.79824373	0.018310464
0	0	0	-2.30482378	-4.10088853	0.018310465
0	0	0	-2.30482378	-4.10088853	0.018310465
0	0	0	-2.28484756	-4.14084098	0.018310465
134.6110895	258.9059837	276.684254	7.459786856	0.749301571	0.018326391
11.47846499	33.3506013	21.68371896	3.690874638	1.239942876	0.018330228
107.4801722	182.5506597	123.1635237	6.856052018	0.694873663	0.018352948
814.9710146	16.67530065	214.2351434	9.486492735	-1.14614993	0.018358114
9.391471359	16.67530065	37.29599662	3.569087297	1.520443591	0.018362483
6451.940824	1405.113491	2749.495565	11.40993407	1.10032774	0.018368313
529.0528866	150.9553532	148.3166377	8.397321186	-0.85803843	0.018376577
269.222179	126.381226	265.4087201	8.116559316	-0.56104298	0.018429358
8.431454287	31.00728273	160.0605399	3.977237703	1.877697123	0.018429863
9.391471359	7.898826623	14.7449289	3.689071089	2.68403893	0.018450765
0.198264395	7.512661765	4.666336321	1.4542492	3.828938664	0.018469962
408.0072557	9.654121428	271.4801614	8.194816915	-1.98491314	0.018472478
0	39.49413311	31.22455531	-8.70511228	10.92912731	0.018495551
0	0	0	0.297349031	-2.35273355	0.018505563
0	2.632942208	3.469395034	-8.70511228	8.129065611	0.018506244
40.69637589	44.76001753	48.57153048	5.119588125	0.784044617	0.018509639
1.043496818	0.877647403	1.734697517	1.240310015	2.909474167	0.018509666
29.21791089	20.18589026	47.70418172	4.525637353	1.355842637	0.018542048
349.5714339	141.3012318	268.0107664	8.215956197	-0.68251361	0.018543566
146.0895545	76.35532402	87.60222462	6.839780714	-0.96606153	0.018562272

9.391471359	10.53176883	4.336743793	3.045645356	1.454504103	0.018564113
337.0494721	199.2259604	242.8576524	8.443070493	-0.77464216	0.018574209
570.7927593	800.4144311	858.675271	9.057845103	0.606939095	0.018577833
317.2230326	319.4636545	399.8477777	8.331528148	0.610143091	0.018587258
0	0	0	-8.70511228	-7.5060078	0.018588348
4042.579716	3906.882518	1517.452674	11.7860521	-0.92854659	0.018603895
2.086993635	1.755294805	0.867348759	1.819928462	-1.47567376	0.018618259
182.6119431	188.6941915	451.8887032	7.332125709	0.864956306	0.018619773
121.0456308	290.5012902	311.3782043	7.51900355	1.455269647	0.018656074
100.1756945	208.8800818	1838.779368	6.11652547	3.393038267	0.018684645
13.56545863	11.40941623	19.94902145	4.360221937	2.099805915	0.018732311
389.224313	324.7295389	797.0935091	8.666851062	1.605579263	0.018757632
126.2631149	107.0729831	625.3584549	6.99885292	1.452875739	0.018797177
38.60938225	19.30824286	12.14288262	5.092468214	-0.88698743	0.018804192
34.43539498	30.71765909	37.29599662	6.783235771	-1.42152027	0.018821088
0	0	0	-8.70511228	-7.56211581	0.018857833
11.47846499	9.654121428	10.4081851	6.15483572	3.52102115	0.01887766
0	0	0.867348759	-8.70511228	8.513163373	0.01887958
171.1334781	300.1554117	148.3166377	7.253767286	0.906369592	0.01888415
9297.556645	4356.641706	20391.36931	12.64487321	1.85471931	0.01890275
255.6567203	320.3413019	646.1748251	8.213867176	0.826178045	0.018944663
252.3279655	426.5980729	796.5297324	8.015175678	0.935466287	0.018952709
286.9616249	607.3320025	457.9601445	8.434164536	0.83597351	0.018988986
138.7850767	107.9506305	101.4798048	7.256652842	-0.80597309	0.018999869
627.1415874	489.7272506	385.1028488	8.696702852	0.586699124	0.019073261
21767.34362	288.7459954	3014.036936	13.08410547	-1.72732601	0.019080734
131.4284242	30.71765909	287.9424409	5.212011811	2.394629635	0.019088745
771.6241568	548.5910619	745.7464626	9.261753219	1.143067875	0.019095942
0	3.51058961	2.602046276	-8.70511228	8.200019299	0.019159526
0	0	0	-8.70511228	-7.62565212	0.019167129
415.3117334	644.1931934	414.5927066	8.45943292	0.622754047	0.019167142
192.0034145	304.5436487	263.6740226	8.109463007	-0.61247671	0.019181102
193.0469113	222.0447928	946.2774956	9.64371705	1.648389597	0.019198712
44.87036316	7.02117922	66.78585441	5.249978146	1.923909183	0.019206378
409.0507525	602.0661181	366.0211761	8.212278232	0.627333473	0.019249028
1192.716863	1186.579288	827.4507157	9.682059891	0.728937607	0.019276638
91.82771995	143.934174	164.7962641	6.333871036	0.695698409	0.019308371

73.04477724	173.7741857	106.6838973	6.407973958	0.79508752	0.019309141
19.82643954	44.76001753	19.08167269	4.040450926	1.180710894	0.01932525
1.043496818	5.265884415	10.4081851	1.135290683	2.742417867	0.019365071
342.2669562	485.3390136	929.7978692	9.020919966	1.307470928	0.019370938
100.1756945	12.28706364	36.42864786	4.505165541	1.534944803	0.019407305
70.9577836	89.52003506	128.3676163	6.046583623	0.820566126	0.019419711
1889.772737	5189.529091	2920.36327	11.26511894	0.983838918	0.019430437
0	10.53176883	2.602046276	1.389315405	6.715299446	0.019443183
60.52281542	0	2.602046276	0.87712272	5.690914075	0.019497046
141.9155672	211.513024	406.7865678	7.061336252	0.981554244	0.019515699
12.52196181	28.08471688	59.84706434	4.599433545	1.479726516	0.019518064
12.52196181	13.16471104	9.540836344	2.795701821	1.324104861	0.019526992
0	17.55294805	2.602046276	3.04926316	4.625925336	0.019529167
1.043496818	0	6.938790069	-8.70511228	9.121388407	0.019543033
41.73987271	2.632942208	33.82660158	0.538823565	4.831624646	0.019549328
34.43539498	7.898826623	14.7449289	4.956372374	-1.18436288	0.019550177
198.2643954	524.8331467	202.0922607	8.281870419	0.831482486	0.019568691
4.173987271	88.64238765	152.6533815	3.809343702	2.252378688	0.019582281
281.7441408	195.7153708	177.8064955	7.802948538	0.652872962	0.019590766
28.17441408	86.00944545	45.10213545	4.05098879	2.197097965	0.019594127
1227.152258	647.7037831	770.2056976	10.17404435	-0.83123552	0.019625608
89.74072632	21.06353766	215.1024921	4.353959869	2.066305744	0.019655781
4.173987271	57.04708116	16.47962641	3.781336821	2.148102066	0.019675553
6.260980906	0.877647403	0.867348759	3.044026621	-1.85731475	0.019677324
187.8294272	292.256585	218.5718872	7.675814204	0.989209666	0.019717117
12.52196181	13.16471104	5.204092551	3.957282044	2.334668313	0.019718619
257.743714	267.6824578	121.4288262	7.415366542	1.228949421	0.019722849
0	2.632942208	0	-8.70511228	9.798119155	0.019747825
8697.545975	16975.45606	8152.210982	13.18505108	0.751028235	0.01975933
0	0.877647403	1.734697517	-0.3759778	3.002021924	0.019791341
1154.10748	917.1415356	515.2051626	9.615895901	-0.99877317	0.019796852
0	0	0	-0.35052372	-2.50163493	0.019873745
4.173987271	0.877647403	0.867348759	1.57098111	-2.5157149	0.019890693
658.4464919	69.3341448	246.3270474	8.78090122	-1.31742936	0.019915411
2.086993635	12.28706364	11.27553386	4.42861465	-2.59685938	0.019920106
488.3565107	405.4731	442.3478669	8.104111402	0.606901198	0.019965417
234.786784	258.9059837	473.5724222	8.228346153	0.821721899	0.019969486

5.217484088	0	4.336743793	-8.70511228	7.822979492	0.019976422
31.805783	24.96029213	34.80670568	4.124396329	1.615209093	0.019987667
137.7415799	104.4400409	121.4288262	6.505693384	1.226225793	0.019989897
0	0	20.81637021	-8.70511228	9.789951034	0.020002741
7.304477724	6.143531818	0.867348759	3.122478868	-1.27612449	0.020016875
151.1400791	237.3948459	374.9201744	7.615970206	0.605199813	0.020017747
5.217484088	1.755294805	4.336743793	2.100065298	2.760129215	0.020017994
211.829854	208.8800818	622.7564087	7.87827095	0.915376455	0.020052825
175.3074654	301.0330591	275.8169052	7.693328603	0.547481228	0.020084685
510.2699438	337.89425	224.6433285	8.840704416	-0.65861318	0.020131572
82.4362486	0	32.95925283	1.619899002	7.176466639	0.02015283
180.5249495	375.6330883	516.0725113	8.034124643	0.751535088	0.020153794
4.173987271	0	0	0.793192384	5.340362285	0.020160683
0	90.39768246	1.734697517	-8.70511228	10.94383174	0.020167537
0	0	0	-8.70511228	7.588682701	0.020169872
1.043496818	0	0	-8.70511228	7.473619861	0.020169872
66.78379633	119.3600467	178.6738443	6.227392074	0.936554156	0.020182672
733.5782628	1457.772336	483.1132585	9.058371163	0.740251069	0.020211189
30.26140771	14.92000584	9.540836344	3.983852458	1.628581884	0.020212137
1.043496818	693.341448	58.97971558	4.876355155	6.487503444	0.020236978
305.7445676	227.3106772	150.1033762	8.155026158	-0.62130692	0.020254436
5.217484088	0.877647403	6.07144131	2.320203097	-2.3671368	0.020258376
1192.716863	2810.226983	3173.629108	10.47309017	0.982297587	0.020271939
205.5688731	141.3012318	346.9395034	7.897126735	-0.78914159	0.020330307
555.140307	720.5485175	1305.359882	9.02647457	0.956251474	0.020352688
0	0.877647403	0	-8.70511228	7.369705756	0.020355815
1208.369315	1105.835727	366.8885249	10.0764016	-0.75271247	0.020402168
297.396593	280.8471688	377.29671	7.855066851	0.60020635	0.020415231
254.6132235	82.49885584	210.7657483	8.636675666	-0.89679349	0.020421038
102.2626881	111.4612201	373.8273149	6.812680333	0.907208771	0.020432519
225.3953126	352.8142558	183.8779368	7.849778661	0.507437164	0.0204366
12.52196181	0	24.28576524	1.682591112	3.034591245	0.020466395
260.8742044	606.4543551	359.082386	8.029250113	0.934433018	0.020473891
247.3087458	216.7789084	209.8983996	7.305048247	0.744729743	0.020484336
7.304477724	32.47295389	27.75516027	3.228752982	1.836672073	0.020494507
106.4366754	53.53649155	51.17357676	6.627450456	-0.85397758	0.020499178
101.2191913	83.37650324	90.20427089	6.570339765	0.859200629	0.020507742

10.43496818	214.1459662	12.14288262	3.956396901	2.620313751	0.020529748
14544.25864	345.7930766	8927.620772	11.32462082	2.316420872	0.020542604
131.480599	859.2168071	320.0516919	7.646635775	1.163345964	0.02054401
156.5245226	204.4918448	372.9599662	7.360560698	0.788945181	0.020544603
92.87121677	36.86119091	103.2145023	6.491547519	-0.76123045	0.020563005
0	5.265884415	0	-8.70511228	8.626161875	0.020567794
696.0123774	413.3719266	388.5722438	9.678647496	1.987303792	0.020595123
10040.52638	11943.02585	7092.310799	13.37217707	-0.75524992	0.020604865
587.4887083	534.4872681	516.9398601	8.783864313	0.742317052	0.020605469
845.2324223	201.8589026	85.8675271	8.732364502	-1.6766957	0.020618534
49.04435043	8.776474025	59.84706434	5.516858969	-1.11149057	0.02062006
149.2200449	119.3600467	97.14306096	7.149060692	-0.58678006	0.020633924
141.9155672	286.9907006	146.5819402	7.077513567	1.043932261	0.02064406
85.56673905	46.51531233	34.69395034	6.288688989	-0.70030857	0.020669472
0	0	7.970935091	-8.70511228	10.94003289	0.02070344
1150.97699	354.5695506	865.614061	11.04860327	-1.64824022	0.020719694
24.00042681	25.45177467	45.9694842	5.7186604	2.355845498	0.0207221
3.130490453	14.04235844	7.806138827	2.73068554	1.990513217	0.020758509
525.9223961	951.3610078	918.5049883	9.147519576	0.498997978	0.020804817
113.7411531	171.1412435	107.5512461	6.899817213	0.65508269	0.020815538
450.7906252	153.5882954	751.9913737	8.504243149	0.991158463	0.020848196
1631.465535	7184.360202	6849.097533	11.3980091	1.192544495	0.020877081
182.6119431	215.0236136	187.3473319	7.227344221	0.773684196	0.020892982
45.91385998	36.86119091	73.72464448	4.974380067	1.055981835	0.020898182
2267.257711	1123.274581	7781.358673	11.57942934	1.202980597	0.02090821
7.304477724	43.88237013	32.95925283	4.995707956	1.848039193	0.020922065
392.3548034	138.6682896	149.1839865	7.997158779	-0.67681808	0.02093105
854.6238937	295.7671746	215.1024921	9.442312506	-1.08112103	0.020936456
25.04392362	25.45177467	37.29599662	5.019522565	1.154659724	0.020941046
280.700644	778.473246	539.4909278	8.327530022	1.568735808	0.020952157
1.043496818	8.776474025	1.734697517	2.100409049	2.691056811	0.020969303
377.745848	824.110911	566.3787393	8.877015162	0.880583117	0.020990589
0	3.51058961	0	2.647876608	-1.80586925	0.020996049
267.1351853	272.9483422	394.6436851	8.723737565	-0.66858484	0.021002478
176.3509622	272.9483422	196.0208194	7.684789088	0.744770953	0.02100323
1556.897252	2251.165587	577.6542732	10.02464158	1.143267811	0.021057628
2.086993635	11.40941623	2.602046276	2.200108874	6.041979251	0.021066387

307.8315612	0.877647403	249.7964425	2.531764323	6.695381988	0.021115855
1.043496818	0.877647403	0	-8.70511228	11.65641006	0.021135941
521.7484088	708.2614538	291.4291829	9.469848063	-0.71191629	0.021171565
3.130490453	43.00472272	52.90827427	-8.70511228	11.10368842	0.021175978
5023.39368	3426.335459	137.0411039	12.33665231	-1.48181925	0.02122153
13.56545863	3.51058961	6.07144131	3.50279037	-1.57710316	0.021265783
823.3189891	1121.63338	1053.828742	10.53899676	-0.93282529	0.021278174
1.043496818	0	15.61227765	-8.70511228	10.87278948	0.021300301
144.0025608	459.0095915	184.7452856	7.78579039	0.760821913	0.021307186
50.08784725	28.96236428	46.83683296	5.674416727	-0.8966214	0.02133847
16.69594908	4.388237013	14.7449289	3.225412719	1.710686027	0.021357415
60.52281542	75.47767662	85.8675271	5.8665722	0.960999718	0.021393088
13.56545863	0	13.01023138	3.627741901	-1.37489976	0.021411307
42.78336952	108.8282779	137.9084526	5.383127894	2.616358744	0.021435456
6435.244875	2667.170456	3573.476885	12.01826569	-1.00307461	0.021444568
15.94463137	11.06713375	8.300527619	3.106336188	2.180799955	0.021454503
26.08742044	8.776474025	604.5420847	6.998744968	3.553964753	0.021517969
65.74029951	541.5084473	33.82660158	5.890631665	2.047352886	0.021559667
108.523669	57.92472857	128.3676163	5.958273286	0.817968904	0.021573537
26.08742044	0.877647403	14.7449289	1.866212494	2.26669008	0.021596053
40.69637589	86.88709285	76.32669075	5.64857503	0.872081474	0.02161394
7.304477724	28.08471688	0	-8.70511228	9.668633187	0.021624623
665.657055	1253.60522	722.1459029	9.296319579	0.659220519	0.021631359
25.04392362	21.06353766	56.37766931	4.547682066	1.599974462	0.021705584
55.30533134	15.79765325	67.65320317	6.515175752	-1.37324423	0.021709055
0	0	0	-0.36712315	-2.57471491	0.021723181
0	0	0	-0.36712315	-2.57471491	0.021723181
138.7850767	726.6920493	789.2873703	8.646636807	2.386790534	0.021741714
298.4400899	418.637811	664.3891491	8.32121262	0.62378306	0.02176273
41.73987271	4.388237013	1.734697517	6.168203712	-1.92583129	0.021785138
20.86993635	78.11061882	19.94902145	4.937322613	2.291659985	0.021811933
173.2204717	120.2376941	144.8472427	7.454747586	-0.62771105	0.021838391
181.5684463	109.7059253	111.0206411	7.336656193	-0.64885435	0.021880682
523.8354025	1176.925167	810.1037405	8.76350846	1.059168876	0.021920144
43693.29875	17993.52705	6713.279391	14.75297916	-0.94596312	0.021935204
707.4908424	694.2190954	618.4196649	9.982138198	-0.8390754	0.021940914
187.8294272	1110.223964	339.1333646	8.327507497	0.838376191	0.021941034

257.743714	836.3979746	451.0213545	8.596696759	0.653947914	0.021942773
72.00128042	12.28706364	71.98994696	6.514862342	-1.08571806	0.021946867
0	0.877647403	0	1.461401742	-2.3515096	0.02195597
1.043496818	8.776474025	17.34697517	2.101891073	2.215247408	0.021976338
5.217484088	188.6941915	195.1534707	6.921700369	1.94706533	0.02199142
0	0.877647403	32.09190407	-8.70511228	10.0263362	0.022037993
2.253953126	0	4.675009809	-8.70511228	7.951033581	0.022049615
9.391471359	16.67530065	29.48985779	2.581443387	1.665824493	0.022078515
3.130490453	320.3413019	7.806138827	3.991285535	4.182298974	0.022112744
21.91343317	43.70684064	78.06138827	5.072692693	0.793735377	0.022117137
520.704912	454.6213545	214.2351434	8.754934399	-0.91556599	0.022132509
38.60938225	21.06353766	185.6126343	4.402725336	1.945026629	0.02216866
0	0	0	3.143621614	-3.17840695	0.022176707
248.3522426	127.2588734	173.4697517	7.738597628	-0.69404322	0.022194586
20.86993635	14.04235844	27.75516027	5.046859326	-0.77958154	0.022198372
138.7850767	104.4400409	255.000535	7.328181646	0.903535619	0.022224234
3313.102396	5286.947953	3074.751349	11.81613877	0.733912181	0.022242109
531.1398802	472.1743025	130.9696625	8.392031365	-1.47816835	0.022330968
85.56673905	234.3318565	322.6537382	6.837192104	0.83581324	0.022345169
237982.6912	72968.92152	78719.11618	16.30045289	1.302837125	0.022351375
235.8302808	363.3460246	235.9188623	7.976757369	0.556070481	0.022370666
60.52281542	53.53649155	45.10213545	6.169529704	-0.67214493	0.022371876
0	0	0	-0.63799996	-2.57144748	0.022374781
6.260980906	28.08471688	1.734697517	3.164394134	9.970549771	0.02240104
1507.852902	2457.412727	1693.932125	10.49568574	0.67119223	0.022411351
13.56545863	2.632942208	0.867348759	2.948692178	-1.50834039	0.02245658
0	0	0	-8.70511228	17.30674834	0.022486865
7.304477724	1.755294805	1.734697517	2.270278205	-2.09496413	0.02249271
403.8332684	358.0801402	380.766105	8.552597339	0.619860857	0.022495456
291.1356121	570.4708116	333.0619233	9.147425434	-0.56117283	0.02250679
925.5816773	399.3295681	1158.777941	9.867769213	1.299257152	0.022513929
0	0.877647403	0.867348759	0.947677384	-2.1221794	0.022533019
299.4835867	221.1671454	266.2760689	7.173995943	1.264419149	0.022565063
15.65245226	15.79765325	83.26548082	3.480569825	2.163478426	0.022575108
2805.962943	2971.714105	3851.895837	10.88386886	0.982234962	0.022578318
116.8716436	170.2460431	683.3580664	7.619425336	1.423819375	0.022589792
1044.540314	2943.629388	2399.954015	10.22503842	1.027922623	0.022627501

569.7492624	383.5319149	298.3679729	9.066355842	-0.74263541	0.02268772
3.130490453	0.877647403	3.469395034	1.095562236	5.705813338	0.022695207
268.1786821	514.3013779	460.5621908	7.993642533	0.661079137	0.022703934
42.78336952	13.16471104	19.94902145	5.183883876	-1.04106163	0.022722165
5.217484088	3.51058961	0.867348759	2.843650978	-1.51998546	0.022739729
21.91343317	118.4823993	24.28576524	5.15358988	1.326829383	0.022758859
78.26226132	358.9577876	185.6126343	6.771377281	1.018206016	0.022780181
211.829854	286.1130532	142.2451964	7.562137737	0.583439056	0.022795333
649.0550206	592.4119967	1029.542976	8.676221643	0.955695647	0.022803059
9.391471359	0.877647403	0.867348759	2.349464594	-1.9904891	0.022809255
0	0	0	-1.5880744	-3.5343873	0.02285042
12.52196181	7.02117922	4.336743793	1.275962644	3.023761789	0.02285142
0	0	0.867348759	-8.70511228	7.637445442	0.022868157
0	9.654121428	15.61227765	-8.70511228	9.687376455	0.022872542
20.86993635	4.388237013	13.01023138	4.821251417	-1.10966247	0.022918427
8.347974541	23.69647987	41.63274041	4.745903798	1.237040227	0.022982011
11.47846499	23.69647987	19.94902145	3.589172174	1.429970447	0.023009005
5.217484088	7.02117922	19.94902145	1.949652344	1.557434309	0.023011551
18.78294272	36.86119091	102.3471535	4.861094619	1.98879295	0.02301532
78.26226132	159.7318273	99.74510724	6.523751219	0.871934584	0.023029548
0.34435395	0.289623643	0	1.295978452	-2.45165438	0.023048229
7.304477724	0	37.29599662	-8.70511228	12.34379487	0.023060582
69.91428678	116.7271045	92.80631717	6.433316529	0.758015948	0.023093479
207.6558667	229.0659721	237.6535598	7.695073772	0.701794554	0.023098363
1.043496818	0.877647403	0	-8.70511228	7.47741718	0.023114482
154.6462284	335.7001315	186.532024	7.030525507	1.443225214	0.023115031
193.0469113	166.7530065	177.8064955	7.876089398	-0.49007268	0.023127019
65.74029951	215.901261	176.071798	6.394206554	1.139926474	0.023144055
24.00042681	51.78119675	1145.76771	4.997383567	3.667909544	0.023174166
0	2.632942208	0	-8.70511228	8.333704489	0.02325665
2403.173171	1613.993573	1502.24805	10.99454594	-0.56128024	0.023263765
229.5692999	816.2120843	426.7355892	7.93886627	0.919671306	0.023271448
5.217484088	1.755294805	0.867348759	2.970056091	-1.71681881	0.023284708
761.7526769	264.1718682	34.69395034	9.850230829	-1.31929351	0.023296788
233.7432872	318.6123365	273.2148589	7.745570789	0.776756849	0.023326355
0	0	0.867348759	0.221138111	-2.53365152	0.023351192
104.3496818	139.545937	108.4185948	7.166006581	-0.56488572	0.023403864

916.1902059	836.3979746	717.2974233	10.58009305	-0.76906916	0.0234061
0	2.632942208	0	-8.70511228	8.142090534	0.023417761
125.2196181	104.4400409	168.2656592	7.153740453	1.168065806	0.023446591
190.9599176	765.308535	294.8985779	7.753934458	1.093445624	0.02345545
6.260980906	0	4.336743793	3.632191222	-2.10375271	0.023463368
7.304477724	13.16471104	4.336743793	2.431772526	1.735879631	0.023502025
1164.542449	1265.567554	1380.758509	10.19598329	0.704563064	0.02350689
1.043496818	0	0	-0.29549016	3.162997202	0.02350911
15.65245226	35.1058961	17.34697517	4.468739522	1.16376943	0.023526958
168.7334354	61.35632991	36.51538274	9.656203281	-2.26704677	0.023534383
181.5684463	204.4918448	222.908631	7.041167865	0.690890511	0.023563328
631.3155747	581.8802279	1189.135148	9.319605586	0.531105379	0.023567341
428.8771921	666.3274609	427.2386515	9.002508409	0.638529487	0.023567484
0	0	0.867348759	-0.58065128	-2.54577855	0.023621399
150.2635417	132.5247578	69.38790069	7.124840752	-0.68335284	0.023642546
80.34925496	322.9742441	56.37766931	6.765492312	0.900731903	0.023655115
43.82686634	56.16943376	88.46957337	5.56941923	0.825036769	0.023659477
3.130490453	78.98826623	18.21432393	2.821307926	4.086321088	0.02373985
16.69594908	78.11061882	57.24501807	5.29710745	2.141553554	0.023791332
0	0	0.867348759	-8.70511228	7.65290193	0.023802562
350.6149307	510.7907883	300.1026705	8.359998424	0.531263833	0.023815013
274.439663	136.9129948	327.8578307	8.270489301	-0.56419162	0.023844837
130.4371022	332.6283656	295.7659267	7.495406145	0.715782471	0.023846322
3.130490453	1.755294805	4.336743793	0.718437492	2.041870308	0.023847035
4002.853793	1412.134671	1949.800009	11.40852108	-0.91478079	0.023850388
12.52196181	4.388237013	16.47962641	1.640627834	2.283266363	0.023864922
162.7855036	178.1624227	267.1434176	7.464223087	0.855748734	0.02388158
1.043496818	3.51058961	0.867348759	2.365130047	-1.63193203	0.023902255
376.7023512	365.9789668	385.9701976	8.642881123	-0.51114979	0.02390347
227.4823063	96.54121428	287.9597878	6.828626873	1.244315042	0.023917363
170.0899813	237.8424461	289.6944854	7.343108882	0.548923756	0.02392424
0	0	0	-0.76676771	-2.37508589	0.023927837
663.663976	458.1319441	580.2563195	8.234098458	0.986177331	0.023947425
109.5671659	87.76474025	141.3778476	7.310464711	-0.68402044	0.023957263
0	3.238518915	0	-8.70511228	8.38093055	0.02399129
0	1.755294805	0	2.195479093	8.032719688	0.024001
319.3100262	272.9483422	222.908631	7.909002292	0.635205317	0.024036548

751.3177087	1735.986562	1646.227944	9.769795238	0.58095156	0.024059568
988.1914863	1220.807537	334.7966208	9.070348898	0.806803368	0.024109998
1.043496818	6.143531818	0	0.092503777	3.938985071	0.024113416
0	7.898826623	2.602046276	-8.70511228	9.604925967	0.024114831
32.34840135	44.76001753	19.08167269	4.257319615	0.982670238	0.02412968
273.3961662	417.7601636	413.7253578	8.389467975	0.845046765	0.024151907
794.1010782	285.2354058	665.2564978	9.442475402	-1.1532434	0.024171691
20.86993635	3.51058961	39.03069414	4.943002068	-1.5179867	0.02418451
22.95692999	21.06353766	4.336743793	3.689811137	1.625056111	0.02419896
115.8281468	304.5436487	186.4799831	7.264341934	0.710215334	0.024232591
1.88872924	5.739814012	7.918894166	2.285628195	2.296171681	0.02424394
554.1802899	997.762226	526.2812062	9.136627534	0.560166232	0.024258753
178.7614398	1916.281668	272.1219995	8.765178835	1.231714095	0.024335296
0	65.82355519	0	1.827886867	5.170462601	0.024377886
180.5249495	186.9388967	316.5822969	6.3485759	1.585581583	0.024422217
187.8294272	26.32942208	744.1852348	6.005506183	2.78237689	0.024424139
64.6968027	1.755294805	32.09190407	5.206838935	-1.29531261	0.024433212
190.9599176	105.3176883	148.3166377	7.890701409	-0.60244899	0.024444895
107.4801722	46.51531233	52.90827427	5.675280534	1.23410475	0.024447317
5.217484088	0.877647403	9.540836344	1.58824905	3.648334601	0.02444763
55.30533134	74.60002921	49.43887924	5.72692929	0.803345685	0.024491439
0	0	0.867348759	4.003960824	7.822436966	0.024492151
165.915994	105.3176883	109.2859436	8.280759286	-0.7402944	0.024519615
0	0	0	-1.00189871	-2.82198165	0.0245274
7.387957469	4.081060422	2.671434176	1.722906607	1.730654705	0.024536404
92.87121677	37.73883831	104.081851	6.334122599	-0.76999605	0.024543413
1009.061423	2194.996154	1655.76878	10.34055472	0.976366367	0.024550359
168.6499557	227.0386066	170.2518878	7.412335336	1.087897107	0.024568806
4279.338709	11380.73472	10705.07858	12.2379334	0.864197159	0.024576228
83.47974541	110.5835727	74.59199324	6.340111704	0.587777719	0.024586366
24.00042681	0	16.47962641	-8.70511228	9.949766882	0.024669296
0	2.632942208	0	-8.70511228	8.694524773	0.02473054
35.4788918	24.57412727	45.9694842	4.940077226	1.708659275	0.024738687
77.21876451	408.1060422	255.000535	8.458368442	-0.80285333	0.024749637
0	0	0	-8.70511228	10.47808886	0.024760565
126.7013836	332.9355421	278.8959933	7.401548413	0.831377001	0.024766112
0	0	0	0.681993816	-2.30983124	0.024786685

1.043496818	3.51058961	1.734697517	1.365037546	3.296903878	0.024868154
2289.432018	812.7014947	7412.362491	10.17970634	1.561908874	0.024902493
2040.036279	1824.62895	983.5734922	11.03681777	-0.68777111	0.024912074
4.194857207	1.43934174	2.072963533	-8.70511228	8.018862724	0.024926077
0	0	80.66343455	-8.70511228	13.09650672	0.025004149
61.56631224	215.901261	129.234965	6.505074139	0.924219465	0.025025441
3.130490453	0	0	1.170061488	-2.18358026	0.025025941
1.043496818	97.41886168	3204.853663	-8.70511228	15.63023315	0.025027789
210.7863572	370.3672039	244.5923499	7.850125102	0.800093408	0.025040628
26.08742044	0.877647403	57.24501807	4.862229438	-1.44768588	0.025045796
75.13177087	15.79765325	36.42864786	5.822001794	-0.98554181	0.025060307
16.69594908	78.98826623	52.04092551	4.742395974	1.430676044	0.025089537
254.6132235	500.2590194	171.7350542	8.368572085	-0.70188733	0.025101719
69.91428678	66.70120259	92.80631717	6.128091173	1.215860086	0.025107438
303.6575739	327.3624811	222.908631	7.752136689	0.642480899	0.02512707
8.347974541	3.51058961	27.75516027	5.208655995	3.68028304	0.025182295
69.91428678	151.376624	158.7248228	6.671793484	0.623363847	0.025245295
217.0473381	123.7482838	225.5106772	7.924579326	-0.8015279	0.025250271
2.086993635	176.0911748	51.66796555	4.728677969	3.079040173	0.025257667
40.69637589	182.5506597	159.5921716	6.318162755	0.972606049	0.025266354
13.05414519	21.8621968	38.30212118	6.79303043	-1.86491863	0.02530216
207.6558667	73.72238181	184.7452856	7.322782567	-0.80012368	0.025302791
1668.551411	5413.329179	520.4092551	11.89651987	-1.17260395	0.025319495
1899.164208	1388.438191	544.6950204	12.00370121	-1.12436232	0.025343338
3.130490453	0	0.867348759	1.590159189	-2.22763205	0.025361836
90.78422314	176.4071279	160.4595203	6.537205043	0.777921369	0.025401841
114.7846499	115.8494571	83.26548082	6.959052525	-0.62321089	0.025417486
232.6997903	150.9553532	176.9391467	8.521530897	-1.0902262	0.025440794
486.269517	345.7930766	701.6851457	8.771174664	1.169007478	0.025460782
248.3522426	411.6166318	546.4297179	8.258459604	0.633039939	0.025484471
539.4878547	552.0402162	637.5013375	8.783416609	0.438629996	0.025543479
180.5249495	230.8212669	366.8885249	7.564295818	0.82137282	0.025543672
5.217484088	10.53176883	3.469395034	3.4430439	1.511383174	0.025678636
3.130490453	0	0	1.475495339	-2.48342434	0.025694577
4.173987271	0	0	-0.39055272	-2.71075201	0.025705717
2.086993635	0	1.734697517	-8.70511228	7.881780115	0.025728111
584.3582179	123.7482838	5.204092551	8.006711192	-1.74351727	0.025739294

2292.562508	826.7438532	982.7061435	11.22603756	-0.73722239	0.025761648
20.86993635	22.81883247	66.78585441	4.256973868	1.067288128	0.025765598
1.043496818	0.877647403	0.867348759	-0.02714921	3.699679106	0.025770153
1.043496818	0	0	-0.45677046	-2.42662985	0.025775639
139.8285736	147.4447636	151.7860327	7.271184865	-0.82626918	0.025802367
13.56545863	95.66356687	21.68371896	4.092171581	1.354655348	0.025803971
31.30490453	31.59530649	18.21432393	4.832863226	-1.09614347	0.025827346
305.7445676	406.9212182	486.4698982	8.554402897	0.642123914	0.025900395
105.3931786	101.8070987	175.2044492	7.312932129	-0.49232431	0.025901013
3.130490453	0	1.734697517	1.766260717	-1.99223034	0.025904139
221.2213253	387.0425045	414.5927066	8.164738466	0.547922771	0.025913007
286.9616249	304.5436487	78.06138827	8.692446128	-0.91480893	0.025915752
67.82729315	76.35532402	52.04092551	5.834696896	0.618070942	0.025920454
0	3.51058961	2.602046276	1.698632541	4.974790788	0.025924166
0	1.755294805	0.867348759	-8.70511228	7.448914562	0.025964166
121.0456308	226.4330298	308.776158	7.488778026	0.674984009	0.025996705
0	0.877647403	0	-1.0317905	-2.95345968	0.026022031
6.260980906	8.776474025	0.867348759	2.774222313	-1.66345601	0.02604455
321.3970198	445.8448805	224.6433285	7.808212772	0.603669351	0.026071269
5648.448274	2524.991577	5545.827962	11.76159639	0.819811104	0.026115437
0	0	0.867348759	-8.70511228	7.162156484	0.02613038
0	0.877647403	0	-8.70511228	7.184961055	0.02613038
310.9620517	571.348459	292.2965316	8.415458786	0.726857124	0.026149222
386.0938225	1241.871075	396.3783827	8.856513757	0.65938742	0.026156223
64.6968027	138.6682896	152.6533815	6.118158388	2.70365004	0.026174813
70.9577836	367.7342617	220.3065847	6.767066131	1.00967374	0.026177996
194.0904081	78.11061882	100.612456	7.736999171	-0.7832255	0.026184253
0	0	0.867348759	-0.5400521	-2.47857146	0.026192672
0	0	3.469395034	-8.70511228	9.523867266	0.02622011
50.08784725	34.2282487	12.14288262	5.439471684	-0.76256333	0.026253206
38.60938225	211.513024	104.9491998	6.285048769	0.857098679	0.026280677
103.3061849	126.381226	92.80631717	7.239616644	-0.78072526	0.026285196
211.829854	298.4001169	130.1023138	8.293080785	-0.69533159	0.026301649
11.47846499	19.30824286	6.938790069	4.110899235	-1.46266438	0.026321867
18.78294272	9.654121428	49.43887924	4.941557595	2.432922874	0.026344004
90.78422314	78.98826623	82.39813206	6.847001129	-0.48758681	0.026355195
26.08742044	298.4001169	169.1330079	6.05010111	2.137623542	0.026390333

1.043496818	1.755294805	3.469395034	-0.2759188	3.202139911	0.026399095
107.4801722	265.0495156	66.78585441	7.068619658	1.282099047	0.026404169
1092.541168	1926.436049	962.757122	9.865698796	1.184575556	0.026424103
99.13219768	78.11061882	57.24501807	6.434528007	-0.54753641	0.026461959
279.6571471	640.6826038	117.9594312	9.066390576	-1.22949184	0.026476365
53.2183377	12.28706364	21.68371896	4.514311003	1.099451941	0.026480017
312.0055485	494.1154876	450.1540057	8.448315345	0.601706721	0.0265286
289.0486185	473.05195	325.2557845	8.477998444	0.531523252	0.026537136
1146.803003	673.1555577	1354.798761	9.391049412	0.772758831	0.026583573
3103.317796	2101.061552	1095.461482	10.77050799	1.06732367	0.026586304
99.13219768	401.0848629	241.1229549	7.148205088	0.929906278	0.026617423
330.7884912	114.9718097	537.7562303	9.266983407	-0.99705705	0.026651902
850.4499064	1056.687473	0	9.462351679	-3.11366225	0.026667365
37.56588544	32.47295389	26.02046276	4.328676884	1.739616544	0.026681604
1121.759079	566.0825746	473.5724222	10.33394777	-0.69900157	0.026747705
2.086993635	17.55294805	0.867348759	4.250108203	-1.9428472	0.026753733
35.4788918	14.04235844	7.806138827	4.80684986	-0.9456689	0.026755543
240.0042681	322.9742441	272.3475102	7.639137399	0.798666617	0.026781617
1736.378705	1060.198062	719.8994696	10.89231278	-0.77402803	0.026795333
3.130490453	10.53176883	5.204092551	3.245485602	-1.06874687	0.026851918
803.4925496	478.3178344	358.2150373	6.883940898	3.978365143	0.026873647
62.60980906	33.3506013	13.87758014	5.375676302	-0.91141553	0.026888589
177.394459	1253.280491	253.2658375	8.402827266	0.931204181	0.026890354
434.2929405	466.092206	390.8793915	8.462824932	0.41967595	0.026912806
51.13134407	54.41413896	148.3166377	5.559690829	1.212245072	0.026913393
1647.681475	1147.085155	502.1949312	10.24432206	-0.66763157	0.026924661
223.308319	70.2117922	49.43887924	6.501627907	1.163784813	0.026965935
0	1.342800526	1.188267799	1.311352072	-1.7847964	0.027001355
0	1.755294805	0.867348759	1.072492642	3.8989628	0.027033065
8.347974541	1.755294805	6.07144131	3.435339864	-1.23040874	0.027046887
12129.34613	12757.41243	12476.06597	13.60490357	-0.92168045	0.02705462
73.04477724	121.9929889	167.3983104	6.534394129	0.842949165	0.027077701
0	7.02117922	15.61227765	2.435839347	3.390620441	0.027086061
349.5714339	388.7977993	416.3274041	7.947193789	0.70512009	0.027160966
2.086993635	2.632942208	2.602046276	0.601183958	4.956345432	0.027196274
389.224313	168.5083013	323.5210869	8.616897198	-0.59516027	0.027203995
14.60895545	21.06353766	10.4081851	2.191418156	2.388572229	0.027205322

5629.863596	1462.160573	1797.779792	11.93299733	-0.81224082	0.027209744
495.6609884	705.6285116	201.224912	9.456298706	-0.82632112	0.027223184
1330.865406	2042.654118	1984.164367	10.39057999	0.71418629	0.027246499
71.95954055	86.42193972	58.84961327	7.189961793	-1.27099032	0.027248169
63.65330588	1570.98885	584.5930633	9.464190823	-1.38155261	0.02725316
0	0	0	-8.70511228	-6.82969974	0.027258598
0	0	0	-8.70511228	-6.82969974	0.027258598
0	0	0	-8.70511228	-6.82969974	0.027258598
0	0	0	-8.70511228	-6.82969974	0.027258598
0	0	0	-8.70511228	-6.82969974	0.027258598
25.04392362	13.16471104	15.61227765	3.926982084	1.307274454	0.027291876
211.829854	66.70120259	321.7863894	7.376083428	0.986096138	0.027321418
0	2.632942208	278.4189515	1.816024949	5.386027414	0.027322042
1.043496818	17.55294805	10.4081851	1.282623637	2.345485013	0.027335144
2578.480636	2859.375237	2839.699836	12.47351284	-0.88978251	0.027341344
14.60895545	12.28706364	11.27553386	4.075088049	1.90952468	0.027465867
205.5688731	153.5882954	124.8982212	7.315966075	0.665885987	0.0274846
502.9654661	160.6094747	511.7357676	8.028758716	1.046028733	0.027485137
8.347974541	1.755294805	2.602046276	1.073401164	1.882720651	0.027491437
313.0490453	1266.445202	634.0319425	8.71591218	0.73902785	0.027502096
2.097428604	0	0.867348759	0.359036821	-1.84916488	0.027502262
0	0.895200351	0	-8.70511228	8.689206513	0.027502844
3879.721168	2694.377526	1046.889952	11.67410642	-0.8209174	0.027558612
57.39232497	64.94590779	274.0822077	6.17635251	1.17039917	0.027571203
2.086993635	0.877647403	5.204092551	-8.70511228	8.021776426	0.027611737
992.3654736	545.019037	243.7250012	10.02910324	-0.84255262	0.027612095
434.0946761	828.499148	1298.421092	8.720654262	0.841798891	0.027623876
426638.8914	74151.55139	74330.92126	17.57664858	-1.06750304	0.027650974
452.8776189	363.3460246	313.9802506	8.57752858	-0.63428801	0.027675183
78.26226132	26.32942208	163.0615666	5.64316728	1.079764844	0.02767651
93.60166454	89.95008228	113.7788101	7.196168643	-0.57817715	0.027698765
122.0891277	12.28706364	72.85729572	6.675552217	-1.21918012	0.027703786
35.4788918	90.39768246	96.2757122	5.912172681	0.743770231	0.027716704
157.6410642	157.5377088	297.3358279	7.565718134	0.626199344	0.027743569
104.3496818	136.9129948	140.5104989	6.61720898	0.708964689	0.027813037
1960.73052	4777.912459	2362.658018	10.86531432	0.825428308	0.027847809
158.6115163	384.4095623	77.19403951	7.360890354	0.730317412	0.02788339

7.304477724	7.898826623	13.87758014	3.037264048	1.330575049	0.027897142
261.9177012	72.84473441	140.5104989	7.797267664	-0.79800794	0.027916888
3.130490453	10.53176883	9.540836344	3.858255535	-1.26738996	0.027917567
254.6132235	309.8095331	414.5927066	8.345944845	0.55603858	0.027920462
0	0	0	-8.70511228	-6.9210453	0.027936781
0	0	0	-8.70511228	-6.65222854	0.027936781
0	0	0	-8.70511228	-6.9210453	0.027936781
0	0	0	-8.70511228	-6.9210453	0.027936781
0	0	0	-8.70511228	-6.9210453	0.027936781
0	0	0	-8.70511228	-6.9210453	0.027936781
0	0	0	-8.70511228	-6.9210453	0.027936781
51.13134407	5.265884415	0	1.921649788	5.485061079	0.02795099
0	0	14.7449289	-8.70511228	9.71254652	0.027977909
1172.890423	3.51058961	1122.349294	4.745748843	5.340286002	0.028005972
44.87036316	21.94118506	8.673487586	3.924968475	1.487250321	0.028022936
12.85588079	34.21069575	40.30569681	4.374851896	1.088179081	0.028032718
101.2191913	6.143531818	64.18380813	6.137673368	-1.33954322	0.028036113
4.173987271	5.265884415	2.602046276	2.293883487	1.928910438	0.02805248
11.47846499	42.12707532	16.47962641	4.081439113	1.122468788	0.028060763
4.173987271	0	0	0.551031557	-2.3661689	0.028076648
327.6580007	267.6824578	26.02046276	9.929982841	-1.62879851	0.02810992
41.73987271	13.16471104	26.88781152	6.391144276	-1.25430141	0.028112626
0	8.776474025	28.62250903	3.078251794	3.249066971	0.02812292
204.5253763	337.89425	256.7352325	8.049338876	0.796310691	0.028140163
156.5245226	84.25415064	116.2247336	7.181101533	-0.64502775	0.028155687
348.5279371	709.1391012	534.2868353	8.556807742	0.619936686	0.028183912
1.043496818	0.877647403	2.602046276	1.244495972	-1.76468837	0.028190909
0	0	1.734697517	0.570531428	-2.03792264	0.028207236
1.043496818	0	15.61227765	-0.10357644	3.729515759	0.028222005
132.5240958	329.1177759	222.0412822	7.308258546	0.878387333	0.028232089
128.3501086	175.5294805	167.3983104	6.583762783	0.802246352	0.02824507
187.8294272	130.769463	109.2859436	7.577680409	-0.55173054	0.028277011
271.3091726	182.5506597	8.673487586	7.233876524	-1.58046043	0.02830231
114.7846499	85.13179804	87.60222462	6.980911043	-0.54923383	0.028360761
0	0	0	-8.70511228	-6.97715331	0.02836238
0	0	0	-8.70511228	-6.97715331	0.02836238
0	0	0	-8.70511228	-6.97715331	0.02836238

0	0	0	-8.70511228	-6.97715331	0.02836238
0	0	0	-8.70511228	-6.97715331	0.02836238
0	0	0	-8.70511228	-6.97715331	0.02836238
0	0	0	-8.70511228	-6.97715331	0.02836238
0	0	0	-8.70511228	-6.97715331	0.02836238
0	0	0	-8.70511228	-6.97715331	0.02836238
0.313049045	0	0	-3.21991831	-4.52595201	0.02836238
0	0	0	-8.70511228	-6.97715331	0.02836238
0	0	0	-8.70511228	-6.97715331	0.02836238
0	0	0	-8.70511228	-7.19127811	0.02836238
0	0	0	-8.70511228	-6.97715331	0.02836238
0	0	0	-8.70511228	-6.97715331	0.02836238
0	0	0	-8.70511228	-6.97715331	0.02836238
0	0	0	-8.70511228	-6.97715331	0.02836238
0	0	0	-8.70511228	-6.97715331	0.02836238
563.4882815	511.6684357	153.5207303	10.15197295	-0.98265545	0.028372626
264.0046949	264.1718682	177.8064955	8.192462147	-0.52311258	0.028378504
38.60938225	10.53176883	18.21432393	5.384438945	-1.12444402	0.028382769
153.3940322	297.5224695	277.5516027	7.343443858	0.680472468	0.028385394
945.4081168	1340.167584	2560.413535	9.738886282	1.278086915	0.028409063
141.9155672	89.52003506	60.7144131	6.848102541	-0.77601603	0.028409459
153.3940322	98.29650908	86.73487586	6.651220579	0.766060379	0.02843725
6321.503721	10332.54287	3482.405266	12.91414769	-0.89489277	0.028487015
79.30575814	232.5765617	45.9694842	6.350824487	1.193702713	0.028491016
918.2771995	705.6285116	2776.383376	10.74071443	1.602713184	0.028492866
1.043496818	0.877647403	0	-8.70511228	9.836742318	0.028527842
1595.506634	0.877647403	2.602046276	3.037600998	7.707055339	0.028539137
2.086993635	1.755294805	0	-0.21453697	3.212687557	0.028540305
106949.0323	20676.49516	24526.02085	15.82078738	-1.11057745	0.028555414
726.2737851	175.5294805	546.4297179	7.782671768	1.177020268	0.028579713
210.7863572	348.4260188	194.2861219	7.500765644	0.642452605	0.02859108
63.65330588	123.7482838	86.73487586	6.199972673	0.727648706	0.028643398
22.95692999	18.43059545	23.41841648	5.252330246	-0.8642703	0.028654034
735.6652565	2065.981986	809.2363917	9.910269567	0.958134059	0.028657251
296.3530962	277.3365792	242.8576524	8.677522447	-0.74963221	0.028659982
180.5249495	202.73655	183.8779368	7.939583227	-0.50934437	0.028685832
210.7863572	288.7459954	249.7964425	7.891899495	0.454201217	0.028695437

72.00128042	67.57884999	63.31645938	5.748071016	1.292105193	0.028697512
837.9279446	548.5296266	526.4806964	9.559046279	-0.56524641	0.028698888
487.3130138	496.7484298	184.7452856	8.735547863	1.282760982	0.028740579
4.173987271	1.755294805	13.87758014	3.500688798	3.757588293	0.028741305
97.04520404	168.5083013	76.32669075	7.048982189	-0.63808793	0.028741698
4.173987271	35.96599055	1.734697517	3.364434898	2.620255588	0.028774487
124.1761213	227.3106772	173.4697517	6.940539964	0.667977982	0.028814377
0	0	0	-8.70511228	-7.04068962	0.02885273
0	0	0	-8.70511228	-7.04068962	0.02885273
0	0	0	-8.70511228	-7.04068962	0.02885273
0	0	0	-3.0540396	-4.09031279	0.02885273
0	0	0	-8.70511228	-7.04068962	0.02885273
0	0	0	-8.70511228	-7.04068962	0.02885273
0	0	0	-8.70511228	-7.04068962	0.02885273
0	0	0	-8.70511228	12.04810192	0.028878271
0	1.755294805	0	-0.09469958	-2.52749593	0.028883888
3.130490453	1.764071279	1.734697517	0.290303566	2.488565182	0.02889098
19.82643954	9.654121428	6.938790069	4.202775934	-1.19789123	0.028893302
125.2196181	100.9294513	117.9594312	7.039179972	1.420501092	0.028900417
252.5262299	202.73655	277.5516027	8.092673196	-0.46966107	0.028913917
236.5294237	107.8628658	455.158608	7.576109229	0.848739244	0.028936891
376.7023512	96.54121428	128.3676163	8.378791865	-0.80117672	0.028937791
3.130490453	4.388237013	0	1.102608218	2.824335959	0.028949067
0	0	0.884695734	-8.70511228	8.720187212	0.028980729
34.43539498	103.5623935	111.8879899	5.441877496	0.885190396	0.028994468
0	0	2.26378026	-1.45157153	3.402374713	0.029023713
0	0.877647403	1.734697517	2.080555851	-2.20158167	0.029049726
2.086993635	2627.676323	712.9606795	6.901531744	6.599039064	0.029055545
1092.541168	1295.407566	1466.686751	9.84533519	0.60304317	0.029060498
0	0	0	-1.10463874	-2.66334124	0.029063027
225.3953126	2.632942208	715.5627258	3.166690806	5.970184781	0.029069647
1807.336488	588.9014071	1176.992265	9.710593577	0.727416896	0.029089478
0	0	0	-0.84693625	-2.68186052	0.029158249
1.043496818	0	0	-0.70641516	-2.38468214	0.0291606
410.0942493	207.124787	715.5627258	9.701717997	2.047182027	0.029179157
590.6191988	416.8825162	572.4501807	9.033567659	0.577389918	0.029181272
915.1467091	791.6379571	471.8377247	10.11261381	-0.73461983	0.029218619

11.47846499	71.96708701	0.867348759	3.903784629	2.62760913	0.029245256
0	10.53176883	0	-8.70511228	10.52377473	0.029245381
9419.645773	6479.670773	364.2864786	12.42089605	-1.24042694	0.029280322
182.6119431	56.16943376	359.082386	9.029855	2.397213543	0.0292955
121.0456308	9.654121428	130.9696625	6.735409547	-1.12275475	0.029302491
595.8366829	621.374361	909.8488477	8.995735038	0.486854185	0.029310473
698.099371	1021.581577	354.7456423	9.736174111	-0.76235069	0.02933967
45.91385998	3.51058961	16.47962641	3.394660646	1.801787534	0.029413457
2.086993635	0.877647403	0.867348759	1.667372461	-1.82249075	0.02944529
282.7876376	208.0024344	198.6228657	8.277635596	-0.51153588	0.029479792
15.65245226	277.3365792	2.602046276	7.586428279	-1.75120445	0.029491708
87.65373268	510.7907883	65.91850565	7.684721578	1.828760732	0.029496742
0	0	0.867348759	-8.70511228	7.686218529	0.029520652
41.73987271	72.84473441	75.459342	5.523254909	0.759489291	0.029531836
574.9667465	855.7062174	456.225447	9.517545344	1.038047449	0.02954901
24.00042681	12.28706364	13.01023138	5.031537172	-0.79234737	0.029569238
16.69594908	2.632942208	8.673487586	3.164902081	1.857358739	0.029570833
70.9577836	40.37178052	53.77562303	6.005263627	-0.68906221	0.029591824
4574.690049	2477.598617	1714.748496	11.85640641	-0.6700442	0.029601961
519.6614152	537.9978577	299.2353217	8.991261099	-0.80484496	0.029605542
4806.711566	2615.301495	3364.402467	12.26390821	-0.60818415	0.029647382
82.4362486	63.19061298	69.38790069	6.629643822	-0.7675886	0.029652199
7.304477724	33.3506013	37.29599662	3.727903474	1.372229909	0.029665998
91.55641078	232.5765617	305.306763	6.851844182	1.208364407	0.02966869
0	0.877647403	1.734697517	2.961706211	-2.50170834	0.029674676
161.7420067	127.2588734	128.3676163	6.661239096	0.895986807	0.029678646
1.043496818	0	0	-8.70511228	7.31514439	0.029700237
411.1377462	443.2119383	295.7659267	8.151110518	0.693568274	0.029707271
109.5671659	243.1083305	237.6535598	7.284230495	0.731596141	0.029711959
0	0	0	-8.70511228	10.3759854	0.029738859
13.06458016	555.998406	154.06716	6.973321687	2.83782035	0.029776839
172.1769749	180.7953649	61.58176186	6.588198709	0.927621111	0.029785769
56.34882815	53.53649155	38.16334538	5.633698205	-0.76218814	0.02987846
52.17484088	37.73883831	46.83683296	5.525037405	-0.62550386	0.02988758
1089.410678	2370.525634	2125.004458	10.51544227	0.529517952	0.02990597
104.3496818	83.37650324	76.32669075	6.931353076	-0.55019974	0.029907821
2306.127967	2065.981986	2952.455174	11.52121936	-0.60620224	0.029933295

105.3931786	137.7906422	173.4697517	6.887893559	0.838529846	0.029945307
31.30490453	2.632942208	25.153114	3.783289517	1.89755266	0.029946231
204.5253763	248.3742149	301.837368	7.191989527	0.82708508	0.030044477
277.5701535	186.9388967	221.1739334	7.522863519	0.816660177	0.030060562
1289.762067	664.3790837	1983.626611	11.06641514	-1.13766853	0.030084903
584.3582179	272.0706948	290.5618341	9.765208429	-0.72661404	0.030086009
5.217484088	0.877647403	6.07144131	2.468041924	-1.99494342	0.030097426
53.2183377	61.43531818	162.1942179	6.972718558	1.40889015	0.030127104
368.3543766	372.1224987	918.5223353	8.666175962	0.643740263	0.030181699
0	0	0	-0.2646055	-2.43995629	0.030182549
6.260980906	164.9977117	1.734697517	1.692454643	4.962434992	0.030217242
265.0481917	324.7295389	346.0721547	8.390711886	-0.93797391	0.03021818
74.08827405	129.8918156	248.0617449	6.856797509	1.164091281	0.030228597
1238.630723	1097.059253	1241.176074	10.36896817	-1.11798082	0.030248808
0	0	0	-8.70511228	10.9963223	0.030259848
12.52196181	10.53176883	3.469395034	2.205786843	2.855278921	0.030311726
3.130490453	2.632942208	1.734697517	-0.35979729	2.922166929	0.030339484
63.65330588	34.2282487	170.0003567	5.537115208	1.010058963	0.030346827
49.04435043	100.0518039	109.2859436	6.074745528	0.806168783	0.030377588
513.4004343	160.6094747	329.5925283	8.847189295	-0.64736581	0.030447501
552.0098165	293.1342324	483.9806073	8.422674226	0.58275441	0.030457102
882.7983077	278.2142266	279.2863003	9.293891736	-0.97867138	0.030457262
1459.852048	705.6285116	541.2256253	10.27639321	-0.58691945	0.030519409
3.130490453	2.632942208	7.806138827	1.133137529	4.051724068	0.030529173
120.002134	245.7412727	226.378026	7.131665533	0.586202484	0.030554404
22.95692999	160.6094747	42.50008917	6.464781979	1.667778693	0.030591068
2463.695987	4533.926481	5251.796733	11.36093957	0.867992472	0.030608315
48.00085361	104.4400409	474.4397709	5.744295151	1.74097128	0.030608807
14.60895545	53.53649155	101.4798048	4.368264795	1.738927736	0.030662245
0	0	0	0.367736943	-2.19568246	0.030667202
8302.060681	4979.771362	1646.227944	12.44187532	-0.76554088	0.0306759
300.5270835	5.265884415	6.938790069	8.965274679	-2.44967328	0.030685377
11.47846499	18.43059545	12.14288262	2.928648155	1.231859649	0.030709228
5.217484088	1.755294805	1.734697517	2.860051175	3.422574393	0.030728586
286.9616249	250.1295097	249.7964425	7.47508442	0.564895836	0.0307407
0	16.67530065	3.469395034	-8.70511228	9.796849293	0.030807086
1.596550131	0	1.170920824	-8.70511228	7.708255997	0.030829318

52289.62553	119367.0679	40068.04325	16.9417989	-1.41006381	0.030847727
19716.87237	12185.25654	5861.54291	14.01932859	-0.90140324	0.03087034
883.8418046	512.5460831	922.8590791	8.720261493	0.822958645	0.0308858
22.95692999	42.12707532	28.62250903	4.395066677	1.168125203	0.03088773
124.1761213	278.2142266	238.5209086	6.894397474	0.878261041	0.030896233
746.1002246	346.670724	206.4290045	9.376227938	-0.72343086	0.030913943
979.8435118	411.6166318	380.766105	10.08170527	-0.6112843	0.030925793
2.086993635	0.877647403	0.867348759	2.519257489	-1.98503139	0.030928075
149.2200449	66.70120259	73.72464448	6.36411034	0.89429014	0.030932536
8.347974541	80.74356103	29.48985779	5.008361497	1.231699834	0.030975265
87.65373268	114.9718097	161.3268691	6.421488857	0.623350129	0.0309833
0	2.413530357	0	1.698638249	10.32675038	0.031086548
324.5275103	229.0659721	172.602403	8.152447671	-0.6691114	0.031119053
8.347974541	4.388237013	29.48985779	2.520592292	2.182846979	0.03112027
1.043496818	2.632942208	0	2.916824674	-2.16762643	0.03112541
1785.423055	1623.647695	1576.840043	11.36687014	-0.47213739	0.031153066
5.217484088	11.40941623	7.806138827	2.618888025	1.38311007	0.031197477
212.8733508	295.7671746	207.2963533	7.901973753	0.559631904	0.031218015
0	0	0	-0.80190004	-2.59178812	0.031265163
357.5437496	740.5939841	510.3219891	8.884195396	0.737883598	0.031271932
15.65245226	28.96236428	28.62250903	5.530218117	2.455773439	0.031298623
76.17526769	59.68002337	101.4798048	5.52289739	0.803272364	0.0313301
598.3828151	381.6800789	507.7112693	9.209018007	-0.4642502	0.031414273
1023.670378	472.1743025	937.604008	9.082566704	0.527941214	0.031424846
57.39232497	91.27532986	91.07161965	6.475980144	0.703948412	0.03142785
1.043496818	8.776474025	7.806138827	2.269785301	6.181332105	0.031429605
51.13134407	17.55294805	19.08167269	5.842929832	-1.02816573	0.031443415
88.6972295	358.9577876	194.2861219	7.076152617	0.671780377	0.031452788
4.173987271	0	0	0.247130611	-2.37677578	0.031456901
0	1.755294805	0	-8.70511228	8.011721097	0.031487138
28.17441408	27.20706948	21.68371896	5.116975603	-0.87462691	0.031489223
0	0	0	0.27797041	-2.67530197	0.031493303
4.173987271	16.67530065	1509.18684	2.967995548	7.633315113	0.031537486
516.5309247	1872.899557	1213.420913	9.245912318	0.804205886	0.031569321
45.91385998	42.12707532	83.26548082	5.542614406	0.921632566	0.0316028
177.394459	78.11061882	264.5413714	6.957660328	1.237926645	0.031625336
512.3569375	1066.341594	556.837903	9.236648508	0.777129032	0.031705478

191.8781948	87.76474025	294.5429649	8.15808519	-0.69528136	0.031733663
3.130490453	14.04235844	5.204092551	4.09991458	-1.81042264	0.031799188
154.437529	1061.07571	123.1635237	7.735214305	1.880552606	0.031803089
0	0	0	-1.17578199	-2.68716098	0.031809599
131.480599	233.4542091	333.929272	7.720195462	0.612133977	0.031903783
5.217484088	7.898826623	1.734697517	4.281455809	-1.60951086	0.031928005
1527.679341	873.2591655	1414.645825	10.12974814	-0.5332043	0.031933625
291.1356121	973.3109694	741.5831886	9.420542072	1.279758167	0.0319738
5.217484088	15.79765325	39.03069414	4.359360782	2.674021679	0.032001974
264.0046949	560.8166902	364.2864786	8.314646756	0.620580872	0.032006694
13.56545863	49.14825454	27.75516027	5.063337655	1.624664774	0.032029536
679.3164283	528.3437363	415.4600554	9.590869257	-0.81816203	0.032052254
120.002134	337.0166026	160.4595203	6.996789274	0.833832232	0.032093715
229.5692999	835.5203272	532.5521378	8.656300769	0.794034413	0.032126694
0	0.877647403	0	-8.70511228	7.498272895	0.032195631
46.95735679	182.5506597	60.7144131	6.112122104	0.853747784	0.032228817
427.8336952	1.755294805	181.2758905	8.058633804	-1.89886857	0.032294507
833.7539573	631.9061298	797.9608579	9.664593777	-0.54589386	0.032301576
0	194.8377234	8.673487586	2.51676643	4.970201495	0.032312765
2.086993635	5.265884415	0	1.332258823	2.296370988	0.032324225
0	0	0	-1.1764867	-2.40804196	0.032377557
2.086993635	1.755294805	0.867348759	-0.3534158	2.934929898	0.032388402
399.6592812	279.091874	383.3681513	8.828053192	-0.47526676	0.032462437
1.043496818	5.265884415	0.867348759	0.452261178	2.748399766	0.032485676
74.08827405	65.82355519	32.09190407	6.356777169	-0.76964028	0.032491743
33.39189817	148.322411	116.2247336	5.740280866	0.874468604	0.032516418
525.9223961	204.4918448	168.2656592	8.302093839	-0.72519437	0.032542214
728.3607787	1738.619504	1450.207124	9.818939993	0.601561586	0.032562145
30.26140771	32.47295389	88.46957337	5.132432802	1.000625342	0.032571409
763.8396705	108.8282779	0.867348759	8.943445721	-2.08469152	0.032585337
0	0	0	-8.70511228	8.005786376	0.032588944
0	15.79765325	5.204092551	-8.70511228	8.679524313	0.03263588
291.1356121	344.9154292	313.9802506	7.928994015	0.559614995	0.032643504
27.13091726	15.79765325	6.938790069	4.936812046	-1.15012319	0.03265526
4636.256361	3833.563854	1948.93266	11.71283431	-0.95605546	0.03267348
99.13219768	115.8494571	91.07161965	6.981967486	-0.64378211	0.03271976
11.47846499	8.776474025	8.673487586	4.511530753	-1.03366808	0.032757673

1.043496818	8.776474025	0.867348759	-8.70511228	7.903038565	0.032781872
7.304477724	2.632942208	2.602046276	1.235505985	2.269589321	0.032816476
0	0	0	-8.70511228	7.284592149	0.032865974
255.6567203	449.3554701	345.2048059	7.795775132	0.616513605	0.032880144
1.043496818	0.877647403	0.867348759	-0.16241571	3.316930087	0.032894158
0	0	0	-8.70511228	7.288737412	0.032895498
77.21876451	17.55294805	58.11236682	6.65464753	-1.0741554	0.032900674
2.086993635	5.265884415	4.336743793	1.467354671	2.011770314	0.032900961
39.65287907	14.04235844	113.6226874	4.016348659	2.732588583	0.032904417
344.3539498	1439.34174	842.1956446	9.361761697	0.754214572	0.032908321
96.00170723	198.348313	90.20427089	5.839557703	1.045935742	0.032917617
1214.630296	607.3320025	396.3783827	9.556872767	-0.54589486	0.032929261
460.1820966	400.2072155	407.6539165	8.392048064	0.544566512	0.032934924
214.9603444	190.4494863	178.6738443	7.530657743	0.600720859	0.032943681
178.4379558	211.513024	94.54101468	6.900920449	0.617522278	0.032957558
12.52196181	7.898826623	6.938790069	2.84362344	2.045287811	0.032982057
281.7441408	322.0965967	202.9596095	7.991184008	0.606270941	0.0329906
2.086993635	3.51058961	4.336743793	0.768108915	2.014899883	0.033031827
663.663976	2269.596183	1282.808814	9.819671689	0.737662899	0.033040415
574.9667465	1442.85233	1569.033904	9.655039981	0.737112992	0.033046865
1520.374863	2206.40557	2046.075721	10.58365637	0.599102766	0.033055109
135.6545863	294.8895272	177.8064955	7.289638623	0.71225894	0.033057753
510.2699438	537.1202103	914.1855915	8.678584104	0.594770952	0.03308308
2.086993635	0	0	-8.70511228	7.483981834	0.033084663
49.04435043	59.68002337	89.33692213	5.263856721	1.018785406	0.033126962
1758.292138	1691.226545	568.1134369	10.63534923	-0.7036857	0.033127473
25.04392362	35.1058961	26.88781152	4.837665696	0.771977049	0.033140083
0	7.02117922	1.734697517	3.158848049	-1.67059497	0.033164698
0	0.877647403	6.07144131	-8.70511228	7.756807461	0.033182135
291.1356121	639.8049564	535.154184	9.432885494	-1.05981422	0.033203117
879.6678173	783.7391304	454.4907495	10.06895288	-0.88249649	0.03321299
0	0	0.867348759	-8.70511228	7.331614213	0.033217775
0	0	0.867348759	-0.80429161	-2.49630604	0.033237321
1.043496818	2.632942208	7.806138827	2.552461529	-1.43178006	0.033254315
8023.447031	1049.666293	2459.801079	12.05165484	-0.87927186	0.03326868
0	0.877647403	0.867348759	1.587931044	-1.79043423	0.033276256
155.4810258	109.7059253	147.449289	7.019112364	0.699399889	0.033292483

2.086993635	0	0	-8.70511228	8.39805583	0.033294716
0	0	0	7.669735202	-3.04328414	0.033319427
1.043496818	0	0	-8.70511228	7.403892316	0.033319442
96.00170723	153.5882954	199.4902145	7.056310441	1.043225345	0.033329931
6852.424467	12248.96496	10470.81636	13.03251213	0.685242531	0.033406336
0	0.877647403	1.778064955	1.610615263	-1.43932615	0.033415767
126.2631149	283.480111	502.1949312	7.558963559	0.836044425	0.033475282
0	0	0	-1.02355611	-2.38270923	0.033494297
716.8823137	1780.74658	650.5115689	9.47130446	0.641579992	0.033495296
0	0	0.867348759	4.820228763	-2.11539304	0.033524551
0	7.898826623	0	0.043622697	4.023914029	0.033538382
120.002134	127.2588734	106.6838973	6.786910259	0.619369703	0.033633708
49.04435043	43.00472272	322.6537382	5.338122239	1.310341962	0.033639979
45.91385998	21.94118506	63.31645938	5.045340727	1.270944791	0.033744143
5.217484088	0.877647403	42.50008917	2.24318265	2.17482116	0.033767714
297.396593	277.3365792	214.2351434	8.333044705	-0.62956353	0.03377672
0	0	0	2.409285693	6.662448797	0.033787622
1.043496818	0.877647403	0	-8.70511228	10.26730425	0.033790959
60.52281542	140.4235844	26.88781152	6.707696308	-0.7910955	0.033809363
145.0460577	20.18589026	65.05115689	6.507080092	-1.46166794	0.033829804
379.8328416	4.388237013	187.3473319	7.837776215	-1.6429295	0.033836085
193.0469113	245.7412727	219.4392359	7.447126792	0.647392164	0.033842418
0	0	0	0.104338273	-2.15699626	0.033866689
1107.150124	851.3179804	922.8590791	9.882147979	-0.65539622	0.033917313
0	0.886423877	0	-8.70511228	7.187027165	0.03394227
0	0	0.867348759	-8.70511228	7.167519679	0.03394227
200.351389	222.0447928	360.8170836	7.168298729	0.704241744	0.033965486
8.347974541	0	0.867348759	-8.70511228	11.79388366	0.033973561
1478.634991	2137.949073	1453.676519	10.44895524	0.545198031	0.03397407
224.3518158	785.4944252	78.06138827	8.290060734	1.578867078	0.033980127
346.4409435	1788.645406	849.1344346	9.076944274	1.028921793	0.033993799
0	0.877647403	1.734697517	-8.70511228	7.175748205	0.033998843
1214.630296	946.9815473	946.2774956	10.15448775	-0.61858699	0.034051479
922.4511868	628.3955402	408.5212653	9.618571004	-0.8415146	0.034053451
2.086993635	18.43059545	5.204092551	1.772615111	2.565232315	0.03405856
219.1760716	364.0656955	343.8517418	7.972911157	0.459455851	0.034070135
6.260980906	0	19.94902145	1.622272391	2.317528142	0.034075297

460.1820966	469.5413603	747.6546299	8.958643291	0.887378257	0.034094203
17.7394459	30.71765909	27.75516027	4.420295548	0.798509311	0.034096316
221.2213253	344.9154292	289.6944854	8.09820476	0.661963434	0.03413563
13334.84583	4040.688641	7327.362312	12.98500787	-0.74760483	0.034219156
156.5245226	257.1506889	210.7657483	7.6465316	0.743066069	0.034230081
174.2222287	193.108758	20.87708462	5.716895636	2.089929056	0.03425622
258.7872108	383.5319149	245.4596987	8.666958542	-0.5527453	0.034298123
0	1.755294805	6.07144131	1.757501888	-1.62986289	0.034307475
69.91428678	57.04708116	85.8675271	6.534240068	-0.54757082	0.034308223
1671.681902	367.7342617	921.9917304	11.04106982	-0.92917781	0.034321982
236.8737776	351.058961	437.1437743	8.228953045	0.697355532	0.034360288
0	0	0.867348759	1.415925545	6.585828607	0.034363084
114.7846499	191.3271337	246.3270474	6.781773306	0.739982534	0.034381077
0	0	0	0.154023179	-2.03631836	0.034411025
25.04392362	97.41886168	113.6226874	6.285862318	1.610895092	0.034411974
1041.409824	261.5389259	132.7043601	9.202960431	-1.04680208	0.034415205
41.73987271	71.96708701	13.87758014	6.049212302	-1.28592699	0.034419827
24.00042681	0	11.27553386	4.127676639	-1.53909753	0.03452819
3590.67255	1272.588734	1695.666823	12.03821182	-1.25535876	0.034546223
52.17484088	33.3506013	58.97971558	5.202254765	0.883807631	0.034575907
944.36462	504.6472564	363.4191298	10.21716295	-0.95227828	0.034585898
1.043496818	0	54.64297179	2.624430648	3.883730274	0.03461537
782.6226132	267.6824578	353.0109447	9.278803777	-0.63352124	0.034633412
366.267383	336.1389552	377.29671	8.427515101	0.58189566	0.034665444
281.7441408	441.4566435	215.9698409	8.140821657	0.612347714	0.034675928
164.8724972	250.1295097	179.541193	7.236631377	0.568169151	0.03469019
0	11.32165149	4.753071197	1.469401536	2.580564574	0.03474152
824.362486	1517.443582	1026.064908	9.663573781	1.05437246	0.034750067
0	0.877647403	0	-8.70511228	8.727560307	0.03479342
506.0959566	478.3178344	587.1951095	9.075633698	0.455937831	0.034798846
160.6985099	65.82355519	140.5104989	7.505721584	-0.92407206	0.034811619
339.1364657	1155.861629	378.1640587	8.687088992	0.739106145	0.03486082
1.043496818	5.265884415	19.94902145	2.359618214	1.500079466	0.034925571
780.5356196	1026.847461	1521.329723	9.933790689	0.885376545	0.034949853
0	34.2282487	0	-8.70511228	13.1551188	0.0349692
135.6545863	181.6730123	213.3677946	7.122824077	0.484110978	0.034999075
382.9633321	251.8848045	161.3268691	8.804994533	-0.64097324	0.035021255

331.831988	667.0120259	550.7664617	8.701086184	0.658617441	0.035145337
73.04477724	86.00944545	88.46957337	6.222554597	0.523512463	0.035164263
7.304477724	3.51058961	9.540836344	3.72981659	-1.14199673	0.035206571
10.43496818	5.265884415	32.95925283	3.13512992	1.393009007	0.035214049
0	0.877647403	2.602046276	-8.70511228	7.20386451	0.035238463
210.7863572	337.0166026	216.8371896	7.876939349	-0.59106429	0.035256353
1517.244373	717.0379278	662.6544515	10.45356845	-0.53499876	0.035258138
0	0	0	-8.70511228	10.17348221	0.035271161
10.43496818	21.06353766	30.35720655	3.534999915	1.381879244	0.035285904
51.13134407	46.51531233	59.84706434	6.396247316	-0.68489994	0.035293506
37.56588544	8.776474025	29.48985779	5.115744112	-0.81981127	0.035346304
3.130490453	1.755294805	17.34697517	0.946926885	2.440013961	0.035367123
0	0	0.867348759	-0.15359223	-2.12441485	0.035370874
78.26226132	50.90354935	78.06138827	6.07904718	0.854034733	0.035371912
1.116541595	15.00777058	12.73267978	3.12103524	1.73049923	0.035377705
220.1778285	110.5835727	261.0719763	7.949736826	0.834855691	0.035378795
60.43933568	64.15602512	66.9333037	6.262741945	0.784463508	0.035383368
467.4865743	662.6237889	801.4302529	9.057387138	0.651756682	0.035385359
31.30490453	129.0141682	58.97971558	6.273591716	0.859388932	0.035422255
7.304477724	13.16471104	32.95925283	4.52343029	1.553563035	0.035448866
487.3130138	667.8721204	664.3891491	8.954239083	0.642629242	0.035459015
137.7415799	257.1506889	189.9493781	6.860820091	0.721941447	0.035476679
352.2219158	764.7380642	524.6245701	8.869491065	0.682408523	0.035492377
162.7855036	313.3201227	124.0308725	7.54505449	-0.72456607	0.035554255
14.60895545	19.30824286	29.48985779	3.938810147	1.196227473	0.035590152
112.6976563	426.5366376	284.4903928	7.224193073	0.812326549	0.035631487
85.56673905	287.868348	130.9696625	7.410174752	0.891392313	0.035634101
172.1769749	808.3132577	385.1028488	8.67249041	1.109468841	0.035634378
579.1407338	900.466235	395.5110339	8.743118082	0.661164697	0.03564158
427.8336952	1116.367496	1447.605078	9.591692457	0.705131785	0.035674027
215.1273039	218.5078738	103.032359	8.257588046	-0.69392455	0.035678524
0	0	0	-1.18000081	-2.57976389	0.035693352
1812.553972	987.3533278	1328.778298	10.74746442	-0.7855466	0.035700211
640.707046	382.6542675	526.4806964	9.970373184	-1.31092544	0.035766906
5465.836331	32658.13749	29715.36847	14.12434483	1.176083217	0.03580362
83.47974541	48.27060714	75.459342	7.093185654	-0.85984786	0.03580442
114.7846499	165.8753591	130.1023138	7.067574641	0.968297959	0.035807797

1.043496818	0	0	-1.17183941	-2.6309679	0.03582206
26.39003452	47.28764205	90.25631182	5.555311066	0.894464187	0.035831085
76.27961737	185.3064726	176.730983	6.420584981	0.740523809	0.035848819
0	2.632942208	0.867348759	1.491492728	2.614838798	0.035851419
2.086993635	1.755294805	5.204092551	1.198600971	4.038963445	0.035876117
339.1364657	374.7554409	261.0719763	8.302258764	0.55776428	0.035885843
0	3.51058961	0.867348759	2.18891178	-2.08975676	0.035892068
37.56588544	66.70120259	104.081851	5.192469725	0.803331306	0.035938487
21.91343317	16.67530065	27.75516027	4.815101449	1.090828371	0.035998258
0	1.755294805	0.867348759	0.074646789	-2.1761153	0.036005356
1860.554826	2135.31613	1190.869846	10.01692716	0.661134062	0.036027793
24.00042681	19.30824286	11.27553386	5.124429113	-0.76765438	0.036040879
419.4857207	796.0261941	411.1233116	8.643939181	0.59368577	0.036102368
0	0	1.734697517	-8.70511228	7.005106366	0.036111687
99.03828296	304.087272	454.6468723	7.081095862	1.360447696	0.036144843
355.8324148	346.670724	424.1335429	8.229459211	0.44588581	0.036150058
0	1.755294805	0.867348759	-8.70511228	7.058592455	0.036158342
31.30490453	2.632942208	91.07161965	7.296643898	-1.29768986	0.036162703
0	0	0	-8.70511228	7.816015732	0.036181002
268.1786821	2.632942208	3.469395034	8.600092791	-2.19591748	0.036181861
2214.300247	1175.169872	815.3078331	10.82112948	-0.73226642	0.036184877
214.9603444	361.5907298	366.0211761	7.582284763	0.955203394	0.036210005
72.00128042	108.8282779	149.1839865	6.556755025	0.698889446	0.036218505
68.87078997	293.1342324	268.0107664	6.22180303	1.215849083	0.036228666
2.086993635	0.877647403	1.734697517	0.269785093	2.236894205	0.036236919
75.13177087	18.43059545	13.87758014	5.173646941	1.763246521	0.036251723
0	0	1.734697517	-8.70511228	7.197682311	0.03626298
0	0	0	-1.31105034	-2.94750956	0.036263673
835.8409509	1301.551098	1340.921181	9.849306749	0.45146399	0.036265595
775.3181355	582.7578753	454.4907495	9.793137826	-0.70769385	0.036295304
250.4392362	835.5203272	595.0012484	8.583014216	0.717888112	0.036314221
127.3066118	126.381226	104.081851	6.695458263	0.634825867	0.036326521
18.99164208	3.659789668	6.661238466	2.357440787	1.607697775	0.03635471
1338.806417	1147.085155	942.8081006	10.35476153	-0.53050308	0.036369103
2182.995343	842.5415064	2265.514957	10.48522465	0.872643807	0.036409327
448.7036316	714.4049856	2061.687999	9.402005346	0.742141688	0.036416692
14.60895545	28.96236428	22.55106772	4.435195774	1.091087703	0.036457964

154.437529	261.5389259	300.1026705	7.295534715	0.682272932	0.03649388
5.113134407	5.827578753	9.922469798	1.915382968	1.579184188	0.036504076
242.0912617	460.7648863	184.7452856	7.598989883	0.773959418	0.036586223
1.043496818	0	6.07144131	0.422228812	-2.39869882	0.036593721
6.260980906	12.28706364	4.336743793	2.569824562	1.52420996	0.036630591
20.86993635	20.18589026	41.63274041	4.482216573	0.988438578	0.036668064
1.043496818	0.877647403	0.867348759	-8.70511228	7.253051401	0.036680018
4.173987271	74.60002921	56.37766931	3.852441504	1.827939423	0.036699917
265.0481917	688.953211	1082.451251	8.395510745	0.979172359	0.036700199
0	0.877647403	1.734697517	-8.70511228	7.209988148	0.036736279
0	12.28706364	0	2.188888105	3.536225741	0.036774145
22.95692999	9.654121428	12.14288262	4.120871964	-1.18460208	0.036785586
1657.072946	444.0895857	2220.412822	9.751637665	1.025292695	0.0367948
0	0.877647403	3.469395034	-8.70511228	7.827481787	0.036859594
0	2.632942208	6.938790069	3.007464546	-1.42638914	0.036882157
0	0	478.7765147	-8.70511228	14.1562546	0.036904124
100.1756945	251.0071571	204.694307	7.150703468	0.681214088	0.036916004
0	3.51058961	0	2.957625339	3.989861542	0.03692888
235.0789631	76.88191246	108.8869632	7.941767901	-0.60845839	0.036998859
57.1523207	98.40182677	102.2690921	6.203559094	1.081263758	0.037001928
160.6985099	101.8070987	42.50008917	7.13368558	-0.63282949	0.037018995
328.7014976	482.7060714	376.4293612	8.272061065	0.597361805	0.037032324
2.086993635	9.654121428	4.336743793	0.510825341	4.775628198	0.037058641
393.3983003	122.8706364	109.2859436	8.027872382	-1.70116743	0.037068958
20.86993635	8.776474025	32.09190407	3.997254677	1.272321858	0.03708102
37.56588544	32.604601	58.84961327	6.172312071	-0.71159012	0.037100727
457.0516061	631.9061298	495.2561411	8.730721814	0.486477774	0.037104757
100.1756945	86.88709285	112.7553386	6.062174952	0.799352259	0.037106261
1.043496818	3.51058961	6.07144131	-0.3527502	2.809188467	0.037137103
124.1761213	286.1130532	181.2758905	7.225495076	0.665728926	0.037146451
15.65245226	34.2282487	5.204092551	2.669449831	2.393491765	0.037193817
115.8281468	116.7271045	97.14306096	7.218653038	-0.69936064	0.037243225
1080.019206	264.1718682	1686.125987	9.048164519	1.276994975	0.037251505
7.304477724	35.1058961	215.1024921	3.909207972	2.482674036	0.037271316
11.47846499	8.776474025	6.07144131	6.824178438	-2.02081684	0.037302144
4.173987271	2.632942208	19.94902145	1.660303154	1.887435094	0.037307107
0	0	0	1.282399689	-2.06117768	0.037308391

105.3931786	67.57884999	67.65320317	6.837349676	-0.56149416	0.03737689
0	0	0	2.01389768	-2.64528588	0.037405443
45.91385998	55.29178636	67.65320317	5.67524125	0.831373893	0.037429161
4.173987271	15.79765325	2.602046276	2.215359418	1.839093675	0.037437492
333.9189817	157.9765325	153.5207303	8.34992154	-0.88030137	0.037459779
11.47846499	2.632942208	1.734697517	3.095459694	-1.359909	0.037480506
155.4810258	208.8800818	111.0206411	7.312974976	0.496878805	0.037486912
31.30490453	441.4566435	83.26548082	6.742145947	1.687147949	0.037495194
0	0	3.469395034	1.761488326	-2.03267059	0.037511142
0	1.755294805	2.602046276	4.039776711	-2.0344326	0.03754373
2.337432872	0.974188617	1.994902145	-8.70511228	7.297792699	0.03754839
123.1326245	133.4024052	219.4392359	6.291100806	1.005315454	0.03755432
0	0	0	2.243152919	8.422974474	0.037564041
942.2776263	607.3320025	1040.81851	10.10854958	-0.66893193	0.037614523
3.130490453	79.86591363	0.867348759	-8.70511228	10.47932542	0.037639705
10.58105773	6.406826038	16.99136218	2.862117258	1.526575354	0.037641781
57.39232497	117.6047519	107.5512461	6.344560714	0.778615615	0.037673257
2024.039472	1038.555277	938.4626833	11.05521682	-0.78187227	0.037705956
5306.181318	6436.66605	10411.6545	12.31485398	1.28668627	0.037715923
0	1.755294805	0	0.797417025	3.292158068	0.037737755
5.217484088	0	6.07144131	2.956160282	-1.50420522	0.037823585
34.43539498	143.934174	279.2863003	6.188877035	1.768972356	0.037832182
914.1032123	952.2474317	865.614061	9.606827512	0.552506077	0.037840519
7.304477724	7.02117922	5.204092551	3.194774242	-1.10502303	0.037845658
8.347974541	14.04235844	11.27553386	3.761147918	1.013725359	0.037858328
1882.468259	890.8121135	2222.147519	10.20797767	0.733957119	0.037879956
6.260980906	1.755294805	0.867348759	2.749856596	-1.50612782	0.037883003
0	0.877647403	0	-8.70511228	9.523214986	0.037895836
50.94351464	122.8706364	85.55528154	5.945745119	1.243775841	0.037923023
240.0042681	152.710648	77.19403951	7.774378174	-0.58877368	0.037951509
24.00042681	15.79765325	39.03069414	4.559737888	1.18087582	0.037962008
2504.392362	4158.293393	3885.722438	11.21698969	0.665710232	0.037991706
1.043496818	0	0	-0.16403345	-2.32233098	0.038005178
84.52324223	157.9765325	111.0206411	6.432441507	0.667640332	0.038077913
14.60895545	0	0.867348759	3.81541996	-2.14176804	0.038097674
323.4840135	165.8753591	293.1638804	7.91154428	0.725303489	0.038105635
3835.894302	5049.983154	4271.692636	12.03865508	0.771364377	0.038109413

0	0	0	-8.70511228	8.630273105	0.038170901
610.4456383	466.9084181	642.7054301	8.714926056	0.48346953	0.038178934
0	0	0	-1.10430989	-2.51129577	0.038186099
19.82643954	4.388237013	6.938790069	4.183595926	-1.0098328	0.038228626
75.13177087	89.52003506	68.52055193	6.202153667	0.773539549	0.038241483
84.52324223	245.7412727	65.05115689	6.384185146	0.863557238	0.038269341
992.3654736	433.5578168	296.6332754	9.81653201	-0.83916394	0.038293692
21.91343317	24.57412727	25.153114	5.115305513	0.964946581	0.038306157
850.4499064	978.5768538	1326.176252	10.03504021	0.476290241	0.038333088
7.304477724	5.265884415	18.21432393	3.039955817	-1.30073308	0.038349655
285.918128	319.4636545	173.4697517	8.070490388	-0.8944949	0.038360279
20.86993635	38.61648571	23.41841648	4.739447439	1.161353592	0.038459453
29.15530109	116.218069	19.61075543	7.335434023	-1.42506404	0.038535129
137.7415799	215.0236136	126.6329188	6.56490366	0.649306217	0.038535411
20.86993635	25.45177467	34.69395034	5.413824294	1.606792317	0.038542923
0	0	0	-8.70511228	10.83073072	0.038590653
128.204019	152.6492127	150.6237854	7.416274382	-0.56175264	0.03863286
12735.87866	5487.05156	845.6650396	13.39279869	-1.07508196	0.038635256
1.043496818	2.632942208	16.47962641	-8.70511228	9.05814288	0.038654961
1364.893838	401.0848629	1965.412287	9.197933276	1.140297786	0.038684685
0	1.755294805	0	-8.70511228	8.831091166	0.038685504
41.73987271	59.68002337	79.79608579	5.543959555	0.720587595	0.038686312
79.30575814	3011.208238	4799.040681	11.46273292	2.383443194	0.038809087
34.43539498	23.69647987	0	-8.70511228	10.09941894	0.038809952
7.304477724	3.51058961	13.01023138	1.597481948	2.245442581	0.038847937
0	0	0	-8.70511228	11.89093658	0.038864606
0	2.632942208	0	0.546005895	-2.55284611	0.038870371
338.0929689	465.1531233	387.7048951	9.108515786	1.37571186	0.038903396
0	6.143531818	0	0.282232635	4.206226773	0.038905412
110.6106627	114.9718097	50.306228	7.161944762	-0.70706473	0.038918195
202.4383826	37.73883831	37.29599662	7.272467025	-1.6370306	0.038954174
44.87036316	42.12707532	59.84706434	6.017774394	-0.630057	0.038969758
460.0881819	705.1019232	807.336898	8.491344163	0.67313293	0.039039357
33.39189817	262.4165733	260.2046276	7.520149601	1.079751691	0.039047517
663.663976	559.9390428	686.9402168	9.249687186	0.599365914	0.039072366
203.4818794	50.02590194	543.8276716	6.456583222	1.417783224	0.039085244
0	1.755294805	2.602046276	2.132164717	-2.25088641	0.039099487

0	13.16471104	10.4081851	1.186751373	4.127480262	0.039181764
15.65245226	48.27060714	66.78585441	5.130997645	1.118260927	0.039195639
54.26183452	36.86119091	65.05115689	6.229621249	-0.73521722	0.03920667
32.34840135	14.04235844	6.07144131	4.832545405	-1.29988039	0.039219183
3013.618809	2448.636253	2373.066203	12.08496129	-0.83927358	0.039280328
90.78422314	28.96236428	88.46957337	7.103667134	1.698591086	0.039283625
52.17484088	46.51531233	43.23733561	5.473333905	0.79777169	0.03929476
1.043496818	0	0	-8.70511228	10.91173783	0.039303253
132.5240958	204.4918448	321.7863894	7.214056638	0.81283569	0.039314352
248.3522426	94.78591947	131.8370113	8.163734182	-0.57873527	0.039317733
200.351389	317.7083597	317.4496456	7.701270203	1.041692038	0.03934475
11436.72512	288.7459954	6270.931524	9.073541569	3.005468618	0.039360619
0	48.27060714	283.6230441	3.019479025	5.792935566	0.039368035
0	4.388237013	0.867348759	3.067816464	4.681793119	0.039403829
1130.107054	168.5083013	78.92873703	8.811600585	-1.39845598	0.039418713
57.39232497	45.63766493	34.69395034	5.112486496	0.80651363	0.039432353
418.4422239	784.6167778	982.7061435	9.080522286	0.628936701	0.039434177
1.043496818	2.852354058	0	3.986552326	6.129232834	0.039437187
161.7420067	728.4473441	477.909166	8.122108197	1.021000291	0.039469051
82.4362486	185.1836019	117.9594312	6.602219487	0.92756199	0.039485088
0	0	0	-8.70511228	12.48286984	0.039549713
216.0038413	68.4564974	73.72464448	6.737795107	1.246716138	0.039559922
0	48.27060714	1.734697517	2.211636959	3.319077359	0.039585509
59.47931861	96.54121428	117.0920824	6.116925677	0.838783453	0.039604755
0	0	0	-8.70511228	8.223729357	0.039630987
0	0.877647403	0	-0.24577765	-2.08718834	0.039643444
38142.93918	42219.2283	36014.9225	15.43092356	-0.82491938	0.039657634
126.2631149	204.4918448	235.9188623	7.104818398	0.869971755	0.039669491
274.6379274	279.0304387	650.2079969	7.838472601	0.641385277	0.039684472
6.761859378	2.474965675	2.732148589	1.587327242	2.740144998	0.039686712
219.1343317	265.0495156	304.4394143	7.417109267	0.791228745	0.039735126
26.08742044	77.23297142	22.55106772	4.680341412	1.249695153	0.03977992
352.4097453	2078.865849	528.5883539	9.299793156	0.976911941	0.039799914
104.3496818	3.51058961	61.58176186	1.531916334	4.578521533	0.039818733
0	0	0	-8.70511228	7.3987525	0.039861437
80.34925496	186.0612493	30.35720655	6.44760486	0.994760935	0.039874178
156.5245226	112.3388675	143.1125452	7.305589502	-0.65765046	0.039898837

45.91385998	93.90827207	135.3064063	6.603801641	-0.64590465	0.039920282
19.82643954	43.00472272	33.82660158	4.770889906	0.898320497	0.039939541
1510.983392	2869.907006	1344.390576	10.38850806	1.028596973	0.039956266
1661.246934	358.0801402	3347.966208	9.484439823	1.138386644	0.039958823
638.6200524	930.3062467	543.8276716	9.407068311	0.434230854	0.039969455
3.130490453	4.388237013	0	2.324092224	-2.01897457	0.039982184
296.3530962	596.8002337	281.8883465	8.134045075	0.621881909	0.039999067
0	0	0	2.389556388	-1.78774206	0.040005413
94.95821041	129.0141682	171.7350542	6.795107639	0.805808874	0.040006272
66989.3652	15311.43658	24498.26569	16.90514304	-1.40968164	0.040018906
13.56545863	37.73883831	13.87758014	4.615707167	1.123969927	0.040022502
219.1343317	645.0708408	330.459877	8.264853908	0.718838522	0.040050696
0	25.45177467	5.204092551	2.783502361	1.933823106	0.040065366
118.9586372	260.6612785	221.1739334	7.49052373	0.484864328	0.040088341
806.6230401	1119.000438	234.1841648	9.7236234	-1.14646735	0.040163395
0	0	0	-1.24496107	-2.82051311	0.040189688
6.260980906	7.02117922	34.69395034	1.185835645	3.949196995	0.040195137
487.3130138	683.6873266	420.6641479	9.659525424	-0.55171855	0.040243081
186.7859304	381.7766201	267.1434176	7.821328822	0.643586639	0.040270026
2.086993635	2.632942208	6.938790069	3.772014177	3.395483776	0.04029729
46.65474272	52.38677346	113.189013	6.028679979	0.658723997	0.040307463
34.43539498	52.65884415	45.9694842	4.931132547	0.881904017	0.040338966
334.9624785	172.8965383	593.2665509	7.809444561	0.892491389	0.040370762
204.5253763	308.0542383	114.4900361	7.126858064	0.593179696	0.040380255
156.5245226	128.1365208	143.9798939	7.316897817	-0.63778037	0.040395438
728.1625143	191.3271337	197.9897011	8.929799455	-0.78451917	0.040428628
1.043496818	13.16471104	4.336743793	1.184150244	1.97776534	0.040457049
358.9629053	270.3154	99.74510724	8.124387802	1.412200808	0.040532958
29.21791089	20.18589026	18.21432393	4.870559769	1.416467177	0.040574467
0	0	0	0.287479596	-2.00504749	0.040576011
865.0588618	286.1130532	706.0218895	8.914513931	-0.8092568	0.040610417
0	1.755294805	25.153114	2.397156701	4.563983757	0.040651942
235.8302808	255.3953941	220.3065847	8.852177499	-0.51485991	0.040699665
56.34882815	111.4612201	48.57153048	5.632443829	0.871680999	0.040722088
18.78294272	0.877647403	11.27553386	4.058460053	-1.54039789	0.040744917
144.0025608	1018.948634	58.11236682	7.136282604	1.791006593	0.040784199
0	0.877647403	1.353064063	-8.70511228	6.984906714	0.040784734

51.13134407	90.38890598	40.76539165	5.439201307	0.690139311	0.040795044
40.69637589	32.47295389	19.94902145	4.389722214	0.867356005	0.040833044
395.4852939	578.3696383	507.3990238	8.380437984	0.561545577	0.040883577
60.52281542	42.12707532	35.5612991	3.79819274	2.205116011	0.040887259
3883.895155	2926.07644	1510.921537	12.0988347	-0.69331514	0.04089091
28.08049936	5.529178636	4.59694842	3.985174411	1.609144924	0.040904299
25.04392362	49.14825454	43.36743793	5.246572531	0.800018233	0.040915993
50.08784725	119.3600467	89.33692213	6.106202556	0.59789895	0.040954499
1109.237117	2711.930474	1492.707213	10.45010585	0.562143703	0.04099189
89.74072632	41.24942792	69.38790069	7.606154601	-1.19483377	0.040994352
157.5680195	394.0636837	429.3376355	7.659552626	0.678032938	0.040997617
10.43496818	35.9835435	45.10213545	4.502147147	0.986253225	0.040999333
0	69.3341448	2.602046276	-8.70511228	10.42312473	0.041008438
0	0	0	-8.70511228	7.443870749	0.041012012
443.4861475	254.5177467	276.684254	8.481052925	-0.52163087	0.041021915
153.3940322	361.5907298	362.5517811	7.887072549	0.60777404	0.041046445
14.60895545	17.55294805	16.47962641	3.172020685	1.421623907	0.041081424
261.9177012	128.1365208	118.8267799	7.54892226	-0.53030909	0.041147632
99.13219768	79.86591363	102.3471535	7.621834805	-0.8807552	0.041155057
0	0	0.867348759	0.189064478	-2.36294191	0.041166582
558.0412281	184.0953191	334.7272329	7.870640381	0.726172675	0.041179535
353.7454212	546.7743318	601.0726897	8.722664972	0.471815582	0.04119339
76.17526769	85.13179804	112.7553386	6.614163027	0.693455263	0.041303981
471.3996874	579.4666975	172.602403	8.423529758	0.533639677	0.041364059
255.6567203	167.6306539	292.2965316	8.287249485	-0.48636245	0.041417001
2145.429457	1597.318273	1326.176252	11.549142	-0.66858198	0.041431884
4.173987271	0	6.938790069	0.813798886	2.150016601	0.041445091
461.2255934	270.3154	209.8983996	9.22646285	-0.78644716	0.041453841
8.347974541	24.57412727	27.75516027	3.868013179	0.87851118	0.041460284
1.043496818	1.755294805	0	-8.70511228	7.726555249	0.04146581
1.043496818	0	0	-8.70511228	8.142512957	0.041525444
0	0.877647403	0.884695734	-8.70511228	7.54783213	0.041530757
96.00170723	174.6518331	159.5921716	6.709127589	0.665603277	0.041542513
0	0	0	-8.70511228	7.393346885	0.041595361
81.39275178	63.19061298	78.06138827	6.781376359	-0.6951613	0.041613172
208.6993635	320.3413019	191.6840756	7.600260363	0.483552635	0.041734001
74.08827405	0	133.5717088	-8.70511228	11.61185988	0.041739644

3.130490453	6.143531818	0.867348759	0.272831793	2.26363504	0.041740153
20.86993635	5.265884415	109.2859436	2.897860667	2.627168645	0.041752
11.47846499	11.40941623	42.50008917	4.244563208	0.917850578	0.041759302
170.0899813	44.76001753	80.66343455	6.806162052	-0.7033071	0.041778754
166.9594908	90.39768246	523.8786502	7.147950474	0.897096264	0.041783536
0	143.934174	463.1642371	3.544184695	4.72765066	0.041800225
0	0	0	0.476693678	-2.01141249	0.041849485
122.0891277	267.6824578	109.2859436	6.76287448	0.651851074	0.04186367
0	0	0	0.10541736	-2.58656774	0.041904867
0	0	0	-8.70511228	13.64215873	0.041984109
16929.69237	6952.722723	6301.288731	13.58250281	-0.95135911	0.042003858
885.9287982	572.2261064	318.3169944	9.675109958	-0.6069054	0.042020862
153.3940322	176.4071279	656.5830102	8.034671887	1.313962749	0.042152488
148.1765481	410.7389844	183.0105881	7.631137708	0.665876185	0.042172429
7.304477724	4.388237013	0.867348759	2.218971341	-1.79628954	0.042234455
20.86993635	45.63766493	33.82660158	4.02023728	1.186625744	0.042235883
968.3650468	1977.339598	1066.838973	10.97449477	-0.6452848	0.04223966
354.788918	1095.303958	926.3284741	9.000671341	0.843967834	0.042378735
148.1765481	387.9201519	255.8678838	7.33730253	0.737698913	0.042448955
81.35101191	189.0452505	76.06648613	6.151978556	0.726523155	0.042450728
0	7.898826623	0.867348759	-8.70511228	10.25561142	0.042457599
12.52196181	0.877647403	3.469395034	2.583361597	-1.77130834	0.042459271
10.43496818	4.388237013	11.30155432	2.790826057	1.029839676	0.042489783
96.00170723	132.5247578	19.94902145	6.975293476	-1.03241371	0.042506789
0	0.877647403	0.867348759	-8.70511228	7.088434595	0.042514998
291.1356121	936.4497785	621.0217111	8.864828798	0.702686245	0.042515574
0	10.53176883	0.867348759	2.884001633	4.429523067	0.042598728
717.9258106	1115.489849	877.7569437	9.620150835	0.668780005	0.042605981
58.43582179	43.88237013	145.7145914	4.823081255	1.46476361	0.042641728
90.78422314	132.5247578	146.5819402	6.871282494	-0.4954281	0.042654645
207.6558667	424.7813428	301.837368	7.933606949	0.876908145	0.042670046
434.0946761	704.7508642	842.1956446	8.944075319	0.619744132	0.042730425
7.304477724	32.47295389	12.14288262	3.138476602	1.291827143	0.042764503
336.0059753	363.3460246	303.5720655	8.257587982	0.554766562	0.042766611
477.9215425	773.2073616	304.4394143	8.791223258	0.533443311	0.04282927
4.173987271	152.710648	2.602046276	2.976344932	2.731692303	0.042863637
79.30575814	32.47295389	97.14306096	5.730846063	0.902849772	0.042903149

435.3468723	188.2290384	233.8632458	8.507828699	-0.5055849	0.042908299
1.043496818	0	0	-0.2774364	-1.97246354	0.042931352
151.3070386	148.322411	235.0515136	7.06305887	0.519794948	0.042935994
125.2196181	155.3435902	241.9903036	7.09067974	0.530636594	0.043003773
12.52196181	28.08471688	31.22455531	4.420688181	0.959449047	0.043004409
97.52521258	151.4380593	60.98329121	6.955310005	-0.94097334	0.043051146
1370.111322	2220.447928	2195.259708	10.52684929	0.656730974	0.043080299
252.5262299	603.8214129	625.3584549	8.174322165	0.790906506	0.043098724
0	0	2.471943962	-8.70511228	6.966650727	0.043099934
2.086993635	16.67530065	3.469395034	2.928271763	1.47663347	0.043125907
255.6567203	295.7671746	266.2760689	7.792184687	0.623466027	0.043155296
68.87078997	78.98826623	38.16334538	7.30555337	-0.73445404	0.043160504
1542.288297	3758.086178	3167.557666	11.3583147	0.663516713	0.043195399
81.39275178	182.5506597	413.7253578	6.856036579	0.771719383	0.043270155
0	0	0	-8.70511228	7.595483574	0.043275834
31.30490453	29.84001169	36.42864786	4.779761029	-0.75931017	0.043276885
417.3987271	294.0118798	303.5720655	8.335148167	0.646439754	0.043278123
208.6993635	42.12707532	60.7144131	5.542375088	1.125181694	0.043295553
24.00042681	3.51058961	12.14288262	5.523539728	-1.17715662	0.04329828
0	0	0	-0.24493166	-2.41525246	0.043321667
1.043496818	0	0	-1.10482742	-2.36577743	0.043349531
1107.150124	931.1663411	1742.503656	10.01605171	0.753924214	0.04336064
72.00128042	3.51058961	22.55106772	6.326656811	-1.13679912	0.043362857
8.347974541	6.143531818	1.734697517	1.797002175	1.776773967	0.043362904
219.1343317	358.0801402	228.9800723	7.773242553	0.492357571	0.043405997
0	0	0.867348759	-0.63183363	-2.26165657	0.043409408
355.8324148	200.9812552	124.8982212	8.64144453	-0.63756598	0.043473389
957.9300786	1111.101612	1812.758905	9.839359139	0.472602055	0.043485305
0	0	0	1.272369838	-2.79147344	0.043553467
7.304477724	38.61648571	26.02046276	4.247557525	1.053295588	0.043567557
0	2.632942208	0	0.016310072	-2.27126441	0.043597489
14.60895545	229.9436195	177.8064955	6.173421562	1.54461175	0.043601164
1.043496818	36.86119091	0	-8.70511228	10.09699935	0.043610921
57.39232497	30.71765909	88.46957337	6.330011102	-0.87027393	0.043639344
4.674865743	0.877647403	3.55612991	4.658015673	-2.11953747	0.04365916
125.1778782	224.2476878	1138.447287	6.969631425	1.554517439	0.043672333
0	0.877647403	0	-8.70511228	10.79045887	0.04370725

87.65373268	746.0002921	242.8576524	7.859546104	1.004575605	0.043723234
41.73987271	0	2.602046276	-8.70511228	9.588782784	0.043798115
273.3961662	778.473246	353.0109447	8.39729768	0.823303917	0.04383664
106.4366754	151.8330006	158.7248228	6.657562131	0.573038654	0.043841388
294.2661026	321.2189493	740.7158398	8.477572614	0.77644795	0.043842042
302.6140771	489.7272506	344.3374572	8.15505236	0.952730791	0.04384823
1.043496818	1.755294805	3.478068522	-0.6127622	2.711144239	0.043933874
3.756588544	6.924638006	3.868375463	1.883680454	1.173235163	0.043957043
149.2200449	466.0307707	307.9088093	7.788254483	0.745920139	0.043997331
103.3061849	176.4071279	135.3064063	6.760115546	0.641496626	0.044031046
849.4064096	563.4496324	554.2358567	9.482411731	-0.6256759	0.044045888
231.6562935	234.2879741	240.3163205	7.711133453	0.833838232	0.044060371
28.17441408	64.94590779	10.4081851	7.206046239	-1.6495524	0.044121397
125.2196181	35.9835435	136.1737551	8.754117746	-1.28811836	0.044132898
2.086993635	0.877647403	1.639289154	-8.70511228	7.417499612	0.044142569
1.043496818	15.79765325	3.469395034	-0.24008849	3.273800531	0.044158293
275.4831599	413.3719266	443.2152156	8.313892491	0.551158126	0.044158747
579.1407338	906.6097668	761.53221	9.365699756	0.428979549	0.044165418
1.043496818	2.632942208	0.867348759	-8.70511228	6.898317539	0.044227679
1.043496818	0	0.867348759	-8.70511228	6.902089632	0.044227679
0	0.877647403	0	-8.70511228	6.902267636	0.044227679
1.784379558	0	0	-8.70511228	6.963368654	0.044227679
0	0.877647403	1.327043601	-8.70511228	6.826207572	0.044227679
1.043496818	1.755294805	0	-8.70511228	6.962516833	0.044227679
1.043496818	1.755294805	0.867348759	-8.70511228	7.019477493	0.044227679
285.918128	329.1177759	261.9393251	7.587328359	0.502738652	0.044234673
10969.23855	5608.166902	9411.601379	11.56732806	1.491751827	0.044268553
145.0460577	321.2189493	492.5933805	7.603690915	0.806742753	0.044317959
6.260980906	23.69647987	13.01023138	3.924701939	1.435883785	0.044369114
41.73987271	62.31296558	36.42864786	5.435544507	0.743478595	0.044400145
0	0	0	-0.31689018	-2.18351508	0.044425078
5.217484088	1.755294805	0.867348759	1.0417981	1.853266555	0.044493383
0	4.388237013	0	-0.2540271	3.133707295	0.044495714
0	0	4.336743793	4.113665532	-2.04792698	0.044508224
0	0	0	-8.70511228	7.260199755	0.044524145
48.00085361	66.27993184	104.9491998	6.273104273	0.687708004	0.044583103
2.086993635	6.143531818	4.336743793	1.231381511	2.170392289	0.044589157

0	27.20706948	64.18380813	2.117491938	3.277253276	0.044606679
0	0	1.734697517	-3.78896353	7.646453951	0.044621154
778.448626	405.4731	533.4194865	8.744306074	0.508831991	0.04463341
5.217484088	0.877647403	3.469395034	-0.32301265	2.86866356	0.044690775
5068.264043	4612.0371	2543.06656	11.82130095	-0.6228219	0.044704177
0	15.79765325	5.204092551	1.382632198	4.279953262	0.044708018
164.8724972	251.8848045	381.6334538	7.503962444	0.634560954	0.044726082
217.0473381	345.7930766	235.0515136	7.907296897	0.597914058	0.044774877
114.7846499	88.64238765	44.23478669	6.886656159	-0.64445657	0.044781892
0	263.2942208	298.3679729	6.90151117	1.811379102	0.044791218
388.1808162	669.6449681	703.4198432	8.730627948	0.637153939	0.044868734
54.43922898	34.62319003	45.414381	6.527266832	-0.57019392	0.044888365
0	0	0	-0.78561249	-2.51670038	0.044895855
168.0029876	459.0095915	353.0109447	7.598145466	0.766583156	0.044911187
2.086993635	0	0	-0.31170299	-2.15145915	0.044917478
46.95735679	15.79765325	62.44911062	4.177948694	1.656113201	0.044929971
0	0	1.734697517	-1.76672281	3.602147159	0.04493898
0	0	0	-8.70511228	9.459884675	0.044953962
33.39189817	43.88237013	18.21432393	5.088882948	0.841930696	0.04495735
625.0545938	553.795511	273.2148589	8.750761328	0.521784837	0.044972686
6.260980906	43.88237013	207.2529859	4.100387879	2.58346343	0.045004986
634.4460651	513.4237305	732.909701	8.355176451	0.696732365	0.045021239
52.17484088	84.25415064	21.68371896	5.949977323	-0.81507991	0.04502553
303.6575739	169.3859487	156.1227765	7.559596941	-0.71386735	0.045026226
3.130490453	0	1.734697517	1.58539351	-1.65997179	0.045037915
29.56226484	12.28706364	5.204092551	4.109520589	-1.06520172	0.045118167
0	0	0	-8.70511228	11.56531368	0.04511854
21.91343317	81.62120843	15.61227765	4.293795176	1.384743585	0.045119523
38.60938225	61.43531818	45.10213545	5.339225063	0.710838793	0.045163406
9.391471359	29.84001169	20.81637021	4.595160484	1.800228166	0.045332859
73.04477724	17.55294805	74.59199324	6.827001488	-1.00507099	0.045338252
127.3066118	325.6071863	388.5722438	7.515522811	0.74015323	0.045341913
218.0908349	243.9859779	243.7250012	7.717185984	0.574658388	0.045356317
0	0	0	-8.70511228	7.816354993	0.045419592
360.0064021	540.6307999	608.8788285	8.371832699	0.617317525	0.045450869
0	0.877647403	0	-8.70511228	7.146339962	0.045487573
61.56631224	126.381226	106.6838973	6.760917658	0.642980156	0.045525429

2.086993635	27.20706948	2.602046276	5.012427742	-1.55457781	0.045557056
42.78336952	113.2165149	124.0308725	6.470492865	-0.73035175	0.045569162
0	0	0	-8.70511228	7.244873375	0.04557394
0	1.755294805	2.602046276	-8.70511228	7.187639624	0.045592348
34.43539498	39.49413311	57.24501807	4.957786496	0.927274906	0.045660739
473.7475552	313.3201227	1837.912019	9.275895387	1.089526352	0.045692774
0	0	1.734697517	0.004581871	-2.27549627	0.045703306
194.0904081	464.2754759	328.7251795	7.64779311	0.635889505	0.045719978
3.130490453	1.755294805	6.938790069	1.438740822	2.095823918	0.045816839
27.13091726	0	0.867348759	-8.70511228	10.1862706	0.045848279
221.2213253	232.5765617	480.5112122	7.812802261	0.693425995	0.045853376
8.347974541	186.9388967	51.17357676	4.69609956	1.538103084	0.045893996
112.6976563	131.6471104	44.23478669	5.898379412	0.819590116	0.045906653
10.43496818	2.632942208	14.7449289	3.484694272	-1.14847784	0.045944099
0	0	0	0.311250306	-1.98287412	0.045973764
333.9189817	361.5907298	531.684789	8.147403753	0.621399233	0.04600725
112.6976563	210.6353766	164.7962641	6.703624888	0.7447307	0.046056498
7.304477724	7.898826623	15.61227765	2.886809115	1.510163128	0.046091857
745.0567278	1308.572277	1196.073938	9.476131333	0.674514773	0.046105725
0	0	0.867348759	1.136603052	-2.14948195	0.046109122
16.69594908	89.52003506	136.1737551	4.849124796	1.395503201	0.04612167
1.022626881	2.062471396	1.595921716	1.502086944	1.865676109	0.04613694
6.260980906	16.67530065	9.540836344	6.319212636	3.034908995	0.046179455
3205.622224	3213.067141	1482.299028	11.71883052	-0.66640329	0.046191483
0	0.877647403	0	-0.6764812	-2.32481421	0.046257289
8.347974541	2.632942208	16.47962641	3.474606333	-1.18497966	0.046260851
0	0	0	0.437982945	2.719843301	0.046268191
298.9618383	734.9858172	548.476661	8.287034736	0.765685968	0.046269157
65.74029951	193.960076	203.8269583	7.551928403	1.497033321	0.046270218
756.5351928	883.7909343	1280.206768	9.537575006	0.659918289	0.046295749
0	3.51058961	3.469395034	-8.70511228	9.064709645	0.046319933
105.7270976	181.7871065	95.04407696	7.366072217	-1.00202884	0.046363493
9.391471359	8.776474025	2.602046276	4.100334578	-1.18637288	0.046377297
10.61236264	0	0	-8.70511228	9.504466077	0.046384565
0	0	0	-0.18062042	-2.16182833	0.04639053
2065.080202	1782.501874	2229.08631	10.62222865	0.617042093	0.046393285
2621.264006	1574.49944	768.4710001	11.00607799	-0.73248728	0.046412625

1121.759079	1263.81226	1726.891378	9.710515354	0.687782522	0.046416092
70.86386889	137.2552773	88.98130914	6.343985566	0.737229379	0.04643296
877.5808237	641.5602512	677.3993804	9.750184467	-0.50468309	0.046461813
98.08870086	132.5247578	125.76557	7.097735225	-0.43663663	0.046464579
135.6545863	272.0706948	162.1942179	7.205751638	0.617315566	0.04654573
0	0.877647403	0	2.556649553	-2.04867741	0.046643148
65.74029951	67.57884999	52.90827427	5.718459843	1.070808248	0.04664786
80.34925496	64.06826038	73.72464448	6.915951712	1.2992746	0.046703196
2100.559094	1520.962949	1598.523762	10.25978826	0.531268116	0.046733249
424.9223391	310.5555334	350.027265	9.086233277	-0.51179514	0.046749505
0	8.776474025	0	2.447509628	4.536780759	0.046789621
378.7893448	121.9929889	144.8472427	7.723886831	-1.20681207	0.046864585
29.21791089	9.654121428	14.7449289	4.812903602	-1.17980506	0.046884952
113.7411531	64.94590779	106.6838973	6.862005913	-0.57631622	0.046886625
0	0	0.867348759	-8.70511228	8.627646815	0.046895495
319.3100262	216.7789084	276.684254	7.604350113	0.439553331	0.046898418
7.304477724	13.16471104	47.70418172	5.359237568	-1.09568759	0.046900957
219.1343317	543.2637422	927.1958229	7.392868256	1.165402999	0.046926518
280.700644	205.3694922	382.5008025	7.76349801	0.567573597	0.046950782
43.82686634	8.776474025	17.34697517	4.762831364	-1.04877507	0.046951918
189.9164208	129.0141682	98.01040972	7.196058434	0.887861981	0.046968708
237.9172744	255.3953941	266.7617842	7.728864193	0.364994533	0.047022591
273.3961662	36.86119091	909.8488477	5.601283183	2.586887506	0.047023718
128.3501086	144.8118214	273.2148589	7.21733801	0.558990977	0.047110951
0	2.632942208	0	-8.70511228	8.065753699	0.047187022
7.304477724	1.755294805	0	1.70961818	2.500516982	0.047211937
52.17484088	59.68002337	29.48985779	5.365714816	1.127487405	0.047215486
0	0	0	-8.70511228	7.300430563	0.047246341
1.043496818	31.59530649	26.88781152	4.449241723	2.582816261	0.047258594
2.086993635	0	0	-8.70511228	7.045318827	0.047261666
35871.71618	7555.376865	26687.41926	13.78111041	0.903658329	0.047331038
94.95821041	173.7741857	108.4185948	6.022389968	1.083599588	0.047346604
0	0.877647403	3.469395034	-8.70511228	7.527980658	0.047383715
169.0464845	262.4165733	282.7556953	8.253370063	-0.95860381	0.047390818
257.743714	222.0447928	537.7562303	8.651919936	-0.64156648	0.047395816
189.9164208	232.5765617	215.1024921	7.35815694	0.443351329	0.047398159
532.183377	1362.108769	792.7567653	9.356249712	0.526454905	0.04742519

147.1330513	202.73655	184.7452856	7.395723706	0.56597982	0.04743454
148.1765481	201.8589026	207.2963533	7.183507816	0.86173075	0.047518055
3903.721595	1474.447636	3563.0687	11.49444185	0.799489123	0.047547694
18.78294272	34.2282487	19.08167269	3.929316703	1.139740951	0.047578206
0	3.51058961	0.867348759	1.907818331	-1.44817045	0.047631698
159.6550131	137.7906422	207.2963533	7.736854871	-0.47907144	0.047660054
0	0	0.867348759	-8.70511228	7.601590027	0.047702994
244.1782553	649.4590779	469.2356784	8.250977897	0.596334798	0.047737369
101.2191913	9.654121428	58.11236682	6.066486607	-0.91358994	0.04776439
113.7411531	354.5695506	222.908631	7.156980746	0.608285409	0.047770484
4729.127578	3271.869517	1611.533993	11.85036419	-0.73211179	0.047781234
66.78379633	200.1036078	65.91850565	7.650172855	1.64060513	0.047795378
799.3185623	1536.734272	1274.091959	9.794302749	0.504195901	0.047796706
512.3569375	532.7319733	443.2152156	8.567675332	0.489443346	0.047796724
12.52196181	168.5083013	104.9491998	4.55827638	2.392968242	0.047824161
24.00042681	38.61648571	31.22455531	4.70048355	0.812769846	0.047830071
1920.284584	2090.635101	1255.929676	10.67498801	0.582914189	0.047873154
18.78294272	1.755294805	6.07144131	3.870654194	2.557151665	0.047954745
21.91343317	26.32942208	9.540836344	4.235157658	0.813779854	0.047955467
397.5722875	704.7508642	584.5930633	8.827205529	0.493940965	0.048042044
7.304477724	3.51058961	6.07144131	3.779272158	-0.97805181	0.048051391
57.39232497	42.12707532	40.76539165	4.9384737	0.717875177	0.048059393
201.3948858	179.0400701	341.7354109	7.61110278	0.59256329	0.048074072
106.6766797	100.9294513	310.588917	7.156003089	0.973310307	0.048082024
74.08827405	338.7718974	84.13282958	6.575386858	1.07553822	0.048149692
37.56588544	142.1788792	26.02046276	5.651610745	0.954367835	0.048233174
25.04392362	141.3012318	72.50168273	5.920205413	1.262524264	0.048233521
0	0	0	-0.81742318	-1.99875212	0.048314756
1.043496818	0.877647403	1.734697517	3.097465593	-1.43821545	0.048327825
574.9667465	1116.367496	981.8387947	9.441016776	-0.74775427	0.048333588
83.47974541	93.90827207	111.8879899	6.357922608	1.512644921	0.04839221
134.6110895	203.6141974	216.8371896	7.236466812	0.657054713	0.048403253
23.00910483	13.19981693	24.28576524	3.642041783	1.002509114	0.048407727
0	0.877647403	0.867348759	0.295766485	2.309504425	0.048415415
40.69637589	31.59530649	11.27553386	7.216243073	-1.4640514	0.048443964
22.96736496	22.81883247	14.7449289	4.782718625	-1.11588888	0.048456046
132.5240958	180.7953649	183.8779368	7.018109286	0.473782855	0.048567585

137.7624499	143.3373738	136.8502871	6.524379382	0.772549891	0.048585074
16.69594908	31.59530649	14.7449289	3.72651547	1.259303372	0.048591184
22.95692999	12.28706364	14.7449289	3.938368953	0.948708305	0.048607483
260.8742044	0	430.2049842	3.20053772	4.935340533	0.048632756
18.78294272	0	5.204092551	-8.70511228	8.682637622	0.048721513
92.87121677	37.73883831	74.59199324	7.443529914	-0.93400805	0.048729191
3.600064021	64.07703686	6.192870136	3.794986047	1.631428993	0.048736215
7.304477724	0.877647403	4.336743793	2.079468773	2.390471578	0.048744341
0	1.52710648	0	-8.70511228	7.262522289	0.048818311
358.8585556	191.3183573	561.0792384	8.479716895	0.572435413	0.048899913
0	3.51058961	0.867348759	5.365937379	-2.1923125	0.048941797
1.043496818	0	0	0.456007994	4.665993505	0.048952951
784.7096069	1110.223964	618.4196649	10.28403822	-0.70043685	0.048982805
231.6562935	387.9201519	337.3986671	7.918164521	0.477262511	0.048988016
9.391471359	0	4.336743793	2.387390525	-1.29704433	0.048989177
379.8328416	216.7789084	576.7869244	8.031199062	0.672523772	0.0490243
171.1334781	86.00944545	62.44911062	7.116650298	0.674291399	0.04902748
2085.950139	1364.741711	3356.639696	10.84514077	0.727560101	0.049047438
61.56631224	86.00944545	92.80631717	6.246448184	-0.78036225	0.049123527
30.26140771	31.59530649	24.28576524	5.033754298	1.228446777	0.049161181
63.65330588	36.86119091	26.88781152	5.999848624	1.863677564	0.049215007
306.7880644	601.1884707	616.6849673	8.253353111	0.689571336	0.049217005
346.4409435	645.0708408	491.7867461	8.497299936	0.640776521	0.049231225
11.47846499	0.877647403	19.94902145	4.244458521	-1.45830297	0.04929036
82.4362486	0	70.25524944	1.431582998	4.560490015	0.049353559
129.3936054	120.2376941	163.0615666	7.185461113	-0.52304335	0.049363334
105.0801295	179.9177175	116.8578982	6.988256181	0.596264898	0.049383556
18.78294272	0	77.19403951	-8.70511228	10.493145	0.049392757
18.78294272	92.15297726	13.01023138	4.383212932	1.149234511	0.049426532
1348.197888	1548.170018	1928.983639	10.86045791	-0.56715025	0.049428351
0	0	0.867348759	-8.70511228	8.372149536	0.049466238
0	0	0.867348759	0.218174733	4.078110968	0.049487979
158.6115163	79.86591363	200.3575632	7.29592713	0.700651318	0.049518688
401.7462748	639.8049564	392.9089876	8.349438806	0.534854371	0.049519421
0	0.877647403	25.153114	-8.70511228	9.345968071	0.049537238
0	0	0.867348759	-8.70511228	7.829078226	0.04953835
0	7.02117922	0.867348759	-8.70511228	8.150278002	0.049573719

637.5765556	528.3437363	282.7556953	9.140744656	-0.52921973	0.049680325
2.086993635	1.755294805	0	2.541506955	4.099018177	0.049680757
212.8733508	271.1930474	428.4702867	8.050496995	0.770409289	0.049711116
471.6605616	413.3719266	725.9709109	8.909059669	0.731720634	0.049741811
916.1902059	494.1154876	154.388079	9.451358308	-0.88310935	0.049747683
32.34840135	108.8282779	64.18380813	6.376610253	0.973756265	0.049772201
0	6.143531818	5.204092551	5.49651532	4.210175192	0.049784218
32.34840135	49.70994888	78.06138827	5.189868229	0.760396476	0.049795249
5.217484088	3.51058961	1.734697517	0.332834517	2.41964655	0.049808305
0	1.755294805	1.734697517	-8.70511228	7.310608307	0.049833346
8533.716975	175.5294805	114.4900361	9.142988616	3.023176364	0.04987164
945.4081168	287.868348	1392.962106	9.463015752	1.005774629	0.049905042
2123.516024	712.6496908	799.6955554	10.22058319	-1.07437545	0.049919445
9.391471359	8.776474025	41.63274041	1.866184369	3.278304972	0.049931742
354.788918	186.0612493	1128.420735	7.959983044	1.108583564	0.049944411
340.1799626	49.14825454	196.0208194	6.780731739	0.952920771	0.049975988
107.4801722	230.8212669	287.9597878	6.677429117	0.826697771	0.049993153
108.523669	172.8965383	169.1330079	6.836666244	0.528410287	0.049995586

q.value
3.80E-05
0.000206815
0.000206815
0.000349849
0.000567688
0.000670537
0.000710029
0.000723411
0.000723411
0.000766704
0.000864378
0.000990291
0.001019147
0.001019147
0.001019147
0.001173107
0.001385804
0.001385804
0.001687231
0.001747767
0.001747767
0.001747767
0.001786473
0.001886659
0.001946639
0.001946639
0.001946639
0.001946639
0.001946639
0.001946639
0.002026359
0.002073641
0.002158878
0.002158878

NL, Normal licer; HCC, Hepatocellular carcinoma

0.002158878
0.002158878
0.002158878
0.002158878
0.002158878
0.002158878
0.002158878
0.002158878
0.002158878
0.002171617
0.002371337
0.002596526
0.002596526
0.002596526
0.003243982
0.003373142
0.003373142
0.003373142
0.003598782
0.003598782
0.003598782
0.004011523
0.004011523
0.004091467
0.004091467
0.004091467
0.004782038
0.00486221
0.005523565
0.005810256
0.005810256
0.005810256
0.005810256
0.005905747
0.005905747
0.006090759
0.006090759

0.006090759
0.006314889
0.006538249
0.006538249
0.006538249
0.006538249
0.006541382
0.007179604
0.007241106
0.007241106
0.00794226
0.008089175
0.008238087
0.008238087
0.008418184
0.008418184
0.00865131
0.009328815
0.009328815
0.009328815
0.009404048
0.009545005
0.009545005
0.009545005
0.009545005
0.010056752
0.010056752
0.010665858
0.011096249
0.011250894
0.011250894
0.011250894
0.011250894
0.011250894
0.011250894
0.011250894
0.012124599

0.0121693
0.012332093
0.012638519
0.012674731
0.012709427
0.012799588
0.012799588
0.012799588
0.012799588
0.013258655
0.013258655
0.013258655
0.013258655
0.013258655
0.013588197
0.013588197
0.013588197
0.014394182
0.015073577
0.015190712
0.015223579
0.015593715
0.015661996
0.015983344
0.016425273
0.016425273
0.016437371
0.016468697
0.016468697
0.01675245
0.017697474
0.018060439
0.018214149
0.01843823
0.018654085
0.018654085
0.019092998

0.019414415
0.019524172
0.019900216
0.020393308
0.020393308
0.021184075
0.021184075
0.021184075
0.021194179
0.021194179
0.021212499
0.022233261
0.022299351
0.022299351
0.022508882
0.022516337
0.022516337
0.022516337
0.022620295
0.023350994
0.023350994
0.023350994
0.023350994
0.023350994
0.023468118
0.023967762
0.024822069
0.024822069
0.024959493
0.024959493
0.0256813
0.025703815
0.026870552
0.02703403
0.027255293
0.027360741
0.027360741

0.027984485
0.027984485
0.027984485
0.028031135
0.028031135
0.028031135
0.028118304
0.028118304
0.028118304
0.028443298
0.028443298
0.028443298
0.029923676
0.029923676
0.03117057
0.031435926
0.031590761
0.031590761
0.032053657
0.033317981
0.033496305
0.034080306
0.034270821
0.034393136
0.034393136
0.034393136
0.034393136
0.034393136
0.034731693
0.035448277
0.035593161
0.035595084
0.035595084
0.036040906
0.036575372
0.036862254
0.036862254

0.03762933
0.038019335
0.038306024
0.038916066
0.039198122
0.041385906
0.041432112
0.04190014
0.042497101
0.042746835
0.042824979
0.043104089
0.043104089
0.043104089
0.043254454
0.043364308
0.043396392
0.043396392
0.043498021
0.044298598
0.044298598
0.044298598
0.045465898
0.046221104
0.046490303
0.046596294
0.046745353
0.046936857
0.046936857
0.046936857
0.046936857
0.046978418
0.047075692
0.047075692
0.048016189
0.048016189
0.048471145

0.048554672
0.048715592
0.049001999
0.049277664
0.049297447
0.049400295
0.049828514
0.050025218
0.050029278
0.050031629
0.050031629
0.050031629
0.050031629
0.050129711
0.050339156
0.050367622
0.050475463
0.050475463
0.050475463
0.050475463
0.050475463
0.050475463
0.050475463
0.050475463
0.050475463
0.050475463
0.050475463
0.050862836
0.050862836
0.050862836
0.051383046
0.051383046
0.051383046
0.051448477
0.051448477
0.051640723
0.052433855

0.052720563
0.052720563
0.052720563
0.053041674
0.053501081
0.053501081
0.053626921
0.053626921
0.054104596
0.054555036
0.054841161
0.055472822
0.055604337
0.055703511
0.055707224
0.056337458
0.056337458
0.056351552
0.057303403
0.057303403
0.057303403
0.057303403
0.057303403
0.057522287
0.057739741
0.057739741
0.057739741
0.057743856
0.057937758
0.058180706
0.058476807
0.058476807
0.058476807
0.058476807
0.058527631
0.058531102
0.058531102

0.058531102
0.058775848
0.059842205
0.059879565
0.060286588
0.061484944
0.061940496
0.061940496
0.061940496
0.062496826
0.062496826
0.062496826
0.062496826
0.062510427
0.062924512
0.06303716
0.06303716
0.064607346
0.064665629
0.064665629
0.064950932
0.065017584
0.065342589
0.065850327
0.065860873
0.065860873
0.065860873
0.066003865
0.066003865
0.066133339
0.066602934
0.066915194
0.067056643
0.067056643
0.067056643
0.067056643
0.067061477

0.067296712
0.067558571
0.067558571
0.067558571
0.067558571
0.067558571
0.067558571
0.067558571
0.067558571
0.067558571
0.067558571
0.068189813
0.068695455
0.069759502
0.069759502
0.069873178
0.069891146
0.070487617
0.070487617
0.071539681
0.071539681
0.071556347
0.071913373
0.071913373
0.071913373
0.071913373
0.071913373
0.071913373
0.071913373
0.071913373
0.071913373
0.071913373
0.071913373
0.071913373
0.071913373
0.071913373
0.071913373

0.071913373
0.071913373
0.071913373
0.071913373
0.071913373
0.071913373
0.071913373
0.071913373
0.071913373
0.071913373
0.071913373
0.071913373
0.071913373
0.071913373
0.071913373
0.072108123
0.072157666
0.072496035
0.073190935
0.073190935
0.073334236
0.073366699
0.073366699
0.073563848
0.073563848
0.073599217
0.073599217
0.074182208
0.074338291
0.074836019
0.075582495
0.075866255
0.075866255
0.075866255
0.075866255
0.075936874
0.076554083

0.076554083
0.076560781
0.076840752
0.077033598
0.077033598
0.077033598
0.077033598
0.077033598
0.077033598
0.077033598
0.077033598
0.077033598
0.077033598
0.077033598
0.077033598
0.077033598
0.077033598
0.077515517
0.077515517
0.077515517
0.077515517
0.077515517
0.077786048
0.077989877
0.078538798
0.078538798
0.078538798
0.078864659
0.079057735
0.079958349
0.08088322
0.081529307
0.081529307
0.081773566
0.082420479
0.082420479
0.082821436

0.083623756
0.084034213
0.084274401
0.085135587
0.0852934
0.086316114
0.086316114
0.086316114
0.086316114
0.086316114
0.086316114
0.086316114
0.086316114
0.086316114
0.086316114
0.086540979
0.087114565
0.088405311
0.088405311
0.088606511
0.088995074
0.090164794
0.090445675
0.090526694
0.090629438
0.090871145
0.090872233
0.091229716
0.091281471
0.091393318
0.091508648
0.092407483
0.09272217
0.092865394
0.093764225
0.09402305
0.094185347

0.095542
0.095542
0.095542
0.096260593
0.096845579
0.098131919
0.09853555
0.09853555
0.09853555
0.098755125
0.098755125
0.098756499
0.100212618
0.100353138
0.100423147
0.100423147
0.102017282
0.102045169
0.102114456
0.102114456
0.102114456
0.102114456
0.102673044
0.105104229
0.105104229
0.105104229
0.105104229
0.105104229
0.105132897
0.10577099
0.105827096
0.105914249
0.105914249
0.106180803
0.106442286
0.106442286
0.106442286

0.106709292
0.106709292
0.107058583
0.107137889
0.108351064
0.108973039
0.108973039
0.109329054
0.109329054
0.109577208
0.110042854
0.110384298
0.110544636
0.110544636
0.111423302
0.111582898
0.111582898
0.111582898
0.111582898
0.111582898
0.111844998
0.111989011
0.112260539
0.112260539
0.112392122
0.11261662
0.113594895
0.114619692
0.114619692
0.114619692
0.114619692
0.114619692
0.114619692
0.114830311
0.114878908
0.11499924
0.11499924

0.11499924
0.115067242
0.115067242
0.1152448
0.1152448
0.115814667
0.116527468
0.116776418
0.116924807
0.116924807
0.116924807
0.116924807
0.116924807
0.116924807
0.116924807
0.116924807
0.116924807
0.116924807
0.116924807
0.116924807
0.116924807
0.116924807
0.117295578
0.117295578
0.117648953
0.118904547
0.118904547
0.119591603
0.119591603
0.119591603
0.119591603
0.119591603
0.119771483
0.120239033
0.120239033
0.120486596
0.120608084

0.12075153
0.120871093
0.120961305
0.120961305
0.121158639
0.121158639
0.121158639
0.121158639
0.121158639
0.121158639
0.121158639
0.121366166
0.121615648
0.121935878
0.121935878
0.122187331
0.122187331
0.123175506
0.124158828
0.124265162
0.124265162
0.124265162
0.124265162
0.124265162
0.125118917
0.125118917
0.125151851
0.125664629
0.125664629
0.12586013
0.12586013
0.126324653
0.126324653
0.126324653
0.127185613
0.127185613
0.127245882

0.127245882
0.128082919
0.128999581
0.129935696
0.130160763
0.131184026
0.131184026
0.131421415
0.131535477
0.131978902
0.13198198
0.132121111
0.132121111
0.132121111
0.132647666
0.132690298
0.133650696
0.133916448
0.133916448
0.134630911
0.134901972
0.134901972
0.13539984
0.135593096
0.136066449
0.136066449
0.136066449
0.136066449
0.136066449
0.136066449
0.136066449
0.136066449
0.136066449
0.136066449
0.136066449
0.136583199
0.136726531

0.137480143
0.137480143
0.137480143
0.137480143
0.137480143
0.137480143
0.137480143
0.137480143
0.137480143
0.137480143
0.137713707
0.137884571
0.137986985
0.139456024
0.139552275
0.13976313
0.13976313
0.13976313
0.13976313
0.140277256
0.140277256
0.140277256
0.140944875
0.141035728
0.141035728
0.141481818
0.14218186
0.14218186
0.143112925
0.143411162
0.143411162
0.143411162
0.145077433
0.145387931
0.1455699
0.145848018
0.145848018

0.145848018
0.145848018
0.145848018
0.145848018
0.145848018
0.145848018
0.146578084
0.146578084
0.146888418
0.147015519
0.147666488
0.147742536
0.148274795
0.148274795
0.149012062
0.149077331
0.149270404
0.149270404
0.149270404
0.149270404
0.149318089
0.149682228
0.150058152
0.15020076
0.15020076
0.150362673
0.150541627
0.151110169
0.151675072
0.151675072
0.151718371
0.151767393
0.151767393
0.151767393
0.151993273
0.151993273
0.152424863

0.152424863
0.152424863
0.152424863
0.152424863
0.152424863
0.152424863
0.153365666
0.153365666
0.153365666
0.153768912
0.153873329
0.153899976
0.153944322
0.154644053
0.154644053
0.154644053
0.154840877
0.154840877
0.154997091
0.155725339
0.155725339
0.155725339
0.155725339
0.155725339
0.155725339
0.155725339
0.155725339
0.155725339
0.155725339
0.1558555
0.155859692
0.155880456
0.155880456
0.15628326
0.1565799
0.1565799
0.156806674

0.156806674
0.156806674
0.156814074
0.157107641
0.15756486
0.15756486
0.157982449
0.158074251
0.158074251
0.158074251
0.159058282
0.159900922
0.159900922
0.159900922
0.160254886
0.160254886
0.160254886
0.160254886
0.160254886
0.160254886
0.160291767
0.160291767
0.160291767
0.160291767
0.160291767
0.160291767
0.160470395
0.160792429
0.160792429
0.160792429
0.160792429
0.160792429
0.160792429
0.160792429
0.160792429
0.160792429
0.160792429

0.160792429
0.160792429
0.160792429
0.160792429
0.160792429
0.160792429
0.160792429
0.160792429
0.160792429
0.160792429
0.160930474
0.16160921
0.16160921
0.161787116
0.162701676
0.1629878
0.1629878
0.163317771
0.163317771
0.163317771
0.163317771
0.163668776
0.163773304
0.163917885
0.164150805
0.164150805
0.164150805
0.164150805
0.164150805
0.164327731
0.164327731
0.164327731
0.164327731
0.164394209
0.165320733
0.165320733
0.165320733

0.165424271
0.165424271
0.165572672
0.165572672
0.165572672
0.165572672
0.165572672
0.165572672
0.165572672
0.166307671
0.166307671
0.1667005
0.166912396
0.167692492
0.167845278
0.167845278
0.167845278
0.168136804
0.169197039
0.169231041
0.170257771
0.170562105
0.170608634
0.170608634
0.170608634
0.171113355
0.171132023
0.171132023
0.171132023
0.171629468
0.171629468
0.172136242
0.172464844
0.172916513
0.173530192
0.174210787
0.174210787

0.174320037
0.174320037
0.174597357
0.174597357
0.174868497
0.174868497
0.175053279
0.175053279
0.17516476
0.17516476
0.17516476
0.17516476
0.17516476
0.17516476
0.17516476
0.17516476
0.17516476
0.17516476
0.175336209
0.175723214
0.175723214
0.175723214
0.175868555
0.175868555
0.176202063
0.176648937
0.176648937
0.176735612
0.176735612
0.176957873
0.177237233
0.177254185
0.178230642
0.178720104
0.178720104
0.178840624
0.178840624

0.179014499
0.179308373
0.179308373
0.179308373
0.179308373
0.179308373
0.179308373
0.179311162
0.179328907
0.179328907
0.179328907
0.179328907
0.179370799
0.179544643
0.179544766
0.179544766
0.179898554
0.180080859
0.180080859
0.18023773
0.180272147
0.180272147
0.18036093
0.18036093
0.18036093
0.180505301
0.180552685
0.180552685
0.180739173
0.181452057
0.181452057
0.181897163
0.181897163
0.182198766
0.183048881
0.183559938
0.183726247

0.183726247
0.183726247
0.183726247
0.183726247
0.183726247
0.183769387
0.18411205
0.184265787
0.184265787
0.184265787
0.184265787
0.184265787
0.184265787
0.184265787
0.184265787
0.184265787
0.184265787
0.184265787
0.184265787
0.184265787
0.184794458
0.184883727
0.184883727
0.184883727
0.184883727
0.184939964
0.184939964
0.185181518
0.185186073
0.185655977
0.186547266
0.186758014
0.186758014
0.186758014
0.186758014
0.186758014
0.186758014

0.186933959
0.186933959
0.187417359
0.18790506
0.188282936
0.188282936
0.188282936
0.188282936
0.188282936
0.188560613
0.188560613
0.188560613
0.188560613
0.188560613
0.188560613
0.188606309
0.188863287
0.188863287
0.188863287
0.188969227
0.189758963
0.190184742
0.19029789
0.19029789
0.19029789
0.19067674
0.190975756
0.191188839
0.191188839
0.191628641
0.192129423
0.192131158
0.192131158
0.192131158
0.192131158
0.192131158
0.192154808

0.192454128
0.192689373
0.19286396
0.192933741
0.192933741
0.192933741
0.192933741
0.192933741
0.193393689
0.193393689
0.193393689
0.193393689
0.193393689
0.193393689
0.193949929
0.19417468
0.19417468
0.19417468
0.194332241
0.194332241
0.194708382
0.194708382
0.195512168
0.195869097
0.195869097
0.195869097
0.196530636
0.196812386
0.196812386
0.196835519
0.197006616
0.197808228
0.197808228
0.197808228
0.197825966
0.197825966
0.198241062

0.19872489
0.19876025
0.199990893
0.199990893
0.199990893
0.199990893
0.199990893
0.199990893
0.199990893
0.199990893
0.199990893
0.199990893
0.199990893
0.199990893
0.199990893
0.199990893
0.200311805
0.200311805
0.200311805
0.200311805
0.201519468
0.201519468
0.202133089
0.202133089
0.202133089
0.202133089
0.202561723
0.203140357
0.204968061
0.204968061
0.20692947
0.20692947
0.20692947
0.207073955
0.208436324
0.208436324
0.209370708

0.210105524
0.210105524
0.211130556
0.211130556
0.211310587
0.211319123
0.212411588
0.212609628
0.212609628
0.212609628
0.213622346
0.213888334
0.214317206
0.214317206
0.214317206
0.214396184
0.214396184
0.214396184
0.214822285
0.215522677
0.21590337
0.216264478
0.217559028
0.217778954
0.218045392
0.218045392
0.218045392
0.218045392
0.218045392
0.218045392
0.218045392
0.218045392
0.218335658
0.218486743
0.218751279
0.219350834
0.219587197

0.220310184
0.220320255
0.220496955
0.221419774
0.221509472
0.221821967
0.221821967
0.221923472
0.221923472
0.221923472
0.222895141
0.222895141
0.223367531
0.223367531
0.223614131
0.223648337
0.224054524
0.224054524
0.224369655
0.224578729
0.224578729
0.224777554
0.225093842
0.225093842
0.225093842
0.225093842
0.225393959
0.225393959
0.225393959
0.225723867
0.225833958
0.225833958
0.226305394
0.226305394
0.227115009
0.227115009
0.227115009

0.227115009
0.227663779
0.227663779
0.22782422
0.22782422
0.22782422
0.22782422
0.22782422
0.22782422
0.22782422
0.22782422
0.22782422
0.22782422
0.22782422
0.22782422
0.22782422
0.22782422
0.22782422
0.22782422
0.228734877
0.228734877
0.228734877
0.228854336
0.229007768
0.229263614
0.229263614
0.229263614
0.229263614
0.229263614
0.229263614
0.230288515
0.230931942
0.231112036
0.231112036
0.231112036
0.231112036
0.231112036

0.231112036
0.231112036
0.231112036
0.231112036
0.231112036
0.231271306
0.231968019
0.233040085
0.233045328
0.233268605
0.233268605
0.233268605
0.233268605
0.233268605
0.233268605
0.233268605
0.233268605
0.233268605
0.233268605
0.233673706
0.233673706
0.234026538
0.234127279
0.234127279
0.234131288
0.234131288
0.23428877
0.234566728
0.234566728
0.234566728
0.234566728
0.234582155
0.234924058
0.235573127
0.235748075
0.235748075
0.235817786

0.235817786
0.235817786
0.235817786
0.235817786
0.235817786
0.235817786
0.235817786
0.235817786
0.235817786
0.235817786
0.235817786
0.236411968
0.236684054
0.236710223
0.236710223
0.236904363
0.236904363
0.237063485
0.237063485
0.237137094
0.237137094
0.237137094
0.237448887
0.237448887
0.237448887
0.237448887
0.237448887
0.237889342
0.238252977
0.238252977
0.23848677
0.23848677
0.23848677
0.23848677
0.23848677
0.23848677
0.23848677

0.23848677
0.23848677
0.23848677
0.238513471
0.239019554
0.239019554
0.239019554
0.239019554
0.239019554
0.239100529
0.239251903
0.239511478
0.239511478
0.239511478
0.239511478
0.240305942
0.240582902
0.241915214
0.242126805
0.242126805
0.242126805
0.242126805
0.242126805
0.242126805
0.242126805
0.242126805
0.242126805
0.242168756
0.242854901
0.242956115
0.243116835
0.243187398
0.243187398
0.243187398
0.243353715
0.243353715
0.244727428

0.245943505
0.246822269
0.247637129
0.248027578
0.24848395
0.248541746
0.248541746
0.248557252
0.248601931
0.248962409
0.249103653
0.249103653
0.249103653
0.249591507
0.250001834
0.250001834
0.250001834
0.250001834
0.250001834
0.250001834
0.250202248
0.250202248
0.250202248
0.251531323
0.251531323
0.251531323
0.251531323
0.251531323
0.251531323
0.251531323
0.251531323
0.2516524
0.252357756
0.252390948
0.252390948
0.252390948
0.25256739

0.252620209
0.252620209
0.252620209
0.252620209
0.252620209
0.252620209
0.252620209
0.252620209
0.254158968
0.254249642
0.254342835
0.254513115
0.254513115
0.255161193
0.255484851
0.255484851
0.255484851
0.255928401
0.256510654
0.256510654
0.256510654
0.256510654
0.256510654
0.256510654
0.256510654
0.256510654
0.257590121
0.257590121
0.257590121
0.257699532
0.257825278
0.257825278
0.257825278
0.257825278
0.257825278
0.257825278
0.257825278

0.257825278
0.257825278
0.257825278
0.257825278
0.258304918
0.258583945
0.258583945
0.258583945
0.258583945
0.258583945
0.258756766
0.258756766
0.258756766
0.258756766
0.259294528
0.259294528
0.259294528
0.259294528
0.259294528
0.259874584
0.261206663
0.261206663
0.261206663
0.261206663
0.261206663
0.261206663
0.261206663
0.261206663
0.261206663
0.261206663
0.261206663
0.261206663
0.261206663
0.261216505
0.261216505
0.262002187
0.262002187

0.262002187
0.262002187
0.262032493
0.262171
0.262355664
0.262658383
0.262658383
0.262658383
0.262658383
0.262658383
0.262658383
0.262658383
0.262658383
0.262658383
0.262658383
0.263110326
0.263110326
0.263352374
0.263352374
0.263352374
0.263923516
0.264167109
0.264960534
0.26496715
0.26496715
0.26557894
0.266040405
0.266040405
0.266040405
0.266112918
0.266112918
0.266507684
0.266656478
0.266678904
0.2668298
0.26691826
0.267236587

0.268573206
0.268573206
0.268573206
0.268958328
0.268958328
0.268958328
0.268958328
0.268958328
0.269024338
0.269024338
0.269024338
0.269024338
0.269024338
0.269024338
0.269024338
0.269024338
0.269024338
0.269024338
0.269024338
0.269024338
0.269024338
0.270334461
0.270334461
0.270334461
0.270334461
0.270334461
0.270334461
0.270334461
0.270425883
0.270647697
0.270673331
0.271297369
0.271465065
0.271465065
0.271465065
0.271465065
0.271465065

0.271465065
0.271465065
0.271465065
0.271465065
0.271465065
0.271465065
0.271465065
0.271465065
0.271465065
0.271764732
0.271837769
0.271837769
0.271837769
0.271837769
0.271837769
0.271837769
0.271837769
0.271912462
0.272608537
0.272843382
0.272843382
0.272843382
0.272843382
0.272868611
0.27290947
0.273233571
0.273365349
0.273394596
0.273394596
0.273394596
0.273704182
0.273704182
0.273935447
0.273935447
0.273935447
0.273935447
0.273935447

0.273935447
0.273935447
0.273935447
0.274302264
0.274691869
0.275074382
0.275074382
0.275074382
0.275074382
0.275074382
0.275074382
0.275074382
0.275120315
0.275629713
0.275629713
0.275629713
0.275870307
0.275870307
0.275870307
0.275870307
0.275870307
0.275870307
0.275870307
0.275870307
0.275870307
0.275870307
0.275870307
0.275870307
0.275870307
0.275870307
0.275870307
0.275870307
0.275870307
0.275870307
0.275870307
0.275870307
0.275870307

0.275910651
0.276246149
0.27735473
0.27735473
0.27735473
0.27735473
0.27735473
0.27735473
0.27735473
0.27735473
0.27735473
0.27735473
0.27735473
0.27735473
0.27735473
0.27735473
0.27735473
0.277542103
0.277542103
0.277542103
0.277542103
0.277542103
0.277579734
0.278014882
0.278014882
0.278225275
0.278270017
0.278335389
0.278335389
0.278335389
0.278335389
0.278335389
0.278335389
0.278335389
0.278335389
0.278335389
0.278335389

0.278335389
0.278335389
0.278335389
0.278335389
0.278335389
0.278335389
0.278335389
0.278335389
0.278650534
0.279246129
0.279409677
0.279723776
0.279903408
0.280141592
0.280686425
0.281032507
0.281032507
0.281298644
0.281588123
0.281713792
0.281789382
0.281813107
0.282185998
0.282422306
0.282422306
0.282422306
0.282422306
0.282422306
0.282422306
0.282422306
0.282422306
0.282422306
0.282422306
0.282422306
0.282422306
0.282805607
0.282805607

0.282805607
0.282950103
0.283085664
0.284279994
0.284625978
0.284625978
0.284986143
0.285109821
0.285109821
0.285109821
0.285109821
0.285109821
0.285109821
0.285176027
0.285280962
0.285498954
0.285984216
0.285984216
0.286113722
0.286113722
0.286113722
0.286113722
0.286113722
0.286507145
0.28660082
0.287205672
0.287233249
0.287687872
0.287753791
0.287753791
0.287753791
0.287753791
0.287873074
0.287931436
0.287972922
0.288185765
0.288185765

0.288185765
0.288185765
0.288185765
0.288185765
0.288306527
0.288422483
0.288422483
0.288580481
0.288580481
0.288580481
0.288580481
0.288580481
0.288580481
0.289183761
0.289183761
0.289183761
0.289432497
0.289432497
0.289432497
0.289432497
0.289432497
0.289432497
0.289432497
0.289432497
0.289432497
0.289432497
0.289432497
0.289432497
0.289432497
0.289432497
0.289432497
0.289432497
0.289432497
0.289432497
0.289432497
0.289432497
0.289432497

0.289432497
0.289432497
0.289432497
0.289436968
0.28983752
0.28983752
0.289905494
0.289917328
0.289917328
0.290031018
0.290031018
0.290031018
0.290139174
0.290139174
0.290142968
0.290142968
0.290142968
0.290266615
0.290417704
0.290488932
0.290488932
0.291031385
0.291031385
0.291031385
0.291031385
0.291031385
0.291031385
0.291031385
0.291031762
0.291040751
0.291167598
0.291467086
0.291467086
0.291604126
0.291604126
0.291624725
0.291624725

0.291624725
0.291624725
0.292303226
0.292303226
0.292910812
0.293239828
0.293239828
0.293239828
0.293239828
0.293239828
0.293239828
0.293239828
0.293239828
0.293249294
0.293737522
0.293737522
0.293737522
0.294092064
0.294092064
0.294092064
0.294525464
0.294525464
0.294749965
0.295059739
0.295059739
0.295110598
0.295188066
0.295269765
0.295681195
0.295681195
0.295826014
0.295924852
0.295924852
0.296155207
0.296155207
0.296155207
0.296155207

0.296769634
0.297167731
0.297887978
0.297887978
0.297887978
0.297887978
0.298099293
0.298099293
0.298099293
0.298099293
0.298099293
0.298099293
0.298099293
0.298099293
0.298099293
0.298099293
0.298099293
0.298099293
0.298099293
0.298099293
0.298099293
0.298099293
0.298099293
0.298099293
0.298099293
0.298099293
0.298099293
0.298099293
0.298099293
0.298099293
0.298099293
0.298099293
0.298099293
0.298099293
0.298099293
0.298099293

0.298099293
0.298099293
0.298099293
0.298099293
0.298099293
0.298099293
0.298099293
0.298099293
0.298099293
0.298099293
0.298099293
0.298099293
0.298099293
0.298099293
0.298099293
0.298099293
0.298099293
0.298099293
0.298099293
0.298099293
0.298099293
0.298099293
0.298099293
0.298099293
0.298650631
0.298650631
0.298692736
0.298692736
0.298692736
0.298692736
0.298692736
0.298692736
0.298692736
0.298692736
0.298716485
0.298716485
0.298716485

0.298716485
0.298716485
0.298716485
0.298716485
0.298716485
0.298756133
0.298756133
0.298756133
0.299183499
0.299486516
0.300095117
0.300345307
0.300779616
0.300779616
0.300894359
0.30128006
0.30128006
0.30128006
0.30128006
0.301421446
0.301906993
0.301906993
0.302329271
0.302347016
0.303252183
0.303252183
0.303252183
0.303252183
0.303915065
0.303915065
0.303915065
0.303980684
0.304069883
0.304069883
0.304589295
0.304870326
0.305072594

0.305072594
0.305171341
0.305580992
0.305580992
0.305998718
0.306038435
0.306051636
0.306096617
0.306533703
0.306533703
0.306533703
0.306533703
0.306533703
0.306533703
0.306533703
0.306533703
0.306601527
0.306601527
0.306601527
0.306601527
0.306938953
0.307255602
0.307281963
0.307281963
0.307527398
0.307527398
0.307527398
0.307761804
0.307786126
0.308060515
0.308060515
0.309101647
0.309209868
0.309356348
0.309356348
0.309478994
0.309478994

0.309478994
0.309478994
0.309478994
0.309478994
0.309478994
0.309478994
0.309478994
0.309862701
0.310200144
0.310431486
0.310431486
0.310431486
0.310431486
0.310431486
0.310431486
0.310431486
0.310474097
0.310618596
0.310618596
0.310846015
0.310866138
0.310866138
0.310920115
0.311660889
0.311743119
0.311743119
0.312207894
0.312207894
0.312207894
0.312207894
0.312207894
0.312316315
0.312316315
0.312316315
0.312316315
0.312316315
0.312316315

0.312316315
0.312316315
0.312316315
0.312316315
0.312362022
0.312362022
0.312389372
0.312389372
0.312389372
0.312389372
0.312389372
0.312446467
0.312447093
0.312678796
0.313015962
0.313015962
0.313015962
0.313412975
0.313958984
0.313967694
0.314307122
0.314481475
0.314481475
0.314481475
0.314481475
0.314481475
0.314481475
0.314481475
0.314481475
0.314495733
0.314499751
0.314499751
0.314499751
0.314499751
0.314499751
0.31514053
0.31514053

0.315727937
0.315875676
0.316168683
0.316168683
0.316696028
0.31720349
0.317235431
0.317362233
0.317362233
0.317675314
0.317804499
0.318182347
0.318300364
0.318483612
0.318483612
0.318483612
0.319272611
0.319738018
0.319790489
0.319971004
0.32003451
0.32003451
0.32003451
0.320779662
0.320779662
0.320779662
0.320779662
0.320900233
0.321057319
0.321234787
0.321476737
0.321713495
0.322183156
0.322218696
0.322218696
0.322218696
0.322218696

0.322218696
0.322218696
0.322218696
0.322364764
0.322433175
0.322963036
0.322980416
0.323250771
0.323510162
0.323510162
0.323582085
0.323922259
0.323922259
0.323932965
0.323932965
0.324157518
0.324265432
0.325280342
0.325280342
0.325280342
0.325280342
0.325280342
0.325280342
0.325506207
0.325506207
0.326010142
0.326077966
0.326077966
0.326077966
0.326077966
0.326077966
0.326201808
0.32639959
0.32639959
0.32639959
0.32641301
0.326805313

0.32745082
0.32745082
0.32745082
0.32756112
0.327661707
0.327784752
0.32790025
0.32790025
0.32790025
0.32790025
0.328200299
0.328200299
0.328200299
0.328200299
0.328706323
0.329465595
0.329485274
0.329485274
0.329485274
0.329536546
0.329651447
0.329675787
0.32984478
0.32984478
0.32984478
0.32984478
0.329863765
0.329954702
0.330231868
0.331160729
0.331160729
0.331160729
0.331197148
0.331216755
0.331484852
0.331685512
0.332160576

0.332160576
0.332173744
0.332363714
0.332363714
0.332363714
0.332555063
0.332555063
0.332555063
0.332608539
0.332608539
0.332620699
0.332620699
0.332620699
0.33322968
0.333311092
0.333311092
0.333311092
0.334291225
0.334781567
0.334781567
0.334781567
0.334781567
0.334781567
0.334781567
0.334781567
0.334805818
0.334805818
0.334805818
0.334805818
0.334805818
0.334805818
0.334805818
0.334914741
0.334914741
0.335223353
0.335223353
0.335568428

0.335738358
0.336054995
0.336054995
0.336054995
0.336110581
0.336268858
0.336392987
0.336420124
0.336469904
0.336786223
0.336792722
0.33684731
0.337758624
0.338198098
0.338255966
0.338255966
0.338255966
0.338255966
0.338255966
0.338255966
0.338569255
0.338569255
0.338617717
0.338617717
0.338617717
0.338617717
0.338617717
0.338661601
0.338661601
0.338661601
0.339652753
0.340228996
0.340228996
0.340228996
0.340228996
0.340228996
0.340360556

0.341327818
0.34162765
0.34162765
0.3416683
0.342399486
0.342399486
0.342399486
0.342399486
0.342399486
0.342399486
0.342446605
0.342636822
0.342636822
0.342636822
0.342753609
0.343355599
0.343895751
0.343895751
0.343895751
0.343895751
0.344089054
0.344089054
0.344164995
0.344337869
0.344437991
0.344829996
0.3448911
0.345056094
0.345175942
0.345345819
0.345845225
0.345845225
0.347520539
0.347583065
0.347583065
0.347617342
0.347617342

0.347617342
0.347617342
0.347617342
0.347617342
0.34769643
0.34769643
0.347859962
0.348103627
0.348103627
0.348103627
0.348103627
0.348103627
0.348103627
0.348103627
0.348489547
0.348775046
0.348963554
0.349114276
0.349321139
0.349585766
0.349585766
0.349585766
0.349585766
0.349585766
0.349585766
0.349585766
0.349585766
0.349585766
0.349801128
0.350091763
0.350216579
0.350216579
0.350284994
0.350403482
0.35054561
0.35054561
0.350744818

0.350744818
0.350744818
0.350859035
0.351149532
0.351149532
0.351149532
0.351605686
0.351605686
0.351955814
0.351955814
0.352217132
0.352522663
0.352576515
0.352614144
0.35313894
0.35313894
0.35313894
0.353332407
0.353362743
0.35395814
0.354012396
0.354012396
0.354012396
0.354012396
0.354012396
0.354012396
0.354404524
0.354719408
0.354967167
0.354967167
0.354967167
0.355078648
0.355078648
0.355234049
0.355234049
0.355234049
0.355234049

0.355234049
0.355234049
0.355234049
0.355234049
0.355234049
0.355234049
0.355234049
0.355234049
0.355234049
0.355234049
0.355518041
0.355563838
0.355563838
0.355563838
0.355563838
0.356615221
0.356615221
0.356615221
0.356615221
0.356615221
0.356615221
0.356615221
0.357188697
0.357202298
0.35742731
0.35742731
0.35742731
0.35742731
0.35742731
0.35742731
0.35742731
0.35742731
0.35742731
0.35742731
0.35742731
0.35742731
0.35742731

0.35742731
0.35742731
0.35742731
0.35742731
0.35742731
0.35742731
0.35742731
0.35742731
0.35742731
0.35742731
0.35742731
0.35742731
0.35742731
0.35742731
0.35742731
0.35742731
0.35742731
0.35742731
0.35742731
0.35742731
0.35742731
0.35742731
0.35742731
0.35742731
0.35742731
0.35742731
0.35742731
0.35742731
0.35742731
0.35742731
0.35742731
0.35742731
0.35742731
0.35742731
0.35742731
0.35742731
0.35742731

0.35742731
0.35742731
0.35742731
0.35742731
0.35742731
0.35742731
0.35742731
0.35742731
0.35742731
0.35742731
0.35742731
0.35742731
0.35742731
0.35742731
0.35742731
0.35742731
0.35742731
0.35742731
0.35742731
0.357439492
0.357439492
0.357643779
0.35790694
0.35790694
0.35790694
0.358066871
0.358066871
0.358066871
0.358111277
0.358268231
0.358268231
0.358458144
0.358458144
0.358458144
0.358458144
0.358458144
0.358458144

0.358458144
0.358458144
0.358515756
0.358515756
0.358515756
0.358515756
0.358515756
0.358515756
0.358515756
0.358515756
0.358515756
0.358515756
0.358515756
0.358515756
0.358515756
0.358515756
0.358515756
0.358515756
0.358515756
0.358539518
0.358544441
0.358544441
0.358544441
0.358793012
0.358819589
0.358888972
0.358888972
0.358888972
0.358888972
0.358888972
0.358888972
0.358990606
0.359549979
0.359549979
0.359549979
0.359549979
0.359866886

0.359910054
0.359910054
0.360138326
0.360138326
0.360138326
0.360138326
0.360354015
0.361116983
0.361364461
0.361565805
0.361565805
0.361565805
0.361709483
0.361709483
0.36175331
0.36175331
0.36175331
0.36175331
0.36175331
0.36175331
0.361768228
0.361768228
0.361768228
0.361768228
0.361768228
0.361768228
0.361768228
0.361768228
0.361768228
0.361953375
0.362239429
0.362239429
0.363152989
0.363152989
0.363152989
0.363152989
0.363191922

0.363191922
0.363191922
0.364240733
0.364293049
0.364311861
0.364311861
0.364311861
0.364528536
0.364914095
0.364914095
0.365043034
0.365043034
0.365043034
0.365135495
0.365135495
0.365618956
0.365757261
0.365757261
0.365985492
0.366517388
0.366517388
0.366517388
0.367097293
0.367097293
0.367257999
0.367483776
0.367483776
0.367483776
0.367832343
0.367832343
0.367832343
0.367832343
0.367975731
0.368065191
0.368067852
0.368720078
0.36879542

0.36879542
0.36879542
0.36879542
0.36879542
0.36879542
0.36879542
0.36879542
0.36879542
0.36879542
0.36911629
0.36911629
0.37020383
0.370239266
0.370239266
0.370239266
0.370425868
0.370811486
0.370913156
0.371268729
0.371268729
0.371443135
0.371674171
0.37217987
0.37217987
0.37217987
0.37217987
0.37217987
0.37217987
0.37217987
0.372183741
0.372183741
0.372183741
0.37256364
0.37279743
0.373050445
0.37317414
0.373977229

0.374167085
0.37463428
0.37463428
0.37463428
0.375600607
0.375666144
0.375666144
0.375995852
0.376096876
0.376096876
0.37622246
0.376346475
0.376690396
0.376934537
0.377600219
0.377846252
0.378391481
0.378391481
0.378391481
0.378391481
0.378856174
0.378856174
0.379578726
0.379634556
0.379634556
0.379779493
0.379909752
0.379909752
0.379909752
0.379909752
0.379909752
0.380253178
0.380253178
0.380253178
0.380322258
0.380717777
0.381015681

0.381153917
0.381173785
0.381173785
0.381173785
0.381173785
0.381173785
0.381173785
0.381173785
0.381173785
0.381173785
0.381173785
0.381173785
0.381173785
0.381173785
0.38119185
0.381289039
0.381289039
0.381353156
0.381353156
0.381353156
0.381353156
0.381353156
0.38137929
0.38137929
0.381588538
0.381588538
0.381591784
0.381733194
0.381776213
0.381776213
0.381776213
0.381776213
0.381789304
0.381789304
0.381789304
0.381789304
0.381789304

0.381789304
0.381789304
0.381789304
0.381789304
0.382489421
0.382489421
0.382974842
0.382974842
0.382974842
0.383167795
0.383184376
0.384061596
0.384061596
0.385075099
0.385075099
0.385075099
0.385075099
0.385075099
0.385094371
0.385094371
0.385094371
0.385094371
0.38522892
0.385457208
0.385457208
0.385457208
0.385457208
0.385457208
0.385457208
0.385457208
0.385457208
0.385457208
0.385573651
0.385573651
0.385573651
0.385573651
0.385573651

0.385573651
0.385573651
0.385877238
0.386662492
0.386662492
0.386816589
0.386980255
0.386980255
0.386980255
0.386994413
0.387110488
0.387110488
0.387110488
0.387110488
0.387110488
0.387110488
0.387110488
0.388188156
0.388249869
0.388413687
0.388413687
0.38860367
0.388665237
0.388860379
0.388860379
0.388879412
0.389268667
0.389268667
0.389533125
0.389533125
0.389535218
0.389944787
0.390527946
0.390658325
0.390733466
0.390926154
0.391032774

0.392276713
0.392346465
0.39254919
0.39254919
0.39254919
0.39254919
0.39254919
0.39254919
0.39254919
0.39254919
0.39254919
0.39254919
0.39254919
0.39254919
0.39254919
0.39254919
0.39254919
0.39254919
0.392817704
0.392943777
0.392943777
0.392998814
0.393032048
0.393576449
0.393832966
0.393838821
0.393838821
0.393838821
0.393838821
0.393924147
0.393924147
0.393924147
0.393924147
0.394467568
0.394467568
0.394467568
0.394467568

0.394467568
0.394467568
0.394467568
0.394467568
0.394494106
0.394494106
0.394494106
0.394864077
0.395318116
0.395318116
0.395424194
0.395427595
0.39550967
0.39550967
0.39550967
0.39550967
0.39550967
0.39550967
0.39550967
0.39550967
0.39550967
0.39550967
0.39550967
0.39550967
0.39550967
0.39550967
0.39550967
0.39550967
0.39550967
0.395694245
0.395755778
0.395755778
0.395923518
0.395940949
0.396180331
0.396180331
0.396489965

0.396712662
0.396712662
0.397407806
0.397407806
0.397668688
0.397866146
0.397866146
0.397866146
0.397866146
0.398117844
0.398333637
0.398333637
0.398333637
0.398895558
0.39900022
0.399087214
0.399087214
0.399087214
0.399597585
0.399597585
0.399608871
0.399608871
0.399608871
0.399608871
0.399608871
0.399608871
0.399608871
0.399608871
0.399763489
0.399763489
0.400134107
0.400476327
0.400476327
0.400531395
0.400531395
0.400531395
0.400531395

0.40112764
0.401294923
0.401360515
0.401360515
0.401422945
0.401422945
0.401422945
0.401422945
0.401454829
0.401465689
0.401465689
0.401465689
0.401465689
0.401866451
0.401880226
0.401880226
0.402077583
0.402255528
0.402255528
0.402349727
0.402924774
0.402924774
0.402924774
0.402924774
0.402924774
0.402924774
0.402924774
0.402924774
0.40307537
0.403212803
0.403212803
0.403389807
0.403394464
0.403946726
0.403946726
0.403946726
0.403946726

0.404344152
0.404344152
0.404344152
0.404653723
0.404653723
0.404810102
0.404922767
0.404922767
0.405069107
0.405079837
0.405079837
0.405988583
0.40645384
0.40645384
0.40645384
0.406818568
0.406995252
0.406995252
0.406995252
0.406995252
0.406995252
0.406995252
0.408018618
0.408018618
0.408237819
0.408237819
0.408237819
0.408324428
0.408324428
0.408324428
0.408563416
0.408588404
0.409126511
0.409126511
0.409244722
0.409244722
0.409255406

0.409953709
0.409953709
0.409953709
0.409953709
0.410152731
0.410152731
0.410152731
0.410152731
0.410152731
0.410152731
0.410262648
0.410262648
0.410262648
0.410292562
0.410292562
0.410292562
0.410292562
0.410292562
0.410486092
0.410514929
0.411016831
0.411016831
0.411144663
0.411206562
0.4112298
0.4112298
0.4112298
0.4112298
0.4112298
0.4112298
0.411593666
0.411919809
0.411989009
0.412046004
0.412225112
0.412225112
0.412225112

0.412225112
0.412225112
0.412225112
0.412225112
0.412225112
0.412225112
0.412225112
0.412225112
0.412225112
0.412225112
0.412225112
0.412378278
0.412392136
0.412491439
0.413126366
0.413178237
0.413178237
0.413533734
0.413673276
0.413783184
0.413783184
0.413969279
0.414118376
0.414118376
0.41413687
0.414339894
0.414493914
0.415134211
0.415300303
0.415300303
0.415515049
0.415802461
0.416153067
0.416244829
0.416340679
0.416440437
0.416440437

0.416440437
0.416690911
0.416831683
0.416831683
0.416831683
0.416831683
0.416831683
0.416831683
0.416831683
0.416831683
0.416831683
0.416831683
0.416831683
0.416831683
0.416831683
0.416944049
0.417162448
0.417610674
0.417610674
0.417610674
0.417610674
0.417614354
0.418598401
0.419070043
0.419277454
0.419277454
0.419277454
0.419277454
0.419277454
0.419277454
0.419641745
0.419641745
0.419641745
0.420038503
0.420081311
0.420840295
0.420840295

0.420840295
0.420840295
0.420840295
0.420840295
0.420840295
0.420871425
0.421230377
0.421236169
0.421513658
0.422173539
0.422234953
0.422268737
0.423453957
0.423516823
0.423779482
0.423779482
0.423779482
0.425037013
0.425293496
0.425293496
0.425293496
0.425293496
0.425306897
0.425306897
0.425306897
0.425306897
0.425935953
0.425935953
0.426147167
0.426147167
0.426163471
0.426628833
0.426714825
0.426714825
0.427202302
0.427407359
0.427577192

0.427577192
0.427577847
0.427577847
0.42798375
0.42798375
0.428311156
0.428383445
0.428383445
0.428383445
0.428503999
0.428572587
0.428572587
0.428781523
0.428931048
0.428931048
0.428931048
0.428931048
0.428931048
0.428931048
0.428931048
0.428931048
0.428931048
0.428931048
0.428931048
0.429116763
0.429116763
0.429592619
0.429592619
0.430128706
0.430130604
0.430147101
0.430147101
0.430147101
0.430290328
0.430341417
0.430341417
0.43054841

0.43056882
0.431113903
0.431113903
0.431113903
0.431113903
0.431113903
0.431818778
0.431909341
0.432167731
0.432167731
0.432167731
0.432167731
0.432167731
0.432167731
0.432167731
0.432167731
0.432167731
0.432167731
0.432167731
0.432167731
0.432167731
0.432167731
0.432167731
0.432167731
0.432167731
0.432167731
0.432362253
0.432708251
0.433071099
0.433237355
0.43334402
0.433744974
0.433744974
0.433744974
0.433763554
0.434123625
0.434123625

0.434144777
0.434144777
0.434144777
0.434460867
0.434460867
0.434460867
0.434499992
0.434723418
0.434723418
0.434723418
0.435071324
0.435071324
0.435071324
0.435071324
0.435071324
0.435071324
0.435071324
0.435071324
0.435071324
0.435071324
0.435071324
0.435071324
0.435071324
0.435071324
0.435451937
0.435451937
0.435451937
0.43573958
0.437053113
0.437053113
0.437053113
0.43705584
0.437529346
0.437694415
0.437911628
0.438139784

0.438197889
0.438197889
0.438197889
0.438238747
0.438759872
0.43889638
0.43889638
0.438920301
0.439713794
0.439791712
0.439791712
0.44002998
0.44002998
0.440252574
0.440400489
0.440584903
0.440920098
0.440998394
0.440998394
0.440998394
0.440998394
0.441008163
0.441256976
0.441256976
0.44132805
0.44132805
0.44132805
0.44132805
0.44132805
0.441435531
0.441494918
0.441494918
0.441494918
0.441494918
0.441494918
0.441494918
0.441494918

0.441494918
0.441519674
0.44154903
0.44154903
0.442184439
0.442882799
0.442882799
0.443272195
0.443421458
0.443439754
0.443684297
0.443924012
0.443924012
0.443924012
0.443924012
0.443924012
0.443924012
0.443999027
0.443999027
0.443999027
0.444022134
0.444270783
0.444270783
0.444959121
0.445538574
0.445538574
0.445538574
0.445566809
0.445566809
0.445566809
0.44603844
0.44603844
0.44603844
0.44603844
0.44603844
0.44603844
0.446109527

0.446109527
0.446759208
0.446902122
0.447053143
0.447419934
0.447550469
0.447747097
0.447747097
0.447747097
0.447747097
0.447747097
0.447747097
0.447747097
0.447747097
0.447788427
0.447788427
0.448056242
0.448555349
0.448555349
0.449060571
0.449060571
0.449060571
0.449060571
0.449060571
0.449556896
0.450068048
0.450068048
0.450594242
0.450594242
0.450594242
0.450813932
0.450813932
0.450813932
0.450813932
0.45092095
0.45092095
0.451771022

0.451771022
0.451771022
0.45178685
0.451886052
0.452379643
0.452379643
0.452379643
0.452379643
0.452930317
0.453551457
0.453699589
0.453699589
0.453699589
0.453699589
0.453699589
0.453782752
0.453782752
0.453831764
0.454399965
0.454612405
0.454852434
0.454852434
0.454852434
0.455262883
0.455664748
0.455664748
0.455664748
0.455664748
0.455721447
0.455721447
0.455920744
0.455920744
0.455920744
0.455920744
0.455920744
0.455920744
0.456110636

0.456823857
0.456823857
0.456967258
0.457031921
0.457031921
0.457046207
0.457046207
0.457046207
0.457046207
0.457140456
0.45735619
0.457481341
0.457481341
0.457481341
0.457481341
0.457543877
0.457543877
0.457543877
